# A multiple genome analysis of *Mycobacterium tuberculosis* reveals specific novel genes and mutations associated with pyrazinamide resistance

**DOI:** 10.1186/s12864-017-4146-z

**Published:** 2017-10-11

**Authors:** Patricia Sheen, David Requena, Eduardo Gushiken, Robert H. Gilman, Ricardo Antiparra, Bryan Lucero, Pilar Lizárraga, Basilio Cieza, Elisa Roncal, Louis Grandjean, Arnab Pain, Ruth McNerney, Taane G. Clark, David Moore, Mirko Zimic

**Affiliations:** 1 Laboratorio de Bioinformática y Biología Molecular. Laboratorios de Investigación y Desarrollo, Facultad de Ciencias y Filosofía, Universidad Peruana Cayetano Heredia, Av. Honorio Delgado 430, San Martín de Porras, 31 Lima, Peru; 2 Department of International Health, Johns Hopkins Bloomberg School of Public Health, 615 North Wolfe St., Room 5515, Baltimore, MD 21205 USA; 3 Department of Infection, Immunology and Rheumatology, Institute of Child Health, University College London, 30 Guilford St, London, WC1N 1EH UK; 4 Biological and Environmental Sciences and Engineering Division, King Abdullah University of Science & Technology, Thuwal, Kingdom of Saudi Arabia; 5 Faculty of Infectious and Tropical Diseases, London School of Hygiene & Tropical Medicine, London, WC1E 7HT UK; 6 Faculty of Epidemiology and Population Health, London School of Hygiene & Tropical Medicine, London, WC1E 7HT UK

**Keywords:** Tuberculosis, Pyrazinamide, Resistance, Genome, Mutations, MDR, Metallochaperone, Efflux pump, Genes, Drugs

## Abstract

**Background:**

Tuberculosis (TB) is a major global health problem and drug resistance compromises the efforts to control this disease. Pyrazinamide (PZA) is an important drug used in both first and second line treatment regimes. However, its complete mechanism of action and resistance remains unclear.

**Results:**

We genotyped and sequenced the complete genomes of 68 *M. tuberculosis* strains isolated from unrelated TB patients in Peru. No clustering pattern of the strains was verified based on spoligotyping. We analyzed the association between PZA resistance with non-synonymous mutations and specific genes.

We found mutations in *pncA* and novel genes significantly associated with PZA resistance in strains without *pncA* mutations. These included genes related to transportation of metal ions, pH regulation and immune system evasion.

**Conclusions:**

These results suggest potential alternate mechanisms of PZA resistance that have not been found in other populations, supporting that the antibacterial activity of PZA may hit multiple targets.

**Electronic supplementary material:**

The online version of this article (10.1186/s12864-017-4146-z) contains supplementary material, which is available to authorized users.

## Background

Tuberculosis (TB) caused by the *Mycobacterium tuberculosis* bacilli is one of the most important global infectious diseases. Approximately one third of the global population has latent TB infection, and 10% of infected people will eventually develop active TB disease in their lifetime [1].

While other first line anti-TB drugs affect fast growing metabolically active bacilli, pyrazinamide (PZA) is particularly effective against non-replicating persistent. *M. tuberculosis*. This unique feature facilitates the shortening of the duration of TB chemotherapy including PZA from 12 to 6 months [2]. The mechanism of action and resistance to PZA is not completely understood. PZA is a pro-drug which enters into *M. tuberculosis* by passive diffusion [3], and it is converted into its active form, pyrazinoic acid (POA) by the action of pyrazinamidase (PZAse) [4]. POA is released into extracellular space by an efflux system, where it is protonated to HPOA provided that the extracellular environment is acidic [3]. External HPOA diffuses back across the membrane of *M. tuberculosis* through an electrical potential gradient, releasing the proton in the cytosol. The combination of POA accumulation of POA protonation of the cytosol produces a lethal effect through the disruption of membrane permeability and reduced membrane potential. This affects transport of nutrients [5], and interference of trans-translation by binding to the ribosomal protein *RpsA* [6] and competing with the tmRNA [7].

It was clearly demonstrated that loss of *PZAse* activity highly correlates with PZA resistance in *M. tuberculosis* [4, 8–10]. PZAse malfunction is caused by mutations in its coding gene (*pncA*) [11], as well as in its promoter region compromising the expression of the protein [12], resulting in lack of conversion of PZA into POA [13]. Previous studies have demonstrated that POA efflux rate varies amongst *M. tuberculosis* strains [14] and its quantitative variability is significantly associated with the level of PZA resistance [15].

Although PZA resistance in *M. tuberculosis* is mostly associated with mutations in pncA, other genes have recently been found involved in the mechanism of action of PZA [16], while there are potential other factors that may alter the PZA susceptibility. These include factors for ROS-induced mutagenesis, selective treatment pressure or immune evasion genes [17].

In the present study, we sequenced and annotated the complete genomes from 68 strains of *M. tuberculosis* isolated from Peruvian TB patients, with known PZA susceptibility. We analyzed the statistical association between PZA resistance with specific mutations and genes. Particular attention was given to specific gene families with potential biological relationship with the PZA mechanism of action.

## Methods

### *M. tuberculosis* samples and PZA resistance

We selected well-characterized strains from our *M. tuberculosis* strains bank. A total of 68 *M. tuberculosis* clinical isolates recovered from different unrelated patients from Lima, Peru with active tuberculosis and with previous PZA susceptibility results were selected for genome analysis and further studies. From these 68 strains, 26 strains were PZA resistant and 42 strains were PZA susceptible (Table 1). 29 strains had BACTEC 460 TB susceptibility (21 resistant and 8 susceptible). Forty-six strains had BACTEC 960-MGIT susceptibility (10 resistant and 36 susceptible). Five strains were resistant in BACTEC 960-MGIT and in BACTEC 460 TB. Two strains were susceptible in BACTEC 960-MGIT and in BACTEC 460 TB. No one strain had a discrepant result between BACTEC 960-MGIT and in BACTEC 460 TB.

**Table 1:** Strains analyzed in the study, experimental test for resistance and mutations in pyrazinamidase

	Resistance profile			Mutations in pncA/PZAse		
Isolate	BACTEC 460 TB	BACTEC 960 TB-MGIT	Res PZA	Gene	Protein	Promoter
CSV4519	R	–	**1**	–	–	–
CSV4644	R	–	**1**	T152C	H51R	–
CSV5769	R	R	**1**	C232A	G78C	–
CSV10399	R	R	**1**	A280G	F94 L	–
CSV11678	R	–	**1**	G185A	P62L	–
LE486	R	–	**1**	T35G	D12A	–
LE492	–	R	**1**	T35G	D12A	–
LN180	–	R	**1**	A392C	V131G	–
LN2358	–	R	**1**	A545C	L182 W	–
LN3756	R	–	**1**	T403G	T135P	–
MDRDM260	–	R	**1**	T403G	T135P	–
MDRDM627	–	R	**1**	–	–	–
MDRDM1098	R	R	**1**	C145T	D49N	–
MDRMA2491	R	R	**1**	T35C	D12G	–
ME1473	R	–	**1**	A100C	Y34D	–
TBDM425	R	–	**1**	C145T	D49N	–
TBV5000	R	–	**1**	–	–	–
TBV5362	R	–	**1**	–	–	–
TBV5365	R	–	**1**	–	–	–
SLM036	R	R	**1**	A190C	Y64D	–
SLM040	R	–	**1**	T170C	H57R	–
SLM056	R	–	**1**	–	–	–
SLM060	R	–	**1**	–	–	T-11C
SLM063	R	–	**1**	A416G	V139A	–
SLM088	R	–	**1**	–	–	–
SLM100	R	–	**1**	–	–	T-11C
CSV383	S	–	**0**	A40C	C14G	
CSV3611	S	–	**0**	–	–	
CSV9577	S	–	**0**	–	–	
LE13	–	S	**0**	T478C	T160A	
LE63	–	S	**0**	–	–	
LE76	–	S	**0**	–	–	
LE79	–	S	**0**	–	–	
LE103	S	S	**0**	–	–	
LE371	–	S	**0**	–	–	
LE410	–	S	**0**	–	–	
LN55	–	S	**0**	A40C	C14G	
LN317	–	S	**0**	–	–	
LN763	–	S	**0**	–	–	
LN2978	–	S	**0**	–	–	
LN3584	–	S	**0**	–	–	
LN3588	–	S	**0**	–	–	
LN3589	–	S	**0**	–	–	
LN3668	–	S	**0**	–	–	
LN3672	–	S	**0**	–	–	
LN3695	–	S	**0**	–	–	
LN1100	–	S	**0**	–	–	
LN1856	S	S	**0**	T143G	K48 T	
LN2900	–	S	**0**	–	–	
MDRDM827	–	S	**0**	–	–	
MDRMA203	–	S	**0**	–	–	
MDRMA701	–	S	**0**	–	–	
MDRMA863	–	S	**0**	–	–	
MDRMA1565	–	S	**0**	–	–	
MDRMA2019	–	S	**0**	–	–	
MDRMA2082	–	S	**0**	–	–	
MDRMA2260	–	S	**0**	–	–	
MDRMA2441	–	S	**0**	–	–	
TBDM1506	–	S	**0**	–	–	
TBDM2189	–	S	**0**	–	–	
TBDM2444	–	S	**0**	–	–	
TBDM2487	–	S	**0**	–	–	
TBDM2489	–	S	**0**	–	–	
TBDM2699	–	S	**0**	–	–	
TBDM2717	–	S	**0**	–	–	
TBV4766	S	–	**0**	–	–	
TBV4768	S	–	**0**	–	–	
TBV4952	S	–	**0**	–	–	

We are only showing the non-synonymous mutations in the gene *pncA* and its promoter, and the corresponding amino acid change in the protein pyrazinamidase

### Genotyping and phylogenetic analysis

Spoligotyping films were blindly interpreted by two different readers. Reads were repeated when a discrepancy was detected. To identify the relationship of strains, and to verify that no bias due to the presence of particular genomic variants is present, a phylogenetic analysis was performed to identify clusters and orphan strains. The lineage (clade and SIT code, (shared-type number corresponding to a spoligotype)) of each strain was identified by the classification of the 43-digits spoligotyping code defined in the SITVIT database (Institut Pasteur of Guadeloupe). To show the strain diversity, strains were categorized in groups according to their clades and SIT codes. A distance-tree of the spoligotypes was constructed using the multi-state discrete-characters parsimony method of the software Phylip v 3.695. The distribution of spoligotypes, stratified by susceptible/resistance strains, was analyzed in a histogram with standard errors.

### Genome sequencing and data processing

Genomic DNA was extracted from *M. tuberculosis* strains by the proteinase K digestion method as described previously [18]. DNA from samples were sequenced at the King Abdullah University of Science and Technology (KAUST) using Illumina HiSeq2000 paired end technology, yielding an average of nineteen million 76 bp reads (IQR 9–23 million) per genome. High quality sequencing reads (>Q30) were assembled by reference (*M. tuberculosis* H37Rv reference genome: GenBank NC_000962), using NextGENe (version 2.2.0, SoftGenetics), with default parameters. Chimeric and low quality reads (less than Q30), were discarded to reduce the likelihood of assembly errors and thus the appearance of false SNPs. After paired end mapping, we obtained 362X fold coverage (IQR 177–463), and 97.7% of each genome was covered with at least 5X fold. Regions of low sequencing coverage (2.3% of genome, less than 5X fold) were ignored. Using the sequence alignments, SNPs and insertions and deletions were identified using NextGENe (version 2.2.0, SoftGenetics). These variants were classified for their occurrence in coding or non-coding regions, and whether mutations led to synonymous or non-synonymous aminoacid changes. Only non-synonymous SNPs and insertions/deletions in coding regions were considered for the subsequent analysis.

### Compilation of a special set of genes

It is currently accepted that POA is expelled outside the mycobacteria by an efflux pump system, however the identity of this pump is not yet known. We compiled a set of reported *M. tuberculosis* efflux pump coding genes, and also a set of homologues to efflux pumps reported in the literature for different organisms, (identity ≥25% in blast).

*M. tuberculosis* PZAse is a metalloenzyme. Our previous studies suggest that PZAse may be activated by a metallochaperone in vivo (to be published). We compiled a set of metallochaperones and divalent ions transporter proteins. Some of them have already been described in other species, including *yodA*, *zinT*, *mntA*, *mntC*, *sitA*, *troA*, *psaA*, *btuF*, *feoB*, *SBP*, *mntH*, *nhlF* and *corA* [19]. Homologues of metallochaperones in *M. tuberculosis* included *Rv2059 and Rv0106*. As metal ion transporters we included the metalloproteins: *Rv2060*, *Rv0106*, *nicT*, *nurA*, *furB, SBP*, *mntH*, and the cation transporter ATPase family: *CtpI*, *CtpE*, *CtpC*, *CtpG* and *CtpV*.

Special attention was given to genes that have been previously proposed as associated to the mechanism of action of PZA: *FAS-1 (Rv2524c)*, *rpsA (Rv1630)*, *gpsL (Rv2783c)*, *mmpL4 (Rv0450c)*, *panD (Rv3601c), hadC (Rv0637)*, *pstC2 (Rv0929)*, *Acyl-CoA synthetase (Rv1683)* and the hypothetical proteins *Rv2731* and *Rv3169* [5, 7].

### Data analysis

Association analyses were performed both at a SNP level and gene level independently.

#### SNP level analysis

For each specific non-synonymous mutation, we compared the proportion of resistant and susceptible strains harboring a particular mutation. For the resistant strains, we found the number of strains with the specific mutation (a) and without the mutation (b). For the susceptible strains we found the number of strains with the specific mutation (c) and without the mutation (d).

Three analyses were performed: (1) a proportion test for a binomial distribution, to compare the frequencies of a particular mutation among the resistant and susceptible strains (proportions a/(a + b) and c/(c + d) respectively); (2) an estimation of the Odds Ratio (ad)/(bc) in order to identify the increased risk of PZA resistance associated to the presence of a particular mutation and; (3) calculation of the Youden Index (sensitivity + specificity −1) [a/(a + c) + d/(b + d) – 1], which determines the overall capacity of the mutation to correctly classify PZA resistance.

#### Gene level analysis

For each annotated gene, we estimated the association between the presence of at least one mutation in a particular gene and PZA resistance. For each gene we calculated the following parameters. For the resistant strains: The number of strains with at least one non-synonymous mutation in this gene (a), and the number of strains with no mutation in this gene (b). Similarly, for the susceptible strains: The number of strains with at least one non-synonymous mutation in this gene (c), and the number of strains with no mutation in this gene (d). We calculated these parameters for every annotated gene of the *M. tuberculosis* genome. Similarly as above, three analyses were performed: (1) a proportion test for a binomial distribution, to compare the frequencies of a particular gene with at least one mutation among the resistant and susceptible strains; (2) estimation of the Odds Ratio in order to identify the increased risk of PZA resistance associated to the presence of at least one mutation in a particular gene; and (3) calculation of the Youden Index, which determines the overall capacity of any mutation in a particular gene to correctly classify PZA resistance. All the statistics were performed with a 5% significance level.

## Results

### Phylogenetic analysis

**Fig. 1:**
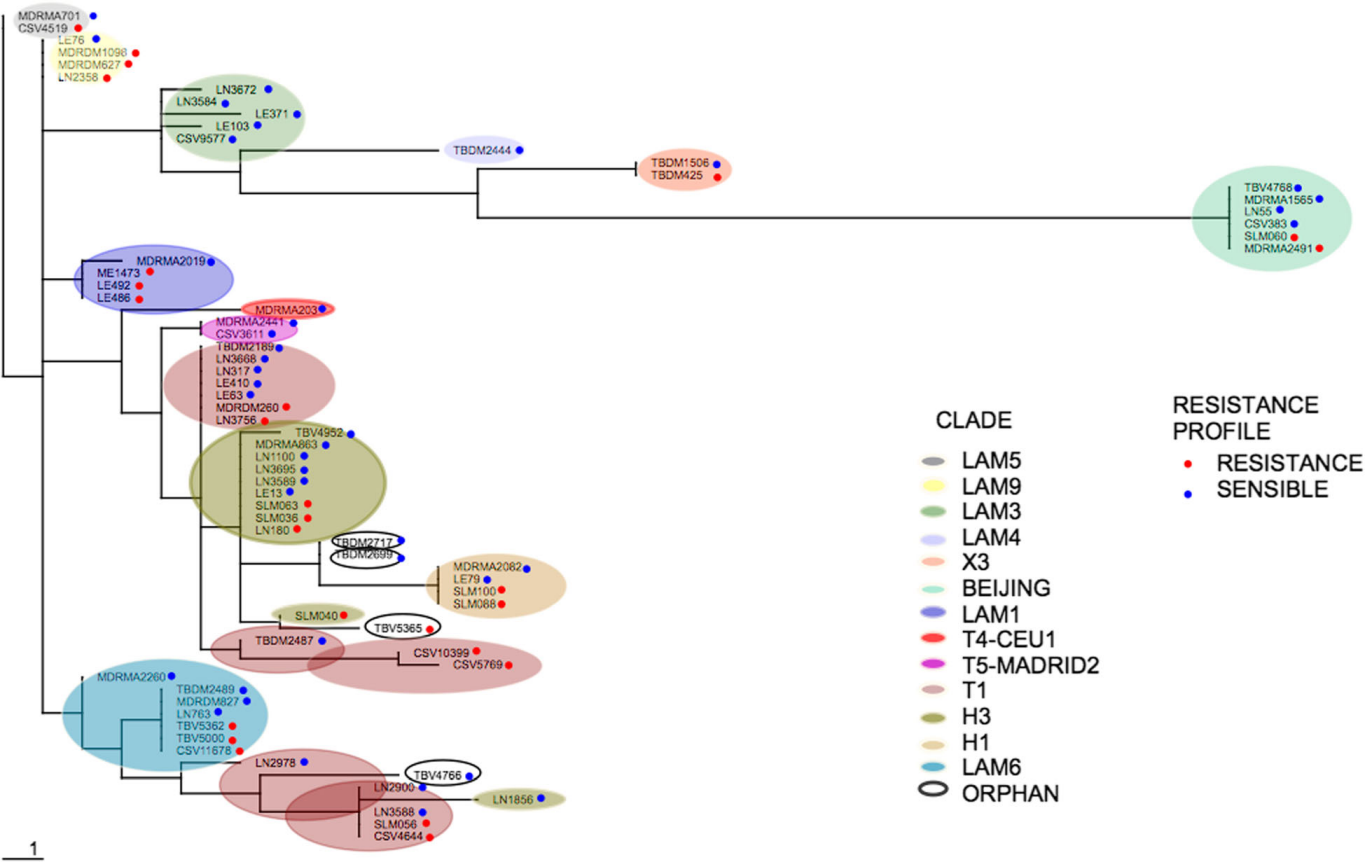
Distances-based tree based on spoligotyping profile of the strains

**Fig. 2:**
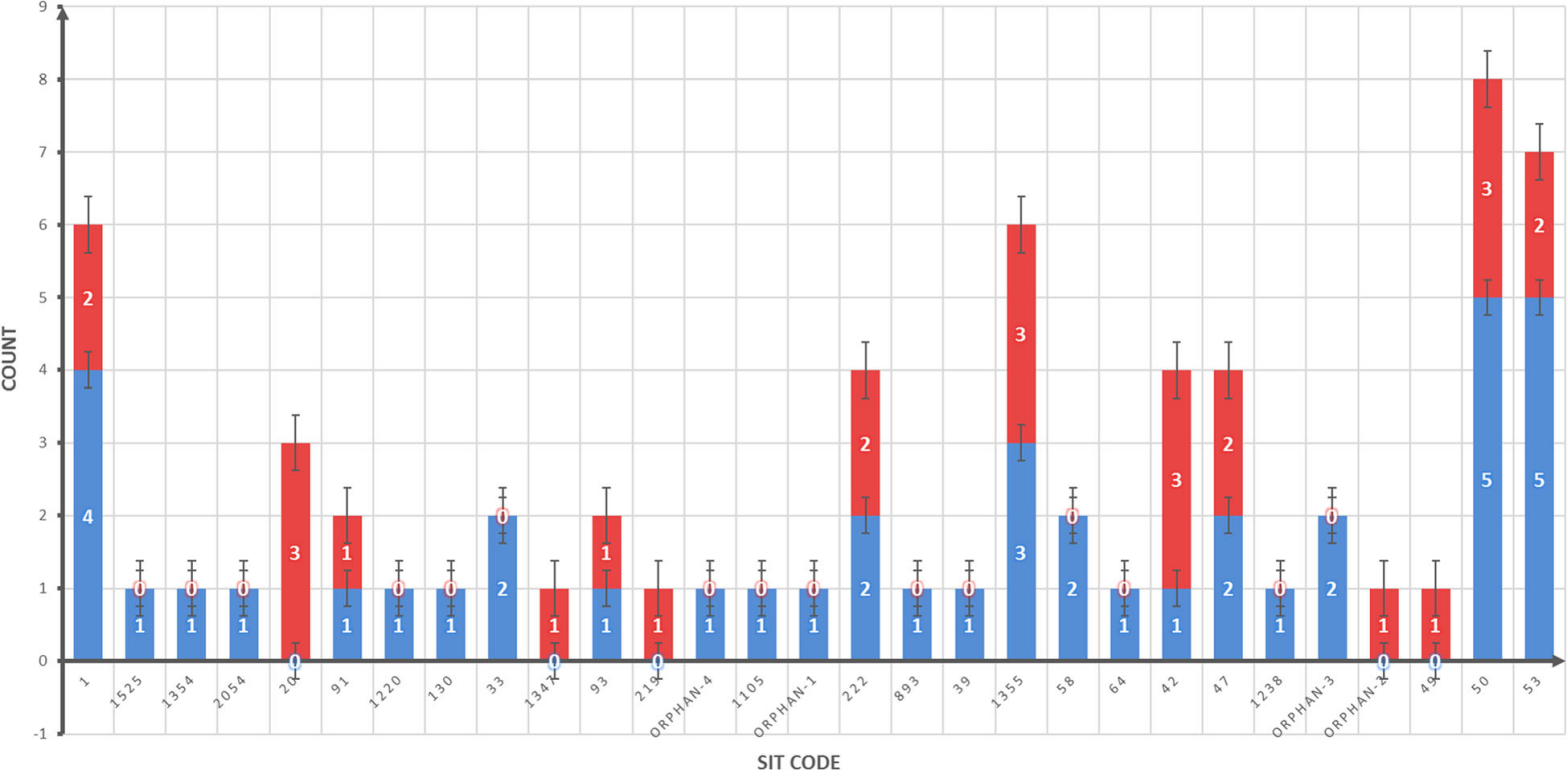
Histogram with standard errors of the strains in the study, according their SIT code. In *black* (PZA resistant strains), in *white* (PZA susceptible strains)

The phylogenetic analysis confirmed that the isolated strains are not related and do not cluster by PZA susceptibility (Fig. 1). In addition, the distribution of the clades and SIT stratified by PZA resistance, confirmed that PZA resistance is not exclusive and do not cluster into any specific clade (Additional file 1: Table S1 and Fig. 2 respectively). This suggests that potential bias due to the presence of particular genomic variants or transmission of particular clones, in the resistant strains is not likely happening in the selected sample.

### Identification of specific mutations in *pncA*/PZAse

Seventeen non-synonymous mutations in *pncA* were found only in resistant strains: D12A, T135P and D49N in two strains and D12G, Y34D, H51R, H57R, P62L, Y64D, G78C, F94 L, V131G, V139A and L182 W in only one strain. Only the mutations D12A, T135P and D49N were significantly associated with PZA-resistance (*P* = 0.034). Three non-synonymous mutations were found only in PZA-susceptible strains: mutation C14G in two strains and mutations K48 T and T160A in only one strain. No non-synonymous mutation in *pncA* appeared in both resistant and susceptible strains. Only one mutation in *pncA* promoter (T-11C) was found in two PZA-resistant strains (SLM60 and SLM100) without compromise of the coding region but was associated to a compromise in PZAse expression [12].

We identified seven PZA-resistant strains with no mutations in the pncA gene neither in its promoter (CSV4519, MDRDM627, TBV5000, TBV5362, TBV5365, SLM056 and SLM088). Five of them (colored in green in Table 2) also with no mutations in the specific set of genes selected. Detailed results are shown in Table 2.

**Table 2:** Mutations in *pncA* and the proteins of the special set selected and the best candidates from the global genome analysis by mutation that appeared to be statistically significant

		PZAse (Rv2043c)				Metal Ion transporters			Efflux pumps											Reported proteins		
**Isolate**	**Res PZA**	**Gene**	**Protein**	**Promoter**	**% of PZAse activity** ^b^	**NicT**	**Ctpl**	**CtpE**	**Rv0194**	**MppL11**	**MmpL3**	**MgtE**	**Rv0987**	**MmpL13a**	**MmpL10**	**Rv1348**	**Rv3239c**	**Mez**	**Rv2994**	**RpsA**	**GpsI**	**PstC2**
CSV4519	1	–	–	–	100.00	–	–	–	A968V	–	–	–	–	–	–	–	P548S	–	–	–	–	–
CSV4644	1	T152C	H51R	–	0.02	Y357C		–	–	–	–	E317Q	–	–		L830 V		–	–	A381V	–	–
CSV5769	1	C232A	G78C	–	18.12	–	–	–	–	–	–	–	–	–	–	–		–	–	–	–	–
CSV10399	1	A280G	F94 L	–	55.18	–	–	–	–	–	–	A77S	V83F	–	–	–		–	–	–	–	–
CSV11678	1	G185A	P62L	–	NA	–	–	–	–	–	–	–	–	–	–	–		–	–	–	–	–
LE486	1	T35G	D12A	–	24.06	–	–	–	–	–	–	–	–	–	–	–		–	–	–	–	–
LE492	1	T35G	D12A	–	24.06	–	–	–	–	–	–	–	–	–	–	–		–	–	–	–	–
LN180	1	A392C	V131G	–	NA	–	–	–	–	–	–	–	W797^a^	–	–	–		–	–	–	–	–
LN2358	1	A545C	L182 W	–	NA	–	–	–	–	–	–	–	–	–	–	–		–	–	–	–	–
LN3756	1	T403G	T135P	–	0.05	–	N355 K	–	–	–	–	–	–	–	L218 V	–		–	–	–	P96R	–
MDRDM260	1	T403G	T135P	–	0.05	–	N355 K	–	–	–	–	–	–	–	L218 V	–		–	–	–	P96R	–
**MDRDM627**	1	–	–	–	100.00	–	–	–	–	–	–	–	–	–	–	–		–	–	–	–	–
MDRDM1098	1	C145T	D49N	–	0.12	–	A1469P	–	A302T	–	E907A	–	–	–	–	–		–	–	M432 T	–	–
MDRMA2491	1	T35C	D12G	–	36.45	–		–	–	–	–	–		S265G		–		–	–	–	–	–
ME1473	1	A100C	Y34D	–	53.59	–	–	–	–	C263R	–	–	T142A	–	–	–		–	W214^a^	–		–
TBDM425	1	C145T	D49N	–	0.12	–	A1469P	–	A302T	–	E907A	–	–	–	–	–		–	–	M432 T	–	–
**TBV5000**	1	–	–	–	100.00	–		–	–	–	–	–	–	–	–	–		–	–	–	–	–
**TBV5362**	1	–	–	–	100.00	–		–	–	–	–	–	–	–	–	–		–	–	–	–	–
**TBV5365**	1	–	–	–	100.00	–	–	–	–	–	–	–	–	–	–	–	–	–	–	–	–	–
SLM036	1	A190C	Y64D	–	NA	–	–	–	–	–	–	–	–	–	–	–	–	–	–	–	–	–
SLM040	1	T170C	H57R	–	NA	–	–	–	–	–	–	–	–	–	–	–	–	D46E	–	–	–	W162G
**SLM056**	1	–	–	–	100.00	–	–	–	–	–	–	–	–	–	–	–	–	–	–	–	–	–
SLM060	1	–	–	T-11C	^c^	–	–	–	–	–	–	–		–		–	–	–	–	–	–	–
SLM063	1	A416G	V139A	–	NA	–	–	A653E	–	–	–	–	W797^a^	–	–	–	–	–	–	–	–	–
SLM088	1	–	–	–	NA	–	–	–	–	–	–	–	–	–	–	–	–	–	–	–	–	–
SLM100	1	–	–	T-11C	^c^	–	M920I	–	–	–	–	–	–	–	–	–	V904I	–	–	–	–	–

We are only showing the mutations that were only present in PZA-resistant strains. Strains harboring mutations strongly related with PZA resistance and strains with no mutations in PZA, neither in the specific set of genes selected, are in bold

^a^Aminoacid deletions

^b^Wild-PZAse (H37Rv) activity = 100%. NA: Not available

^c^Affects the expression of the Pyrazinamidase, but not its enzimatic activity

### Association of mutations with PZA-resistance

In the 68 genomes we found 53,515 DNA mutations in 2763 genes (20,763 synonymous and 32,752 non-synonymous mutations). When considering only non-synonymous mutations, there were 4118 different mutations in 2242 genes. From these, 152 mutations were noninformative as they were present in all the strains analyzed.

From the 4118 non-synonymous mutations, 715 (comprised in 582 genes) appeared only in resistant strains. The non-synonymous mutations that appeared in two or more resistant strains (202 in total, comprised in 187 genes) resulted significantly associated with PZA resistance (*P* < 0.05).

Those 202 mutations have the following frequency: 1 mutation (S1298 N in the protein *PPE34*) appeared in 4 strains; 8 mutations appeared in 3 strains and 193 mutations appeared in 2 strains. The complete results of the association analysis between resistance and mutations expressed in odds ratios are shown in Additional file 2: Table S2.

### Association of genes with PZA resistance

The analysis by genes showed that *pncA* was the gene with the highest association with PZA-resistance (*P* < 0.0001, proportion test, OR = 17.94). Mutations only present in resistant strains but not in susceptible strains were found in 214 genes. Five of these mutated genes *(rpsA*, *nrdR*, *FadD22*, *Rv1556* and *Rv3242c*) appeared in 3 strains and 63 in 2 strains. These genes were significantly associated with PZA resistance. The complete results of the association analysis between resistance and genes expressed in odds ratios are shown in Additional file 3: Table S3.

Additionally, intersecting the two-association analysis described above, we found 110 genes significantly associated with PZA resistance at gene-level, that also harbored mutations significantly associated with PZA resistance at mutation-level. The association analysis at mutation level was reproduced for this set, excluding the strains harboring mutations strongly related with PZA resistance (mostly mutations in pncA, colored in orange, in Table 2), calculating new association *P*-values. In order to understand the biological relevance, these genes were manually annotated to identify or predict their biological function (Additional file 4: Table S4). After excluding genes linked to resistance to other drugs and *pncA*, we found a set of genes associated with PZA resistance. Two of them (*rpsA* and *gpsL*) were recently reported [7]. Most of them remain incompletely studied and represent potential candidate genes to account for alternate mechanisms of PZA resistance. This group of genes was further analyzed. By manual annotation, cellular location and biological function were determined. These genes coded for soluble and trans membrane proteins, including functions of pH regulation, metallochaperones, and immune system evasion (Additional file 4: Table S4). The genes that remained significantly associated with PZA resistance during the re-analysis after excluding the strains with critical mutations in *pncA*, were *Rv2505c, Rv2777c, Rv0735, Rv0787A, Rv0994, Rv1327c, Rv1742, Rv2317, Rv2557, Rv2857c, Rv3362c, Rv3393, Rv3410c, Rv3767c, Rv2646, Rv0668*, and *Rv0667*.

### Analysis of mutations in the special set of genes

For each of the mutations identified as significantly associated with PZA resistance in genes of the special set of interest, we verified if the strains harboring them also showed mutations in *pncA* (colored in orange, Table 2). Mutations found only in resistant strains, even they were not statistically associated, were identified (Table 2).

#### Metallochaperones and metal ion transporters

Two non-synonymous mutations significantly associated with PZA resistance (*P* = 0.034) were found in the protein CtpI in four resistant strains only (mutation A1469P was found in strains MDRDM1098 and TBDM425, and mutation N355 K was found in strains MDRDM260 and LN3576) (Table 2). Nevertheless, these strains also showed known *pncA* mutations that impairs the PZAse activity [8] (strains MDRDM1098 and TBDM425 have the mutation D49N, and strains MDRDM260 and LN3576 have the mutation T135P in the PZAse).

The potential chaperon phoY1 showed the mutation 5YS statistically associated with PZA resistance, however this mutation was found in strains (MDRDM260 and LE3756), which have the mutation T135P in *pncA*. Remarkably, the CtpE chaperone showed the mutation A653E that occurs only in the PZA-resistant strain SLM063 that harbors the *pncA* mutation V139A. Mutation V139A has not been studied before, and its effect on the PZAse activity is still unknown (Table 2).

#### Efflux pumps

Four non-synonymous mutations significantly associated with PZA-resistance were found only in PZA-resistant strains. Three of them were found in the genes *MmpL10*, *MmpL3*, and *Rv0194* (A302T, in strains TBDM425 and MDRDM1098) (Table 2). However, these strains showed *pncA* mutations (T135P and D49N) associated with reduced PZAse activity [8].

The fourth mutation (the non-sense mutation W797*, in strains SLM063 and LN180) was found in the gene *Rv0987.* These strains showed the *pncA* mutations V139A and V131G, respectively, for which their effect on the PZAse activity is unknown.

#### *Other reported proteins associated to PZA-resistance (*RpsA*,* PstC2*,* Fas*,* PanD*)*

Mutations in *rpsA* were only found in resistant strains that harbored critical *pncA* mutations (D49N, T135P, and H51R), which are known to cause PZA resistance.

Mutations in *pstC2* were only found in a resistant strain (*P* = 0.100) that harbored the *pncA* mutation H57R associated with PZAse malfunction (Table 2).

Non-synonymous mutations were found in the *fas* gene in resistant strains without *pncA* mutations. However, no significant association with PZA resistance was found, probably due to the reduced sample size. We did not find any non-synonymous mutation in *panD*, in any of the strains analyzed in this study.

## Discussion

In this study we analyzed 68 *M. tuberculosis* complete genomes isolated from TB patients in Peru, and identified a set of novel mutations and genes significantly associated with PZA resistance in *M. tuberculosis*. Although some of these mutations are found only in PZA-resistant strains, some of these strains also harbored *pncA* critical mutations, confounding the contribution to PZA resistance. To evaluate the effect of this potential confounder, we conducted an analysis by including and excluding these strains.

It is known that a mutation in *pncA* does not necessarily cause a PZAse critical failure and PZA resistance. Previous studies have shown mutations that partially reduced PZAse activity resulting in a minor level of PZA resistance [8]. Therefore, some mutations in other genes, harbored in strains with *pncA* mutations, may eventually explain some level of PZA resistance in combination with reduction of PZAse activity.

We found *pncA* mutations V139A, L182 W and V131G, which have been previously reported in PZA-resistant strains, although no studies about their effect on PZAse activity are available yet. This leaves some PZA-resistant strains whose resistance eventually could not be explained by known genes and mechanisms.

Interestingly, this study found that several genes associated with PZA-resistance are also associated with resistance to other drugs including rifampin (*rpoB*), ethambutol (*embB*) and isoniazid (*katG*). This observation is likely explained by the high correlation between PZA resistance and multidrug resistance. In Peru, PZA is administered in both first and second line drugs treatment schemes, DOTS and DOTS Plus.

It is important to highlight that five PZA resistant strains analyzed (MDRDM627, TBV5000, TBV5362, TBV5365, and SLM056; Table 2) do not have mutations in *pncA* neither in the reported genes included in the special set of genes. After the *P*-value recalculation, we found 2 genes statistically associated with PZA resistance in two of these strains: *Rv2646* an integrase-like enzyme, and the RNA polymerase component *rpoC* for TBV5000 and TBV5365. It is likely that *rpoC* association could be simply a confounding due to association between MDR and PZA-resistance.

Notably, two strains showed no mutations in *pncA* but in the genes of the special set. These genes are *fadD35, Rv2777c, sigL, Rv0787A, moeA1, glgE, Rv1742, uspB, Rv2557, Rv2857c, Rv3362c, iunH, guaB3* and *Rv3767c*, and were present in the strains LN180 and SLM063 which share the same spoligotype.

The annotation of this group of genes, reveals different functions. Rv3659c is a putative membrane protein within a conserved ATP-binding transporter region that belongs to a Type IV pili – like operon and is homologous to VirB11 a gram negative protein from the type IV secretion system essential for pathogenicity [20]. Silencing Rv3659c increases macrophage apoptosis, while Rv3659c activation reduces macrophage apoptosis due to secretion of Rv3654c and Rv3655c, both required for suppression of macrophage apoptosis inactivation [20]. Nevertheless, as yet there is no experimental evidence that Rv3659c transports Rv3654c or Rv3655c. Rv3236c is an integral membrane protein that functions as a K^+^/H transporter that reduces phagosome ROS production by releasing K^+^ to the phagosomal space [21]. It has been proposed that K^+^ accumulation may be regulating NOX2 activity from phagocytes [21] based on similar events on neutrophils [22].

Rv2777c is a putative metal dependent hydrolase, related to bacilli persistence in macrophage and is up regulated during exposure to drugs that are active against non-replicating persistence such as capreomycin and PA-824 [23] and also in MDR strains [24]. Further experimental studies would be required in order to explore the association of these genes with PZA resistance.

The identification of genes related to the regulation of pH suggests an alternate mechanism of PZA resistance. An acidic extracellular environment within granulomata and macrophages in the latent state is necessary for PZA to be effective. Protons carried by the protonated pyrazinoic acid are likely to produce intracellular acidification that may be important in the mechanism of action of PZA, in particular favoring the interaction between POA and *RpsA* (Zimic, personal communication). Therefore, factors associated to stabilize intracellular pH are likely associated to PZA resistance.

Metal ions are necessary for *PZAse* activity and have a direct effect in the PZA antibiotic mechanism [5, 25, 26]. It has been speculated that the metalloenzyme PZAse of *M. tuberculosis* coordinates divalent metal ions with the participation of a metallochaperone [26]. We found genes associated to PZA resistance that are related with metal transporting and appeared mutated only in PZA-resistant strains. These proteins are members of Cation Transporter ATPase family. This family is specialized in ion transport (Mg^+2^, Zn^+2^, Co^+2^ and Ni^+2^) across membrane using ATP hydrolysis energy [26]. In particular, *CtpI* (*P* < 0.05) and *CtpE* (*P* < 0.10) (mutations in these genes appeared only in resistant strains) are involved in the cellular homeostasis of metal cations. Further studies would be required in order to explore its association with PZA resistance.

We identified mutations associated to PZA-resistance in four genes related to efflux pumps. These genes have been reported as ABC-transporters or mmpL proteins and drug-efflux pumps in *M. tuberculosis.* It is important to highlight that *Rv0194* showed a mutation (A968V) and Rv3239c showed a mutation (P548S), in a PZA-resistant strain with no mutation in *pncA*. Overexpression of *Rv0194* conferred multidrug resistance in *M. smegmatis* by increasing drug extrusion or lipid transport across the membrane [27]. It is important to further investigate if pyrazinoic acid (POA), could be a substrate of the Rv0194 ABC transporter. Notably, the mutation A77S in the *MgtE* efflux pump is only present in one resistant strain that harbors the *pncA* mutation F94 L, which reduces the PZAse activity partially [8]. Therefore, it is possible that the mutation A77S in MgtE may partially contribute with the resistance observed in this strain.

Shi et al. [7] discovered several proteins that bind to POA, including *RpsA*, *Rv2783c* a bifunctional protein (polyribonucleotide nucleotidyltransferase and ppGpp synthetase) involved in mRNA degradation, Rv2731 a conserved alanine and arginine rich domain protein, and Rv3169 a hypothetical protein. Feuerriegel [28] sequenced the *pncA* and *RpsA* genes of *M. canettii*, and found the mutations T5A, T210A, P9P and E457E in *RpsA*, which were suggested to be associated with PZA resistance [8]. It has been found that that the mutation (L122E) in *RpsA* is not associated with PZA resistance [29]. Our data found mutations in *rpsA*, however they were found in strains with *pncA* mutations that cause PZAse malfunction.

A recent study analyzing strains isolated from TB patients in China, found that mutations in *panD* are closely associated with PZA resistance [30]. This study showed that of 5 PZA-resistant isogenic mutants without *pncA* or *rpsA* mutations, all had *panD* mutations (A128S, V138A, H21R, I49V, and E130G). Our data, did not show mutations in *panD*.

A deep knowledge of the mutations associated with PZA resistance would contribute with PZA-resistance genome-sequencing testing efforts. A panel of SNPs could be established by considering geographic specificity, as it could be a differential lineage-related propensity for mutations at certain sites, or lineage-related propensity to allow compensation for otherwise lethal mutations. As an increasing number of strains are tested, more mutations in genes will be discovered. Although we cannot expect to have detected all of them in this small sample size, these results are evidence of the power of linking genomics with strong metadata, particularly phenotypic drug susceptibility. With the increasing speed and the reducing cost of bacterial genome sequencing, this could be an alternative to determine drug susceptibility using molecular tests.

A better knowledge of the genetic basis of PZA resistance will help clinicians to plan and deliver a more effective clinical management to TB cases, reducing the likelihood of drug resistance appearance. It also will aid the efforts for the development of new generation molecular-based point- of-care diagnostic assays with better sensitivity, specificity, and cost-effectiveness.

## Conclusions

In conclusion, this study identified new mutations and genes from *M. tuberculosis* statistically significant associated with PZA resistance and potentially linked biologically. Our evidence confirms that the patterns of mutations (SNPs and genes) associated with PZA resistance found in Peru do not match with those recently found in Asia, suggesting that the evolution of the mechanisms of PZA resistance may have evolved different in these two populations. The new mutations and genes found in this study, represent a source of potential alternate mechanisms of PZA resistance that need to be further studied and could significantly improve the sensitivity of molecular tests of PZA resistance.

## Additional files

Additional file 1: Table S1. Strains of the study, including their spoligotyping code (15 digits and 43 digits), PZA resistance, clade, SIT code and year of isolation. (DOCX 20 kb)

Additional file 2: Table S2. Complete results of the analysis by mutation, showing the number of resistant strains with the mutation (M-Res), without the mutation (NM-Res) and analogously for the susceptible strains (M-Sus and NM-Sus, respectively). The *P*-value of the Proportion Test, the Youden Index and Odds Ratio are also included. (DOCX 1264 kb)

Additional file 3: Table S3. Complete results of the analysis by gene, showing for each gene the number of resistant strains with at least one mutation (M-Res), without mutation (NM-Res) and analogously for the susceptible strains (M-Sus and NM-Sus, respectively). The P-value of the Proportion Test, the Youden Index and Odds Ratio are also included. (DOCX 610 kb)

Additional file 4: Table S4. Table of the 110 genes that were significantly associated with PZA-resistance at gene level, which also have mutations significantly associated with PZA-resistance at the mutation level. The association *p*-value at mutation level was re-calculated excluding the strains harboring mutations strongly related with PZA resistance. These genes were annotated with their molecular function. (DOCX 297 kb)

## Abbreviations

POA: Pyrazinoic acid
PZA: Pyrazinamide
PZAse: Pyrazinamidase
TB: Tuberculosis
