# Supplementary material for: A multiple genome analysis of Mycobacterium tuberculosis reveals specific novel genes and mutations associated with pyrazinamide resistance
Source: BMC Genomics. 2017 Oct 11;18:769. doi: 10.1186/s12864-017-4146-z (PMC5637355; doi:10.1186/s12864-017-4146-z)
Supplement: Supplementary file 1 — Strains of the study, including their spoligotyping code (15 digits and 43 digits), PZA resistance, clade, SIT code and year of isolation. (DOCX 20 kb) [file 12864_2017_4146_MOESM1_ESM.docx]

Additional file 1: Table S1. Strains of the study, including their spoligotyping code (15 digits and 43 digits), PZA resistance, clade, and SIT code.

| ID | SPOLIGO (15) | SPOLIGO (43) | PZA | CLADE | SIT |
| --- | --- | --- | --- | --- | --- |
| MDRMA2491 | 000000000003771 | 0000000000000000000000000000000000111111111 | R | Beijing | 1 |
| SLM060 | 000000000003771 | 0000000000000000000000000000000000111111111 | R | Beijing | 1 |
| CSV383 | 000000000003771 | 0000000000000000000000000000000000111111111 | S | Beijing | 1 |
| LN55 | 000000000003771 | 0000000000000000000000000000000000111111111 | S | Beijing | 1 |
| MDRMA1565 | 000000000003771 | 0000000000000000000000000000000000111111111 | S | Beijing | 1 |
| TBV4768 | 000000000003771 | 0000000000000000000000000000000000111111111 | S | Beijing | 1 |
| SLM088 | 777777774020771 | 1111111111111111111111111000000100001111111 | R | H1 | 47 |
| SLM100 | 777777774020771 | 1111111111111111111111111000000100001111111 | R | H1 | 47 |
| LE79 | 777777774020771 | 1111111111111111111111111000000100001111111 | S | H1 | 47 |
| MDRMA2082 | 777777774020771 | 1111111111111111111111111000000100001111111 | S | H1 | 47 |
| LN180 | 777777777720771 | 1111111111111111111111111111110100001111111 | R | H3 | 50 |
| SLM036 | 777777777720771 | 1111111111111111111111111111110100001111111 | R | H3 | 50 |
| SLM040 | 777777777720731 | 1111111111111111111111111111110100001110111 | R | H3 | 49 |
| SLM063 | 777777777720771 | 1111111111111111111111111111110100001111111 | R | H3 | 50 |
| LE13 | 777777777720771 | 1111111111111111111111111111110100001111111 | S | H3 | 50 |
| LN3589 | 777777777720771 | 1111111111111111111111111111110100001111111 | S | H3 | 50 |
| LN3695 | 777777777720771 | 1111111111111111111111111111110100001111111 | S | H3 | 50 |
| LN1100 | 777777777720771 | 1111111111111111111111111111110100001111111 | S | H3 | 50 |
| LN1856 | 777774077020771 | 1111111111111111000001111110000100001111111 | S | H3 | Orphan-1 |
| MDRMA863 | 777777777720771 | 1111111111111111111111111111110100001111111 | S | H3 | 50 |
| TBV4952 | 777777776720771 | 1111111111111111111111111101110100001111111 | S | H3 | 1238 |
| LE486 | 677777607760771 | 1101111111111111111100001111111100001111111 | R | LAM1 | 20 |
| LE492 | 677777607760771 | 1101111111111111111100001111111100001111111 | R | LAM1 | 20 |
| ME1473 | 677777607760771 | 1101111111111111111100001111111100001111111 | R | LAM1 | 20 |
| MDRMA2019 | 677777607760371 | 1101111111111111111100001111111100000111111 | S | LAM1 | 2054 |
| CSV9577 | 776177607760771 | 1111111100011111111100001111111100001111111 | S | LAM3 | 33 |
| LE103 | 776177607760731 | 1111111100011111111100001111111100001110111 | S | LAM3 | 130 |
| LE371 | 376137607760771 | 0111111100010111111100001111111100001111111 | S | LAM3 | 1525 |
| LN3584 | 776177607760771 | 1111111100011111111100001111111100001111111 | S | LAM3 | 33 |
| LN3672 | 576177607760771 | 1011111100011111111100001111111100001111111 | S | LAM3 | 1354 |
| TBDM2444 | 754377607760700 | 1111011000111111111100001111111100001110000 | S | LAM4 | 1220 |
| CSV4519 | 777737607760771 | 1111111111110111111100001111111100001111111 | R | LAM5 | 93 |
| MDRMA701 | 777737607760771 | 1111111111110111111100001111111100001111111 | S | LAM5 | 93 |
| CSV11678 | 777777407560731 | 1111111111111111111000001111011100001110111 | R | LAM6 | 1355 |
| TBV5000 | 777777407560731 | 1111111111111111111000001111011100001110111 | R | LAM6 | 1355 |
| TBV5362 | 777777407560731 | 1111111111111111111000001111011100001110111 | R | LAM6 | 1355 |
| LN763 | 777777407560731 | 1111111111111111111000001111011100001110111 | S | LAM6 | 1355 |
| MDRDM827 | 777777407560731 | 1111111111111111111000001111011100001110111 | S | LAM6 | 1355 |
| MDRMA2260 | 777777607560771 | 1111111111111111111100001111011100001111111 | S | LAM6 | 64 |
| TBDM2489 | 777777407560731 | 1111111111111111111000001111011100001110111 | S | LAM6 | 1355 |
| LN2358 | 777777607760771 | 1111111111111111111100001111111100001111111 | R | LAM9 | 42 |
| MDRDM627 | 777777607760771 | 1111111111111111111100001111111100001111111 | R | LAM9 | 42 |
| MDRDM1098 | 777777607760771 | 1111111111111111111100001111111100001111111 | R | LAM9 | 42 |
| LE76 | 777777607760771 | 1111111111111111111100001111111100001111111 | S | LAM9 | 42 |
| TBV5365 | 777777777720131 | 1111111111111111111111111111110100000010111 | R | Orphan | Orphan-2 |
| TBDM2699 | 777777777120771 | 1111111111111111111111111110010100001111111 | S | Orphan | Orphan-3 |
| TBDM2717 | 777777777120771 | 1111111111111111111111111110010100001111111 | S | Orphan | Orphan-3 |
| TBV4766 | 777760003560771 | 1111111111111100000000000111011100001111111 | S | Orphan | Orphan-4 |
| CSV4644 | 777774077560771 | 1111111111111111000001111111011100001111111 | R | T1 | 222 |
| CSV5769 | 777700777760771 | 1111111111110000001111111111111100001111111 | R | T1 | 1347 |
| CSV10399 | 777740777760771 | 1111111111111000001111111111111100001111111 | R | T1 | 219 |
| LN3756 | 777777777760771 | 1111111111111111111111111111111100001111111 | R | T1 | 53 |
| MDRDM260 | 777777777760771 | 1111111111111111111111111111111100001111111 | R | T1 | 53 |
| SLM056 | 777774077560771 | 1111111111111111000001111111011100001111111 | R | T1 | 222 |
| LE63 | 777777777760771 | 1111111111111111111111111111111100001111111 | S | T1 | 53 |
| LE410 | 777777777760771 | 1111111111111111111111111111111100001111111 | S | T1 | 53 |
| LN317 | 777777777760771 | 1111111111111111111111111111111100001111111 | S | T1 | 53 |
| LN2978 | 777777017760771 | 1111111111111111110000011111111100001111111 | S | T1 | 893 |
| LN3588 | 777774077560771 | 1111111111111111000001111111011100001111111 | S | T1 | 222 |
| LN3668 | 777777777760771 | 1111111111111111111111111111111100001111111 | S | T1 | 53 |
| LN2900 | 777774077560771 | 1111111111111111000001111111011100001111111 | S | T1 | 222 |
| TBDM2189 | 777777777760771 | 1111111111111111111111111111111100001111111 | S | T1 | 53 |
| TBDM2487 | 777773777760771 | 1111111111111110111111111111111100001111111 | S | T1 | 1105 |
| MDRMA203 | 777777347760471 | 1111111111111111110111001111111100001001111 | S | T4-CEU1 | 39 |
| CSV3611 | 777777557760771 | 1111111111111111111011011111111100001111111 | S | T5-Madrid2 | 58 |
| MDRMA2441 | 777777557760771 | 1111111111111111111011011111111100001111111 | S | T5-Madrid2 | 58 |
| TBDM425 | 700036777760771 | 1110000000000111101111111111111100001111111 | R | X3 | 91 |
| TBDM1506 | 700036777760771 | 1110000000000111101111111111111100001111111 | S | X3 | 91 |
