# Supplementary material for: A multiple genome analysis of Mycobacterium tuberculosis reveals specific novel genes and mutations associated with pyrazinamide resistance
Source: BMC Genomics. 2017 Oct 11;18:769. doi: 10.1186/s12864-017-4146-z (PMC5637355; doi:10.1186/s12864-017-4146-z)
Supplement: Supplementary file 2 — Complete results of the analysis by mutation, showing the number of resistant strains with the mutation (M-Res), without the mutation (NM-Res) and analogously for the susceptible strains (M-Sus and NM-Sus, respectively). The P-value of the Proportion Test, the Youden Index and Odds Ratio are also included. (DOCX 1264 kb) [file 12864_2017_4146_MOESM2_ESM.docx]

Additional file 2: Table S2. Complete results of the analysis by mutation, showing the number of resistant strains with the mutation (M-Res), without the mutation (NM-Res) and analogously for the susceptible strains (M-Sus and NM-Sus, respectively). The P-value of the Proportion Test, the Youden Index and Odds Ratio are also included.

| **Synonym** | **Gen Name** | **Mutation**  **(aa)** | **M-Res** | **NM- Res** | **M-Sus** | **NM-Sus** | **pvalue Test prop** | **Youden J** | **O.R.** |
| --- | --- | --- | --- | --- | --- | --- | --- | --- | --- |
| Rv0667  Rv1908c  Rv2853  Rv1917c  Rv1313c  Rv3508  Rv3508  Rv1917c  Rv0280  Rv0278c  Rv2487c  Rv2543  Rv2718c  Rv2935  Rv3347c Rv3659c Rv3795  Rv3888c | rpoB katG PE_PGRS48  PPE34  Rv1313c  PE_PGRS54  PE_PGRS54  PPE34  PPE3  PE_PGRS3  PE_PGRS42 lppA nrdR ppsE  PPE55  Rv3659c embB Rv3888c | 450S>L  315S>T  180R>G  1298S>N  433L>R  1180S>A  979S>A  1290N>D  337S>P  807R>G  313G>R  151A>V  95A>V  1463T>P  1568Y>H  337D>A  354D>A  310V>L | 19  22  23  4  19  11  11  5  22  18  3  3  3  3  3  3  3  3 | 7  4  3  22  7  15  15  21  4  8  23  23  23  23  23  23  23  23 | 5  13  24  0  17  6  6  1  24  17  0  0  0  0  0  0  0  0 | 37  29  18  42  25  36  36  41  18  25  42  42  42  42  42  42  42  42 | 0,0000  0,0000  0,0033  0,0044  0,0044  0,0048  0,0048  0,0086  0,0093  0,0106  0,0122  0,0122  0,0122  0,0122  0,0122  0,0122  0,0122  0,0122 | 0,6117  0,5366  0,3132  0,1538  0,3260  0,2802  0,2802  0,1685  0,2747  0,2875  0,1154  0,1154  0,1154  0,1154  0,1154  0,1154  0,1154  0,1154 | 20,0857  12,2692  5,7500 non-defined  3,9916  4,4000  4,4000  9,7619  4,1250  3,3088 non-defined  non-defined non-defined non-defined non-defined non-defined non-defined  non-defined |

| Rv1396c | PE_PGRS25 | 66R>S | 19 | 7 | 19 | 23 | 0,0123 | 0,2784 | 3,2857 |
| --- | --- | --- | --- | --- | --- | --- | --- | --- | --- |
| Rv0532 | PE_PGRS6 | 227D>G | 23 | 3 | 27 | 15 | 0,0140 | 0,2418 | 4,2593 |
| Rv0532 | PE_PGRS6 | 239A>G | 23 | 3 | 27 | 15 | 0,0140 | 0,2418 | 4,2593 |
| Rv1753c | PPE24 | 488N>T | 17 | 9 | 16 | 26 | 0,0143 | 0,2729 | 3,0694 |
| Rv3511 | PE_PGRS55 | 396N>D | 21 | 5 | 23 | 19 | 0,0146 | 0,2601 | 3,4696 |
| Rv0958 | Rv0958 | 274S>P | 26 | 0 | 36 | 6 | 0,0218 | 0,1429 | non-defined |
| Rv1872c | lldD2 | 3V>I | 4 | 22 | 1 | 41 | 0,0229 | 0,1300 | 7,4545 |
| Rv3508 | PE_PGRS54 | 1232S>N | 7 | 19 | 4 | 38 | 0,0291 | 0,1740 | 3,5000 |
| Rv0006 | gyrA | 668G>D | 26 | 0 | 37 | 5 | 0,0338 | 0,1190 | non-defined |
| Rv0006 | gyrA | 95S>T | 26 | 0 | 37 | 5 | 0,0338 | 0,1190 | non-defined |
| Rv0218 | Rv0218 | 316C>R | 26 | 0 | 37 | 5 | 0,0338 | 0,1190 | non-defined |
| Rv0338c | Rv0338c | 506R>G | 26 | 0 | 37 | 5 | 0,0338 | 0,1190 | non-defined |
| Rv0545c | pitA | 49P>S | 26 | 0 | 37 | 5 | 0,0338 | 0,1190 | non-defined |
| Rv0785 | Rv0785 | 408C>F | 26 | 0 | 37 | 5 | 0,0338 | 0,1190 | non-defined |
| Rv0848 | cysK2 | 93G>S | 26 | 0 | 37 | 5 | 0,0338 | 0,1190 | non-defined |
| Rv0930 | pstA1 | 5M>T | 26 | 0 | 37 | 5 | 0,0338 | 0,1190 | non-defined |
| Rv0938 | Rv0938 | 344C>R | 26 | 0 | 37 | 5 | 0,0338 | 0,1190 | non-defined |
| Rv0974c | accD2 | 51N>K | 26 | 0 | 37 | 5 | 0,0338 | 0,1190 | non-defined |
| Rv0989c | grcC2 | 321I>V | 26 | 0 | 37 | 5 | 0,0338 | 0,1190 | non-defined |
| Rv1394c | cyp132 | 135R>L | 26 | 0 | 37 | 5 | 0,0338 | 0,1190 | non-defined |

| Rv1449c | tkt | 18Y>D | 26 | 0 | 37 | 5 | 0,0338 | 0,1190 | non-defined |
| --- | --- | --- | --- | --- | --- | --- | --- | --- | --- |
| Rv1604 | impA | 124P>Q | 26 | 0 | 37 | 5 | 0,0338 | 0,1190 | non-defined |
| Rv1644 | tsnR | 232L>P | 26 | 0 | 37 | 5 | 0,0338 | 0,1190 | non-defined |
| Rv1807 | PPE31 | 234V>L | 26 | 0 | 37 | 5 | 0,0338 | 0,1190 | non-defined |
| Rv1900c | lipJ | 204I>M | 26 | 0 | 37 | 5 | 0,0338 | 0,1190 | non-defined |
| Rv2072c | cobL | 205L>P | 26 | 0 | 37 | 5 | 0,0338 | 0,1190 | non-defined |
| Rv2236c | cobD | 79S>C | 26 | 0 | 37 | 5 | 0,0338 | 0,1190 | non-defined |
| Rv2379c | mbtF | 589E>D | 26 | 0 | 37 | 5 | 0,0338 | 0,1190 | non-defined |
| Rv2398c | cysW | 141G>A | 26 | 0 | 37 | 5 | 0,0338 | 0,1190 | non-defined |
| Rv2691 | ceoB | 117T>A | 26 | 0 | 37 | 5 | 0,0338 | 0,1190 | non-defined |
| Rv2729c | Rv2729c | 202A>E | 26 | 0 | 37 | 5 | 0,0338 | 0,1190 | non-defined |
| Rv2825c | Rv2825c | 2K>E | 26 | 0 | 37 | 5 | 0,0338 | 0,1190 | non-defined |
| Rv2981c | ddl | 365T>A | 26 | 0 | 37 | 5 | 0,0338 | 0,1190 | non-defined |
| Rv3042c | serB2 | 116G>E | 26 | 0 | 37 | 5 | 0,0338 | 0,1190 | non-defined |
| Rv3151 | nuoG | 474I>M | 26 | 0 | 37 | 5 | 0,0338 | 0,1190 | non-defined |
| Rv3383c | idsB | 132V>G | 26 | 0 | 37 | 5 | 0,0338 | 0,1190 | non-defined |

| Rv3447c | Rv3447c | 1082S>G | 26 | 0 | 37 | 5 | 0,0338 | 0,1190 | non-defined |
| --- | --- | --- | --- | --- | --- | --- | --- | --- | --- |
| Rv3468c | Rv3468c | 62I>V | 26 | 0 | 37 | 5 | 0,0338 | 0,1190 | non-defined |
| Rv3521 | Rv3521 | 295N>D | 26 | 0 | 37 | 5 | 0,0338 | 0,1190 | non-defined |
| Rv3630 | Rv3630 | 40A>T | 26 | 0 | 37 | 5 | 0,0338 | 0,1190 | non-defined |
| Rv3731 | ligC | 313R>H | 26 | 0 | 37 | 5 | 0,0338 | 0,1190 | non-defined |
| Rv0002 | dnaN | 307R>L | 2 | 24 | 0 | 42 | 0,0340 | 0,0769 | non-defined |
| Rv0003 | recF | 54V>I | 2 | 24 | 0 | 42 | 0,0340 | 0,0769 | non-defined |
| Rv0006 | gyrA | 90A>V | 2 | 24 | 0 | 42 | 0,0340 | 0,0769 | non-defined |
| Rv0045c | Rv0045c | 45W>* | 2 | 24 | 0 | 42 | 0,0340 | 0,0769 | non-defined |
| Rv0062 | celA1 | 250W>S | 2 | 24 | 0 | 42 | 0,0340 | 0,0769 | non-defined |
| Rv0095c | Rv0095c | 80R>C | 2 | 24 | 0 | 42 | 0,0340 | 0,0769 | non-defined |
| Rv0107c | ctpI | 1469A>P | 2 | 24 | 0 | 42 | 0,0340 | 0,0769 | non-defined |
| Rv0107c | ctpI | 355N>K | 2 | 24 | 0 | 42 | 0,0340 | 0,0769 | non-defined |
| Rv0161 | Rv0161 | 87E>K | 2 | 24 | 0 | 42 | 0,0340 | 0,0769 | non-defined |
| Rv0166 | fadD5 | 233P>A | 2 | 24 | 0 | 42 | 0,0340 | 0,0769 | non-defined |
| Rv0168 | yrbE1B | 288T>A | 2 | 24 | 0 | 42 | 0,0340 | 0,0769 | non-defined |

| Rv0194 | Rv0194 | 302A>T | 2 | 24 | 0 | 42 | 0,0340 | 0,0769 | non-defined |
| --- | --- | --- | --- | --- | --- | --- | --- | --- | --- |
| Rv0206c | mmpL3 | 907E>A | 2 | 24 | 0 | 42 | 0,0340 | 0,0769 | non-defined |
| Rv0209 | Rv0209 | 313H>R | 2 | 24 | 0 | 42 | 0,0340 | 0,0769 | non-defined |
| Rv0231 | fadE4 | 148M>T | 2 | 24 | 0 | 42 | 0,0340 | 0,0769 | non-defined |
| Rv0233 | nrdB | 295A>E | 2 | 24 | 0 | 42 | 0,0340 | 0,0769 | non-defined |
| Rv0273c | Rv0273c | 167C>Y | 2 | 24 | 0 | 42 | 0,0340 | 0,0769 | non-defined |
| Rv0275c | Rv0275c | 110A>V | 2 | 24 | 0 | 42 | 0,0340 | 0,0769 | non-defined |
| Rv0304c | PPE5 | 1845F>S | 2 | 24 | 0 | 42 | 0,0340 | 0,0769 | non-defined |
| Rv0329c | Rv0329c | 142A>T | 2 | 24 | 0 | 42 | 0,0340 | 0,0769 | non-defined |
| Rv0355c | PPE8 | 2749H>N | 2 | 24 | 0 | 42 | 0,0340 | 0,0769 | non-defined |
| Rv0381c | Rv0381c | 251A>T | 2 | 24 | 0 | 42 | 0,0340 | 0,0769 | non-defined |
| Rv0389 | purT | 378E>* | 2 | 24 | 0 | 42 | 0,0340 | 0,0769 | non-defined |
| Rv0405 | pks6 | 769E>D | 2 | 24 | 0 | 42 | 0,0340 | 0,0769 | non-defined |
| Rv0508 | Rv0508 | 7E>K | 2 | 24 | 0 | 42 | 0,0340 | 0,0769 | non-defined |
| Rv0548c | menB | 122T>N | 2 | 24 | 0 | 42 | 0,0340 | 0,0769 | non-defined |
| Rv0566c | Rv0566c | 104S>R | 2 | 24 | 0 | 42 | 0,0340 | 0,0769 | non-defined |

| Rv0578c | PE_PGRS7 | 837T>N | 2 | 24 | 0 | 42 | 0,0340 | 0,0769 | non-defined |
| --- | --- | --- | --- | --- | --- | --- | --- | --- | --- |
| Rv0589 | mce2A | 32V>G | 2 | 24 | 0 | 42 | 0,0340 | 0,0769 | non-defined |
| Rv0630c | recB | 974I>V | 2 | 24 | 0 | 42 | 0,0340 | 0,0769 | non-defined |
| Rv0631c | recC | 976I>V | 2 | 24 | 0 | 42 | 0,0340 | 0,0769 | non-defined |
| Rv0644c | mmaA2 | 244Q>R | 2 | 24 | 0 | 42 | 0,0340 | 0,0769 | non-defined |
| Rv0667 | rpoB | 692A>T | 2 | 24 | 0 | 42 | 0,0340 | 0,0769 | non-defined |
| Rv0668 | rpoC | 1252V>M | 2 | 24 | 0 | 42 | 0,0340 | 0,0769 | non-defined |
| Rv0668 | rpoC | 812T>I | 2 | 24 | 0 | 42 | 0,0340 | 0,0769 | non-defined |
| Rv0682 | rpsL | 88K>T | 2 | 24 | 0 | 42 | 0,0340 | 0,0769 | non-defined |
| Rv0697 | Rv0697 | 155T>A | 2 | 24 | 0 | 42 | 0,0340 | 0,0769 | non-defined |
| Rv0735 | sigL | 160H>Y | 2 | 24 | 0 | 42 | 0,0340 | 0,0769 | non-defined |
| Rv0747 | PE_PGRS10 | 502G>D | 2 | 24 | 0 | 42 | 0,0340 | 0,0769 | non-defined |
| Rv0787A | Rv0787A | 71D>N | 2 | 24 | 0 | 42 | 0,0340 | 0,0769 | non-defined |
| Rv0800 | pepC | 118D>A | 2 | 24 | 0 | 42 | 0,0340 | 0,0769 | non-defined |
| Rv0853c | pdc | 247P>T | 2 | 24 | 0 | 42 | 0,0340 | 0,0769 | non-defined |
| Rv0894 | Rv0894 | 383V>I | 2 | 24 | 0 | 42 | 0,0340 | 0,0769 | non-defined |

| Rv0921 | Rv0921 | 139G>R | 2 | 24 | 0 | 42 | 0,0340 | 0,0769 | non-defined |
| --- | --- | --- | --- | --- | --- | --- | --- | --- | --- |
| Rv0937c | Rv0937c | 233F>S | 2 | 24 | 0 | 42 | 0,0340 | 0,0769 | non-defined |
| Rv0987 | Rv0987 | 797W>* | 2 | 24 | 0 | 42 | 0,0340 | 0,0769 | non-defined |
| Rv0994 | moeA1 | 394T>A | 2 | 24 | 0 | 42 | 0,0340 | 0,0769 | non-defined |
| Rv1024 | Rv1024 | 158P>T | 2 | 24 | 0 | 42 | 0,0340 | 0,0769 | non-defined |
| Rv1027c | kdpE | 169G>S | 2 | 24 | 0 | 42 | 0,0340 | 0,0769 | non-defined |
| Rv1034c | Rv1034c | 49G>A | 2 | 24 | 0 | 42 | 0,0340 | 0,0769 | non-defined |
| Rv1046c | Rv1046c | 26A>V | 2 | 24 | 0 | 42 | 0,0340 | 0,0769 | non-defined |
| Rv1052 | Rv1052 | 14G>S | 2 | 24 | 0 | 42 | 0,0340 | 0,0769 | non-defined |
| Rv1060 | Rv1060 | 52A>V | 2 | 24 | 0 | 42 | 0,0340 | 0,0769 | non-defined |
| Rv1079 | metB | 38A>V | 2 | 24 | 0 | 42 | 0,0340 | 0,0769 | non-defined |
| Rv1087 | PE_PGRS21 | 424G>S | 2 | 24 | 0 | 42 | 0,0340 | 0,0769 | non-defined |
| Rv1087 | PE_PGRS21 | 705W>S | 2 | 24 | 0 | 42 | 0,0340 | 0,0769 | non-defined |
| Rv1129c | Rv1129c | 323T>A | 2 | 24 | 0 | 42 | 0,0340 | 0,0769 | non-defined |
| Rv1130 | Rv1130 | 162A>P | 2 | 24 | 0 | 42 | 0,0340 | 0,0769 | non-defined |
| Rv1138c | Rv1138c | 48G>R | 2 | 24 | 0 | 42 | 0,0340 | 0,0769 | non-defined |

| Rv1161 | narG | 1004V>M | 2 | 24 | 0 | 42 | 0,0340 | 0,0769 | non-defined |
| --- | --- | --- | --- | --- | --- | --- | --- | --- | --- |
| Rv1161 | narG | 253D>N | 2 | 24 | 0 | 42 | 0,0340 | 0,0769 | non-defined |
| Rv1183 | mmpL10 | 218L>V | 2 | 24 | 0 | 42 | 0,0340 | 0,0769 | non-defined |
| Rv1187 | rocA | 61V>M | 2 | 24 | 0 | 42 | 0,0340 | 0,0769 | non-defined |
| Rv1188 | Rv1188 | 244I>V | 2 | 24 | 0 | 42 | 0,0340 | 0,0769 | non-defined |
| Rv1251c | Rv1251c | 629G>R | 2 | 24 | 0 | 42 | 0,0340 | 0,0769 | non-defined |
| Rv1292 | argS | 91M>I | 2 | 24 | 0 | 42 | 0,0340 | 0,0769 | non-defined |
| Rv1320c | Rv1320c | 76A>V | 2 | 24 | 0 | 42 | 0,0340 | 0,0769 | non-defined |
| Rv1326c | glgB | 539G>R | 2 | 24 | 0 | 42 | 0,0340 | 0,0769 | non-defined |
| Rv1327c | glgE | 8T>A | 2 | 24 | 0 | 42 | 0,0340 | 0,0769 | non-defined |
| Rv1334 | Rv1334 | 42G>A | 2 | 24 | 0 | 42 | 0,0340 | 0,0769 | non-defined |
| Rv1508c | Rv1508c | 192V>I | 2 | 24 | 0 | 42 | 0,0340 | 0,0769 | non-defined |
| Rv1539 | lspA | 2P>S | 2 | 24 | 0 | 42 | 0,0340 | 0,0769 | non-defined |
| Rv1541c | lprI | 143D>A | 2 | 24 | 0 | 42 | 0,0340 | 0,0769 | non-defined |
| Rv1544 | Rv1544 | 35P>R | 2 | 24 | 0 | 42 | 0,0340 | 0,0769 | non-defined |
| Rv1547 | dnaE | 1031A>S | 2 | 24 | 0 | 42 | 0,0340 | 0,0769 | non-defined |

| Rv1551 | plsB1 | 32P>A | 2 | 24 | 0 | 42 | 0,0340 | 0,0769 | non-defined |
| --- | --- | --- | --- | --- | --- | --- | --- | --- | --- |
| Rv1556 | Rv1556 | 164L>V | 2 | 24 | 0 | 42 | 0,0340 | 0,0769 | non-defined |
| Rv1612 | trpB | 276V>I | 2 | 24 | 0 | 42 | 0,0340 | 0,0769 | non-defined |
| Rv1618 | tesB1 | 24S>G | 2 | 24 | 0 | 42 | 0,0340 | 0,0769 | non-defined |
| Rv1621c | cydD | 13G>S | 2 | 24 | 0 | 42 | 0,0340 | 0,0769 | non-defined |
| Rv1630 | rpsA | 432M>T | 2 | 24 | 0 | 42 | 0,0340 | 0,0769 | non-defined |
| Rv1653 | argJ | 72L>R | 2 | 24 | 0 | 42 | 0,0340 | 0,0769 | non-defined |
| Rv1742 | Rv1742 | 185Y>C | 2 | 24 | 0 | 42 | 0,0340 | 0,0769 | non-defined |
| Rv1749c | Rv1749c | 136H>R | 2 | 24 | 0 | 42 | 0,0340 | 0,0769 | non-defined |
| Rv1752 | Rv1752 | 46G>E | 2 | 24 | 0 | 42 | 0,0340 | 0,0769 | non-defined |
| Rv1753c | PPE24 | 338N>D | 2 | 24 | 0 | 42 | 0,0340 | 0,0769 | non-defined |
| Rv1771 | Rv1771 | 116A>V | 2 | 24 | 0 | 42 | 0,0340 | 0,0769 | non-defined |
| Rv1781c | malQ | 172R>H | 2 | 24 | 0 | 42 | 0,0340 | 0,0769 | non-defined |
| Rv1812c | Rv1812c | 232G>S | 2 | 24 | 0 | 42 | 0,0340 | 0,0769 | non-defined |
| Rv1865c | Rv1865c | 208G>R | 2 | 24 | 0 | 42 | 0,0340 | 0,0769 | non-defined |
| Rv1868 | Rv1868 | 9T>P | 2 | 24 | 0 | 42 | 0,0340 | 0,0769 | non-defined |

| Rv1937 | Rv1937 | 552A>G | 2 | 24 | 0 | 42 | 0,0340 | 0,0769 | non-defined |
| --- | --- | --- | --- | --- | --- | --- | --- | --- | --- |
| Rv1939 | Rv1939 | 124A>G | 2 | 24 | 0 | 42 | 0,0340 | 0,0769 | non-defined |
| Rv1946c | lppG | 77A>T | 2 | 24 | 0 | 42 | 0,0340 | 0,0769 | non-defined |
| Rv1960c | Rv1960c | 30S>T | 2 | 24 | 0 | 42 | 0,0340 | 0,0769 | non-defined |
| Rv1968 | mce3C | 228R>H | 2 | 24 | 0 | 42 | 0,0340 | 0,0769 | non-defined |
| Rv1998c | Rv1998c | 183I>V | 2 | 24 | 0 | 42 | 0,0340 | 0,0769 | non-defined |
| Rv1999c | Rv1999c | 271N>D | 2 | 24 | 0 | 42 | 0,0340 | 0,0769 | non-defined |
| Rv2025c | Rv2025c | 127I>T | 2 | 24 | 0 | 42 | 0,0340 | 0,0769 | non-defined |
| Rv2025c | Rv2025c | 188D>N | 2 | 24 | 0 | 42 | 0,0340 | 0,0769 | non-defined |
| Rv2036 | Rv2036 | 209A>V | 2 | 24 | 0 | 42 | 0,0340 | 0,0769 | non-defined |
| Rv2043c | pncA | 12D>A | 2 | 24 | 0 | 42 | 0,0340 | 0,0769 | non-defined |
| Rv2043c | pncA | 135T>P | 2 | 24 | 0 | 42 | 0,0340 | 0,0769 | non-defined |
| Rv2043c | pncA | 49D>N | 2 | 24 | 0 | 42 | 0,0340 | 0,0769 | non-defined |
| Rv2052c | Rv2052c | 429A>T | 2 | 24 | 0 | 42 | 0,0340 | 0,0769 | non-defined |
| Rv2157c | murF | 70A>T | 2 | 24 | 0 | 42 | 0,0340 | 0,0769 | non-defined |
| Rv2205c | Rv2205c | 301Q>* | 2 | 24 | 0 | 42 | 0,0340 | 0,0769 | non-defined |

| Rv2210c | ilvE | 91V>M | 2 | 24 | 0 | 42 | 0,0340 | 0,0769 | non-defined |
| --- | --- | --- | --- | --- | --- | --- | --- | --- | --- |
| Rv2276 | cyp121 | 208R>H | 2 | 24 | 0 | 42 | 0,0340 | 0,0769 | non-defined |
| Rv2317 | uspB | 265V>F | 2 | 24 | 0 | 42 | 0,0340 | 0,0769 | non-defined |
| Rv2351c | plcA | 377G>A | 2 | 24 | 0 | 42 | 0,0340 | 0,0769 | non-defined |
| Rv2370c | Rv2370c | 293Q>E | 2 | 24 | 0 | 42 | 0,0340 | 0,0769 | non-defined |
| Rv2402 | Rv2402 | 582D>N | 2 | 24 | 0 | 42 | 0,0340 | 0,0769 | non-defined |
| Rv2465c | Rv2465c | 114M>I | 2 | 24 | 0 | 42 | 0,0340 | 0,0769 | non-defined |
| Rv2484c | Rv2484c | 308V>A | 2 | 24 | 0 | 42 | 0,0340 | 0,0769 | non-defined |
| Rv2487c | PE_PGRS42 | 427G>V | 2 | 24 | 0 | 42 | 0,0340 | 0,0769 | non-defined |
| Rv2505c | fadD35 | 202Y>H | 2 | 24 | 0 | 42 | 0,0340 | 0,0769 | non-defined |
| Rv2551c | Rv2551c | 58V>M | 2 | 24 | 0 | 42 | 0,0340 | 0,0769 | non-defined |
| Rv2557 | Rv2557 | 128W>* | 2 | 24 | 0 | 42 | 0,0340 | 0,0769 | non-defined |
| Rv2560 | Rv2560 | 134N>S | 2 | 24 | 0 | 42 | 0,0340 | 0,0769 | non-defined |
| Rv2646 | Rv2646 | 316Y>D | 2 | 24 | 0 | 42 | 0,0340 | 0,0769 | non-defined |
| Rv2657c | Rv2657c | 57R>G | 2 | 24 | 0 | 42 | 0,0340 | 0,0769 | non-defined |
| Rv2675c | Rv2675c | 40V>M | 2 | 24 | 0 | 42 | 0,0340 | 0,0769 | non-defined |

| Rv2764c | thyA | 207H>R | 2 | 24 | 0 | 42 | 0,0340 | 0,0769 | non-defined |
| --- | --- | --- | --- | --- | --- | --- | --- | --- | --- |
| Rv2777c | Rv2777c | 112P>S | 2 | 24 | 0 | 42 | 0,0340 | 0,0769 | non-defined |
| Rv2783c | gpsI | 96P>R | 2 | 24 | 0 | 42 | 0,0340 | 0,0769 | non-defined |
| Rv2802c | Rv2802c | 198R>W | 2 | 24 | 0 | 42 | 0,0340 | 0,0769 | non-defined |
| Rv2802c | Rv2802c | 249R>L | 2 | 24 | 0 | 42 | 0,0340 | 0,0769 | non-defined |
| Rv2823c | Rv2823c | 266T>I | 2 | 24 | 0 | 42 | 0,0340 | 0,0769 | non-defined |
| Rv2833c | ugpB | 214N>T | 2 | 24 | 0 | 42 | 0,0340 | 0,0769 | non-defined |
| Rv2857c | Rv2857c | 164V>G | 2 | 24 | 0 | 42 | 0,0340 | 0,0769 | non-defined |
| Rv2905 | lppW | 137V>G | 2 | 24 | 0 | 42 | 0,0340 | 0,0769 | non-defined |
| Rv2934 | ppsD | 1714A>T | 2 | 24 | 0 | 42 | 0,0340 | 0,0769 | non-defined |
| Rv2948c | fadD22 | 2R>W | 2 | 24 | 0 | 42 | 0,0340 | 0,0769 | non-defined |
| Rv3039c | echA17 | 244G>D | 2 | 24 | 0 | 42 | 0,0340 | 0,0769 | non-defined |
| Rv3049c | Rv3049c | 57V>I | 2 | 24 | 0 | 42 | 0,0340 | 0,0769 | non-defined |
| Rv3080c | pknK | 621G>R | 2 | 24 | 0 | 42 | 0,0340 | 0,0769 | non-defined |
| Rv3094c | Rv3094c | 325D>N | 2 | 24 | 0 | 42 | 0,0340 | 0,0769 | non-defined |
| Rv3097c | lipY | 49S>A | 2 | 24 | 0 | 42 | 0,0340 | 0,0769 | non-defined |

| Rv3120 | Rv3120 | 71C>R | 2 | 24 | 0 | 42 | 0,0340 | 0,0769 | non-defined |
| --- | --- | --- | --- | --- | --- | --- | --- | --- | --- |
| Rv3151 | nuoG | 769V>I | 2 | 24 | 0 | 42 | 0,0340 | 0,0769 | non-defined |
| Rv3158 | nuoN | 336V>I | 2 | 24 | 0 | 42 | 0,0340 | 0,0769 | non-defined |
| Rv3190c | Rv3190c | 394G>A | 2 | 24 | 0 | 42 | 0,0340 | 0,0769 | non-defined |
| Rv3223c | sigH | 16G>R | 2 | 24 | 0 | 42 | 0,0340 | 0,0769 | non-defined |
| Rv3227 | aroA | 267V>G | 2 | 24 | 0 | 42 | 0,0340 | 0,0769 | non-defined |
| Rv3236c | Rv3236c | 300V>I | 2 | 24 | 0 | 42 | 0,0340 | 0,0769 | non-defined |
| Rv3236c | Rv3236c | 91P>S | 2 | 24 | 0 | 42 | 0,0340 | 0,0769 | non-defined |
| Rv3242c | Rv3242c | 148R>W | 2 | 24 | 0 | 42 | 0,0340 | 0,0769 | non-defined |
| Rv3256c | Rv3256c | 106G>A | 2 | 24 | 0 | 42 | 0,0340 | 0,0769 | non-defined |
| Rv3279c | birA | 197R>Q | 2 | 24 | 0 | 42 | 0,0340 | 0,0769 | non-defined |
| Rv3297 | nei | 111I>F | 2 | 24 | 0 | 42 | 0,0340 | 0,0769 | non-defined |
| Rv3301c | phoY1 | 5Y>S | 2 | 24 | 0 | 42 | 0,0340 | 0,0769 | non-defined |
| Rv3317 | sdhD | 114R>Q | 2 | 24 | 0 | 42 | 0,0340 | 0,0769 | non-defined |
| Rv3329 | Rv3329 | 49V>F | 2 | 24 | 0 | 42 | 0,0340 | 0,0769 | non-defined |
| Rv3343c | PPE54 | 1322P>Q | 2 | 24 | 0 | 42 | 0,0340 | 0,0769 | non-defined |

| Rv3362c | Rv3362c | 80V>A | 2 | 24 | 0 | 42 | 0,0340 | 0,0769 | non-defined |
| --- | --- | --- | --- | --- | --- | --- | --- | --- | --- |
| Rv3391 | acrA1 | 615P>L | 2 | 24 | 0 | 42 | 0,0340 | 0,0769 | non-defined |
| Rv3393 | iunH | 31G>S | 2 | 24 | 0 | 42 | 0,0340 | 0,0769 | non-defined |
| Rv3410c | guaB3 | 36A>T | 2 | 24 | 0 | 42 | 0,0340 | 0,0769 | non-defined |
| Rv3468c | Rv3468c | 28T>A | 2 | 24 | 0 | 42 | 0,0340 | 0,0769 | non-defined |
| Rv3472 | Rv3472 | 70R>C | 2 | 24 | 0 | 42 | 0,0340 | 0,0769 | non-defined |
| Rv3479 | Rv3479 | 27N>T | 2 | 24 | 0 | 42 | 0,0340 | 0,0769 | non-defined |
| Rv3492c | Rv3492c | 74T>M | 2 | 24 | 0 | 42 | 0,0340 | 0,0769 | non-defined |
| Rv3494c | mce4F | 537G>V | 2 | 24 | 0 | 42 | 0,0340 | 0,0769 | non-defined |
| Rv3497c | mce4C | 324V>A | 2 | 24 | 0 | 42 | 0,0340 | 0,0769 | non-defined |
| Rv3506 | fadD17 | 197M>I | 2 | 24 | 0 | 42 | 0,0340 | 0,0769 | non-defined |
| Rv3507 | PE_PGRS53 | 1170A>G | 2 | 24 | 0 | 42 | 0,0340 | 0,0769 | non-defined |
| Rv3507 | PE_PGRS53 | 734G>D | 2 | 24 | 0 | 42 | 0,0340 | 0,0769 | non-defined |
| Rv3508 | PE_PGRS54 | 1244A>T | 2 | 24 | 0 | 42 | 0,0340 | 0,0769 | non-defined |
| Rv3529c | Rv3529c | 115P>A | 2 | 24 | 0 | 42 | 0,0340 | 0,0769 | non-defined |
| Rv3558 | PPE64 | 500A>T | 2 | 24 | 0 | 42 | 0,0340 | 0,0769 | non-defined |

| Rv3586 | Rv3586 | 69C>Y | 2 | 24 | 0 | 42 | 0,0340 | 0,0769 | non-defined |
| --- | --- | --- | --- | --- | --- | --- | --- | --- | --- |
| Rv3608c | folP1 | 263A>G | 2 | 24 | 0 | 42 | 0,0340 | 0,0769 | non-defined |
| Rv3653 | PE_PGRS61 | 178A>G | 2 | 24 | 0 | 42 | 0,0340 | 0,0769 | non-defined |
| Rv3659c | Rv3659c | 226R>W | 2 | 24 | 0 | 42 | 0,0340 | 0,0769 | non-defined |
| Rv3660c | Rv3660c | 16A>T | 2 | 24 | 0 | 42 | 0,0340 | 0,0769 | non-defined |
| Rv3711c | dnaQ | 88V>A | 2 | 24 | 0 | 42 | 0,0340 | 0,0769 | non-defined |
| Rv3746c | PE34 | 87S>L | 2 | 24 | 0 | 42 | 0,0340 | 0,0769 | non-defined |
| Rv3767c | Rv3767c | 90F>C | 2 | 24 | 0 | 42 | 0,0340 | 0,0769 | non-defined |
| Rv3795 | embB | 497Q>R | 2 | 24 | 0 | 42 | 0,0340 | 0,0769 | non-defined |
| Rv3797 | fadE35 | 93R>W | 2 | 24 | 0 | 42 | 0,0340 | 0,0769 | non-defined |
| Rv3825c | pks2 | 2051A>V | 2 | 24 | 0 | 42 | 0,0340 | 0,0769 | non-defined |
| Rv3869 | Rv3869 | 248P>T | 2 | 24 | 0 | 42 | 0,0340 | 0,0769 | non-defined |
| Rv3877 | Rv3877 | 11T>I | 2 | 24 | 0 | 42 | 0,0340 | 0,0769 | non-defined |
| Rv3883c | mycP1 | 159E>G | 2 | 24 | 0 | 42 | 0,0340 | 0,0769 | non-defined |
| Rv3884c | Rv3884c | 618V>I | 2 | 24 | 0 | 42 | 0,0340 | 0,0769 | non-defined |
| Rv3886c | mycP2 | 117G>S | 2 | 24 | 0 | 42 | 0,0340 | 0,0769 | non-defined |

| Rv3894c | Rv3894c | 615Y>* | 2 | 24 | 0 | 42 | 0,0340 | 0,0769 | non-defined |
| --- | --- | --- | --- | --- | --- | --- | --- | --- | --- |
| Rv3907c | pcnA | 144A>V | 2 | 24 | 0 | 42 | 0,0340 | 0,0769 | non-defined |
| Rv3908 | Rv3908 | 28H>R | 2 | 24 | 0 | 42 | 0,0340 | 0,0769 | non-defined |
| Rv3919c | gidB | 67D>G | 2 | 24 | 0 | 42 | 0,0340 | 0,0769 | non-defined |
| Rv3919c | gidB | 79L>W | 2 | 24 | 0 | 42 | 0,0340 | 0,0769 | non-defined |
| Rv3919c | gidB | 96R>L | 2 | 24 | 0 | 42 | 0,0340 | 0,0769 | non-defined |
| Rv2741 | PE_PGRS47 | 271S>G | 21 | 5 | 25 | 17 | 0,0344 | 0,2125 | non-defined |
| Rv0833 | PE_PGRS13 | 584S>G | 17 | 9 | 18 | 24 | 0,0354 | 0,2253 | non-defined |
| Rv0095c | Rv0095c | 85A>V | 15 | 11 | 15 | 27 | 0,0380 | 0,2198 | non-defined |
| Rv0095c | Rv0095c | 92Q>G | 15 | 11 | 15 | 27 | 0,0380 | 0,2198 | non-defined |
| Rv0095c | Rv0095c | 91A>V | 14 | 12 | 14 | 28 | 0,0474 | 0,2051 | non-defined |
| Rv0109 | PE_PGRS1 | 346R>G | 26 | 0 | 38 | 4 | 0,0524 | 0,0952 | non-defined |
| Rv2566 | Rv2566 | 610L>P | 26 | 0 | 38 | 4 | 0,0524 | 0,0952 | non-defined |
| Rv2177c | Rv2177c | 163I>T | 7 | 19 | 5 | 37 | 0,0572 | 0,1502 | 2,7263 |
| Rv2177c | Rv2177c | 164A>T | 7 | 19 | 5 | 37 | 0,0572 | 0,1502 | 2,7263 |
| Rv3512 | PE_PGRS56 | 306I>T | 7 | 19 | 5 | 37 | 0,0572 | 0,1502 | 2,7263 |
| Rv0086 | hycQ | 307A>P | 3 | 23 | 1 | 41 | 0,0594 | 0,0916 | 5,3478 |
| Rv0096 | PPE1 | 360D>E | 3 | 23 | 1 | 41 | 0,0594 | 0,0916 | 5,3478 |
| Rv0131c | fadE1 | 290M>L | 3 | 23 | 1 | 41 | 0,0594 | 0,0916 | 5,3478 |
| Rv0132c | fgd2 | 329L>V | 3 | 23 | 1 | 41 | 0,0594 | 0,0916 | 5,3478 |

| Rv0134 | ephF | 139K>M | 3 | 23 | 1 | 41 | 0,0594 | 0,0916 | 5,3478 |
| --- | --- | --- | --- | --- | --- | --- | --- | --- | --- |
| Rv0167 | yrbE1A | 63M>V | 3 | 23 | 1 | 41 | 0,0594 | 0,0916 | 5,3478 |
| Rv0171 | mce1C | 97I>V | 3 | 23 | 1 | 41 | 0,0594 | 0,0916 | 5,3478 |
| Rv0244c | fadE5 | 8V>L | 3 | 23 | 1 | 41 | 0,0594 | 0,0916 | 5,3478 |
| Rv0278c | PE_PGRS3 | 803L>I | 3 | 23 | 1 | 41 | 0,0594 | 0,0916 | 5,3478 |
| Rv0297 | PE_PGRS5 | 10M>T | 3 | 23 | 1 | 41 | 0,0594 | 0,0916 | 5,3478 |
| Rv0327c | cyp135A1 | 89E>A | 3 | 23 | 1 | 41 | 0,0594 | 0,0916 | 5,3478 |
| Rv0370c | Rv0370c | 116D>N | 3 | 23 | 1 | 41 | 0,0594 | 0,0916 | 5,3478 |
| Rv0423c | thiC | 520E>K | 3 | 23 | 1 | 41 | 0,0594 | 0,0916 | 5,3478 |
| Rv0446c | Rv0446c | 57W>C | 3 | 23 | 1 | 41 | 0,0594 | 0,0916 | 5,3478 |
| Rv0667 | rpoB | 435D>V | 3 | 23 | 1 | 41 | 0,0594 | 0,0916 | 5,3478 |
| Rv0668 | rpoC | 483V>G | 3 | 23 | 1 | 41 | 0,0594 | 0,0916 | 5,3478 |
| Rv0668 | rpoC | 741R>S | 3 | 23 | 1 | 41 | 0,0594 | 0,0916 | 5,3478 |
| Rv0698 | Rv0698 | 34R>L | 3 | 23 | 1 | 41 | 0,0594 | 0,0916 | 5,3478 |
| Rv0725c | Rv0724A | 71A>S | 3 | 23 | 1 | 41 | 0,0594 | 0,0916 | 5,3478 |
| Rv0739 | Rv0739 | 118Q>* | 3 | 23 | 1 | 41 | 0,0594 | 0,0916 | 5,3478 |
| Rv0854 | Rv0854 | 50S>T | 3 | 23 | 1 | 41 | 0,0594 | 0,0916 | 5,3478 |
| Rv0989c | grcC2 | 245Y>C | 3 | 23 | 1 | 41 | 0,0594 | 0,0916 | 5,3478 |
| Rv1028c | kdpD | 515A>D | 3 | 23 | 1 | 41 | 0,0594 | 0,0916 | 5,3478 |
| Rv1092c | coaA | 300A>T | 3 | 23 | 1 | 41 | 0,0594 | 0,0916 | 5,3478 |
| Rv1128c | Rv1128c | 340A>T | 3 | 23 | 1 | 41 | 0,0594 | 0,0916 | 5,3478 |
| Rv1148c | Rv1148c | 356N>H | 3 | 23 | 1 | 41 | 0,0594 | 0,0916 | 5,3478 |
| Rv1148c | Rv1148c | 7L>I | 3 | 23 | 1 | 41 | 0,0594 | 0,0916 | 5,3478 |
| Rv1207 | folP2 | 290E>K | 3 | 23 | 1 | 41 | 0,0594 | 0,0916 | 5,3478 |
| Rv1221 | sigE | 8R>W | 3 | 23 | 1 | 41 | 0,0594 | 0,0916 | 5,3478 |
| Rv1231c | Rv1231c | 143R>C | 3 | 23 | 1 | 41 | 0,0594 | 0,0916 | 5,3478 |
| Rv1255c | Rv1255c | 10R>C | 3 | 23 | 1 | 41 | 0,0594 | 0,0916 | 5,3478 |
| Rv1286 | cysN | 269A>T | 3 | 23 | 1 | 41 | 0,0594 | 0,0916 | 5,3478 |
| Rv1337 | Rv1337 | 142P>T | 3 | 23 | 1 | 41 | 0,0594 | 0,0916 | 5,3478 |
| Rv1438 | tpiA | 256A>V | 3 | 23 | 1 | 41 | 0,0594 | 0,0916 | 5,3478 |
| Rv1727 | Rv1727 | 127W>R | 3 | 23 | 1 | 41 | 0,0594 | 0,0916 | 5,3478 |
| Rv1747 | Rv1747 | 285H>D | 3 | 23 | 1 | 41 | 0,0594 | 0,0916 | 5,3478 |
| Rv1774 | Rv1774 | 271E>K | 3 | 23 | 1 | 41 | 0,0594 | 0,0916 | 5,3478 |

| Rv1848 | ureA | 36I>L | 3 | 23 | 1 | 41 | 0,0594 | 0,0916 | 5,3478 |
| --- | --- | --- | --- | --- | --- | --- | --- | --- | --- |
| Rv1883c | Rv1883c | 9G>C | 3 | 23 | 1 | 41 | 0,0594 | 0,0916 | 5,3478 |
| Rv1901 | cinA | 244P>T | 3 | 23 | 1 | 41 | 0,0594 | 0,0916 | 5,3478 |
| Rv1931c | Rv1931c | 6F>L | 3 | 23 | 1 | 41 | 0,0594 | 0,0916 | 5,3478 |
| Rv1965 | yrbE3B | 108V>A | 3 | 23 | 1 | 41 | 0,0594 | 0,0916 | 5,3478 |
| Rv1967 | mce3B | 47N>T | 3 | 23 | 1 | 41 | 0,0594 | 0,0916 | 5,3478 |
| Rv1991c | Rv1991c | 32S>A | 3 | 23 | 1 | 41 | 0,0594 | 0,0916 | 5,3478 |
| Rv2019 | Rv2019 | 47Q>P | 3 | 23 | 1 | 41 | 0,0594 | 0,0916 | 5,3478 |
| Rv2021c | Rv2021c | 72Q>* | 3 | 23 | 1 | 41 | 0,0594 | 0,0916 | 5,3478 |
| Rv2046 | lppI | 59E>D | 3 | 23 | 1 | 41 | 0,0594 | 0,0916 | 5,3478 |
| Rv2052c | Rv2052c | 422V>I | 3 | 23 | 1 | 41 | 0,0594 | 0,0916 | 5,3478 |
| Rv2101 | helZ | 219G>R | 3 | 23 | 1 | 41 | 0,0594 | 0,0916 | 5,3478 |
| Rv2101 | helZ | 470E>A | 3 | 23 | 1 | 41 | 0,0594 | 0,0916 | 5,3478 |
| Rv2275 | Rv2275 | 100R>Q | 3 | 23 | 1 | 41 | 0,0594 | 0,0916 | 5,3478 |
| Rv2339 | mmpL9 | 483G>A | 3 | 23 | 1 | 41 | 0,0594 | 0,0916 | 5,3478 |
| Rv2425c | Rv2425c | 27A>V | 3 | 23 | 1 | 41 | 0,0594 | 0,0916 | 5,3478 |
| Rv2483c | plsC | 151I>V | 3 | 23 | 1 | 41 | 0,0594 | 0,0916 | 5,3478 |
| Rv2505c | fadD35 | 57S>R | 3 | 23 | 1 | 41 | 0,0594 | 0,0916 | 5,3478 |
| Rv2565 | Rv2565 | 248D>N | 3 | 23 | 1 | 41 | 0,0594 | 0,0916 | 5,3478 |
| Rv2574 | Rv2574 | 146L>P | 3 | 23 | 1 | 41 | 0,0594 | 0,0916 | 5,3478 |
| Rv2685 | arsB1 | 47Y>* | 3 | 23 | 1 | 41 | 0,0594 | 0,0916 | 5,3478 |
| Rv2733c | Rv2733c | 269F>L | 3 | 23 | 1 | 41 | 0,0594 | 0,0916 | 5,3478 |
| Rv2783c | gpsI | 678D>A | 3 | 23 | 1 | 41 | 0,0594 | 0,0916 | 5,3478 |
| Rv2813 | Rv2813 | 251D>E | 3 | 23 | 1 | 41 | 0,0594 | 0,0916 | 5,3478 |
| Rv2881c | cdsA | 34L>V | 3 | 23 | 1 | 41 | 0,0594 | 0,0916 | 5,3478 |
| Rv3092c | Rv3092c | 41V>L | 3 | 23 | 1 | 41 | 0,0594 | 0,0916 | 5,3478 |
| Rv3104c | Rv3104c | 14Q>* | 3 | 23 | 1 | 41 | 0,0594 | 0,0916 | 5,3478 |
| Rv3224 | Rv3224 | 209E>D | 3 | 23 | 1 | 41 | 0,0594 | 0,0916 | 5,3478 |
| Rv3328c | sigJ | 255G>D | 3 | 23 | 1 | 41 | 0,0594 | 0,0916 | 5,3478 |
| Rv3421c | Rv3421c | 65L>M | 3 | 23 | 1 | 41 | 0,0594 | 0,0916 | 5,3478 |
| Rv3454 | Rv3454 | 243F>L | 3 | 23 | 1 | 41 | 0,0594 | 0,0916 | 5,3478 |
| Rv3579c | Rv3579c | 30R>S | 3 | 23 | 1 | 41 | 0,0594 | 0,0916 | 5,3478 |
| Rv3657c | Rv3657c | 165F>V | 3 | 23 | 1 | 41 | 0,0594 | 0,0916 | 5,3478 |

| Rv3664c | dppC | 59G>S | 3 | 23 | 1 | 41 | 0,0594 | 0,0916 | 5,3478 |
| --- | --- | --- | --- | --- | --- | --- | --- | --- | --- |
| Rv3762c | Rv3762c | 545F>C | 3 | 23 | 1 | 41 | 0,0594 | 0,0916 | 5,3478 |
| Rv3823c | mmpL8 | 501L>V | 3 | 23 | 1 | 41 | 0,0594 | 0,0916 | 5,3478 |
| Rv3860 | Rv3860 | 77T>P | 3 | 23 | 1 | 41 | 0,0594 | 0,0916 | 5,3478 |
| Rv3869 | Rv3869 | 128V>F | 3 | 23 | 1 | 41 | 0,0594 | 0,0916 | 5,3478 |
| Rv3922c | Rv3922c | 36P>L | 3 | 23 | 1 | 41 | 0,0594 | 0,0916 | 5,3478 |
| Rv0032 | bioF2 | 268I>T | 5 | 21 | 3 | 39 | 0,0664 | 0,1209 | 3,0952 |
| Rv0092 | ctpA | 382G>E | 5 | 21 | 3 | 39 | 0,0664 | 0,1209 | 3,0952 |
| Rv0149 | Rv0149 | 202H>Y | 5 | 21 | 3 | 39 | 0,0664 | 0,1209 | 3,0952 |
| Rv0399c | lpqK | 67E>K | 5 | 21 | 3 | 39 | 0,0664 | 0,1209 | 3,0952 |
| Rv0466 | Rv0466 | 226V>I | 5 | 21 | 3 | 39 | 0,0664 | 0,1209 | 3,0952 |
| Rv0574c | Rv0574c | 246D>N | 5 | 21 | 3 | 39 | 0,0664 | 0,1209 | 3,0952 |
| Rv0638 | secE | 21S>T | 5 | 21 | 3 | 39 | 0,0664 | 0,1209 | 3,0952 |
| Rv0933 | pstB | 61T>M | 5 | 21 | 3 | 39 | 0,0664 | 0,1209 | 3,0952 |
| Rv1148c | Rv1148c | 271L>V | 5 | 21 | 3 | 39 | 0,0664 | 0,1209 | 3,0952 |
| Rv1155 | Rv1154c | 14V>V | 5 | 21 | 3 | 39 | 0,0664 | 0,1209 | 3,0952 |
| Rv1288 | Rv1288 | 62D>E | 5 | 21 | 3 | 39 | 0,0664 | 0,1209 | 3,0952 |
| Rv1450c | PE_PGRS27 | 7A>T | 5 | 21 | 3 | 39 | 0,0664 | 0,1209 | 3,0952 |
| Rv1500 | Rv1500 | 317T>I | 5 | 21 | 3 | 39 | 0,0664 | 0,1209 | 3,0952 |
| Rv1518 | Rv1518 | 86N>H | 5 | 21 | 3 | 39 | 0,0664 | 0,1209 | 3,0952 |
| Rv1650 | pheT | 506R>H | 5 | 21 | 3 | 39 | 0,0664 | 0,1209 | 3,0952 |
| Rv1877 | Rv1877 | 155V>L | 5 | 21 | 3 | 39 | 0,0664 | 0,1209 | 3,0952 |
| Rv2008c | Rv2008c | 55I>L | 5 | 21 | 3 | 39 | 0,0664 | 0,1209 | 3,0952 |
| Rv2177c | Rv2177c | 183Y>H | 5 | 21 | 3 | 39 | 0,0664 | 0,1209 | 3,0952 |
| Rv2252 | Rv2252 | 230G>S | 5 | 21 | 3 | 39 | 0,0664 | 0,1209 | 3,0952 |
| Rv2326c | Rv2326c | 43A>S | 5 | 21 | 3 | 39 | 0,0664 | 0,1209 | 3,0952 |
| Rv2570 | Rv2570 | 115Q>* | 5 | 21 | 3 | 39 | 0,0664 | 0,1209 | 3,0952 |
| Rv2617c | Rv2617c | 44N>S | 5 | 21 | 3 | 39 | 0,0664 | 0,1209 | 3,0952 |
| Rv2790c | ltp1 | 313F>V | 5 | 21 | 3 | 39 | 0,0664 | 0,1209 | 3,0952 |
| Rv2897c | Rv2897c | 216L>M | 5 | 21 | 3 | 39 | 0,0664 | 0,1209 | 3,0952 |
| Rv2959c | Rv2959c | 69W>* | 5 | 21 | 3 | 39 | 0,0664 | 0,1209 | 3,0952 |
| Rv3236c | Rv3236c | 370A>T | 5 | 21 | 3 | 39 | 0,0664 | 0,1209 | 3,0952 |
| Rv3463 | Rv3463 | 94G>D | 5 | 21 | 3 | 39 | 0,0664 | 0,1209 | 3,0952 |

| Rv3795 | embB | 306M>I | 5 | 21 | 3 | 39 | 0,0664 | 0,1209 | 3,0952 |
| --- | --- | --- | --- | --- | --- | --- | --- | --- | --- |
| Rv3838c | pheA | 267H>Y | 5 | 21 | 3 | 39 | 0,0664 | 0,1209 | 3,0952 |
| Rv3888c | Rv3888c | 16I>V | 5 | 21 | 3 | 39 | 0,0664 | 0,1209 | 3,0952 |
| Rv3897c | Rv3897c | 183C>R | 5 | 21 | 3 | 39 | 0,0664 | 0,1209 | 3,0952 |
| Rv3512 | PE_PGRS56 | 892A>T | 4 | 22 | 2 | 40 | 0,0667 | 0,1062 | 3,6364 |
| Rv0015c | pknA | 385S>R | 12 | 14 | 12 | 30 | 0,0702 | 0,1758 | 2,1429 |
| Rv0095c | Rv0095c | 57D>E | 12 | 14 | 12 | 30 | 0,0702 | 0,1758 | 2,1429 |
| Rv0642c | mmaA4 | 165N>S | 12 | 14 | 12 | 30 | 0,0702 | 0,1758 | 2,1429 |
| Rv0948c | Rv0948c | 59K>T | 12 | 14 | 12 | 30 | 0,0702 | 0,1758 | 2,1429 |
| Rv1569 | bioF1 | 171A>G | 12 | 14 | 12 | 30 | 0,0702 | 0,1758 | 2,1429 |
| Rv1606 | hisI | 99T>I | 12 | 14 | 12 | 30 | 0,0702 | 0,1758 | 2,1429 |
| Rv1884c | rpfC | 16H>R | 12 | 14 | 12 | 30 | 0,0702 | 0,1758 | 2,1429 |
| Rv2022c | Rv2022c | 118V>A | 12 | 14 | 12 | 30 | 0,0702 | 0,1758 | 2,1429 |
| Rv2716 | Rv2716 | 147A>T | 12 | 14 | 12 | 30 | 0,0702 | 0,1758 | 2,1429 |
| Rv1319c | Rv1319c | 439D>E | 23 | 3 | 31 | 11 | 0,0732 | 0,1465 | 2,7204 |
| Rv2512c | Rv2512c | 328Q>K | 17 | 9 | 20 | 22 | 0,0764 | 0,1777 | 2,0778 |
| Rv0159c | PE3 | 14T>A | 26 | 0 | 39 | 3 | 0,0817 | 0,0714 | non-defined |
| Rv0787 | Rv0787 | 267Y>H | 26 | 0 | 39 | 3 | 0,0817 | 0,0714 | non-defined |
| Rv1498c | Rv1498c | 191R>H | 26 | 0 | 39 | 3 | 0,0817 | 0,0714 | non-defined |
| Rv1502 | Rv1502 | 213Y>C | 26 | 0 | 39 | 3 | 0,0817 | 0,0714 | non-defined |
| Rv1548c | PPE21 | 258D>G | 26 | 0 | 39 | 3 | 0,0817 | 0,0714 | non-defined |
| Rv1809 | PPE33 | 252V>L | 26 | 0 | 39 | 3 | 0,0817 | 0,0714 | non-defined |
| Rv2079 | Rv2079 | 47Y>C | 26 | 0 | 39 | 3 | 0,0817 | 0,0714 | non-defined |
| Rv2226 | Rv2226 | 299D>N | 26 | 0 | 39 | 3 | 0,0817 | 0,0714 | non-defined |

| Rv3093c | Rv3093c | 210C>W | 26 | 0 | 39 | 3 | 0,0817 | 0,0714 | non-defined |
| --- | --- | --- | --- | --- | --- | --- | --- | --- | --- |
| Rv0747 | PE_PGRS10 | 225R>G | 15 | 11 | 17 | 25 | 0,0835 | 0,1722 | 2,0053 |
| Rv0747 | PE_PGRS10 | 227R>G | 15 | 11 | 17 | 25 | 0,0835 | 0,1722 | 2,0053 |
| Rv0747 | PE_PGRS10 | 295K>R | 15 | 11 | 17 | 25 | 0,0835 | 0,1722 | 2,0053 |
| Rv0747 | PE_PGRS10 | 300S>G | 15 | 11 | 17 | 25 | 0,0835 | 0,1722 | 2,0053 |
| Rv0355c | PPE8 | 2591W>G | 25 | 1 | 36 | 6 | 0,0843 | 0,1044 | 4,1667 |
| Rv0004 | Rv0004 | 187G>V | 1 | 25 | 0 | 42 | 0,1002 | 0,0385 | non-defined |
| Rv0006 | gyrA | 105L>R | 1 | 25 | 0 | 42 | 0,1002 | 0,0385 | non-defined |
| Rv0006 | gyrA | 94D>H | 1 | 25 | 0 | 42 | 0,1002 | 0,0385 | non-defined |
| Rv0006 | gyrA | 94D>Y | 1 | 25 | 0 | 42 | 0,1002 | 0,0385 | non-defined |
| Rv0010c | Rv0010c | 36G>V | 1 | 25 | 0 | 42 | 0,1002 | 0,0385 | non-defined |
| Rv0016c | pbpA | 469A>T | 1 | 25 | 0 | 42 | 0,1002 | 0,0385 | non-defined |
| Rv0021c | Rv0021c | 147G>S | 1 | 25 | 0 | 42 | 0,1002 | 0,0385 | non-defined |
| Rv0028 | Rv0028 | 23R>L | 1 | 25 | 0 | 42 | 0,1002 | 0,0385 | non-defined |
| Rv0050 | ponA1 | 41I>M | 1 | 25 | 0 | 42 | 0,1002 | 0,0385 | non-defined |
| Rv0058 | dnaB | 345K>N | 1 | 25 | 0 | 42 | 0,1002 | 0,0385 | non-defined |
| Rv0060 | Rv0060 | 101R>C | 1 | 25 | 0 | 42 | 0,1002 | 0,0385 | non-defined |
| Rv0064 | Rv0064 | 536S>* | 1 | 25 | 0 | 42 | 0,1002 | 0,0385 | non-defined |
| Rv0069c | sdaA | 262D>G | 1 | 25 | 0 | 42 | 0,1002 | 0,0385 | non-defined |

| Rv0074 | Rv0074 | 255A>T | 1 | 25 | 0 | 42 | 0,1002 | 0,0385 | non-defined |
| --- | --- | --- | --- | --- | --- | --- | --- | --- | --- |
| Rv0074 | Rv0074 | 29F>L | 1 | 25 | 0 | 42 | 0,1002 | 0,0385 | non-defined |
| Rv0075 | Rv0075 | 140V>A | 1 | 25 | 0 | 42 | 0,1002 | 0,0385 | non-defined |
| Rv0077c | Rv0077c | 218P>L | 1 | 25 | 0 | 42 | 0,1002 | 0,0385 | non-defined |
| Rv0083 | Rv0083 | 573H>Y | 1 | 25 | 0 | 42 | 0,1002 | 0,0385 | non-defined |
| Rv0084 | hycD | 68P>L | 1 | 25 | 0 | 42 | 0,1002 | 0,0385 | non-defined |
| Rv0088 | Rv0088 | 154W>C | 1 | 25 | 0 | 42 | 0,1002 | 0,0385 | non-defined |
| Rv0095c | Rv0095c | 26G>V | 1 | 25 | 0 | 42 | 0,1002 | 0,0385 | non-defined |
| Rv0095c | Rv0095c | 70S>T | 1 | 25 | 0 | 42 | 0,1002 | 0,0385 | non-defined |
| Rv0099 | fadD10 | 424S>F | 1 | 25 | 0 | 42 | 0,1002 | 0,0385 | non-defined |
| Rv0107c | ctpI | 920M>I | 1 | 25 | 0 | 42 | 0,1002 | 0,0385 | non-defined |
| Rv0110 | Rv0110 | 111G>D | 1 | 25 | 0 | 42 | 0,1002 | 0,0385 | non-defined |
| Rv0118c | oxcA | 99T>P | 1 | 25 | 0 | 42 | 0,1002 | 0,0385 | non-defined |
| Rv0120c | fusA2 | 172V>L | 1 | 25 | 0 | 42 | 0,1002 | 0,0385 | non-defined |
| Rv0120c | fusA2 | 263V>M | 1 | 25 | 0 | 42 | 0,1002 | 0,0385 | non-defined |
| Rv0121c | Rv0121c | 120A>T | 1 | 25 | 0 | 42 | 0,1002 | 0,0385 | non-defined |

| Rv0122 | Rv0122 | 3G>A | 1 | 25 | 0 | 42 | 0,1002 | 0,0385 | non-defined |
| --- | --- | --- | --- | --- | --- | --- | --- | --- | --- |
| Rv0124 | PE_PGRS2 | 269G>A | 1 | 25 | 0 | 42 | 0,1002 | 0,0385 | non-defined |
| Rv0127 | Rv0127 | 194G>R | 1 | 25 | 0 | 42 | 0,1002 | 0,0385 | non-defined |
| Rv0134 | ephF | 87A>P | 1 | 25 | 0 | 42 | 0,1002 | 0,0385 | non-defined |
| Rv0136 | cyp138 | 286P>L | 1 | 25 | 0 | 42 | 0,1002 | 0,0385 | non-defined |
| Rv0143c | Rv0143c | 176A>S | 1 | 25 | 0 | 42 | 0,1002 | 0,0385 | non-defined |
| Rv0151c | PE1 | 320E>K | 1 | 25 | 0 | 42 | 0,1002 | 0,0385 | non-defined |
| Rv0159c | PE3 | 368R>W | 1 | 25 | 0 | 42 | 0,1002 | 0,0385 | non-defined |
| Rv0170 | mce1B | 284E>D | 1 | 25 | 0 | 42 | 0,1002 | 0,0385 | non-defined |
| Rv0184 | Rv0184 | 176D>N | 1 | 25 | 0 | 42 | 0,1002 | 0,0385 | non-defined |
| Rv0193c | Rv0193c | 165G>D | 1 | 25 | 0 | 42 | 0,1002 | 0,0385 | non-defined |
| Rv0194 | Rv0194 | 968A>V | 1 | 25 | 0 | 42 | 0,1002 | 0,0385 | non-defined |
| Rv0198c | Rv0198c | 84Y>C | 1 | 25 | 0 | 42 | 0,1002 | 0,0385 | non-defined |
| Rv0202c | mmpL11 | 263C>R | 1 | 25 | 0 | 42 | 0,1002 | 0,0385 | non-defined |
| Rv0210 | Rv0210 | 450R>L | 1 | 25 | 0 | 42 | 0,1002 | 0,0385 | non-defined |
| Rv0211 | pckA | 386P>S | 1 | 25 | 0 | 42 | 0,1002 | 0,0385 | non-defined |

| Rv0213c | Rv0213c | 38A>D | 1 | 25 | 0 | 42 | 0,1002 | 0,0385 | non-defined |
| --- | --- | --- | --- | --- | --- | --- | --- | --- | --- |
| Rv0214 | fadD4 | 373D>G | 1 | 25 | 0 | 42 | 0,1002 | 0,0385 | non-defined |
| Rv0222 | echA1 | 73M>I | 1 | 25 | 0 | 42 | 0,1002 | 0,0385 | non-defined |
| Rv0224c | Rv0224c | 42A>V | 1 | 25 | 0 | 42 | 0,1002 | 0,0385 | non-defined |
| Rv0226c | Rv0226c | 556G>S | 1 | 25 | 0 | 42 | 0,1002 | 0,0385 | non-defined |
| Rv0232 | Rv0232 | 12A>V | 1 | 25 | 0 | 42 | 0,1002 | 0,0385 | non-defined |
| Rv0237 | lpqI | 258T>M | 1 | 25 | 0 | 42 | 0,1002 | 0,0385 | non-defined |
| Rv0237 | lpqI | 365P>S | 1 | 25 | 0 | 42 | 0,1002 | 0,0385 | non-defined |
| Rv0252 | nirB | 605G>V | 1 | 25 | 0 | 42 | 0,1002 | 0,0385 | non-defined |
| Rv0255c | cobQ1 | 458L>V | 1 | 25 | 0 | 42 | 0,1002 | 0,0385 | non-defined |
| Rv0258c | Rv0258c | 51T>A | 1 | 25 | 0 | 42 | 0,1002 | 0,0385 | non-defined |
| Rv0259c | Rv0259c | 59I>V | 1 | 25 | 0 | 42 | 0,1002 | 0,0385 | non-defined |
| Rv0261c | narK3 | 126L>V | 1 | 25 | 0 | 42 | 0,1002 | 0,0385 | non-defined |
| Rv0263c | Rv0263c | 84V>M | 1 | 25 | 0 | 42 | 0,1002 | 0,0385 | non-defined |
| Rv0266c | oplA | 373P>S | 1 | 25 | 0 | 42 | 0,1002 | 0,0385 | non-defined |
| Rv0277c | Rv0277c | 117E>G | 1 | 25 | 0 | 42 | 0,1002 | 0,0385 | non-defined |

| Rv0278c | PE_PGRS3 | 77V>T | 1 | 25 | 0 | 42 | 0,1002 | 0,0385 | non-defined |
| --- | --- | --- | --- | --- | --- | --- | --- | --- | --- |
| Rv0278c | PE_PGRS3 | 841S>A | 1 | 25 | 0 | 42 | 0,1002 | 0,0385 | non-defined |
| Rv0280 | PPE3 | 256Q>R | 1 | 25 | 0 | 42 | 0,1002 | 0,0385 | non-defined |
| Rv0290 | Rv0290 | 207G>R | 1 | 25 | 0 | 42 | 0,1002 | 0,0385 | non-defined |
| Rv0292 | Rv0292 | 323P>L | 1 | 25 | 0 | 42 | 0,1002 | 0,0385 | non-defined |
| Rv0297 | PE_PGRS5 | 158A>T | 1 | 25 | 0 | 42 | 0,1002 | 0,0385 | non-defined |
| Rv0297 | PE_PGRS5 | 445A>V | 1 | 25 | 0 | 42 | 0,1002 | 0,0385 | non-defined |
| Rv0310c | Rv0310c | 34W>C | 1 | 25 | 0 | 42 | 0,1002 | 0,0385 | non-defined |
| Rv0338c | Rv0338c | 31Y>S | 1 | 25 | 0 | 42 | 0,1002 | 0,0385 | non-defined |
| Rv0339c | Rv0339c | 427M>R | 1 | 25 | 0 | 42 | 0,1002 | 0,0385 | non-defined |
| Rv0352 | dnaJ1 | 333G>S | 1 | 25 | 0 | 42 | 0,1002 | 0,0385 | non-defined |
| Rv0355c | PPE8 | 2150I>T | 1 | 25 | 0 | 42 | 0,1002 | 0,0385 | non-defined |
| Rv0355c | PPE8 | 2151A>Y | 1 | 25 | 0 | 42 | 0,1002 | 0,0385 | non-defined |
| Rv0355c | PPE8 | 2155S>G | 1 | 25 | 0 | 42 | 0,1002 | 0,0385 | non-defined |
| Rv0355c | PPE8 | 2545I>V | 1 | 25 | 0 | 42 | 0,1002 | 0,0385 | non-defined |
| Rv0355c | PPE8 | 2750Y>H | 1 | 25 | 0 | 42 | 0,1002 | 0,0385 | non-defined |

| Rv0355c | PPE8 | 2937G>R | 1 | 25 | 0 | 42 | 0,1002 | 0,0385 | non-defined |
| --- | --- | --- | --- | --- | --- | --- | --- | --- | --- |
| Rv0355c | PPE8 | 715G>D | 1 | 25 | 0 | 42 | 0,1002 | 0,0385 | non-defined |
| Rv0357c | purA | 339E>K | 1 | 25 | 0 | 42 | 0,1002 | 0,0385 | non-defined |
| Rv0362 | mgtE | 317E>Q | 1 | 25 | 0 | 42 | 0,1002 | 0,0385 | non-defined |
| Rv0362 | mgtE | 77A>S | 1 | 25 | 0 | 42 | 0,1002 | 0,0385 | non-defined |
| Rv0386 | Rv0386 | 894A>E | 1 | 25 | 0 | 42 | 0,1002 | 0,0385 | non-defined |
| Rv0392c | ndhA | 422S>F | 1 | 25 | 0 | 42 | 0,1002 | 0,0385 | non-defined |
| Rv0422c | thiD | 113H>R | 1 | 25 | 0 | 42 | 0,1002 | 0,0385 | non-defined |
| Rv0425c | ctpH | 644D>G | 1 | 25 | 0 | 42 | 0,1002 | 0,0385 | non-defined |
| Rv0448c | Rv0448c | 51R>C | 1 | 25 | 0 | 42 | 0,1002 | 0,0385 | non-defined |
| Rv0470A | Rv0470A | 81W>R | 1 | 25 | 0 | 42 | 0,1002 | 0,0385 | non-defined |
| Rv0493c | Rv0493c | 92A>V | 1 | 25 | 0 | 42 | 0,1002 | 0,0385 | non-defined |
| Rv0496 | Rv0496 | 297E>D | 1 | 25 | 0 | 42 | 0,1002 | 0,0385 | non-defined |
| Rv0527 | ccdA | 127L>V | 1 | 25 | 0 | 42 | 0,1002 | 0,0385 | non-defined |
| Rv0537c | Rv0537c | 214A>P | 1 | 25 | 0 | 42 | 0,1002 | 0,0385 | non-defined |
| Rv0564c | gpsA | 58D>Y | 1 | 25 | 0 | 42 | 0,1002 | 0,0385 | non-defined |

| Rv0567 | Rv0567 | 1V>A | 1 | 25 | 0 | 42 | 0,1002 | 0,0385 | non-defined |
| --- | --- | --- | --- | --- | --- | --- | --- | --- | --- |
| Rv0584 | Rv0584 | 103S>L | 1 | 25 | 0 | 42 | 0,1002 | 0,0385 | non-defined |
| Rv0591 | mce2C | 100D>E | 1 | 25 | 0 | 42 | 0,1002 | 0,0385 | non-defined |
| Rv0591 | mce2C | 199N>K | 1 | 25 | 0 | 42 | 0,1002 | 0,0385 | non-defined |
| Rv0611c | Rv0611c | 113R>* | 1 | 25 | 0 | 42 | 0,1002 | 0,0385 | non-defined |
| Rv0631c | recC | 747R>W | 1 | 25 | 0 | 42 | 0,1002 | 0,0385 | non-defined |
| Rv0646c | lipG | 264R>P | 1 | 25 | 0 | 42 | 0,1002 | 0,0385 | non-defined |
| Rv0667 | rpoB | 250E>G | 1 | 25 | 0 | 42 | 0,1002 | 0,0385 | non-defined |
| Rv0668 | rpoC | 1252V>L | 1 | 25 | 0 | 42 | 0,1002 | 0,0385 | non-defined |
| Rv0668 | rpoC | 445K>R | 1 | 25 | 0 | 42 | 0,1002 | 0,0385 | non-defined |
| Rv0668 | rpoC | 452F>L | 1 | 25 | 0 | 42 | 0,1002 | 0,0385 | non-defined |
| Rv0668 | rpoC | 507L>V | 1 | 25 | 0 | 42 | 0,1002 | 0,0385 | non-defined |
| Rv0668 | rpoC | 518E>D | 1 | 25 | 0 | 42 | 0,1002 | 0,0385 | non-defined |
| Rv0669c | Rv0669c | 32G>C | 1 | 25 | 0 | 42 | 0,1002 | 0,0385 | non-defined |
| Rv0682 | rpsL | 88K>M | 1 | 25 | 0 | 42 | 0,1002 | 0,0385 | non-defined |
| Rv0683 | rpsG | 105V>I | 1 | 25 | 0 | 42 | 0,1002 | 0,0385 | non-defined |

| Rv0684 | fusA1 | 539G>E | 1 | 25 | 0 | 42 | 0,1002 | 0,0385 | non-defined |
| --- | --- | --- | --- | --- | --- | --- | --- | --- | --- |
| Rv0696 | Rv0696 | 414V>M | 1 | 25 | 0 | 42 | 0,1002 | 0,0385 | non-defined |
| Rv0705 | rpsS | 69H>R | 1 | 25 | 0 | 42 | 0,1002 | 0,0385 | non-defined |
| Rv0739 | Rv0739 | 216T>A | 1 | 25 | 0 | 42 | 0,1002 | 0,0385 | non-defined |
| Rv0747 | PE_PGRS10 | 515L>F | 1 | 25 | 0 | 42 | 0,1002 | 0,0385 | non-defined |
| Rv0747 | PE_PGRS10 | 517A>S | 1 | 25 | 0 | 42 | 0,1002 | 0,0385 | non-defined |
| Rv0747 | PE_PGRS10 | 518A>G | 1 | 25 | 0 | 42 | 0,1002 | 0,0385 | non-defined |
| Rv0747 | PE_PGRS10 | 523A>S | 1 | 25 | 0 | 42 | 0,1002 | 0,0385 | non-defined |
| Rv0747 | PE_PGRS10 | 580A>N | 1 | 25 | 0 | 42 | 0,1002 | 0,0385 | non-defined |
| Rv0747 | PE_PGRS10 | 782S>A | 1 | 25 | 0 | 42 | 0,1002 | 0,0385 | non-defined |
| Rv0756c | Rv0756c | 112T>P | 1 | 25 | 0 | 42 | 0,1002 | 0,0385 | non-defined |
| Rv0757 | phoP | 102D>N | 1 | 25 | 0 | 42 | 0,1002 | 0,0385 | non-defined |
| Rv0775 | Rv0775 | 28E>K | 1 | 25 | 0 | 42 | 0,1002 | 0,0385 | non-defined |
| Rv0791c | Rv0791c | 108L>S | 1 | 25 | 0 | 42 | 0,1002 | 0,0385 | non-defined |
| Rv0806c | cpsY | 312P>L | 1 | 25 | 0 | 42 | 0,1002 | 0,0385 | non-defined |
| Rv0825c | Rv0825c | 111L>R | 1 | 25 | 0 | 42 | 0,1002 | 0,0385 | non-defined |

| Rv0830 | Rv0830 | 131V>A | 1 | 25 | 0 | 42 | 0,1002 | 0,0385 | non-defined |
| --- | --- | --- | --- | --- | --- | --- | --- | --- | --- |
| Rv0833 | PE_PGRS13 | 514G>A | 1 | 25 | 0 | 42 | 0,1002 | 0,0385 | non-defined |
| Rv0834c | PE_PGRS14 | 804N>G | 1 | 25 | 0 | 42 | 0,1002 | 0,0385 | non-defined |
| Rv0893c | Rv0893c | 63H>Y | 1 | 25 | 0 | 42 | 0,1002 | 0,0385 | non-defined |
| Rv0906 | Rv0906 | 223V>M | 1 | 25 | 0 | 42 | 0,1002 | 0,0385 | non-defined |
| Rv0908 | ctpE | 653A>E | 1 | 25 | 0 | 42 | 0,1002 | 0,0385 | non-defined |
| Rv0923c | Rv0923c | 342T>I | 1 | 25 | 0 | 42 | 0,1002 | 0,0385 | non-defined |
| Rv0926c | Rv0926c | 179M>T | 1 | 25 | 0 | 42 | 0,1002 | 0,0385 | non-defined |
| Rv0929 | pstC2 | 162W>G | 1 | 25 | 0 | 42 | 0,1002 | 0,0385 | non-defined |
| Rv0938 | Rv0938 | 398D>H | 1 | 25 | 0 | 42 | 0,1002 | 0,0385 | non-defined |
| Rv0938 | Rv0938 | 714A>P | 1 | 25 | 0 | 42 | 0,1002 | 0,0385 | non-defined |
| Rv0939 | Rv0939 | 420I>M | 1 | 25 | 0 | 42 | 0,1002 | 0,0385 | non-defined |
| Rv0939 | Rv0939 | 446D>A | 1 | 25 | 0 | 42 | 0,1002 | 0,0385 | non-defined |
| Rv0940c | Rv0940c | 266D>G | 1 | 25 | 0 | 42 | 0,1002 | 0,0385 | non-defined |
| Rv0987 | Rv0987 | 142T>A | 1 | 25 | 0 | 42 | 0,1002 | 0,0385 | non-defined |
| Rv0987 | Rv0987 | 83V>F | 1 | 25 | 0 | 42 | 0,1002 | 0,0385 | non-defined |

| Rv0989c | grcC2 | 169Y>C | 1 | 25 | 0 | 42 | 0,1002 | 0,0385 | non-defined |
| --- | --- | --- | --- | --- | --- | --- | --- | --- | --- |
| Rv1005c | pabB | 15R>H | 1 | 25 | 0 | 42 | 0,1002 | 0,0385 | non-defined |
| Rv1014c | pth | 53G>S | 1 | 25 | 0 | 42 | 0,1002 | 0,0385 | non-defined |
| Rv1020 | mfd | 563A>P | 1 | 25 | 0 | 42 | 0,1002 | 0,0385 | non-defined |
| Rv1028c | kdpD | 75L>P | 1 | 25 | 0 | 42 | 0,1002 | 0,0385 | non-defined |
| Rv1029 | kdpA | 204H>Y | 1 | 25 | 0 | 42 | 0,1002 | 0,0385 | non-defined |
| Rv1050 | Rv1050 | 85R>H | 1 | 25 | 0 | 42 | 0,1002 | 0,0385 | non-defined |
| Rv1056 | Rv1056 | 165I>M | 1 | 25 | 0 | 42 | 0,1002 | 0,0385 | non-defined |
| Rv1057 | Rv1057 | 240I>T | 1 | 25 | 0 | 42 | 0,1002 | 0,0385 | non-defined |
| Rv1061 | Rv1061 | 269P>L | 1 | 25 | 0 | 42 | 0,1002 | 0,0385 | non-defined |
| Rv1067c | PE_PGRS19 | 496P>S | 1 | 25 | 0 | 42 | 0,1002 | 0,0385 | non-defined |
| Rv1067c | PE_PGRS19 | 85S>G | 1 | 25 | 0 | 42 | 0,1002 | 0,0385 | non-defined |
| Rv1069c | Rv1069c | 116P>L | 1 | 25 | 0 | 42 | 0,1002 | 0,0385 | non-defined |
| Rv1093 | glyA | 191L>P | 1 | 25 | 0 | 42 | 0,1002 | 0,0385 | non-defined |
| Rv1104 | Rv1104 | 96P>L | 1 | 25 | 0 | 42 | 0,1002 | 0,0385 | non-defined |
| Rv1111c | Rv1111c | 211R>G | 1 | 25 | 0 | 42 | 0,1002 | 0,0385 | non-defined |

| Rv1121 | zwf1 | 317A>S | 1 | 25 | 0 | 42 | 0,1002 | 0,0385 | non-defined |
| --- | --- | --- | --- | --- | --- | --- | --- | --- | --- |
| Rv1126c | Rv1126c | 11R>Q | 1 | 25 | 0 | 42 | 0,1002 | 0,0385 | non-defined |
| Rv1145 | mmpL13a | 265S>G | 1 | 25 | 0 | 42 | 0,1002 | 0,0385 | non-defined |
| Rv1148c | Rv1148c | 273D>A | 1 | 25 | 0 | 42 | 0,1002 | 0,0385 | non-defined |
| Rv1148c | Rv1148c | 299A>G | 1 | 25 | 0 | 42 | 0,1002 | 0,0385 | non-defined |
| Rv1175c | fadH | 509G>D | 1 | 25 | 0 | 42 | 0,1002 | 0,0385 | non-defined |
| Rv1179c | Rv1179c | 871L>P | 1 | 25 | 0 | 42 | 0,1002 | 0,0385 | non-defined |
| Rv1196 | PPE18 | 235R>L | 1 | 25 | 0 | 42 | 0,1002 | 0,0385 | non-defined |
| Rv1196 | PPE18 | 238I>L | 1 | 25 | 0 | 42 | 0,1002 | 0,0385 | non-defined |
| Rv1196 | PPE18 | 249M>V | 1 | 25 | 0 | 42 | 0,1002 | 0,0385 | non-defined |
| Rv1210 | tagA | 177G>R | 1 | 25 | 0 | 42 | 0,1002 | 0,0385 | non-defined |
| Rv1212c | Rv1212c | 209T>N | 1 | 25 | 0 | 42 | 0,1002 | 0,0385 | non-defined |
| Rv1222 | Rv1222 | 23A>T | 1 | 25 | 0 | 42 | 0,1002 | 0,0385 | non-defined |
| Rv1234 | Rv1234 | 89G>C | 1 | 25 | 0 | 42 | 0,1002 | 0,0385 | non-defined |
| Rv1238 | sugC | 209Y>C | 1 | 25 | 0 | 42 | 0,1002 | 0,0385 | non-defined |
| Rv1245c | Rv1245c | 136H>Y | 1 | 25 | 0 | 42 | 0,1002 | 0,0385 | non-defined |

| Rv1257c | Rv1257c | 424L>W | 1 | 25 | 0 | 42 | 0,1002 | 0,0385 | non-defined |
| --- | --- | --- | --- | --- | --- | --- | --- | --- | --- |
| Rv1269c | Rv1269c | 39Y>S | 1 | 25 | 0 | 42 | 0,1002 | 0,0385 | non-defined |
| Rv1270c | lprA | 123Q>* | 1 | 25 | 0 | 42 | 0,1002 | 0,0385 | non-defined |
| Rv1290c | Rv1290c | 5S>P | 1 | 25 | 0 | 42 | 0,1002 | 0,0385 | non-defined |
| Rv1307 | atpH | 25L>V | 1 | 25 | 0 | 42 | 0,1002 | 0,0385 | non-defined |
| Rv1311 | atpC | 77V>I | 1 | 25 | 0 | 42 | 0,1002 | 0,0385 | non-defined |
| Rv1317c | alkA | 60E>D | 1 | 25 | 0 | 42 | 0,1002 | 0,0385 | non-defined |
| Rv1322 | Rv1322 | 18W>R | 1 | 25 | 0 | 42 | 0,1002 | 0,0385 | non-defined |
| Rv1323 | fadA4 | 336V>I | 1 | 25 | 0 | 42 | 0,1002 | 0,0385 | non-defined |
| Rv1332 | Rv1332 | 205V>I | 1 | 25 | 0 | 42 | 0,1002 | 0,0385 | non-defined |
| Rv1345 | fadD33 | 21V>L | 1 | 25 | 0 | 42 | 0,1002 | 0,0385 | non-defined |
| Rv1345 | fadD33 | 286D>H | 1 | 25 | 0 | 42 | 0,1002 | 0,0385 | non-defined |
| Rv1348 | Rv1348 | 830L>V | 1 | 25 | 0 | 42 | 0,1002 | 0,0385 | non-defined |
| Rv1361c | PPE19 | 153F>Y | 1 | 25 | 0 | 42 | 0,1002 | 0,0385 | non-defined |
| Rv1365c | rsfA | 55T>A | 1 | 25 | 0 | 42 | 0,1002 | 0,0385 | non-defined |
| Rv1373 | Rv1373 | 177Q>P | 1 | 25 | 0 | 42 | 0,1002 | 0,0385 | non-defined |

| Rv1384 | carB | 549Y>H | 1 | 25 | 0 | 42 | 0,1002 | 0,0385 | non-defined |
| --- | --- | --- | --- | --- | --- | --- | --- | --- | --- |
| Rv1423 | whiA | 60I>V | 1 | 25 | 0 | 42 | 0,1002 | 0,0385 | non-defined |
| Rv1429 | Rv1429 | 91P>L | 1 | 25 | 0 | 42 | 0,1002 | 0,0385 | non-defined |
| Rv1441c | PE_PGRS26 | 229G>D | 1 | 25 | 0 | 42 | 0,1002 | 0,0385 | non-defined |
| Rv1450c | PE_PGRS27 | 631G>S | 1 | 25 | 0 | 42 | 0,1002 | 0,0385 | non-defined |
| Rv1452c | PE_PGRS28 | 412R>G | 1 | 25 | 0 | 42 | 0,1002 | 0,0385 | non-defined |
| Rv1452c | PE_PGRS28 | 416G>A | 1 | 25 | 0 | 42 | 0,1002 | 0,0385 | non-defined |
| Rv1452c | PE_PGRS28 | 417P>N | 1 | 25 | 0 | 42 | 0,1002 | 0,0385 | non-defined |
| Rv1455 | Rv1455 | 105R>Q | 1 | 25 | 0 | 42 | 0,1002 | 0,0385 | non-defined |
| Rv1459c | Rv1459c | 269V>I | 1 | 25 | 0 | 42 | 0,1002 | 0,0385 | non-defined |
| Rv1460 | Rv1460 | 257R>S | 1 | 25 | 0 | 42 | 0,1002 | 0,0385 | non-defined |
| Rv1467c | fadE15 | 117M>I | 1 | 25 | 0 | 42 | 0,1002 | 0,0385 | non-defined |
| Rv1467c | fadE15 | 93W>C | 1 | 25 | 0 | 42 | 0,1002 | 0,0385 | non-defined |
| Rv1480 | Rv1480 | 75D>A | 1 | 25 | 0 | 42 | 0,1002 | 0,0385 | non-defined |
| Rv1484 | inhA | 21I>T | 1 | 25 | 0 | 42 | 0,1002 | 0,0385 | non-defined |
| Rv1498c | Rv1498c | 117C>S | 1 | 25 | 0 | 42 | 0,1002 | 0,0385 | non-defined |

| Rv1498c | Rv1498c | 166R>S | 1 | 25 | 0 | 42 | 0,1002 | 0,0385 | non-defined |
| --- | --- | --- | --- | --- | --- | --- | --- | --- | --- |
| Rv1508c | Rv1508c | 374G>D | 1 | 25 | 0 | 42 | 0,1002 | 0,0385 | non-defined |
| Rv1521 | fadD25 | 537I>V | 1 | 25 | 0 | 42 | 0,1002 | 0,0385 | non-defined |
| Rv1525 | wbbL2 | 91K>R | 1 | 25 | 0 | 42 | 0,1002 | 0,0385 | non-defined |
| Rv1527c | pks5 | 676E>K | 1 | 25 | 0 | 42 | 0,1002 | 0,0385 | non-defined |
| Rv1550 | fadD11.1 | 104R>Q | 1 | 25 | 0 | 42 | 0,1002 | 0,0385 | non-defined |
| Rv1556 | Rv1556 | 185V>A | 1 | 25 | 0 | 42 | 0,1002 | 0,0385 | non-defined |
| Rv1571 | Rv1571 | 170*>G | 1 | 25 | 0 | 42 | 0,1002 | 0,0385 | non-defined |
| Rv1588c | Rv1588c | 19E>D | 1 | 25 | 0 | 42 | 0,1002 | 0,0385 | non-defined |
| Rv1595 | nadB | 289D>E | 1 | 25 | 0 | 42 | 0,1002 | 0,0385 | non-defined |
| Rv1630 | rpsA | 381A>V | 1 | 25 | 0 | 42 | 0,1002 | 0,0385 | non-defined |
| Rv1650 | pheT | 781T>N | 1 | 25 | 0 | 42 | 0,1002 | 0,0385 | non-defined |
| Rv1654 | argB | 246S>L | 1 | 25 | 0 | 42 | 0,1002 | 0,0385 | non-defined |
| Rv1654 | argB | 43D>A | 1 | 25 | 0 | 42 | 0,1002 | 0,0385 | non-defined |
| Rv1656 | argF | 303L>M | 1 | 25 | 0 | 42 | 0,1002 | 0,0385 | non-defined |
| Rv1659 | argH | 90A>S | 1 | 25 | 0 | 42 | 0,1002 | 0,0385 | non-defined |

| Rv1662 | pks8 | 1390V>M | 1 | 25 | 0 | 42 | 0,1002 | 0,0385 | non-defined |
| --- | --- | --- | --- | --- | --- | --- | --- | --- | --- |
| Rv1682 | Rv1682 | 303R>Q | 1 | 25 | 0 | 42 | 0,1002 | 0,0385 | non-defined |
| Rv1688 | mpg | 22I>T | 1 | 25 | 0 | 42 | 0,1002 | 0,0385 | non-defined |
| Rv1705c | PPE22 | 364T>M | 1 | 25 | 0 | 42 | 0,1002 | 0,0385 | non-defined |
| Rv1717 | Rv1717 | 26Q>P | 1 | 25 | 0 | 42 | 0,1002 | 0,0385 | non-defined |
| Rv1724c | Rv1724c | 117A>V | 1 | 25 | 0 | 42 | 0,1002 | 0,0385 | non-defined |
| Rv1726 | Rv1726 | 252P>T | 1 | 25 | 0 | 42 | 0,1002 | 0,0385 | non-defined |
| Rv1730c | Rv1730c | 241T>M | 1 | 25 | 0 | 42 | 0,1002 | 0,0385 | non-defined |
| Rv1737c | narK2 | 156R>W | 1 | 25 | 0 | 42 | 0,1002 | 0,0385 | non-defined |
| Rv1743 | pknE | 9E>G | 1 | 25 | 0 | 42 | 0,1002 | 0,0385 | non-defined |
| Rv1744c | Rv1744c | 9S>N | 1 | 25 | 0 | 42 | 0,1002 | 0,0385 | non-defined |
| Rv1753c | PPE24 | 370N>S | 1 | 25 | 0 | 42 | 0,1002 | 0,0385 | non-defined |
| Rv1764 | Rv1764 | 181G>S | 1 | 25 | 0 | 42 | 0,1002 | 0,0385 | non-defined |
| Rv1774 | Rv1774 | 347L>V | 1 | 25 | 0 | 42 | 0,1002 | 0,0385 | non-defined |
| Rv1779c | Rv1779c | 167L>V | 1 | 25 | 0 | 42 | 0,1002 | 0,0385 | non-defined |
| Rv1779c | Rv1779c | 99A>T | 1 | 25 | 0 | 42 | 0,1002 | 0,0385 | non-defined |

| Rv1818c | PE_PGRS33 | 188A>D | 1 | 25 | 0 | 42 | 0,1002 | 0,0385 | non-defined |
| --- | --- | --- | --- | --- | --- | --- | --- | --- | --- |
| Rv1818c | PE_PGRS33 | 246A>S | 1 | 25 | 0 | 42 | 0,1002 | 0,0385 | non-defined |
| Rv1842c | Rv1842c | 389G>S | 1 | 25 | 0 | 42 | 0,1002 | 0,0385 | non-defined |
| Rv1855c | Rv1855c | 84G>S | 1 | 25 | 0 | 42 | 0,1002 | 0,0385 | non-defined |
| Rv1867 | Rv1867 | 89G>S | 1 | 25 | 0 | 42 | 0,1002 | 0,0385 | non-defined |
| Rv1902c | nanT | 92F>V | 1 | 25 | 0 | 42 | 0,1002 | 0,0385 | non-defined |
| Rv1918c | PPE35 | 403N>S | 1 | 25 | 0 | 42 | 0,1002 | 0,0385 | non-defined |
| Rv1925 | fadD31 | 318S>T | 1 | 25 | 0 | 42 | 0,1002 | 0,0385 | non-defined |
| Rv1928c | Rv1928c | 13R>S | 1 | 25 | 0 | 42 | 0,1002 | 0,0385 | non-defined |
| Rv1944c | Rv1944c | 4D>E | 1 | 25 | 0 | 42 | 0,1002 | 0,0385 | non-defined |
| Rv1944c | Rv1944c | 5T>A | 1 | 25 | 0 | 42 | 0,1002 | 0,0385 | non-defined |
| Rv1955 | Rv1954c | 29T>I | 1 | 25 | 0 | 42 | 0,1002 | 0,0385 | non-defined |
| Rv1961 | Rv1961 | 17S>G | 1 | 25 | 0 | 42 | 0,1002 | 0,0385 | non-defined |
| Rv1996 | Rv1996 | 116A>T | 1 | 25 | 0 | 42 | 0,1002 | 0,0385 | non-defined |
| Rv1998c | Rv1998c | 217A>T | 1 | 25 | 0 | 42 | 0,1002 | 0,0385 | non-defined |
| Rv2020c | Rv2020c | 91K>E | 1 | 25 | 0 | 42 | 0,1002 | 0,0385 | non-defined |

| Rv2030c | Rv2030c | 338P>L | 1 | 25 | 0 | 42 | 0,1002 | 0,0385 | non-defined |
| --- | --- | --- | --- | --- | --- | --- | --- | --- | --- |
| Rv2038c | Rv2038c | 268G>D | 1 | 25 | 0 | 42 | 0,1002 | 0,0385 | non-defined |
| Rv2043c | pncA | 12D>G | 1 | 25 | 0 | 42 | 0,1002 | 0,0385 | non-defined |
| Rv2043c | pncA | 131V>G | 1 | 25 | 0 | 42 | 0,1002 | 0,0385 | non-defined |
| Rv2043c | pncA | 139V>A | 1 | 25 | 0 | 42 | 0,1002 | 0,0385 | non-defined |
| Rv2043c | pncA | 182L>W | 1 | 25 | 0 | 42 | 0,1002 | 0,0385 | non-defined |
| Rv2043c | pncA | 34Y>D | 1 | 25 | 0 | 42 | 0,1002 | 0,0385 | non-defined |
| Rv2043c | pncA | 51H>R | 1 | 25 | 0 | 42 | 0,1002 | 0,0385 | non-defined |
| Rv2043c | pncA | 57H>R | 1 | 25 | 0 | 42 | 0,1002 | 0,0385 | non-defined |
| Rv2043c | pncA | 62P>L | 1 | 25 | 0 | 42 | 0,1002 | 0,0385 | non-defined |
| Rv2043c | pncA | 64Y>D | 1 | 25 | 0 | 42 | 0,1002 | 0,0385 | non-defined |
| Rv2043c | pncA | 78G>C | 1 | 25 | 0 | 42 | 0,1002 | 0,0385 | non-defined |
| Rv2043c | pncA | 94F>L | 1 | 25 | 0 | 42 | 0,1002 | 0,0385 | non-defined |
| Rv2048c | pks12 | 1132P>L | 1 | 25 | 0 | 42 | 0,1002 | 0,0385 | non-defined |
| Rv2049c | Rv2049c | 37E>K | 1 | 25 | 0 | 42 | 0,1002 | 0,0385 | non-defined |
| Rv2062c | cobN | 396G>R | 1 | 25 | 0 | 42 | 0,1002 | 0,0385 | non-defined |

| Rv2082 | Rv2082 | 34R>G | 1 | 25 | 0 | 42 | 0,1002 | 0,0385 | non-defined |
| --- | --- | --- | --- | --- | --- | --- | --- | --- | --- |
| Rv2101 | helZ | 942A>V | 1 | 25 | 0 | 42 | 0,1002 | 0,0385 | non-defined |
| Rv2108 | PPE36 | 125W>R | 1 | 25 | 0 | 42 | 0,1002 | 0,0385 | non-defined |
| Rv2118c | Rv2118c | 53V>L | 1 | 25 | 0 | 42 | 0,1002 | 0,0385 | non-defined |
| Rv2124c | metH | 1132R>C | 1 | 25 | 0 | 42 | 0,1002 | 0,0385 | non-defined |
| Rv2127 | ansP1 | 272Y>H | 1 | 25 | 0 | 42 | 0,1002 | 0,0385 | non-defined |
| Rv2133c | Rv2133c | 256P>T | 1 | 25 | 0 | 42 | 0,1002 | 0,0385 | non-defined |
| Rv2134c | Rv2134c | 2A>G | 1 | 25 | 0 | 42 | 0,1002 | 0,0385 | non-defined |
| Rv2138 | lppL | 33N>S | 1 | 25 | 0 | 42 | 0,1002 | 0,0385 | non-defined |
| Rv2163c | pbpB | 153R>H | 1 | 25 | 0 | 42 | 0,1002 | 0,0385 | non-defined |
| Rv2178c | aroG | 44Q>R | 1 | 25 | 0 | 42 | 0,1002 | 0,0385 | non-defined |
| Rv2183c | Rv2183c | 66L>R | 1 | 25 | 0 | 42 | 0,1002 | 0,0385 | non-defined |
| Rv2188c | Rv2188c | 60R>W | 1 | 25 | 0 | 42 | 0,1002 | 0,0385 | non-defined |
| Rv2214c | ephD | 205H>Y | 1 | 25 | 0 | 42 | 0,1002 | 0,0385 | non-defined |
| Rv2221c | glnE | 181A>V | 1 | 25 | 0 | 42 | 0,1002 | 0,0385 | non-defined |
| Rv2241 | aceE | 828L>V | 1 | 25 | 0 | 42 | 0,1002 | 0,0385 | non-defined |

| Rv2248 | Rv2248 | 63T>I | 1 | 25 | 0 | 42 | 0,1002 | 0,0385 | non-defined |
| --- | --- | --- | --- | --- | --- | --- | --- | --- | --- |
| Rv2266 | cyp124 | 75Y>N | 1 | 25 | 0 | 42 | 0,1002 | 0,0385 | non-defined |
| Rv2267c | Rv2267c | 297T>I | 1 | 25 | 0 | 42 | 0,1002 | 0,0385 | non-defined |
| Rv2281 | pitB | 143D>N | 1 | 25 | 0 | 42 | 0,1002 | 0,0385 | non-defined |
| Rv2295 | Rv2295 | 143A>S | 1 | 25 | 0 | 42 | 0,1002 | 0,0385 | non-defined |
| Rv2297 | Rv2297 | 147K>E | 1 | 25 | 0 | 42 | 0,1002 | 0,0385 | non-defined |
| Rv2303c | Rv2303c | 216Q>P | 1 | 25 | 0 | 42 | 0,1002 | 0,0385 | non-defined |
| Rv2318 | uspC | 434A>T | 1 | 25 | 0 | 42 | 0,1002 | 0,0385 | non-defined |
| Rv2329c | narK1 | 391M>V | 1 | 25 | 0 | 42 | 0,1002 | 0,0385 | non-defined |
| Rv2332 | mez | 46D>E | 1 | 25 | 0 | 42 | 0,1002 | 0,0385 | non-defined |
| Rv2334 | cysK1 | 32A>T | 1 | 25 | 0 | 42 | 0,1002 | 0,0385 | non-defined |
| Rv2344c | dgt | 174G>A | 1 | 25 | 0 | 42 | 0,1002 | 0,0385 | non-defined |
| Rv2362c | recO | 89R>C | 1 | 25 | 0 | 42 | 0,1002 | 0,0385 | non-defined |
| Rv2368c | phoH1 | 63L>F | 1 | 25 | 0 | 42 | 0,1002 | 0,0385 | non-defined |
| Rv2376c | cfp2 | 142Q>R | 1 | 25 | 0 | 42 | 0,1002 | 0,0385 | non-defined |
| Rv2381c | mbtD | 729T>A | 1 | 25 | 0 | 42 | 0,1002 | 0,0385 | non-defined |

| Rv2384 | mbtA | 292E>G | 1 | 25 | 0 | 42 | 0,1002 | 0,0385 | non-defined |
| --- | --- | --- | --- | --- | --- | --- | --- | --- | --- |
| Rv2393 | Rv2393 | 31R>P | 1 | 25 | 0 | 42 | 0,1002 | 0,0385 | non-defined |
| Rv2406c | Rv2406c | 41V>L | 1 | 25 | 0 | 42 | 0,1002 | 0,0385 | non-defined |
| Rv2409c | Rv2409c | 199Y>H | 1 | 25 | 0 | 42 | 0,1002 | 0,0385 | non-defined |
| Rv2413c | Rv2413c | 268K>N | 1 | 25 | 0 | 42 | 0,1002 | 0,0385 | non-defined |
| Rv2423 | Rv2423 | 135V>M | 1 | 25 | 0 | 42 | 0,1002 | 0,0385 | non-defined |
| Rv2425c | Rv2425c | 219A>T | 1 | 25 | 0 | 42 | 0,1002 | 0,0385 | non-defined |
| Rv2427c | proA | 75M>T | 1 | 25 | 0 | 42 | 0,1002 | 0,0385 | non-defined |
| Rv2428 | ahpC | 130V>M | 1 | 25 | 0 | 42 | 0,1002 | 0,0385 | non-defined |
| Rv2428 | ahpC | 73D>H | 1 | 25 | 0 | 42 | 0,1002 | 0,0385 | non-defined |
| Rv2447c | folC | 441D>G | 1 | 25 | 0 | 42 | 0,1002 | 0,0385 | non-defined |
| Rv2455c | Rv2455c | 406V>A | 1 | 25 | 0 | 42 | 0,1002 | 0,0385 | non-defined |
| Rv2457c | clpX | 136K>T | 1 | 25 | 0 | 42 | 0,1002 | 0,0385 | non-defined |
| Rv2471 | aglA | 123M>T | 1 | 25 | 0 | 42 | 0,1002 | 0,0385 | non-defined |
| Rv2478c | Rv2478c | 121P>A | 1 | 25 | 0 | 42 | 0,1002 | 0,0385 | non-defined |
| Rv2479c | Rv2479c | 233G>S | 1 | 25 | 0 | 42 | 0,1002 | 0,0385 | non-defined |

| Rv2487c | PE_PGRS42 | 594A>V | 1 | 25 | 0 | 42 | 0,1002 | 0,0385 | non-defined |
| --- | --- | --- | --- | --- | --- | --- | --- | --- | --- |
| Rv2490c | PE_PGRS43 | 999A>E | 1 | 25 | 0 | 42 | 0,1002 | 0,0385 | non-defined |
| Rv2508c | Rv2508c | 278A>T | 1 | 25 | 0 | 42 | 0,1002 | 0,0385 | non-defined |
| Rv2543 | lppA | 47G>D | 1 | 25 | 0 | 42 | 0,1002 | 0,0385 | non-defined |
| Rv2543 | lppA | 93D>T | 1 | 25 | 0 | 42 | 0,1002 | 0,0385 | non-defined |
| Rv2543 | lppA | 94D>E | 1 | 25 | 0 | 42 | 0,1002 | 0,0385 | non-defined |
| Rv2544 | lppB | 151V>A | 1 | 25 | 0 | 42 | 0,1002 | 0,0385 | non-defined |
| Rv2544 | lppB | 176N>R | 1 | 25 | 0 | 42 | 0,1002 | 0,0385 | non-defined |
| Rv2552c | aroE | 68G>D | 1 | 25 | 0 | 42 | 0,1002 | 0,0385 | non-defined |
| Rv2586c | secF | 312V>L | 1 | 25 | 0 | 42 | 0,1002 | 0,0385 | non-defined |
| Rv2634c | PE_PGRS46 | 2S>A | 1 | 25 | 0 | 42 | 0,1002 | 0,0385 | non-defined |
| Rv2650c | Rv2650c | 89D>A | 1 | 25 | 0 | 42 | 0,1002 | 0,0385 | non-defined |
| Rv2676c | Rv2676c | 222P>S | 1 | 25 | 0 | 42 | 0,1002 | 0,0385 | non-defined |
| Rv2712c | Rv2712c | 155Q>K | 1 | 25 | 0 | 42 | 0,1002 | 0,0385 | non-defined |
| Rv2712c | Rv2712c | 92Y>C | 1 | 25 | 0 | 42 | 0,1002 | 0,0385 | non-defined |
| Rv2713 | sthA | 106N>I | 1 | 25 | 0 | 42 | 0,1002 | 0,0385 | non-defined |

| Rv2724c | fadE20 | 66Y>C | 1 | 25 | 0 | 42 | 0,1002 | 0,0385 | non-defined |
| --- | --- | --- | --- | --- | --- | --- | --- | --- | --- |
| Rv2725c | hflX | 333P>A | 1 | 25 | 0 | 42 | 0,1002 | 0,0385 | non-defined |
| Rv2730 | Rv2730 | 128V>L | 1 | 25 | 0 | 42 | 0,1002 | 0,0385 | non-defined |
| Rv2737c | recA | 473D>G | 1 | 25 | 0 | 42 | 0,1002 | 0,0385 | non-defined |
| Rv2738c | Rv2738c | 9E>D | 1 | 25 | 0 | 42 | 0,1002 | 0,0385 | non-defined |
| Rv2751 | Rv2751 | 74L>P | 1 | 25 | 0 | 42 | 0,1002 | 0,0385 | non-defined |
| Rv2784c | lppU | 95I>V | 1 | 25 | 0 | 42 | 0,1002 | 0,0385 | non-defined |
| Rv2793c | truB | 296H>R | 1 | 25 | 0 | 42 | 0,1002 | 0,0385 | non-defined |
| Rv2805 | Rv2804c | 17V>V | 1 | 25 | 0 | 42 | 0,1002 | 0,0385 | non-defined |
| Rv2819c | Rv2819c | 273T>M | 1 | 25 | 0 | 42 | 0,1002 | 0,0385 | non-defined |
| Rv2850c | Rv2850c | 396S>L | 1 | 25 | 0 | 42 | 0,1002 | 0,0385 | non-defined |
| Rv2856 | nicT | 357Y>C | 1 | 25 | 0 | 42 | 0,1002 | 0,0385 | non-defined |
| Rv2885c | Rv2885c | 106G>A | 1 | 25 | 0 | 42 | 0,1002 | 0,0385 | non-defined |
| Rv2885c | Rv2885c | 107A>T | 1 | 25 | 0 | 42 | 0,1002 | 0,0385 | non-defined |
| Rv2888c | amiC | 469P>R | 1 | 25 | 0 | 42 | 0,1002 | 0,0385 | non-defined |
| Rv2890c | rpsB | 157V>A | 1 | 25 | 0 | 42 | 0,1002 | 0,0385 | non-defined |

| Rv2897c | Rv2897c | 218G>V | 1 | 25 | 0 | 42 | 0,1002 | 0,0385 | non-defined |
| --- | --- | --- | --- | --- | --- | --- | --- | --- | --- |
| Rv2900c | fdhF | 574I>V | 1 | 25 | 0 | 42 | 0,1002 | 0,0385 | non-defined |
| Rv2917 | Rv2917 | 376S>G | 1 | 25 | 0 | 42 | 0,1002 | 0,0385 | non-defined |
| Rv2931 | ppsA | 1323G>S | 1 | 25 | 0 | 42 | 0,1002 | 0,0385 | non-defined |
| Rv2932 | ppsB | 580A>P | 1 | 25 | 0 | 42 | 0,1002 | 0,0385 | non-defined |
| Rv2933 | ppsC | 1661D>N | 1 | 25 | 0 | 42 | 0,1002 | 0,0385 | non-defined |
| Rv2940c | mas | 22N>K | 1 | 25 | 0 | 42 | 0,1002 | 0,0385 | non-defined |
| Rv2940c | mas | 448R>W | 1 | 25 | 0 | 42 | 0,1002 | 0,0385 | non-defined |
| Rv2946c | pks1 | 1381N>T | 1 | 25 | 0 | 42 | 0,1002 | 0,0385 | non-defined |
| Rv2948c | fadD22 | 598P>H | 1 | 25 | 0 | 42 | 0,1002 | 0,0385 | non-defined |
| Rv2951c | Rv2951c | 194A>S | 1 | 25 | 0 | 42 | 0,1002 | 0,0385 | non-defined |
| Rv2958c | Rv2958c | 327R>C | 1 | 25 | 0 | 42 | 0,1002 | 0,0385 | non-defined |
| Rv2994 | Rv2994 | 214W>* | 1 | 25 | 0 | 42 | 0,1002 | 0,0385 | non-defined |
| Rv2997 | Rv2997 | 160T>A | 1 | 25 | 0 | 42 | 0,1002 | 0,0385 | non-defined |
| Rv3003c | ilvB1 | 610F>L | 1 | 25 | 0 | 42 | 0,1002 | 0,0385 | non-defined |
| Rv3009c | gatB | 456V>L | 1 | 25 | 0 | 42 | 0,1002 | 0,0385 | non-defined |

| Rv3015c | Rv3015c | 15G>A | 1 | 25 | 0 | 42 | 0,1002 | 0,0385 | non-defined |
| --- | --- | --- | --- | --- | --- | --- | --- | --- | --- |
| Rv3020c | esxS | 49A>V | 1 | 25 | 0 | 42 | 0,1002 | 0,0385 | non-defined |
| Rv3034c | Rv3034c | 30T>N | 1 | 25 | 0 | 42 | 0,1002 | 0,0385 | non-defined |
| Rv3035 | Rv3035 | 128D>E | 1 | 25 | 0 | 42 | 0,1002 | 0,0385 | non-defined |
| Rv3036c | TB22.2 | 2R>C | 1 | 25 | 0 | 42 | 0,1002 | 0,0385 | non-defined |
| Rv3053c | nrdH | 5V>I | 1 | 25 | 0 | 42 | 0,1002 | 0,0385 | non-defined |
| Rv3061c | fadE22 | 139A>V | 1 | 25 | 0 | 42 | 0,1002 | 0,0385 | non-defined |
| Rv3073c | Rv3073c | 24V>I | 1 | 25 | 0 | 42 | 0,1002 | 0,0385 | non-defined |
| Rv3083 | Rv3083 | 330L>P | 1 | 25 | 0 | 42 | 0,1002 | 0,0385 | non-defined |
| Rv3083 | Rv3083 | 451M>V | 1 | 25 | 0 | 42 | 0,1002 | 0,0385 | non-defined |
| Rv3086 | adhD | 65G>E | 1 | 25 | 0 | 42 | 0,1002 | 0,0385 | non-defined |
| Rv3089 | fadD13 | 367E>D | 1 | 25 | 0 | 42 | 0,1002 | 0,0385 | non-defined |
| Rv3090 | Rv3090 | 150R>H | 1 | 25 | 0 | 42 | 0,1002 | 0,0385 | non-defined |
| Rv3139 | fadE24 | 379Q>R | 1 | 25 | 0 | 42 | 0,1002 | 0,0385 | non-defined |
| Rv3147 | nuoC | 199M>L | 1 | 25 | 0 | 42 | 0,1002 | 0,0385 | non-defined |
| Rv3170 | aofH | 295S>N | 1 | 25 | 0 | 42 | 0,1002 | 0,0385 | non-defined |

| Rv3176c | mesT | 78R>H | 1 | 25 | 0 | 42 | 0,1002 | 0,0385 | non-defined |
| --- | --- | --- | --- | --- | --- | --- | --- | --- | --- |
| Rv3190c | Rv3190c | 377P>T | 1 | 25 | 0 | 42 | 0,1002 | 0,0385 | non-defined |
| Rv3197 | Rv3197 | 193V>M | 1 | 25 | 0 | 42 | 0,1002 | 0,0385 | non-defined |
| Rv3199c | nudC | 289S>P | 1 | 25 | 0 | 42 | 0,1002 | 0,0385 | non-defined |
| Rv3205c | Rv3205c | 280G>D | 1 | 25 | 0 | 42 | 0,1002 | 0,0385 | non-defined |
| Rv3220c | Rv3220c | 340S>L | 1 | 25 | 0 | 42 | 0,1002 | 0,0385 | non-defined |
| Rv3239c | Rv3239c | 548P>S | 1 | 25 | 0 | 42 | 0,1002 | 0,0385 | non-defined |
| Rv3239c | Rv3239c | 904V>I | 1 | 25 | 0 | 42 | 0,1002 | 0,0385 | non-defined |
| Rv3242c | Rv3242c | 40P>L | 1 | 25 | 0 | 42 | 0,1002 | 0,0385 | non-defined |
| Rv3245c | mtrB | 174G>A | 1 | 25 | 0 | 42 | 0,1002 | 0,0385 | non-defined |
| Rv3249c | Rv3249c | 154T>A | 1 | 25 | 0 | 42 | 0,1002 | 0,0385 | non-defined |
| Rv3263 | Rv3263 | 402T>A | 1 | 25 | 0 | 42 | 0,1002 | 0,0385 | non-defined |
| Rv3281 | Rv3281 | 35E>P | 1 | 25 | 0 | 42 | 0,1002 | 0,0385 | non-defined |
| Rv3309c | upp | 96G>S | 1 | 25 | 0 | 42 | 0,1002 | 0,0385 | non-defined |
| Rv3310 | Rv3310 | 168N>S | 1 | 25 | 0 | 42 | 0,1002 | 0,0385 | non-defined |
| Rv3322c | Rv3322c | 53E>G | 1 | 25 | 0 | 42 | 0,1002 | 0,0385 | non-defined |

| Rv3328c | sigJ | 125D>G | 1 | 25 | 0 | 42 | 0,1002 | 0,0385 | non-defined |
| --- | --- | --- | --- | --- | --- | --- | --- | --- | --- |
| Rv3328c | sigJ | 211A>T | 1 | 25 | 0 | 42 | 0,1002 | 0,0385 | non-defined |
| Rv3341 | metX | 87G>S | 1 | 25 | 0 | 42 | 0,1002 | 0,0385 | non-defined |
| Rv3344c | PE_PGRS49 | 405P>A | 1 | 25 | 0 | 42 | 0,1002 | 0,0385 | non-defined |
| Rv3345c | PE_PGRS50 | 1112G>A | 1 | 25 | 0 | 42 | 0,1002 | 0,0385 | non-defined |
| Rv3345c | PE_PGRS50 | 1367H>N | 1 | 25 | 0 | 42 | 0,1002 | 0,0385 | non-defined |
| Rv3346c | Rv3346c | 7L>F | 1 | 25 | 0 | 42 | 0,1002 | 0,0385 | non-defined |
| Rv3347c | PPE55 | 122M>V | 1 | 25 | 0 | 42 | 0,1002 | 0,0385 | non-defined |
| Rv3347c | PPE55 | 215I>V | 1 | 25 | 0 | 42 | 0,1002 | 0,0385 | non-defined |
| Rv3347c | PPE55 | 276L>F | 1 | 25 | 0 | 42 | 0,1002 | 0,0385 | non-defined |
| Rv3347c | PPE55 | 3054P>S | 1 | 25 | 0 | 42 | 0,1002 | 0,0385 | non-defined |
| Rv3350c | PPE56 | 2083G>R | 1 | 25 | 0 | 42 | 0,1002 | 0,0385 | non-defined |
| Rv3373 | echA18 | 24M>I | 1 | 25 | 0 | 42 | 0,1002 | 0,0385 | non-defined |
| Rv3377c | Rv3377c | 306K>E | 1 | 25 | 0 | 42 | 0,1002 | 0,0385 | non-defined |
| Rv3378c | Rv3378c | 91I>S | 1 | 25 | 0 | 42 | 0,1002 | 0,0385 | non-defined |
| Rv3379c | dxs2 | 232V>L | 1 | 25 | 0 | 42 | 0,1002 | 0,0385 | non-defined |

| Rv3379c | dxs2 | 278T>I | 1 | 25 | 0 | 42 | 0,1002 | 0,0385 | non-defined |
| --- | --- | --- | --- | --- | --- | --- | --- | --- | --- |
| Rv3380c | Rv3380c | 139A>V | 1 | 25 | 0 | 42 | 0,1002 | 0,0385 | non-defined |
| Rv3390 | lpqD | 221V>G | 1 | 25 | 0 | 42 | 0,1002 | 0,0385 | non-defined |
| Rv3401 | Rv3401 | 786R>W | 1 | 25 | 0 | 42 | 0,1002 | 0,0385 | non-defined |
| Rv3419c | gcp | 146H>Q | 1 | 25 | 0 | 42 | 0,1002 | 0,0385 | non-defined |
| Rv3423c | alr | 140E>D | 1 | 25 | 0 | 42 | 0,1002 | 0,0385 | non-defined |
| Rv3423c | alr | 23L>M | 1 | 25 | 0 | 42 | 0,1002 | 0,0385 | non-defined |
| Rv3425 | PPE57 | 125G>A | 1 | 25 | 0 | 42 | 0,1002 | 0,0385 | non-defined |
| Rv3431c | Rv3431c | 236M>T | 1 | 25 | 0 | 42 | 0,1002 | 0,0385 | non-defined |
| Rv3436c | glmS | 64T>K | 1 | 25 | 0 | 42 | 0,1002 | 0,0385 | non-defined |
| Rv3446c | Rv3446c | 240G>C | 1 | 25 | 0 | 42 | 0,1002 | 0,0385 | non-defined |
| Rv3448 | Rv3448 | 346R>T | 1 | 25 | 0 | 42 | 0,1002 | 0,0385 | non-defined |
| Rv3449 | mycP4 | 356Q>P | 1 | 25 | 0 | 42 | 0,1002 | 0,0385 | non-defined |
| Rv3449 | mycP4 | 67A>V | 1 | 25 | 0 | 42 | 0,1002 | 0,0385 | non-defined |
| Rv3451 | cut3 | 37P>L | 1 | 25 | 0 | 42 | 0,1002 | 0,0385 | non-defined |
| Rv3452 | cut4 | 219A>T | 1 | 25 | 0 | 42 | 0,1002 | 0,0385 | non-defined |

| Rv3470c | ilvB2 | 264D>Y | 1 | 25 | 0 | 42 | 0,1002 | 0,0385 | non-defined |
| --- | --- | --- | --- | --- | --- | --- | --- | --- | --- |
| Rv3478 | PPE60 | 356H>N | 1 | 25 | 0 | 42 | 0,1002 | 0,0385 | non-defined |
| Rv3480c | Rv3480c | 427L>V | 1 | 25 | 0 | 42 | 0,1002 | 0,0385 | non-defined |
| Rv3481c | Rv3481c | 216V>A | 1 | 25 | 0 | 42 | 0,1002 | 0,0385 | non-defined |
| Rv3498c | mce4B | 107L>P | 1 | 25 | 0 | 42 | 0,1002 | 0,0385 | non-defined |
| Rv3506 | fadD17 | 447K>T | 1 | 25 | 0 | 42 | 0,1002 | 0,0385 | non-defined |
| Rv3507 | PE_PGRS53 | 1298G>D | 1 | 25 | 0 | 42 | 0,1002 | 0,0385 | non-defined |
| Rv3507 | PE_PGRS53 | 343A>T | 1 | 25 | 0 | 42 | 0,1002 | 0,0385 | non-defined |
| Rv3507 | PE_PGRS53 | 346M>K | 1 | 25 | 0 | 42 | 0,1002 | 0,0385 | non-defined |
| Rv3508 | PE_PGRS54 | 1000R>G | 1 | 25 | 0 | 42 | 0,1002 | 0,0385 | non-defined |
| Rv3508 | PE_PGRS54 | 1491Q>D | 1 | 25 | 0 | 42 | 0,1002 | 0,0385 | non-defined |
| Rv3508 | PE_PGRS54 | 1497A>D | 1 | 25 | 0 | 42 | 0,1002 | 0,0385 | non-defined |
| Rv3508 | PE_PGRS54 | 1696Q>D | 1 | 25 | 0 | 42 | 0,1002 | 0,0385 | non-defined |
| Rv3508 | PE_PGRS54 | 1702A>D | 1 | 25 | 0 | 42 | 0,1002 | 0,0385 | non-defined |
| Rv3508 | PE_PGRS54 | 195G>S | 1 | 25 | 0 | 42 | 0,1002 | 0,0385 | non-defined |
| Rv3508 | PE_PGRS54 | 284G>R | 1 | 25 | 0 | 42 | 0,1002 | 0,0385 | non-defined |

| Rv3512 | PE_PGRS56 | 392E>K | 1 | 25 | 0 | 42 | 0,1002 | 0,0385 | non-defined |
| --- | --- | --- | --- | --- | --- | --- | --- | --- | --- |
| Rv3517 | Rv3517 | 141A>E | 1 | 25 | 0 | 42 | 0,1002 | 0,0385 | non-defined |
| Rv3524 | Rv3524 | 165E>G | 1 | 25 | 0 | 42 | 0,1002 | 0,0385 | non-defined |
| Rv3532 | PPE61 | 141Q>* | 1 | 25 | 0 | 42 | 0,1002 | 0,0385 | non-defined |
| Rv3543c | fadE29 | 331E>G | 1 | 25 | 0 | 42 | 0,1002 | 0,0385 | non-defined |
| Rv3570c | Rv3570c | 306A>G | 1 | 25 | 0 | 42 | 0,1002 | 0,0385 | non-defined |
| Rv3573c | fadE34 | 340L>V | 1 | 25 | 0 | 42 | 0,1002 | 0,0385 | non-defined |
| Rv3593 | lpqF | 328T>M | 1 | 25 | 0 | 42 | 0,1002 | 0,0385 | non-defined |
| Rv3605c | Rv3605c | 72L>R | 1 | 25 | 0 | 42 | 0,1002 | 0,0385 | non-defined |
| Rv3610c | ftsH | 390V>I | 1 | 25 | 0 | 42 | 0,1002 | 0,0385 | non-defined |
| Rv3616c | Rv3616c | 11I>V | 1 | 25 | 0 | 42 | 0,1002 | 0,0385 | non-defined |
| Rv3630 | Rv3630 | 83L>M | 1 | 25 | 0 | 42 | 0,1002 | 0,0385 | non-defined |
| Rv3649 | Rv3649 | 518G>R | 1 | 25 | 0 | 42 | 0,1002 | 0,0385 | non-defined |
| Rv3662c | Rv3662c | 197H>D | 1 | 25 | 0 | 42 | 0,1002 | 0,0385 | non-defined |
| Rv3675 | Rv3675 | 76R>* | 1 | 25 | 0 | 42 | 0,1002 | 0,0385 | non-defined |
| Rv3685c | cyp137 | 319H>Y | 1 | 25 | 0 | 42 | 0,1002 | 0,0385 | non-defined |

| Rv3720 | Rv3720 | 129R>H | 1 | 25 | 0 | 42 | 0,1002 | 0,0385 | non-defined |
| --- | --- | --- | --- | --- | --- | --- | --- | --- | --- |
| Rv3721c | dnaZX | 366S>A | 1 | 25 | 0 | 42 | 0,1002 | 0,0385 | non-defined |
| Rv3738c | PPE66 | 102D>G | 1 | 25 | 0 | 42 | 0,1002 | 0,0385 | non-defined |
| Rv3743c | ctpJ | 274P>R | 1 | 25 | 0 | 42 | 0,1002 | 0,0385 | non-defined |
| Rv3759c | proX | 125R>Q | 1 | 25 | 0 | 42 | 0,1002 | 0,0385 | non-defined |
| Rv3763 | lpqH | 117G>R | 1 | 25 | 0 | 42 | 0,1002 | 0,0385 | non-defined |
| Rv3763 | lpqH | 158C>S | 1 | 25 | 0 | 42 | 0,1002 | 0,0385 | non-defined |
| Rv3765c | Rv3765c | 183A>G | 1 | 25 | 0 | 42 | 0,1002 | 0,0385 | non-defined |
| Rv3766 | Rv3766 | 183V>A | 1 | 25 | 0 | 42 | 0,1002 | 0,0385 | non-defined |
| Rv3772 | hisC2 | 349W>* | 1 | 25 | 0 | 42 | 0,1002 | 0,0385 | non-defined |
| Rv3779 | Rv3779 | 42G>S | 1 | 25 | 0 | 42 | 0,1002 | 0,0385 | non-defined |
| Rv3792 | Rv3792 | 103V>A | 1 | 25 | 0 | 42 | 0,1002 | 0,0385 | non-defined |
| Rv3795 | embB | 406G>A | 1 | 25 | 0 | 42 | 0,1002 | 0,0385 | non-defined |
| Rv3795 | embB | 445Q>R | 1 | 25 | 0 | 42 | 0,1002 | 0,0385 | non-defined |
| Rv3795 | embB | 489I>T | 1 | 25 | 0 | 42 | 0,1002 | 0,0385 | non-defined |
| Rv3799c | accD4 | 241Y>C | 1 | 25 | 0 | 42 | 0,1002 | 0,0385 | non-defined |

| Rv3806c | Rv3806c | 38A>V | 1 | 25 | 0 | 42 | 0,1002 | 0,0385 | non-defined |
| --- | --- | --- | --- | --- | --- | --- | --- | --- | --- |
| Rv3808c | glfT | 426D>Y | 1 | 25 | 0 | 42 | 0,1002 | 0,0385 | non-defined |
| Rv3808c | glfT | 482Q>R | 1 | 25 | 0 | 42 | 0,1002 | 0,0385 | non-defined |
| Rv3809c | glf | 131A>S | 1 | 25 | 0 | 42 | 0,1002 | 0,0385 | non-defined |
| Rv3811 | Rv3811 | 414G>R | 1 | 25 | 0 | 42 | 0,1002 | 0,0385 | non-defined |
| Rv3820c | papA2 | 167L>R | 1 | 25 | 0 | 42 | 0,1002 | 0,0385 | non-defined |
| Rv3822 | Rv3822 | 130S>N | 1 | 25 | 0 | 42 | 0,1002 | 0,0385 | non-defined |
| Rv3824c | papA1 | 290M>I | 1 | 25 | 0 | 42 | 0,1002 | 0,0385 | non-defined |
| Rv3832c | Rv3832c | 114L>M | 1 | 25 | 0 | 42 | 0,1002 | 0,0385 | non-defined |
| Rv3839 | Rv3839 | 131R>Q | 1 | 25 | 0 | 42 | 0,1002 | 0,0385 | non-defined |
| Rv3843c | Rv3843c | 256V>I | 1 | 25 | 0 | 42 | 0,1002 | 0,0385 | non-defined |
| Rv3852 | hns | 129R>Q | 1 | 25 | 0 | 42 | 0,1002 | 0,0385 | non-defined |
| Rv3854c | ethA | 131C>* | 1 | 25 | 0 | 42 | 0,1002 | 0,0385 | non-defined |
| Rv3854c | ethA | 197S>* | 1 | 25 | 0 | 42 | 0,1002 | 0,0385 | non-defined |
| Rv3854c | ethA | 399S>* | 1 | 25 | 0 | 42 | 0,1002 | 0,0385 | non-defined |
| Rv3854c | ethA | 43G>V | 1 | 25 | 0 | 42 | 0,1002 | 0,0385 | non-defined |

| Rv3854c | ethA | 50Y>C | 1 | 25 | 0 | 42 | 0,1002 | 0,0385 | non-defined |
| --- | --- | --- | --- | --- | --- | --- | --- | --- | --- |
| Rv3855 | ethR | 146I>M | 1 | 25 | 0 | 42 | 0,1002 | 0,0385 | non-defined |
| Rv3859c | gltB | 784V>I | 1 | 25 | 0 | 42 | 0,1002 | 0,0385 | non-defined |
| Rv3876 | Rv3876 | 563S>A | 1 | 25 | 0 | 42 | 0,1002 | 0,0385 | non-defined |
| Rv3877 | Rv3877 | 393F>L | 1 | 25 | 0 | 42 | 0,1002 | 0,0385 | non-defined |
| Rv3894c | Rv3894c | 901S>A | 1 | 25 | 0 | 42 | 0,1002 | 0,0385 | non-defined |
| Rv3897c | Rv3897c | 116A>G | 1 | 25 | 0 | 42 | 0,1002 | 0,0385 | non-defined |
| Rv3899c | Rv3899c | 90A>V | 1 | 25 | 0 | 42 | 0,1002 | 0,0385 | non-defined |
| Rv3901c | Rv3901c | 101L>V | 1 | 25 | 0 | 42 | 0,1002 | 0,0385 | non-defined |
| Rv3903c | Rv3903c | 35A>T | 1 | 25 | 0 | 42 | 0,1002 | 0,0385 | non-defined |
| Rv3909 | Rv3909 | 772R>H | 1 | 25 | 0 | 42 | 0,1002 | 0,0385 | non-defined |
| Rv3910 | Rv3910 | 158G>S | 1 | 25 | 0 | 42 | 0,1002 | 0,0385 | non-defined |
| Rv3915 | Rv3915 | 182R>H | 1 | 25 | 0 | 42 | 0,1002 | 0,0385 | non-defined |
| Rv3919c | gidB | 110V>A | 1 | 25 | 0 | 42 | 0,1002 | 0,0385 | non-defined |
| Rv3919c | gidB | 133A>P | 1 | 25 | 0 | 42 | 0,1002 | 0,0385 | non-defined |
| Rv3919c | gidB | 137R>W | 1 | 25 | 0 | 42 | 0,1002 | 0,0385 | non-defined |

| Rv3919c | gidB | 37G>A | 1 | 25 | 0 | 42 | 0,1002 | 0,0385 | non-defined |
| --- | --- | --- | --- | --- | --- | --- | --- | --- | --- |
| Rv3919c | gidB | 90L>R | 1 | 25 | 0 | 42 | 0,1002 | 0,0385 | non-defined |
| Rv3920c | Rv3920c | 124R>C | 1 | 25 | 0 | 42 | 0,1002 | 0,0385 | non-defined |
| Rv3921c | Rv3921c | 275G>S | 1 | 25 | 0 | 42 | 0,1002 | 0,0385 | non-defined |
| Rv0572c | Rv0572c | 31F>L | 23 | 3 | 32 | 10 | 0,1056 | 0,1227 | 2,3958 |
| Rv3190c | Rv3190c | 138L>P | 23 | 3 | 32 | 10 | 0,1056 | 0,1227 | 2,3958 |
| Rv3512 | PE_PGRS56 | 253A>G | 6 | 20 | 5 | 37 | 0,1120 | 0,1117 | 2,2200 |
| Rv0977 | PE_PGRS16 | 294N>S | 5 | 21 | 4 | 38 | 0,1255 | 0,0971 | 2,2619 |
| Rv3018c | PPE46 | 316L>V | 5 | 21 | 4 | 38 | 0,1255 | 0,0971 | 2,2619 |
| Rv0064 | Rv0064 | 457G>D | 26 | 0 | 40 | 2 | 0,1294 | 0,0476 | non-defined |
| Rv0064 | Rv0064 | 733N>D | 26 | 0 | 40 | 2 | 0,1294 | 0,0476 | non-defined |
| Rv0064 | Rv0064 | 906R>P | 26 | 0 | 40 | 2 | 0,1294 | 0,0476 | non-defined |
| Rv0962c | lprP | 186P>L | 26 | 0 | 40 | 2 | 0,1294 | 0,0476 | non-defined |
| Rv3894c | Rv3894c | 258R>P | 26 | 0 | 40 | 2 | 0,1294 | 0,0476 | non-defined |
| Rv2396 | PE_PGRS41 | 269V>I | 4 | 22 | 3 | 39 | 0,1386 | 0,0824 | 2,3636 |
| Rv3021c | PPE47 | 240L>V | 4 | 22 | 3 | 39 | 0,1386 | 0,0824 | 2,3636 |
| Rv1148c | Rv1148c | 354H>N | 3 | 23 | 2 | 40 | 0,1491 | 0,0678 | 2,6087 |
| Rv1387 | PPE20 | 325G>D | 3 | 23 | 2 | 40 | 0,1491 | 0,0678 | 2,6087 |
| Rv3590c | PE_PGRS58 | 141L>P | 3 | 23 | 2 | 40 | 0,1491 | 0,0678 | 2,6087 |
| Rv0236c | Rv0236c | 1080S>G | 23 | 3 | 33 | 9 | 0,1493 | 0,0989 | 2,0909 |
| Rv0406c | pks6 | 1402T>T | 23 | 3 | 33 | 9 | 0,1493 | 0,0989 | 2,0909 |
| Rv0412c | Rv0412c | 355D>Y | 23 | 3 | 33 | 9 | 0,1493 | 0,0989 | 2,0909 |
| Rv0538 | Rv0538 | 228R>P | 23 | 3 | 33 | 9 | 0,1493 | 0,0989 | 2,0909 |
| Rv0727c | fucA | 6A>D | 23 | 3 | 33 | 9 | 0,1493 | 0,0989 | 2,0909 |

| Rv1175c | fadH | 210C>S | 23 | 3 | 33 | 9 | 0,1493 | 0,0989 | 2,0909 |
| --- | --- | --- | --- | --- | --- | --- | --- | --- | --- |
| Rv1446c | opcA | 192R>P | 23 | 3 | 33 | 9 | 0,1493 | 0,0989 | 2,0909 |
| Rv1459c | Rv1459c | 113K>E | 23 | 3 | 33 | 9 | 0,1493 | 0,0989 | 2,0909 |
| Rv1486c | Rv1486c | 198K>N | 23 | 3 | 33 | 9 | 0,1493 | 0,0989 | 2,0909 |
| Rv1597 | Rv1597 | 21G>D | 23 | 3 | 33 | 9 | 0,1493 | 0,0989 | 2,0909 |
| Rv1716 | Rv1716 | 178S>G | 23 | 3 | 33 | 9 | 0,1493 | 0,0989 | 2,0909 |
| Rv1860 | apa | 136F>L | 23 | 3 | 33 | 9 | 0,1493 | 0,0989 | 2,0909 |
| Rv2078 | Rv2078 | 6E>G | 23 | 3 | 33 | 9 | 0,1493 | 0,0989 | 2,0909 |
| Rv2090 | Rv2090 | 358F>L | 23 | 3 | 33 | 9 | 0,1493 | 0,0989 | 2,0909 |
| Rv2178c | aroG | 265D>E | 23 | 3 | 33 | 9 | 0,1493 | 0,0989 | 2,0909 |
| Rv2560 | Rv2560 | 210V>L | 23 | 3 | 33 | 9 | 0,1493 | 0,0989 | 2,0909 |
| Rv2688c | Rv2688c | 156P>T | 23 | 3 | 33 | 9 | 0,1493 | 0,0989 | 2,0909 |
| Rv2807 | Rv2807 | 72E>V | 23 | 3 | 33 | 9 | 0,1493 | 0,0989 | 2,0909 |
| Rv3245c | mtrB | 517M>L | 23 | 3 | 33 | 9 | 0,1493 | 0,0989 | 2,0909 |
| Rv3329 | Rv3329 | 150Q>H | 23 | 3 | 33 | 9 | 0,1493 | 0,0989 | 2,0909 |
| Rv3833 | Rv3833 | 105V>I | 23 | 3 | 33 | 9 | 0,1493 | 0,0989 | 2,0909 |
| Rv0018c | ppp | 109N>S | 2 | 24 | 1 | 41 | 0,1500 | 0,0531 | 3,4167 |
| Rv0037c | Rv0037c | 347M>I | 2 | 24 | 1 | 41 | 0,1500 | 0,0531 | 3,4167 |
| Rv0042c | Rv0042c | 155E>G | 2 | 24 | 1 | 41 | 0,1500 | 0,0531 | 3,4167 |
| Rv0067c | Rv0067c | 126E>G | 2 | 24 | 1 | 41 | 0,1500 | 0,0531 | 3,4167 |
| Rv0101 | nrp | 2341I>L | 2 | 24 | 1 | 41 | 0,1500 | 0,0531 | 3,4167 |
| Rv0104 | Rv0104 | 393Y>D | 2 | 24 | 1 | 41 | 0,1500 | 0,0531 | 3,4167 |
| Rv0107c | ctpI | 1156V>A | 2 | 24 | 1 | 41 | 0,1500 | 0,0531 | 3,4167 |
| Rv0110 | Rv0110 | 162H>Y | 2 | 24 | 1 | 41 | 0,1500 | 0,0531 | 3,4167 |
| Rv0111 | Rv0111 | 618N>S | 2 | 24 | 1 | 41 | 0,1500 | 0,0531 | 3,4167 |
| Rv0143c | Rv0143c | 274W>S | 2 | 24 | 1 | 41 | 0,1500 | 0,0531 | 3,4167 |
| Rv0160c | PE4 | 139S>T | 2 | 24 | 1 | 41 | 0,1500 | 0,0531 | 3,4167 |
| Rv0215c | fadE3 | 59Y>H | 2 | 24 | 1 | 41 | 0,1500 | 0,0531 | 3,4167 |
| Rv0235c | Rv0235c | 143R>C | 2 | 24 | 1 | 41 | 0,1500 | 0,0531 | 3,4167 |
| Rv0257 | Rv0257 | 24G>R | 2 | 24 | 1 | 41 | 0,1500 | 0,0531 | 3,4167 |
| Rv0278c | PE_PGRS3 | 40M>I | 2 | 24 | 1 | 41 | 0,1500 | 0,0531 | 3,4167 |
| Rv0317c | glpQ2 | 30F>S | 2 | 24 | 1 | 41 | 0,1500 | 0,0531 | 3,4167 |
| Rv0321 | dcd | 153R>S | 2 | 24 | 1 | 41 | 0,1500 | 0,0531 | 3,4167 |

| Rv0355c | PPE8 | 1727G>D | 2 | 24 | 1 | 41 | 0,1500 | 0,0531 | 3,4167 |
| --- | --- | --- | --- | --- | --- | --- | --- | --- | --- |
| Rv0400c | fadE7 | 364M>I | 2 | 24 | 1 | 41 | 0,1500 | 0,0531 | 3,4167 |
| Rv0404 | fadD30 | 151S>F | 2 | 24 | 1 | 41 | 0,1500 | 0,0531 | 3,4167 |
| Rv0416 | thiS | 40V>M | 2 | 24 | 1 | 41 | 0,1500 | 0,0531 | 3,4167 |
| Rv0458 | Rv0458 | 474Q>P | 2 | 24 | 1 | 41 | 0,1500 | 0,0531 | 3,4167 |
| Rv0532 | PE_PGRS6 | 77V>L | 2 | 24 | 1 | 41 | 0,1500 | 0,0531 | 3,4167 |
| Rv0541c | Rv0541c | 122D>H | 2 | 24 | 1 | 41 | 0,1500 | 0,0531 | 3,4167 |
| Rv0589 | mce2A | 124A>V | 2 | 24 | 1 | 41 | 0,1500 | 0,0531 | 3,4167 |
| Rv0608 | Rv0608 | 38A>V | 2 | 24 | 1 | 41 | 0,1500 | 0,0531 | 3,4167 |
| Rv0695 | Rv0695 | 67E>D | 2 | 24 | 1 | 41 | 0,1500 | 0,0531 | 3,4167 |
| Rv0750 | Rv0750 | 46L>R | 2 | 24 | 1 | 41 | 0,1500 | 0,0531 | 3,4167 |
| Rv0754 | PE_PGRS11 | 228V>G | 2 | 24 | 1 | 41 | 0,1500 | 0,0531 | 3,4167 |
| Rv0825c | Rv0825c | 45R>G | 2 | 24 | 1 | 41 | 0,1500 | 0,0531 | 3,4167 |
| Rv0850 | Rv0850 | 63D>G | 2 | 24 | 1 | 41 | 0,1500 | 0,0531 | 3,4167 |
| Rv0892 | Rv0892 | 421K>R | 2 | 24 | 1 | 41 | 0,1500 | 0,0531 | 3,4167 |
| Rv0934 | pstS1 | 314Y>C | 2 | 24 | 1 | 41 | 0,1500 | 0,0531 | 3,4167 |
| Rv0946c | pgi | 546R>H | 2 | 24 | 1 | 41 | 0,1500 | 0,0531 | 3,4167 |
| Rv0982 | mprB | 498S>A | 2 | 24 | 1 | 41 | 0,1500 | 0,0531 | 3,4167 |
| Rv1020 | mfd | 799V>A | 2 | 24 | 1 | 41 | 0,1500 | 0,0531 | 3,4167 |
| Rv1032c | trcS | 136A>G | 2 | 24 | 1 | 41 | 0,1500 | 0,0531 | 3,4167 |
| Rv1125 | Rv1125 | 313R>Q | 2 | 24 | 1 | 41 | 0,1500 | 0,0531 | 3,4167 |
| Rv1126c | Rv1126c | 192D>E | 2 | 24 | 1 | 41 | 0,1500 | 0,0531 | 3,4167 |
| Rv1133c | metE | 575P>S | 2 | 24 | 1 | 41 | 0,1500 | 0,0531 | 3,4167 |
| Rv1140 | Rv1140 | 269G>C | 2 | 24 | 1 | 41 | 0,1500 | 0,0531 | 3,4167 |
| Rv1158c | Rv1158c | 83G>W | 2 | 24 | 1 | 41 | 0,1500 | 0,0531 | 3,4167 |
| Rv1198 | esxL | 23L>S | 2 | 24 | 1 | 41 | 0,1500 | 0,0531 | 3,4167 |
| Rv1204c | Rv1204c | 234L>P | 2 | 24 | 1 | 41 | 0,1500 | 0,0531 | 3,4167 |
| Rv1230c | Rv1230c | 343P>R | 2 | 24 | 1 | 41 | 0,1500 | 0,0531 | 3,4167 |
| Rv1280c | oppA | 43L>I | 2 | 24 | 1 | 41 | 0,1500 | 0,0531 | 3,4167 |
| Rv1350 | fabG | 232S>L | 2 | 24 | 1 | 41 | 0,1500 | 0,0531 | 3,4167 |
| Rv1511 | gmdA | 161Y>S | 2 | 24 | 1 | 41 | 0,1500 | 0,0531 | 3,4167 |
| Rv1551 | plsB1 | 52A>V | 2 | 24 | 1 | 41 | 0,1500 | 0,0531 | 3,4167 |
| Rv1563c | treY | 112D>Y | 2 | 24 | 1 | 41 | 0,1500 | 0,0531 | 3,4167 |

| Rv1738 | Rv1738 | 52R>H | 2 | 24 | 1 | 41 | 0,1500 | 0,0531 | 3,4167 |
| --- | --- | --- | --- | --- | --- | --- | --- | --- | --- |
| Rv1770 | Rv1770 | 331G>D | 2 | 24 | 1 | 41 | 0,1500 | 0,0531 | 3,4167 |
| Rv1783 | Rv1783 | 229Q>R | 2 | 24 | 1 | 41 | 0,1500 | 0,0531 | 3,4167 |
| Rv1787 | PPE25 | 242Q>R | 2 | 24 | 1 | 41 | 0,1500 | 0,0531 | 3,4167 |
| Rv1787 | PPE25 | 245A>T | 2 | 24 | 1 | 41 | 0,1500 | 0,0531 | 3,4167 |
| Rv1846c | Rv1846c | 68R>G | 2 | 24 | 1 | 41 | 0,1500 | 0,0531 | 3,4167 |
| Rv1920 | Rv1920 | 100P>L | 2 | 24 | 1 | 41 | 0,1500 | 0,0531 | 3,4167 |
| Rv1937 | Rv1937 | 664R>S | 2 | 24 | 1 | 41 | 0,1500 | 0,0531 | 3,4167 |
| Rv1966 | mce3A | 67Q>R | 2 | 24 | 1 | 41 | 0,1500 | 0,0531 | 3,4167 |
| Rv1999c | Rv1999c | 94W>C | 2 | 24 | 1 | 41 | 0,1500 | 0,0531 | 3,4167 |
| Rv2052c | Rv2052c | 512R>W | 2 | 24 | 1 | 41 | 0,1500 | 0,0531 | 3,4167 |
| Rv2056c | rpsN | 12R>Q | 2 | 24 | 1 | 41 | 0,1500 | 0,0531 | 3,4167 |
| Rv2124c | metH | 125G>R | 2 | 24 | 1 | 41 | 0,1500 | 0,0531 | 3,4167 |
| Rv2158c | murE | 25G>V | 2 | 24 | 1 | 41 | 0,1500 | 0,0531 | 3,4167 |
| Rv2262c | Rv2262c | 206P>A | 2 | 24 | 1 | 41 | 0,1500 | 0,0531 | 3,4167 |
| Rv2284 | lipM | 144R>Q | 2 | 24 | 1 | 41 | 0,1500 | 0,0531 | 3,4167 |
| Rv2323c | Rv2323c | 61W>R | 2 | 24 | 1 | 41 | 0,1500 | 0,0531 | 3,4167 |
| Rv2382c | mbtC | 211Y>C | 2 | 24 | 1 | 41 | 0,1500 | 0,0531 | 3,4167 |
| Rv2383c | mbtB | 412S>R | 2 | 24 | 1 | 41 | 0,1500 | 0,0531 | 3,4167 |
| Rv2395 | Rv2395 | 292S>* | 2 | 24 | 1 | 41 | 0,1500 | 0,0531 | 3,4167 |
| Rv2601 | speE | 391D>Y | 2 | 24 | 1 | 41 | 0,1500 | 0,0531 | 3,4167 |
| Rv2689c | Rv2689c | 99L>R | 2 | 24 | 1 | 41 | 0,1500 | 0,0531 | 3,4167 |
| Rv2707 | Rv2707 | 21L>F | 2 | 24 | 1 | 41 | 0,1500 | 0,0531 | 3,4167 |
| Rv2753c | dapA | 29L>P | 2 | 24 | 1 | 41 | 0,1500 | 0,0531 | 3,4167 |
| Rv2777c | Rv2777c | 102H>Q | 2 | 24 | 1 | 41 | 0,1500 | 0,0531 | 3,4167 |
| Rv2782c | pepR | 228P>L | 2 | 24 | 1 | 41 | 0,1500 | 0,0531 | 3,4167 |
| Rv2850c | Rv2850c | 374R>G | 2 | 24 | 1 | 41 | 0,1500 | 0,0531 | 3,4167 |
| Rv2934 | ppsD | 1782M>I | 2 | 24 | 1 | 41 | 0,1500 | 0,0531 | 3,4167 |
| Rv2935 | ppsE | 3I>V | 2 | 24 | 1 | 41 | 0,1500 | 0,0531 | 3,4167 |
| Rv2946c | pks1 | 599G>D | 2 | 24 | 1 | 41 | 0,1500 | 0,0531 | 3,4167 |
| Rv2961 | Rv2961 | 96G>S | 2 | 24 | 1 | 41 | 0,1500 | 0,0531 | 3,4167 |
| Rv2975c | Rv2975c | 37F>V | 2 | 24 | 1 | 41 | 0,1500 | 0,0531 | 3,4167 |
| Rv3016 | lpqA | 63T>M | 2 | 24 | 1 | 41 | 0,1500 | 0,0531 | 3,4167 |

| Rv3061c | fadE22 | 393V>A | 2 | 24 | 1 | 41 | 0,1500 | 0,0531 | 3,4167 |
| --- | --- | --- | --- | --- | --- | --- | --- | --- | --- |
| Rv3090 | Rv3090 | 76L>P | 2 | 24 | 1 | 41 | 0,1500 | 0,0531 | 3,4167 |
| Rv3096 | Rv3096 | 249P>S | 2 | 24 | 1 | 41 | 0,1500 | 0,0531 | 3,4167 |
| Rv3148 | nuoD | 392G>A | 2 | 24 | 1 | 41 | 0,1500 | 0,0531 | 3,4167 |
| Rv3174 | Rv3174 | 2T>A | 2 | 24 | 1 | 41 | 0,1500 | 0,0531 | 3,4167 |
| Rv3193c | Rv3193c | 673A>V | 2 | 24 | 1 | 41 | 0,1500 | 0,0531 | 3,4167 |
| Rv3270 | ctpC | 633D>N | 2 | 24 | 1 | 41 | 0,1500 | 0,0531 | 3,4167 |
| Rv3297 | nei | 206W>C | 2 | 24 | 1 | 41 | 0,1500 | 0,0531 | 3,4167 |
| Rv3341 | metX | 278D>E | 2 | 24 | 1 | 41 | 0,1500 | 0,0531 | 3,4167 |
| Rv3370c | dnaE2 | 355V>I | 2 | 24 | 1 | 41 | 0,1500 | 0,0531 | 3,4167 |
| Rv3379c | dxs2 | 513C>S | 2 | 24 | 1 | 41 | 0,1500 | 0,0531 | 3,4167 |
| Rv3429 | PPE59 | 67A>V | 2 | 24 | 1 | 41 | 0,1500 | 0,0531 | 3,4167 |
| Rv3429 | PPE59 | 68G>E | 2 | 24 | 1 | 41 | 0,1500 | 0,0531 | 3,4167 |
| Rv3429 | PPE59 | 72D>Q | 2 | 24 | 1 | 41 | 0,1500 | 0,0531 | 3,4167 |
| Rv3431c | Rv3431c | 172T>N | 2 | 24 | 1 | 41 | 0,1500 | 0,0531 | 3,4167 |
| Rv3434c | Rv3434c | 68L>R | 2 | 24 | 1 | 41 | 0,1500 | 0,0531 | 3,4167 |
| Rv3448 | Rv3448 | 133G>S | 2 | 24 | 1 | 41 | 0,1500 | 0,0531 | 3,4167 |
| Rv3478 | PPE60 | 371S>R | 2 | 24 | 1 | 41 | 0,1500 | 0,0531 | 3,4167 |
| Rv3508 | PE_PGRS54 | 153P>A | 2 | 24 | 1 | 41 | 0,1500 | 0,0531 | 3,4167 |
| Rv3521 | Rv3521 | 189Y>S | 2 | 24 | 1 | 41 | 0,1500 | 0,0531 | 3,4167 |
| Rv3573c | fadE34 | 371A>V | 2 | 24 | 1 | 41 | 0,1500 | 0,0531 | 3,4167 |
| Rv3682 | ponA2 | 641D>N | 2 | 24 | 1 | 41 | 0,1500 | 0,0531 | 3,4167 |
| Rv3704c | gshA | 385R>L | 2 | 24 | 1 | 41 | 0,1500 | 0,0531 | 3,4167 |
| Rv3729 | Rv3729 | 549R>K | 2 | 24 | 1 | 41 | 0,1500 | 0,0531 | 3,4167 |
| Rv3773c | Rv3773c | 159K>R | 2 | 24 | 1 | 41 | 0,1500 | 0,0531 | 3,4167 |
| Rv3795 | embB | 306M>V | 2 | 24 | 1 | 41 | 0,1500 | 0,0531 | 3,4167 |
| Rv3797 | fadE35 | 417D>N | 2 | 24 | 1 | 41 | 0,1500 | 0,0531 | 3,4167 |
| Rv3823c | mmpL8 | 1042A>E | 2 | 24 | 1 | 41 | 0,1500 | 0,0531 | 3,4167 |
| Rv3823c | mmpL8 | 891R>C | 2 | 24 | 1 | 41 | 0,1500 | 0,0531 | 3,4167 |
| Rv3824c | papA1 | 303G>D | 2 | 24 | 1 | 41 | 0,1500 | 0,0531 | 3,4167 |
| Rv3850 | Rv3850 | 125V>F | 2 | 24 | 1 | 41 | 0,1500 | 0,0531 | 3,4167 |
| Rv3871 | Rv3871 | 74S>F | 2 | 24 | 1 | 41 | 0,1500 | 0,0531 | 3,4167 |
| Rv3879c | Rv3879c | 703V>A | 2 | 24 | 1 | 41 | 0,1500 | 0,0531 | 3,4167 |

| Rv3900c | Rv3900c | 63A>P | 2 | 24 | 1 | 41 | 0,1500 | 0,0531 | 3,4167 |
| --- | --- | --- | --- | --- | --- | --- | --- | --- | --- |
| Rv3910 | Rv3910 | 212K>N | 2 | 24 | 1 | 41 | 0,1500 | 0,0531 | 3,4167 |
| Rv3913 | trxB2 | 2T>N | 2 | 24 | 1 | 41 | 0,1500 | 0,0531 | 3,4167 |
| Rv3915 | Rv3915 | 237M>V | 2 | 24 | 1 | 41 | 0,1500 | 0,0531 | 3,4167 |
| Rv4007 | Rv4007 | 61V>I | 2 | 24 | 1 | 41 | 0,1500 | 0,0531 | 3,4167 |
| Rv1651c | PE_PGRS30 | 191N>S | 7 | 19 | 7 | 35 | 0,1547 | 0,1026 | 1,8421 |
| Rv0797 | Rv0797 | 54W>* | 15 | 11 | 19 | 23 | 0,1591 | 0,1245 | 1,6507 |
| Rv3021c | PPE47 | 222G>A | 19 | 7 | 26 | 16 | 0,1720 | 0,1117 | 1,6703 |
| Rv0980c | PE_PGRS18 | 43H>Q | 6 | 20 | 6 | 36 | 0,1777 | 0,0879 | 1,8000 |
| Rv1047 | Rv1047 | 328Q>K | 20 | 6 | 28 | 14 | 0,1835 | 0,1026 | 1,6667 |
| Rv3023c | Rv3023c | 328Q>K | 20 | 6 | 28 | 14 | 0,1835 | 0,1026 | 1,6667 |
| Rv3115 | Rv3115 | 328Q>K | 20 | 6 | 28 | 14 | 0,1835 | 0,1026 | 1,6667 |
| Rv1945 | Rv1945 | 326N>H | 8 | 18 | 9 | 33 | 0,1937 | 0,0934 | 1,6296 |
| Rv1945 | Rv1945 | 328H>N | 8 | 18 | 9 | 33 | 0,1937 | 0,0934 | 1,6296 |
| Rv2048c | pks12 | 1652R>C | 5 | 21 | 5 | 37 | 0,2036 | 0,0733 | 1,7619 |
| Rv3018c | PPE46 | 319V>L | 5 | 21 | 5 | 37 | 0,2036 | 0,0733 | 1,7619 |
| Rv3018c | PPE46 | 325L>V | 5 | 21 | 5 | 37 | 0,2036 | 0,0733 | 1,7619 |
| Rv3879c | Rv3879c | 39L>W | 5 | 21 | 5 | 37 | 0,2036 | 0,0733 | 1,7619 |
| Rv1733c | Rv1733c | 68Q>H | 23 | 3 | 34 | 8 | 0,2069 | 0,0751 | 1,8039 |
| Rv1199c | Rv1199c | 328Q>K | 15 | 11 | 20 | 22 | 0,2096 | 0,1007 | 1,5000 |
| Rv0082 | Rv0082 | 74Q>R | 26 | 0 | 41 | 1 | 0,2140 | 0,0238 | non-defined |
| Rv0323c | Rv0323c | 142S>G | 26 | 0 | 41 | 1 | 0,2140 | 0,0238 | non-defined |
| Rv2650c | Rv2650c | 101I>T | 26 | 0 | 41 | 1 | 0,2140 | 0,0238 | non-defined |
| Rv2668 | Rv2668 | 3H>R | 26 | 0 | 41 | 1 | 0,2140 | 0,0238 | non-defined |
| Rv3063 | cstA | 559R>S | 26 | 0 | 41 | 1 | 0,2140 | 0,0238 | non-defined |
| Rv0012 | Rv0012 | 55D>N | 13 | 13 | 17 | 25 | 0,2211 | 0,0952 | 1,4706 |
| Rv0030 | Rv0030 | 110*>G | 13 | 13 | 17 | 25 | 0,2211 | 0,0952 | 1,4706 |
| Rv0059 | Rv0059 | 191P>L | 13 | 13 | 17 | 25 | 0,2211 | 0,0952 | 1,4706 |

| Rv0134 | ephF | 271G>S | 13 | 13 | 17 | 25 | 0,2211 | 0,0952 | 1,4706 |
| --- | --- | --- | --- | --- | --- | --- | --- | --- | --- |
| Rv0152c | PE2 | 291G>E | 13 | 13 | 17 | 25 | 0,2211 | 0,0952 | 1,4706 |
| Rv0172 | mce1D | 265A>V | 13 | 13 | 17 | 25 | 0,2211 | 0,0952 | 1,4706 |
| Rv0175 | Rv0175 | 160M>T | 13 | 13 | 17 | 25 | 0,2211 | 0,0952 | 1,4706 |
| Rv0181c | Rv0181c | 220R>H | 13 | 13 | 17 | 25 | 0,2211 | 0,0952 | 1,4706 |
| Rv0264c | Rv0264c | 96G>A | 13 | 13 | 17 | 25 | 0,2211 | 0,0952 | 1,4706 |
| Rv0366c | Rv0366c | 155N>K | 13 | 13 | 17 | 25 | 0,2211 | 0,0952 | 1,4706 |
| Rv0446c | Rv0446c | 175W>* | 13 | 13 | 17 | 25 | 0,2211 | 0,0952 | 1,4706 |
| Rv0472c | Rv0472c | 3E>G | 13 | 13 | 17 | 25 | 0,2211 | 0,0952 | 1,4706 |
| Rv0493c | Rv0493c | 85P>S | 13 | 13 | 17 | 25 | 0,2211 | 0,0952 | 1,4706 |
| Rv0613c | Rv0613c | 97T>I | 13 | 13 | 17 | 25 | 0,2211 | 0,0952 | 1,4706 |
| Rv0631c | recC | 535R>M | 13 | 13 | 17 | 25 | 0,2211 | 0,0952 | 1,4706 |
| Rv0655 | mkl | 276E>G | 13 | 13 | 17 | 25 | 0,2211 | 0,0952 | 1,4706 |
| Rv0696 | Rv0696 | 331G>C | 13 | 13 | 17 | 25 | 0,2211 | 0,0952 | 1,4706 |
| Rv0791c | Rv0791c | 100S>C | 13 | 13 | 17 | 25 | 0,2211 | 0,0952 | 1,4706 |
| Rv0850 | Rv0850 | 40S>K | 13 | 13 | 17 | 25 | 0,2211 | 0,0952 | 1,4706 |
| Rv0990c | Rv0990c | 61H>Q | 13 | 13 | 17 | 25 | 0,2211 | 0,0952 | 1,4706 |
| Rv1075c | Rv1075c | 275P>L | 13 | 13 | 17 | 25 | 0,2211 | 0,0952 | 1,4706 |
| Rv1256c | cyp130 | 145K>N | 13 | 13 | 17 | 25 | 0,2211 | 0,0952 | 1,4706 |
| Rv1278 | Rv1278 | 365A>S | 13 | 13 | 17 | 25 | 0,2211 | 0,0952 | 1,4706 |
| Rv1292 | argS | 182I>S | 13 | 13 | 17 | 25 | 0,2211 | 0,0952 | 1,4706 |
| Rv1318c | Rv1318c | 267F>L | 13 | 13 | 17 | 25 | 0,2211 | 0,0952 | 1,4706 |
| Rv1347c | Rv1347c | 192D>A | 13 | 13 | 17 | 25 | 0,2211 | 0,0952 | 1,4706 |
| Rv1537 | dinX | 311T>P | 13 | 13 | 17 | 25 | 0,2211 | 0,0952 | 1,4706 |
| Rv1674c | Rv1674c | 189E>G | 13 | 13 | 17 | 25 | 0,2211 | 0,0952 | 1,4706 |
| Rv1716 | Rv1716 | 6P>L | 13 | 13 | 17 | 25 | 0,2211 | 0,0952 | 1,4706 |
| Rv1722 | Rv1722 | 15V>L | 13 | 13 | 17 | 25 | 0,2211 | 0,0952 | 1,4706 |
| Rv1971 | mce3F | 331P>R | 13 | 13 | 17 | 25 | 0,2211 | 0,0952 | 1,4706 |
| Rv1987 | Rv1987 | 36S>N | 13 | 13 | 17 | 25 | 0,2211 | 0,0952 | 1,4706 |
| Rv2438c | nadE | 133R>L | 13 | 13 | 17 | 25 | 0,2211 | 0,0952 | 1,4706 |
| Rv2446c | Rv2446c | 84A>T | 13 | 13 | 17 | 25 | 0,2211 | 0,0952 | 1,4706 |
| Rv2561 | Rv2561 | 16Y>C | 13 | 13 | 17 | 25 | 0,2211 | 0,0952 | 1,4706 |
| Rv2764c | thyA | 202T>A | 13 | 13 | 17 | 25 | 0,2211 | 0,0952 | 1,4706 |

| Rv2768c | PPE43 | 347G>V | 13 | 13 | 17 | 25 | 0,2211 | 0,0952 | 1,4706 |
| --- | --- | --- | --- | --- | --- | --- | --- | --- | --- |
| Rv2791c | Rv2791c | 155R>S | 13 | 13 | 17 | 25 | 0,2211 | 0,0952 | 1,4706 |
| Rv2854 | Rv2854 | 308V>A | 13 | 13 | 17 | 25 | 0,2211 | 0,0952 | 1,4706 |
| Rv2864c | Rv2864c | 522I>V | 13 | 13 | 17 | 25 | 0,2211 | 0,0952 | 1,4706 |
| Rv2922c | smc | 526R>L | 13 | 13 | 17 | 25 | 0,2211 | 0,0952 | 1,4706 |
| Rv3057c | Rv3057c | 112D>A | 13 | 13 | 17 | 25 | 0,2211 | 0,0952 | 1,4706 |
| Rv3063 | cstA | 654Y>D | 13 | 13 | 17 | 25 | 0,2211 | 0,0952 | 1,4706 |
| Rv3092c | Rv3092c | 250P>L | 13 | 13 | 17 | 25 | 0,2211 | 0,0952 | 1,4706 |
| Rv3179 | Rv3179 | 342Y>H | 13 | 13 | 17 | 25 | 0,2211 | 0,0952 | 1,4706 |
| Rv3217c | Rv3217c | 38A>T | 13 | 13 | 17 | 25 | 0,2211 | 0,0952 | 1,4706 |
| Rv3234c | Rv3234c | 250R>H | 13 | 13 | 17 | 25 | 0,2211 | 0,0952 | 1,4706 |
| Rv3365c | Rv3365c | 266A>T | 13 | 13 | 17 | 25 | 0,2211 | 0,0952 | 1,4706 |
| Rv3439c | Rv3439c | 96G>E | 13 | 13 | 17 | 25 | 0,2211 | 0,0952 | 1,4706 |
| Rv3451 | cut3 | 259L>R | 13 | 13 | 17 | 25 | 0,2211 | 0,0952 | 1,4706 |
| Rv3544c | fadE28 | 292I>L | 13 | 13 | 17 | 25 | 0,2211 | 0,0952 | 1,4706 |
| Rv3636 | Rv3636 | 69A>D | 13 | 13 | 17 | 25 | 0,2211 | 0,0952 | 1,4706 |
| Rv3693 | Rv3693 | 129M>I | 13 | 13 | 17 | 25 | 0,2211 | 0,0952 | 1,4706 |
| Rv3720 | Rv3720 | 77H>R | 13 | 13 | 17 | 25 | 0,2211 | 0,0952 | 1,4706 |
| Rv3727 | Rv3727 | 537I>T | 13 | 13 | 17 | 25 | 0,2211 | 0,0952 | 1,4706 |
| Rv3775 | lipE | 164D>N | 13 | 13 | 17 | 25 | 0,2211 | 0,0952 | 1,4706 |
| Rv3786c | Rv3786c | 100T>I | 13 | 13 | 17 | 25 | 0,2211 | 0,0952 | 1,4706 |
| Rv3802c | Rv3802c | 50V>F | 13 | 13 | 17 | 25 | 0,2211 | 0,0952 | 1,4706 |
| Rv3824c | papA1 | 35L>F | 13 | 13 | 17 | 25 | 0,2211 | 0,0952 | 1,4706 |
| Rv3842c | glpQ1 | 60D>G | 13 | 13 | 17 | 25 | 0,2211 | 0,0952 | 1,4706 |
| Rv3864 | Rv3864 | 21L>V | 13 | 13 | 17 | 25 | 0,2211 | 0,0952 | 1,4706 |
| Rv3919c | gidB | 16L>R | 13 | 13 | 17 | 25 | 0,2211 | 0,0952 | 1,4706 |
| Rv3798 | Rv3798 | 433L>R | 9 | 17 | 11 | 31 | 0,2294 | 0,0842 | 1,4920 |
| Rv3021c | PPE47 | 243V>L | 4 | 22 | 4 | 38 | 0,2330 | 0,0586 | 1,7273 |
| Rv3021c | PPE47 | 249L>V | 4 | 22 | 4 | 38 | 0,2330 | 0,0586 | 1,7273 |
| Rv0797 | Rv0797 | 111M>L | 14 | 12 | 19 | 23 | 0,2450 | 0,0861 | 1,4123 |
| Rv2933 | ppsC | 1899N>S | 6 | 20 | 7 | 35 | 0,2568 | 0,0641 | 1,5000 |
| Rv3879c | Rv3879c | 729C>S | 8 | 18 | 10 | 32 | 0,2636 | 0,0696 | 1,4222 |
| Rv0021c | Rv0021c | 169I>V | 3 | 23 | 3 | 39 | 0,2673 | 0,0440 | 1,6957 |

| Rv0021c | Rv0021c | 77Q>* | 3 | 23 | 3 | 39 | 0,2673 | 0,0440 | 1,6957 |
| --- | --- | --- | --- | --- | --- | --- | --- | --- | --- |
| Rv0058 | dnaB | 611D>N | 3 | 23 | 3 | 39 | 0,2673 | 0,0440 | 1,6957 |
| Rv0062 | celA1 | 264G>D | 3 | 23 | 3 | 39 | 0,2673 | 0,0440 | 1,6957 |
| Rv0071 | Rv0071 | 19P>L | 3 | 23 | 3 | 39 | 0,2673 | 0,0440 | 1,6957 |
| Rv0172 | mce1D | 410V>A | 3 | 23 | 3 | 39 | 0,2673 | 0,0440 | 1,6957 |
| Rv0197 | Rv0197 | 644N>H | 3 | 23 | 3 | 39 | 0,2673 | 0,0440 | 1,6957 |
| Rv0244c | fadE5 | 71T>P | 3 | 23 | 3 | 39 | 0,2673 | 0,0440 | 1,6957 |
| Rv0298 | Rv0298 | 10V>M | 3 | 23 | 3 | 39 | 0,2673 | 0,0440 | 1,6957 |
| Rv0341 | iniB | 70A>T | 3 | 23 | 3 | 39 | 0,2673 | 0,0440 | 1,6957 |
| Rv0355c | PPE8 | 2755Y>F | 3 | 23 | 3 | 39 | 0,2673 | 0,0440 | 1,6957 |
| Rv0355c | PPE8 | 2757T>I | 3 | 23 | 3 | 39 | 0,2673 | 0,0440 | 1,6957 |
| Rv0355c | PPE8 | 2759S>T | 3 | 23 | 3 | 39 | 0,2673 | 0,0440 | 1,6957 |
| Rv0355c | PPE8 | 2760Y>F | 3 | 23 | 3 | 39 | 0,2673 | 0,0440 | 1,6957 |
| Rv0421c | Rv0421c | 94A>V | 3 | 23 | 3 | 39 | 0,2673 | 0,0440 | 1,6957 |
| Rv0513 | Rv0513 | 3P>S | 3 | 23 | 3 | 39 | 0,2673 | 0,0440 | 1,6957 |
| Rv0522 | gabP | 318S>P | 3 | 23 | 3 | 39 | 0,2673 | 0,0440 | 1,6957 |
| Rv0533c | fabH | 13V>I | 3 | 23 | 3 | 39 | 0,2673 | 0,0440 | 1,6957 |
| Rv0584 | Rv0584 | 260G>V | 3 | 23 | 3 | 39 | 0,2673 | 0,0440 | 1,6957 |
| Rv0641 | rplA | 129R>W | 3 | 23 | 3 | 39 | 0,2673 | 0,0440 | 1,6957 |
| Rv0667 | rpoB | 695V>L | 3 | 23 | 3 | 39 | 0,2673 | 0,0440 | 1,6957 |
| Rv0716 | rplE | 94V>A | 3 | 23 | 3 | 39 | 0,2673 | 0,0440 | 1,6957 |
| Rv0853c | pdc | 494S>R | 3 | 23 | 3 | 39 | 0,2673 | 0,0440 | 1,6957 |
| Rv0940c | Rv0940c | 215K>T | 3 | 23 | 3 | 39 | 0,2673 | 0,0440 | 1,6957 |
| Rv0961 | Rv0961 | 68W>S | 3 | 23 | 3 | 39 | 0,2673 | 0,0440 | 1,6957 |
| Rv0972c | fadE12 | 33D>N | 3 | 23 | 3 | 39 | 0,2673 | 0,0440 | 1,6957 |
| Rv0999 | Rv0999 | 81T>A | 3 | 23 | 3 | 39 | 0,2673 | 0,0440 | 1,6957 |
| Rv1159 | pimE | 53G>S | 3 | 23 | 3 | 39 | 0,2673 | 0,0440 | 1,6957 |
| Rv1189 | sigI | 24R>P | 3 | 23 | 3 | 39 | 0,2673 | 0,0440 | 1,6957 |
| Rv1198 | esxL | 39S>G | 3 | 23 | 3 | 39 | 0,2673 | 0,0440 | 1,6957 |
| Rv1204c | Rv1204c | 393G>C | 3 | 23 | 3 | 39 | 0,2673 | 0,0440 | 1,6957 |
| Rv1308 | atpA | 54E>G | 3 | 23 | 3 | 39 | 0,2673 | 0,0440 | 1,6957 |
| Rv1319c | Rv1319c | 416I>L | 3 | 23 | 3 | 39 | 0,2673 | 0,0440 | 1,6957 |
| Rv1321 | Rv1321 | 39S>G | 3 | 23 | 3 | 39 | 0,2673 | 0,0440 | 1,6957 |

| Rv1324 | Rv1324 | 237D>N | 3 | 23 | 3 | 39 | 0,2673 | 0,0440 | 1,6957 |
| --- | --- | --- | --- | --- | --- | --- | --- | --- | --- |
| Rv1336 | cysM | 212Y>C | 3 | 23 | 3 | 39 | 0,2673 | 0,0440 | 1,6957 |
| Rv1355c | moeY | 244R>* | 3 | 23 | 3 | 39 | 0,2673 | 0,0440 | 1,6957 |
| Rv1355c | moeY | 662H>Y | 3 | 23 | 3 | 39 | 0,2673 | 0,0440 | 1,6957 |
| Rv1429 | Rv1429 | 135R>L | 3 | 23 | 3 | 39 | 0,2673 | 0,0440 | 1,6957 |
| Rv1504c | Rv1504c | 73E>D | 3 | 23 | 3 | 39 | 0,2673 | 0,0440 | 1,6957 |
| Rv1508A | Rv1508A | 75P>S | 3 | 23 | 3 | 39 | 0,2673 | 0,0440 | 1,6957 |
| Rv1547 | dnaE | 618P>R | 3 | 23 | 3 | 39 | 0,2673 | 0,0440 | 1,6957 |
| Rv1565c | Rv1565c | 605T>S | 3 | 23 | 3 | 39 | 0,2673 | 0,0440 | 1,6957 |
| Rv1588c | Rv1588c | 24D>A | 3 | 23 | 3 | 39 | 0,2673 | 0,0440 | 1,6957 |
| Rv1600 | hisC1 | 23A>V | 3 | 23 | 3 | 39 | 0,2673 | 0,0440 | 1,6957 |
| Rv1607 | chaA | 35A>T | 3 | 23 | 3 | 39 | 0,2673 | 0,0440 | 1,6957 |
| Rv1668c | Rv1668c | 32R>S | 3 | 23 | 3 | 39 | 0,2673 | 0,0440 | 1,6957 |
| Rv1673c | Rv1673c | 32G>S | 3 | 23 | 3 | 39 | 0,2673 | 0,0440 | 1,6957 |
| Rv1731 | gabD2 | 137G>E | 3 | 23 | 3 | 39 | 0,2673 | 0,0440 | 1,6957 |
| Rv1750c | fadD1 | 517P>L | 3 | 23 | 3 | 39 | 0,2673 | 0,0440 | 1,6957 |
| Rv1769 | Rv1769 | 314G>D | 3 | 23 | 3 | 39 | 0,2673 | 0,0440 | 1,6957 |
| Rv1787 | PPE25 | 191G>R | 3 | 23 | 3 | 39 | 0,2673 | 0,0440 | 1,6957 |
| Rv1809 | PPE33 | 300G>E | 3 | 23 | 3 | 39 | 0,2673 | 0,0440 | 1,6957 |
| Rv1863c | Rv1863c | 83R>H | 3 | 23 | 3 | 39 | 0,2673 | 0,0440 | 1,6957 |
| Rv1886c | fbpB | 186A>T | 3 | 23 | 3 | 39 | 0,2673 | 0,0440 | 1,6957 |
| Rv1980c | mpt64 | 35T>A | 3 | 23 | 3 | 39 | 0,2673 | 0,0440 | 1,6957 |
| Rv2077c | Rv2077c | 181Q>P | 3 | 23 | 3 | 39 | 0,2673 | 0,0440 | 1,6957 |
| Rv2078 | Rv2078 | 97A>T | 3 | 23 | 3 | 39 | 0,2673 | 0,0440 | 1,6957 |
| Rv2237 | Rv2237 | 59L>M | 3 | 23 | 3 | 39 | 0,2673 | 0,0440 | 1,6957 |
| Rv2308 | Rv2308 | 31T>A | 3 | 23 | 3 | 39 | 0,2673 | 0,0440 | 1,6957 |
| Rv2339 | mmpL9 | 785M>T | 3 | 23 | 3 | 39 | 0,2673 | 0,0440 | 1,6957 |
| Rv2368c | phoH1 | 210A>V | 3 | 23 | 3 | 39 | 0,2673 | 0,0440 | 1,6957 |
| Rv2373c | dnaJ2 | 217I>V | 3 | 23 | 3 | 39 | 0,2673 | 0,0440 | 1,6957 |
| Rv2380c | mbtE | 865E>D | 3 | 23 | 3 | 39 | 0,2673 | 0,0440 | 1,6957 |
| Rv2383c | mbtB | 1000S>L | 3 | 23 | 3 | 39 | 0,2673 | 0,0440 | 1,6957 |
| Rv2385 | mbtJ | 8G>V | 3 | 23 | 3 | 39 | 0,2673 | 0,0440 | 1,6957 |
| Rv2403c | lppR | 50D>E | 3 | 23 | 3 | 39 | 0,2673 | 0,0440 | 1,6957 |

| Rv2414c | Rv2414c | 126A>E | 3 | 23 | 3 | 39 | 0,2673 | 0,0440 | 1,6957 |
| --- | --- | --- | --- | --- | --- | --- | --- | --- | --- |
| Rv2452c | Rv2452c | 36A>V | 3 | 23 | 3 | 39 | 0,2673 | 0,0440 | 1,6957 |
| Rv2511 | orn | 70D>N | 3 | 23 | 3 | 39 | 0,2673 | 0,0440 | 1,6957 |
| Rv2561 | Rv2561 | 37L>F | 3 | 23 | 3 | 39 | 0,2673 | 0,0440 | 1,6957 |
| Rv2612c | pgsA1 | 87V>L | 3 | 23 | 3 | 39 | 0,2673 | 0,0440 | 1,6957 |
| Rv2664 | Rv2664 | 21A>V | 3 | 23 | 3 | 39 | 0,2673 | 0,0440 | 1,6957 |
| Rv2692 | ceoC | 24H>Q | 3 | 23 | 3 | 39 | 0,2673 | 0,0440 | 1,6957 |
| Rv2739c | Rv2739c | 343A>T | 3 | 23 | 3 | 39 | 0,2673 | 0,0440 | 1,6957 |
| Rv2824c | Rv2824c | 191A>V | 3 | 23 | 3 | 39 | 0,2673 | 0,0440 | 1,6957 |
| Rv2870c | dxr | 81P>L | 3 | 23 | 3 | 39 | 0,2673 | 0,0440 | 1,6957 |
| Rv2930 | fadD26 | 163Q>R | 3 | 23 | 3 | 39 | 0,2673 | 0,0440 | 1,6957 |
| Rv2935 | ppsE | 1435P>S | 3 | 23 | 3 | 39 | 0,2673 | 0,0440 | 1,6957 |
| Rv2958c | Rv2958c | 88P>R | 3 | 23 | 3 | 39 | 0,2673 | 0,0440 | 1,6957 |
| Rv2982c | gpsA | 270G>S | 3 | 23 | 3 | 39 | 0,2673 | 0,0440 | 1,6957 |
| Rv2984 | ppk | 340G>C | 3 | 23 | 3 | 39 | 0,2673 | 0,0440 | 1,6957 |
| Rv2994 | Rv2994 | 318V>F | 3 | 23 | 3 | 39 | 0,2673 | 0,0440 | 1,6957 |
| Rv2997 | Rv2997 | 457A>S | 3 | 23 | 3 | 39 | 0,2673 | 0,0440 | 1,6957 |
| Rv2997 | Rv2997 | 475G>S | 3 | 23 | 3 | 39 | 0,2673 | 0,0440 | 1,6957 |
| Rv3058c | Rv3058c | 35E>K | 3 | 23 | 3 | 39 | 0,2673 | 0,0440 | 1,6957 |
| Rv3074 | Rv3074 | 151A>S | 3 | 23 | 3 | 39 | 0,2673 | 0,0440 | 1,6957 |
| Rv3106 | fprA | 98D>G | 3 | 23 | 3 | 39 | 0,2673 | 0,0440 | 1,6957 |
| Rv3139 | fadE24 | 116I>L | 3 | 23 | 3 | 39 | 0,2673 | 0,0440 | 1,6957 |
| Rv3139 | fadE24 | 130P>L | 3 | 23 | 3 | 39 | 0,2673 | 0,0440 | 1,6957 |
| Rv3140 | fadE23 | 273I>V | 3 | 23 | 3 | 39 | 0,2673 | 0,0440 | 1,6957 |
| Rv3239c | Rv3239c | 44N>S | 3 | 23 | 3 | 39 | 0,2673 | 0,0440 | 1,6957 |
| Rv3252c | alkB | 379W>R | 3 | 23 | 3 | 39 | 0,2673 | 0,0440 | 1,6957 |
| Rv3350c | PPE56 | 203V>G | 3 | 23 | 3 | 39 | 0,2673 | 0,0440 | 1,6957 |
| Rv3355c | Rv3355c | 4R>S | 3 | 23 | 3 | 39 | 0,2673 | 0,0440 | 1,6957 |
| Rv3411c | guaB2 | 529R>H | 3 | 23 | 3 | 39 | 0,2673 | 0,0440 | 1,6957 |
| Rv3466 | Rv3466 | 24D>A | 3 | 23 | 3 | 39 | 0,2673 | 0,0440 | 1,6957 |
| Rv3538 | Rv3538 | 108T>S | 3 | 23 | 3 | 39 | 0,2673 | 0,0440 | 1,6957 |
| Rv3558 | PPE64 | 196N>Y | 3 | 23 | 3 | 39 | 0,2673 | 0,0440 | 1,6957 |
| Rv3580c | cysS | 392I>F | 3 | 23 | 3 | 39 | 0,2673 | 0,0440 | 1,6957 |

| Rv3587c | Rv3587c | 249P>L | 3 | 23 | 3 | 39 | 0,2673 | 0,0440 | 1,6957 |
| --- | --- | --- | --- | --- | --- | --- | --- | --- | --- |
| Rv3673c | Rv3673c | 76N>T | 3 | 23 | 3 | 39 | 0,2673 | 0,0440 | 1,6957 |
| Rv3712 | Rv3712 | 32G>S | 3 | 23 | 3 | 39 | 0,2673 | 0,0440 | 1,6957 |
| Rv3825c | pks2 | 1811M>T | 3 | 23 | 3 | 39 | 0,2673 | 0,0440 | 1,6957 |
| Rv3864 | Rv3864 | 374D>G | 3 | 23 | 3 | 39 | 0,2673 | 0,0440 | 1,6957 |
| Rv3870 | Rv3870 | 103K>T | 3 | 23 | 3 | 39 | 0,2673 | 0,0440 | 1,6957 |
| Rv3871 | Rv3871 | 207D>A | 3 | 23 | 3 | 39 | 0,2673 | 0,0440 | 1,6957 |
| Rv3894c | Rv3894c | 1114V>G | 3 | 23 | 3 | 39 | 0,2673 | 0,0440 | 1,6957 |
| Rv3910 | Rv3910 | 853M>I | 3 | 23 | 3 | 39 | 0,2673 | 0,0440 | 1,6957 |
| Rv2082 | Rv2082 | 638P>R | 25 | 1 | 39 | 3 | 0,2872 | 0,0330 | 1,9231 |
| Rv0515 | Rv0515 | 496H>P | 7 | 19 | 9 | 33 | 0,3019 | 0,0549 | 1,3509 |
| Rv1353c | Rv1353c | 47G>R | 7 | 19 | 9 | 33 | 0,3019 | 0,0549 | 1,3509 |
| Rv0754 | PE_PGRS11 | 485E>D | 2 | 24 | 2 | 40 | 0,3089 | 0,0293 | 1,6667 |
| Rv1030 | kdpB | 308S>F | 2 | 24 | 2 | 40 | 0,3089 | 0,0293 | 1,6667 |
| Rv1945 | Rv1945 | 387T>N | 2 | 24 | 2 | 40 | 0,3089 | 0,0293 | 1,6667 |
| Rv2059 | Rv2059 | 185L>F | 2 | 24 | 2 | 40 | 0,3089 | 0,0293 | 1,6667 |
| Rv2769c | PE27 | 136A>V | 2 | 24 | 2 | 40 | 0,3089 | 0,0293 | 1,6667 |
| Rv2996c | serA1 | 5V>I | 2 | 24 | 2 | 40 | 0,3089 | 0,0293 | 1,6667 |
| Rv3894c | Rv3894c | 650D>G | 2 | 24 | 2 | 40 | 0,3089 | 0,0293 | 1,6667 |
| Rv0747 | PE_PGRS10 | 500A>G | 10 | 16 | 14 | 28 | 0,3336 | 0,0513 | 1,2500 |
| Rv2048c | pks12 | 3649P>A | 15 | 11 | 22 | 20 | 0,3346 | 0,0531 | 1,2397 |
| Rv3327 | Rv3327 | 54W>* | 15 | 11 | 22 | 20 | 0,3346 | 0,0531 | 1,2397 |
| Rv1196 | PPE18 | 30Q>K | 4 | 22 | 5 | 37 | 0,3403 | 0,0348 | 1,3455 |
| Rv1588c | Rv1588c | 56A>T | 4 | 22 | 5 | 37 | 0,3403 | 0,0348 | 1,3455 |
| Rv1588c | Rv1588c | 63A>T | 4 | 22 | 5 | 37 | 0,3403 | 0,0348 | 1,3455 |
| Rv3466 | Rv3466 | 56A>T | 4 | 22 | 5 | 37 | 0,3403 | 0,0348 | 1,3455 |
| Rv3508 | PE_PGRS54 | 1444V>A | 4 | 22 | 5 | 37 | 0,3403 | 0,0348 | 1,3455 |
| Rv3508 | PE_PGRS54 | 1649V>A | 4 | 22 | 5 | 37 | 0,3403 | 0,0348 | 1,3455 |
| Rv1198 | esxL | 33R>S | 6 | 20 | 8 | 34 | 0,3448 | 0,0403 | 1,2750 |
| Rv1872c | lldD2 | 253V>M | 6 | 20 | 8 | 34 | 0,3448 | 0,0403 | 1,2750 |
| Rv1995 | Rv1995 | 158L>P | 6 | 20 | 8 | 34 | 0,3448 | 0,0403 | 1,2750 |
| Rv2543 | lppA | 137I>V | 6 | 20 | 8 | 34 | 0,3448 | 0,0403 | 1,2750 |
| Rv2543 | lppA | 138A>V | 6 | 20 | 8 | 34 | 0,3448 | 0,0403 | 1,2750 |

| Rv2543 | lppA | 139A>T | 6 | 20 | 8 | 34 | 0,3448 | 0,0403 | 1,2750 |
| --- | --- | --- | --- | --- | --- | --- | --- | --- | --- |
| Rv2544 | lppB | 75Q>R | 6 | 20 | 8 | 34 | 0,3448 | 0,0403 | 1,2750 |
| Rv0001 | dnaA | 24P>L | 1 | 25 | 1 | 41 | 0,3641 | 0,0147 | 1,6400 |
| Rv0014c | pknB | 480I>T | 1 | 25 | 1 | 41 | 0,3641 | 0,0147 | 1,6400 |
| Rv0024 | Rv0024 | 23P>L | 1 | 25 | 1 | 41 | 0,3641 | 0,0147 | 1,6400 |
| Rv0026 | Rv0026 | 323S>A | 1 | 25 | 1 | 41 | 0,3641 | 0,0147 | 1,6400 |
| Rv0035 | fadD34 | 192R>H | 1 | 25 | 1 | 41 | 0,3641 | 0,0147 | 1,6400 |
| Rv0062 | celA1 | 245A>G | 1 | 25 | 1 | 41 | 0,3641 | 0,0147 | 1,6400 |
| Rv0063 | Rv0063 | 18L>V | 1 | 25 | 1 | 41 | 0,3641 | 0,0147 | 1,6400 |
| Rv0071 | Rv0071 | 64Q>E | 1 | 25 | 1 | 41 | 0,3641 | 0,0147 | 1,6400 |
| Rv0087 | hycE | 60E>* | 1 | 25 | 1 | 41 | 0,3641 | 0,0147 | 1,6400 |
| Rv0095c | Rv0095c | 94T>A | 1 | 25 | 1 | 41 | 0,3641 | 0,0147 | 1,6400 |
| Rv0163 | Rv0163 | 45T>A | 1 | 25 | 1 | 41 | 0,3641 | 0,0147 | 1,6400 |
| Rv0197 | Rv0197 | 115G>V | 1 | 25 | 1 | 41 | 0,3641 | 0,0147 | 1,6400 |
| Rv0212c | nadR | 322R>L | 1 | 25 | 1 | 41 | 0,3641 | 0,0147 | 1,6400 |
| Rv0227c | Rv0227c | 412H>Y | 1 | 25 | 1 | 41 | 0,3641 | 0,0147 | 1,6400 |
| Rv0363c | fba | 198E>K | 1 | 25 | 1 | 41 | 0,3641 | 0,0147 | 1,6400 |
| Rv0407 | fgd1 | 45R>C | 1 | 25 | 1 | 41 | 0,3641 | 0,0147 | 1,6400 |
| Rv0419 | lpqM | 67S>R | 1 | 25 | 1 | 41 | 0,3641 | 0,0147 | 1,6400 |
| Rv0425c | ctpH | 502L>Q | 1 | 25 | 1 | 41 | 0,3641 | 0,0147 | 1,6400 |
| Rv0442c | PPE10 | 433P>L | 1 | 25 | 1 | 41 | 0,3641 | 0,0147 | 1,6400 |
| Rv0507 | mmpL2 | 656E>A | 1 | 25 | 1 | 41 | 0,3641 | 0,0147 | 1,6400 |
| Rv0533c | PE_PGRS6 | 587P>R | 1 | 25 | 1 | 41 | 0,3641 | 0,0147 | 1,6400 |
| Rv0631c | recC | 497F>L | 1 | 25 | 1 | 41 | 0,3641 | 0,0147 | 1,6400 |
| Rv0667 | rpoB | 435D>F | 1 | 25 | 1 | 41 | 0,3641 | 0,0147 | 1,6400 |
| Rv0668 | rpoC | 483V>A | 1 | 25 | 1 | 41 | 0,3641 | 0,0147 | 1,6400 |
| Rv0687 | fabG | 107N>S | 1 | 25 | 1 | 41 | 0,3641 | 0,0147 | 1,6400 |
| Rv0783c | emrB | 406G>V | 1 | 25 | 1 | 41 | 0,3641 | 0,0147 | 1,6400 |
| Rv0791c | Rv0791c | 104T>M | 1 | 25 | 1 | 41 | 0,3641 | 0,0147 | 1,6400 |
| Rv0796 | Rv0796 | 199G>S | 1 | 25 | 1 | 41 | 0,3641 | 0,0147 | 1,6400 |
| Rv0822c | Rv0822c | 3D>N | 1 | 25 | 1 | 41 | 0,3641 | 0,0147 | 1,6400 |
| Rv0853c | pdc | 297D>N | 1 | 25 | 1 | 41 | 0,3641 | 0,0147 | 1,6400 |
| Rv0870c | Rv0870c | 92M>I | 1 | 25 | 1 | 41 | 0,3641 | 0,0147 | 1,6400 |

| Rv1048c | Rv1048c | 227A>T | 1 | 25 | 1 | 41 | 0,3641 | 0,0147 | 1,6400 |
| --- | --- | --- | --- | --- | --- | --- | --- | --- | --- |
| Rv1187 | rocA | 525I>T | 1 | 25 | 1 | 41 | 0,3641 | 0,0147 | 1,6400 |
| Rv1273c | Rv1273c | 266S>R | 1 | 25 | 1 | 41 | 0,3641 | 0,0147 | 1,6400 |
| Rv1361c | PPE19 | 158T>A | 1 | 25 | 1 | 41 | 0,3641 | 0,0147 | 1,6400 |
| Rv1361c | PPE19 | 159A>T | 1 | 25 | 1 | 41 | 0,3641 | 0,0147 | 1,6400 |
| Rv1364c | Rv1364c | 487H>L | 1 | 25 | 1 | 41 | 0,3641 | 0,0147 | 1,6400 |
| Rv1369c | Rv1369c | 181G>S | 1 | 25 | 1 | 41 | 0,3641 | 0,0147 | 1,6400 |
| Rv1374c | Rv1374c | 106A>T | 1 | 25 | 1 | 41 | 0,3641 | 0,0147 | 1,6400 |
| Rv1391 | dfp | 415G>D | 1 | 25 | 1 | 41 | 0,3641 | 0,0147 | 1,6400 |
| Rv1394c | cyp132 | 111T>A | 1 | 25 | 1 | 41 | 0,3641 | 0,0147 | 1,6400 |
| Rv1450c | PE_PGRS27 | 339A>D | 1 | 25 | 1 | 41 | 0,3641 | 0,0147 | 1,6400 |
| Rv1452c | PE_PGRS28 | 339A>D | 1 | 25 | 1 | 41 | 0,3641 | 0,0147 | 1,6400 |
| Rv1470 | trxA | 45R>W | 1 | 25 | 1 | 41 | 0,3641 | 0,0147 | 1,6400 |
| Rv1506c | Rv1506c | 144S>F | 1 | 25 | 1 | 41 | 0,3641 | 0,0147 | 1,6400 |
| Rv1527c | pks5 | 1230T>A | 1 | 25 | 1 | 41 | 0,3641 | 0,0147 | 1,6400 |
| Rv1587c | Rv1587c | 198N>K | 1 | 25 | 1 | 41 | 0,3641 | 0,0147 | 1,6400 |
| Rv1670 | Rv1670 | 90W>R | 1 | 25 | 1 | 41 | 0,3641 | 0,0147 | 1,6400 |
| Rv1705c | PPE22 | 257I>T | 1 | 25 | 1 | 41 | 0,3641 | 0,0147 | 1,6400 |
| Rv1714 | Rv1714 | 137R>W | 1 | 25 | 1 | 41 | 0,3641 | 0,0147 | 1,6400 |
| Rv1733c | Rv1733c | 33P>S | 1 | 25 | 1 | 41 | 0,3641 | 0,0147 | 1,6400 |
| Rv1737c | narK2 | 94M>T | 1 | 25 | 1 | 41 | 0,3641 | 0,0147 | 1,6400 |
| Rv1749c | Rv1749c | 28G>A | 1 | 25 | 1 | 41 | 0,3641 | 0,0147 | 1,6400 |
| Rv1756c | Rv1756c | 181G>S | 1 | 25 | 1 | 41 | 0,3641 | 0,0147 | 1,6400 |
| Rv1817 | Rv1817 | 123Q>R | 1 | 25 | 1 | 41 | 0,3641 | 0,0147 | 1,6400 |
| Rv1866 | Rv1866 | 707N>H | 1 | 25 | 1 | 41 | 0,3641 | 0,0147 | 1,6400 |
| Rv1881c | lppE | 60I>V | 1 | 25 | 1 | 41 | 0,3641 | 0,0147 | 1,6400 |
| Rv1922 | Rv1922 | 282D>Y | 1 | 25 | 1 | 41 | 0,3641 | 0,0147 | 1,6400 |
| Rv1937 | Rv1937 | 286T>I | 1 | 25 | 1 | 41 | 0,3641 | 0,0147 | 1,6400 |
| Rv1945 | Rv1945 | 243V>L | 1 | 25 | 1 | 41 | 0,3641 | 0,0147 | 1,6400 |
| Rv1963c | mce3R | 104A>T | 1 | 25 | 1 | 41 | 0,3641 | 0,0147 | 1,6400 |
| Rv1979c | Rv1979c | 222A>T | 1 | 25 | 1 | 41 | 0,3641 | 0,0147 | 1,6400 |
| Rv2025c | Rv2025c | 3H>R | 1 | 25 | 1 | 41 | 0,3641 | 0,0147 | 1,6400 |
| Rv2073c | Rv2073c | 214R>C | 1 | 25 | 1 | 41 | 0,3641 | 0,0147 | 1,6400 |

| Rv2077A | Rv2077A | 48S>T | 1 | 25 | 1 | 41 | 0,3641 | 0,0147 | 1,6400 |
| --- | --- | --- | --- | --- | --- | --- | --- | --- | --- |
| Rv2106 | Rv2106 | 199G>S | 1 | 25 | 1 | 41 | 0,3641 | 0,0147 | 1,6400 |
| Rv2129c | Rv2129c | 170E>A | 1 | 25 | 1 | 41 | 0,3641 | 0,0147 | 1,6400 |
| Rv2167c | Rv2167c | 233G>S | 1 | 25 | 1 | 41 | 0,3641 | 0,0147 | 1,6400 |
| Rv2211c | gcvT | 112I>T | 1 | 25 | 1 | 41 | 0,3641 | 0,0147 | 1,6400 |
| Rv2241 | aceE | 161G>S | 1 | 25 | 1 | 41 | 0,3641 | 0,0147 | 1,6400 |
| Rv2264c | Rv2264c | 472A>S | 1 | 25 | 1 | 41 | 0,3641 | 0,0147 | 1,6400 |
| Rv2279 | Rv2279 | 199G>S | 1 | 25 | 1 | 41 | 0,3641 | 0,0147 | 1,6400 |
| Rv2280 | Rv2280 | 308G>R | 1 | 25 | 1 | 41 | 0,3641 | 0,0147 | 1,6400 |
| Rv2308 | Rv2308 | 179R>P | 1 | 25 | 1 | 41 | 0,3641 | 0,0147 | 1,6400 |
| Rv2314c | Rv2314c | 285A>T | 1 | 25 | 1 | 41 | 0,3641 | 0,0147 | 1,6400 |
| Rv2338c | moeW | 85Q>H | 1 | 25 | 1 | 41 | 0,3641 | 0,0147 | 1,6400 |
| Rv2355 | Rv2355 | 199G>S | 1 | 25 | 1 | 41 | 0,3641 | 0,0147 | 1,6400 |
| Rv2381c | mbtD | 661L>F | 1 | 25 | 1 | 41 | 0,3641 | 0,0147 | 1,6400 |
| Rv2391 | nirA | 514G>R | 1 | 25 | 1 | 41 | 0,3641 | 0,0147 | 1,6400 |
| Rv2394 | ggtB | 629G>C | 1 | 25 | 1 | 41 | 0,3641 | 0,0147 | 1,6400 |
| Rv2408 | PE24 | 17M>T | 1 | 25 | 1 | 41 | 0,3641 | 0,0147 | 1,6400 |
| Rv2447c | folC | 49R>Q | 1 | 25 | 1 | 41 | 0,3641 | 0,0147 | 1,6400 |
| Rv2524c | fas | 2699A>S | 1 | 25 | 1 | 41 | 0,3641 | 0,0147 | 1,6400 |
| Rv2542 | Rv2542 | 363T>A | 1 | 25 | 1 | 41 | 0,3641 | 0,0147 | 1,6400 |
| Rv2544 | lppB | 47G>D | 1 | 25 | 1 | 41 | 0,3641 | 0,0147 | 1,6400 |
| Rv2544 | lppB | 59H>N | 1 | 25 | 1 | 41 | 0,3641 | 0,0147 | 1,6400 |
| Rv2565 | Rv2565 | 375G>D | 1 | 25 | 1 | 41 | 0,3641 | 0,0147 | 1,6400 |
| Rv2592c | ruvB | 344E>G | 1 | 25 | 1 | 41 | 0,3641 | 0,0147 | 1,6400 |
| Rv2618 | Rv2618 | 46G>D | 1 | 25 | 1 | 41 | 0,3641 | 0,0147 | 1,6400 |
| Rv2634c | PE_PGRS46 | 274A>T | 1 | 25 | 1 | 41 | 0,3641 | 0,0147 | 1,6400 |
| Rv2649 | Rv2649 | 215G>S | 1 | 25 | 1 | 41 | 0,3641 | 0,0147 | 1,6400 |
| Rv2653c | Rv2653c | 27Q>P | 1 | 25 | 1 | 41 | 0,3641 | 0,0147 | 1,6400 |
| Rv2670c | Rv2670c | 5A>V | 1 | 25 | 1 | 41 | 0,3641 | 0,0147 | 1,6400 |
| Rv2678c | hemE | 153S>A | 1 | 25 | 1 | 41 | 0,3641 | 0,0147 | 1,6400 |
| Rv2684 | arsA | 261V>A | 1 | 25 | 1 | 41 | 0,3641 | 0,0147 | 1,6400 |
| Rv2685 | arsB1 | 389A>T | 1 | 25 | 1 | 41 | 0,3641 | 0,0147 | 1,6400 |
| Rv2702 | ppgK | 140N>S | 1 | 25 | 1 | 41 | 0,3641 | 0,0147 | 1,6400 |

| Rv2733c | Rv2733c | 23P>L | 1 | 25 | 1 | 41 | 0,3641 | 0,0147 | 1,6400 |
| --- | --- | --- | --- | --- | --- | --- | --- | --- | --- |
| Rv2782c | pepR | 196R>W | 1 | 25 | 1 | 41 | 0,3641 | 0,0147 | 1,6400 |
| Rv2797c | Rv2797c | 520M>I | 1 | 25 | 1 | 41 | 0,3641 | 0,0147 | 1,6400 |
| Rv2814c | Rv2814c | 199G>S | 1 | 25 | 1 | 41 | 0,3641 | 0,0147 | 1,6400 |
| Rv2858c | aldC | 21T>A | 1 | 25 | 1 | 41 | 0,3641 | 0,0147 | 1,6400 |
| Rv2871 | Rv2871 | 59L>M | 1 | 25 | 1 | 41 | 0,3641 | 0,0147 | 1,6400 |
| Rv2897c | Rv2897c | 296A>E | 1 | 25 | 1 | 41 | 0,3641 | 0,0147 | 1,6400 |
| Rv2940c | mas | 699T>K | 1 | 25 | 1 | 41 | 0,3641 | 0,0147 | 1,6400 |
| Rv2973c | recG | 374N>S | 1 | 25 | 1 | 41 | 0,3641 | 0,0147 | 1,6400 |
| Rv2994 | Rv2994 | 326T>A | 1 | 25 | 1 | 41 | 0,3641 | 0,0147 | 1,6400 |
| Rv2998 | Rv2998 | 40M>I | 1 | 25 | 1 | 41 | 0,3641 | 0,0147 | 1,6400 |
| Rv3043c | ctaD | 487S>A | 1 | 25 | 1 | 41 | 0,3641 | 0,0147 | 1,6400 |
| Rv3052c | nrdI | 74L>F | 1 | 25 | 1 | 41 | 0,3641 | 0,0147 | 1,6400 |
| Rv3067 | Rv3067 | 36A>T | 1 | 25 | 1 | 41 | 0,3641 | 0,0147 | 1,6400 |
| Rv3124 | Rv3124 | 274E>D | 1 | 25 | 1 | 41 | 0,3641 | 0,0147 | 1,6400 |
| Rv3170 | aofH | 337I>V | 1 | 25 | 1 | 41 | 0,3641 | 0,0147 | 1,6400 |
| Rv3185 | Rv3185 | 199G>S | 1 | 25 | 1 | 41 | 0,3641 | 0,0147 | 1,6400 |
| Rv3187 | Rv3187 | 199G>S | 1 | 25 | 1 | 41 | 0,3641 | 0,0147 | 1,6400 |
| Rv3287c | rsbW | 125G>A | 1 | 25 | 1 | 41 | 0,3641 | 0,0147 | 1,6400 |
| Rv3326 | Rv3326 | 199G>S | 1 | 25 | 1 | 41 | 0,3641 | 0,0147 | 1,6400 |
| Rv3344c | PE_PGRS49 | 215I>M | 1 | 25 | 1 | 41 | 0,3641 | 0,0147 | 1,6400 |
| Rv3350c | PPE56 | 84E>V | 1 | 25 | 1 | 41 | 0,3641 | 0,0147 | 1,6400 |
| Rv3380c | Rv3380c | 181G>S | 1 | 25 | 1 | 41 | 0,3641 | 0,0147 | 1,6400 |
| Rv3395A | Rv3395A | 21G>C | 1 | 25 | 1 | 41 | 0,3641 | 0,0147 | 1,6400 |
| Rv3403c | Rv3403c | 23S>R | 1 | 25 | 1 | 41 | 0,3641 | 0,0147 | 1,6400 |
| Rv3463 | Rv3463 | 196V>M | 1 | 25 | 1 | 41 | 0,3641 | 0,0147 | 1,6400 |
| Rv3475 | Rv3475 | 233G>S | 1 | 25 | 1 | 41 | 0,3641 | 0,0147 | 1,6400 |
| Rv3507 | PE_PGRS53 | 560A>G | 1 | 25 | 1 | 41 | 0,3641 | 0,0147 | 1,6400 |
| Rv3694c | Rv3694c | 21I>V | 1 | 25 | 1 | 41 | 0,3641 | 0,0147 | 1,6400 |
| Rv3696c | glpK | 416S>Y | 1 | 25 | 1 | 41 | 0,3641 | 0,0147 | 1,6400 |
| Rv3710 | leuA | 391K>E | 1 | 25 | 1 | 41 | 0,3641 | 0,0147 | 1,6400 |
| Rv3727 | Rv3727 | 287D>Y | 1 | 25 | 1 | 41 | 0,3641 | 0,0147 | 1,6400 |
| Rv3741c | Rv3741c | 89A>V | 1 | 25 | 1 | 41 | 0,3641 | 0,0147 | 1,6400 |

| Rv3746c | PE34 | 95A>T | 1 | 25 | 1 | 41 | 0,3641 | 0,0147 | 1,6400 |
| --- | --- | --- | --- | --- | --- | --- | --- | --- | --- |
| Rv3786c | Rv3786c | 178G>A | 1 | 25 | 1 | 41 | 0,3641 | 0,0147 | 1,6400 |
| Rv3786c | Rv3786c | 245D>E | 1 | 25 | 1 | 41 | 0,3641 | 0,0147 | 1,6400 |
| Rv3795 | embB | 406G>S | 1 | 25 | 1 | 41 | 0,3641 | 0,0147 | 1,6400 |
| Rv3796 | Rv3796 | 237A>S | 1 | 25 | 1 | 41 | 0,3641 | 0,0147 | 1,6400 |
| Rv3804c | fbpA | 158V>F | 1 | 25 | 1 | 41 | 0,3641 | 0,0147 | 1,6400 |
| Rv3805c | Rv3805c | 322Q>E | 1 | 25 | 1 | 41 | 0,3641 | 0,0147 | 1,6400 |
| Rv3811 | Rv3811 | 197A>P | 1 | 25 | 1 | 41 | 0,3641 | 0,0147 | 1,6400 |
| Rv3825c | pks2 | 1762P>S | 1 | 25 | 1 | 41 | 0,3641 | 0,0147 | 1,6400 |
| Rv3825c | pks2 | 586P>S | 1 | 25 | 1 | 41 | 0,3641 | 0,0147 | 1,6400 |
| Rv3887c | Rv3887c | 386P>T | 1 | 25 | 1 | 41 | 0,3641 | 0,0147 | 1,6400 |
| Rv3911 | sigM | 133R>Q | 1 | 25 | 1 | 41 | 0,3641 | 0,0147 | 1,6400 |
| Rv3919c | gidB | 50L>R | 1 | 25 | 1 | 41 | 0,3641 | 0,0147 | 1,6400 |
| Rv3919c | gidB | 73G>A | 1 | 25 | 1 | 41 | 0,3641 | 0,0147 | 1,6400 |
| Rv3922c | Rv3922c | 99D>H | 1 | 25 | 1 | 41 | 0,3641 | 0,0147 | 1,6400 |
| Rv3892c | PPE69 | 19T>K | 20 | 6 | 31 | 11 | 0,3866 | 0,0311 | 1,1828 |
| Rv0376c | Rv0376c | 69Y>C | 7 | 19 | 10 | 32 | 0,3866 | 0,0311 | 1,1789 |
| Rv1577c | Rv1577c | 52L>V | 7 | 19 | 10 | 32 | 0,3866 | 0,0311 | 1,1789 |
| Rv0751c | mmsB | 290A>T | 5 | 21 | 7 | 35 | 0,3938 | 0,0256 | 1,1905 |
| Rv1552 | frdA | 365G>S | 5 | 21 | 7 | 35 | 0,3938 | 0,0256 | 1,1905 |
| Rv1762c | Rv1762c | 180Q>* | 5 | 21 | 7 | 35 | 0,3938 | 0,0256 | 1,1905 |
| Rv2082 | Rv2082 | 466M>L | 5 | 21 | 7 | 35 | 0,3938 | 0,0256 | 1,1905 |
| Rv2476c | gdh | 1481S>L | 5 | 21 | 7 | 35 | 0,3938 | 0,0256 | 1,1905 |
| Rv3466 | Rv3466 | 63A>T | 5 | 21 | 7 | 35 | 0,3938 | 0,0256 | 1,1905 |
| Rv3863 | Rv3863 | 130P>S | 5 | 21 | 7 | 35 | 0,3938 | 0,0256 | 1,1905 |
| Rv3619c | esxV | 23S>L | 3 | 23 | 4 | 38 | 0,3952 | 0,0201 | 1,2391 |
| Rv0233 | nrdB | 33H>D | 25 | 1 | 40 | 2 | 0,4291 | 0,0092 | 1,2500 |
| Rv0006 | gyrA | 247G>S | 6 | 20 | 9 | 33 | 0,4367 | 0,0165 | 1,1000 |
| Rv0035 | fadD34 | 169D>H | 6 | 20 | 9 | 33 | 0,4367 | 0,0165 | 1,1000 |
| Rv0064 | Rv0064 | 769L>F | 6 | 20 | 9 | 33 | 0,4367 | 0,0165 | 1,1000 |
| Rv0271c | fadE6 | 189V>I | 6 | 20 | 9 | 33 | 0,4367 | 0,0165 | 1,1000 |
| Rv0319 | pcp | 69G>D | 6 | 20 | 9 | 33 | 0,4367 | 0,0165 | 1,1000 |
| Rv0338c | Rv0338c | 641R>H | 6 | 20 | 9 | 33 | 0,4367 | 0,0165 | 1,1000 |

| Rv0400c | fadE7 | 41T>N | 6 | 20 | 9 | 33 | 0,4367 | 0,0165 | 1,1000 |
| --- | --- | --- | --- | --- | --- | --- | --- | --- | --- |
| Rv0415 | thiO | 130V>F | 6 | 20 | 9 | 33 | 0,4367 | 0,0165 | 1,1000 |
| Rv0717 | rpsN | 61W>* | 6 | 20 | 9 | 33 | 0,4367 | 0,0165 | 1,1000 |
| Rv0770 | Rv0770 | 184F>L | 6 | 20 | 9 | 33 | 0,4367 | 0,0165 | 1,1000 |
| Rv0825c | Rv0825c | 54T>R | 6 | 20 | 9 | 33 | 0,4367 | 0,0165 | 1,1000 |
| Rv0890c | Rv0890c | 361G>D | 6 | 20 | 9 | 33 | 0,4367 | 0,0165 | 1,1000 |
| Rv0974c | accD2 | 23K>E | 6 | 20 | 9 | 33 | 0,4367 | 0,0165 | 1,1000 |
| Rv1023 | eno | 34R>Q | 6 | 20 | 9 | 33 | 0,4367 | 0,0165 | 1,1000 |
| Rv1039c | PPE15 | 67A>T | 6 | 20 | 9 | 33 | 0,4367 | 0,0165 | 1,1000 |
| Rv1056 | Rv1056 | 123Y>H | 6 | 20 | 9 | 33 | 0,4367 | 0,0165 | 1,1000 |
| Rv1184c | Rv1184c | 296M>I | 6 | 20 | 9 | 33 | 0,4367 | 0,0165 | 1,1000 |
| Rv1551 | plsB1 | 25M>I | 6 | 20 | 9 | 33 | 0,4367 | 0,0165 | 1,1000 |
| Rv1557 | mmpL6 | 291A>V | 6 | 20 | 9 | 33 | 0,4367 | 0,0165 | 1,1000 |
| Rv1595 | nadB | 408T>I | 6 | 20 | 9 | 33 | 0,4367 | 0,0165 | 1,1000 |
| Rv1615 | Rv1615 | 118A>T | 6 | 20 | 9 | 33 | 0,4367 | 0,0165 | 1,1000 |
| Rv1668c | Rv1668c | 57D>N | 6 | 20 | 9 | 33 | 0,4367 | 0,0165 | 1,1000 |
| Rv1825 | Rv1825 | 181P>S | 6 | 20 | 9 | 33 | 0,4367 | 0,0165 | 1,1000 |
| Rv1878 | glnA3 | 296S>F | 6 | 20 | 9 | 33 | 0,4367 | 0,0165 | 1,1000 |
| Rv1897c | Rv1897c | 89P>L | 6 | 20 | 9 | 33 | 0,4367 | 0,0165 | 1,1000 |
| Rv2059 | Rv2059 | 166V>I | 6 | 20 | 9 | 33 | 0,4367 | 0,0165 | 1,1000 |
| Rv2082 | Rv2082 | 96A>T | 6 | 20 | 9 | 33 | 0,4367 | 0,0165 | 1,1000 |
| Rv2176 | pknL | 11E>K | 6 | 20 | 9 | 33 | 0,4367 | 0,0165 | 1,1000 |
| Rv2245 | kasA | 269G>S | 6 | 20 | 9 | 33 | 0,4367 | 0,0165 | 1,1000 |
| Rv2687c | Rv2687c | 220R>W | 6 | 20 | 9 | 33 | 0,4367 | 0,0165 | 1,1000 |
| Rv2748c | ftsK | 298M>V | 6 | 20 | 9 | 33 | 0,4367 | 0,0165 | 1,1000 |
| Rv2911 | dacB2 | 2R>Q | 6 | 20 | 9 | 33 | 0,4367 | 0,0165 | 1,1000 |
| Rv2933 | ppsC | 864S>R | 6 | 20 | 9 | 33 | 0,4367 | 0,0165 | 1,1000 |
| Rv2962c | Rv2962c | 165W>* | 6 | 20 | 9 | 33 | 0,4367 | 0,0165 | 1,1000 |
| Rv2994 | Rv2994 | 220S>P | 6 | 20 | 9 | 33 | 0,4367 | 0,0165 | 1,1000 |
| Rv3088 | Rv3088 | 216A>E | 6 | 20 | 9 | 33 | 0,4367 | 0,0165 | 1,1000 |
| Rv3174 | Rv3174 | 42L>R | 6 | 20 | 9 | 33 | 0,4367 | 0,0165 | 1,1000 |
| Rv3195 | Rv3195 | 13G>S | 6 | 20 | 9 | 33 | 0,4367 | 0,0165 | 1,1000 |
| Rv3263 | Rv3263 | 152G>S | 6 | 20 | 9 | 33 | 0,4367 | 0,0165 | 1,1000 |

| Rv3430c | PPE59 | 174P>P | 6 | 20 | 9 | 33 | 0,4367 | 0,0165 | 1,1000 |
| --- | --- | --- | --- | --- | --- | --- | --- | --- | --- |
| Rv3535c | Rv3535c | 183G>R | 6 | 20 | 9 | 33 | 0,4367 | 0,0165 | 1,1000 |
| Rv3843c | Rv3843c | 184V>A | 6 | 20 | 9 | 33 | 0,4367 | 0,0165 | 1,1000 |
| Rv3882c | Rv3882c | 118I>V | 6 | 20 | 9 | 33 | 0,4367 | 0,0165 | 1,1000 |
| Rv3900c | Rv3900c | 260P>R | 6 | 20 | 9 | 33 | 0,4367 | 0,0165 | 1,1000 |
| Rv2037c | Rv2037c | 231I>T | 11 | 15 | 17 | 25 | 0,4407 | 0,0183 | 1,0784 |
| Rv0578c | PE_PGRS7 | 82A>T | 4 | 22 | 6 | 36 | 0,4505 | 0,0110 | 1,0909 |
| Rv3159c | PPE53 | 33R>G | 9 | 17 | 14 | 28 | 0,4568 | 0,0128 | 1,0588 |
| Rv0050 | ponA1 | 516A>T | 2 | 24 | 3 | 39 | 0,4664 | 0,0055 | 1,0833 |
| Rv0062 | celA1 | 270G>E | 2 | 24 | 3 | 39 | 0,4664 | 0,0055 | 1,0833 |
| Rv0154c | fadE2 | 326R>H | 2 | 24 | 3 | 39 | 0,4664 | 0,0055 | 1,0833 |
| Rv0244c | fadE5 | 350D>N | 2 | 24 | 3 | 39 | 0,4664 | 0,0055 | 1,0833 |
| Rv0276 | Rv0276 | 134E>G | 2 | 24 | 3 | 39 | 0,4664 | 0,0055 | 1,0833 |
| Rv0277c | Rv0277c | 74N>T | 2 | 24 | 3 | 39 | 0,4664 | 0,0055 | 1,0833 |
| Rv0495c | Rv0495c | 34A>V | 2 | 24 | 3 | 39 | 0,4664 | 0,0055 | 1,0833 |
| Rv0682 | rpsL | 43K>R | 2 | 24 | 3 | 39 | 0,4664 | 0,0055 | 1,0833 |
| Rv0813c | Rv0813c | 68L>P | 2 | 24 | 3 | 39 | 0,4664 | 0,0055 | 1,0833 |
| Rv0889c | citA | 48T>N | 2 | 24 | 3 | 39 | 0,4664 | 0,0055 | 1,0833 |
| Rv0922 | Rv0922 | 522G>D | 2 | 24 | 3 | 39 | 0,4664 | 0,0055 | 1,0833 |
| Rv0957 | purH | 416F>V | 2 | 24 | 3 | 39 | 0,4664 | 0,0055 | 1,0833 |
| Rv1196 | PPE18 | 384M>I | 2 | 24 | 3 | 39 | 0,4664 | 0,0055 | 1,0833 |
| Rv1280c | oppA | 546E>K | 2 | 24 | 3 | 39 | 0,4664 | 0,0055 | 1,0833 |
| Rv1360 | Rv1360 | 317D>H | 2 | 24 | 3 | 39 | 0,4664 | 0,0055 | 1,0833 |
| Rv1434 | Rv1434 | 12A>T | 2 | 24 | 3 | 39 | 0,4664 | 0,0055 | 1,0833 |
| Rv1452c | PE_PGRS28 | 438A>T | 2 | 24 | 3 | 39 | 0,4664 | 0,0055 | 1,0833 |
| Rv1523 | Rv1523 | 10L>S | 2 | 24 | 3 | 39 | 0,4664 | 0,0055 | 1,0833 |
| Rv1709 | Rv1709 | 239L>V | 2 | 24 | 3 | 39 | 0,4664 | 0,0055 | 1,0833 |
| Rv1718 | Rv1718 | 107P>R | 2 | 24 | 3 | 39 | 0,4664 | 0,0055 | 1,0833 |
| Rv1785c | cyp143 | 192T>R | 2 | 24 | 3 | 39 | 0,4664 | 0,0055 | 1,0833 |
| Rv1787 | PPE25 | 236F>L | 2 | 24 | 3 | 39 | 0,4664 | 0,0055 | 1,0833 |
| Rv1802 | PPE30 | 270F>V | 2 | 24 | 3 | 39 | 0,4664 | 0,0055 | 1,0833 |
| Rv1888c | Rv1888c | 180P>S | 2 | 24 | 3 | 39 | 0,4664 | 0,0055 | 1,0833 |
| Rv1916 | aceAb | 206S>G | 2 | 24 | 3 | 39 | 0,4664 | 0,0055 | 1,0833 |

| Rv1969 | mce3D | 7R>G | 2 | 24 | 3 | 39 | 0,4664 | 0,0055 | 1,0833 |
| --- | --- | --- | --- | --- | --- | --- | --- | --- | --- |
| Rv2030c | Rv2030c | 14P>L | 2 | 24 | 3 | 39 | 0,4664 | 0,0055 | 1,0833 |
| Rv2213 | pepB | 101W>L | 2 | 24 | 3 | 39 | 0,4664 | 0,0055 | 1,0833 |
| Rv2214c | ephD | 515T>I | 2 | 24 | 3 | 39 | 0,4664 | 0,0055 | 1,0833 |
| Rv2285 | Rv2285 | 315L>P | 2 | 24 | 3 | 39 | 0,4664 | 0,0055 | 1,0833 |
| Rv2351c | plcA | 446T>A | 2 | 24 | 3 | 39 | 0,4664 | 0,0055 | 1,0833 |
| Rv2477c | Rv2477c | 41G>E | 2 | 24 | 3 | 39 | 0,4664 | 0,0055 | 1,0833 |
| Rv2786c | ribF | 17I>S | 2 | 24 | 3 | 39 | 0,4664 | 0,0055 | 1,0833 |
| Rv2802c | Rv2802c | 121E>A | 2 | 24 | 3 | 39 | 0,4664 | 0,0055 | 1,0833 |
| Rv2877c | Rv2877c | 96L>F | 2 | 24 | 3 | 39 | 0,4664 | 0,0055 | 1,0833 |
| Rv2931 | ppsA | 862V>I | 2 | 24 | 3 | 39 | 0,4664 | 0,0055 | 1,0833 |
| Rv3089 | fadD13 | 179E>K | 2 | 24 | 3 | 39 | 0,4664 | 0,0055 | 1,0833 |
| Rv3169 | Rv3169 | 171W>R | 2 | 24 | 3 | 39 | 0,4664 | 0,0055 | 1,0833 |
| Rv3299c | atsB | 625I>M | 2 | 24 | 3 | 39 | 0,4664 | 0,0055 | 1,0833 |
| Rv3300c | Rv3300c | 232R>P | 2 | 24 | 3 | 39 | 0,4664 | 0,0055 | 1,0833 |
| Rv3476c | kgtP | 377L>F | 2 | 24 | 3 | 39 | 0,4664 | 0,0055 | 1,0833 |
| Rv3489 | Rv3489 | 6D>N | 2 | 24 | 3 | 39 | 0,4664 | 0,0055 | 1,0833 |
| Rv3714c | Rv3714c | 109I>V | 2 | 24 | 3 | 39 | 0,4664 | 0,0055 | 1,0833 |
| Rv3737 | Rv3737 | 320V>L | 2 | 24 | 3 | 39 | 0,4664 | 0,0055 | 1,0833 |
| Rv3864 | Rv3864 | 235S>P | 2 | 24 | 3 | 39 | 0,4664 | 0,0055 | 1,0833 |
| Rv3901c | Rv3901c | 68T>I | 2 | 24 | 3 | 39 | 0,4664 | 0,0055 | 1,0833 |
| Rv3916c | Rv3916c | 71R>G | 2 | 24 | 3 | 39 | 0,4664 | 0,0055 | 1,0833 |
| Rv3919c | gidB | 77V>G | 2 | 24 | 3 | 39 | 0,4664 | 0,0055 | 1,0833 |
| Rv0064 | Rv0064 | 418L>F | 7 | 19 | 11 | 31 | 0,4735 | 0,0073 | 1,0383 |
| Rv1041c | Rv1041c | 209Q>H | 7 | 19 | 11 | 31 | 0,4735 | 0,0073 | 1,0383 |
| Rv1787 | PPE25 | 283A>V | 7 | 19 | 11 | 31 | 0,4735 | 0,0073 | 1,0383 |
| Rv3063 | cstA | 378H>D | 7 | 19 | 11 | 31 | 0,4735 | 0,0073 | 1,0383 |
| Rv3190c | Rv3190c | 27S>R | 7 | 19 | 11 | 31 | 0,4735 | 0,0073 | 1,0383 |
| Rv0008c | Rv0008c | 98R>C | 5 | 21 | 8 | 34 | 0,4926 | 0,0018 | 1,0119 |
| Rv0143c | Rv0143c | 182F>L | 5 | 21 | 8 | 34 | 0,4926 | 0,0018 | 1,0119 |
| Rv0158 | Rv0158 | 148G>S | 5 | 21 | 8 | 34 | 0,4926 | 0,0018 | 1,0119 |
| Rv0371c | Rv0371c | 123A>V | 5 | 21 | 8 | 34 | 0,4926 | 0,0018 | 1,0119 |
| Rv0750 | Rv0750 | 27L>V | 5 | 21 | 8 | 34 | 0,4926 | 0,0018 | 1,0119 |

| Rv0938 | Rv0938 | 208P>S | 5 | 21 | 8 | 34 | 0,4926 | 0,0018 | 1,0119 |
| --- | --- | --- | --- | --- | --- | --- | --- | --- | --- |
| Rv1384 | carB | 483V>A | 5 | 21 | 8 | 34 | 0,4926 | 0,0018 | 1,0119 |
| Rv1384 | carB | 922H>D | 5 | 21 | 8 | 34 | 0,4926 | 0,0018 | 1,0119 |
| Rv1709 | Rv1709 | 216W>S | 5 | 21 | 8 | 34 | 0,4926 | 0,0018 | 1,0119 |
| Rv2300c | Rv2300c | 47K>Q | 5 | 21 | 8 | 34 | 0,4926 | 0,0018 | 1,0119 |
| Rv2347c | esxP | 3T>S | 5 | 21 | 8 | 34 | 0,4926 | 0,0018 | 1,0119 |
| Rv2578c | Rv2578c | 128T>A | 5 | 21 | 8 | 34 | 0,4926 | 0,0018 | 1,0119 |
| Rv2731 | Rv2731 | 40S>P | 5 | 21 | 8 | 34 | 0,4926 | 0,0018 | 1,0119 |
| Rv3097c | lipY | 197G>A | 5 | 21 | 8 | 34 | 0,4926 | 0,0018 | 1,0119 |
| Rv3416 | whiB3 | 20Q>R | 5 | 21 | 8 | 34 | 0,4926 | 0,0018 | 1,0119 |
| Rv1760 | Rv1760 | 219E>V | 18 | 8 | 29 | 13 | 0,4937 | 0,0018 | 1,0086 |
| Rv2048c | pks12 | 2147H>Q | 13 | 13 | 21 | 21 | 0,5000 | 0,0000 | 1,0000 |
| Rv0095c | Rv0095c | 126E>D | 3 | 23 | 5 | 37 | 0,5182 | -0,0037 | 0,9652 |
| Rv0096 | PPE1 | 159T>M | 6 | 20 | 10 | 32 | 0,5276 | -0,0073 | 0,9600 |
| Rv0355c | PPE8 | 1947G>D | 6 | 20 | 10 | 32 | 0,5276 | -0,0073 | 0,9600 |
| Rv0359 | Rv0359 | 214L>R | 6 | 20 | 10 | 32 | 0,5276 | -0,0073 | 0,9600 |
| Rv0380c | Rv0380c | 180G>D | 6 | 20 | 10 | 32 | 0,5276 | -0,0073 | 0,9600 |
| Rv0486 | Rv0486 | 111N>S | 6 | 20 | 10 | 32 | 0,5276 | -0,0073 | 0,9600 |
| Rv0557 | pimB | 152R>P | 6 | 20 | 10 | 32 | 0,5276 | -0,0073 | 0,9600 |
| Rv0808 | purF | 508A>V | 6 | 20 | 10 | 32 | 0,5276 | -0,0073 | 0,9600 |
| Rv0816c | thiX | 104L>V | 6 | 20 | 10 | 32 | 0,5276 | -0,0073 | 0,9600 |
| Rv0914c | Rv0914c | 112Y>H | 6 | 20 | 10 | 32 | 0,5276 | -0,0073 | 0,9600 |
| Rv1108c | xseA | 400A>G | 6 | 20 | 10 | 32 | 0,5276 | -0,0073 | 0,9600 |
| Rv1194c | Rv1194c | 114F>L | 6 | 20 | 10 | 32 | 0,5276 | -0,0073 | 0,9600 |
| Rv1316c | ogt | 15T>S | 6 | 20 | 10 | 32 | 0,5276 | -0,0073 | 0,9600 |
| Rv1358 | Rv1358 | 1114G>E | 6 | 20 | 10 | 32 | 0,5276 | -0,0073 | 0,9600 |
| Rv1361c | PPE19 | 269F>L | 6 | 20 | 10 | 32 | 0,5276 | -0,0073 | 0,9600 |
| Rv1361c | PPE19 | 286Q>W | 6 | 20 | 10 | 32 | 0,5276 | -0,0073 | 0,9600 |
| Rv1386 | PE15 | 51A>T | 6 | 20 | 10 | 32 | 0,5276 | -0,0073 | 0,9600 |
| Rv1461 | Rv1461 | 544M>I | 6 | 20 | 10 | 32 | 0,5276 | -0,0073 | 0,9600 |
| Rv1497 | lipL | 237A>P | 6 | 20 | 10 | 32 | 0,5276 | -0,0073 | 0,9600 |
| Rv1505c | Rv1505c | 195G>R | 6 | 20 | 10 | 32 | 0,5276 | -0,0073 | 0,9600 |
| Rv1521 | fadD25 | 175T>A | 6 | 20 | 10 | 32 | 0,5276 | -0,0073 | 0,9600 |

| Rv1650 | pheT | 683G>C | 6 | 20 | 10 | 32 | 0,5276 | -0,0073 | 0,9600 |
| --- | --- | --- | --- | --- | --- | --- | --- | --- | --- |
| Rv1652 | argC | 134P>L | 6 | 20 | 10 | 32 | 0,5276 | -0,0073 | 0,9600 |
| Rv1811 | mgtC | 182R>H | 6 | 20 | 10 | 32 | 0,5276 | -0,0073 | 0,9600 |
| Rv2048c | pks12 | 973E>D | 6 | 20 | 10 | 32 | 0,5276 | -0,0073 | 0,9600 |
| Rv2482c | plsB2 | 329D>G | 6 | 20 | 10 | 32 | 0,5276 | -0,0073 | 0,9600 |
| Rv2573 | Rv2573 | 128P>S | 6 | 20 | 10 | 32 | 0,5276 | -0,0073 | 0,9600 |
| Rv2694c | Rv2694c | 26L>V | 6 | 20 | 10 | 32 | 0,5276 | -0,0073 | 0,9600 |
| Rv2697c | dut | 122V>L | 6 | 20 | 10 | 32 | 0,5276 | -0,0073 | 0,9600 |
| Rv2984 | ppk | 159V>F | 6 | 20 | 10 | 32 | 0,5276 | -0,0073 | 0,9600 |
| Rv3057c | Rv3057c | 275V>A | 6 | 20 | 10 | 32 | 0,5276 | -0,0073 | 0,9600 |
| Rv3084 | lipR | 156A>V | 6 | 20 | 10 | 32 | 0,5276 | -0,0073 | 0,9600 |
| Rv3125c | PPE49 | 183Q>* | 6 | 20 | 10 | 32 | 0,5276 | -0,0073 | 0,9600 |
| Rv3200c | Rv3200c | 194V>F | 6 | 20 | 10 | 32 | 0,5276 | -0,0073 | 0,9600 |
| Rv3306c | amiB1 | 39H>Y | 6 | 20 | 10 | 32 | 0,5276 | -0,0073 | 0,9600 |
| Rv3347c | PPE55 | 2119S>T | 6 | 20 | 10 | 32 | 0,5276 | -0,0073 | 0,9600 |
| Rv3347c | PPE55 | 2132F>Y | 6 | 20 | 10 | 32 | 0,5276 | -0,0073 | 0,9600 |
| Rv3347c | PPE55 | 2151G>S | 6 | 20 | 10 | 32 | 0,5276 | -0,0073 | 0,9600 |
| Rv3347c | PPE55 | 643L>R | 6 | 20 | 10 | 32 | 0,5276 | -0,0073 | 0,9600 |
| Rv3529c | Rv3529c | 76Q>* | 6 | 20 | 10 | 32 | 0,5276 | -0,0073 | 0,9600 |
| Rv3533c | PPE62 | 342S>R | 6 | 20 | 10 | 32 | 0,5276 | -0,0073 | 0,9600 |
| Rv3534c | Rv3534c | 147E>D | 6 | 20 | 10 | 32 | 0,5276 | -0,0073 | 0,9600 |
| Rv3545c | cyp125 | 3W>G | 6 | 20 | 10 | 32 | 0,5276 | -0,0073 | 0,9600 |
| Rv3657c | Rv3657c | 130G>R | 6 | 20 | 10 | 32 | 0,5276 | -0,0073 | 0,9600 |
| Rv3729 | Rv3729 | 142V>A | 6 | 20 | 10 | 32 | 0,5276 | -0,0073 | 0,9600 |
| Rv3729 | Rv3729 | 24R>C | 6 | 20 | 10 | 32 | 0,5276 | -0,0073 | 0,9600 |
| Rv3792 | Rv3792 | 456A>V | 6 | 20 | 10 | 32 | 0,5276 | -0,0073 | 0,9600 |
| Rv3873 | PPE68 | 29A>V | 6 | 20 | 10 | 32 | 0,5276 | -0,0073 | 0,9600 |
| Rv3874 | esxB | 68E>K | 6 | 20 | 10 | 32 | 0,5276 | -0,0073 | 0,9600 |
| Rv3882c | Rv3882c | 205V>A | 6 | 20 | 10 | 32 | 0,5276 | -0,0073 | 0,9600 |
| Rv3884c | Rv3884c | 460Q>* | 6 | 20 | 10 | 32 | 0,5276 | -0,0073 | 0,9600 |
| Rv2101 | helZ | 462M>L | 14 | 12 | 23 | 19 | 0,5294 | -0,0092 | 0,9638 |
| Rv2101 | helZ | 601P>Q | 14 | 12 | 23 | 19 | 0,5294 | -0,0092 | 0,9638 |
| Rv2769c | PE27 | 270V>M | 9 | 17 | 15 | 27 | 0,5367 | -0,0110 | 0,9529 |

| Rv2828c | Rv2828c | 141T>R | 4 | 22 | 7 | 35 | 0,5555 | -0,0128 | 0,9091 |
| --- | --- | --- | --- | --- | --- | --- | --- | --- | --- |
| Rv0026 | Rv0026 | 408P>S | 7 | 19 | 12 | 30 | 0,5585 | -0,0165 | 0,9211 |
| Rv0048c | Rv0048c | 248E>D | 7 | 19 | 12 | 30 | 0,5585 | -0,0165 | 0,9211 |
| Rv0151c | PE1 | 122A>T | 7 | 19 | 12 | 30 | 0,5585 | -0,0165 | 0,9211 |
| Rv0151c | PE1 | 572I>V | 7 | 19 | 12 | 30 | 0,5585 | -0,0165 | 0,9211 |
| Rv0331 | Rv0331 | 359A>P | 7 | 19 | 12 | 30 | 0,5585 | -0,0165 | 0,9211 |
| Rv0405 | pks6 | 1115L>V | 7 | 19 | 12 | 30 | 0,5585 | -0,0165 | 0,9211 |
| Rv0453 | PPE11 | 430R>C | 7 | 19 | 12 | 30 | 0,5585 | -0,0165 | 0,9211 |
| Rv0565c | Rv0565c | 68S>P | 7 | 19 | 12 | 30 | 0,5585 | -0,0165 | 0,9211 |
| Rv0588 | yrbE2B | 243T>I | 7 | 19 | 12 | 30 | 0,5585 | -0,0165 | 0,9211 |
| Rv0668 | rpoC | 594G>E | 7 | 19 | 12 | 30 | 0,5585 | -0,0165 | 0,9211 |
| Rv0688 | Rv0688 | 107G>V | 7 | 19 | 12 | 30 | 0,5585 | -0,0165 | 0,9211 |
| Rv0803 | purL | 464E>Q | 7 | 19 | 12 | 30 | 0,5585 | -0,0165 | 0,9211 |
| Rv0885 | Rv0885 | 242S>A | 7 | 19 | 12 | 30 | 0,5585 | -0,0165 | 0,9211 |
| Rv1087 | PE_PGRS21 | 173G>S | 7 | 19 | 12 | 30 | 0,5585 | -0,0165 | 0,9211 |
| Rv1162 | narH | 467A>V | 7 | 19 | 12 | 30 | 0,5585 | -0,0165 | 0,9211 |
| Rv1290c | Rv1290c | 282L>F | 7 | 19 | 12 | 30 | 0,5585 | -0,0165 | 0,9211 |
| Rv1328 | glgP | 255K>E | 7 | 19 | 12 | 30 | 0,5585 | -0,0165 | 0,9211 |
| Rv1479 | moxR1 | 26H>N | 7 | 19 | 12 | 30 | 0,5585 | -0,0165 | 0,9211 |
| Rv1759c | wag22 | 553F>L | 7 | 19 | 12 | 30 | 0,5585 | -0,0165 | 0,9211 |
| Rv1773c | Rv1773c | 89H>Y | 7 | 19 | 12 | 30 | 0,5585 | -0,0165 | 0,9211 |
| Rv1800 | PPE28 | 150A>V | 7 | 19 | 12 | 30 | 0,5585 | -0,0165 | 0,9211 |
| Rv1957 | Rv1957 | 84D>N | 7 | 19 | 12 | 30 | 0,5585 | -0,0165 | 0,9211 |
| Rv1982c | Rv1982c | 126L>H | 7 | 19 | 12 | 30 | 0,5585 | -0,0165 | 0,9211 |
| Rv2002 | fabG3 | 174S>G | 7 | 19 | 12 | 30 | 0,5585 | -0,0165 | 0,9211 |
| Rv2016 | Rv2016 | 112P>L | 7 | 19 | 12 | 30 | 0,5585 | -0,0165 | 0,9211 |
| Rv2067c | Rv2067c | 288E>K | 7 | 19 | 12 | 30 | 0,5585 | -0,0165 | 0,9211 |
| Rv2155c | murD | 83T>I | 7 | 19 | 12 | 30 | 0,5585 | -0,0165 | 0,9211 |
| Rv2402 | Rv2402 | 609V>M | 7 | 19 | 12 | 30 | 0,5585 | -0,0165 | 0,9211 |
| Rv2458 | mmuM | 155E>K | 7 | 19 | 12 | 30 | 0,5585 | -0,0165 | 0,9211 |
| Rv2482c | plsB2 | 180R>Q | 7 | 19 | 12 | 30 | 0,5585 | -0,0165 | 0,9211 |
| Rv2494 | Rv2494 | 66R>P | 7 | 19 | 12 | 30 | 0,5585 | -0,0165 | 0,9211 |
| Rv2631 | Rv2631 | 393G>D | 7 | 19 | 12 | 30 | 0,5585 | -0,0165 | 0,9211 |

| Rv3077 | Rv3077 | 452R>H | 7 | 19 | 12 | 30 | 0,5585 | -0,0165 | 0,9211 |
| --- | --- | --- | --- | --- | --- | --- | --- | --- | --- |
| Rv3097c | lipY | 427P>L | 7 | 19 | 12 | 30 | 0,5585 | -0,0165 | 0,9211 |
| Rv3396c | guaA | 420L>V | 7 | 19 | 12 | 30 | 0,5585 | -0,0165 | 0,9211 |
| Rv3435c | Rv3435c | 207A>V | 7 | 19 | 12 | 30 | 0,5585 | -0,0165 | 0,9211 |
| Rv3447c | Rv3447c | 630G>E | 7 | 19 | 12 | 30 | 0,5585 | -0,0165 | 0,9211 |
| Rv3451 | cut3 | 209G>D | 7 | 19 | 12 | 30 | 0,5585 | -0,0165 | 0,9211 |
| Rv3506 | fadD17 | 282F>V | 7 | 19 | 12 | 30 | 0,5585 | -0,0165 | 0,9211 |
| Rv3624c | hpt | 75L>M | 7 | 19 | 12 | 30 | 0,5585 | -0,0165 | 0,9211 |
| Rv3703c | Rv3703c | 193P>Q | 7 | 19 | 12 | 30 | 0,5585 | -0,0165 | 0,9211 |
| Rv3793 | embC | 981V>L | 7 | 19 | 12 | 30 | 0,5585 | -0,0165 | 0,9211 |
| Rv3878 | Rv3878 | 144T>K | 7 | 19 | 12 | 30 | 0,5585 | -0,0165 | 0,9211 |
| Rv3901c | Rv3901c | 36A>T | 7 | 19 | 12 | 30 | 0,5585 | -0,0165 | 0,9211 |
| Rv3911 | sigM | 21D>N | 7 | 19 | 12 | 30 | 0,5585 | -0,0165 | 0,9211 |
| Rv2048c | pks12 | 3004S>L | 15 | 11 | 25 | 17 | 0,5593 | -0,0183 | 0,9273 |
| Rv0336 | Rv0336 | 207D>Y | 1 | 25 | 2 | 40 | 0,5709 | -0,0092 | 0,8000 |
| Rv0515 | Rv0515 | 207D>Y | 1 | 25 | 2 | 40 | 0,5709 | -0,0092 | 0,8000 |
| Rv1196 | PPE18 | 186A>V | 1 | 25 | 2 | 40 | 0,5709 | -0,0092 | 0,8000 |
| Rv2591 | PE_PGRS44 | 433Q>L | 1 | 25 | 2 | 40 | 0,5709 | -0,0092 | 0,8000 |
| Rv3179 | Rv3179 | 59V>A | 1 | 25 | 2 | 40 | 0,5709 | -0,0092 | 0,8000 |
| Rv3347c | PPE55 | 2441P>L | 1 | 25 | 2 | 40 | 0,5709 | -0,0092 | 0,8000 |
| Rv3508 | PE_PGRS54 | 1499G>A | 1 | 25 | 2 | 40 | 0,5709 | -0,0092 | 0,8000 |
| Rv3597c | lsr2 | 8T>I | 1 | 25 | 2 | 40 | 0,5709 | -0,0092 | 0,8000 |
| Rv0050 | ponA1 | 631P>S | 13 | 13 | 22 | 20 | 0,5757 | -0,0238 | 0,9091 |
| Rv0969 | ctpV | 618A>T | 5 | 21 | 9 | 33 | 0,5862 | -0,0220 | 0,8730 |
| Rv0012 | Rv0012 | 258G>V | 2 | 24 | 4 | 38 | 0,6021 | -0,0183 | 0,7917 |
| Rv0013 | trpG | 68I>M | 2 | 24 | 4 | 38 | 0,6021 | -0,0183 | 0,7917 |
| Rv0014c | pknB | 451R>L | 2 | 24 | 4 | 38 | 0,6021 | -0,0183 | 0,7917 |
| Rv0015c | pknA | 369Q>R | 2 | 24 | 4 | 38 | 0,6021 | -0,0183 | 0,7917 |
| Rv0015c | pknA | 370Q>P | 2 | 24 | 4 | 38 | 0,6021 | -0,0183 | 0,7917 |
| Rv0032 | bioF2 | 572D>H | 2 | 24 | 4 | 38 | 0,6021 | -0,0183 | 0,7917 |
| Rv0035 | fadD34 | 16S>W | 2 | 24 | 4 | 38 | 0,6021 | -0,0183 | 0,7917 |
| Rv0035 | fadD34 | 26T>S | 2 | 24 | 4 | 38 | 0,6021 | -0,0183 | 0,7917 |
| Rv0039c | Rv0039c | 24C>F | 2 | 24 | 4 | 38 | 0,6021 | -0,0183 | 0,7917 |

| Rv0041 | leuS | 403R>G | 2 | 24 | 4 | 38 | 0,6021 | -0,0183 | 0,7917 |
| --- | --- | --- | --- | --- | --- | --- | --- | --- | --- |
| Rv0045c | Rv0045c | 194V>I | 2 | 24 | 4 | 38 | 0,6021 | -0,0183 | 0,7917 |
| Rv0061 | Rv0061 | 54P>S | 2 | 24 | 4 | 38 | 0,6021 | -0,0183 | 0,7917 |
| Rv0064 | Rv0064 | 550V>F | 2 | 24 | 4 | 38 | 0,6021 | -0,0183 | 0,7917 |
| Rv0078A | Rv0078A | 112E>K | 2 | 24 | 4 | 38 | 0,6021 | -0,0183 | 0,7917 |
| Rv0096 | PPE1 | 138V>A | 2 | 24 | 4 | 38 | 0,6021 | -0,0183 | 0,7917 |
| Rv0104 | Rv0104 | 380Q>* | 2 | 24 | 4 | 38 | 0,6021 | -0,0183 | 0,7917 |
| Rv0104 | Rv0104 | 402Y>H | 2 | 24 | 4 | 38 | 0,6021 | -0,0183 | 0,7917 |
| Rv0115 | hddA | 262G>V | 2 | 24 | 4 | 38 | 0,6021 | -0,0183 | 0,7917 |
| Rv0117 | oxyS | 142D>N | 2 | 24 | 4 | 38 | 0,6021 | -0,0183 | 0,7917 |
| Rv0118c | oxcA | 224S>G | 2 | 24 | 4 | 38 | 0,6021 | -0,0183 | 0,7917 |
| Rv0120c | fusA2 | 170D>E | 2 | 24 | 4 | 38 | 0,6021 | -0,0183 | 0,7917 |
| Rv0136 | cyp138 | 114P>F | 2 | 24 | 4 | 38 | 0,6021 | -0,0183 | 0,7917 |
| Rv0169 | mce1A | 313S>A | 2 | 24 | 4 | 38 | 0,6021 | -0,0183 | 0,7917 |
| Rv0172 | mce1D | 188I>T | 2 | 24 | 4 | 38 | 0,6021 | -0,0183 | 0,7917 |
| Rv0182c | sigG | 287G>D | 2 | 24 | 4 | 38 | 0,6021 | -0,0183 | 0,7917 |
| Rv0182c | sigG | 332D>Y | 2 | 24 | 4 | 38 | 0,6021 | -0,0183 | 0,7917 |
| Rv0185 | Rv0185 | 88A>V | 2 | 24 | 4 | 38 | 0,6021 | -0,0183 | 0,7917 |
| Rv0191 | Rv0191 | 213A>T | 2 | 24 | 4 | 38 | 0,6021 | -0,0183 | 0,7917 |
| Rv0194 | Rv0194 | 1098P>L | 2 | 24 | 4 | 38 | 0,6021 | -0,0183 | 0,7917 |
| Rv0209 | Rv0209 | 162V>A | 2 | 24 | 4 | 38 | 0,6021 | -0,0183 | 0,7917 |
| Rv0210 | Rv0210 | 486A>T | 2 | 24 | 4 | 38 | 0,6021 | -0,0183 | 0,7917 |
| Rv0221 | Rv0221 | 21M>I | 2 | 24 | 4 | 38 | 0,6021 | -0,0183 | 0,7917 |
| Rv0223c | Rv0223c | 5A>G | 2 | 24 | 4 | 38 | 0,6021 | -0,0183 | 0,7917 |
| Rv0226c | Rv0226c | 379P>A | 2 | 24 | 4 | 38 | 0,6021 | -0,0183 | 0,7917 |
| Rv0236c | Rv0236c | 1081A>V | 2 | 24 | 4 | 38 | 0,6021 | -0,0183 | 0,7917 |
| Rv0245 | Rv0245 | 103S>F | 2 | 24 | 4 | 38 | 0,6021 | -0,0183 | 0,7917 |
| Rv0252 | nirB | 775V>L | 2 | 24 | 4 | 38 | 0,6021 | -0,0183 | 0,7917 |
| Rv0282 | Rv0282 | 6E>A | 2 | 24 | 4 | 38 | 0,6021 | -0,0183 | 0,7917 |
| Rv0290 | Rv0290 | 76S>N | 2 | 24 | 4 | 38 | 0,6021 | -0,0183 | 0,7917 |
| Rv0290 | Rv0290 | 95A>T | 2 | 24 | 4 | 38 | 0,6021 | -0,0183 | 0,7917 |
| Rv0302 | Rv0302 | 84H>D | 2 | 24 | 4 | 38 | 0,6021 | -0,0183 | 0,7917 |
| Rv0311 | Rv0311 | 119E>D | 2 | 24 | 4 | 38 | 0,6021 | -0,0183 | 0,7917 |

| Rv0315 | Rv0315 | 39P>S | 2 | 24 | 4 | 38 | 0,6021 | -0,0183 | 0,7917 |
| --- | --- | --- | --- | --- | --- | --- | --- | --- | --- |
| Rv0325 | Rv0325 | 75*>Q | 2 | 24 | 4 | 38 | 0,6021 | -0,0183 | 0,7917 |
| Rv0368c | Rv0368c | 249R>H | 2 | 24 | 4 | 38 | 0,6021 | -0,0183 | 0,7917 |
| Rv0376c | Rv0376c | 14T>P | 2 | 24 | 4 | 38 | 0,6021 | -0,0183 | 0,7917 |
| Rv0380c | Rv0380c | 76M>V | 2 | 24 | 4 | 38 | 0,6021 | -0,0183 | 0,7917 |
| Rv0398c | Rv0398c | 29E>D | 2 | 24 | 4 | 38 | 0,6021 | -0,0183 | 0,7917 |
| Rv0404 | fadD30 | 207P>L | 2 | 24 | 4 | 38 | 0,6021 | -0,0183 | 0,7917 |
| Rv0408 | pta | 122G>A | 2 | 24 | 4 | 38 | 0,6021 | -0,0183 | 0,7917 |
| Rv0419 | lpqM | 297A>T | 2 | 24 | 4 | 38 | 0,6021 | -0,0183 | 0,7917 |
| Rv0428c | Rv0428c | 149D>G | 2 | 24 | 4 | 38 | 0,6021 | -0,0183 | 0,7917 |
| Rv0444c | Rv0444c | 81E>D | 2 | 24 | 4 | 38 | 0,6021 | -0,0183 | 0,7917 |
| Rv0452 | Rv0452 | 125H>D | 2 | 24 | 4 | 38 | 0,6021 | -0,0183 | 0,7917 |
| Rv0457c | Rv0457c | 82R>P | 2 | 24 | 4 | 38 | 0,6021 | -0,0183 | 0,7917 |
| Rv0465c | Rv0465c | 106C>R | 2 | 24 | 4 | 38 | 0,6021 | -0,0183 | 0,7917 |
| Rv0486 | Rv0486 | 187A>V | 2 | 24 | 4 | 38 | 0,6021 | -0,0183 | 0,7917 |
| Rv0492c | Rv0492c | 70S>A | 2 | 24 | 4 | 38 | 0,6021 | -0,0183 | 0,7917 |
| Rv0493c | Rv0493c | 174S>G | 2 | 24 | 4 | 38 | 0,6021 | -0,0183 | 0,7917 |
| Rv0529 | ccsA | 245I>M | 2 | 24 | 4 | 38 | 0,6021 | -0,0183 | 0,7917 |
| Rv0530 | Rv0530 | 231P>L | 2 | 24 | 4 | 38 | 0,6021 | -0,0183 | 0,7917 |
| Rv0536 | galE3 | 80V>I | 2 | 24 | 4 | 38 | 0,6021 | -0,0183 | 0,7917 |
| Rv0537c | Rv0537c | 290T>A | 2 | 24 | 4 | 38 | 0,6021 | -0,0183 | 0,7917 |
| Rv0565c | Rv0565c | 110R>H | 2 | 24 | 4 | 38 | 0,6021 | -0,0183 | 0,7917 |
| Rv0575c | Rv0575c | 238D>V | 2 | 24 | 4 | 38 | 0,6021 | -0,0183 | 0,7917 |
| Rv0576 | Rv0576 | 233R>H | 2 | 24 | 4 | 38 | 0,6021 | -0,0183 | 0,7917 |
| Rv0578c | PE_PGRS7 | 785A>T | 2 | 24 | 4 | 38 | 0,6021 | -0,0183 | 0,7917 |
| Rv0588 | yrbE2B | 113A>T | 2 | 24 | 4 | 38 | 0,6021 | -0,0183 | 0,7917 |
| Rv0594 | mce2F | 432N>S | 2 | 24 | 4 | 38 | 0,6021 | -0,0183 | 0,7917 |
| Rv0613c | Rv0613c | 728T>A | 2 | 24 | 4 | 38 | 0,6021 | -0,0183 | 0,7917 |
| Rv0620 | galK | 199C>R | 2 | 24 | 4 | 38 | 0,6021 | -0,0183 | 0,7917 |
| Rv0629c | recD | 120E>D | 2 | 24 | 4 | 38 | 0,6021 | -0,0183 | 0,7917 |
| Rv0644c | mmaA2 | 213E>D | 2 | 24 | 4 | 38 | 0,6021 | -0,0183 | 0,7917 |
| Rv0663 | atsD | 335R>S | 2 | 24 | 4 | 38 | 0,6021 | -0,0183 | 0,7917 |
| Rv0663 | atsD | 349D>G | 2 | 24 | 4 | 38 | 0,6021 | -0,0183 | 0,7917 |

| Rv0673 | echA4 | 125F>L | 2 | 24 | 4 | 38 | 0,6021 | -0,0183 | 0,7917 |
| --- | --- | --- | --- | --- | --- | --- | --- | --- | --- |
| Rv0676c | mmpL5 | 767D>N | 2 | 24 | 4 | 38 | 0,6021 | -0,0183 | 0,7917 |
| Rv0676c | mmpL5 | 794T>I | 2 | 24 | 4 | 38 | 0,6021 | -0,0183 | 0,7917 |
| Rv0679c | Rv0679c | 142N>K | 2 | 24 | 4 | 38 | 0,6021 | -0,0183 | 0,7917 |
| Rv0691c | Rv0691c | 140A>T | 2 | 24 | 4 | 38 | 0,6021 | -0,0183 | 0,7917 |
| Rv0697 | Rv0697 | 475A>P | 2 | 24 | 4 | 38 | 0,6021 | -0,0183 | 0,7917 |
| Rv0699 | Rv0699 | 13D>G | 2 | 24 | 4 | 38 | 0,6021 | -0,0183 | 0,7917 |
| Rv0728c | serA2 | 242R>H | 2 | 24 | 4 | 38 | 0,6021 | -0,0183 | 0,7917 |
| Rv0745 | Rv0745 | 153T>M | 2 | 24 | 4 | 38 | 0,6021 | -0,0183 | 0,7917 |
| Rv0768 | aldA | 102L>R | 2 | 24 | 4 | 38 | 0,6021 | -0,0183 | 0,7917 |
| Rv0773c | ggtA | 447R>C | 2 | 24 | 4 | 38 | 0,6021 | -0,0183 | 0,7917 |
| Rv0808 | purF | 476R>L | 2 | 24 | 4 | 38 | 0,6021 | -0,0183 | 0,7917 |
| Rv0814c | sseC2 | 100T>A | 2 | 24 | 4 | 38 | 0,6021 | -0,0183 | 0,7917 |
| Rv0825c | Rv0825c | 68F>C | 2 | 24 | 4 | 38 | 0,6021 | -0,0183 | 0,7917 |
| Rv0836c | Rv0836c | 218*>W | 2 | 24 | 4 | 38 | 0,6021 | -0,0183 | 0,7917 |
| Rv0844c | narL | 169G>R | 2 | 24 | 4 | 38 | 0,6021 | -0,0183 | 0,7917 |
| Rv0845 | Rv0845 | 219A>E | 2 | 24 | 4 | 38 | 0,6021 | -0,0183 | 0,7917 |
| Rv0855 | far | 24A>V | 2 | 24 | 4 | 38 | 0,6021 | -0,0183 | 0,7917 |
| Rv0858c | Rv0858c | 274A>T | 2 | 24 | 4 | 38 | 0,6021 | -0,0183 | 0,7917 |
| Rv0862c | Rv0862c | 749L>S | 2 | 24 | 4 | 38 | 0,6021 | -0,0183 | 0,7917 |
| Rv0874c | Rv0874c | 243G>S | 2 | 24 | 4 | 38 | 0,6021 | -0,0183 | 0,7917 |
| Rv0890c | Rv0890c | 234E>G | 2 | 24 | 4 | 38 | 0,6021 | -0,0183 | 0,7917 |
| Rv0893c | Rv0893c | 31K>E | 2 | 24 | 4 | 38 | 0,6021 | -0,0183 | 0,7917 |
| Rv0907 | Rv0907 | 459Y>S | 2 | 24 | 4 | 38 | 0,6021 | -0,0183 | 0,7917 |
| Rv0918 | Rv0918 | 46S>G | 2 | 24 | 4 | 38 | 0,6021 | -0,0183 | 0,7917 |
| Rv0922 | Rv0922 | 178G>D | 2 | 24 | 4 | 38 | 0,6021 | -0,0183 | 0,7917 |
| Rv0945 | Rv0945 | 180R>G | 2 | 24 | 4 | 38 | 0,6021 | -0,0183 | 0,7917 |
| Rv0959 | Rv0959 | 238N>D | 2 | 24 | 4 | 38 | 0,6021 | -0,0183 | 0,7917 |
| Rv0969 | ctpV | 506D>N | 2 | 24 | 4 | 38 | 0,6021 | -0,0183 | 0,7917 |
| Rv0982 | mprB | 339L>H | 2 | 24 | 4 | 38 | 0,6021 | -0,0183 | 0,7917 |
| Rv0987 | Rv0987 | 372A>V | 2 | 24 | 4 | 38 | 0,6021 | -0,0183 | 0,7917 |
| Rv0987 | Rv0987 | 717F>V | 2 | 24 | 4 | 38 | 0,6021 | -0,0183 | 0,7917 |
| Rv0988 | Rv0988 | 191L>A | 2 | 24 | 4 | 38 | 0,6021 | -0,0183 | 0,7917 |

| Rv0990c | Rv0990c | 54S>A | 2 | 24 | 4 | 38 | 0,6021 | -0,0183 | 0,7917 |
| --- | --- | --- | --- | --- | --- | --- | --- | --- | --- |
| Rv0995 | rimJ | 23G>S | 2 | 24 | 4 | 38 | 0,6021 | -0,0183 | 0,7917 |
| Rv1023 | eno | 429K>Q | 2 | 24 | 4 | 38 | 0,6021 | -0,0183 | 0,7917 |
| Rv1023 | eno | 8R>G | 2 | 24 | 4 | 38 | 0,6021 | -0,0183 | 0,7917 |
| Rv1027c | kdpE | 60G>S | 2 | 24 | 4 | 38 | 0,6021 | -0,0183 | 0,7917 |
| Rv1037c | esxI | 23S>L | 2 | 24 | 4 | 38 | 0,6021 | -0,0183 | 0,7917 |
| Rv1042c | Rv1041c | 108G>E | 2 | 24 | 4 | 38 | 0,6021 | -0,0183 | 0,7917 |
| Rv1046c | Rv1046c | 151R>S | 2 | 24 | 4 | 38 | 0,6021 | -0,0183 | 0,7917 |
| Rv1048c | Rv1048c | 23E>D | 2 | 24 | 4 | 38 | 0,6021 | -0,0183 | 0,7917 |
| Rv1054 | Rv1054 | 77G>D | 2 | 24 | 4 | 38 | 0,6021 | -0,0183 | 0,7917 |
| Rv1076 | lipU | 272Q>P | 2 | 24 | 4 | 38 | 0,6021 | -0,0183 | 0,7917 |
| Rv1086 | Rv1086 | 259S>R | 2 | 24 | 4 | 38 | 0,6021 | -0,0183 | 0,7917 |
| Rv1090 | celA2b | 49Q>K | 2 | 24 | 4 | 38 | 0,6021 | -0,0183 | 0,7917 |
| Rv1102c | Rv1102c | 65T>I | 2 | 24 | 4 | 38 | 0,6021 | -0,0183 | 0,7917 |
| Rv1125 | Rv1125 | 101S>G | 2 | 24 | 4 | 38 | 0,6021 | -0,0183 | 0,7917 |
| Rv1130 | Rv1130 | 3D>G | 2 | 24 | 4 | 38 | 0,6021 | -0,0183 | 0,7917 |
| Rv1149 | Rv1149 | 129G>E | 2 | 24 | 4 | 38 | 0,6021 | -0,0183 | 0,7917 |
| Rv1155 | Rv1155 | 115S>P | 2 | 24 | 4 | 38 | 0,6021 | -0,0183 | 0,7917 |
| Rv1159 | pimE | 4T>P | 2 | 24 | 4 | 38 | 0,6021 | -0,0183 | 0,7917 |
| Rv1160 | mutT2 | 58G>R | 2 | 24 | 4 | 38 | 0,6021 | -0,0183 | 0,7917 |
| Rv1163 | narJ | 179P>R | 2 | 24 | 4 | 38 | 0,6021 | -0,0183 | 0,7917 |
| Rv1168c | PPE17 | 167P>L | 2 | 24 | 4 | 38 | 0,6021 | -0,0183 | 0,7917 |
| Rv1183 | mmpL10 | 408T>A | 2 | 24 | 4 | 38 | 0,6021 | -0,0183 | 0,7917 |
| Rv1193 | fadD36 | 124P>L | 2 | 24 | 4 | 38 | 0,6021 | -0,0183 | 0,7917 |
| Rv1206 | fadD6 | 134E>D | 2 | 24 | 4 | 38 | 0,6021 | -0,0183 | 0,7917 |
| Rv1217c | Rv1217c | 173A>T | 2 | 24 | 4 | 38 | 0,6021 | -0,0183 | 0,7917 |
| Rv1224 | tatB | 8W>G | 2 | 24 | 4 | 38 | 0,6021 | -0,0183 | 0,7917 |
| Rv1248c | kgd | 764V>M | 2 | 24 | 4 | 38 | 0,6021 | -0,0183 | 0,7917 |
| Rv1317c | alkA | 12I>V | 2 | 24 | 4 | 38 | 0,6021 | -0,0183 | 0,7917 |
| Rv1326c | glgB | 470S>P | 2 | 24 | 4 | 38 | 0,6021 | -0,0183 | 0,7917 |
| Rv1329c | dinG | 80T>K | 2 | 24 | 4 | 38 | 0,6021 | -0,0183 | 0,7917 |
| Rv1367c | Rv1367c | 169I>V | 2 | 24 | 4 | 38 | 0,6021 | -0,0183 | 0,7917 |
| Rv1368 | lprF | 260A>V | 2 | 24 | 4 | 38 | 0,6021 | -0,0183 | 0,7917 |

| Rv1373 | Rv1373 | 231P>L | 2 | 24 | 4 | 38 | 0,6021 | -0,0183 | 0,7917 |
| --- | --- | --- | --- | --- | --- | --- | --- | --- | --- |
| Rv1383 | carA | 273S>P | 2 | 24 | 4 | 38 | 0,6021 | -0,0183 | 0,7917 |
| Rv1387 | PPE20 | 57E>D | 2 | 24 | 4 | 38 | 0,6021 | -0,0183 | 0,7917 |
| Rv1400c | lipI | 106T>P | 2 | 24 | 4 | 38 | 0,6021 | -0,0183 | 0,7917 |
| Rv1400c | lipI | 90F>L | 2 | 24 | 4 | 38 | 0,6021 | -0,0183 | 0,7917 |
| Rv1420 | uvrC | 289V>I | 2 | 24 | 4 | 38 | 0,6021 | -0,0183 | 0,7917 |
| Rv1420 | uvrC | 434V>A | 2 | 24 | 4 | 38 | 0,6021 | -0,0183 | 0,7917 |
| Rv1431 | Rv1431 | 65N>T | 2 | 24 | 4 | 38 | 0,6021 | -0,0183 | 0,7917 |
| Rv1439c | Rv1439c | 29L>I | 2 | 24 | 4 | 38 | 0,6021 | -0,0183 | 0,7917 |
| Rv1442 | bisC | 131L>V | 2 | 24 | 4 | 38 | 0,6021 | -0,0183 | 0,7917 |
| Rv1458c | Rv1458c | 133T>A | 2 | 24 | 4 | 38 | 0,6021 | -0,0183 | 0,7917 |
| Rv1461 | Rv1461 | 150V>I | 2 | 24 | 4 | 38 | 0,6021 | -0,0183 | 0,7917 |
| Rv1463 | Rv1463 | 198E>G | 2 | 24 | 4 | 38 | 0,6021 | -0,0183 | 0,7917 |
| Rv1489 | Rv1489 | 52K>T | 2 | 24 | 4 | 38 | 0,6021 | -0,0183 | 0,7917 |
| Rv1497 | lipL | 42M>V | 2 | 24 | 4 | 38 | 0,6021 | -0,0183 | 0,7917 |
| Rv1505c | Rv1505c | 51A>T | 2 | 24 | 4 | 38 | 0,6021 | -0,0183 | 0,7917 |
| Rv1508c | Rv1508c | 16P>S | 2 | 24 | 4 | 38 | 0,6021 | -0,0183 | 0,7917 |
| Rv1515c | Rv1515c | 281G>V | 2 | 24 | 4 | 38 | 0,6021 | -0,0183 | 0,7917 |
| Rv1520 | Rv1520 | 226R>C | 2 | 24 | 4 | 38 | 0,6021 | -0,0183 | 0,7917 |
| Rv1522c | mmpL12 | 381S>P | 2 | 24 | 4 | 38 | 0,6021 | -0,0183 | 0,7917 |
| Rv1527c | pks5 | 2061L>R | 2 | 24 | 4 | 38 | 0,6021 | -0,0183 | 0,7917 |
| Rv1532c | Rv1532c | 76A>P | 2 | 24 | 4 | 38 | 0,6021 | -0,0183 | 0,7917 |
| Rv1538c | ansA | 281G>S | 2 | 24 | 4 | 38 | 0,6021 | -0,0183 | 0,7917 |
| Rv1629 | polA | 186T>P | 2 | 24 | 4 | 38 | 0,6021 | -0,0183 | 0,7917 |
| Rv1629 | polA | 188R>G | 2 | 24 | 4 | 38 | 0,6021 | -0,0183 | 0,7917 |
| Rv1634 | Rv1634 | 198G>R | 2 | 24 | 4 | 38 | 0,6021 | -0,0183 | 0,7917 |
| Rv1640c | lysS | 701I>T | 2 | 24 | 4 | 38 | 0,6021 | -0,0183 | 0,7917 |
| Rv1649 | pheS | 287A>D | 2 | 24 | 4 | 38 | 0,6021 | -0,0183 | 0,7917 |
| Rv1661 | pks7 | 814E>A | 2 | 24 | 4 | 38 | 0,6021 | -0,0183 | 0,7917 |
| Rv1662 | pks8 | 1228L>V | 2 | 24 | 4 | 38 | 0,6021 | -0,0183 | 0,7917 |
| Rv1681 | moeX | 277T>A | 2 | 24 | 4 | 38 | 0,6021 | -0,0183 | 0,7917 |
| Rv1705c | PPE22 | 313L>V | 2 | 24 | 4 | 38 | 0,6021 | -0,0183 | 0,7917 |
| Rv1730c | Rv1730c | 435D>E | 2 | 24 | 4 | 38 | 0,6021 | -0,0183 | 0,7917 |

| Rv1739c | Rv1739c | 32G>V | 2 | 24 | 4 | 38 | 0,6021 | -0,0183 | 0,7917 |
| --- | --- | --- | --- | --- | --- | --- | --- | --- | --- |
| Rv1765c | Rv1765c | 151E>D | 2 | 24 | 4 | 38 | 0,6021 | -0,0183 | 0,7917 |
| Rv1769 | Rv1769 | 401D>A | 2 | 24 | 4 | 38 | 0,6021 | -0,0183 | 0,7917 |
| Rv1785c | cyp143 | 334G>A | 2 | 24 | 4 | 38 | 0,6021 | -0,0183 | 0,7917 |
| Rv1808 | PPE32 | 301G>A | 2 | 24 | 4 | 38 | 0,6021 | -0,0183 | 0,7917 |
| Rv1821 | secA2 | 410V>M | 2 | 24 | 4 | 38 | 0,6021 | -0,0183 | 0,7917 |
| Rv1823 | Rv1823 | 24Q>K | 2 | 24 | 4 | 38 | 0,6021 | -0,0183 | 0,7917 |
| Rv1826 | gcvH | 121T>K | 2 | 24 | 4 | 38 | 0,6021 | -0,0183 | 0,7917 |
| Rv1835c | Rv1835c | 179C>R | 2 | 24 | 4 | 38 | 0,6021 | -0,0183 | 0,7917 |
| Rv1837c | glcB | 104G>S | 2 | 24 | 4 | 38 | 0,6021 | -0,0183 | 0,7917 |
| Rv1847 | Rv1847 | 132R>W | 2 | 24 | 4 | 38 | 0,6021 | -0,0183 | 0,7917 |
| Rv1872c | lldD2 | 59G>A | 2 | 24 | 4 | 38 | 0,6021 | -0,0183 | 0,7917 |
| Rv1904 | Rv1904 | 90L>W | 2 | 24 | 4 | 38 | 0,6021 | -0,0183 | 0,7917 |
| Rv1908c | katG | 463R>L | 2 | 24 | 4 | 38 | 0,6021 | -0,0183 | 0,7917 |
| Rv1912c | fadB5 | 328D>G | 2 | 24 | 4 | 38 | 0,6021 | -0,0183 | 0,7917 |
| Rv1918c | PPE35 | 896L>S | 2 | 24 | 4 | 38 | 0,6021 | -0,0183 | 0,7917 |
| Rv1920 | Rv1920 | 253E>A | 2 | 24 | 4 | 38 | 0,6021 | -0,0183 | 0,7917 |
| Rv1925 | fadD31 | 94R>C | 2 | 24 | 4 | 38 | 0,6021 | -0,0183 | 0,7917 |
| Rv1927 | Rv1927 | 51E>D | 2 | 24 | 4 | 38 | 0,6021 | -0,0183 | 0,7917 |
| Rv1934c | fadE17 | 172I>T | 2 | 24 | 4 | 38 | 0,6021 | -0,0183 | 0,7917 |
| Rv1936 | Rv1936 | 68Q>H | 2 | 24 | 4 | 38 | 0,6021 | -0,0183 | 0,7917 |
| Rv1938 | ephB | 158G>W | 2 | 24 | 4 | 38 | 0,6021 | -0,0183 | 0,7917 |
| Rv1948c | Rv1948c | 5G>R | 2 | 24 | 4 | 38 | 0,6021 | -0,0183 | 0,7917 |
| Rv1966 | mce3A | 47A>T | 2 | 24 | 4 | 38 | 0,6021 | -0,0183 | 0,7917 |
| Rv1972 | Rv1972 | 124H>Q | 2 | 24 | 4 | 38 | 0,6021 | -0,0183 | 0,7917 |
| Rv1978 | Rv1978 | 226S>T | 2 | 24 | 4 | 38 | 0,6021 | -0,0183 | 0,7917 |
| Rv1986 | Rv1986 | 12C>Y | 2 | 24 | 4 | 38 | 0,6021 | -0,0183 | 0,7917 |
| Rv1999c | Rv1999c | 240L>M | 2 | 24 | 4 | 38 | 0,6021 | -0,0183 | 0,7917 |
| Rv2000 | Rv2000 | 219P>Q | 2 | 24 | 4 | 38 | 0,6021 | -0,0183 | 0,7917 |
| Rv2015c | Rv2015c | 151E>D | 2 | 24 | 4 | 38 | 0,6021 | -0,0183 | 0,7917 |
| Rv2047c | Rv2047c | 174T>A | 2 | 24 | 4 | 38 | 0,6021 | -0,0183 | 0,7917 |
| Rv2057c | rpmG | 46R>L | 2 | 24 | 4 | 38 | 0,6021 | -0,0183 | 0,7917 |
| Rv2059 | Rv2059 | 317T>K | 2 | 24 | 4 | 38 | 0,6021 | -0,0183 | 0,7917 |

| Rv2059 | Rv2059 | 71H>Q | 2 | 24 | 4 | 38 | 0,6021 | -0,0183 | 0,7917 |
| --- | --- | --- | --- | --- | --- | --- | --- | --- | --- |
| Rv2062c | cobN | 145R>H | 2 | 24 | 4 | 38 | 0,6021 | -0,0183 | 0,7917 |
| Rv2062c | cobN | 677E>K | 2 | 24 | 4 | 38 | 0,6021 | -0,0183 | 0,7917 |
| Rv2071c | cobM | 145I>M | 2 | 24 | 4 | 38 | 0,6021 | -0,0183 | 0,7917 |
| Rv2075c | Rv2075c | 364P>Q | 2 | 24 | 4 | 38 | 0,6021 | -0,0183 | 0,7917 |
| Rv2079 | Rv2079 | 609Q>* | 2 | 24 | 4 | 38 | 0,6021 | -0,0183 | 0,7917 |
| Rv2082 | Rv2082 | 183T>A | 2 | 24 | 4 | 38 | 0,6021 | -0,0183 | 0,7917 |
| Rv2082 | Rv2082 | 35K>E | 2 | 24 | 4 | 38 | 0,6021 | -0,0183 | 0,7917 |
| Rv2082 | Rv2082 | 376P>L | 2 | 24 | 4 | 38 | 0,6021 | -0,0183 | 0,7917 |
| Rv2088 | pknJ | 30L>R | 2 | 24 | 4 | 38 | 0,6021 | -0,0183 | 0,7917 |
| Rv2089c | pepE | 336P>L | 2 | 24 | 4 | 38 | 0,6021 | -0,0183 | 0,7917 |
| Rv2096c | Rv2096c | 174S>P | 2 | 24 | 4 | 38 | 0,6021 | -0,0183 | 0,7917 |
| Rv2103c | Rv2103c | 16T>A | 2 | 24 | 4 | 38 | 0,6021 | -0,0183 | 0,7917 |
| Rv2109c | prcA | 182R>G | 2 | 24 | 4 | 38 | 0,6021 | -0,0183 | 0,7917 |
| Rv2127 | ansP1 | 9G>D | 2 | 24 | 4 | 38 | 0,6021 | -0,0183 | 0,7917 |
| Rv2139 | pyrD | 339G>S | 2 | 24 | 4 | 38 | 0,6021 | -0,0183 | 0,7917 |
| Rv2153c | murG | 36V>L | 2 | 24 | 4 | 38 | 0,6021 | -0,0183 | 0,7917 |
| Rv2160c | Rv2160A | 155V>A | 2 | 24 | 4 | 38 | 0,6021 | -0,0183 | 0,7917 |
| Rv2180c | Rv2180c | 249W>* | 2 | 24 | 4 | 38 | 0,6021 | -0,0183 | 0,7917 |
| Rv2187 | fadD15 | 100T>I | 2 | 24 | 4 | 38 | 0,6021 | -0,0183 | 0,7917 |
| Rv2247 | accD6 | 229D>G | 2 | 24 | 4 | 38 | 0,6021 | -0,0183 | 0,7917 |
| Rv2248 | Rv2248 | 199V>G | 2 | 24 | 4 | 38 | 0,6021 | -0,0183 | 0,7917 |
| Rv2264c | Rv2264c | 487T>A | 2 | 24 | 4 | 38 | 0,6021 | -0,0183 | 0,7917 |
| Rv2274c | Rv2274c | 41G>V | 2 | 24 | 4 | 38 | 0,6021 | -0,0183 | 0,7917 |
| Rv2276 | cyp121 | 318H>Y | 2 | 24 | 4 | 38 | 0,6021 | -0,0183 | 0,7917 |
| Rv2290 | lppO | 16A>S | 2 | 24 | 4 | 38 | 0,6021 | -0,0183 | 0,7917 |
| Rv2308 | Rv2308 | 212D>N | 2 | 24 | 4 | 38 | 0,6021 | -0,0183 | 0,7917 |
| Rv2316 | uspA | 67D>H | 2 | 24 | 4 | 38 | 0,6021 | -0,0183 | 0,7917 |
| Rv2328 | PE23 | 282S>R | 2 | 24 | 4 | 38 | 0,6021 | -0,0183 | 0,7917 |
| Rv2328 | PE23 | 344A>T | 2 | 24 | 4 | 38 | 0,6021 | -0,0183 | 0,7917 |
| Rv2349c | plcC | 361G>C | 2 | 24 | 4 | 38 | 0,6021 | -0,0183 | 0,7917 |
| Rv2366c | Rv2366c | 155R>C | 2 | 24 | 4 | 38 | 0,6021 | -0,0183 | 0,7917 |
| Rv2383c | mbtB | 674V>L | 2 | 24 | 4 | 38 | 0,6021 | -0,0183 | 0,7917 |

| Rv2400c | subI | 76A>T | 2 | 24 | 4 | 38 | 0,6021 | -0,0183 | 0,7917 |
| --- | --- | --- | --- | --- | --- | --- | --- | --- | --- |
| Rv2409c | PE24 | 216T>N | 2 | 24 | 4 | 38 | 0,6021 | -0,0183 | 0,7917 |
| Rv2433c | Rv2433c | 26L>R | 2 | 24 | 4 | 38 | 0,6021 | -0,0183 | 0,7917 |
| Rv2440c | obgE | 471S>R | 2 | 24 | 4 | 38 | 0,6021 | -0,0183 | 0,7917 |
| Rv2450c | rpfE | 20T>R | 2 | 24 | 4 | 38 | 0,6021 | -0,0183 | 0,7917 |
| Rv2471 | aglA | 331V>A | 2 | 24 | 4 | 38 | 0,6021 | -0,0183 | 0,7917 |
| Rv2484c | Rv2484c | 466G>D | 2 | 24 | 4 | 38 | 0,6021 | -0,0183 | 0,7917 |
| Rv2485c | lipQ | 60R>L | 2 | 24 | 4 | 38 | 0,6021 | -0,0183 | 0,7917 |
| Rv2488c | Rv2488c | 265T>I | 2 | 24 | 4 | 38 | 0,6021 | -0,0183 | 0,7917 |
| Rv2490c | PE_PGRS43 | 1399G>D | 2 | 24 | 4 | 38 | 0,6021 | -0,0183 | 0,7917 |
| Rv2492 | Rv2492 | 70A>D | 2 | 24 | 4 | 38 | 0,6021 | -0,0183 | 0,7917 |
| Rv2494 | Rv2494 | 48V>A | 2 | 24 | 4 | 38 | 0,6021 | -0,0183 | 0,7917 |
| Rv2495c | pdhC | 103Y>D | 2 | 24 | 4 | 38 | 0,6021 | -0,0183 | 0,7917 |
| Rv2502c | accD1 | 325F>V | 2 | 24 | 4 | 38 | 0,6021 | -0,0183 | 0,7917 |
| Rv2516c | Rv2516c | 62A>V | 2 | 24 | 4 | 38 | 0,6021 | -0,0183 | 0,7917 |
| Rv2519 | PE26 | 67V>A | 2 | 24 | 4 | 38 | 0,6021 | -0,0183 | 0,7917 |
| Rv2524c | fas | 2771C>R | 2 | 24 | 4 | 38 | 0,6021 | -0,0183 | 0,7917 |
| Rv2562 | Rv2562 | 73A>T | 2 | 24 | 4 | 38 | 0,6021 | -0,0183 | 0,7917 |
| Rv2564 | glnQ | 243M>L | 2 | 24 | 4 | 38 | 0,6021 | -0,0183 | 0,7917 |
| Rv2596 | Rv2596 | 77C>R | 2 | 24 | 4 | 38 | 0,6021 | -0,0183 | 0,7917 |
| Rv2621c | Rv2621c | 110A>V | 2 | 24 | 4 | 38 | 0,6021 | -0,0183 | 0,7917 |
| Rv2629 | Rv2629 | 64D>A | 2 | 24 | 4 | 38 | 0,6021 | -0,0183 | 0,7917 |
| Rv2650c | Rv2650c | 3N>T | 2 | 24 | 4 | 38 | 0,6021 | -0,0183 | 0,7917 |
| Rv2672 | Rv2672 | 317H>D | 2 | 24 | 4 | 38 | 0,6021 | -0,0183 | 0,7917 |
| Rv2685 | arsB1 | 378A>G | 2 | 24 | 4 | 38 | 0,6021 | -0,0183 | 0,7917 |
| Rv2691 | ceoB | 164D>G | 2 | 24 | 4 | 38 | 0,6021 | -0,0183 | 0,7917 |
| Rv2692 | ceoC | 133I>V | 2 | 24 | 4 | 38 | 0,6021 | -0,0183 | 0,7917 |
| Rv2693c | Rv2693c | 126G>R | 2 | 24 | 4 | 38 | 0,6021 | -0,0183 | 0,7917 |
| Rv2712c | Rv2712c | 329T>I | 2 | 24 | 4 | 38 | 0,6021 | -0,0183 | 0,7917 |
| Rv2714 | Rv2714 | 245V>A | 2 | 24 | 4 | 38 | 0,6021 | -0,0183 | 0,7917 |
| Rv2719c | Rv2719c | 124Y>H | 2 | 24 | 4 | 38 | 0,6021 | -0,0183 | 0,7917 |
| Rv2724c | fadE20 | 156V>M | 2 | 24 | 4 | 38 | 0,6021 | -0,0183 | 0,7917 |
| Rv2736c | recX | 59V>L | 2 | 24 | 4 | 38 | 0,6021 | -0,0183 | 0,7917 |

| Rv2770c | PPE44 | 194F>S | 2 | 24 | 4 | 38 | 0,6021 | -0,0183 | 0,7917 |
| --- | --- | --- | --- | --- | --- | --- | --- | --- | --- |
| Rv2805 | Rv2804c | 73G>D | 2 | 24 | 4 | 38 | 0,6021 | -0,0183 | 0,7917 |
| Rv2831 | echA16 | 156D>Y | 2 | 24 | 4 | 38 | 0,6021 | -0,0183 | 0,7917 |
| Rv2838c | Rv2837c | 8E>E | 2 | 24 | 4 | 38 | 0,6021 | -0,0183 | 0,7917 |
| Rv2862c | Rv2862c | 50C>R | 2 | 24 | 4 | 38 | 0,6021 | -0,0183 | 0,7917 |
| Rv2869c | Rv2869c | 259V>F | 2 | 24 | 4 | 38 | 0,6021 | -0,0183 | 0,7917 |
| Rv2891 | Rv2891 | 6A>V | 2 | 24 | 4 | 38 | 0,6021 | -0,0183 | 0,7917 |
| Rv2899c | fdhD | 84A>V | 2 | 24 | 4 | 38 | 0,6021 | -0,0183 | 0,7917 |
| Rv2905 | lppW | 81Q>R | 2 | 24 | 4 | 38 | 0,6021 | -0,0183 | 0,7917 |
| Rv2931 | ppsA | 1194L>R | 2 | 24 | 4 | 38 | 0,6021 | -0,0183 | 0,7917 |
| Rv2940c | mas | 2005T>P | 2 | 24 | 4 | 38 | 0,6021 | -0,0183 | 0,7917 |
| Rv2941 | fadD28 | 436T>A | 2 | 24 | 4 | 38 | 0,6021 | -0,0183 | 0,7917 |
| Rv2952 | Rv2952 | 176G>R | 2 | 24 | 4 | 38 | 0,6021 | -0,0183 | 0,7917 |
| Rv2964 | purU | 143M>L | 2 | 24 | 4 | 38 | 0,6021 | -0,0183 | 0,7917 |
| Rv2971 | Rv2971 | 152N>H | 2 | 24 | 4 | 38 | 0,6021 | -0,0183 | 0,7917 |
| Rv2979c | Rv2979c | 14P>R | 2 | 24 | 4 | 38 | 0,6021 | -0,0183 | 0,7917 |
| Rv3015c | Rv3015c | 167F>C | 2 | 24 | 4 | 38 | 0,6021 | -0,0183 | 0,7917 |
| Rv3047c | Rv3047c | 53T>A | 2 | 24 | 4 | 38 | 0,6021 | -0,0183 | 0,7917 |
| Rv3051c | nrdE | 519Q>R | 2 | 24 | 4 | 38 | 0,6021 | -0,0183 | 0,7917 |
| Rv3059 | cyp136 | 445D>G | 2 | 24 | 4 | 38 | 0,6021 | -0,0183 | 0,7917 |
| Rv3081 | Rv3081 | 220F>L | 2 | 24 | 4 | 38 | 0,6021 | -0,0183 | 0,7917 |
| Rv3087 | Rv3087 | 447L>V | 2 | 24 | 4 | 38 | 0,6021 | -0,0183 | 0,7917 |
| Rv3097c | lipY | 58G>A | 2 | 24 | 4 | 38 | 0,6021 | -0,0183 | 0,7917 |
| Rv3114 | Rv3114 | 11S>P | 2 | 24 | 4 | 38 | 0,6021 | -0,0183 | 0,7917 |
| Rv3144c | PPE52 | 400K>Q | 2 | 24 | 4 | 38 | 0,6021 | -0,0183 | 0,7917 |
| Rv3161c | Rv3161c | 62V>L | 2 | 24 | 4 | 38 | 0,6021 | -0,0183 | 0,7917 |
| Rv3174 | Rv3174 | 28G>S | 2 | 24 | 4 | 38 | 0,6021 | -0,0183 | 0,7917 |
| Rv3199c | nudC | 239P>R | 2 | 24 | 4 | 38 | 0,6021 | -0,0183 | 0,7917 |
| Rv3201c | Rv3201c | 269Q>R | 2 | 24 | 4 | 38 | 0,6021 | -0,0183 | 0,7917 |
| Rv3202c | Rv3202c | 902E>K | 2 | 24 | 4 | 38 | 0,6021 | -0,0183 | 0,7917 |
| Rv3220c | Rv3220c | 96G>R | 2 | 24 | 4 | 38 | 0,6021 | -0,0183 | 0,7917 |
| Rv3236c | Rv3236c | 102T>A | 2 | 24 | 4 | 38 | 0,6021 | -0,0183 | 0,7917 |
| Rv3245c | mtrB | 18P>S | 2 | 24 | 4 | 38 | 0,6021 | -0,0183 | 0,7917 |

| Rv3257c | manB | 206S>L | 2 | 24 | 4 | 38 | 0,6021 | -0,0183 | 0,7917 |
| --- | --- | --- | --- | --- | --- | --- | --- | --- | --- |
| Rv3263 | Rv3263 | 270E>A | 2 | 24 | 4 | 38 | 0,6021 | -0,0183 | 0,7917 |
| Rv3272 | Rv3272 | 181V>M | 2 | 24 | 4 | 38 | 0,6021 | -0,0183 | 0,7917 |
| Rv3282 | maf | 80A>D | 2 | 24 | 4 | 38 | 0,6021 | -0,0183 | 0,7917 |
| Rv3283 | sseA | 276E>K | 2 | 24 | 4 | 38 | 0,6021 | -0,0183 | 0,7917 |
| Rv3293 | pcd | 186C>S | 2 | 24 | 4 | 38 | 0,6021 | -0,0183 | 0,7917 |
| Rv3296 | lhr | 492K>T | 2 | 24 | 4 | 38 | 0,6021 | -0,0183 | 0,7917 |
| Rv3303c | lpdA | 308L>S | 2 | 24 | 4 | 38 | 0,6021 | -0,0183 | 0,7917 |
| Rv3317 | sdhD | 112T>A | 2 | 24 | 4 | 38 | 0,6021 | -0,0183 | 0,7917 |
| Rv3365c | Rv3365c | 687S>R | 2 | 24 | 4 | 38 | 0,6021 | -0,0183 | 0,7917 |
| Rv3365c | Rv3365c | 698Q>R | 2 | 24 | 4 | 38 | 0,6021 | -0,0183 | 0,7917 |
| Rv3389c | Rv3389c | 165P>S | 2 | 24 | 4 | 38 | 0,6021 | -0,0183 | 0,7917 |
| Rv3402c | Rv3402c | 16A>E | 2 | 24 | 4 | 38 | 0,6021 | -0,0183 | 0,7917 |
| Rv3407 | Rv3407 | 84R>C | 2 | 24 | 4 | 38 | 0,6021 | -0,0183 | 0,7917 |
| Rv3411c | guaB2 | 391A>T | 2 | 24 | 4 | 38 | 0,6021 | -0,0183 | 0,7917 |
| Rv3424c | Rv3424c | 96V>A | 2 | 24 | 4 | 38 | 0,6021 | -0,0183 | 0,7917 |
| Rv3429 | PPE59 | 24S>F | 2 | 24 | 4 | 38 | 0,6021 | -0,0183 | 0,7917 |
| Rv3429 | PPE59 | 25A>F | 2 | 24 | 4 | 38 | 0,6021 | -0,0183 | 0,7917 |
| Rv3433c | Rv3433c | 443S>A | 2 | 24 | 4 | 38 | 0,6021 | -0,0183 | 0,7917 |
| Rv3445c | esxU | 63P>S | 2 | 24 | 4 | 38 | 0,6021 | -0,0183 | 0,7917 |
| Rv3446c | Rv3446c | 284R>P | 2 | 24 | 4 | 38 | 0,6021 | -0,0183 | 0,7917 |
| Rv3449 | mycP4 | 87T>A | 2 | 24 | 4 | 38 | 0,6021 | -0,0183 | 0,7917 |
| Rv3476c | kgtP | 81L>F | 2 | 24 | 4 | 38 | 0,6021 | -0,0183 | 0,7917 |
| Rv3478 | PPE60 | 103R>G | 2 | 24 | 4 | 38 | 0,6021 | -0,0183 | 0,7917 |
| Rv3478 | PPE60 | 387P>L | 2 | 24 | 4 | 38 | 0,6021 | -0,0183 | 0,7917 |
| Rv3487c | lipF | 290R>C | 2 | 24 | 4 | 38 | 0,6021 | -0,0183 | 0,7917 |
| Rv3490 | otsA | 334V>L | 2 | 24 | 4 | 38 | 0,6021 | -0,0183 | 0,7917 |
| Rv3490 | otsA | 77E>G | 2 | 24 | 4 | 38 | 0,6021 | -0,0183 | 0,7917 |
| Rv3497c | mce4C | 191R>S | 2 | 24 | 4 | 38 | 0,6021 | -0,0183 | 0,7917 |
| Rv3515c | fadD19 | 55D>Y | 2 | 24 | 4 | 38 | 0,6021 | -0,0183 | 0,7917 |
| Rv3523 | ltp3 | 68F>L | 2 | 24 | 4 | 38 | 0,6021 | -0,0183 | 0,7917 |
| Rv3531c | Rv3531c | 313F>L | 2 | 24 | 4 | 38 | 0,6021 | -0,0183 | 0,7917 |
| Rv3537 | Rv3537 | 409R>S | 2 | 24 | 4 | 38 | 0,6021 | -0,0183 | 0,7917 |

| Rv3540c | ltp2 | 224V>L | 2 | 24 | 4 | 38 | 0,6021 | -0,0183 | 0,7917 |
| --- | --- | --- | --- | --- | --- | --- | --- | --- | --- |
| Rv3554 | fdxB | 125G>E | 2 | 24 | 4 | 38 | 0,6021 | -0,0183 | 0,7917 |
| Rv3563 | fadE32 | 275W>S | 2 | 24 | 4 | 38 | 0,6021 | -0,0183 | 0,7917 |
| Rv3567c | Rv3567c | 179I>T | 2 | 24 | 4 | 38 | 0,6021 | -0,0183 | 0,7917 |
| Rv3586 | Rv3586 | 288T>A | 2 | 24 | 4 | 38 | 0,6021 | -0,0183 | 0,7917 |
| Rv3591c | Rv3591c | 156F>L | 2 | 24 | 4 | 38 | 0,6021 | -0,0183 | 0,7917 |
| Rv3598c | lysS | 454A>T | 2 | 24 | 4 | 38 | 0,6021 | -0,0183 | 0,7917 |
| Rv3618 | Rv3618 | 149E>A | 2 | 24 | 4 | 38 | 0,6021 | -0,0183 | 0,7917 |
| Rv3649 | Rv3649 | 93L>P | 2 | 24 | 4 | 38 | 0,6021 | -0,0183 | 0,7917 |
| Rv3666c | dppA | 4Q>R | 2 | 24 | 4 | 38 | 0,6021 | -0,0183 | 0,7917 |
| Rv3671c | Rv3671c | 363I>V | 2 | 24 | 4 | 38 | 0,6021 | -0,0183 | 0,7917 |
| Rv3674c | nth | 2P>R | 2 | 24 | 4 | 38 | 0,6021 | -0,0183 | 0,7917 |
| Rv3676 | Rv3676 | 45D>N | 2 | 24 | 4 | 38 | 0,6021 | -0,0183 | 0,7917 |
| Rv3680 | Rv3680 | 199G>A | 2 | 24 | 4 | 38 | 0,6021 | -0,0183 | 0,7917 |
| Rv3682 | ponA2 | 619A>T | 2 | 24 | 4 | 38 | 0,6021 | -0,0183 | 0,7917 |
| Rv3691 | Rv3691 | 267T>S | 2 | 24 | 4 | 38 | 0,6021 | -0,0183 | 0,7917 |
| Rv3711c | dnaQ | 76G>D | 2 | 24 | 4 | 38 | 0,6021 | -0,0183 | 0,7917 |
| Rv3715c | recR | 44G>C | 2 | 24 | 4 | 38 | 0,6021 | -0,0183 | 0,7917 |
| Rv3722c | Rv3722c | 158M>T | 2 | 24 | 4 | 38 | 0,6021 | -0,0183 | 0,7917 |
| Rv3724B | cut5b | 83A>S | 2 | 24 | 4 | 38 | 0,6021 | -0,0183 | 0,7917 |
| Rv3725 | Rv3725 | 176V>L | 2 | 24 | 4 | 38 | 0,6021 | -0,0183 | 0,7917 |
| Rv3727 | Rv3727 | 393G>R | 2 | 24 | 4 | 38 | 0,6021 | -0,0183 | 0,7917 |
| Rv3729 | Rv3729 | 269P>S | 2 | 24 | 4 | 38 | 0,6021 | -0,0183 | 0,7917 |
| Rv3729 | Rv3729 | 516Q>H | 2 | 24 | 4 | 38 | 0,6021 | -0,0183 | 0,7917 |
| Rv3758c | proV | 84N>D | 2 | 24 | 4 | 38 | 0,6021 | -0,0183 | 0,7917 |
| Rv3759c | proX | 17W>R | 2 | 24 | 4 | 38 | 0,6021 | -0,0183 | 0,7917 |
| Rv3770c | Rv3770c | 98P>A | 2 | 24 | 4 | 38 | 0,6021 | -0,0183 | 0,7917 |
| Rv3772 | hisC2 | 142T>A | 2 | 24 | 4 | 38 | 0,6021 | -0,0183 | 0,7917 |
| Rv3773c | Rv3773c | 137G>V | 2 | 24 | 4 | 38 | 0,6021 | -0,0183 | 0,7917 |
| Rv3776 | Rv3776 | 112S>L | 2 | 24 | 4 | 38 | 0,6021 | -0,0183 | 0,7917 |
| Rv3782 | Rv3782 | 274V>A | 2 | 24 | 4 | 38 | 0,6021 | -0,0183 | 0,7917 |
| Rv3788 | Rv3788 | 61I>L | 2 | 24 | 4 | 38 | 0,6021 | -0,0183 | 0,7917 |
| Rv3797 | fadE35 | 328S>P | 2 | 24 | 4 | 38 | 0,6021 | -0,0183 | 0,7917 |

| Rv3800c | pks13 | 1014T>M | 2 | 24 | 4 | 38 | 0,6021 | -0,0183 | 0,7917 |
| --- | --- | --- | --- | --- | --- | --- | --- | --- | --- |
| Rv3800c | pks13 | 1646A>D | 2 | 24 | 4 | 38 | 0,6021 | -0,0183 | 0,7917 |
| Rv3801c | fadD32 | 227G>S | 2 | 24 | 4 | 38 | 0,6021 | -0,0183 | 0,7917 |
| Rv3805c | Rv3805c | 397D>G | 2 | 24 | 4 | 38 | 0,6021 | -0,0183 | 0,7917 |
| Rv3820c | papA2 | 466P>L | 2 | 24 | 4 | 38 | 0,6021 | -0,0183 | 0,7917 |
| Rv3826 | fadD23 | 422E>Q | 2 | 24 | 4 | 38 | 0,6021 | -0,0183 | 0,7917 |
| Rv3835 | Rv3835 | 294L>R | 2 | 24 | 4 | 38 | 0,6021 | -0,0183 | 0,7917 |
| Rv3837c | Rv3837c | 15G>D | 2 | 24 | 4 | 38 | 0,6021 | -0,0183 | 0,7917 |
| Rv3839 | Rv3839 | 122P>S | 2 | 24 | 4 | 38 | 0,6021 | -0,0183 | 0,7917 |
| Rv3842c | glpQ1 | 255H>Y | 2 | 24 | 4 | 38 | 0,6021 | -0,0183 | 0,7917 |
| Rv3879c | Rv3879c | 44D>N | 2 | 24 | 4 | 38 | 0,6021 | -0,0183 | 0,7917 |
| Rv3879c | Rv3879c | 660E>A | 2 | 24 | 4 | 38 | 0,6021 | -0,0183 | 0,7917 |
| Rv3898c | Rv3898c | 111*>Q | 2 | 24 | 4 | 38 | 0,6021 | -0,0183 | 0,7917 |
| Rv3908 | Rv3908 | 48R>G | 2 | 24 | 4 | 38 | 0,6021 | -0,0183 | 0,7917 |
| Rv3919c | gidB | 92E>D | 2 | 24 | 4 | 38 | 0,6021 | -0,0183 | 0,7917 |
| Rv0086 | hycQ | 132V>I | 6 | 20 | 11 | 31 | 0,6134 | -0,0311 | 0,8455 |
| Rv0097 | Rv0097 | 251I>T | 6 | 20 | 11 | 31 | 0,6134 | -0,0311 | 0,8455 |
| Rv0158 | Rv0158 | 212K>N | 6 | 20 | 11 | 31 | 0,6134 | -0,0311 | 0,8455 |
| Rv0365c | Rv0365c | 307D>E | 6 | 20 | 11 | 31 | 0,6134 | -0,0311 | 0,8455 |
| Rv0368c | Rv0368c | 121Q>E | 6 | 20 | 11 | 31 | 0,6134 | -0,0311 | 0,8455 |
| Rv0405 | pks6 | 708L>V | 6 | 20 | 11 | 31 | 0,6134 | -0,0311 | 0,8455 |
| Rv0407 | fgd1 | 270K>M | 6 | 20 | 11 | 31 | 0,6134 | -0,0311 | 0,8455 |
| Rv0437c | psd | 84V>M | 6 | 20 | 11 | 31 | 0,6134 | -0,0311 | 0,8455 |
| Rv0741 | Rv0741 | 59N>H | 6 | 20 | 11 | 31 | 0,6134 | -0,0311 | 0,8455 |
| Rv0995 | rimJ | 105Y>C | 6 | 20 | 11 | 31 | 0,6134 | -0,0311 | 0,8455 |
| Rv0995 | rimJ | 72R>L | 6 | 20 | 11 | 31 | 0,6134 | -0,0311 | 0,8455 |
| Rv1016c | lpqT | 72E>A | 6 | 20 | 11 | 31 | 0,6134 | -0,0311 | 0,8455 |
| Rv1029 | kdpA | 555F>L | 6 | 20 | 11 | 31 | 0,6134 | -0,0311 | 0,8455 |
| Rv1249c | Rv1249c | 97A>S | 6 | 20 | 11 | 31 | 0,6134 | -0,0311 | 0,8455 |
| Rv1251c | Rv1251c | 977P>H | 6 | 20 | 11 | 31 | 0,6134 | -0,0311 | 0,8455 |
| Rv1527c | pks5 | 1903L>R | 6 | 20 | 11 | 31 | 0,6134 | -0,0311 | 0,8455 |
| Rv1538c | ansA | 190R>P | 6 | 20 | 11 | 31 | 0,6134 | -0,0311 | 0,8455 |
| Rv1553 | frdB | 157S>L | 6 | 20 | 11 | 31 | 0,6134 | -0,0311 | 0,8455 |

| Rv1571 | Rv1571 | 84R>L | 6 | 20 | 11 | 31 | 0,6134 | -0,0311 | 0,8455 |
| --- | --- | --- | --- | --- | --- | --- | --- | --- | --- |
| Rv1704c | cycA | 477R>G | 6 | 20 | 11 | 31 | 0,6134 | -0,0311 | 0,8455 |
| Rv1760 | Rv1760 | 397M>T | 6 | 20 | 11 | 31 | 0,6134 | -0,0311 | 0,8455 |
| Rv1788 | PE18 | 2S>P | 6 | 20 | 11 | 31 | 0,6134 | -0,0311 | 0,8455 |
| Rv1821 | secA2 | 59S>R | 6 | 20 | 11 | 31 | 0,6134 | -0,0311 | 0,8455 |
| Rv2147c | Rv2147c | 220A>T | 6 | 20 | 11 | 31 | 0,6134 | -0,0311 | 0,8455 |
| Rv2296 | Rv2296 | 257D>G | 6 | 20 | 11 | 31 | 0,6134 | -0,0311 | 0,8455 |
| Rv2394 | ggtB | 569G>A | 6 | 20 | 11 | 31 | 0,6134 | -0,0311 | 0,8455 |
| Rv2473 | Rv2473 | 9A>T | 6 | 20 | 11 | 31 | 0,6134 | -0,0311 | 0,8455 |
| Rv2503c | scoB | 40P>L | 6 | 20 | 11 | 31 | 0,6134 | -0,0311 | 0,8455 |
| Rv2565 | Rv2565 | 187A>E | 6 | 20 | 11 | 31 | 0,6134 | -0,0311 | 0,8455 |
| Rv2709 | Rv2709 | 86S>P | 6 | 20 | 11 | 31 | 0,6134 | -0,0311 | 0,8455 |
| Rv2761c | hsdS | 25G>V | 6 | 20 | 11 | 31 | 0,6134 | -0,0311 | 0,8455 |
| Rv2787 | Rv2787 | 566S>I | 6 | 20 | 11 | 31 | 0,6134 | -0,0311 | 0,8455 |
| Rv2800 | Rv2800 | 348G>S | 6 | 20 | 11 | 31 | 0,6134 | -0,0311 | 0,8455 |
| Rv2875 | mpt70 | 21A>T | 6 | 20 | 11 | 31 | 0,6134 | -0,0311 | 0,8455 |
| Rv2923c | acyP | 4A>V | 6 | 20 | 11 | 31 | 0,6134 | -0,0311 | 0,8455 |
| Rv3037c | Rv3037c | 23A>V | 6 | 20 | 11 | 31 | 0,6134 | -0,0311 | 0,8455 |
| Rv3279c | birA | 225V>I | 6 | 20 | 11 | 31 | 0,6134 | -0,0311 | 0,8455 |
| Rv3306c | amiB1 | 101A>T | 6 | 20 | 11 | 31 | 0,6134 | -0,0311 | 0,8455 |
| Rv3347c | PPE55 | 2800A>V | 6 | 20 | 11 | 31 | 0,6134 | -0,0311 | 0,8455 |
| Rv3515c | fadD19 | 348A>P | 6 | 20 | 11 | 31 | 0,6134 | -0,0311 | 0,8455 |
| Rv3523 | ltp3 | 143Y>* | 6 | 20 | 11 | 31 | 0,6134 | -0,0311 | 0,8455 |
| Rv0046c | ino1 | 190R>G | 9 | 17 | 16 | 26 | 0,6138 | -0,0348 | 0,8603 |
| Rv0048c | Rv0048c | 250V>A | 9 | 17 | 16 | 26 | 0,6138 | -0,0348 | 0,8603 |
| Rv0324 | Rv0324 | 168T>A | 9 | 17 | 16 | 26 | 0,6138 | -0,0348 | 0,8603 |
| Rv0436c | pssA | 167G>V | 9 | 17 | 16 | 26 | 0,6138 | -0,0348 | 0,8603 |
| Rv0619 | galTb | 174T>A | 9 | 17 | 16 | 26 | 0,6138 | -0,0348 | 0,8603 |
| Rv1127c | ppdK | 69G>E | 9 | 17 | 16 | 26 | 0,6138 | -0,0348 | 0,8603 |
| Rv1128c | Rv1128c | 270E>G | 9 | 17 | 16 | 26 | 0,6138 | -0,0348 | 0,8603 |
| Rv1218c | Rv1218c | 243Q>R | 9 | 17 | 16 | 26 | 0,6138 | -0,0348 | 0,8603 |
| Rv1448c | tal | 244T>A | 9 | 17 | 16 | 26 | 0,6138 | -0,0348 | 0,8603 |
| Rv1517 | Rv1517 | 188L>F | 9 | 17 | 16 | 26 | 0,6138 | -0,0348 | 0,8603 |

| Rv1592c | Rv1592c | 322I>V | 9 | 17 | 16 | 26 | 0,6138 | -0,0348 | 0,8603 |
| --- | --- | --- | --- | --- | --- | --- | --- | --- | --- |
| Rv1915 | aceAa | 179G>D | 9 | 17 | 16 | 26 | 0,6138 | -0,0348 | 0,8603 |
| Rv2109c | prcA | 135R>P | 9 | 17 | 16 | 26 | 0,6138 | -0,0348 | 0,8603 |
| Rv2307c | Rv2307c | 24M>T | 9 | 17 | 16 | 26 | 0,6138 | -0,0348 | 0,8603 |
| Rv2316 | uspA | 127V>L | 9 | 17 | 16 | 26 | 0,6138 | -0,0348 | 0,8603 |
| Rv2333c | Rv2333c | 69D>Y | 9 | 17 | 16 | 26 | 0,6138 | -0,0348 | 0,8603 |
| Rv2360c | Rv2360c | 66A>T | 9 | 17 | 16 | 26 | 0,6138 | -0,0348 | 0,8603 |
| Rv2439c | proB | 226A>S | 9 | 17 | 16 | 26 | 0,6138 | -0,0348 | 0,8603 |
| Rv2756c | hsdM | 306L>P | 9 | 17 | 16 | 26 | 0,6138 | -0,0348 | 0,8603 |
| Rv3113 | Rv3113 | 134G>E | 9 | 17 | 16 | 26 | 0,6138 | -0,0348 | 0,8603 |
| Rv3759c | proX | 85L>P | 9 | 17 | 16 | 26 | 0,6138 | -0,0348 | 0,8603 |
| Rv3777 | Rv3777 | 160V>A | 9 | 17 | 16 | 26 | 0,6138 | -0,0348 | 0,8603 |
| Rv3884c | Rv3884c | 215E>G | 9 | 17 | 16 | 26 | 0,6138 | -0,0348 | 0,8603 |
| Rv1037c | esxI | 20Q>L | 4 | 22 | 8 | 34 | 0,6499 | -0,0366 | 0,7727 |
| Rv3467 | Rv3467 | 262G>D | 4 | 22 | 8 | 34 | 0,6499 | -0,0366 | 0,7727 |
| Rv3619c | esxV | 20Q>L | 4 | 22 | 8 | 34 | 0,6499 | -0,0366 | 0,7727 |
| Rv3347c | PPE55 | 2259L>P | 20 | 6 | 34 | 8 | 0,6552 | -0,0403 | 0,7843 |
| Rv0540 | Rv0540 | 28I>M | 5 | 21 | 10 | 32 | 0,6709 | -0,0458 | 0,7619 |
| Rv1226c | Rv1226c | 479D>A | 5 | 21 | 10 | 32 | 0,6709 | -0,0458 | 0,7619 |
| Rv1523 | Rv1523 | 38A>T | 5 | 21 | 10 | 32 | 0,6709 | -0,0458 | 0,7619 |
| Rv2443 | dctA | 88G>S | 5 | 21 | 10 | 32 | 0,6709 | -0,0458 | 0,7619 |
| Rv2621c | Rv2621c | 190A>D | 5 | 21 | 10 | 32 | 0,6709 | -0,0458 | 0,7619 |
| Rv0094c | Rv0094c | 276N>K | 2 | 24 | 5 | 37 | 0,7107 | -0,0421 | 0,6167 |
| Rv0280 | PPE3 | 257E>K | 2 | 24 | 5 | 37 | 0,7107 | -0,0421 | 0,6167 |
| Rv3465 | rmlC | 115E>D | 2 | 24 | 5 | 37 | 0,7107 | -0,0421 | 0,6167 |
| Rv3467 | Rv3467 | 276N>K | 2 | 24 | 5 | 37 | 0,7107 | -0,0421 | 0,6167 |
| Rv3327 | Rv3327 | 111L>M | 7 | 19 | 14 | 28 | 0,7109 | -0,0641 | 0,7368 |
| Rv0070c | glyA | 159V>I | 1 | 25 | 3 | 39 | 0,7128 | -0,0330 | 0,5200 |
| Rv0162c | adhE1 | 163H>Y | 1 | 25 | 3 | 39 | 0,7128 | -0,0330 | 0,5200 |
| Rv0194 | Rv0194 | 51A>V | 1 | 25 | 3 | 39 | 0,7128 | -0,0330 | 0,5200 |
| Rv0198c | Rv0198c | 609R>P | 1 | 25 | 3 | 39 | 0,7128 | -0,0330 | 0,5200 |
| Rv0278c | PE_PGRS3 | 957P>L | 1 | 25 | 3 | 39 | 0,7128 | -0,0330 | 0,5200 |
| Rv0278c | PE_PGRS3 | 958*>L | 1 | 25 | 3 | 39 | 0,7128 | -0,0330 | 0,5200 |

| Rv0304c | PPE5 | 159G>A | 1 | 25 | 3 | 39 | 0,7128 | -0,0330 | 0,5200 |
| --- | --- | --- | --- | --- | --- | --- | --- | --- | --- |
| Rv0376c | Rv0376c | 96R>W | 1 | 25 | 3 | 39 | 0,7128 | -0,0330 | 0,5200 |
| Rv0380c | Rv0380c | 171A>T | 1 | 25 | 3 | 39 | 0,7128 | -0,0330 | 0,5200 |
| Rv0508 | Rv0508 | 34D>A | 1 | 25 | 3 | 39 | 0,7128 | -0,0330 | 0,5200 |
| Rv0656c | Rv0656c | 89I>V | 1 | 25 | 3 | 39 | 0,7128 | -0,0330 | 0,5200 |
| Rv0755c | PPE12 | 104G>D | 1 | 25 | 3 | 39 | 0,7128 | -0,0330 | 0,5200 |
| Rv0770 | Rv0770 | 180N>S | 1 | 25 | 3 | 39 | 0,7128 | -0,0330 | 0,5200 |
| Rv0980c | PE_PGRS18 | 270A>T | 1 | 25 | 3 | 39 | 0,7128 | -0,0330 | 0,5200 |
| Rv0980c | PE_PGRS18 | 73E>Q | 1 | 25 | 3 | 39 | 0,7128 | -0,0330 | 0,5200 |
| Rv0980c | PE_PGRS18 | 83S>G | 1 | 25 | 3 | 39 | 0,7128 | -0,0330 | 0,5200 |
| Rv0992c | Rv0992c | 189G>V | 1 | 25 | 3 | 39 | 0,7128 | -0,0330 | 0,5200 |
| Rv1067c | PE_PGRS19 | 91E>K | 1 | 25 | 3 | 39 | 0,7128 | -0,0330 | 0,5200 |
| Rv1142c | echA10 | 147L>F | 1 | 25 | 3 | 39 | 0,7128 | -0,0330 | 0,5200 |
| Rv1142c | echA10 | 220N>T | 1 | 25 | 3 | 39 | 0,7128 | -0,0330 | 0,5200 |
| Rv1144 | Rv1144 | 240R>C | 1 | 25 | 3 | 39 | 0,7128 | -0,0330 | 0,5200 |
| Rv1148c | Rv1148c | 445H>K | 1 | 25 | 3 | 39 | 0,7128 | -0,0330 | 0,5200 |
| Rv1148c | Rv1148c | 470A>E | 1 | 25 | 3 | 39 | 0,7128 | -0,0330 | 0,5200 |
| Rv1148c | Rv1148c | 476Q>P | 1 | 25 | 3 | 39 | 0,7128 | -0,0330 | 0,5200 |
| Rv1163 | narJ | 195P>L | 1 | 25 | 3 | 39 | 0,7128 | -0,0330 | 0,5200 |
| Rv1175c | fadH | 569K>T | 1 | 25 | 3 | 39 | 0,7128 | -0,0330 | 0,5200 |
| Rv1212c | Rv1212c | 234A>E | 1 | 25 | 3 | 39 | 0,7128 | -0,0330 | 0,5200 |
| Rv1223 | htrA | 466S>G | 1 | 25 | 3 | 39 | 0,7128 | -0,0330 | 0,5200 |
| Rv1264 | Rv1264 | 126Q>* | 1 | 25 | 3 | 39 | 0,7128 | -0,0330 | 0,5200 |
| Rv1281c | oppD | 351R>Q | 1 | 25 | 3 | 39 | 0,7128 | -0,0330 | 0,5200 |
| Rv1284 | Rv1284 | 105I>V | 1 | 25 | 3 | 39 | 0,7128 | -0,0330 | 0,5200 |
| Rv1292 | argS | 354A>V | 1 | 25 | 3 | 39 | 0,7128 | -0,0330 | 0,5200 |
| Rv1372 | Rv1372 | 164S>P | 1 | 25 | 3 | 39 | 0,7128 | -0,0330 | 0,5200 |
| Rv1463 | Rv1463 | 18E>D | 1 | 25 | 3 | 39 | 0,7128 | -0,0330 | 0,5200 |
| Rv1497 | lipL | 41S>G | 1 | 25 | 3 | 39 | 0,7128 | -0,0330 | 0,5200 |
| Rv1502 | Rv1502 | 99L>I | 1 | 25 | 3 | 39 | 0,7128 | -0,0330 | 0,5200 |
| Rv1527c | pks5 | 1977Y>C | 1 | 25 | 3 | 39 | 0,7128 | -0,0330 | 0,5200 |
| Rv1540 | Rv1540 | 245H>D | 1 | 25 | 3 | 39 | 0,7128 | -0,0330 | 0,5200 |
| Rv1668c | Rv1668c | 169W>R | 1 | 25 | 3 | 39 | 0,7128 | -0,0330 | 0,5200 |

| Rv1695 | ppnK | 277T>A | 1 | 25 | 3 | 39 | 0,7128 | -0,0330 | 0,5200 |
| --- | --- | --- | --- | --- | --- | --- | --- | --- | --- |
| Rv1695 | ppnK | 282A>V | 1 | 25 | 3 | 39 | 0,7128 | -0,0330 | 0,5200 |
| Rv1735c | Rv1735c | 26T>A | 1 | 25 | 3 | 39 | 0,7128 | -0,0330 | 0,5200 |
| Rv1768 | PE_PGRS31 | 75Q>P | 1 | 25 | 3 | 39 | 0,7128 | -0,0330 | 0,5200 |
| Rv1834 | Rv1834 | 75R>C | 1 | 25 | 3 | 39 | 0,7128 | -0,0330 | 0,5200 |
| Rv1921c | lppF | 159G>E | 1 | 25 | 3 | 39 | 0,7128 | -0,0330 | 0,5200 |
| Rv2048c | pks12 | 3768V>L | 1 | 25 | 3 | 39 | 0,7128 | -0,0330 | 0,5200 |
| Rv2103c | Rv2103c | 109A>T | 1 | 25 | 3 | 39 | 0,7128 | -0,0330 | 0,5200 |
| Rv2185c | TB16.3 | 125K>R | 1 | 25 | 3 | 39 | 0,7128 | -0,0330 | 0,5200 |
| Rv2476c | gdh | 787R>W | 1 | 25 | 3 | 39 | 0,7128 | -0,0330 | 0,5200 |
| Rv2622 | Rv2622 | 1M>T | 1 | 25 | 3 | 39 | 0,7128 | -0,0330 | 0,5200 |
| Rv2832c | ugpC | 133P>S | 1 | 25 | 3 | 39 | 0,7128 | -0,0330 | 0,5200 |
| Rv2868c | ispG | 298T>A | 1 | 25 | 3 | 39 | 0,7128 | -0,0330 | 0,5200 |
| Rv2885c | Rv2885c | 291V>G | 1 | 25 | 3 | 39 | 0,7128 | -0,0330 | 0,5200 |
| Rv2894c | xerC | 195V>I | 1 | 25 | 3 | 39 | 0,7128 | -0,0330 | 0,5200 |
| Rv3008 | Rv3008 | 112A>T | 1 | 25 | 3 | 39 | 0,7128 | -0,0330 | 0,5200 |
| Rv3024c | mnmA | 290P>L | 1 | 25 | 3 | 39 | 0,7128 | -0,0330 | 0,5200 |
| Rv3078 | hab | 15I>N | 1 | 25 | 3 | 39 | 0,7128 | -0,0330 | 0,5200 |
| Rv3111 | moaC | 59M>I | 1 | 25 | 3 | 39 | 0,7128 | -0,0330 | 0,5200 |
| Rv3119 | moaE1 | 4V>M | 1 | 25 | 3 | 39 | 0,7128 | -0,0330 | 0,5200 |
| Rv3193c | Rv3193c | 902T>S | 1 | 25 | 3 | 39 | 0,7128 | -0,0330 | 0,5200 |
| Rv3209 | Rv3209 | 102V>I | 1 | 25 | 3 | 39 | 0,7128 | -0,0330 | 0,5200 |
| Rv3225c | Rv3225c | 23D>N | 1 | 25 | 3 | 39 | 0,7128 | -0,0330 | 0,5200 |
| Rv3299c | atsB | 223G>R | 1 | 25 | 3 | 39 | 0,7128 | -0,0330 | 0,5200 |
| Rv3345c | PE_PGRS50 | 505D>G | 1 | 25 | 3 | 39 | 0,7128 | -0,0330 | 0,5200 |
| Rv3414c | sigD | 89P>R | 1 | 25 | 3 | 39 | 0,7128 | -0,0330 | 0,5200 |
| Rv3438 | Rv3438 | 111V>M | 1 | 25 | 3 | 39 | 0,7128 | -0,0330 | 0,5200 |
| Rv3469c | mhpE | 43A>E | 1 | 25 | 3 | 39 | 0,7128 | -0,0330 | 0,5200 |
| Rv3550 | echA20 | 17V>A | 1 | 25 | 3 | 39 | 0,7128 | -0,0330 | 0,5200 |
| Rv3566c | nat | 27D>N | 1 | 25 | 3 | 39 | 0,7128 | -0,0330 | 0,5200 |
| Rv3651 | Rv3651 | 73V>A | 1 | 25 | 3 | 39 | 0,7128 | -0,0330 | 0,5200 |
| Rv3666c | dppA | 484P>S | 1 | 25 | 3 | 39 | 0,7128 | -0,0330 | 0,5200 |
| Rv3729 | Rv3729 | 555G>R | 1 | 25 | 3 | 39 | 0,7128 | -0,0330 | 0,5200 |

| Rv3888c | Rv3888c | 29D>Y | 1 | 25 | 3 | 39 | 0,7128 | -0,0330 | 0,5200 |
| --- | --- | --- | --- | --- | --- | --- | --- | --- | --- |
| Rv3897c | Rv3897c | 74G>V | 1 | 25 | 3 | 39 | 0,7128 | -0,0330 | 0,5200 |
| Rv3907c | pcnA | 472S>C | 1 | 25 | 3 | 39 | 0,7128 | -0,0330 | 0,5200 |
| Rv1044 | Rv1044 | 199R>C | 3 | 23 | 7 | 35 | 0,7191 | -0,0513 | 0,6522 |
| Rv1205 | Rv1205 | 95M>V | 3 | 23 | 7 | 35 | 0,7191 | -0,0513 | 0,6522 |
| Rv1760 | Rv1760 | 260P>S | 3 | 23 | 7 | 35 | 0,7191 | -0,0513 | 0,6522 |
| Rv1920 | Rv1920 | 115T>S | 3 | 23 | 7 | 35 | 0,7191 | -0,0513 | 0,6522 |
| Rv2424c | Rv2424c | 275T>I | 3 | 23 | 7 | 35 | 0,7191 | -0,0513 | 0,6522 |
| Rv2424c | Rv2424c | 276T>A | 3 | 23 | 7 | 35 | 0,7191 | -0,0513 | 0,6522 |
| Rv2807 | Rv2807 | 77D>G | 3 | 23 | 7 | 35 | 0,7191 | -0,0513 | 0,6522 |
| Rv2839c | infB | 235G>D | 3 | 23 | 7 | 35 | 0,7191 | -0,0513 | 0,6522 |
| Rv3910 | Rv3910 | 1088V>L | 3 | 23 | 7 | 35 | 0,7191 | -0,0513 | 0,6522 |
| Rv0151c | PE1 | 123N>K | 4 | 22 | 9 | 33 | 0,7310 | -0,0604 | 0,6667 |
| Rv3343c | PPE54 | 103E>A | 4 | 22 | 9 | 33 | 0,7310 | -0,0604 | 0,6667 |
| Rv3466 | Rv3466 | 131V>I | 4 | 22 | 9 | 33 | 0,7310 | -0,0604 | 0,6667 |
| Rv0001 | dnaA | 483E>A | 0 | 26 | 1 | 41 | 0,7860 | -0,0238 | 0,0000 |
| Rv0005 | gyrB | 310I>M | 0 | 26 | 1 | 41 | 0,7860 | -0,0238 | 0,0000 |
| Rv0005 | gyrB | 439P>R | 0 | 26 | 1 | 41 | 0,7860 | -0,0238 | 0,0000 |
| Rv0005 | gyrB | 700S>T | 0 | 26 | 1 | 41 | 0,7860 | -0,0238 | 0,0000 |
| Rv0006 | gyrA | 411S>A | 0 | 26 | 1 | 41 | 0,7860 | -0,0238 | 0,0000 |
| Rv0011c | Rv0011c | 8K>N | 0 | 26 | 1 | 41 | 0,7860 | -0,0238 | 0,0000 |
| Rv0014c | pknB | 570F>L | 0 | 26 | 1 | 41 | 0,7860 | -0,0238 | 0,0000 |
| Rv0015c | pknA | 287P>A | 0 | 26 | 1 | 41 | 0,7860 | -0,0238 | 0,0000 |
| Rv0016c | pbpA | 68T>N | 0 | 26 | 1 | 41 | 0,7860 | -0,0238 | 0,0000 |
| Rv0020c | TB39.8 | 188G>R | 0 | 26 | 1 | 41 | 0,7860 | -0,0238 | 0,0000 |
| Rv0023 | Rv0023 | 160V>A | 0 | 26 | 1 | 41 | 0,7860 | -0,0238 | 0,0000 |
| Rv0026 | Rv0026 | 207A>V | 0 | 26 | 1 | 41 | 0,7860 | -0,0238 | 0,0000 |
| Rv0026 | Rv0026 | 214Q>R | 0 | 26 | 1 | 41 | 0,7860 | -0,0238 | 0,0000 |
| Rv0026 | Rv0026 | 259T>A | 0 | 26 | 1 | 41 | 0,7860 | -0,0238 | 0,0000 |
| Rv0027 | Rv0027 | 68Q>R | 0 | 26 | 1 | 41 | 0,7860 | -0,0238 | 0,0000 |
| Rv0032 | bioF2 | 31A>V | 0 | 26 | 1 | 41 | 0,7860 | -0,0238 | 0,0000 |
| Rv0032 | bioF2 | 342M>V | 0 | 26 | 1 | 41 | 0,7860 | -0,0238 | 0,0000 |
| Rv0032 | bioF2 | 641H>R | 0 | 26 | 1 | 41 | 0,7860 | -0,0238 | 0,0000 |

| Rv0032 | bioF2 | 767Q>E | 0 | 26 | 1 | 41 | 0,7860 | -0,0238 | 0,0000 |
| --- | --- | --- | --- | --- | --- | --- | --- | --- | --- |
| Rv0041 | leuS | 206A>V | 0 | 26 | 1 | 41 | 0,7860 | -0,0238 | 0,0000 |
| Rv0041 | leuS | 756A>V | 0 | 26 | 1 | 41 | 0,7860 | -0,0238 | 0,0000 |
| Rv0043c | Rv0043c | 112V>L | 0 | 26 | 1 | 41 | 0,7860 | -0,0238 | 0,0000 |
| Rv0044c | Rv0044c | 193Y>C | 0 | 26 | 1 | 41 | 0,7860 | -0,0238 | 0,0000 |
| Rv0046c | ino1 | 163D>N | 0 | 26 | 1 | 41 | 0,7860 | -0,0238 | 0,0000 |
| Rv0046c | ino1 | 337V>M | 0 | 26 | 1 | 41 | 0,7860 | -0,0238 | 0,0000 |
| Rv0048c | Rv0048c | 1V>A | 0 | 26 | 1 | 41 | 0,7860 | -0,0238 | 0,0000 |
| Rv0048c | Rv0048c | 23D>N | 0 | 26 | 1 | 41 | 0,7860 | -0,0238 | 0,0000 |
| Rv0049 | Rv0049 | 118P>L | 0 | 26 | 1 | 41 | 0,7860 | -0,0238 | 0,0000 |
| Rv0052 | Rv0052 | 190R>L | 0 | 26 | 1 | 41 | 0,7860 | -0,0238 | 0,0000 |
| Rv0056 | rplI | 133S>P | 0 | 26 | 1 | 41 | 0,7860 | -0,0238 | 0,0000 |
| Rv0058 | dnaB | 115N>S | 0 | 26 | 1 | 41 | 0,7860 | -0,0238 | 0,0000 |
| Rv0062 | celA1 | 348R>L | 0 | 26 | 1 | 41 | 0,7860 | -0,0238 | 0,0000 |
| Rv0063 | Rv0063 | 436V>A | 0 | 26 | 1 | 41 | 0,7860 | -0,0238 | 0,0000 |
| Rv0063 | Rv0063 | 99K>Q | 0 | 26 | 1 | 41 | 0,7860 | -0,0238 | 0,0000 |
| Rv0064 | Rv0064 | 826H>R | 0 | 26 | 1 | 41 | 0,7860 | -0,0238 | 0,0000 |
| Rv0073 | Rv0073 | 144L>V | 0 | 26 | 1 | 41 | 0,7860 | -0,0238 | 0,0000 |
| Rv0073 | Rv0073 | 46K>T | 0 | 26 | 1 | 41 | 0,7860 | -0,0238 | 0,0000 |
| Rv0075 | Rv0075 | 164S>* | 0 | 26 | 1 | 41 | 0,7860 | -0,0238 | 0,0000 |
| Rv0075 | Rv0075 | 61P>L | 0 | 26 | 1 | 41 | 0,7860 | -0,0238 | 0,0000 |
| Rv0078 | Rv0078 | 14A>E | 0 | 26 | 1 | 41 | 0,7860 | -0,0238 | 0,0000 |
| Rv0079 | Rv0079 | 95D>H | 0 | 26 | 1 | 41 | 0,7860 | -0,0238 | 0,0000 |
| Rv0085 | hycP | 204R>H | 0 | 26 | 1 | 41 | 0,7860 | -0,0238 | 0,0000 |
| Rv0091 | mtn | 247R>C | 0 | 26 | 1 | 41 | 0,7860 | -0,0238 | 0,0000 |
| Rv0093c | Rv0093c | 188P>A | 0 | 26 | 1 | 41 | 0,7860 | -0,0238 | 0,0000 |
| Rv0093c | Rv0093c | 93A>P | 0 | 26 | 1 | 41 | 0,7860 | -0,0238 | 0,0000 |
| Rv0095c | Rv0095c | 101T>A | 0 | 26 | 1 | 41 | 0,7860 | -0,0238 | 0,0000 |
| Rv0099 | fadD10 | 250T>I | 0 | 26 | 1 | 41 | 0,7860 | -0,0238 | 0,0000 |
| Rv0101 | nrp | 2300L>V | 0 | 26 | 1 | 41 | 0,7860 | -0,0238 | 0,0000 |
| Rv0101 | nrp | 908G>R | 0 | 26 | 1 | 41 | 0,7860 | -0,0238 | 0,0000 |
| Rv0103c | ctpB | 135Y>* | 0 | 26 | 1 | 41 | 0,7860 | -0,0238 | 0,0000 |
| Rv0103c | ctpB | 406L>I | 0 | 26 | 1 | 41 | 0,7860 | -0,0238 | 0,0000 |

| Rv0103c | ctpB | 448T>I | 0 | 26 | 1 | 41 | 0,7860 | -0,0238 | 0,0000 |
| --- | --- | --- | --- | --- | --- | --- | --- | --- | --- |
| Rv0104 | Rv0104 | 495H>Y | 0 | 26 | 1 | 41 | 0,7860 | -0,0238 | 0,0000 |
| Rv0106 | Rv0106 | 177P>L | 0 | 26 | 1 | 41 | 0,7860 | -0,0238 | 0,0000 |
| Rv0110 | Rv0110 | 62R>W | 0 | 26 | 1 | 41 | 0,7860 | -0,0238 | 0,0000 |
| Rv0112 | gca | 66D>N | 0 | 26 | 1 | 41 | 0,7860 | -0,0238 | 0,0000 |
| Rv0118c | oxcA | 307V>I | 0 | 26 | 1 | 41 | 0,7860 | -0,0238 | 0,0000 |
| Rv0124 | PE_PGRS2 | 67A>T | 0 | 26 | 1 | 41 | 0,7860 | -0,0238 | 0,0000 |
| Rv0126 | treS | 528V>A | 0 | 26 | 1 | 41 | 0,7860 | -0,0238 | 0,0000 |
| Rv0131c | fadE1 | 446E>K | 0 | 26 | 1 | 41 | 0,7860 | -0,0238 | 0,0000 |
| Rv0136 | cyp138 | 322A>G | 0 | 26 | 1 | 41 | 0,7860 | -0,0238 | 0,0000 |
| Rv0136 | cyp138 | 402L>R | 0 | 26 | 1 | 41 | 0,7860 | -0,0238 | 0,0000 |
| Rv0139 | Rv0139 | 12G>S | 0 | 26 | 1 | 41 | 0,7860 | -0,0238 | 0,0000 |
| Rv0139 | Rv0139 | 182G>D | 0 | 26 | 1 | 41 | 0,7860 | -0,0238 | 0,0000 |
| Rv0140 | Rv0140 | 79S>T | 0 | 26 | 1 | 41 | 0,7860 | -0,0238 | 0,0000 |
| Rv0154c | fadE2 | 147T>I | 0 | 26 | 1 | 41 | 0,7860 | -0,0238 | 0,0000 |
| Rv0155 | pntAa | 344G>S | 0 | 26 | 1 | 41 | 0,7860 | -0,0238 | 0,0000 |
| Rv0157 | pntB | 114F>L | 0 | 26 | 1 | 41 | 0,7860 | -0,0238 | 0,0000 |
| Rv0157 | pntB | 50L>S | 0 | 26 | 1 | 41 | 0,7860 | -0,0238 | 0,0000 |
| Rv0159c | PE3 | 115S>* | 0 | 26 | 1 | 41 | 0,7860 | -0,0238 | 0,0000 |
| Rv0159c | PE3 | 196D>E | 0 | 26 | 1 | 41 | 0,7860 | -0,0238 | 0,0000 |
| Rv0166 | fadD5 | 159D>E | 0 | 26 | 1 | 41 | 0,7860 | -0,0238 | 0,0000 |
| Rv0168 | yrbE1B | 282D>E | 0 | 26 | 1 | 41 | 0,7860 | -0,0238 | 0,0000 |
| Rv0170 | mce1B | 243L>F | 0 | 26 | 1 | 41 | 0,7860 | -0,0238 | 0,0000 |
| Rv0171 | mce1C | 463G>W | 0 | 26 | 1 | 41 | 0,7860 | -0,0238 | 0,0000 |
| Rv0172 | mce1D | 367L>F | 0 | 26 | 1 | 41 | 0,7860 | -0,0238 | 0,0000 |
| Rv0174 | mce1F | 256G>D | 0 | 26 | 1 | 41 | 0,7860 | -0,0238 | 0,0000 |
| Rv0174 | mce1F | 9Q>H | 0 | 26 | 1 | 41 | 0,7860 | -0,0238 | 0,0000 |
| Rv0175 | Rv0175 | 140Q>R | 0 | 26 | 1 | 41 | 0,7860 | -0,0238 | 0,0000 |
| Rv0176 | Rv0176 | 283P>L | 0 | 26 | 1 | 41 | 0,7860 | -0,0238 | 0,0000 |
| Rv0176 | Rv0176 | 72L>P | 0 | 26 | 1 | 41 | 0,7860 | -0,0238 | 0,0000 |
| Rv0183 | Rv0183 | 224T>I | 0 | 26 | 1 | 41 | 0,7860 | -0,0238 | 0,0000 |
| Rv0186 | bglS | 134A>V | 0 | 26 | 1 | 41 | 0,7860 | -0,0238 | 0,0000 |
| Rv0186 | bglS | 471E>K | 0 | 26 | 1 | 41 | 0,7860 | -0,0238 | 0,0000 |

| Rv0189c | ilvD | 257A>V | 0 | 26 | 1 | 41 | 0,7860 | -0,0238 | 0,0000 |
| --- | --- | --- | --- | --- | --- | --- | --- | --- | --- |
| Rv0191 | Rv0191 | 65Y>F | 0 | 26 | 1 | 41 | 0,7860 | -0,0238 | 0,0000 |
| Rv0193c | Rv0193c | 292E>D | 0 | 26 | 1 | 41 | 0,7860 | -0,0238 | 0,0000 |
| Rv0194 | Rv0194 | 277A>V | 0 | 26 | 1 | 41 | 0,7860 | -0,0238 | 0,0000 |
| Rv0197 | Rv0197 | 131R>W | 0 | 26 | 1 | 41 | 0,7860 | -0,0238 | 0,0000 |
| Rv0197 | Rv0197 | 369D>H | 0 | 26 | 1 | 41 | 0,7860 | -0,0238 | 0,0000 |
| Rv0198c | Rv0198c | 342K>E | 0 | 26 | 1 | 41 | 0,7860 | -0,0238 | 0,0000 |
| Rv0208c | trmB | 179L>M | 0 | 26 | 1 | 41 | 0,7860 | -0,0238 | 0,0000 |
| Rv0210 | Rv0210 | 127R>C | 0 | 26 | 1 | 41 | 0,7860 | -0,0238 | 0,0000 |
| Rv0211 | pckA | 127V>M | 0 | 26 | 1 | 41 | 0,7860 | -0,0238 | 0,0000 |
| Rv0211 | pckA | 368D>A | 0 | 26 | 1 | 41 | 0,7860 | -0,0238 | 0,0000 |
| Rv0212c | nadR | 41E>Q | 0 | 26 | 1 | 41 | 0,7860 | -0,0238 | 0,0000 |
| Rv0217c | lipW | 102G>D | 0 | 26 | 1 | 41 | 0,7860 | -0,0238 | 0,0000 |
| Rv0218 | Rv0218 | 203H>D | 0 | 26 | 1 | 41 | 0,7860 | -0,0238 | 0,0000 |
| Rv0233 | nrdB | 220D>A | 0 | 26 | 1 | 41 | 0,7860 | -0,0238 | 0,0000 |
| Rv0234c | gabD1 | 97S>T | 0 | 26 | 1 | 41 | 0,7860 | -0,0238 | 0,0000 |
| Rv0235c | Rv0235c | 139L>P | 0 | 26 | 1 | 41 | 0,7860 | -0,0238 | 0,0000 |
| Rv0236c | Rv0236c | 1169T>S | 0 | 26 | 1 | 41 | 0,7860 | -0,0238 | 0,0000 |
| Rv0236c | Rv0236c | 1198E>G | 0 | 26 | 1 | 41 | 0,7860 | -0,0238 | 0,0000 |
| Rv0236c | Rv0236c | 285G>R | 0 | 26 | 1 | 41 | 0,7860 | -0,0238 | 0,0000 |
| Rv0239 | Rv0239 | 51T>A | 0 | 26 | 1 | 41 | 0,7860 | -0,0238 | 0,0000 |
| Rv0241c | Rv0241c | 31T>P | 0 | 26 | 1 | 41 | 0,7860 | -0,0238 | 0,0000 |
| Rv0244c | fadE5 | 522V>F | 0 | 26 | 1 | 41 | 0,7860 | -0,0238 | 0,0000 |
| Rv0245 | Rv0245 | 43V>I | 0 | 26 | 1 | 41 | 0,7860 | -0,0238 | 0,0000 |
| Rv0252 | nirB | 182Q>E | 0 | 26 | 1 | 41 | 0,7860 | -0,0238 | 0,0000 |
| Rv0253 | nirD | 49G>S | 0 | 26 | 1 | 41 | 0,7860 | -0,0238 | 0,0000 |
| Rv0253 | nirD | 6D>E | 0 | 26 | 1 | 41 | 0,7860 | -0,0238 | 0,0000 |
| Rv0255c | cobQ1 | 23C>R | 0 | 26 | 1 | 41 | 0,7860 | -0,0238 | 0,0000 |
| Rv0259c | Rv0259c | 171R>W | 0 | 26 | 1 | 41 | 0,7860 | -0,0238 | 0,0000 |
| Rv0259c | Rv0259c | 94G>R | 0 | 26 | 1 | 41 | 0,7860 | -0,0238 | 0,0000 |
| Rv0260c | Rv0260c | 341D>N | 0 | 26 | 1 | 41 | 0,7860 | -0,0238 | 0,0000 |
| Rv0265c | Rv0265c | 235A>D | 0 | 26 | 1 | 41 | 0,7860 | -0,0238 | 0,0000 |
| Rv0266c | oplA | 386A>T | 0 | 26 | 1 | 41 | 0,7860 | -0,0238 | 0,0000 |

| Rv0266c | oplA | 67M>L | 0 | 26 | 1 | 41 | 0,7860 | -0,0238 | 0,0000 |
| --- | --- | --- | --- | --- | --- | --- | --- | --- | --- |
| Rv0267 | narU | 174F>L | 0 | 26 | 1 | 41 | 0,7860 | -0,0238 | 0,0000 |
| Rv0269c | Rv0269c | 132D>N | 0 | 26 | 1 | 41 | 0,7860 | -0,0238 | 0,0000 |
| Rv0274 | Rv0274 | 151F>L | 0 | 26 | 1 | 41 | 0,7860 | -0,0238 | 0,0000 |
| Rv0278c | PE_PGRS3 | 363L>P | 0 | 26 | 1 | 41 | 0,7860 | -0,0238 | 0,0000 |
| Rv0278c | PE_PGRS3 | 656G>S | 0 | 26 | 1 | 41 | 0,7860 | -0,0238 | 0,0000 |
| Rv0283 | Rv0283 | 470D>H | 0 | 26 | 1 | 41 | 0,7860 | -0,0238 | 0,0000 |
| Rv0291 | mycP3 | 32D>A | 0 | 26 | 1 | 41 | 0,7860 | -0,0238 | 0,0000 |
| Rv0296c | Rv0296c | 41L>V | 0 | 26 | 1 | 41 | 0,7860 | -0,0238 | 0,0000 |
| Rv0304c | PPE5 | 1043A>V | 0 | 26 | 1 | 41 | 0,7860 | -0,0238 | 0,0000 |
| Rv0304c | PPE5 | 1843L>P | 0 | 26 | 1 | 41 | 0,7860 | -0,0238 | 0,0000 |
| Rv0304c | PPE5 | 1955I>T | 0 | 26 | 1 | 41 | 0,7860 | -0,0238 | 0,0000 |
| Rv0304c | PPE5 | 1982S>A | 0 | 26 | 1 | 41 | 0,7860 | -0,0238 | 0,0000 |
| Rv0305c | PPE6 | 75W>S | 0 | 26 | 1 | 41 | 0,7860 | -0,0238 | 0,0000 |
| Rv0305c | PPE6 | 890R>C | 0 | 26 | 1 | 41 | 0,7860 | -0,0238 | 0,0000 |
| Rv0305c | PPE6 | 939H>Y | 0 | 26 | 1 | 41 | 0,7860 | -0,0238 | 0,0000 |
| Rv0306 | Rv0306 | 52P>L | 0 | 26 | 1 | 41 | 0,7860 | -0,0238 | 0,0000 |
| Rv0312 | Rv0312 | 565V>L | 0 | 26 | 1 | 41 | 0,7860 | -0,0238 | 0,0000 |
| Rv0317c | glpQ2 | 200P>R | 0 | 26 | 1 | 41 | 0,7860 | -0,0238 | 0,0000 |
| Rv0320 | Rv0320 | 149P>S | 0 | 26 | 1 | 41 | 0,7860 | -0,0238 | 0,0000 |
| Rv0323c | Rv0323c | 41L>P | 0 | 26 | 1 | 41 | 0,7860 | -0,0238 | 0,0000 |
| Rv0331 | Rv0331 | 70G>S | 0 | 26 | 1 | 41 | 0,7860 | -0,0238 | 0,0000 |
| Rv0331 | Rv0331 | 99A>V | 0 | 26 | 1 | 41 | 0,7860 | -0,0238 | 0,0000 |
| Rv0334 | rmlA | 137A>G | 0 | 26 | 1 | 41 | 0,7860 | -0,0238 | 0,0000 |
| Rv0339c | Rv0339c | 149V>I | 0 | 26 | 1 | 41 | 0,7860 | -0,0238 | 0,0000 |
| Rv0355c | PPE8 | 1244G>D | 0 | 26 | 1 | 41 | 0,7860 | -0,0238 | 0,0000 |
| Rv0355c | PPE8 | 1258G>D | 0 | 26 | 1 | 41 | 0,7860 | -0,0238 | 0,0000 |
| Rv0355c | PPE8 | 2364N>T | 0 | 26 | 1 | 41 | 0,7860 | -0,0238 | 0,0000 |
| Rv0372c | Rv0372c | 103L>P | 0 | 26 | 1 | 41 | 0,7860 | -0,0238 | 0,0000 |
| Rv0381c | Rv0381c | 141V>A | 0 | 26 | 1 | 41 | 0,7860 | -0,0238 | 0,0000 |
| Rv0386 | Rv0386 | 384L>V | 0 | 26 | 1 | 41 | 0,7860 | -0,0238 | 0,0000 |
| Rv0386 | Rv0386 | 638E>G | 0 | 26 | 1 | 41 | 0,7860 | -0,0238 | 0,0000 |
| Rv0392c | ndhA | 145L>V | 0 | 26 | 1 | 41 | 0,7860 | -0,0238 | 0,0000 |

| Rv0393 | Rv0393 | 329C>W | 0 | 26 | 1 | 41 | 0,7860 | -0,0238 | 0,0000 |
| --- | --- | --- | --- | --- | --- | --- | --- | --- | --- |
| Rv0393 | Rv0393 | 348N>D | 0 | 26 | 1 | 41 | 0,7860 | -0,0238 | 0,0000 |
| Rv0400c | fadE7 | 332L>R | 0 | 26 | 1 | 41 | 0,7860 | -0,0238 | 0,0000 |
| Rv0402c | mmpL1 | 126A>E | 0 | 26 | 1 | 41 | 0,7860 | -0,0238 | 0,0000 |
| Rv0402c | mmpL1 | 773C>Y | 0 | 26 | 1 | 41 | 0,7860 | -0,0238 | 0,0000 |
| Rv0404 | fadD30 | 500D>A | 0 | 26 | 1 | 41 | 0,7860 | -0,0238 | 0,0000 |
| Rv0405 | fadD30 | 586T>A | 0 | 26 | 1 | 41 | 0,7860 | -0,0238 | 0,0000 |
| Rv0405 | pks6 | 856Q>R | 0 | 26 | 1 | 41 | 0,7860 | -0,0238 | 0,0000 |
| Rv0410c | pknG | 359V>A | 0 | 26 | 1 | 41 | 0,7860 | -0,0238 | 0,0000 |
| Rv0419 | lpqM | 295P>S | 0 | 26 | 1 | 41 | 0,7860 | -0,0238 | 0,0000 |
| Rv0421c | Rv0421c | 177E>G | 0 | 26 | 1 | 41 | 0,7860 | -0,0238 | 0,0000 |
| Rv0422c | thiD | 58D>Y | 0 | 26 | 1 | 41 | 0,7860 | -0,0238 | 0,0000 |
| Rv0425c | ctpH | 197V>A | 0 | 26 | 1 | 41 | 0,7860 | -0,0238 | 0,0000 |
| Rv0425c | ctpH | 442P>S | 0 | 26 | 1 | 41 | 0,7860 | -0,0238 | 0,0000 |
| Rv0434 | Rv0434 | 126Q>H | 0 | 26 | 1 | 41 | 0,7860 | -0,0238 | 0,0000 |
| Rv0434 | Rv0434 | 84R>G | 0 | 26 | 1 | 41 | 0,7860 | -0,0238 | 0,0000 |
| Rv0446c | Rv0446c | 131L>V | 0 | 26 | 1 | 41 | 0,7860 | -0,0238 | 0,0000 |
| Rv0446c | Rv0446c | 59L>P | 0 | 26 | 1 | 41 | 0,7860 | -0,0238 | 0,0000 |
| Rv0447c | ufaA1 | 54Y>D | 0 | 26 | 1 | 41 | 0,7860 | -0,0238 | 0,0000 |
| Rv0450c | mmpL4 | 572F>V | 0 | 26 | 1 | 41 | 0,7860 | -0,0238 | 0,0000 |
| Rv0453 | PPE11 | 292N>S | 0 | 26 | 1 | 41 | 0,7860 | -0,0238 | 0,0000 |
| Rv0457c | Rv0457c | 542L>W | 0 | 26 | 1 | 41 | 0,7860 | -0,0238 | 0,0000 |
| Rv0457c | Rv0457c | 61I>L | 0 | 26 | 1 | 41 | 0,7860 | -0,0238 | 0,0000 |
| Rv0457c | Rv0457c | 655Q>* | 0 | 26 | 1 | 41 | 0,7860 | -0,0238 | 0,0000 |
| Rv0465c | Rv0465c | 128E>K | 0 | 26 | 1 | 41 | 0,7860 | -0,0238 | 0,0000 |
| Rv0465c | Rv0465c | 231A>V | 0 | 26 | 1 | 41 | 0,7860 | -0,0238 | 0,0000 |
| Rv0465c | Rv0465c | 313Q>* | 0 | 26 | 1 | 41 | 0,7860 | -0,0238 | 0,0000 |
| Rv0471c | Rv0471c | 67W>* | 0 | 26 | 1 | 41 | 0,7860 | -0,0238 | 0,0000 |
| Rv0480c | Rv0480c | 42T>A | 0 | 26 | 1 | 41 | 0,7860 | -0,0238 | 0,0000 |
| Rv0482 | murB | 190T>I | 0 | 26 | 1 | 41 | 0,7860 | -0,0238 | 0,0000 |
| Rv0490 | senX3 | 257R>C | 0 | 26 | 1 | 41 | 0,7860 | -0,0238 | 0,0000 |
| Rv0492A | Rv0492A | 90A>T | 0 | 26 | 1 | 41 | 0,7860 | -0,0238 | 0,0000 |
| Rv0497 | Rv0497 | 12G>S | 0 | 26 | 1 | 41 | 0,7860 | -0,0238 | 0,0000 |

| Rv0500 | proC | 168P>Q | 0 | 26 | 1 | 41 | 0,7860 | -0,0238 | 0,0000 |
| --- | --- | --- | --- | --- | --- | --- | --- | --- | --- |
| Rv0511 | hemD | 321P>L | 0 | 26 | 1 | 41 | 0,7860 | -0,0238 | 0,0000 |
| Rv0519c | Rv0519c | 117M>T | 0 | 26 | 1 | 41 | 0,7860 | -0,0238 | 0,0000 |
| Rv0522 | gabP | 413W>C | 0 | 26 | 1 | 41 | 0,7860 | -0,0238 | 0,0000 |
| Rv0532 | PE_PGRS6 | 139N>H | 0 | 26 | 1 | 41 | 0,7860 | -0,0238 | 0,0000 |
| Rv0532 | PE_PGRS6 | 142N>T | 0 | 26 | 1 | 41 | 0,7860 | -0,0238 | 0,0000 |
| Rv0536 | galE3 | 110R>C | 0 | 26 | 1 | 41 | 0,7860 | -0,0238 | 0,0000 |
| Rv0545c | pitA | 404H>R | 0 | 26 | 1 | 41 | 0,7860 | -0,0238 | 0,0000 |
| Rv0563 | htpX | 39A>V | 0 | 26 | 1 | 41 | 0,7860 | -0,0238 | 0,0000 |
| Rv0566c | Rv0566c | 18D>N | 0 | 26 | 1 | 41 | 0,7860 | -0,0238 | 0,0000 |
| Rv0567 | Rv0567 | 228G>R | 0 | 26 | 1 | 41 | 0,7860 | -0,0238 | 0,0000 |
| Rv0568 | cyp135B1 | 209D>G | 0 | 26 | 1 | 41 | 0,7860 | -0,0238 | 0,0000 |
| Rv0568 | cyp135B1 | 29L>R | 0 | 26 | 1 | 41 | 0,7860 | -0,0238 | 0,0000 |
| Rv0570 | nrdZ | 25E>D | 0 | 26 | 1 | 41 | 0,7860 | -0,0238 | 0,0000 |
| Rv0570 | nrdZ | 394N>K | 0 | 26 | 1 | 41 | 0,7860 | -0,0238 | 0,0000 |
| Rv0570 | nrdZ | 618V>F | 0 | 26 | 1 | 41 | 0,7860 | -0,0238 | 0,0000 |
| Rv0570 | nrdZ | 632A>T | 0 | 26 | 1 | 41 | 0,7860 | -0,0238 | 0,0000 |
| Rv0576 | Rv0576 | 254A>V | 0 | 26 | 1 | 41 | 0,7860 | -0,0238 | 0,0000 |
| Rv0576 | Rv0576 | 338T>M | 0 | 26 | 1 | 41 | 0,7860 | -0,0238 | 0,0000 |
| Rv0577 | TB27.3 | 220K>N | 0 | 26 | 1 | 41 | 0,7860 | -0,0238 | 0,0000 |
| Rv0578c | PE_PGRS7 | 128A>V | 0 | 26 | 1 | 41 | 0,7860 | -0,0238 | 0,0000 |
| Rv0579 | Rv0579 | 144R>G | 0 | 26 | 1 | 41 | 0,7860 | -0,0238 | 0,0000 |
| Rv0579 | Rv0579 | 54L>R | 0 | 26 | 1 | 41 | 0,7860 | -0,0238 | 0,0000 |
| Rv0582 | Rv0582 | 102V>L | 0 | 26 | 1 | 41 | 0,7860 | -0,0238 | 0,0000 |
| Rv0585c | Rv0585c | 505V>F | 0 | 26 | 1 | 41 | 0,7860 | -0,0238 | 0,0000 |
| Rv0586 | Rv0586 | 48R>* | 0 | 26 | 1 | 41 | 0,7860 | -0,0238 | 0,0000 |
| Rv0587 | yrbE2A | 263T>K | 0 | 26 | 1 | 41 | 0,7860 | -0,0238 | 0,0000 |
| Rv0588 | yrbE2B | 46R>W | 0 | 26 | 1 | 41 | 0,7860 | -0,0238 | 0,0000 |
| Rv0594 | mce2F | 253T>A | 0 | 26 | 1 | 41 | 0,7860 | -0,0238 | 0,0000 |
| Rv0594 | mce2F | 410A>V | 0 | 26 | 1 | 41 | 0,7860 | -0,0238 | 0,0000 |
| Rv0600c | Rv0600c | 68V>L | 0 | 26 | 1 | 41 | 0,7860 | -0,0238 | 0,0000 |
| Rv0604 | lpqO | 203G>S | 0 | 26 | 1 | 41 | 0,7860 | -0,0238 | 0,0000 |
| Rv0610c | Rv0610c | 243H>P | 0 | 26 | 1 | 41 | 0,7860 | -0,0238 | 0,0000 |

| Rv0629c | recD | 155R>C | 0 | 26 | 1 | 41 | 0,7860 | -0,0238 | 0,0000 |
| --- | --- | --- | --- | --- | --- | --- | --- | --- | --- |
| Rv0630c | recB | 878E>D | 0 | 26 | 1 | 41 | 0,7860 | -0,0238 | 0,0000 |
| Rv0630c | recB | 946D>G | 0 | 26 | 1 | 41 | 0,7860 | -0,0238 | 0,0000 |
| Rv0632c | echA3 | 224G>R | 0 | 26 | 1 | 41 | 0,7860 | -0,0238 | 0,0000 |
| Rv0638 | secE | 45R>Q | 0 | 26 | 1 | 41 | 0,7860 | -0,0238 | 0,0000 |
| Rv0641 | rplA | 144T>I | 0 | 26 | 1 | 41 | 0,7860 | -0,0238 | 0,0000 |
| Rv0645c | mmaA1 | 232A>T | 0 | 26 | 1 | 41 | 0,7860 | -0,0238 | 0,0000 |
| Rv0646c | lipG | 161R>C | 0 | 26 | 1 | 41 | 0,7860 | -0,0238 | 0,0000 |
| Rv0648 | Rv0648 | 665P>S | 0 | 26 | 1 | 41 | 0,7860 | -0,0238 | 0,0000 |
| Rv0648 | Rv0648 | 823R>L | 0 | 26 | 1 | 41 | 0,7860 | -0,0238 | 0,0000 |
| Rv0654 | Rv0654 | 148P>A | 0 | 26 | 1 | 41 | 0,7860 | -0,0238 | 0,0000 |
| Rv0655 | mkl | 144V>A | 0 | 26 | 1 | 41 | 0,7860 | -0,0238 | 0,0000 |
| Rv0659c | Rv0659c | 29R>C | 0 | 26 | 1 | 41 | 0,7860 | -0,0238 | 0,0000 |
| Rv0662c | Rv0662c | 53D>H | 0 | 26 | 1 | 41 | 0,7860 | -0,0238 | 0,0000 |
| Rv0663 | atsD | 302E>K | 0 | 26 | 1 | 41 | 0,7860 | -0,0238 | 0,0000 |
| Rv0667 | rpoB | 432Q>P | 0 | 26 | 1 | 41 | 0,7860 | -0,0238 | 0,0000 |
| Rv0667 | rpoB | 445H>S | 0 | 26 | 1 | 41 | 0,7860 | -0,0238 | 0,0000 |
| Rv0667 | rpoB | 452L>P | 0 | 26 | 1 | 41 | 0,7860 | -0,0238 | 0,0000 |
| Rv0667 | rpoB | 835H>R | 0 | 26 | 1 | 41 | 0,7860 | -0,0238 | 0,0000 |
| Rv0669c | Rv0669c | 104Y>C | 0 | 26 | 1 | 41 | 0,7860 | -0,0238 | 0,0000 |
| Rv0669c | Rv0669c | 609Y>C | 0 | 26 | 1 | 41 | 0,7860 | -0,0238 | 0,0000 |
| Rv0670 | end | 248E>G | 0 | 26 | 1 | 41 | 0,7860 | -0,0238 | 0,0000 |
| Rv0672 | fadE8 | 13P>S | 0 | 26 | 1 | 41 | 0,7860 | -0,0238 | 0,0000 |
| Rv0676c | mmpL5 | 894S>P | 0 | 26 | 1 | 41 | 0,7860 | -0,0238 | 0,0000 |
| Rv0690c | Rv0690c | 165R>C | 0 | 26 | 1 | 41 | 0,7860 | -0,0238 | 0,0000 |
| Rv0696 | Rv0696 | 374A>V | 0 | 26 | 1 | 41 | 0,7860 | -0,0238 | 0,0000 |
| Rv0701 | rplC | 115T>A | 0 | 26 | 1 | 41 | 0,7860 | -0,0238 | 0,0000 |
| Rv0711 | atsA | 19D>H | 0 | 26 | 1 | 41 | 0,7860 | -0,0238 | 0,0000 |
| Rv0713 | Rv0713 | 314*>E | 0 | 26 | 1 | 41 | 0,7860 | -0,0238 | 0,0000 |
| Rv0725c | Rv0724A | 4G>R | 0 | 26 | 1 | 41 | 0,7860 | -0,0238 | 0,0000 |
| Rv0727c | fucA | 139P>L | 0 | 26 | 1 | 41 | 0,7860 | -0,0238 | 0,0000 |
| Rv0729 | xylB | 166P>L | 0 | 26 | 1 | 41 | 0,7860 | -0,0238 | 0,0000 |
| Rv0729 | xylB | 227D>G | 0 | 26 | 1 | 41 | 0,7860 | -0,0238 | 0,0000 |

| Rv0729 | xylB | 377A>T | 0 | 26 | 1 | 41 | 0,7860 | -0,0238 | 0,0000 |
| --- | --- | --- | --- | --- | --- | --- | --- | --- | --- |
| Rv0733 | adk | 125K>R | 0 | 26 | 1 | 41 | 0,7860 | -0,0238 | 0,0000 |
| Rv0742 | PE_PGRS8 | 130G>R | 0 | 26 | 1 | 41 | 0,7860 | -0,0238 | 0,0000 |
| Rv0751c | mmsB | 45V>F | 0 | 26 | 1 | 41 | 0,7860 | -0,0238 | 0,0000 |
| Rv0756c | Rv0756c | 179A>T | 0 | 26 | 1 | 41 | 0,7860 | -0,0238 | 0,0000 |
| Rv0758 | phoR | 143V>A | 0 | 26 | 1 | 41 | 0,7860 | -0,0238 | 0,0000 |
| Rv0758 | phoR | 279A>V | 0 | 26 | 1 | 41 | 0,7860 | -0,0238 | 0,0000 |
| Rv0758 | phoR | 81F>V | 0 | 26 | 1 | 41 | 0,7860 | -0,0238 | 0,0000 |
| Rv0767c | Rv0767c | 68Y>D | 0 | 26 | 1 | 41 | 0,7860 | -0,0238 | 0,0000 |
| Rv0770 | Rv0770 | 261G>R | 0 | 26 | 1 | 41 | 0,7860 | -0,0238 | 0,0000 |
| Rv0773c | ggtA | 344M>L | 0 | 26 | 1 | 41 | 0,7860 | -0,0238 | 0,0000 |
| Rv0778 | cyp126 | 397R>L | 0 | 26 | 1 | 41 | 0,7860 | -0,0238 | 0,0000 |
| Rv0783c | emrB | 121G>D | 0 | 26 | 1 | 41 | 0,7860 | -0,0238 | 0,0000 |
| Rv0786c | Rv0786c | 51G>D | 0 | 26 | 1 | 41 | 0,7860 | -0,0238 | 0,0000 |
| Rv0800 | pepC | 199A>V | 0 | 26 | 1 | 41 | 0,7860 | -0,0238 | 0,0000 |
| Rv0803 | purL | 273G>R | 0 | 26 | 1 | 41 | 0,7860 | -0,0238 | 0,0000 |
| Rv0804 | Rv0804 | 163I>T | 0 | 26 | 1 | 41 | 0,7860 | -0,0238 | 0,0000 |
| Rv0807 | Rv0807 | 35V>M | 0 | 26 | 1 | 41 | 0,7860 | -0,0238 | 0,0000 |
| Rv0811c | Rv0811c | 362R>Q | 0 | 26 | 1 | 41 | 0,7860 | -0,0238 | 0,0000 |
| Rv0822c | Rv0822c | 128A>D | 0 | 26 | 1 | 41 | 0,7860 | -0,0238 | 0,0000 |
| Rv0822c | Rv0822c | 36A>D | 0 | 26 | 1 | 41 | 0,7860 | -0,0238 | 0,0000 |
| Rv0825c | Rv0825c | 119P>S | 0 | 26 | 1 | 41 | 0,7860 | -0,0238 | 0,0000 |
| Rv0825c | Rv0825c | 1V>A | 0 | 26 | 1 | 41 | 0,7860 | -0,0238 | 0,0000 |
| Rv0825c | Rv0825c | 41A>T | 0 | 26 | 1 | 41 | 0,7860 | -0,0238 | 0,0000 |
| Rv0827c | Rv0827c | 98F>L | 0 | 26 | 1 | 41 | 0,7860 | -0,0238 | 0,0000 |
| Rv0830 | Rv0830 | 12A>V | 0 | 26 | 1 | 41 | 0,7860 | -0,0238 | 0,0000 |
| Rv0832 | PE_PGRS12 | 132G>W | 0 | 26 | 1 | 41 | 0,7860 | -0,0238 | 0,0000 |
| Rv0837c | Rv0837c | 168I>V | 0 | 26 | 1 | 41 | 0,7860 | -0,0238 | 0,0000 |
| Rv0838 | lpqR | 95P>S | 0 | 26 | 1 | 41 | 0,7860 | -0,0238 | 0,0000 |
| Rv0845 | Rv0845 | 83W>R | 0 | 26 | 1 | 41 | 0,7860 | -0,0238 | 0,0000 |
| Rv0849 | Rv0849 | 12R>H | 0 | 26 | 1 | 41 | 0,7860 | -0,0238 | 0,0000 |
| Rv0854 | Rv0854 | 127G>R | 0 | 26 | 1 | 41 | 0,7860 | -0,0238 | 0,0000 |
| Rv0855 | far | 1V>V | 0 | 26 | 1 | 41 | 0,7860 | -0,0238 | 0,0000 |

| Rv0858c | Rv0858c | 233M>L | 0 | 26 | 1 | 41 | 0,7860 | -0,0238 | 0,0000 |
| --- | --- | --- | --- | --- | --- | --- | --- | --- | --- |
| Rv0861c | ercc3 | 269T>P | 0 | 26 | 1 | 41 | 0,7860 | -0,0238 | 0,0000 |
| Rv0864 | moaC | 69A>T | 0 | 26 | 1 | 41 | 0,7860 | -0,0238 | 0,0000 |
| Rv0866 | moaE2 | 135D>H | 0 | 26 | 1 | 41 | 0,7860 | -0,0238 | 0,0000 |
| Rv0875c | Rv0875c | 37E>K | 0 | 26 | 1 | 41 | 0,7860 | -0,0238 | 0,0000 |
| Rv0878c | PPE13 | 137A>T | 0 | 26 | 1 | 41 | 0,7860 | -0,0238 | 0,0000 |
| Rv0878c | PPE13 | 302T>A | 0 | 26 | 1 | 41 | 0,7860 | -0,0238 | 0,0000 |
| Rv0881 | Rv0881 | 244A>T | 0 | 26 | 1 | 41 | 0,7860 | -0,0238 | 0,0000 |
| Rv0882 | Rv0882 | 40H>Y | 0 | 26 | 1 | 41 | 0,7860 | -0,0238 | 0,0000 |
| Rv0883c | Rv0883c | 186T>A | 0 | 26 | 1 | 41 | 0,7860 | -0,0238 | 0,0000 |
| Rv0889c | citA | 20T>I | 0 | 26 | 1 | 41 | 0,7860 | -0,0238 | 0,0000 |
| Rv0891c | Rv0891c | 205G>D | 0 | 26 | 1 | 41 | 0,7860 | -0,0238 | 0,0000 |
| Rv0892 | Rv0892 | 243A>V | 0 | 26 | 1 | 41 | 0,7860 | -0,0238 | 0,0000 |
| Rv0894 | Rv0894 | 377L>V | 0 | 26 | 1 | 41 | 0,7860 | -0,0238 | 0,0000 |
| Rv0895 | Rv0895 | 34A>T | 0 | 26 | 1 | 41 | 0,7860 | -0,0238 | 0,0000 |
| Rv0897c | Rv0897c | 123P>T | 0 | 26 | 1 | 41 | 0,7860 | -0,0238 | 0,0000 |
| Rv0897c | Rv0897c | 124D>G | 0 | 26 | 1 | 41 | 0,7860 | -0,0238 | 0,0000 |
| Rv0897c | Rv0897c | 234V>F | 0 | 26 | 1 | 41 | 0,7860 | -0,0238 | 0,0000 |
| Rv0902c | prrB | 361T>S | 0 | 26 | 1 | 41 | 0,7860 | -0,0238 | 0,0000 |
| Rv0902c | prrB | 369S>G | 0 | 26 | 1 | 41 | 0,7860 | -0,0238 | 0,0000 |
| Rv0903c | prrA | 157N>K | 0 | 26 | 1 | 41 | 0,7860 | -0,0238 | 0,0000 |
| Rv0903c | prrA | 216A>T | 0 | 26 | 1 | 41 | 0,7860 | -0,0238 | 0,0000 |
| Rv0905 | echA6 | 144H>Q | 0 | 26 | 1 | 41 | 0,7860 | -0,0238 | 0,0000 |
| Rv0908 | ctpE | 101A>E | 0 | 26 | 1 | 41 | 0,7860 | -0,0238 | 0,0000 |
| Rv0913c | Rv0913c | 338R>Q | 0 | 26 | 1 | 41 | 0,7860 | -0,0238 | 0,0000 |
| Rv0915c | PPE14 | 251G>R | 0 | 26 | 1 | 41 | 0,7860 | -0,0238 | 0,0000 |
| Rv0915c | PPE14 | 313G>R | 0 | 26 | 1 | 41 | 0,7860 | -0,0238 | 0,0000 |
| Rv0915c | PPE14 | 88T>I | 0 | 26 | 1 | 41 | 0,7860 | -0,0238 | 0,0000 |
| Rv0918 | Rv0918 | 60T>A | 0 | 26 | 1 | 41 | 0,7860 | -0,0238 | 0,0000 |
| Rv0923c | Rv0923c | 122D>H | 0 | 26 | 1 | 41 | 0,7860 | -0,0238 | 0,0000 |
| Rv0923c | Rv0923c | 292I>T | 0 | 26 | 1 | 41 | 0,7860 | -0,0238 | 0,0000 |
| Rv0926c | Rv0926c | 111P>L | 0 | 26 | 1 | 41 | 0,7860 | -0,0238 | 0,0000 |
| Rv0931c | pknD | 137R>H | 0 | 26 | 1 | 41 | 0,7860 | -0,0238 | 0,0000 |

| Rv0931c | pknD | 399Y>* | 0 | 26 | 1 | 41 | 0,7860 | -0,0238 | 0,0000 |
| --- | --- | --- | --- | --- | --- | --- | --- | --- | --- |
| Rv0938 | Rv0938 | 315T>M | 0 | 26 | 1 | 41 | 0,7860 | -0,0238 | 0,0000 |
| Rv0944 | Rv0944 | 69L>V | 0 | 26 | 1 | 41 | 0,7860 | -0,0238 | 0,0000 |
| Rv0946c | pgi | 542V>L | 0 | 26 | 1 | 41 | 0,7860 | -0,0238 | 0,0000 |
| Rv0950c | Rv0950c | 264T>A | 0 | 26 | 1 | 41 | 0,7860 | -0,0238 | 0,0000 |
| Rv0953c | Rv0953c | 140R>C | 0 | 26 | 1 | 41 | 0,7860 | -0,0238 | 0,0000 |
| Rv0958 | Rv0958 | 212T>M | 0 | 26 | 1 | 41 | 0,7860 | -0,0238 | 0,0000 |
| Rv0958 | Rv0958 | 396T>R | 0 | 26 | 1 | 41 | 0,7860 | -0,0238 | 0,0000 |
| Rv0969 | ctpV | 661Q>* | 0 | 26 | 1 | 41 | 0,7860 | -0,0238 | 0,0000 |
| Rv0971c | echA7 | 117G>R | 0 | 26 | 1 | 41 | 0,7860 | -0,0238 | 0,0000 |
| Rv0972c | fadE12 | 59P>T | 0 | 26 | 1 | 41 | 0,7860 | -0,0238 | 0,0000 |
| Rv0973c | accA2 | 9A>V | 0 | 26 | 1 | 41 | 0,7860 | -0,0238 | 0,0000 |
| Rv0978c | PE_PGRS17 | 245N>D | 0 | 26 | 1 | 41 | 0,7860 | -0,0238 | 0,0000 |
| Rv0978c | PE_PGRS17 | 247A>T | 0 | 26 | 1 | 41 | 0,7860 | -0,0238 | 0,0000 |
| Rv0989c | grcC2 | 76A>P | 0 | 26 | 1 | 41 | 0,7860 | -0,0238 | 0,0000 |
| Rv0990c | Rv0990c | 1V>V | 0 | 26 | 1 | 41 | 0,7860 | -0,0238 | 0,0000 |
| Rv1001 | arcA | 228V>I | 0 | 26 | 1 | 41 | 0,7860 | -0,0238 | 0,0000 |
| Rv1003 | Rv1003 | 45A>V | 0 | 26 | 1 | 41 | 0,7860 | -0,0238 | 0,0000 |
| Rv1004c | Rv1004c | 167P>S | 0 | 26 | 1 | 41 | 0,7860 | -0,0238 | 0,0000 |
| Rv1004c | Rv1004c | 347P>L | 0 | 26 | 1 | 41 | 0,7860 | -0,0238 | 0,0000 |
| Rv1005c | pabB | 169R>Q | 0 | 26 | 1 | 41 | 0,7860 | -0,0238 | 0,0000 |
| Rv1009 | rpfB | 19Y>D | 0 | 26 | 1 | 41 | 0,7860 | -0,0238 | 0,0000 |
| Rv1011 | ispE | 261C>W | 0 | 26 | 1 | 41 | 0,7860 | -0,0238 | 0,0000 |
| Rv1013 | pks16 | 21T>A | 0 | 26 | 1 | 41 | 0,7860 | -0,0238 | 0,0000 |
| Rv1018c | glmU | 110V>A | 0 | 26 | 1 | 41 | 0,7860 | -0,0238 | 0,0000 |
| Rv1020 | mfd | 826T>A | 0 | 26 | 1 | 41 | 0,7860 | -0,0238 | 0,0000 |
| Rv1020 | mfd | 865N>D | 0 | 26 | 1 | 41 | 0,7860 | -0,0238 | 0,0000 |
| Rv1025 | Rv1025 | 61T>A | 0 | 26 | 1 | 41 | 0,7860 | -0,0238 | 0,0000 |
| Rv1027c | kdpE | 166R>P | 0 | 26 | 1 | 41 | 0,7860 | -0,0238 | 0,0000 |
| Rv1028c | kdpD | 365V>F | 0 | 26 | 1 | 41 | 0,7860 | -0,0238 | 0,0000 |
| Rv1028c | kdpD | 750A>V | 0 | 26 | 1 | 41 | 0,7860 | -0,0238 | 0,0000 |
| Rv1030 | kdpB | 67T>I | 0 | 26 | 1 | 41 | 0,7860 | -0,0238 | 0,0000 |
| Rv1035c | Rv1035c | 206D>N | 0 | 26 | 1 | 41 | 0,7860 | -0,0238 | 0,0000 |

| Rv1039c | PPE15 | 177G>S | 0 | 26 | 1 | 41 | 0,7860 | -0,0238 | 0,0000 |
| --- | --- | --- | --- | --- | --- | --- | --- | --- | --- |
| Rv1048c | Rv1048c | 204L>R | 0 | 26 | 1 | 41 | 0,7860 | -0,0238 | 0,0000 |
| Rv1050 | Rv1050 | 52I>V | 0 | 26 | 1 | 41 | 0,7860 | -0,0238 | 0,0000 |
| Rv1050 | Rv1050 | 63P>L | 0 | 26 | 1 | 41 | 0,7860 | -0,0238 | 0,0000 |
| Rv1056 | Rv1056 | 90A>V | 0 | 26 | 1 | 41 | 0,7860 | -0,0238 | 0,0000 |
| Rv1057 | Rv1057 | 258A>V | 0 | 26 | 1 | 41 | 0,7860 | -0,0238 | 0,0000 |
| Rv1061 | Rv1061 | 43F>S | 0 | 26 | 1 | 41 | 0,7860 | -0,0238 | 0,0000 |
| Rv1063c | Rv1063c | 297S>F | 0 | 26 | 1 | 41 | 0,7860 | -0,0238 | 0,0000 |
| Rv1065 | Rv1065 | 93V>L | 0 | 26 | 1 | 41 | 0,7860 | -0,0238 | 0,0000 |
| Rv1067c | PE_PGRS19 | 507G>S | 0 | 26 | 1 | 41 | 0,7860 | -0,0238 | 0,0000 |
| Rv1075c | Rv1075c | 205V>M | 0 | 26 | 1 | 41 | 0,7860 | -0,0238 | 0,0000 |
| Rv1075c | Rv1075c | 57G>S | 0 | 26 | 1 | 41 | 0,7860 | -0,0238 | 0,0000 |
| Rv1076 | lipU | 258P>L | 0 | 26 | 1 | 41 | 0,7860 | -0,0238 | 0,0000 |
| Rv1078 | pra | 41P>S | 0 | 26 | 1 | 41 | 0,7860 | -0,0238 | 0,0000 |
| Rv1079 | metB | 70S>L | 0 | 26 | 1 | 41 | 0,7860 | -0,0238 | 0,0000 |
| Rv1084 | Rv1084 | 330S>A | 0 | 26 | 1 | 41 | 0,7860 | -0,0238 | 0,0000 |
| Rv1087 | PE_PGRS21 | 191F>C | 0 | 26 | 1 | 41 | 0,7860 | -0,0238 | 0,0000 |
| Rv1087 | PE_PGRS21 | 259V>A | 0 | 26 | 1 | 41 | 0,7860 | -0,0238 | 0,0000 |
| Rv1087 | PE_PGRS21 | 496G>S | 0 | 26 | 1 | 41 | 0,7860 | -0,0238 | 0,0000 |
| Rv1088 | PE9 | 84C>G | 0 | 26 | 1 | 41 | 0,7860 | -0,0238 | 0,0000 |
| Rv1094 | desA2 | 241D>Y | 0 | 26 | 1 | 41 | 0,7860 | -0,0238 | 0,0000 |
| Rv1096 | Rv1096 | 21V>I | 0 | 26 | 1 | 41 | 0,7860 | -0,0238 | 0,0000 |
| Rv1121 | zwf1 | 57V>L | 0 | 26 | 1 | 41 | 0,7860 | -0,0238 | 0,0000 |
| Rv1122 | gnd2 | 24H>R | 0 | 26 | 1 | 41 | 0,7860 | -0,0238 | 0,0000 |
| Rv1129c | Rv1129c | 190L>S | 0 | 26 | 1 | 41 | 0,7860 | -0,0238 | 0,0000 |
| Rv1130 | Rv1130 | 327E>D | 0 | 26 | 1 | 41 | 0,7860 | -0,0238 | 0,0000 |
| Rv1131 | gltA1 | 260A>V | 0 | 26 | 1 | 41 | 0,7860 | -0,0238 | 0,0000 |
| Rv1132 | Rv1132 | 251H>R | 0 | 26 | 1 | 41 | 0,7860 | -0,0238 | 0,0000 |
| Rv1133c | metE | 99D>G | 0 | 26 | 1 | 41 | 0,7860 | -0,0238 | 0,0000 |
| Rv1135c | PPE16 | 503P>L | 0 | 26 | 1 | 41 | 0,7860 | -0,0238 | 0,0000 |
| Rv1137c | Rv1137c | 66G>V | 0 | 26 | 1 | 41 | 0,7860 | -0,0238 | 0,0000 |
| Rv1138c | Rv1138c | 182H>P | 0 | 26 | 1 | 41 | 0,7860 | -0,0238 | 0,0000 |
| Rv1140 | Rv1140 | 91A>S | 0 | 26 | 1 | 41 | 0,7860 | -0,0238 | 0,0000 |

| Rv1161 | narG | 541G>S | 0 | 26 | 1 | 41 | 0,7860 | -0,0238 | 0,0000 |
| --- | --- | --- | --- | --- | --- | --- | --- | --- | --- |
| Rv1161 | narG | 741P>L | 0 | 26 | 1 | 41 | 0,7860 | -0,0238 | 0,0000 |
| Rv1165 | typA | 323D>G | 0 | 26 | 1 | 41 | 0,7860 | -0,0238 | 0,0000 |
| Rv1173 | fbiC | 45A>T | 0 | 26 | 1 | 41 | 0,7860 | -0,0238 | 0,0000 |
| Rv1175c | fadH | 254W>* | 0 | 26 | 1 | 41 | 0,7860 | -0,0238 | 0,0000 |
| Rv1176c | Rv1176c | 51N>S | 0 | 26 | 1 | 41 | 0,7860 | -0,0238 | 0,0000 |
| Rv1179c | Rv1179c | 408T>A | 0 | 26 | 1 | 41 | 0,7860 | -0,0238 | 0,0000 |
| Rv1179c | Rv1179c | 47G>S | 0 | 26 | 1 | 41 | 0,7860 | -0,0238 | 0,0000 |
| Rv1180 | pks3 | 392F>I | 0 | 26 | 1 | 41 | 0,7860 | -0,0238 | 0,0000 |
| Rv1181 | pks4 | 1040L>V | 0 | 26 | 1 | 41 | 0,7860 | -0,0238 | 0,0000 |
| Rv1181 | pks4 | 1169H>R | 0 | 26 | 1 | 41 | 0,7860 | -0,0238 | 0,0000 |
| Rv1182 | papA3 | 243S>T | 0 | 26 | 1 | 41 | 0,7860 | -0,0238 | 0,0000 |
| Rv1183 | mmpL10 | 996V>A | 0 | 26 | 1 | 41 | 0,7860 | -0,0238 | 0,0000 |
| Rv1185c | fadD21 | 391C>Y | 0 | 26 | 1 | 41 | 0,7860 | -0,0238 | 0,0000 |
| Rv1186c | Rv1186c | 211V>L | 0 | 26 | 1 | 41 | 0,7860 | -0,0238 | 0,0000 |
| Rv1193 | fadD36 | 157D>V | 0 | 26 | 1 | 41 | 0,7860 | -0,0238 | 0,0000 |
| Rv1193 | fadD36 | 211A>V | 0 | 26 | 1 | 41 | 0,7860 | -0,0238 | 0,0000 |
| Rv1193 | fadD36 | 472E>A | 0 | 26 | 1 | 41 | 0,7860 | -0,0238 | 0,0000 |
| Rv1194c | Rv1194c | 138R>C | 0 | 26 | 1 | 41 | 0,7860 | -0,0238 | 0,0000 |
| Rv1194c | Rv1194c | 329T>A | 0 | 26 | 1 | 41 | 0,7860 | -0,0238 | 0,0000 |
| Rv1194c | Rv1194c | 403G>D | 0 | 26 | 1 | 41 | 0,7860 | -0,0238 | 0,0000 |
| Rv1198 | esxL | 37T>A | 0 | 26 | 1 | 41 | 0,7860 | -0,0238 | 0,0000 |
| Rv1200 | Rv1200 | 389A>V | 0 | 26 | 1 | 41 | 0,7860 | -0,0238 | 0,0000 |
| Rv1206 | fadD6 | 545T>A | 0 | 26 | 1 | 41 | 0,7860 | -0,0238 | 0,0000 |
| Rv1212c | Rv1212c | 201M>V | 0 | 26 | 1 | 41 | 0,7860 | -0,0238 | 0,0000 |
| Rv1213 | glgC | 52R>G | 0 | 26 | 1 | 41 | 0,7860 | -0,0238 | 0,0000 |
| Rv1218c | Rv1218c | 91V>G | 0 | 26 | 1 | 41 | 0,7860 | -0,0238 | 0,0000 |
| Rv1219c | Rv1219c | 160M>I | 0 | 26 | 1 | 41 | 0,7860 | -0,0238 | 0,0000 |
| Rv1230c | Rv1230c | 372P>Q | 0 | 26 | 1 | 41 | 0,7860 | -0,0238 | 0,0000 |
| Rv1235 | lpqY | 466L>P | 0 | 26 | 1 | 41 | 0,7860 | -0,0238 | 0,0000 |
| Rv1240 | mdh | 47A>V | 0 | 26 | 1 | 41 | 0,7860 | -0,0238 | 0,0000 |
| Rv1244 | lpqZ | 106Y>F | 0 | 26 | 1 | 41 | 0,7860 | -0,0238 | 0,0000 |
| Rv1246c | Rv1246c | 63D>G | 0 | 26 | 1 | 41 | 0,7860 | -0,0238 | 0,0000 |

| Rv1251c | Rv1251c | 3V>A | 0 | 26 | 1 | 41 | 0,7860 | -0,0238 | 0,0000 |
| --- | --- | --- | --- | --- | --- | --- | --- | --- | --- |
| Rv1251c | Rv1251c | 59R>C | 0 | 26 | 1 | 41 | 0,7860 | -0,0238 | 0,0000 |
| Rv1253 | deaD | 332E>Q | 0 | 26 | 1 | 41 | 0,7860 | -0,0238 | 0,0000 |
| Rv1253 | deaD | 371Q>R | 0 | 26 | 1 | 41 | 0,7860 | -0,0238 | 0,0000 |
| Rv1256c | cyp130 | 105S>L | 0 | 26 | 1 | 41 | 0,7860 | -0,0238 | 0,0000 |
| Rv1264 | Rv1264 | 256P>S | 0 | 26 | 1 | 41 | 0,7860 | -0,0238 | 0,0000 |
| Rv1266c | pknH | 236S>T | 0 | 26 | 1 | 41 | 0,7860 | -0,0238 | 0,0000 |
| Rv1267c | embR | 217L>V | 0 | 26 | 1 | 41 | 0,7860 | -0,0238 | 0,0000 |
| Rv1271c | Rv1271c | 62A>T | 0 | 26 | 1 | 41 | 0,7860 | -0,0238 | 0,0000 |
| Rv1272c | Rv1272c | 100A>D | 0 | 26 | 1 | 41 | 0,7860 | -0,0238 | 0,0000 |
| Rv1273c | Rv1273c | 226A>T | 0 | 26 | 1 | 41 | 0,7860 | -0,0238 | 0,0000 |
| Rv1277 | Rv1277 | 16R>P | 0 | 26 | 1 | 41 | 0,7860 | -0,0238 | 0,0000 |
| Rv1280c | oppA | 395D>A | 0 | 26 | 1 | 41 | 0,7860 | -0,0238 | 0,0000 |
| Rv1281c | oppD | 224R>Q | 0 | 26 | 1 | 41 | 0,7860 | -0,0238 | 0,0000 |
| Rv1283c | oppB | 59D>G | 0 | 26 | 1 | 41 | 0,7860 | -0,0238 | 0,0000 |
| Rv1290c | Rv1290c | 173T>A | 0 | 26 | 1 | 41 | 0,7860 | -0,0238 | 0,0000 |
| Rv1290c | Rv1290c | 329R>C | 0 | 26 | 1 | 41 | 0,7860 | -0,0238 | 0,0000 |
| Rv1295 | thrC | 204Y>S | 0 | 26 | 1 | 41 | 0,7860 | -0,0238 | 0,0000 |
| Rv1295 | thrC | 8T>P | 0 | 26 | 1 | 41 | 0,7860 | -0,0238 | 0,0000 |
| Rv1296 | thrB | 315P>S | 0 | 26 | 1 | 41 | 0,7860 | -0,0238 | 0,0000 |
| Rv1297 | rho | 163G>D | 0 | 26 | 1 | 41 | 0,7860 | -0,0238 | 0,0000 |
| Rv1297 | rho | 184G>A | 0 | 26 | 1 | 41 | 0,7860 | -0,0238 | 0,0000 |
| Rv1303 | Rv1303 | 79R>Q | 0 | 26 | 1 | 41 | 0,7860 | -0,0238 | 0,0000 |
| Rv1304 | atpB | 250H>P | 0 | 26 | 1 | 41 | 0,7860 | -0,0238 | 0,0000 |
| Rv1315 | murA | 257P>T | 0 | 26 | 1 | 41 | 0,7860 | -0,0238 | 0,0000 |
| Rv1317c | alkA | 143V>M | 0 | 26 | 1 | 41 | 0,7860 | -0,0238 | 0,0000 |
| Rv1317c | alkA | 226G>S | 0 | 26 | 1 | 41 | 0,7860 | -0,0238 | 0,0000 |
| Rv1318c | Rv1318c | 323G>E | 0 | 26 | 1 | 41 | 0,7860 | -0,0238 | 0,0000 |
| Rv1320c | Rv1320c | 342V>A | 0 | 26 | 1 | 41 | 0,7860 | -0,0238 | 0,0000 |
| Rv1321 | Rv1321 | 100G>W | 0 | 26 | 1 | 41 | 0,7860 | -0,0238 | 0,0000 |
| Rv1323 | fadA4 | 339G>S | 0 | 26 | 1 | 41 | 0,7860 | -0,0238 | 0,0000 |
| Rv1328 | glgP | 227L>S | 0 | 26 | 1 | 41 | 0,7860 | -0,0238 | 0,0000 |
| Rv1329c | dinG | 272A>S | 0 | 26 | 1 | 41 | 0,7860 | -0,0238 | 0,0000 |

| Rv1329c | dinG | 515V>A | 0 | 26 | 1 | 41 | 0,7860 | -0,0238 | 0,0000 |
| --- | --- | --- | --- | --- | --- | --- | --- | --- | --- |
| Rv1333 | Rv1333 | 181V>I | 0 | 26 | 1 | 41 | 0,7860 | -0,0238 | 0,0000 |
| Rv1333 | Rv1333 | 213Q>P | 0 | 26 | 1 | 41 | 0,7860 | -0,0238 | 0,0000 |
| Rv1333 | Rv1333 | 345*>G | 0 | 26 | 1 | 41 | 0,7860 | -0,0238 | 0,0000 |
| Rv1333 | Rv1333 | 44A>E | 0 | 26 | 1 | 41 | 0,7860 | -0,0238 | 0,0000 |
| Rv1347c | Rv1347c | 10A>D | 0 | 26 | 1 | 41 | 0,7860 | -0,0238 | 0,0000 |
| Rv1350 | fabG | 155Y>D | 0 | 26 | 1 | 41 | 0,7860 | -0,0238 | 0,0000 |
| Rv1353c | Rv1353c | 208P>S | 0 | 26 | 1 | 41 | 0,7860 | -0,0238 | 0,0000 |
| Rv1357c | Rv1357c | 187R>H | 0 | 26 | 1 | 41 | 0,7860 | -0,0238 | 0,0000 |
| Rv1358 | Rv1358 | 258G>D | 0 | 26 | 1 | 41 | 0,7860 | -0,0238 | 0,0000 |
| Rv1358 | Rv1358 | 754P>S | 0 | 26 | 1 | 41 | 0,7860 | -0,0238 | 0,0000 |
| Rv1366 | Rv1366 | 143A>S | 0 | 26 | 1 | 41 | 0,7860 | -0,0238 | 0,0000 |
| Rv1368 | lprF | 212P>Q | 0 | 26 | 1 | 41 | 0,7860 | -0,0238 | 0,0000 |
| Rv1371 | Rv1371 | 114D>E | 0 | 26 | 1 | 41 | 0,7860 | -0,0238 | 0,0000 |
| Rv1372 | Rv1372 | 173M>I | 0 | 26 | 1 | 41 | 0,7860 | -0,0238 | 0,0000 |
| Rv1373 | Rv1373 | 215F>S | 0 | 26 | 1 | 41 | 0,7860 | -0,0238 | 0,0000 |
| Rv1378c | Rv1378c | 208A>G | 0 | 26 | 1 | 41 | 0,7860 | -0,0238 | 0,0000 |
| Rv1381 | pyrC | 38T>M | 0 | 26 | 1 | 41 | 0,7860 | -0,0238 | 0,0000 |
| Rv1382 | Rv1382 | 150R>W | 0 | 26 | 1 | 41 | 0,7860 | -0,0238 | 0,0000 |
| Rv1387 | PPE20 | 234L>P | 0 | 26 | 1 | 41 | 0,7860 | -0,0238 | 0,0000 |
| Rv1390 | rpoZ | 107T>A | 0 | 26 | 1 | 41 | 0,7860 | -0,0238 | 0,0000 |
| Rv1391 | dfp | 418S>G | 0 | 26 | 1 | 41 | 0,7860 | -0,0238 | 0,0000 |
| Rv1393c | Rv1393c | 63F>V | 0 | 26 | 1 | 41 | 0,7860 | -0,0238 | 0,0000 |
| Rv1393c | Rv1393c | 99R>W | 0 | 26 | 1 | 41 | 0,7860 | -0,0238 | 0,0000 |
| Rv1399c | lipH | 304E>Q | 0 | 26 | 1 | 41 | 0,7860 | -0,0238 | 0,0000 |
| Rv1403c | Rv1403c | 70A>V | 0 | 26 | 1 | 41 | 0,7860 | -0,0238 | 0,0000 |
| Rv1407 | fmu | 57E>K | 0 | 26 | 1 | 41 | 0,7860 | -0,0238 | 0,0000 |
| Rv1410c | Rv1410c | 268F>C | 0 | 26 | 1 | 41 | 0,7860 | -0,0238 | 0,0000 |
| Rv1419 | Rv1419 | 49D>G | 0 | 26 | 1 | 41 | 0,7860 | -0,0238 | 0,0000 |
| Rv1420 | uvrC | 488P>S | 0 | 26 | 1 | 41 | 0,7860 | -0,0238 | 0,0000 |
| Rv1423 | whiA | 84Y>N | 0 | 26 | 1 | 41 | 0,7860 | -0,0238 | 0,0000 |
| Rv1425 | Rv1425 | 393L>R | 0 | 26 | 1 | 41 | 0,7860 | -0,0238 | 0,0000 |
| Rv1430 | PE16 | 9E>K | 0 | 26 | 1 | 41 | 0,7860 | -0,0238 | 0,0000 |

| Rv1433 | Rv1433 | 26H>D | 0 | 26 | 1 | 41 | 0,7860 | -0,0238 | 0,0000 |
| --- | --- | --- | --- | --- | --- | --- | --- | --- | --- |
| Rv1441c | PE_PGRS26 | 86S>* | 0 | 26 | 1 | 41 | 0,7860 | -0,0238 | 0,0000 |
| Rv1449c | tkt | 540R>H | 0 | 26 | 1 | 41 | 0,7860 | -0,0238 | 0,0000 |
| Rv1452c | PE_PGRS28 | 429A>D | 0 | 26 | 1 | 41 | 0,7860 | -0,0238 | 0,0000 |
| Rv1452c | PE_PGRS28 | 435D>N | 0 | 26 | 1 | 41 | 0,7860 | -0,0238 | 0,0000 |
| Rv1452c | PE_PGRS28 | 471G>V | 0 | 26 | 1 | 41 | 0,7860 | -0,0238 | 0,0000 |
| Rv1460 | Rv1460 | 132R>W | 0 | 26 | 1 | 41 | 0,7860 | -0,0238 | 0,0000 |
| Rv1463 | Rv1463 | 107F>L | 0 | 26 | 1 | 41 | 0,7860 | -0,0238 | 0,0000 |
| Rv1469 | ctpD | 645T>S | 0 | 26 | 1 | 41 | 0,7860 | -0,0238 | 0,0000 |
| Rv1473 | Rv1473 | 243G>D | 0 | 26 | 1 | 41 | 0,7860 | -0,0238 | 0,0000 |
| Rv1478 | Rv1478 | 220K>E | 0 | 26 | 1 | 41 | 0,7860 | -0,0238 | 0,0000 |
| Rv1481 | Rv1481 | 60R>W | 0 | 26 | 1 | 41 | 0,7860 | -0,0238 | 0,0000 |
| Rv1484 | inhA | 21I>V | 0 | 26 | 1 | 41 | 0,7860 | -0,0238 | 0,0000 |
| Rv1487 | Rv1487 | 11A>P | 0 | 26 | 1 | 41 | 0,7860 | -0,0238 | 0,0000 |
| Rv1489A | Rv1489A | 41A>V | 0 | 26 | 1 | 41 | 0,7860 | -0,0238 | 0,0000 |
| Rv1489A | Rv1489A | 56S>R | 0 | 26 | 1 | 41 | 0,7860 | -0,0238 | 0,0000 |
| Rv1492 | mutA | 408Q>H | 0 | 26 | 1 | 41 | 0,7860 | -0,0238 | 0,0000 |
| Rv1498A | Rv1498A | 41E>D | 0 | 26 | 1 | 41 | 0,7860 | -0,0238 | 0,0000 |
| Rv1500 | Rv1500 | 91I>T | 0 | 26 | 1 | 41 | 0,7860 | -0,0238 | 0,0000 |
| Rv1501 | Rv1501 | 34A>E | 0 | 26 | 1 | 41 | 0,7860 | -0,0238 | 0,0000 |
| Rv1503c | Rv1503c | 39S>R | 0 | 26 | 1 | 41 | 0,7860 | -0,0238 | 0,0000 |
| Rv1511 | gmdA | 251R>C | 0 | 26 | 1 | 41 | 0,7860 | -0,0238 | 0,0000 |
| Rv1511 | gmdA | 92R>W | 0 | 26 | 1 | 41 | 0,7860 | -0,0238 | 0,0000 |
| Rv1513 | Rv1513 | 148A>S | 0 | 26 | 1 | 41 | 0,7860 | -0,0238 | 0,0000 |
| Rv1522c | mmpL12 | 496G>D | 0 | 26 | 1 | 41 | 0,7860 | -0,0238 | 0,0000 |
| Rv1523 | Rv1523 | 204H>R | 0 | 26 | 1 | 41 | 0,7860 | -0,0238 | 0,0000 |
| Rv1534 | Rv1534 | 211R>P | 0 | 26 | 1 | 41 | 0,7860 | -0,0238 | 0,0000 |
| Rv1536 | ileS | 666D>E | 0 | 26 | 1 | 41 | 0,7860 | -0,0238 | 0,0000 |
| Rv1545 | Rv1545 | 9G>C | 0 | 26 | 1 | 41 | 0,7860 | -0,0238 | 0,0000 |
| Rv1548c | PPE21 | 591A>T | 0 | 26 | 1 | 41 | 0,7860 | -0,0238 | 0,0000 |
| Rv1550 | fadD11.1 | 106P>A | 0 | 26 | 1 | 41 | 0,7860 | -0,0238 | 0,0000 |
| Rv1552 | frdA | 425A>G | 0 | 26 | 1 | 41 | 0,7860 | -0,0238 | 0,0000 |
| Rv1554 | frdC | 53Y>* | 0 | 26 | 1 | 41 | 0,7860 | -0,0238 | 0,0000 |

| Rv1554 | frdC | 89R>P | 0 | 26 | 1 | 41 | 0,7860 | -0,0238 | 0,0000 |
| --- | --- | --- | --- | --- | --- | --- | --- | --- | --- |
| Rv1557 | mmpL6 | 131A>V | 0 | 26 | 1 | 41 | 0,7860 | -0,0238 | 0,0000 |
| Rv1557 | mmpL6 | 156Q>* | 0 | 26 | 1 | 41 | 0,7860 | -0,0238 | 0,0000 |
| Rv1558 | Rv1558 | 62D>N | 0 | 26 | 1 | 41 | 0,7860 | -0,0238 | 0,0000 |
| Rv1559 | ilvA | 361R>W | 0 | 26 | 1 | 41 | 0,7860 | -0,0238 | 0,0000 |
| Rv1559 | ilvA | 386T>A | 0 | 26 | 1 | 41 | 0,7860 | -0,0238 | 0,0000 |
| Rv1560 | Rv1560 | 61G>C | 0 | 26 | 1 | 41 | 0,7860 | -0,0238 | 0,0000 |
| Rv1562c | treZ | 501N>H | 0 | 26 | 1 | 41 | 0,7860 | -0,0238 | 0,0000 |
| Rv1563c | treY | 473W>* | 0 | 26 | 1 | 41 | 0,7860 | -0,0238 | 0,0000 |
| Rv1565c | Rv1565c | 228A>T | 0 | 26 | 1 | 41 | 0,7860 | -0,0238 | 0,0000 |
| Rv1565c | Rv1565c | 293G>E | 0 | 26 | 1 | 41 | 0,7860 | -0,0238 | 0,0000 |
| Rv1566c | Rv1566c | 224F>L | 0 | 26 | 1 | 41 | 0,7860 | -0,0238 | 0,0000 |
| Rv1568 | bioA | 436L>R | 0 | 26 | 1 | 41 | 0,7860 | -0,0238 | 0,0000 |
| Rv1575 | Rv1575 | 106Q>E | 0 | 26 | 1 | 41 | 0,7860 | -0,0238 | 0,0000 |
| Rv1575 | Rv1575 | 44L>R | 0 | 26 | 1 | 41 | 0,7860 | -0,0238 | 0,0000 |
| Rv1587c | Rv1587c | 184G>D | 0 | 26 | 1 | 41 | 0,7860 | -0,0238 | 0,0000 |
| Rv1587c | Rv1586c | 6T>T | 0 | 26 | 1 | 41 | 0,7860 | -0,0238 | 0,0000 |
| Rv1597 | Rv1597 | 132V>I | 0 | 26 | 1 | 41 | 0,7860 | -0,0238 | 0,0000 |
| Rv1598c | Rv1598c | 3A>V | 0 | 26 | 1 | 41 | 0,7860 | -0,0238 | 0,0000 |
| Rv1603 | hisA | 216T>A | 0 | 26 | 1 | 41 | 0,7860 | -0,0238 | 0,0000 |
| Rv1604 | impA | 241P>A | 0 | 26 | 1 | 41 | 0,7860 | -0,0238 | 0,0000 |
| Rv1606 | hisI | 102H>Q | 0 | 26 | 1 | 41 | 0,7860 | -0,0238 | 0,0000 |
| Rv1610 | Rv1610 | 226R>W | 0 | 26 | 1 | 41 | 0,7860 | -0,0238 | 0,0000 |
| Rv1610 | Rv1610 | 67L>R | 0 | 26 | 1 | 41 | 0,7860 | -0,0238 | 0,0000 |
| Rv1617 | pykA | 22R>S | 0 | 26 | 1 | 41 | 0,7860 | -0,0238 | 0,0000 |
| Rv1621c | cydD | 47L>V | 0 | 26 | 1 | 41 | 0,7860 | -0,0238 | 0,0000 |
| Rv1626 | Rv1626 | 117S>R | 0 | 26 | 1 | 41 | 0,7860 | -0,0238 | 0,0000 |
| Rv1627c | Rv1627c | 123F>L | 0 | 26 | 1 | 41 | 0,7860 | -0,0238 | 0,0000 |
| Rv1629 | polA | 245A>V | 0 | 26 | 1 | 41 | 0,7860 | -0,0238 | 0,0000 |
| Rv1635c | Rv1635c | 328G>R | 0 | 26 | 1 | 41 | 0,7860 | -0,0238 | 0,0000 |
| Rv1637c | Rv1637c | 130A>T | 0 | 26 | 1 | 41 | 0,7860 | -0,0238 | 0,0000 |
| Rv1638 | uvrA | 299V>M | 0 | 26 | 1 | 41 | 0,7860 | -0,0238 | 0,0000 |
| Rv1639c | Rv1639c | 92W>* | 0 | 26 | 1 | 41 | 0,7860 | -0,0238 | 0,0000 |

| Rv1648 | Rv1648 | 176T>N | 0 | 26 | 1 | 41 | 0,7860 | -0,0238 | 0,0000 |
| --- | --- | --- | --- | --- | --- | --- | --- | --- | --- |
| Rv1651c | PE_PGRS30 | 166A>N | 0 | 26 | 1 | 41 | 0,7860 | -0,0238 | 0,0000 |
| Rv1651c | PE_PGRS30 | 821A>T | 0 | 26 | 1 | 41 | 0,7860 | -0,0238 | 0,0000 |
| Rv1661 | pks7 | 1017V>G | 0 | 26 | 1 | 41 | 0,7860 | -0,0238 | 0,0000 |
| Rv1661 | pks7 | 1156H>Y | 0 | 26 | 1 | 41 | 0,7860 | -0,0238 | 0,0000 |
| Rv1661 | pks7 | 925G>D | 0 | 26 | 1 | 41 | 0,7860 | -0,0238 | 0,0000 |
| Rv1663 | pks17 | 271G>V | 0 | 26 | 1 | 41 | 0,7860 | -0,0238 | 0,0000 |
| Rv1667c | Rv1667c | 101V>F | 0 | 26 | 1 | 41 | 0,7860 | -0,0238 | 0,0000 |
| Rv1669 | Rv1669 | 44L>F | 0 | 26 | 1 | 41 | 0,7860 | -0,0238 | 0,0000 |
| Rv1671 | Rv1671 | 82L>R | 0 | 26 | 1 | 41 | 0,7860 | -0,0238 | 0,0000 |
| Rv1672c | Rv1672c | 429D>E | 0 | 26 | 1 | 41 | 0,7860 | -0,0238 | 0,0000 |
| Rv1677 | dsbF | 4S>Y | 0 | 26 | 1 | 41 | 0,7860 | -0,0238 | 0,0000 |
| Rv1679 | fadE16 | 269T>M | 0 | 26 | 1 | 41 | 0,7860 | -0,0238 | 0,0000 |
| Rv1683 | Rv1683 | 424T>K | 0 | 26 | 1 | 41 | 0,7860 | -0,0238 | 0,0000 |
| Rv1683 | Rv1683 | 666L>P | 0 | 26 | 1 | 41 | 0,7860 | -0,0238 | 0,0000 |
| Rv1692 | Rv1692 | 83G>S | 0 | 26 | 1 | 41 | 0,7860 | -0,0238 | 0,0000 |
| Rv1694 | tlyA | 62W>L | 0 | 26 | 1 | 41 | 0,7860 | -0,0238 | 0,0000 |
| Rv1695 | ppnK | 48S>L | 0 | 26 | 1 | 41 | 0,7860 | -0,0238 | 0,0000 |
| Rv1697 | Rv1697 | 262R>L | 0 | 26 | 1 | 41 | 0,7860 | -0,0238 | 0,0000 |
| Rv1699 | pyrG | 58M>V | 0 | 26 | 1 | 41 | 0,7860 | -0,0238 | 0,0000 |
| Rv1702c | Rv1702c | 3S>L | 0 | 26 | 1 | 41 | 0,7860 | -0,0238 | 0,0000 |
| Rv1703c | Rv1703c | 194K>T | 0 | 26 | 1 | 41 | 0,7860 | -0,0238 | 0,0000 |
| Rv1704c | cycA | 161W>* | 0 | 26 | 1 | 41 | 0,7860 | -0,0238 | 0,0000 |
| Rv1704c | cycA | 207T>A | 0 | 26 | 1 | 41 | 0,7860 | -0,0238 | 0,0000 |
| Rv1706c | PPE23 | 223V>L | 0 | 26 | 1 | 41 | 0,7860 | -0,0238 | 0,0000 |
| Rv1709 | Rv1709 | 260Q>H | 0 | 26 | 1 | 41 | 0,7860 | -0,0238 | 0,0000 |
| Rv1709 | Rv1709 | 56V>L | 0 | 26 | 1 | 41 | 0,7860 | -0,0238 | 0,0000 |
| Rv1714 | Rv1714 | 2E>G | 0 | 26 | 1 | 41 | 0,7860 | -0,0238 | 0,0000 |
| Rv1722 | Rv1722 | 426A>V | 0 | 26 | 1 | 41 | 0,7860 | -0,0238 | 0,0000 |
| Rv1724c | Rv1724c | 124E>Q | 0 | 26 | 1 | 41 | 0,7860 | -0,0238 | 0,0000 |
| Rv1724c | Rv1724c | 60Q>E | 0 | 26 | 1 | 41 | 0,7860 | -0,0238 | 0,0000 |
| Rv1731 | gabD2 | 26T>P | 0 | 26 | 1 | 41 | 0,7860 | -0,0238 | 0,0000 |
| Rv1744c | Rv1744c | 69T>S | 0 | 26 | 1 | 41 | 0,7860 | -0,0238 | 0,0000 |

| Rv1747 | Rv1747 | 146Q>K | 0 | 26 | 1 | 41 | 0,7860 | -0,0238 | 0,0000 |
| --- | --- | --- | --- | --- | --- | --- | --- | --- | --- |
| Rv1747 | Rv1747 | 6P>L | 0 | 26 | 1 | 41 | 0,7860 | -0,0238 | 0,0000 |
| Rv1751 | Rv1751 | 249F>V | 0 | 26 | 1 | 41 | 0,7860 | -0,0238 | 0,0000 |
| Rv1753c | PPE24 | 934P>T | 0 | 26 | 1 | 41 | 0,7860 | -0,0238 | 0,0000 |
| Rv1754c | Rv1754c | 25R>G | 0 | 26 | 1 | 41 | 0,7860 | -0,0238 | 0,0000 |
| Rv1754c | Rv1754c | 544Y>D | 0 | 26 | 1 | 41 | 0,7860 | -0,0238 | 0,0000 |
| Rv1759c | wag22 | 393F>V | 0 | 26 | 1 | 41 | 0,7860 | -0,0238 | 0,0000 |
| Rv1760 | Rv1760 | 346C>G | 0 | 26 | 1 | 41 | 0,7860 | -0,0238 | 0,0000 |
| Rv1768 | PE_PGRS31 | 132N>D | 0 | 26 | 1 | 41 | 0,7860 | -0,0238 | 0,0000 |
| Rv1771 | Rv1771 | 194T>A | 0 | 26 | 1 | 41 | 0,7860 | -0,0238 | 0,0000 |
| Rv1774 | Rv1774 | 180A>V | 0 | 26 | 1 | 41 | 0,7860 | -0,0238 | 0,0000 |
| Rv1781c | malQ | 271R>W | 0 | 26 | 1 | 41 | 0,7860 | -0,0238 | 0,0000 |
| Rv1784 | Rv1784 | 308N>S | 0 | 26 | 1 | 41 | 0,7860 | -0,0238 | 0,0000 |
| Rv1785c | cyp143 | 345S>L | 0 | 26 | 1 | 41 | 0,7860 | -0,0238 | 0,0000 |
| Rv1795 | Rv1795 | 128S>R | 0 | 26 | 1 | 41 | 0,7860 | -0,0238 | 0,0000 |
| Rv1796 | mycP5 | 31L>V | 0 | 26 | 1 | 41 | 0,7860 | -0,0238 | 0,0000 |
| Rv1802 | PPE30 | 336V>I | 0 | 26 | 1 | 41 | 0,7860 | -0,0238 | 0,0000 |
| Rv1807 | PPE31 | 188H>R | 0 | 26 | 1 | 41 | 0,7860 | -0,0238 | 0,0000 |
| Rv1814 | erg3 | 245T>A | 0 | 26 | 1 | 41 | 0,7860 | -0,0238 | 0,0000 |
| Rv1815 | Rv1815 | 32V>A | 0 | 26 | 1 | 41 | 0,7860 | -0,0238 | 0,0000 |
| Rv1816 | Rv1816 | 3Q>P | 0 | 26 | 1 | 41 | 0,7860 | -0,0238 | 0,0000 |
| Rv1829 | Rv1829 | 163A>V | 0 | 26 | 1 | 41 | 0,7860 | -0,0238 | 0,0000 |
| Rv1832 | gcvB | 633L>I | 0 | 26 | 1 | 41 | 0,7860 | -0,0238 | 0,0000 |
| Rv1832 | gcvB | 657D>N | 0 | 26 | 1 | 41 | 0,7860 | -0,0238 | 0,0000 |
| Rv1835c | Rv1835c | 148G>A | 0 | 26 | 1 | 41 | 0,7860 | -0,0238 | 0,0000 |
| Rv1835c | Rv1835c | 303Y>* | 0 | 26 | 1 | 41 | 0,7860 | -0,0238 | 0,0000 |
| Rv1840c | PE_PGRS34 | 112P>A | 0 | 26 | 1 | 41 | 0,7860 | -0,0238 | 0,0000 |
| Rv1840c | PE_PGRS34 | 22G>R | 0 | 26 | 1 | 41 | 0,7860 | -0,0238 | 0,0000 |
| Rv1842c | Rv1842c | 384P>L | 0 | 26 | 1 | 41 | 0,7860 | -0,0238 | 0,0000 |
| Rv1843c | guaB1 | 212S>N | 0 | 26 | 1 | 41 | 0,7860 | -0,0238 | 0,0000 |
| Rv1844c | gnd1 | 226D>N | 0 | 26 | 1 | 41 | 0,7860 | -0,0238 | 0,0000 |
| Rv1844c | gnd1 | 70A>V | 0 | 26 | 1 | 41 | 0,7860 | -0,0238 | 0,0000 |
| Rv1850 | ureC | 110M>I | 0 | 26 | 1 | 41 | 0,7860 | -0,0238 | 0,0000 |

| Rv1859 | modC | 258S>A | 0 | 26 | 1 | 41 | 0,7860 | -0,0238 | 0,0000 |
| --- | --- | --- | --- | --- | --- | --- | --- | --- | --- |
| Rv1859 | modC | 258S>F | 0 | 26 | 1 | 41 | 0,7860 | -0,0238 | 0,0000 |
| Rv1864c | Rv1864c | 132E>A | 0 | 26 | 1 | 41 | 0,7860 | -0,0238 | 0,0000 |
| Rv1868 | Rv1868 | 514R>T | 0 | 26 | 1 | 41 | 0,7860 | -0,0238 | 0,0000 |
| Rv1878 | glnA3 | 151P>L | 0 | 26 | 1 | 41 | 0,7860 | -0,0238 | 0,0000 |
| Rv1879 | Rv1879 | 152G>S | 0 | 26 | 1 | 41 | 0,7860 | -0,0238 | 0,0000 |
| Rv1880c | cyp140 | 139A>V | 0 | 26 | 1 | 41 | 0,7860 | -0,0238 | 0,0000 |
| Rv1880c | cyp140 | 430L>R | 0 | 26 | 1 | 41 | 0,7860 | -0,0238 | 0,0000 |
| Rv1881c | lppE | 40V>A | 0 | 26 | 1 | 41 | 0,7860 | -0,0238 | 0,0000 |
| Rv1881c | lppE | 75G>S | 0 | 26 | 1 | 41 | 0,7860 | -0,0238 | 0,0000 |
| Rv1882c | Rv1882c | 237T>I | 0 | 26 | 1 | 41 | 0,7860 | -0,0238 | 0,0000 |
| Rv1888A | Rv1888A | 17A>P | 0 | 26 | 1 | 41 | 0,7860 | -0,0238 | 0,0000 |
| Rv1889c | Rv1889c | 41F>S | 0 | 26 | 1 | 41 | 0,7860 | -0,0238 | 0,0000 |
| Rv1895 | Rv1895 | 28V>M | 0 | 26 | 1 | 41 | 0,7860 | -0,0238 | 0,0000 |
| Rv1896c | Rv1896c | 29V>A | 0 | 26 | 1 | 41 | 0,7860 | -0,0238 | 0,0000 |
| Rv1901 | cinA | 369G>S | 0 | 26 | 1 | 41 | 0,7860 | -0,0238 | 0,0000 |
| Rv1908c | katG | 109A>V | 0 | 26 | 1 | 41 | 0,7860 | -0,0238 | 0,0000 |
| Rv1908c | katG | 121G>S | 0 | 26 | 1 | 41 | 0,7860 | -0,0238 | 0,0000 |
| Rv1908c | katG | 452E>Q | 0 | 26 | 1 | 41 | 0,7860 | -0,0238 | 0,0000 |
| Rv1909c | furA | 151*>G | 0 | 26 | 1 | 41 | 0,7860 | -0,0238 | 0,0000 |
| Rv1915 | aceAa | 366R>Q | 0 | 26 | 1 | 41 | 0,7860 | -0,0238 | 0,0000 |
| Rv1916 | aceAb | 113D>E | 0 | 26 | 1 | 41 | 0,7860 | -0,0238 | 0,0000 |
| Rv1916 | aceAb | 185R>C | 0 | 26 | 1 | 41 | 0,7860 | -0,0238 | 0,0000 |
| Rv1917c | PPE34 | 129N>D | 0 | 26 | 1 | 41 | 0,7860 | -0,0238 | 0,0000 |
| Rv1918c | PPE35 | 345G>D | 0 | 26 | 1 | 41 | 0,7860 | -0,0238 | 0,0000 |
| Rv1920 | Rv1920 | 15S>P | 0 | 26 | 1 | 41 | 0,7860 | -0,0238 | 0,0000 |
| Rv1920 | Rv1920 | 179G>S | 0 | 26 | 1 | 41 | 0,7860 | -0,0238 | 0,0000 |
| Rv1923 | lipD | 105K>N | 0 | 26 | 1 | 41 | 0,7860 | -0,0238 | 0,0000 |
| Rv1929c | Rv1929c | 36D>V | 0 | 26 | 1 | 41 | 0,7860 | -0,0238 | 0,0000 |
| Rv1931c | Rv1931c | 105H>Q | 0 | 26 | 1 | 41 | 0,7860 | -0,0238 | 0,0000 |
| Rv1935c | echA13 | 69D>N | 0 | 26 | 1 | 41 | 0,7860 | -0,0238 | 0,0000 |
| Rv1942c | Rv1942c | 83V>L | 0 | 26 | 1 | 41 | 0,7860 | -0,0238 | 0,0000 |
| Rv1945 | Rv1945 | 285R>K | 0 | 26 | 1 | 41 | 0,7860 | -0,0238 | 0,0000 |

| Rv1946c | lppG | 105N>H | 0 | 26 | 1 | 41 | 0,7860 | -0,0238 | 0,0000 |
| --- | --- | --- | --- | --- | --- | --- | --- | --- | --- |
| Rv1949c | Rv1949c | 140A>V | 0 | 26 | 1 | 41 | 0,7860 | -0,0238 | 0,0000 |
| Rv1956 | Rv1956 | 91A>V | 0 | 26 | 1 | 41 | 0,7860 | -0,0238 | 0,0000 |
| Rv1966 | mce3A | 266S>R | 0 | 26 | 1 | 41 | 0,7860 | -0,0238 | 0,0000 |
| Rv1968 | mce3C | 387P>S | 0 | 26 | 1 | 41 | 0,7860 | -0,0238 | 0,0000 |
| Rv1969 | mce3D | 378V>A | 0 | 26 | 1 | 41 | 0,7860 | -0,0238 | 0,0000 |
| Rv1973 | Rv1973 | 88V>A | 0 | 26 | 1 | 41 | 0,7860 | -0,0238 | 0,0000 |
| Rv1978 | Rv1978 | 69I>M | 0 | 26 | 1 | 41 | 0,7860 | -0,0238 | 0,0000 |
| Rv1979c | Rv1979c | 416V>A | 0 | 26 | 1 | 41 | 0,7860 | -0,0238 | 0,0000 |
| Rv1979c | Rv1979c | 426V>I | 0 | 26 | 1 | 41 | 0,7860 | -0,0238 | 0,0000 |
| Rv1980c | mpt64 | 12V>I | 0 | 26 | 1 | 41 | 0,7860 | -0,0238 | 0,0000 |
| Rv1982c | Rv1982c | 128F>L | 0 | 26 | 1 | 41 | 0,7860 | -0,0238 | 0,0000 |
| Rv1983 | PE_PGRS35 | 423L>R | 0 | 26 | 1 | 41 | 0,7860 | -0,0238 | 0,0000 |
| Rv1984c | cfp21 | 118S>W | 0 | 26 | 1 | 41 | 0,7860 | -0,0238 | 0,0000 |
| Rv1985c | Rv1985c | 158H>Y | 0 | 26 | 1 | 41 | 0,7860 | -0,0238 | 0,0000 |
| Rv1997 | ctpF | 889I>M | 0 | 26 | 1 | 41 | 0,7860 | -0,0238 | 0,0000 |
| Rv1998c | Rv1998c | 21V>A | 0 | 26 | 1 | 41 | 0,7860 | -0,0238 | 0,0000 |
| Rv1999c | Rv1999c | 47A>T | 0 | 26 | 1 | 41 | 0,7860 | -0,0238 | 0,0000 |
| Rv2000 | Rv2000 | 31K>T | 0 | 26 | 1 | 41 | 0,7860 | -0,0238 | 0,0000 |
| Rv2006 | otsB1 | 1263P>S | 0 | 26 | 1 | 41 | 0,7860 | -0,0238 | 0,0000 |
| Rv2006 | otsB1 | 374A>S | 0 | 26 | 1 | 41 | 0,7860 | -0,0238 | 0,0000 |
| Rv2006 | otsB1 | 617D>N | 0 | 26 | 1 | 41 | 0,7860 | -0,0238 | 0,0000 |
| Rv2014 | Rv2014 | 103S>A | 0 | 26 | 1 | 41 | 0,7860 | -0,0238 | 0,0000 |
| Rv2015c | Rv2015c | 116S>L | 0 | 26 | 1 | 41 | 0,7860 | -0,0238 | 0,0000 |
| Rv2017 | Rv2017 | 227F>L | 0 | 26 | 1 | 41 | 0,7860 | -0,0238 | 0,0000 |
| Rv2025c | Rv2025c | 103T>A | 0 | 26 | 1 | 41 | 0,7860 | -0,0238 | 0,0000 |
| Rv2027c | Rv2027c | 416P>H | 0 | 26 | 1 | 41 | 0,7860 | -0,0238 | 0,0000 |
| Rv2035 | Rv2035 | 22A>P | 0 | 26 | 1 | 41 | 0,7860 | -0,0238 | 0,0000 |
| Rv2035 | Rv2035 | 99A>T | 0 | 26 | 1 | 41 | 0,7860 | -0,0238 | 0,0000 |
| Rv2038c | Rv2038c | 334S>T | 0 | 26 | 1 | 41 | 0,7860 | -0,0238 | 0,0000 |
| Rv2040c | Rv2040c | 218V>I | 0 | 26 | 1 | 41 | 0,7860 | -0,0238 | 0,0000 |
| Rv2042c | Rv2042c | 27G>C | 0 | 26 | 1 | 41 | 0,7860 | -0,0238 | 0,0000 |
| Rv2043c | pncA | 160T>A | 0 | 26 | 1 | 41 | 0,7860 | -0,0238 | 0,0000 |

| Rv2043c | pncA | 48K>T | 0 | 26 | 1 | 41 | 0,7860 | -0,0238 | 0,0000 |
| --- | --- | --- | --- | --- | --- | --- | --- | --- | --- |
| Rv2045c | lipT | 59P>A | 0 | 26 | 1 | 41 | 0,7860 | -0,0238 | 0,0000 |
| Rv2047c | Rv2047c | 103S>N | 0 | 26 | 1 | 41 | 0,7860 | -0,0238 | 0,0000 |
| Rv2047c | Rv2047c | 762Q>R | 0 | 26 | 1 | 41 | 0,7860 | -0,0238 | 0,0000 |
| Rv2048c | pks12 | 1133D>N | 0 | 26 | 1 | 41 | 0,7860 | -0,0238 | 0,0000 |
| Rv2048c | pks12 | 3527L>F | 0 | 26 | 1 | 41 | 0,7860 | -0,0238 | 0,0000 |
| Rv2048c | pks12 | 3682C>R | 0 | 26 | 1 | 41 | 0,7860 | -0,0238 | 0,0000 |
| Rv2052c | Rv2052c | 83P>L | 0 | 26 | 1 | 41 | 0,7860 | -0,0238 | 0,0000 |
| Rv2067c | Rv2067c | 206Y>D | 0 | 26 | 1 | 41 | 0,7860 | -0,0238 | 0,0000 |
| Rv2077A | Rv2077A | 74A>G | 0 | 26 | 1 | 41 | 0,7860 | -0,0238 | 0,0000 |
| Rv2077c | Rv2077c | 112S>L | 0 | 26 | 1 | 41 | 0,7860 | -0,0238 | 0,0000 |
| Rv2077c | Rv2077c | 308L>P | 0 | 26 | 1 | 41 | 0,7860 | -0,0238 | 0,0000 |
| Rv2090 | Rv2090 | 24D>H | 0 | 26 | 1 | 41 | 0,7860 | -0,0238 | 0,0000 |
| Rv2095c | Rv2095c | 244A>V | 0 | 26 | 1 | 41 | 0,7860 | -0,0238 | 0,0000 |
| Rv2100 | Rv2100 | 120S>A | 0 | 26 | 1 | 41 | 0,7860 | -0,0238 | 0,0000 |
| Rv2113 | Rv2113 | 19A>P | 0 | 26 | 1 | 41 | 0,7860 | -0,0238 | 0,0000 |
| Rv2113 | Rv2113 | 240P>A | 0 | 26 | 1 | 41 | 0,7860 | -0,0238 | 0,0000 |
| Rv2113 | Rv2113 | 7R>C | 0 | 26 | 1 | 41 | 0,7860 | -0,0238 | 0,0000 |
| Rv2122c | hisE | 89V>I | 0 | 26 | 1 | 41 | 0,7860 | -0,0238 | 0,0000 |
| Rv2123 | PPE37 | 39A>V | 0 | 26 | 1 | 41 | 0,7860 | -0,0238 | 0,0000 |
| Rv2124c | metH | 388C>G | 0 | 26 | 1 | 41 | 0,7860 | -0,0238 | 0,0000 |
| Rv2130c | cysS | 175E>K | 0 | 26 | 1 | 41 | 0,7860 | -0,0238 | 0,0000 |
| Rv2136c | uppP | 109R>W | 0 | 26 | 1 | 41 | 0,7860 | -0,0238 | 0,0000 |
| Rv2151c | ftsQ | 242L>V | 0 | 26 | 1 | 41 | 0,7860 | -0,0238 | 0,0000 |
| Rv2157c | murF | 22P>L | 0 | 26 | 1 | 41 | 0,7860 | -0,0238 | 0,0000 |
| Rv2158c | murE | 287R>L | 0 | 26 | 1 | 41 | 0,7860 | -0,0238 | 0,0000 |
| Rv2158c | murE | 49E>D | 0 | 26 | 1 | 41 | 0,7860 | -0,0238 | 0,0000 |
| Rv2159c | Rv2159c | 76A>T | 0 | 26 | 1 | 41 | 0,7860 | -0,0238 | 0,0000 |
| Rv2161c | Rv2161c | 71A>P | 0 | 26 | 1 | 41 | 0,7860 | -0,0238 | 0,0000 |
| Rv2163c | pbpB | 152A>E | 0 | 26 | 1 | 41 | 0,7860 | -0,0238 | 0,0000 |
| Rv2180c | Rv2180c | 241R>C | 0 | 26 | 1 | 41 | 0,7860 | -0,0238 | 0,0000 |
| Rv2181 | Rv2181 | 186P>R | 0 | 26 | 1 | 41 | 0,7860 | -0,0238 | 0,0000 |
| Rv2181 | Rv2181 | 211L>V | 0 | 26 | 1 | 41 | 0,7860 | -0,0238 | 0,0000 |

| Rv2186c | Rv2186c | 37R>G | 0 | 26 | 1 | 41 | 0,7860 | -0,0238 | 0,0000 |
| --- | --- | --- | --- | --- | --- | --- | --- | --- | --- |
| Rv2191 | Rv2191 | 327R>K | 0 | 26 | 1 | 41 | 0,7860 | -0,0238 | 0,0000 |
| Rv2196 | qcrB | 491K>T | 0 | 26 | 1 | 41 | 0,7860 | -0,0238 | 0,0000 |
| Rv2197c | Rv2197c | 152V>A | 0 | 26 | 1 | 41 | 0,7860 | -0,0238 | 0,0000 |
| Rv2201 | asnB | 216G>R | 0 | 26 | 1 | 41 | 0,7860 | -0,0238 | 0,0000 |
| Rv2209 | Rv2209 | 318Q>P | 0 | 26 | 1 | 41 | 0,7860 | -0,0238 | 0,0000 |
| Rv2211c | gcvT | 335Q>H | 0 | 26 | 1 | 41 | 0,7860 | -0,0238 | 0,0000 |
| Rv2216 | Rv2216 | 226A>T | 0 | 26 | 1 | 41 | 0,7860 | -0,0238 | 0,0000 |
| Rv2217 | lipB | 221V>A | 0 | 26 | 1 | 41 | 0,7860 | -0,0238 | 0,0000 |
| Rv2218 | lipA | 4A>S | 0 | 26 | 1 | 41 | 0,7860 | -0,0238 | 0,0000 |
| Rv2219 | Rv2219 | 63G>R | 0 | 26 | 1 | 41 | 0,7860 | -0,0238 | 0,0000 |
| Rv2220 | glnA1 | 43D>G | 0 | 26 | 1 | 41 | 0,7860 | -0,0238 | 0,0000 |
| Rv2221c | glnE | 414E>K | 0 | 26 | 1 | 41 | 0,7860 | -0,0238 | 0,0000 |
| Rv2221c | glnE | 596G>V | 0 | 26 | 1 | 41 | 0,7860 | -0,0238 | 0,0000 |
| Rv2226 | Rv2226 | 225P>S | 0 | 26 | 1 | 41 | 0,7860 | -0,0238 | 0,0000 |
| Rv2242 | Rv2242 | 345P>S | 0 | 26 | 1 | 41 | 0,7860 | -0,0238 | 0,0000 |
| Rv2247 | accD6 | 113Q>R | 0 | 26 | 1 | 41 | 0,7860 | -0,0238 | 0,0000 |
| Rv2252 | Rv2252 | 199E>D | 0 | 26 | 1 | 41 | 0,7860 | -0,0238 | 0,0000 |
| Rv2253 | Rv2253 | 25A>P | 0 | 26 | 1 | 41 | 0,7860 | -0,0238 | 0,0000 |
| Rv2257c | Rv2257c | 73R>C | 0 | 26 | 1 | 41 | 0,7860 | -0,0238 | 0,0000 |
| Rv2259 | adhE2 | 292V>I | 0 | 26 | 1 | 41 | 0,7860 | -0,0238 | 0,0000 |
| Rv2266 | cyp124 | 74K>R | 0 | 26 | 1 | 41 | 0,7860 | -0,0238 | 0,0000 |
| Rv2267c | Rv2267c | 310D>Y | 0 | 26 | 1 | 41 | 0,7860 | -0,0238 | 0,0000 |
| Rv2267c | Rv2267c | 46H>R | 0 | 26 | 1 | 41 | 0,7860 | -0,0238 | 0,0000 |
| Rv2268c | cyp128 | 296T>I | 0 | 26 | 1 | 41 | 0,7860 | -0,0238 | 0,0000 |
| Rv2275 | Rv2275 | 143N>D | 0 | 26 | 1 | 41 | 0,7860 | -0,0238 | 0,0000 |
| Rv2281 | pitB | 22A>T | 0 | 26 | 1 | 41 | 0,7860 | -0,0238 | 0,0000 |
| Rv2281 | pitB | 30G>R | 0 | 26 | 1 | 41 | 0,7860 | -0,0238 | 0,0000 |
| Rv2282c | Rv2282c | 105S>T | 0 | 26 | 1 | 41 | 0,7860 | -0,0238 | 0,0000 |
| Rv2290 | lppO | 123M>L | 0 | 26 | 1 | 41 | 0,7860 | -0,0238 | 0,0000 |
| Rv2294 | Rv2294 | 38M>T | 0 | 26 | 1 | 41 | 0,7860 | -0,0238 | 0,0000 |
| Rv2297 | Rv2297 | 105G>R | 0 | 26 | 1 | 41 | 0,7860 | -0,0238 | 0,0000 |
| Rv2300c | Rv2300c | 190H>D | 0 | 26 | 1 | 41 | 0,7860 | -0,0238 | 0,0000 |

| Rv2307B | Rv2307B | 104W>C | 0 | 26 | 1 | 41 | 0,7860 | -0,0238 | 0,0000 |
| --- | --- | --- | --- | --- | --- | --- | --- | --- | --- |
| Rv2310 | Rv2310 | 109T>I | 0 | 26 | 1 | 41 | 0,7860 | -0,0238 | 0,0000 |
| Rv2311 | Rv2311 | 38N>S | 0 | 26 | 1 | 41 | 0,7860 | -0,0238 | 0,0000 |
| Rv2315c | Rv2315c | 99A>V | 0 | 26 | 1 | 41 | 0,7860 | -0,0238 | 0,0000 |
| Rv2320c | rocE | 120L>Q | 0 | 26 | 1 | 41 | 0,7860 | -0,0238 | 0,0000 |
| Rv2323c | Rv2323c | 218P>L | 0 | 26 | 1 | 41 | 0,7860 | -0,0238 | 0,0000 |
| Rv2323c | Rv2323c | 52F>L | 0 | 26 | 1 | 41 | 0,7860 | -0,0238 | 0,0000 |
| Rv2325c | Rv2325c | 68A>V | 0 | 26 | 1 | 41 | 0,7860 | -0,0238 | 0,0000 |
| Rv2328 | PE23 | 212T>S | 0 | 26 | 1 | 41 | 0,7860 | -0,0238 | 0,0000 |
| Rv2328 | PE23 | 239A>V | 0 | 26 | 1 | 41 | 0,7860 | -0,0238 | 0,0000 |
| Rv2328 | PE23 | 258N>I | 0 | 26 | 1 | 41 | 0,7860 | -0,0238 | 0,0000 |
| Rv2334 | cysK1 | 303P>T | 0 | 26 | 1 | 41 | 0,7860 | -0,0238 | 0,0000 |
| Rv2336 | Rv2336 | 177D>E | 0 | 26 | 1 | 41 | 0,7860 | -0,0238 | 0,0000 |
| Rv2337c | Rv2337c | 301V>M | 0 | 26 | 1 | 41 | 0,7860 | -0,0238 | 0,0000 |
| Rv2338c | moeW | 183T>M | 0 | 26 | 1 | 41 | 0,7860 | -0,0238 | 0,0000 |
| Rv2340c | PE_PGRS39 | 128G>S | 0 | 26 | 1 | 41 | 0,7860 | -0,0238 | 0,0000 |
| Rv2340c | PE_PGRS39 | 190A>P | 0 | 26 | 1 | 41 | 0,7860 | -0,0238 | 0,0000 |
| Rv2343c | dnaG | 195G>D | 0 | 26 | 1 | 41 | 0,7860 | -0,0238 | 0,0000 |
| Rv2343c | dnaG | 388D>N | 0 | 26 | 1 | 41 | 0,7860 | -0,0238 | 0,0000 |
| Rv2345 | Rv2345 | 118G>D | 0 | 26 | 1 | 41 | 0,7860 | -0,0238 | 0,0000 |
| Rv2346c | esxO | 20Q>L | 0 | 26 | 1 | 41 | 0,7860 | -0,0238 | 0,0000 |
| Rv2349c | plcC | 118Q>L | 0 | 26 | 1 | 41 | 0,7860 | -0,0238 | 0,0000 |
| Rv2365c | Rv2365c | 29R>G | 0 | 26 | 1 | 41 | 0,7860 | -0,0238 | 0,0000 |
| Rv2376c | cfp2 | 91E>G | 0 | 26 | 1 | 41 | 0,7860 | -0,0238 | 0,0000 |
| Rv2379c | mbtF | 555M>V | 0 | 26 | 1 | 41 | 0,7860 | -0,0238 | 0,0000 |
| Rv2380c | mbtE | 285A>V | 0 | 26 | 1 | 41 | 0,7860 | -0,0238 | 0,0000 |
| Rv2380c | mbtE | 388G>D | 0 | 26 | 1 | 41 | 0,7860 | -0,0238 | 0,0000 |
| Rv2394 | ggtB | 476D>G | 0 | 26 | 1 | 41 | 0,7860 | -0,0238 | 0,0000 |
| Rv2394 | ggtB | 68Y>* | 0 | 26 | 1 | 41 | 0,7860 | -0,0238 | 0,0000 |
| Rv2399c | cysT | 108V>A | 0 | 26 | 1 | 41 | 0,7860 | -0,0238 | 0,0000 |
| Rv2402 | Rv2402 | 160D>G | 0 | 26 | 1 | 41 | 0,7860 | -0,0238 | 0,0000 |
| Rv2404c | lepA | 227V>L | 0 | 26 | 1 | 41 | 0,7860 | -0,0238 | 0,0000 |
| Rv2405 | Rv2405 | 45V>A | 0 | 26 | 1 | 41 | 0,7860 | -0,0238 | 0,0000 |

| Rv2416c | eis | 388A>V | 0 | 26 | 1 | 41 | 0,7860 | -0,0238 | 0,0000 |
| --- | --- | --- | --- | --- | --- | --- | --- | --- | --- |
| Rv2425c | Rv2425c | 479P>L | 0 | 26 | 1 | 41 | 0,7860 | -0,0238 | 0,0000 |
| Rv2426c | Rv2426c | 18S>P | 0 | 26 | 1 | 41 | 0,7860 | -0,0238 | 0,0000 |
| Rv2427c | proA | 332E>A | 0 | 26 | 1 | 41 | 0,7860 | -0,0238 | 0,0000 |
| Rv2435c | Rv2435c | 277Q>R | 0 | 26 | 1 | 41 | 0,7860 | -0,0238 | 0,0000 |
| Rv2435c | Rv2435c | 452I>V | 0 | 26 | 1 | 41 | 0,7860 | -0,0238 | 0,0000 |
| Rv2435c | Rv2435c | 500D>Y | 0 | 26 | 1 | 41 | 0,7860 | -0,0238 | 0,0000 |
| Rv2436 | rbsK | 232V>A | 0 | 26 | 1 | 41 | 0,7860 | -0,0238 | 0,0000 |
| Rv2443 | dctA | 361A>T | 0 | 26 | 1 | 41 | 0,7860 | -0,0238 | 0,0000 |
| Rv2444c | rne | 375V>L | 0 | 26 | 1 | 41 | 0,7860 | -0,0238 | 0,0000 |
| Rv2445c | ndk | 21E>K | 0 | 26 | 1 | 41 | 0,7860 | -0,0238 | 0,0000 |
| Rv2445c | ndk | 95V>M | 0 | 26 | 1 | 41 | 0,7860 | -0,0238 | 0,0000 |
| Rv2449c | Rv2449c | 51E>G | 0 | 26 | 1 | 41 | 0,7860 | -0,0238 | 0,0000 |
| Rv2450c | rpfE | 149Q>* | 0 | 26 | 1 | 41 | 0,7860 | -0,0238 | 0,0000 |
| Rv2450c | rpfE | 46F>C | 0 | 26 | 1 | 41 | 0,7860 | -0,0238 | 0,0000 |
| Rv2469c | Rv2469c | 34L>I | 0 | 26 | 1 | 41 | 0,7860 | -0,0238 | 0,0000 |
| Rv2471 | aglA | 515E>K | 0 | 26 | 1 | 41 | 0,7860 | -0,0238 | 0,0000 |
| Rv2473 | Rv2473 | 111P>L | 0 | 26 | 1 | 41 | 0,7860 | -0,0238 | 0,0000 |
| Rv2474c | Rv2474c | 75L>F | 0 | 26 | 1 | 41 | 0,7860 | -0,0238 | 0,0000 |
| Rv2476c | gdh | 423D>N | 0 | 26 | 1 | 41 | 0,7860 | -0,0238 | 0,0000 |
| Rv2476c | gdh | 521K>N | 0 | 26 | 1 | 41 | 0,7860 | -0,0238 | 0,0000 |
| Rv2477c | Rv2477c | 440A>T | 0 | 26 | 1 | 41 | 0,7860 | -0,0238 | 0,0000 |
| Rv2481c | Rv2481c | 49S>T | 0 | 26 | 1 | 41 | 0,7860 | -0,0238 | 0,0000 |
| Rv2484c | Rv2484c | 369S>T | 0 | 26 | 1 | 41 | 0,7860 | -0,0238 | 0,0000 |
| Rv2486 | echA14 | 65G>R | 0 | 26 | 1 | 41 | 0,7860 | -0,0238 | 0,0000 |
| Rv2487c | PE_PGRS42 | 613G>A | 0 | 26 | 1 | 41 | 0,7860 | -0,0238 | 0,0000 |
| Rv2488c | Rv2488c | 216D>G | 0 | 26 | 1 | 41 | 0,7860 | -0,0238 | 0,0000 |
| Rv2490c | PE_PGRS43 | 37T>M | 0 | 26 | 1 | 41 | 0,7860 | -0,0238 | 0,0000 |
| Rv2492 | Rv2492 | 119S>R | 0 | 26 | 1 | 41 | 0,7860 | -0,0238 | 0,0000 |
| Rv2492 | Rv2492 | 79M>I | 0 | 26 | 1 | 41 | 0,7860 | -0,0238 | 0,0000 |
| Rv2494 | Rv2494 | 78R>H | 0 | 26 | 1 | 41 | 0,7860 | -0,0238 | 0,0000 |
| Rv2510c | Rv2510c | 396V>M | 0 | 26 | 1 | 41 | 0,7860 | -0,0238 | 0,0000 |
| Rv2519 | PE26 | 351G>D | 0 | 26 | 1 | 41 | 0,7860 | -0,0238 | 0,0000 |

| Rv2524c | fas | 1148D>Y | 0 | 26 | 1 | 41 | 0,7860 | -0,0238 | 0,0000 |
| --- | --- | --- | --- | --- | --- | --- | --- | --- | --- |
| Rv2524c | fas | 2195T>N | 0 | 26 | 1 | 41 | 0,7860 | -0,0238 | 0,0000 |
| Rv2524c | fas | 288T>A | 0 | 26 | 1 | 41 | 0,7860 | -0,0238 | 0,0000 |
| Rv2528c | mrr | 52Q>L | 0 | 26 | 1 | 41 | 0,7860 | -0,0238 | 0,0000 |
| Rv2531c | Rv2531c | 677R>C | 0 | 26 | 1 | 41 | 0,7860 | -0,0238 | 0,0000 |
| Rv2532c | Rv2532c | 133D>G | 0 | 26 | 1 | 41 | 0,7860 | -0,0238 | 0,0000 |
| Rv2539c | aroK | 118P>S | 0 | 26 | 1 | 41 | 0,7860 | -0,0238 | 0,0000 |
| Rv2542 | Rv2542 | 208T>A | 0 | 26 | 1 | 41 | 0,7860 | -0,0238 | 0,0000 |
| Rv2542 | Rv2542 | 339D>G | 0 | 26 | 1 | 41 | 0,7860 | -0,0238 | 0,0000 |
| Rv2544 | lppB | 43P>R | 0 | 26 | 1 | 41 | 0,7860 | -0,0238 | 0,0000 |
| Rv2544 | lppB | 44H>R | 0 | 26 | 1 | 41 | 0,7860 | -0,0238 | 0,0000 |
| Rv2549c | Rv2549c | 128V>A | 0 | 26 | 1 | 41 | 0,7860 | -0,0238 | 0,0000 |
| Rv2551c | Rv2551c | 125M>T | 0 | 26 | 1 | 41 | 0,7860 | -0,0238 | 0,0000 |
| Rv2552c | aroE | 17H>Y | 0 | 26 | 1 | 41 | 0,7860 | -0,0238 | 0,0000 |
| Rv2555c | alaS | 382S>F | 0 | 26 | 1 | 41 | 0,7860 | -0,0238 | 0,0000 |
| Rv2558 | Rv2558 | 209R>H | 0 | 26 | 1 | 41 | 0,7860 | -0,0238 | 0,0000 |
| Rv2563 | Rv2563 | 189Q>* | 0 | 26 | 1 | 41 | 0,7860 | -0,0238 | 0,0000 |
| Rv2567 | Rv2567 | 63A>S | 0 | 26 | 1 | 41 | 0,7860 | -0,0238 | 0,0000 |
| Rv2567 | Rv2567 | 737V>M | 0 | 26 | 1 | 41 | 0,7860 | -0,0238 | 0,0000 |
| Rv2574 | Rv2574 | 34E>Q | 0 | 26 | 1 | 41 | 0,7860 | -0,0238 | 0,0000 |
| Rv2577 | Rv2577 | 346C>G | 0 | 26 | 1 | 41 | 0,7860 | -0,0238 | 0,0000 |
| Rv2577 | Rv2577 | 376Y>D | 0 | 26 | 1 | 41 | 0,7860 | -0,0238 | 0,0000 |
| Rv2583c | relA | 268D>G | 0 | 26 | 1 | 41 | 0,7860 | -0,0238 | 0,0000 |
| Rv2583c | relA | 309G>C | 0 | 26 | 1 | 41 | 0,7860 | -0,0238 | 0,0000 |
| Rv2586c | secF | 153R>Q | 0 | 26 | 1 | 41 | 0,7860 | -0,0238 | 0,0000 |
| Rv2586c | secF | 20T>I | 0 | 26 | 1 | 41 | 0,7860 | -0,0238 | 0,0000 |
| Rv2587c | secD | 19V>M | 0 | 26 | 1 | 41 | 0,7860 | -0,0238 | 0,0000 |
| Rv2587c | secD | 62P>L | 0 | 26 | 1 | 41 | 0,7860 | -0,0238 | 0,0000 |
| Rv2602 | Rv2602 | 147*>C | 0 | 26 | 1 | 41 | 0,7860 | -0,0238 | 0,0000 |
| Rv2608 | PPE42 | 290Y>* | 0 | 26 | 1 | 41 | 0,7860 | -0,0238 | 0,0000 |
| Rv2608 | PPE42 | 75W>* | 0 | 26 | 1 | 41 | 0,7860 | -0,0238 | 0,0000 |
| Rv2611c | Rv2611c | 147W>L | 0 | 26 | 1 | 41 | 0,7860 | -0,0238 | 0,0000 |
| Rv2614c | thrS | 434R>S | 0 | 26 | 1 | 41 | 0,7860 | -0,0238 | 0,0000 |

| Rv2614c | thrS | 624V>L | 0 | 26 | 1 | 41 | 0,7860 | -0,0238 | 0,0000 |
| --- | --- | --- | --- | --- | --- | --- | --- | --- | --- |
| Rv2615c | PE_PGRS45 | 43Q>H | 0 | 26 | 1 | 41 | 0,7860 | -0,0238 | 0,0000 |
| Rv2623 | TB31.7 | 233N>D | 0 | 26 | 1 | 41 | 0,7860 | -0,0238 | 0,0000 |
| Rv2630 | Rv2630 | 168R>C | 0 | 26 | 1 | 41 | 0,7860 | -0,0238 | 0,0000 |
| Rv2631 | Rv2631 | 4V>A | 0 | 26 | 1 | 41 | 0,7860 | -0,0238 | 0,0000 |
| Rv2634c | PE_PGRS46 | 575G>A | 0 | 26 | 1 | 41 | 0,7860 | -0,0238 | 0,0000 |
| Rv2636 | Rv2636 | 21P>R | 0 | 26 | 1 | 41 | 0,7860 | -0,0238 | 0,0000 |
| Rv2643 | arsC | 165F>L | 0 | 26 | 1 | 41 | 0,7860 | -0,0238 | 0,0000 |
| Rv2643 | arsC | 499*>W | 0 | 26 | 1 | 41 | 0,7860 | -0,0238 | 0,0000 |
| Rv2644c | Rv2644c | 92P>S | 0 | 26 | 1 | 41 | 0,7860 | -0,0238 | 0,0000 |
| Rv2645 | Rv2645 | 2T>A | 0 | 26 | 1 | 41 | 0,7860 | -0,0238 | 0,0000 |
| Rv2655c | Rv2655c | 413T>I | 0 | 26 | 1 | 41 | 0,7860 | -0,0238 | 0,0000 |
| Rv2656c | Rv2656c | 94Q>E | 0 | 26 | 1 | 41 | 0,7860 | -0,0238 | 0,0000 |
| Rv2659c | Rv2659c | 265I>V | 0 | 26 | 1 | 41 | 0,7860 | -0,0238 | 0,0000 |
| Rv2667 | clpC2 | 156A>V | 0 | 26 | 1 | 41 | 0,7860 | -0,0238 | 0,0000 |
| Rv2674 | Rv2674 | 136V>L | 0 | 26 | 1 | 41 | 0,7860 | -0,0238 | 0,0000 |
| Rv2678c | hemE | 269A>T | 0 | 26 | 1 | 41 | 0,7860 | -0,0238 | 0,0000 |
| Rv2683 | Rv2683 | 50P>L | 0 | 26 | 1 | 41 | 0,7860 | -0,0238 | 0,0000 |
| Rv2686c | Rv2686c | 206V>I | 0 | 26 | 1 | 41 | 0,7860 | -0,0238 | 0,0000 |
| Rv2689c | Rv2689c | 56W>* | 0 | 26 | 1 | 41 | 0,7860 | -0,0238 | 0,0000 |
| Rv2693c | Rv2693c | 129R>L | 0 | 26 | 1 | 41 | 0,7860 | -0,0238 | 0,0000 |
| Rv2696c | Rv2696c | 68A>V | 0 | 26 | 1 | 41 | 0,7860 | -0,0238 | 0,0000 |
| Rv2700 | Rv2700 | 102G>S | 0 | 26 | 1 | 41 | 0,7860 | -0,0238 | 0,0000 |
| Rv2707 | Rv2707 | 300A>V | 0 | 26 | 1 | 41 | 0,7860 | -0,0238 | 0,0000 |
| Rv2712c | Rv2712c | 291G>C | 0 | 26 | 1 | 41 | 0,7860 | -0,0238 | 0,0000 |
| Rv2713 | sthA | 240T>A | 0 | 26 | 1 | 41 | 0,7860 | -0,0238 | 0,0000 |
| Rv2713 | sthA | 450K>T | 0 | 26 | 1 | 41 | 0,7860 | -0,0238 | 0,0000 |
| Rv2716 | Rv2716 | 67P>L | 0 | 26 | 1 | 41 | 0,7860 | -0,0238 | 0,0000 |
| Rv2720 | lexA | 122R>S | 0 | 26 | 1 | 41 | 0,7860 | -0,0238 | 0,0000 |
| Rv2720 | lexA | 176Q>K | 0 | 26 | 1 | 41 | 0,7860 | -0,0238 | 0,0000 |
| Rv2721c | Rv2721c | 239P>A | 0 | 26 | 1 | 41 | 0,7860 | -0,0238 | 0,0000 |
| Rv2723 | Rv2723 | 101Q>P | 0 | 26 | 1 | 41 | 0,7860 | -0,0238 | 0,0000 |
| Rv2724c | fadE20 | 259A>S | 0 | 26 | 1 | 41 | 0,7860 | -0,0238 | 0,0000 |

| Rv2725c | hflX | 280N>S | 0 | 26 | 1 | 41 | 0,7860 | -0,0238 | 0,0000 |
| --- | --- | --- | --- | --- | --- | --- | --- | --- | --- |
| Rv2728c | Rv2728c | 151N>K | 0 | 26 | 1 | 41 | 0,7860 | -0,0238 | 0,0000 |
| Rv2729c | Rv2729c | 108M>N | 0 | 26 | 1 | 41 | 0,7860 | -0,0238 | 0,0000 |
| Rv2729c | Rv2729c | 208L>R | 0 | 26 | 1 | 41 | 0,7860 | -0,0238 | 0,0000 |
| Rv2731 | Rv2731 | 305S>Y | 0 | 26 | 1 | 41 | 0,7860 | -0,0238 | 0,0000 |
| Rv2731 | Rv2731 | 50D>H | 0 | 26 | 1 | 41 | 0,7860 | -0,0238 | 0,0000 |
| Rv2733c | Rv2733c | 309E>G | 0 | 26 | 1 | 41 | 0,7860 | -0,0238 | 0,0000 |
| Rv2748c | ftsK | 198A>S | 0 | 26 | 1 | 41 | 0,7860 | -0,0238 | 0,0000 |
| Rv2748c | ftsK | 718A>G | 0 | 26 | 1 | 41 | 0,7860 | -0,0238 | 0,0000 |
| Rv2748c | ftsK | 98S>A | 0 | 26 | 1 | 41 | 0,7860 | -0,0238 | 0,0000 |
| Rv2751 | Rv2751 | 205F>S | 0 | 26 | 1 | 41 | 0,7860 | -0,0238 | 0,0000 |
| Rv2752c | Rv2752c | 123P>L | 0 | 26 | 1 | 41 | 0,7860 | -0,0238 | 0,0000 |
| Rv2752c | Rv2752c | 132Q>P | 0 | 26 | 1 | 41 | 0,7860 | -0,0238 | 0,0000 |
| Rv2752c | Rv2752c | 249I>S | 0 | 26 | 1 | 41 | 0,7860 | -0,0238 | 0,0000 |
| Rv2753c | dapA | 76A>V | 0 | 26 | 1 | 41 | 0,7860 | -0,0238 | 0,0000 |
| Rv2756c | hsdM | 227P>A | 0 | 26 | 1 | 41 | 0,7860 | -0,0238 | 0,0000 |
| Rv2764c | thyA | 157G>S | 0 | 26 | 1 | 41 | 0,7860 | -0,0238 | 0,0000 |
| Rv2775 | Rv2775 | 152W>R | 0 | 26 | 1 | 41 | 0,7860 | -0,0238 | 0,0000 |
| Rv2780 | ald | 198D>N | 0 | 26 | 1 | 41 | 0,7860 | -0,0238 | 0,0000 |
| Rv2780 | ald | 69D>E | 0 | 26 | 1 | 41 | 0,7860 | -0,0238 | 0,0000 |
| Rv2783c | gpsI | 732L>V | 0 | 26 | 1 | 41 | 0,7860 | -0,0238 | 0,0000 |
| Rv2786c | ribF | 207R>H | 0 | 26 | 1 | 41 | 0,7860 | -0,0238 | 0,0000 |
| Rv2789c | fadE21 | 96G>W | 0 | 26 | 1 | 41 | 0,7860 | -0,0238 | 0,0000 |
| Rv2790c | ltp1 | 284D>G | 0 | 26 | 1 | 41 | 0,7860 | -0,0238 | 0,0000 |
| Rv2791c | Rv2791c | 105G>S | 0 | 26 | 1 | 41 | 0,7860 | -0,0238 | 0,0000 |
| Rv2795c | Rv2795c | 272R>G | 0 | 26 | 1 | 41 | 0,7860 | -0,0238 | 0,0000 |
| Rv2796c | lppV | 46L>S | 0 | 26 | 1 | 41 | 0,7860 | -0,0238 | 0,0000 |
| Rv2797c | Rv2797c | 308T>S | 0 | 26 | 1 | 41 | 0,7860 | -0,0238 | 0,0000 |
| Rv2807 | Rv2807 | 292Y>N | 0 | 26 | 1 | 41 | 0,7860 | -0,0238 | 0,0000 |
| Rv2812 | Rv2812 | 232Y>H | 0 | 26 | 1 | 41 | 0,7860 | -0,0238 | 0,0000 |
| Rv2812 | Rv2812 | 410A>T | 0 | 26 | 1 | 41 | 0,7860 | -0,0238 | 0,0000 |
| Rv2818c | Rv2818c | 330E>V | 0 | 26 | 1 | 41 | 0,7860 | -0,0238 | 0,0000 |
| Rv2823c | Rv2823c | 101Y>D | 0 | 26 | 1 | 41 | 0,7860 | -0,0238 | 0,0000 |

| Rv2823c | Rv2823c | 377P>S | 0 | 26 | 1 | 41 | 0,7860 | -0,0238 | 0,0000 |
| --- | --- | --- | --- | --- | --- | --- | --- | --- | --- |
| Rv2825c | Rv2825c | 173A>E | 0 | 26 | 1 | 41 | 0,7860 | -0,0238 | 0,0000 |
| Rv2827c | Rv2827c | 52I>M | 0 | 26 | 1 | 41 | 0,7860 | -0,0238 | 0,0000 |
| Rv2832c | ugpC | 348V>M | 0 | 26 | 1 | 41 | 0,7860 | -0,0238 | 0,0000 |
| Rv2845c | proS | 195V>I | 0 | 26 | 1 | 41 | 0,7860 | -0,0238 | 0,0000 |
| Rv2847c | cysG | 256E>G | 0 | 26 | 1 | 41 | 0,7860 | -0,0238 | 0,0000 |
| Rv2850c | Rv2850c | 320G>S | 0 | 26 | 1 | 41 | 0,7860 | -0,0238 | 0,0000 |
| Rv2859c | Rv2859c | 205R>W | 0 | 26 | 1 | 41 | 0,7860 | -0,0238 | 0,0000 |
| Rv2861c | mapB | 94T>A | 0 | 26 | 1 | 41 | 0,7860 | -0,0238 | 0,0000 |
| Rv2864c | Rv2864c | 70A>V | 0 | 26 | 1 | 41 | 0,7860 | -0,0238 | 0,0000 |
| Rv2872 | Rv2872 | 35E>D | 0 | 26 | 1 | 41 | 0,7860 | -0,0238 | 0,0000 |
| Rv2873 | mpt83 | 40A>T | 0 | 26 | 1 | 41 | 0,7860 | -0,0238 | 0,0000 |
| Rv2883c | pyrH | 7A>T | 0 | 26 | 1 | 41 | 0,7860 | -0,0238 | 0,0000 |
| Rv2905 | lppW | 106V>I | 0 | 26 | 1 | 41 | 0,7860 | -0,0238 | 0,0000 |
| Rv2905 | lppW | 258Q>R | 0 | 26 | 1 | 41 | 0,7860 | -0,0238 | 0,0000 |
| Rv2908c | Rv2908c | 75V>M | 0 | 26 | 1 | 41 | 0,7860 | -0,0238 | 0,0000 |
| Rv2910c | Rv2910c | 146M>I | 0 | 26 | 1 | 41 | 0,7860 | -0,0238 | 0,0000 |
| Rv2915c | Rv2915c | 58G>E | 0 | 26 | 1 | 41 | 0,7860 | -0,0238 | 0,0000 |
| Rv2915c | Rv2915c | 85T>A | 0 | 26 | 1 | 41 | 0,7860 | -0,0238 | 0,0000 |
| Rv2916c | ffh | 437G>V | 0 | 26 | 1 | 41 | 0,7860 | -0,0238 | 0,0000 |
| Rv2918c | glnD | 279S>G | 0 | 26 | 1 | 41 | 0,7860 | -0,0238 | 0,0000 |
| Rv2918c | glnD | 589L>F | 0 | 26 | 1 | 41 | 0,7860 | -0,0238 | 0,0000 |
| Rv2921c | ftsY | 139R>S | 0 | 26 | 1 | 41 | 0,7860 | -0,0238 | 0,0000 |
| Rv2921c | ftsY | 308V>A | 0 | 26 | 1 | 41 | 0,7860 | -0,0238 | 0,0000 |
| Rv2926c | Rv2926c | 122T>I | 0 | 26 | 1 | 41 | 0,7860 | -0,0238 | 0,0000 |
| Rv2928 | tesA | 133S>P | 0 | 26 | 1 | 41 | 0,7860 | -0,0238 | 0,0000 |
| Rv2933 | ppsC | 369G>D | 0 | 26 | 1 | 41 | 0,7860 | -0,0238 | 0,0000 |
| Rv2934 | ppsD | 1401S>I | 0 | 26 | 1 | 41 | 0,7860 | -0,0238 | 0,0000 |
| Rv2935 | ppsE | 925V>F | 0 | 26 | 1 | 41 | 0,7860 | -0,0238 | 0,0000 |
| Rv2939 | papA5 | 85A>T | 0 | 26 | 1 | 41 | 0,7860 | -0,0238 | 0,0000 |
| Rv2940c | mas | 1130R>C | 0 | 26 | 1 | 41 | 0,7860 | -0,0238 | 0,0000 |
| Rv2940c | mas | 1322T>S | 0 | 26 | 1 | 41 | 0,7860 | -0,0238 | 0,0000 |
| Rv2940c | mas | 1820V>M | 0 | 26 | 1 | 41 | 0,7860 | -0,0238 | 0,0000 |

| Rv2940c | mas | 1854V>A | 0 | 26 | 1 | 41 | 0,7860 | -0,0238 | 0,0000 |
| --- | --- | --- | --- | --- | --- | --- | --- | --- | --- |
| Rv2940c | mas | 818L>F | 0 | 26 | 1 | 41 | 0,7860 | -0,0238 | 0,0000 |
| Rv2941 | fadD28 | 250R>C | 0 | 26 | 1 | 41 | 0,7860 | -0,0238 | 0,0000 |
| Rv2942 | mmpL7 | 614I>M | 0 | 26 | 1 | 41 | 0,7860 | -0,0238 | 0,0000 |
| Rv2945c | lppX | 26G>R | 0 | 26 | 1 | 41 | 0,7860 | -0,0238 | 0,0000 |
| Rv2947c | pks15 | 269A>T | 0 | 26 | 1 | 41 | 0,7860 | -0,0238 | 0,0000 |
| Rv2947c | pks15 | 475P>H | 0 | 26 | 1 | 41 | 0,7860 | -0,0238 | 0,0000 |
| Rv2951c | Rv2951c | 36D>A | 0 | 26 | 1 | 41 | 0,7860 | -0,0238 | 0,0000 |
| Rv2953 | Rv2953 | 82V>M | 0 | 26 | 1 | 41 | 0,7860 | -0,0238 | 0,0000 |
| Rv2954c | Rv2954c | 202W>C | 0 | 26 | 1 | 41 | 0,7860 | -0,0238 | 0,0000 |
| Rv2957 | Rv2957 | 195D>N | 0 | 26 | 1 | 41 | 0,7860 | -0,0238 | 0,0000 |
| Rv2978c | Rv2978c | 430T>I | 0 | 26 | 1 | 41 | 0,7860 | -0,0238 | 0,0000 |
| Rv2981c | ddl | 90P>R | 0 | 26 | 1 | 41 | 0,7860 | -0,0238 | 0,0000 |
| Rv2989 | Rv2989 | 35D>G | 0 | 26 | 1 | 41 | 0,7860 | -0,0238 | 0,0000 |
| Rv2991 | Rv2991 | 159G>D | 0 | 26 | 1 | 41 | 0,7860 | -0,0238 | 0,0000 |
| Rv2991 | Rv2991 | 62A>T | 0 | 26 | 1 | 41 | 0,7860 | -0,0238 | 0,0000 |
| Rv2992c | gltX | 247L>F | 0 | 26 | 1 | 41 | 0,7860 | -0,0238 | 0,0000 |
| Rv2992c | gltX | 277P>S | 0 | 26 | 1 | 41 | 0,7860 | -0,0238 | 0,0000 |
| Rv3001c | ilvC | 68P>T | 0 | 26 | 1 | 41 | 0,7860 | -0,0238 | 0,0000 |
| Rv3011c | gatA | 151R>Q | 0 | 26 | 1 | 41 | 0,7860 | -0,0238 | 0,0000 |
| Rv3012c | gatC | 7D>G | 0 | 26 | 1 | 41 | 0,7860 | -0,0238 | 0,0000 |
| Rv3016 | lpqA | 10L>S | 0 | 26 | 1 | 41 | 0,7860 | -0,0238 | 0,0000 |
| Rv3020c | esxS | 34Q>R | 0 | 26 | 1 | 41 | 0,7860 | -0,0238 | 0,0000 |
| Rv3021c | PPE47 | 180T>A | 0 | 26 | 1 | 41 | 0,7860 | -0,0238 | 0,0000 |
| Rv3022A | PE29 | 30G>R | 0 | 26 | 1 | 41 | 0,7860 | -0,0238 | 0,0000 |
| Rv3025c | iscS | 168V>F | 0 | 26 | 1 | 41 | 0,7860 | -0,0238 | 0,0000 |
| Rv3026c | Rv3026c | 252L>R | 0 | 26 | 1 | 41 | 0,7860 | -0,0238 | 0,0000 |
| Rv3028c | fixB | 181I>V | 0 | 26 | 1 | 41 | 0,7860 | -0,0238 | 0,0000 |
| Rv3030 | Rv3030 | 171T>I | 0 | 26 | 1 | 41 | 0,7860 | -0,0238 | 0,0000 |
| Rv3031 | Rv3031 | 424A>T | 0 | 26 | 1 | 41 | 0,7860 | -0,0238 | 0,0000 |
| Rv3032 | Rv3032 | 222V>M | 0 | 26 | 1 | 41 | 0,7860 | -0,0238 | 0,0000 |
| Rv3032 | Rv3032 | 352V>A | 0 | 26 | 1 | 41 | 0,7860 | -0,0238 | 0,0000 |
| Rv3035 | Rv3035 | 248P>L | 0 | 26 | 1 | 41 | 0,7860 | -0,0238 | 0,0000 |

| Rv3037c | Rv3037c | 327R>Q | 0 | 26 | 1 | 41 | 0,7860 | -0,0238 | 0,0000 |
| --- | --- | --- | --- | --- | --- | --- | --- | --- | --- |
| Rv3038c | Rv3038c | 278T>A | 0 | 26 | 1 | 41 | 0,7860 | -0,0238 | 0,0000 |
| Rv3040c | Rv3040c | 121S>P | 0 | 26 | 1 | 41 | 0,7860 | -0,0238 | 0,0000 |
| Rv3040c | Rv3040c | 214I>V | 0 | 26 | 1 | 41 | 0,7860 | -0,0238 | 0,0000 |
| Rv3042c | serB2 | 400G>E | 0 | 26 | 1 | 41 | 0,7860 | -0,0238 | 0,0000 |
| Rv3046c | Rv3046c | 36S>P | 0 | 26 | 1 | 41 | 0,7860 | -0,0238 | 0,0000 |
| Rv3051c | nrdE | 442D>N | 0 | 26 | 1 | 41 | 0,7860 | -0,0238 | 0,0000 |
| Rv3055 | Rv3055 | 143L>M | 0 | 26 | 1 | 41 | 0,7860 | -0,0238 | 0,0000 |
| Rv3059 | cyp136 | 189V>I | 0 | 26 | 1 | 41 | 0,7860 | -0,0238 | 0,0000 |
| Rv3071 | Rv3071 | 168T>A | 0 | 26 | 1 | 41 | 0,7860 | -0,0238 | 0,0000 |
| Rv3072c | Rv3072c | 43W>R | 0 | 26 | 1 | 41 | 0,7860 | -0,0238 | 0,0000 |
| Rv3074 | Rv3074 | 1M>T | 0 | 26 | 1 | 41 | 0,7860 | -0,0238 | 0,0000 |
| Rv3074 | Rv3074 | 350T>S | 0 | 26 | 1 | 41 | 0,7860 | -0,0238 | 0,0000 |
| Rv3075c | Rv3075c | 131I>L | 0 | 26 | 1 | 41 | 0,7860 | -0,0238 | 0,0000 |
| Rv3075c | Rv3075c | 4M>V | 0 | 26 | 1 | 41 | 0,7860 | -0,0238 | 0,0000 |
| Rv3077 | Rv3077 | 416V>I | 0 | 26 | 1 | 41 | 0,7860 | -0,0238 | 0,0000 |
| Rv3077 | Rv3077 | 590S>Y | 0 | 26 | 1 | 41 | 0,7860 | -0,0238 | 0,0000 |
| Rv3080c | pknK | 620A>P | 0 | 26 | 1 | 41 | 0,7860 | -0,0238 | 0,0000 |
| Rv3080c | pknK | 671V>A | 0 | 26 | 1 | 41 | 0,7860 | -0,0238 | 0,0000 |
| Rv3080c | pknK | 982L>V | 0 | 26 | 1 | 41 | 0,7860 | -0,0238 | 0,0000 |
| Rv3081 | Rv3081 | 318W>* | 0 | 26 | 1 | 41 | 0,7860 | -0,0238 | 0,0000 |
| Rv3081 | Rv3081 | 395A>T | 0 | 26 | 1 | 41 | 0,7860 | -0,0238 | 0,0000 |
| Rv3087 | Rv3087 | 270A>V | 0 | 26 | 1 | 41 | 0,7860 | -0,0238 | 0,0000 |
| Rv3087 | Rv3087 | 50R>H | 0 | 26 | 1 | 41 | 0,7860 | -0,0238 | 0,0000 |
| Rv3089 | fadD13 | 102S>T | 0 | 26 | 1 | 41 | 0,7860 | -0,0238 | 0,0000 |
| Rv3092c | Rv3092c | 195R>G | 0 | 26 | 1 | 41 | 0,7860 | -0,0238 | 0,0000 |
| Rv3093c | Rv3093c | 2T>I | 0 | 26 | 1 | 41 | 0,7860 | -0,0238 | 0,0000 |
| Rv3103c | Rv3103c | 109P>L | 0 | 26 | 1 | 41 | 0,7860 | -0,0238 | 0,0000 |
| Rv3105c | prfB | 187A>G | 0 | 26 | 1 | 41 | 0,7860 | -0,0238 | 0,0000 |
| Rv3109 | moaA1 | 339V>M | 0 | 26 | 1 | 41 | 0,7860 | -0,0238 | 0,0000 |
| Rv3116 | moeB2 | 240L>P | 0 | 26 | 1 | 41 | 0,7860 | -0,0238 | 0,0000 |
| Rv3120 | Rv3120 | 111G>R | 0 | 26 | 1 | 41 | 0,7860 | -0,0238 | 0,0000 |
| Rv3129 | Rv3129 | 44K>T | 0 | 26 | 1 | 41 | 0,7860 | -0,0238 | 0,0000 |

| Rv3136 | PPE51 | 222A>T | 0 | 26 | 1 | 41 | 0,7860 | -0,0238 | 0,0000 |
| --- | --- | --- | --- | --- | --- | --- | --- | --- | --- |
| Rv3138 | pflA | 77S>* | 0 | 26 | 1 | 41 | 0,7860 | -0,0238 | 0,0000 |
| Rv3139 | fadE24 | 15G>D | 0 | 26 | 1 | 41 | 0,7860 | -0,0238 | 0,0000 |
| Rv3139 | fadE24 | 49G>S | 0 | 26 | 1 | 41 | 0,7860 | -0,0238 | 0,0000 |
| Rv3140 | fadE23 | 123R>W | 0 | 26 | 1 | 41 | 0,7860 | -0,0238 | 0,0000 |
| Rv3140 | fadE23 | 339G>A | 0 | 26 | 1 | 41 | 0,7860 | -0,0238 | 0,0000 |
| Rv3143 | Rv3143 | 122R>H | 0 | 26 | 1 | 41 | 0,7860 | -0,0238 | 0,0000 |
| Rv3150 | nuoF | 335T>A | 0 | 26 | 1 | 41 | 0,7860 | -0,0238 | 0,0000 |
| Rv3159c | PPE53 | 484S>L | 0 | 26 | 1 | 41 | 0,7860 | -0,0238 | 0,0000 |
| Rv3167c | Rv3167c | 81L>V | 0 | 26 | 1 | 41 | 0,7860 | -0,0238 | 0,0000 |
| Rv3169 | Rv3169 | 56N>S | 0 | 26 | 1 | 41 | 0,7860 | -0,0238 | 0,0000 |
| Rv3178 | Rv3178 | 58N>K | 0 | 26 | 1 | 41 | 0,7860 | -0,0238 | 0,0000 |
| Rv3182 | Rv3182 | 58R>P | 0 | 26 | 1 | 41 | 0,7860 | -0,0238 | 0,0000 |
| Rv3191c | Rv3191c | 283N>D | 0 | 26 | 1 | 41 | 0,7860 | -0,0238 | 0,0000 |
| Rv3191c | Rv3191c | 81D>V | 0 | 26 | 1 | 41 | 0,7860 | -0,0238 | 0,0000 |
| Rv3195 | Rv3195 | 191G>D | 0 | 26 | 1 | 41 | 0,7860 | -0,0238 | 0,0000 |
| Rv3197 | Rv3197 | 416G>A | 0 | 26 | 1 | 41 | 0,7860 | -0,0238 | 0,0000 |
| Rv3197 | Rv3197 | 423D>N | 0 | 26 | 1 | 41 | 0,7860 | -0,0238 | 0,0000 |
| Rv3201c | Rv3201c | 134P>T | 0 | 26 | 1 | 41 | 0,7860 | -0,0238 | 0,0000 |
| Rv3202c | Rv3202c | 645G>D | 0 | 26 | 1 | 41 | 0,7860 | -0,0238 | 0,0000 |
| Rv3202c | Rv3202c | 952V>A | 0 | 26 | 1 | 41 | 0,7860 | -0,0238 | 0,0000 |
| Rv3212 | Rv3212 | 132V>I | 0 | 26 | 1 | 41 | 0,7860 | -0,0238 | 0,0000 |
| Rv3216 | Rv3216 | 60V>I | 0 | 26 | 1 | 41 | 0,7860 | -0,0238 | 0,0000 |
| Rv3221A | Rv3221A | 48R>L | 0 | 26 | 1 | 41 | 0,7860 | -0,0238 | 0,0000 |
| Rv3225c | Rv3225c | 201A>T | 0 | 26 | 1 | 41 | 0,7860 | -0,0238 | 0,0000 |
| Rv3226c | Rv3226c | 210L>M | 0 | 26 | 1 | 41 | 0,7860 | -0,0238 | 0,0000 |
| Rv3240c | secA1 | 366D>G | 0 | 26 | 1 | 41 | 0,7860 | -0,0238 | 0,0000 |
| Rv3243c | Rv3243c | 72T>A | 0 | 26 | 1 | 41 | 0,7860 | -0,0238 | 0,0000 |
| Rv3244c | lpqB | 570P>A | 0 | 26 | 1 | 41 | 0,7860 | -0,0238 | 0,0000 |
| Rv3247c | tmk | 185Q>E | 0 | 26 | 1 | 41 | 0,7860 | -0,0238 | 0,0000 |
| Rv3254 | Rv3254 | 149V>A | 0 | 26 | 1 | 41 | 0,7860 | -0,0238 | 0,0000 |
| Rv3256c | Rv3256c | 185S>P | 0 | 26 | 1 | 41 | 0,7860 | -0,0238 | 0,0000 |
| Rv3263 | Rv3263 | 119G>D | 0 | 26 | 1 | 41 | 0,7860 | -0,0238 | 0,0000 |

| Rv3266c | rmlD | 115A>T | 0 | 26 | 1 | 41 | 0,7860 | -0,0238 | 0,0000 |
| --- | --- | --- | --- | --- | --- | --- | --- | --- | --- |
| Rv3270 | ctpC | 344A>T | 0 | 26 | 1 | 41 | 0,7860 | -0,0238 | 0,0000 |
| Rv3273 | Rv3273 | 32R>P | 0 | 26 | 1 | 41 | 0,7860 | -0,0238 | 0,0000 |
| Rv3274c | fadE25 | 46R>W | 0 | 26 | 1 | 41 | 0,7860 | -0,0238 | 0,0000 |
| Rv3277 | Rv3277 | 200A>V | 0 | 26 | 1 | 41 | 0,7860 | -0,0238 | 0,0000 |
| Rv3277 | Rv3277 | 23G>R | 0 | 26 | 1 | 41 | 0,7860 | -0,0238 | 0,0000 |
| Rv3282 | maf | 82D>H | 0 | 26 | 1 | 41 | 0,7860 | -0,0238 | 0,0000 |
| Rv3292 | Rv3292 | 14A>T | 0 | 26 | 1 | 41 | 0,7860 | -0,0238 | 0,0000 |
| Rv3296 | lhr | 581D>E | 0 | 26 | 1 | 41 | 0,7860 | -0,0238 | 0,0000 |
| Rv3299c | atsB | 60R>W | 0 | 26 | 1 | 41 | 0,7860 | -0,0238 | 0,0000 |
| Rv3299c | atsB | 620T>A | 0 | 26 | 1 | 41 | 0,7860 | -0,0238 | 0,0000 |
| Rv3303c | lpdA | 108M>R | 0 | 26 | 1 | 41 | 0,7860 | -0,0238 | 0,0000 |
| Rv3305c | amiA1 | 85M>I | 0 | 26 | 1 | 41 | 0,7860 | -0,0238 | 0,0000 |
| Rv3314c | deoA | 24A>P | 0 | 26 | 1 | 41 | 0,7860 | -0,0238 | 0,0000 |
| Rv3314c | deoA | 414S>R | 0 | 26 | 1 | 41 | 0,7860 | -0,0238 | 0,0000 |
| Rv3324c | moaC | 165Y>D | 0 | 26 | 1 | 41 | 0,7860 | -0,0238 | 0,0000 |
| Rv3333c | Rv3333c | 273P>S | 0 | 26 | 1 | 41 | 0,7860 | -0,0238 | 0,0000 |
| Rv3339c | icd1 | 202Y>H | 0 | 26 | 1 | 41 | 0,7860 | -0,0238 | 0,0000 |
| Rv3343c | PPE54 | 1531A>V | 0 | 26 | 1 | 41 | 0,7860 | -0,0238 | 0,0000 |
| Rv3343c | PPE54 | 2107A>V | 0 | 26 | 1 | 41 | 0,7860 | -0,0238 | 0,0000 |
| Rv3343c | PPE54 | 2157I>T | 0 | 26 | 1 | 41 | 0,7860 | -0,0238 | 0,0000 |
| Rv3343c | PPE54 | 375E>Q | 0 | 26 | 1 | 41 | 0,7860 | -0,0238 | 0,0000 |
| Rv3345c | PE_PGRS49 | 15R>R | 0 | 26 | 1 | 41 | 0,7860 | -0,0238 | 0,0000 |
| Rv3345c | PE_PGRS49 | 54Q>R | 0 | 26 | 1 | 41 | 0,7860 | -0,0238 | 0,0000 |
| Rv3350c | PPE56 | 3129L>V | 0 | 26 | 1 | 41 | 0,7860 | -0,0238 | 0,0000 |
| Rv3350c | PPE56 | 3371T>A | 0 | 26 | 1 | 41 | 0,7860 | -0,0238 | 0,0000 |
| Rv3365c | Rv3365c | 331T>N | 0 | 26 | 1 | 41 | 0,7860 | -0,0238 | 0,0000 |
| Rv3365c | Rv3365c | 6R>C | 0 | 26 | 1 | 41 | 0,7860 | -0,0238 | 0,0000 |
| Rv3367 | PE_PGRS51 | 489G>R | 0 | 26 | 1 | 41 | 0,7860 | -0,0238 | 0,0000 |
| Rv3370c | dnaE2 | 59A>V | 0 | 26 | 1 | 41 | 0,7860 | -0,0238 | 0,0000 |
| Rv3371 | Rv3371 | 203A>V | 0 | 26 | 1 | 41 | 0,7860 | -0,0238 | 0,0000 |
| Rv3372 | otsB2 | 40T>I | 0 | 26 | 1 | 41 | 0,7860 | -0,0238 | 0,0000 |
| Rv3372 | otsB2 | 5G>D | 0 | 26 | 1 | 41 | 0,7860 | -0,0238 | 0,0000 |

| Rv3378c | Rv3378c | 272N>D | 0 | 26 | 1 | 41 | 0,7860 | -0,0238 | 0,0000 |
| --- | --- | --- | --- | --- | --- | --- | --- | --- | --- |
| Rv3379c | dxs2 | 305L>S | 0 | 26 | 1 | 41 | 0,7860 | -0,0238 | 0,0000 |
| Rv3379c | dxs2 | 348P>S | 0 | 26 | 1 | 41 | 0,7860 | -0,0238 | 0,0000 |
| Rv3382c | lytB1 | 5F>L | 0 | 26 | 1 | 41 | 0,7860 | -0,0238 | 0,0000 |
| Rv3383c | idsB | 40E>G | 0 | 26 | 1 | 41 | 0,7860 | -0,0238 | 0,0000 |
| Rv3388 | PE_PGRS52 | 234G>D | 0 | 26 | 1 | 41 | 0,7860 | -0,0238 | 0,0000 |
| Rv3388 | PE_PGRS52 | 351G>A | 0 | 26 | 1 | 41 | 0,7860 | -0,0238 | 0,0000 |
| Rv3391 | acrA1 | 403A>P | 0 | 26 | 1 | 41 | 0,7860 | -0,0238 | 0,0000 |
| Rv3391 | acrA1 | 56V>A | 0 | 26 | 1 | 41 | 0,7860 | -0,0238 | 0,0000 |
| Rv3395c | Rv3395c | 15A>V | 0 | 26 | 1 | 41 | 0,7860 | -0,0238 | 0,0000 |
| Rv3401 | Rv3401 | 192D>A | 0 | 26 | 1 | 41 | 0,7860 | -0,0238 | 0,0000 |
| Rv3401 | Rv3401 | 346P>S | 0 | 26 | 1 | 41 | 0,7860 | -0,0238 | 0,0000 |
| Rv3406 | Rv3406 | 172R>P | 0 | 26 | 1 | 41 | 0,7860 | -0,0238 | 0,0000 |
| Rv3409c | choD | 544P>L | 0 | 26 | 1 | 41 | 0,7860 | -0,0238 | 0,0000 |
| Rv3413c | Rv3413c | 228T>P | 0 | 26 | 1 | 41 | 0,7860 | -0,0238 | 0,0000 |
| Rv3415c | Rv3415c | 13V>E | 0 | 26 | 1 | 41 | 0,7860 | -0,0238 | 0,0000 |
| Rv3417c | groEL | 511A>V | 0 | 26 | 1 | 41 | 0,7860 | -0,0238 | 0,0000 |
| Rv3421c | Rv3421c | 164A>T | 0 | 26 | 1 | 41 | 0,7860 | -0,0238 | 0,0000 |
| Rv3421c | Rv3421c | 17P>Q | 0 | 26 | 1 | 41 | 0,7860 | -0,0238 | 0,0000 |
| Rv3423c | alr | 270G>E | 0 | 26 | 1 | 41 | 0,7860 | -0,0238 | 0,0000 |
| Rv3423c | alr | 325R>P | 0 | 26 | 1 | 41 | 0,7860 | -0,0238 | 0,0000 |
| Rv3424c | Rv3424c | 113P>L | 0 | 26 | 1 | 41 | 0,7860 | -0,0238 | 0,0000 |
| Rv3424c | Rv3424c | 118P>A | 0 | 26 | 1 | 41 | 0,7860 | -0,0238 | 0,0000 |
| Rv3424c | Rv3424c | 90H>R | 0 | 26 | 1 | 41 | 0,7860 | -0,0238 | 0,0000 |
| Rv3426 | PPE58 | 143G>D | 0 | 26 | 1 | 41 | 0,7860 | -0,0238 | 0,0000 |
| Rv3430c | PPE59 | 177A>A | 0 | 26 | 1 | 41 | 0,7860 | -0,0238 | 0,0000 |
| Rv3432c | gadB | 266P>L | 0 | 26 | 1 | 41 | 0,7860 | -0,0238 | 0,0000 |
| Rv3435c | Rv3435c | 252T>A | 0 | 26 | 1 | 41 | 0,7860 | -0,0238 | 0,0000 |
| Rv3436c | glmS | 465P>Q | 0 | 26 | 1 | 41 | 0,7860 | -0,0238 | 0,0000 |
| Rv3442c | rpsI | 10T>I | 0 | 26 | 1 | 41 | 0,7860 | -0,0238 | 0,0000 |
| Rv3447c | Rv3447c | 1046R>W | 0 | 26 | 1 | 41 | 0,7860 | -0,0238 | 0,0000 |
| Rv3447c | Rv3447c | 1162L>V | 0 | 26 | 1 | 41 | 0,7860 | -0,0238 | 0,0000 |
| Rv3447c | Rv3447c | 701G>R | 0 | 26 | 1 | 41 | 0,7860 | -0,0238 | 0,0000 |

| Rv3447c | Rv3447c | 967D>G | 0 | 26 | 1 | 41 | 0,7860 | -0,0238 | 0,0000 |
| --- | --- | --- | --- | --- | --- | --- | --- | --- | --- |
| Rv3448 | Rv3448 | 280A>T | 0 | 26 | 1 | 41 | 0,7860 | -0,0238 | 0,0000 |
| Rv3450c | Rv3450c | 267G>S | 0 | 26 | 1 | 41 | 0,7860 | -0,0238 | 0,0000 |
| Rv3455c | truA | 53A>V | 0 | 26 | 1 | 41 | 0,7860 | -0,0238 | 0,0000 |
| Rv3472 | Rv3472 | 82G>R | 0 | 26 | 1 | 41 | 0,7860 | -0,0238 | 0,0000 |
| Rv3478 | PPE60 | 215A>T | 0 | 26 | 1 | 41 | 0,7860 | -0,0238 | 0,0000 |
| Rv3478 | PPE60 | 218V>T | 0 | 26 | 1 | 41 | 0,7860 | -0,0238 | 0,0000 |
| Rv3481c | Rv3481c | 110T>A | 0 | 26 | 1 | 41 | 0,7860 | -0,0238 | 0,0000 |
| Rv3482c | Rv3482c | 208T>A | 0 | 26 | 1 | 41 | 0,7860 | -0,0238 | 0,0000 |
| Rv3504 | fadE26 | 378G>D | 0 | 26 | 1 | 41 | 0,7860 | -0,0238 | 0,0000 |
| Rv3505 | fadE27 | 113S>* | 0 | 26 | 1 | 41 | 0,7860 | -0,0238 | 0,0000 |
| Rv3507 | PE_PGRS53 | 569T>N | 0 | 26 | 1 | 41 | 0,7860 | -0,0238 | 0,0000 |
| Rv3508 | PE_PGRS54 | 1031S>N | 0 | 26 | 1 | 41 | 0,7860 | -0,0238 | 0,0000 |
| Rv3508 | PE_PGRS54 | 1046N>T | 0 | 26 | 1 | 41 | 0,7860 | -0,0238 | 0,0000 |
| Rv3508 | PE_PGRS54 | 1200G>D | 0 | 26 | 1 | 41 | 0,7860 | -0,0238 | 0,0000 |
| Rv3508 | PE_PGRS54 | 1704G>A | 0 | 26 | 1 | 41 | 0,7860 | -0,0238 | 0,0000 |
| Rv3508 | PE_PGRS54 | 350T>P | 0 | 26 | 1 | 41 | 0,7860 | -0,0238 | 0,0000 |
| Rv3508 | PE_PGRS54 | 830S>N | 0 | 26 | 1 | 41 | 0,7860 | -0,0238 | 0,0000 |
| Rv3508 | PE_PGRS54 | 845N>T | 0 | 26 | 1 | 41 | 0,7860 | -0,0238 | 0,0000 |
| Rv3510c | Rv3510c | 250G>R | 0 | 26 | 1 | 41 | 0,7860 | -0,0238 | 0,0000 |
| Rv3512 | PE_PGRS56 | 913G>A | 0 | 26 | 1 | 41 | 0,7860 | -0,0238 | 0,0000 |
| Rv3512 | PE_PGRS56 | 914D>A | 0 | 26 | 1 | 41 | 0,7860 | -0,0238 | 0,0000 |
| Rv3512 | PE_PGRS56 | 917A>G | 0 | 26 | 1 | 41 | 0,7860 | -0,0238 | 0,0000 |
| Rv3525c | Rv3525c | 152W>R | 0 | 26 | 1 | 41 | 0,7860 | -0,0238 | 0,0000 |
| Rv3533c | PPE62 | 239G>S | 0 | 26 | 1 | 41 | 0,7860 | -0,0238 | 0,0000 |
| Rv3535c | Rv3535c | 265E>V | 0 | 26 | 1 | 41 | 0,7860 | -0,0238 | 0,0000 |
| Rv3537 | Rv3537 | 554L>V | 0 | 26 | 1 | 41 | 0,7860 | -0,0238 | 0,0000 |
| Rv3538 | Rv3538 | 122I>V | 0 | 26 | 1 | 41 | 0,7860 | -0,0238 | 0,0000 |
| Rv3540c | ltp2 | 249P>S | 0 | 26 | 1 | 41 | 0,7860 | -0,0238 | 0,0000 |
| Rv3550 | echA20 | 166T>I | 0 | 26 | 1 | 41 | 0,7860 | -0,0238 | 0,0000 |
| Rv3551 | Rv3551 | 149G>V | 0 | 26 | 1 | 41 | 0,7860 | -0,0238 | 0,0000 |
| Rv3554 | fdxB | 614G>D | 0 | 26 | 1 | 41 | 0,7860 | -0,0238 | 0,0000 |
| Rv3554 | fdxB | 66S>T | 0 | 26 | 1 | 41 | 0,7860 | -0,0238 | 0,0000 |

| Rv3555c | Rv3555c | 64G>D | 0 | 26 | 1 | 41 | 0,7860 | -0,0238 | 0,0000 |
| --- | --- | --- | --- | --- | --- | --- | --- | --- | --- |
| Rv3556c | fadA6 | 288P>T | 0 | 26 | 1 | 41 | 0,7860 | -0,0238 | 0,0000 |
| Rv3560c | fadE30 | 172L>F | 0 | 26 | 1 | 41 | 0,7860 | -0,0238 | 0,0000 |
| Rv3560c | fadE30 | 230P>A | 0 | 26 | 1 | 41 | 0,7860 | -0,0238 | 0,0000 |
| Rv3562 | fadE31 | 204L>P | 0 | 26 | 1 | 41 | 0,7860 | -0,0238 | 0,0000 |
| Rv3563 | fadE32 | 181V>I | 0 | 26 | 1 | 41 | 0,7860 | -0,0238 | 0,0000 |
| Rv3570c | Rv3570c | 285E>A | 0 | 26 | 1 | 41 | 0,7860 | -0,0238 | 0,0000 |
| Rv3576 | lppH | 191G>S | 0 | 26 | 1 | 41 | 0,7860 | -0,0238 | 0,0000 |
| Rv3577 | Rv3577 | 177R>C | 0 | 26 | 1 | 41 | 0,7860 | -0,0238 | 0,0000 |
| Rv3589 | mutY | 286D>G | 0 | 26 | 1 | 41 | 0,7860 | -0,0238 | 0,0000 |
| Rv3596c | clpC1 | 244D>Y | 0 | 26 | 1 | 41 | 0,7860 | -0,0238 | 0,0000 |
| Rv3598c | lysS | 455M>R | 0 | 26 | 1 | 41 | 0,7860 | -0,0238 | 0,0000 |
| Rv3604c | Rv3604c | 343P>L | 0 | 26 | 1 | 41 | 0,7860 | -0,0238 | 0,0000 |
| Rv3608c | folP1 | 169V>I | 0 | 26 | 1 | 41 | 0,7860 | -0,0238 | 0,0000 |
| Rv3608c | folP1 | 201A>V | 0 | 26 | 1 | 41 | 0,7860 | -0,0238 | 0,0000 |
| Rv3621c | PPE65 | 274V>A | 0 | 26 | 1 | 41 | 0,7860 | -0,0238 | 0,0000 |
| Rv3623 | lpqG | 216G>D | 0 | 26 | 1 | 41 | 0,7860 | -0,0238 | 0,0000 |
| Rv3625c | mesJ | 128T>A | 0 | 26 | 1 | 41 | 0,7860 | -0,0238 | 0,0000 |
| Rv3625c | mesJ | 232A>V | 0 | 26 | 1 | 41 | 0,7860 | -0,0238 | 0,0000 |
| Rv3626c | Rv3626c | 212H>R | 0 | 26 | 1 | 41 | 0,7860 | -0,0238 | 0,0000 |
| Rv3636 | Rv3636 | 84W>C | 0 | 26 | 1 | 41 | 0,7860 | -0,0238 | 0,0000 |
| Rv3639c | Rv3639c | 105A>V | 0 | 26 | 1 | 41 | 0,7860 | -0,0238 | 0,0000 |
| Rv3639c | Rv3639c | 132A>E | 0 | 26 | 1 | 41 | 0,7860 | -0,0238 | 0,0000 |
| Rv3640c | Rv3640c | 270K>N | 0 | 26 | 1 | 41 | 0,7860 | -0,0238 | 0,0000 |
| Rv3645 | Rv3645 | 173A>G | 0 | 26 | 1 | 41 | 0,7860 | -0,0238 | 0,0000 |
| Rv3645 | Rv3645 | 381A>T | 0 | 26 | 1 | 41 | 0,7860 | -0,0238 | 0,0000 |
| Rv3647c | Rv3647c | 133D>N | 0 | 26 | 1 | 41 | 0,7860 | -0,0238 | 0,0000 |
| Rv3648c | cspA | 49E>G | 0 | 26 | 1 | 41 | 0,7860 | -0,0238 | 0,0000 |
| Rv3651 | Rv3651 | 251Q>P | 0 | 26 | 1 | 41 | 0,7860 | -0,0238 | 0,0000 |
| Rv3652 | PE_PGRS60 | 8P>S | 0 | 26 | 1 | 41 | 0,7860 | -0,0238 | 0,0000 |
| Rv3653 | PE_PGRS61 | 190G>D | 0 | 26 | 1 | 41 | 0,7860 | -0,0238 | 0,0000 |
| Rv3658c | Rv3658c | 62T>A | 0 | 26 | 1 | 41 | 0,7860 | -0,0238 | 0,0000 |
| Rv3659c | Rv3659c | 301A>V | 0 | 26 | 1 | 41 | 0,7860 | -0,0238 | 0,0000 |

| Rv3659c | Rv3659c | 320E>K | 0 | 26 | 1 | 41 | 0,7860 | -0,0238 | 0,0000 |
| --- | --- | --- | --- | --- | --- | --- | --- | --- | --- |
| Rv3660c | Rv3660c | 282P>S | 0 | 26 | 1 | 41 | 0,7860 | -0,0238 | 0,0000 |
| Rv3660c | Rv3660c | 58P>S | 0 | 26 | 1 | 41 | 0,7860 | -0,0238 | 0,0000 |
| Rv3661 | Rv3661 | 260A>T | 0 | 26 | 1 | 41 | 0,7860 | -0,0238 | 0,0000 |
| Rv3663c | dppD | 538A>G | 0 | 26 | 1 | 41 | 0,7860 | -0,0238 | 0,0000 |
| Rv3666c | dppA | 287A>V | 0 | 26 | 1 | 41 | 0,7860 | -0,0238 | 0,0000 |
| Rv3668c | Rv3668c | 20V>G | 0 | 26 | 1 | 41 | 0,7860 | -0,0238 | 0,0000 |
| Rv3669 | Rv3669 | 10V>A | 0 | 26 | 1 | 41 | 0,7860 | -0,0238 | 0,0000 |
| Rv3684 | Rv3684 | 200V>A | 0 | 26 | 1 | 41 | 0,7860 | -0,0238 | 0,0000 |
| Rv3684 | Rv3684 | 21I>T | 0 | 26 | 1 | 41 | 0,7860 | -0,0238 | 0,0000 |
| Rv3687c | rsfB | 17V>I | 0 | 26 | 1 | 41 | 0,7860 | -0,0238 | 0,0000 |
| Rv3695 | Rv3695 | 124P>L | 0 | 26 | 1 | 41 | 0,7860 | -0,0238 | 0,0000 |
| Rv3697c | Rv3697c | 141D>Y | 0 | 26 | 1 | 41 | 0,7860 | -0,0238 | 0,0000 |
| Rv3705c | Rv3705c | 59R>S | 0 | 26 | 1 | 41 | 0,7860 | -0,0238 | 0,0000 |
| Rv3706c | Rv3706c | 102A>P | 0 | 26 | 1 | 41 | 0,7860 | -0,0238 | 0,0000 |
| Rv3710 | leuA | 385K>N | 0 | 26 | 1 | 41 | 0,7860 | -0,0238 | 0,0000 |
| Rv3710 | leuA | 9A>S | 0 | 26 | 1 | 41 | 0,7860 | -0,0238 | 0,0000 |
| Rv3714c | Rv3714c | 76G>V | 0 | 26 | 1 | 41 | 0,7860 | -0,0238 | 0,0000 |
| Rv3716c | Rv3716c | 96L>S | 0 | 26 | 1 | 41 | 0,7860 | -0,0238 | 0,0000 |
| Rv3720 | Rv3720 | 289K>R | 0 | 26 | 1 | 41 | 0,7860 | -0,0238 | 0,0000 |
| Rv3720 | Rv3720 | 364G>S | 0 | 26 | 1 | 41 | 0,7860 | -0,0238 | 0,0000 |
| Rv3728 | Rv3728 | 900E>A | 0 | 26 | 1 | 41 | 0,7860 | -0,0238 | 0,0000 |
| Rv3731 | ligC | 64A>T | 0 | 26 | 1 | 41 | 0,7860 | -0,0238 | 0,0000 |
| Rv3736 | Rv3736 | 183R>W | 0 | 26 | 1 | 41 | 0,7860 | -0,0238 | 0,0000 |
| Rv3737 | Rv3737 | 11L>S | 0 | 26 | 1 | 41 | 0,7860 | -0,0238 | 0,0000 |
| Rv3737 | Rv3737 | 145A>T | 0 | 26 | 1 | 41 | 0,7860 | -0,0238 | 0,0000 |
| Rv3741c | Rv3741c | 96N>D | 0 | 26 | 1 | 41 | 0,7860 | -0,0238 | 0,0000 |
| Rv3743c | ctpJ | 631H>R | 0 | 26 | 1 | 41 | 0,7860 | -0,0238 | 0,0000 |
| Rv3744 | Rv3744 | 6E>K | 0 | 26 | 1 | 41 | 0,7860 | -0,0238 | 0,0000 |
| Rv3749c | Rv3749c | 106L>S | 0 | 26 | 1 | 41 | 0,7860 | -0,0238 | 0,0000 |
| Rv3749c | Rv3749c | 139G>S | 0 | 26 | 1 | 41 | 0,7860 | -0,0238 | 0,0000 |
| Rv3754 | tyrA | 77M>I | 0 | 26 | 1 | 41 | 0,7860 | -0,0238 | 0,0000 |
| Rv3755c | Rv3755c | 44R>P | 0 | 26 | 1 | 41 | 0,7860 | -0,0238 | 0,0000 |

| Rv3759c | proX | 52E>G | 0 | 26 | 1 | 41 | 0,7860 | -0,0238 | 0,0000 |
| --- | --- | --- | --- | --- | --- | --- | --- | --- | --- |
| Rv3762c | Rv3762c | 512F>V | 0 | 26 | 1 | 41 | 0,7860 | -0,0238 | 0,0000 |
| Rv3762c | Rv3762c | 582K>E | 0 | 26 | 1 | 41 | 0,7860 | -0,0238 | 0,0000 |
| Rv3763 | lpqH | 37A>V | 0 | 26 | 1 | 41 | 0,7860 | -0,0238 | 0,0000 |
| Rv3764c | Rv3764c | 135V>A | 0 | 26 | 1 | 41 | 0,7860 | -0,0238 | 0,0000 |
| Rv3780 | Rv3780 | 11T>N | 0 | 26 | 1 | 41 | 0,7860 | -0,0238 | 0,0000 |
| Rv3784 | Rv3784 | 219V>F | 0 | 26 | 1 | 41 | 0,7860 | -0,0238 | 0,0000 |
| Rv3784 | Rv3784 | 287S>N | 0 | 26 | 1 | 41 | 0,7860 | -0,0238 | 0,0000 |
| Rv3784 | Rv3784 | 41M>I | 0 | 26 | 1 | 41 | 0,7860 | -0,0238 | 0,0000 |
| Rv3785 | Rv3785 | 276L>R | 0 | 26 | 1 | 41 | 0,7860 | -0,0238 | 0,0000 |
| Rv3786c | Rv3786c | 213N>Y | 0 | 26 | 1 | 41 | 0,7860 | -0,0238 | 0,0000 |
| Rv3787c | Rv3787c | 107I>S | 0 | 26 | 1 | 41 | 0,7860 | -0,0238 | 0,0000 |
| Rv3787c | Rv3787c | 307L>P | 0 | 26 | 1 | 41 | 0,7860 | -0,0238 | 0,0000 |
| Rv3791 | Rv3791 | 97G>R | 0 | 26 | 1 | 41 | 0,7860 | -0,0238 | 0,0000 |
| Rv3792 | Rv3792 | 18T>A | 0 | 26 | 1 | 41 | 0,7860 | -0,0238 | 0,0000 |
| Rv3794 | embA | 479V>M | 0 | 26 | 1 | 41 | 0,7860 | -0,0238 | 0,0000 |
| Rv3794 | embA | 54N>D | 0 | 26 | 1 | 41 | 0,7860 | -0,0238 | 0,0000 |
| Rv3794 | embA | 951E>D | 0 | 26 | 1 | 41 | 0,7860 | -0,0238 | 0,0000 |
| Rv3797 | fadE35 | 322L>F | 0 | 26 | 1 | 41 | 0,7860 | -0,0238 | 0,0000 |
| Rv3799c | accD4 | 288D>E | 0 | 26 | 1 | 41 | 0,7860 | -0,0238 | 0,0000 |
| Rv3799c | accD4 | 459A>T | 0 | 26 | 1 | 41 | 0,7860 | -0,0238 | 0,0000 |
| Rv3800c | pks13 | 135P>L | 0 | 26 | 1 | 41 | 0,7860 | -0,0238 | 0,0000 |
| Rv3800c | pks13 | 1545G>S | 0 | 26 | 1 | 41 | 0,7860 | -0,0238 | 0,0000 |
| Rv3801c | fadD32 | 457I>S | 0 | 26 | 1 | 41 | 0,7860 | -0,0238 | 0,0000 |
| Rv3802c | Rv3802c | 46V>L | 0 | 26 | 1 | 41 | 0,7860 | -0,0238 | 0,0000 |
| Rv3805c | Rv3805c | 172L>F | 0 | 26 | 1 | 41 | 0,7860 | -0,0238 | 0,0000 |
| Rv3806c | Rv3806c | 35A>S | 0 | 26 | 1 | 41 | 0,7860 | -0,0238 | 0,0000 |
| Rv3808c | glfT | 497P>L | 0 | 26 | 1 | 41 | 0,7860 | -0,0238 | 0,0000 |
| Rv3811 | Rv3811 | 102L>P | 0 | 26 | 1 | 41 | 0,7860 | -0,0238 | 0,0000 |
| Rv3812 | PE_PGRS62 | 471P>S | 0 | 26 | 1 | 41 | 0,7860 | -0,0238 | 0,0000 |
| Rv3813c | Rv3813c | 269E>A | 0 | 26 | 1 | 41 | 0,7860 | -0,0238 | 0,0000 |
| Rv3815c | Rv3815c | 160H>Y | 0 | 26 | 1 | 41 | 0,7860 | -0,0238 | 0,0000 |
| Rv3819 | Rv3819 | 30K>E | 0 | 26 | 1 | 41 | 0,7860 | -0,0238 | 0,0000 |

| Rv3820c | papA2 | 330T>I | 0 | 26 | 1 | 41 | 0,7860 | -0,0238 | 0,0000 |
| --- | --- | --- | --- | --- | --- | --- | --- | --- | --- |
| Rv3821 | Rv3821 | 66F>L | 0 | 26 | 1 | 41 | 0,7860 | -0,0238 | 0,0000 |
| Rv3823c | mmpL8 | 564D>E | 0 | 26 | 1 | 41 | 0,7860 | -0,0238 | 0,0000 |
| Rv3824c | papA1 | 122E>D | 0 | 26 | 1 | 41 | 0,7860 | -0,0238 | 0,0000 |
| Rv3825c | pks2 | 1218P>A | 0 | 26 | 1 | 41 | 0,7860 | -0,0238 | 0,0000 |
| Rv3825c | pks2 | 1907A>S | 0 | 26 | 1 | 41 | 0,7860 | -0,0238 | 0,0000 |
| Rv3825c | pks2 | 228E>G | 0 | 26 | 1 | 41 | 0,7860 | -0,0238 | 0,0000 |
| Rv3826 | fadD23 | 47R>G | 0 | 26 | 1 | 41 | 0,7860 | -0,0238 | 0,0000 |
| Rv3829c | Rv3829c | 319D>E | 0 | 26 | 1 | 41 | 0,7860 | -0,0238 | 0,0000 |
| Rv3830c | Rv3830c | 42A>G | 0 | 26 | 1 | 41 | 0,7860 | -0,0238 | 0,0000 |
| Rv3834c | serS | 144G>R | 0 | 26 | 1 | 41 | 0,7860 | -0,0238 | 0,0000 |
| Rv3842c | glpQ1 | 19G>A | 0 | 26 | 1 | 41 | 0,7860 | -0,0238 | 0,0000 |
| Rv3842c | glpQ1 | 45C>S | 0 | 26 | 1 | 41 | 0,7860 | -0,0238 | 0,0000 |
| Rv3847 | Rv3847 | 22G>R | 0 | 26 | 1 | 41 | 0,7860 | -0,0238 | 0,0000 |
| Rv3854c | ethA | 341A>V | 0 | 26 | 1 | 41 | 0,7860 | -0,0238 | 0,0000 |
| Rv3854c | ethA | 378P>L | 0 | 26 | 1 | 41 | 0,7860 | -0,0238 | 0,0000 |
| Rv3854c | ethA | 423G>R | 0 | 26 | 1 | 41 | 0,7860 | -0,0238 | 0,0000 |
| Rv3854c | ethA | 453T>I | 0 | 26 | 1 | 41 | 0,7860 | -0,0238 | 0,0000 |
| Rv3857c | Rv3857c | 37A>E | 0 | 26 | 1 | 41 | 0,7860 | -0,0238 | 0,0000 |
| Rv3858c | gltD | 278D>N | 0 | 26 | 1 | 41 | 0,7860 | -0,0238 | 0,0000 |
| Rv3859c | gltB | 4K>T | 0 | 26 | 1 | 41 | 0,7860 | -0,0238 | 0,0000 |
| Rv3859c | gltB | 567R>S | 0 | 26 | 1 | 41 | 0,7860 | -0,0238 | 0,0000 |
| Rv3862c | whiB6 | 16W>C | 0 | 26 | 1 | 41 | 0,7860 | -0,0238 | 0,0000 |
| Rv3863 | Rv3863 | 304R>H | 0 | 26 | 1 | 41 | 0,7860 | -0,0238 | 0,0000 |
| Rv3863 | Rv3863 | 5R>P | 0 | 26 | 1 | 41 | 0,7860 | -0,0238 | 0,0000 |
| Rv3863 | Rv3863 | 74H>N | 0 | 26 | 1 | 41 | 0,7860 | -0,0238 | 0,0000 |
| Rv3865 | Rv3865 | 16A>G | 0 | 26 | 1 | 41 | 0,7860 | -0,0238 | 0,0000 |
| Rv3867 | Rv3867 | 35D>Y | 0 | 26 | 1 | 41 | 0,7860 | -0,0238 | 0,0000 |
| Rv3870 | Rv3870 | 104E>Q | 0 | 26 | 1 | 41 | 0,7860 | -0,0238 | 0,0000 |
| Rv3870 | Rv3870 | 345G>S | 0 | 26 | 1 | 41 | 0,7860 | -0,0238 | 0,0000 |
| Rv3870 | Rv3870 | 645K>N | 0 | 26 | 1 | 41 | 0,7860 | -0,0238 | 0,0000 |
| Rv3870 | Rv3870 | 690M>T | 0 | 26 | 1 | 41 | 0,7860 | -0,0238 | 0,0000 |
| Rv3876 | Rv3876 | 111P>R | 0 | 26 | 1 | 41 | 0,7860 | -0,0238 | 0,0000 |

| Rv3876 | Rv3876 | 15M>T | 0 | 26 | 1 | 41 | 0,7860 | -0,0238 | 0,0000 |
| --- | --- | --- | --- | --- | --- | --- | --- | --- | --- |
| Rv3876 | Rv3876 | 616V>I | 0 | 26 | 1 | 41 | 0,7860 | -0,0238 | 0,0000 |
| Rv3877 | Rv3877 | 224R>C | 0 | 26 | 1 | 41 | 0,7860 | -0,0238 | 0,0000 |
| Rv3877 | Rv3877 | 255R>G | 0 | 26 | 1 | 41 | 0,7860 | -0,0238 | 0,0000 |
| Rv3881c | Rv3881c | 125A>T | 0 | 26 | 1 | 41 | 0,7860 | -0,0238 | 0,0000 |
| Rv3885c | Rv3885c | 52Q>P | 0 | 26 | 1 | 41 | 0,7860 | -0,0238 | 0,0000 |
| Rv3886c | mycP2 | 219Q>E | 0 | 26 | 1 | 41 | 0,7860 | -0,0238 | 0,0000 |
| Rv3886c | mycP2 | 400A>T | 0 | 26 | 1 | 41 | 0,7860 | -0,0238 | 0,0000 |
| Rv3887c | Rv3887c | 190W>* | 0 | 26 | 1 | 41 | 0,7860 | -0,0238 | 0,0000 |
| Rv3887c | Rv3887c | 40G>R | 0 | 26 | 1 | 41 | 0,7860 | -0,0238 | 0,0000 |
| Rv3887c | Rv3887c | 421L>F | 0 | 26 | 1 | 41 | 0,7860 | -0,0238 | 0,0000 |
| Rv3888c | Rv3888c | 145D>H | 0 | 26 | 1 | 41 | 0,7860 | -0,0238 | 0,0000 |
| Rv3888c | Rv3888c | 93T>K | 0 | 26 | 1 | 41 | 0,7860 | -0,0238 | 0,0000 |
| Rv3894c | Rv3894c | 1277G>R | 0 | 26 | 1 | 41 | 0,7860 | -0,0238 | 0,0000 |
| Rv3894c | Rv3894c | 467S>T | 0 | 26 | 1 | 41 | 0,7860 | -0,0238 | 0,0000 |
| Rv3898c | Rv3898c | 71S>F | 0 | 26 | 1 | 41 | 0,7860 | -0,0238 | 0,0000 |
| Rv3903c | Rv3903c | 326L>V | 0 | 26 | 1 | 41 | 0,7860 | -0,0238 | 0,0000 |
| Rv3905c | esxF | 104*>W | 0 | 26 | 1 | 41 | 0,7860 | -0,0238 | 0,0000 |
| Rv3906c | Rv3906c | 14I>M | 0 | 26 | 1 | 41 | 0,7860 | -0,0238 | 0,0000 |
| Rv3906c | Rv3906c | 86R>G | 0 | 26 | 1 | 41 | 0,7860 | -0,0238 | 0,0000 |
| Rv3909 | Rv3909 | 384G>D | 0 | 26 | 1 | 41 | 0,7860 | -0,0238 | 0,0000 |
| Rv3910 | Rv3910 | 211A>T | 0 | 26 | 1 | 41 | 0,7860 | -0,0238 | 0,0000 |
| Rv3910 | Rv3910 | 612A>V | 0 | 26 | 1 | 41 | 0,7860 | -0,0238 | 0,0000 |
| Rv3910 | Rv3910 | 895T>I | 0 | 26 | 1 | 41 | 0,7860 | -0,0238 | 0,0000 |
| Rv3910 | Rv3910 | 957P>Q | 0 | 26 | 1 | 41 | 0,7860 | -0,0238 | 0,0000 |
| Rv3913 | trxB2 | 247A>T | 0 | 26 | 1 | 41 | 0,7860 | -0,0238 | 0,0000 |
| Rv3914 | trxC | 76Q>E | 0 | 26 | 1 | 41 | 0,7860 | -0,0238 | 0,0000 |
| Rv3915 | Rv3915 | 147A>T | 0 | 26 | 1 | 41 | 0,7860 | -0,0238 | 0,0000 |
| Rv3915 | Rv3915 | 210G>S | 0 | 26 | 1 | 41 | 0,7860 | -0,0238 | 0,0000 |
| Rv3915 | Rv3915 | 402R>S | 0 | 26 | 1 | 41 | 0,7860 | -0,0238 | 0,0000 |
| Rv3916c | Rv3916c | 34G>C | 0 | 26 | 1 | 41 | 0,7860 | -0,0238 | 0,0000 |
| Rv3917c | parB | 136A>T | 0 | 26 | 1 | 41 | 0,7860 | -0,0238 | 0,0000 |
| Rv3919c | gidB | 108L>R | 0 | 26 | 1 | 41 | 0,7860 | -0,0238 | 0,0000 |

| Rv3919c | gidB | 135V>A | 0 | 26 | 1 | 41 | 0,7860 | -0,0238 | 0,0000 |
| --- | --- | --- | --- | --- | --- | --- | --- | --- | --- |
| Rv3919c | gidB | 137R>Q | 0 | 26 | 1 | 41 | 0,7860 | -0,0238 | 0,0000 |
| Rv3919c | gidB | 138A>E | 0 | 26 | 1 | 41 | 0,7860 | -0,0238 | 0,0000 |
| Rv3919c | gidB | 164G>S | 0 | 26 | 1 | 41 | 0,7860 | -0,0238 | 0,0000 |
| Rv3919c | gidB | 34G>V | 0 | 26 | 1 | 41 | 0,7860 | -0,0238 | 0,0000 |
| Rv3919c | gidB | 47R>W | 0 | 26 | 1 | 41 | 0,7860 | -0,0238 | 0,0000 |
| Rv3919c | gidB | 71G>V | 0 | 26 | 1 | 41 | 0,7860 | -0,0238 | 0,0000 |
| Rv3919c | gidB | 73G>R | 0 | 26 | 1 | 41 | 0,7860 | -0,0238 | 0,0000 |
| Rv3919c | gidB | 74L>F | 0 | 26 | 1 | 41 | 0,7860 | -0,0238 | 0,0000 |
| Rv3919c | gidB | 87Q>* | 0 | 26 | 1 | 41 | 0,7860 | -0,0238 | 0,0000 |
| Rv4002 | Rv1118c | 16R>R | 0 | 26 | 1 | 41 | 0,7860 | -0,0238 | 0,0000 |
| Rv4004 | Rv4004 | 9R>H | 0 | 26 | 1 | 41 | 0,7860 | -0,0238 | 0,0000 |
| Rv4013 | Rv4013 | 30G>D | 0 | 26 | 1 | 41 | 0,7860 | -0,0238 | 0,0000 |
| Rv0107c | ctpI | 105M>I | 3 | 23 | 8 | 34 | 0,7931 | -0,0751 | 0,5543 |
| Rv0170 | mce1B | 87V>A | 3 | 23 | 8 | 34 | 0,7931 | -0,0751 | 0,5543 |
| Rv0732 | secY | 431L>R | 3 | 23 | 8 | 34 | 0,7931 | -0,0751 | 0,5543 |
| Rv0945 | Rv0945 | 39R>H | 3 | 23 | 8 | 34 | 0,7931 | -0,0751 | 0,5543 |
| Rv1363c | Rv1363c | 84D>E | 3 | 23 | 8 | 34 | 0,7931 | -0,0751 | 0,5543 |
| Rv1569 | bioF1 | 161A>T | 3 | 23 | 8 | 34 | 0,7931 | -0,0751 | 0,5543 |
| Rv1588c | Rv1588c | 131V>I | 3 | 23 | 8 | 34 | 0,7931 | -0,0751 | 0,5543 |
| Rv2455c | Rv2455c | 596K>N | 3 | 23 | 8 | 34 | 0,7931 | -0,0751 | 0,5543 |
| Rv2537c | aroD | 81T>A | 3 | 23 | 8 | 34 | 0,7931 | -0,0751 | 0,5543 |
| Rv2714 | Rv2714 | 173E>G | 3 | 23 | 8 | 34 | 0,7931 | -0,0751 | 0,5543 |
| Rv3124 | Rv3124 | 116A>S | 3 | 23 | 8 | 34 | 0,7931 | -0,0751 | 0,5543 |
| Rv3395c | Rv3395c | 104V>M | 3 | 23 | 8 | 34 | 0,7931 | -0,0751 | 0,5543 |
| Rv3689 | Rv3689 | 263A>V | 3 | 23 | 8 | 34 | 0,7931 | -0,0751 | 0,5543 |
| Rv3793 | embC | 774A>S | 3 | 23 | 8 | 34 | 0,7931 | -0,0751 | 0,5543 |
| Rv3823c | mmpL8 | 38L>V | 3 | 23 | 8 | 34 | 0,7931 | -0,0751 | 0,5543 |
| Rv3896c | Rv3896c | 293A>V | 3 | 23 | 8 | 34 | 0,7931 | -0,0751 | 0,5543 |
| Rv1196 | PPE18 | 287R>Q | 2 | 24 | 6 | 36 | 0,7939 | -0,0659 | 0,5000 |
| Rv2346c | esxO | 52E>G | 2 | 24 | 6 | 36 | 0,7939 | -0,0659 | 0,5000 |
| Rv3620c | esxW | 2T>A | 2 | 24 | 6 | 36 | 0,7939 | -0,0659 | 0,5000 |
| Rv0166 | fadD5 | 532C>W | 1 | 25 | 4 | 38 | 0,8083 | -0,0568 | 0,3800 |

| Rv0261c | narK3 | 410N>S | 1 | 25 | 4 | 38 | 0,8083 | -0,0568 | 0,3800 |
| --- | --- | --- | --- | --- | --- | --- | --- | --- | --- |
| Rv0823c | Rv0823c | 156D>N | 1 | 25 | 4 | 38 | 0,8083 | -0,0568 | 0,3800 |
| Rv0834c | PE_PGRS14 | 654S>N | 1 | 25 | 4 | 38 | 0,8083 | -0,0568 | 0,3800 |
| Rv1014c | pth | 177I>S | 1 | 25 | 4 | 38 | 0,8083 | -0,0568 | 0,3800 |
| Rv1183 | mmpL10 | 384K>T | 1 | 25 | 4 | 38 | 0,8083 | -0,0568 | 0,3800 |
| Rv1289 | Rv1289 | 117P>S | 1 | 25 | 4 | 38 | 0,8083 | -0,0568 | 0,3800 |
| Rv1441c | PE_PGRS26 | 236D>G | 1 | 25 | 4 | 38 | 0,8083 | -0,0568 | 0,3800 |
| Rv1446c | opcA | 38A>T | 1 | 25 | 4 | 38 | 0,8083 | -0,0568 | 0,3800 |
| Rv1468c | PE_PGRS29 | 288G>C | 1 | 25 | 4 | 38 | 0,8083 | -0,0568 | 0,3800 |
| Rv1914c | Rv1914c | 16T>I | 1 | 25 | 4 | 38 | 0,8083 | -0,0568 | 0,3800 |
| Rv2242 | Rv2242 | 43S>L | 1 | 25 | 4 | 38 | 0,8083 | -0,0568 | 0,3800 |
| Rv2328 | PE23 | 314S>L | 1 | 25 | 4 | 38 | 0,8083 | -0,0568 | 0,3800 |
| Rv2342 | Rv2342 | 3G>R | 1 | 25 | 4 | 38 | 0,8083 | -0,0568 | 0,3800 |
| Rv2391 | nirA | 372L>R | 1 | 25 | 4 | 38 | 0,8083 | -0,0568 | 0,3800 |
| Rv2652c | Rv2652c | 5A>E | 1 | 25 | 4 | 38 | 0,8083 | -0,0568 | 0,3800 |
| Rv2666 | Rv2666 | 9T>A | 1 | 25 | 4 | 38 | 0,8083 | -0,0568 | 0,3800 |
| Rv2800 | Rv2800 | 182S>A | 1 | 25 | 4 | 38 | 0,8083 | -0,0568 | 0,3800 |
| Rv3034c | Rv3034c | 2N>S | 1 | 25 | 4 | 38 | 0,8083 | -0,0568 | 0,3800 |
| Rv3209 | Rv3209 | 75T>S | 1 | 25 | 4 | 38 | 0,8083 | -0,0568 | 0,3800 |
| Rv3347c | PPE55 | 1003F>D | 1 | 25 | 4 | 38 | 0,8083 | -0,0568 | 0,3800 |
| Rv3347c | PPE55 | 922S>G | 1 | 25 | 4 | 38 | 0,8083 | -0,0568 | 0,3800 |
| Rv3347c | PPE55 | 923F>Y | 1 | 25 | 4 | 38 | 0,8083 | -0,0568 | 0,3800 |
| Rv3347c | PPE55 | 994D>N | 1 | 25 | 4 | 38 | 0,8083 | -0,0568 | 0,3800 |
| Rv3347c | PPE55 | 998L>F | 1 | 25 | 4 | 38 | 0,8083 | -0,0568 | 0,3800 |
| Rv3347c | PPE55 | 999M>I | 1 | 25 | 4 | 38 | 0,8083 | -0,0568 | 0,3800 |
| Rv3349c | Rv3349c | 176L>V | 1 | 25 | 4 | 38 | 0,8083 | -0,0568 | 0,3800 |
| Rv3350c | PPE56 | 1697L>R | 1 | 25 | 4 | 38 | 0,8083 | -0,0568 | 0,3800 |
| Rv3350c | PPE56 | 1971V>A | 1 | 25 | 4 | 38 | 0,8083 | -0,0568 | 0,3800 |
| Rv3428c | Rv3428c | 121L>P | 1 | 25 | 4 | 38 | 0,8083 | -0,0568 | 0,3800 |
| Rv3466 | Rv3466 | 47V>A | 1 | 25 | 4 | 38 | 0,8083 | -0,0568 | 0,3800 |
| Rv3590c | PE_PGRS58 | 314A>V | 1 | 25 | 4 | 38 | 0,8083 | -0,0568 | 0,3800 |
| Rv3824c | papA1 | 92L>V | 1 | 25 | 4 | 38 | 0,8083 | -0,0568 | 0,3800 |
| Rv3831 | Rv3831 | 118G>V | 1 | 25 | 4 | 38 | 0,8083 | -0,0568 | 0,3800 |

| Rv3900c | Rv3900c | 256A>V | 1 | 25 | 4 | 38 | 0,8083 | -0,0568 | 0,3800 |
| --- | --- | --- | --- | --- | --- | --- | --- | --- | --- |
| Rv0094c | Rv0094c | 262G>D | 4 | 22 | 11 | 31 | 0,8518 | -0,1081 | 0,5124 |
| Rv1622c | cydB | 284N>K | 2 | 24 | 7 | 35 | 0,8557 | -0,0897 | 0,4167 |
| Rv0001 | dnaA | 124P>L | 0 | 26 | 2 | 40 | 0,8706 | -0,0476 | 0,0000 |
| Rv0001 | dnaA | 28D>H | 0 | 26 | 2 | 40 | 0,8706 | -0,0476 | 0,0000 |
| Rv0013 | trpG | 70H>R | 0 | 26 | 2 | 40 | 0,8706 | -0,0476 | 0,0000 |
| Rv0020c | TB39.8 | 184E>G | 0 | 26 | 2 | 40 | 0,8706 | -0,0476 | 0,0000 |
| Rv0031 | Rv0031 | 30A>V | 0 | 26 | 2 | 40 | 0,8706 | -0,0476 | 0,0000 |
| Rv0036c | Rv0036c | 50W>* | 0 | 26 | 2 | 40 | 0,8706 | -0,0476 | 0,0000 |
| Rv0041 | leuS | 925V>M | 0 | 26 | 2 | 40 | 0,8706 | -0,0476 | 0,0000 |
| Rv0050 | ponA1 | 630P>S | 0 | 26 | 2 | 40 | 0,8706 | -0,0476 | 0,0000 |
| Rv0057 | Rv0057 | 79A>V | 0 | 26 | 2 | 40 | 0,8706 | -0,0476 | 0,0000 |
| Rv0058 | dnaB | 650D>G | 0 | 26 | 2 | 40 | 0,8706 | -0,0476 | 0,0000 |
| Rv0064 | Rv0064 | 409Q>H | 0 | 26 | 2 | 40 | 0,8706 | -0,0476 | 0,0000 |
| Rv0071 | Rv0071 | 100V>M | 0 | 26 | 2 | 40 | 0,8706 | -0,0476 | 0,0000 |
| Rv0101 | nrp | 2123L>M | 0 | 26 | 2 | 40 | 0,8706 | -0,0476 | 0,0000 |
| Rv0102 | Rv0102 | 56P>R | 0 | 26 | 2 | 40 | 0,8706 | -0,0476 | 0,0000 |
| Rv0113 | gmhA | 122T>S | 0 | 26 | 2 | 40 | 0,8706 | -0,0476 | 0,0000 |
| Rv0118c | oxcA | 388V>A | 0 | 26 | 2 | 40 | 0,8706 | -0,0476 | 0,0000 |
| Rv0120c | fusA2 | 479H>P | 0 | 26 | 2 | 40 | 0,8706 | -0,0476 | 0,0000 |
| Rv0125 | pepA | 8S>A | 0 | 26 | 2 | 40 | 0,8706 | -0,0476 | 0,0000 |
| Rv0132c | fgd2 | 330D>G | 0 | 26 | 2 | 40 | 0,8706 | -0,0476 | 0,0000 |
| Rv0140 | Rv0140 | 6V>M | 0 | 26 | 2 | 40 | 0,8706 | -0,0476 | 0,0000 |
| Rv0158 | Rv0158 | 8N>S | 0 | 26 | 2 | 40 | 0,8706 | -0,0476 | 0,0000 |
| Rv0169 | mce1A | 63K>E | 0 | 26 | 2 | 40 | 0,8706 | -0,0476 | 0,0000 |
| Rv0171 | mce1C | 322D>G | 0 | 26 | 2 | 40 | 0,8706 | -0,0476 | 0,0000 |
| Rv0175 | Rv0175 | 65L>P | 0 | 26 | 2 | 40 | 0,8706 | -0,0476 | 0,0000 |
| Rv0194 | Rv0194 | 86H>Y | 0 | 26 | 2 | 40 | 0,8706 | -0,0476 | 0,0000 |
| Rv0206c | mmpL3 | 534P>S | 0 | 26 | 2 | 40 | 0,8706 | -0,0476 | 0,0000 |
| Rv0213c | Rv0213c | 346K>R | 0 | 26 | 2 | 40 | 0,8706 | -0,0476 | 0,0000 |
| Rv0236c | Rv0236c | 755G>E | 0 | 26 | 2 | 40 | 0,8706 | -0,0476 | 0,0000 |
| Rv0276 | Rv0276 | 118V>M | 0 | 26 | 2 | 40 | 0,8706 | -0,0476 | 0,0000 |
| Rv0291 | mycP3 | 13V>G | 0 | 26 | 2 | 40 | 0,8706 | -0,0476 | 0,0000 |

| Rv0303 | Rv0303 | 94V>M | 0 | 26 | 2 | 40 | 0,8706 | -0,0476 | 0,0000 |
| --- | --- | --- | --- | --- | --- | --- | --- | --- | --- |
| Rv0318c | Rv0318c | 195I>V | 0 | 26 | 2 | 40 | 0,8706 | -0,0476 | 0,0000 |
| Rv0332 | Rv0332 | 157G>R | 0 | 26 | 2 | 40 | 0,8706 | -0,0476 | 0,0000 |
| Rv0367c | Rv0367c | 41G>D | 0 | 26 | 2 | 40 | 0,8706 | -0,0476 | 0,0000 |
| Rv0374c | Rv0374c | 129I>V | 0 | 26 | 2 | 40 | 0,8706 | -0,0476 | 0,0000 |
| Rv0376c | Rv0376c | 301D>E | 0 | 26 | 2 | 40 | 0,8706 | -0,0476 | 0,0000 |
| Rv0384c | clpB | 672P>S | 0 | 26 | 2 | 40 | 0,8706 | -0,0476 | 0,0000 |
| Rv0395 | Rv0395 | 104N>K | 0 | 26 | 2 | 40 | 0,8706 | -0,0476 | 0,0000 |
| Rv0412c | Rv0412c | 223A>T | 0 | 26 | 2 | 40 | 0,8706 | -0,0476 | 0,0000 |
| Rv0415 | thiO | 45W>C | 0 | 26 | 2 | 40 | 0,8706 | -0,0476 | 0,0000 |
| Rv0424c | Rv0424c | 36K>N | 0 | 26 | 2 | 40 | 0,8706 | -0,0476 | 0,0000 |
| Rv0471c | Rv0471c | 54G>E | 0 | 26 | 2 | 40 | 0,8706 | -0,0476 | 0,0000 |
| Rv0509 | hemA | 306V>L | 0 | 26 | 2 | 40 | 0,8706 | -0,0476 | 0,0000 |
| Rv0535 | pnp | 169W>G | 0 | 26 | 2 | 40 | 0,8706 | -0,0476 | 0,0000 |
| Rv0580c | Rv0580c | 22N>D | 0 | 26 | 2 | 40 | 0,8706 | -0,0476 | 0,0000 |
| Rv0594 | mce2F | 221N>K | 0 | 26 | 2 | 40 | 0,8706 | -0,0476 | 0,0000 |
| Rv0620 | galK | 60P>S | 0 | 26 | 2 | 40 | 0,8706 | -0,0476 | 0,0000 |
| Rv0628c | Rv0628c | 372H>Y | 0 | 26 | 2 | 40 | 0,8706 | -0,0476 | 0,0000 |
| Rv0653c | Rv0653c | 54I>T | 0 | 26 | 2 | 40 | 0,8706 | -0,0476 | 0,0000 |
| Rv0658c | Rv0658c | 42G>D | 0 | 26 | 2 | 40 | 0,8706 | -0,0476 | 0,0000 |
| Rv0667 | rpoB | 170V>F | 0 | 26 | 2 | 40 | 0,8706 | -0,0476 | 0,0000 |
| Rv0668 | rpoC | 416N>S | 0 | 26 | 2 | 40 | 0,8706 | -0,0476 | 0,0000 |
| Rv0687 | fabG | 74V>L | 0 | 26 | 2 | 40 | 0,8706 | -0,0476 | 0,0000 |
| Rv0694 | lldD1 | 29I>M | 0 | 26 | 2 | 40 | 0,8706 | -0,0476 | 0,0000 |
| Rv0696 | Rv0696 | 453Y>C | 0 | 26 | 2 | 40 | 0,8706 | -0,0476 | 0,0000 |
| Rv0713 | Rv0713 | 199G>R | 0 | 26 | 2 | 40 | 0,8706 | -0,0476 | 0,0000 |
| Rv0737 | Rv0737 | 164D>E | 0 | 26 | 2 | 40 | 0,8706 | -0,0476 | 0,0000 |
| Rv0791c | Rv0791c | 286A>T | 0 | 26 | 2 | 40 | 0,8706 | -0,0476 | 0,0000 |
| Rv0848 | cysK2 | 199S>L | 0 | 26 | 2 | 40 | 0,8706 | -0,0476 | 0,0000 |
| Rv0857 | Rv0857 | 8V>A | 0 | 26 | 2 | 40 | 0,8706 | -0,0476 | 0,0000 |
| Rv0873 | fadE10 | 27P>S | 0 | 26 | 2 | 40 | 0,8706 | -0,0476 | 0,0000 |
| Rv0899 | ompA | 53P>H | 0 | 26 | 2 | 40 | 0,8706 | -0,0476 | 0,0000 |
| Rv0904c | accD3 | 147T>M | 0 | 26 | 2 | 40 | 0,8706 | -0,0476 | 0,0000 |

| Rv0931c | pknD | 343S>N | 0 | 26 | 2 | 40 | 0,8706 | -0,0476 | 0,0000 |
| --- | --- | --- | --- | --- | --- | --- | --- | --- | --- |
| Rv0933 | pstB | 223A>S | 0 | 26 | 2 | 40 | 0,8706 | -0,0476 | 0,0000 |
| Rv0949 | uvrD1 | 462G>S | 0 | 26 | 2 | 40 | 0,8706 | -0,0476 | 0,0000 |
| Rv0989c | grcC2 | 142A>T | 0 | 26 | 2 | 40 | 0,8706 | -0,0476 | 0,0000 |
| Rv1002c | Rv1002c | 1V>V | 0 | 26 | 2 | 40 | 0,8706 | -0,0476 | 0,0000 |
| Rv1028c | kdpD | 416A>P | 0 | 26 | 2 | 40 | 0,8706 | -0,0476 | 0,0000 |
| Rv1057 | Rv1057 | 226R>Q | 0 | 26 | 2 | 40 | 0,8706 | -0,0476 | 0,0000 |
| Rv1068c | PE_PGRS20 | 111L>W | 0 | 26 | 2 | 40 | 0,8706 | -0,0476 | 0,0000 |
| Rv1083 | mca | 289N>D | 0 | 26 | 2 | 40 | 0,8706 | -0,0476 | 0,0000 |
| Rv1089 | PE9 | 119N>D | 0 | 26 | 2 | 40 | 0,8706 | -0,0476 | 0,0000 |
| Rv1091 | PE_PGRS22 | 369G>N | 0 | 26 | 2 | 40 | 0,8706 | -0,0476 | 0,0000 |
| Rv1091 | PE_PGRS22 | 689N>S | 0 | 26 | 2 | 40 | 0,8706 | -0,0476 | 0,0000 |
| Rv1104 | Rv1104 | 145G>S | 0 | 26 | 2 | 40 | 0,8706 | -0,0476 | 0,0000 |
| Rv1115 | Rv1115 | 157V>G | 0 | 26 | 2 | 40 | 0,8706 | -0,0476 | 0,0000 |
| Rv1116 | Rv1116 | 37R>G | 0 | 26 | 2 | 40 | 0,8706 | -0,0476 | 0,0000 |
| Rv1122 | gnd2 | 318D>Y | 0 | 26 | 2 | 40 | 0,8706 | -0,0476 | 0,0000 |
| Rv1130 | Rv1130 | 10F>C | 0 | 26 | 2 | 40 | 0,8706 | -0,0476 | 0,0000 |
| Rv1171 | Rv1171 | 145S>P | 0 | 26 | 2 | 40 | 0,8706 | -0,0476 | 0,0000 |
| Rv1183 | mmpL10 | 521A>V | 0 | 26 | 2 | 40 | 0,8706 | -0,0476 | 0,0000 |
| Rv1192 | Rv1192 | 227P>L | 0 | 26 | 2 | 40 | 0,8706 | -0,0476 | 0,0000 |
| Rv1198 | esxL | 32I>V | 0 | 26 | 2 | 40 | 0,8706 | -0,0476 | 0,0000 |
| Rv1198 | esxL | 48A>V | 0 | 26 | 2 | 40 | 0,8706 | -0,0476 | 0,0000 |
| Rv1198 | esxL | 76Q>* | 0 | 26 | 2 | 40 | 0,8706 | -0,0476 | 0,0000 |
| Rv1200 | Rv1200 | 165V>L | 0 | 26 | 2 | 40 | 0,8706 | -0,0476 | 0,0000 |
| Rv1215c | Rv1215c | 345D>G | 0 | 26 | 2 | 40 | 0,8706 | -0,0476 | 0,0000 |
| Rv1226c | Rv1226c | 479D>N | 0 | 26 | 2 | 40 | 0,8706 | -0,0476 | 0,0000 |
| Rv1244 | lpqZ | 194A>T | 0 | 26 | 2 | 40 | 0,8706 | -0,0476 | 0,0000 |
| Rv1277 | Rv1277 | 304I>L | 0 | 26 | 2 | 40 | 0,8706 | -0,0476 | 0,0000 |
| Rv1290c | Rv1290c | 481A>S | 0 | 26 | 2 | 40 | 0,8706 | -0,0476 | 0,0000 |
| Rv1310 | atpD | 131I>V | 0 | 26 | 2 | 40 | 0,8706 | -0,0476 | 0,0000 |
| Rv1317c | alkA | 313R>C | 0 | 26 | 2 | 40 | 0,8706 | -0,0476 | 0,0000 |
| Rv1336 | cysM | 60E>* | 0 | 26 | 2 | 40 | 0,8706 | -0,0476 | 0,0000 |
| Rv1337 | Rv1337 | 22R>H | 0 | 26 | 2 | 40 | 0,8706 | -0,0476 | 0,0000 |

| Rv1348 | Rv1348 | 859R>* | 0 | 26 | 2 | 40 | 0,8706 | -0,0476 | 0,0000 |
| --- | --- | --- | --- | --- | --- | --- | --- | --- | --- |
| Rv1376 | Rv1376 | 183P>L | 0 | 26 | 2 | 40 | 0,8706 | -0,0476 | 0,0000 |
| Rv1425 | Rv1425 | 128G>C | 0 | 26 | 2 | 40 | 0,8706 | -0,0476 | 0,0000 |
| Rv1484 | inhA | 95I>L | 0 | 26 | 2 | 40 | 0,8706 | -0,0476 | 0,0000 |
| Rv1527c | pks5 | 393A>E | 0 | 26 | 2 | 40 | 0,8706 | -0,0476 | 0,0000 |
| Rv1559 | ilvA | 147A>V | 0 | 26 | 2 | 40 | 0,8706 | -0,0476 | 0,0000 |
| Rv1563c | treY | 719T>I | 0 | 26 | 2 | 40 | 0,8706 | -0,0476 | 0,0000 |
| Rv1609 | trpE | 215S>P | 0 | 26 | 2 | 40 | 0,8706 | -0,0476 | 0,0000 |
| Rv1638 | uvrA | 806I>T | 0 | 26 | 2 | 40 | 0,8706 | -0,0476 | 0,0000 |
| Rv1661 | pks7 | 1814E>K | 0 | 26 | 2 | 40 | 0,8706 | -0,0476 | 0,0000 |
| Rv1661 | pks7 | 6E>K | 0 | 26 | 2 | 40 | 0,8706 | -0,0476 | 0,0000 |
| Rv1662 | pks8 | 141D>N | 0 | 26 | 2 | 40 | 0,8706 | -0,0476 | 0,0000 |
| Rv1663 | pks17 | 28P>T | 0 | 26 | 2 | 40 | 0,8706 | -0,0476 | 0,0000 |
| Rv1672c | Rv1672c | 145V>I | 0 | 26 | 2 | 40 | 0,8706 | -0,0476 | 0,0000 |
| Rv1727 | Rv1727 | 33D>N | 0 | 26 | 2 | 40 | 0,8706 | -0,0476 | 0,0000 |
| Rv1733c | Rv1733c | 30S>I | 0 | 26 | 2 | 40 | 0,8706 | -0,0476 | 0,0000 |
| Rv1743 | pknE | 91V>A | 0 | 26 | 2 | 40 | 0,8706 | -0,0476 | 0,0000 |
| Rv1770 | Rv1770 | 252V>L | 0 | 26 | 2 | 40 | 0,8706 | -0,0476 | 0,0000 |
| Rv1776c | Rv1776c | 32G>R | 0 | 26 | 2 | 40 | 0,8706 | -0,0476 | 0,0000 |
| Rv1797 | Rv1797 | 165S>L | 0 | 26 | 2 | 40 | 0,8706 | -0,0476 | 0,0000 |
| Rv1810 | Rv1810 | 118S>R | 0 | 26 | 2 | 40 | 0,8706 | -0,0476 | 0,0000 |
| Rv1831 | Rv1831 | 79L>F | 0 | 26 | 2 | 40 | 0,8706 | -0,0476 | 0,0000 |
| Rv1844c | gnd1 | 237D>G | 0 | 26 | 2 | 40 | 0,8706 | -0,0476 | 0,0000 |
| Rv1877 | Rv1877 | 631I>S | 0 | 26 | 2 | 40 | 0,8706 | -0,0476 | 0,0000 |
| Rv1887 | Rv1887 | 83Q>R | 0 | 26 | 2 | 40 | 0,8706 | -0,0476 | 0,0000 |
| Rv1894c | Rv1894c | 373A>T | 0 | 26 | 2 | 40 | 0,8706 | -0,0476 | 0,0000 |
| Rv1902c | nanT | 74V>L | 0 | 26 | 2 | 40 | 0,8706 | -0,0476 | 0,0000 |
| Rv1907c | Rv1907c | 129A>V | 0 | 26 | 2 | 40 | 0,8706 | -0,0476 | 0,0000 |
| Rv1908c | katG | 315S>N | 0 | 26 | 2 | 40 | 0,8706 | -0,0476 | 0,0000 |
| Rv1917c | PPE34 | 1378S>F | 0 | 26 | 2 | 40 | 0,8706 | -0,0476 | 0,0000 |
| Rv1921c | lppF | 236A>P | 0 | 26 | 2 | 40 | 0,8706 | -0,0476 | 0,0000 |
| Rv1933c | fadE18 | 261A>V | 0 | 26 | 2 | 40 | 0,8706 | -0,0476 | 0,0000 |
| Rv1945 | Rv1945 | 291V>I | 0 | 26 | 2 | 40 | 0,8706 | -0,0476 | 0,0000 |

| Rv1952 | Rv1952 | 10K>R | 0 | 26 | 2 | 40 | 0,8706 | -0,0476 | 0,0000 |
| --- | --- | --- | --- | --- | --- | --- | --- | --- | --- |
| Rv1958c | Rv1958c | 121P>S | 0 | 26 | 2 | 40 | 0,8706 | -0,0476 | 0,0000 |
| Rv1969 | mce3D | 181I>V | 0 | 26 | 2 | 40 | 0,8706 | -0,0476 | 0,0000 |
| Rv2001 | Rv2001 | 17S>L | 0 | 26 | 2 | 40 | 0,8706 | -0,0476 | 0,0000 |
| Rv2004c | Rv2004c | 397L>F | 0 | 26 | 2 | 40 | 0,8706 | -0,0476 | 0,0000 |
| Rv2006 | otsB1 | 1276F>L | 0 | 26 | 2 | 40 | 0,8706 | -0,0476 | 0,0000 |
| Rv2013 | Rv2013 | 104L>F | 0 | 26 | 2 | 40 | 0,8706 | -0,0476 | 0,0000 |
| Rv2022c | Rv2022c | 20D>N | 0 | 26 | 2 | 40 | 0,8706 | -0,0476 | 0,0000 |
| Rv2043c | pncA | 14C>G | 0 | 26 | 2 | 40 | 0,8706 | -0,0476 | 0,0000 |
| Rv2077c | Rv2077c | 324*>E | 0 | 26 | 2 | 40 | 0,8706 | -0,0476 | 0,0000 |
| Rv2101 | helZ | 482I>V | 0 | 26 | 2 | 40 | 0,8706 | -0,0476 | 0,0000 |
| Rv2119 | Rv2119 | 143Y>C | 0 | 26 | 2 | 40 | 0,8706 | -0,0476 | 0,0000 |
| Rv2138 | lppL | 102H>Q | 0 | 26 | 2 | 40 | 0,8706 | -0,0476 | 0,0000 |
| Rv2151c | ftsQ | 82S>R | 0 | 26 | 2 | 40 | 0,8706 | -0,0476 | 0,0000 |
| Rv2152c | murC | 431H>R | 0 | 26 | 2 | 40 | 0,8706 | -0,0476 | 0,0000 |
| Rv2207 | cobT | 227D>G | 0 | 26 | 2 | 40 | 0,8706 | -0,0476 | 0,0000 |
| Rv2217 | lipB | 59T>A | 0 | 26 | 2 | 40 | 0,8706 | -0,0476 | 0,0000 |
| Rv2221c | glnE | 606M>T | 0 | 26 | 2 | 40 | 0,8706 | -0,0476 | 0,0000 |
| Rv2221c | glnE | 800Q>R | 0 | 26 | 2 | 40 | 0,8706 | -0,0476 | 0,0000 |
| Rv2230c | Rv2230c | 260R>C | 0 | 26 | 2 | 40 | 0,8706 | -0,0476 | 0,0000 |
| Rv2231c | cobC | 349V>A | 0 | 26 | 2 | 40 | 0,8706 | -0,0476 | 0,0000 |
| Rv2241 | aceE | 129V>E | 0 | 26 | 2 | 40 | 0,8706 | -0,0476 | 0,0000 |
| Rv2298 | Rv2298 | 65E>A | 0 | 26 | 2 | 40 | 0,8706 | -0,0476 | 0,0000 |
| Rv2305 | Rv2305 | 239A>V | 0 | 26 | 2 | 40 | 0,8706 | -0,0476 | 0,0000 |
| Rv2310 | Rv2310 | 44E>K | 0 | 26 | 2 | 40 | 0,8706 | -0,0476 | 0,0000 |
| Rv2329c | narK1 | 234M>I | 0 | 26 | 2 | 40 | 0,8706 | -0,0476 | 0,0000 |
| Rv2338c | moeW | 189D>G | 0 | 26 | 2 | 40 | 0,8706 | -0,0476 | 0,0000 |
| Rv2339 | mmpL9 | 241T>P | 0 | 26 | 2 | 40 | 0,8706 | -0,0476 | 0,0000 |
| Rv2339 | mmpL9 | 374R>P | 0 | 26 | 2 | 40 | 0,8706 | -0,0476 | 0,0000 |
| Rv2348c | Rv2348c | 101I>M | 0 | 26 | 2 | 40 | 0,8706 | -0,0476 | 0,0000 |
| Rv2367c | Rv2367c | 21A>V | 0 | 26 | 2 | 40 | 0,8706 | -0,0476 | 0,0000 |
| Rv2381c | mbtD | 907Y>C | 0 | 26 | 2 | 40 | 0,8706 | -0,0476 | 0,0000 |
| Rv2444c | rne | 93E>D | 0 | 26 | 2 | 40 | 0,8706 | -0,0476 | 0,0000 |

| Rv2460c | clpP2 | 195K>T | 0 | 26 | 2 | 40 | 0,8706 | -0,0476 | 0,0000 |
| --- | --- | --- | --- | --- | --- | --- | --- | --- | --- |
| Rv2473 | Rv2473 | 159G>D | 0 | 26 | 2 | 40 | 0,8706 | -0,0476 | 0,0000 |
| Rv2501c | accA1 | 142D>V | 0 | 26 | 2 | 40 | 0,8706 | -0,0476 | 0,0000 |
| Rv2514c | Rv2514c | 78A>V | 0 | 26 | 2 | 40 | 0,8706 | -0,0476 | 0,0000 |
| Rv2522c | Rv2522c | 376I>L | 0 | 26 | 2 | 40 | 0,8706 | -0,0476 | 0,0000 |
| Rv2524c | fas | 1045A>G | 0 | 26 | 2 | 40 | 0,8706 | -0,0476 | 0,0000 |
| Rv2546 | Rv2546 | 130W>G | 0 | 26 | 2 | 40 | 0,8706 | -0,0476 | 0,0000 |
| Rv2567 | Rv2567 | 792E>K | 0 | 26 | 2 | 40 | 0,8706 | -0,0476 | 0,0000 |
| Rv2575 | Rv2575 | 30G>S | 0 | 26 | 2 | 40 | 0,8706 | -0,0476 | 0,0000 |
| Rv2581c | Rv2581c | 24T>I | 0 | 26 | 2 | 40 | 0,8706 | -0,0476 | 0,0000 |
| Rv2601A | Rv2601A | 62G>D | 0 | 26 | 2 | 40 | 0,8706 | -0,0476 | 0,0000 |
| Rv2643 | arsC | 49L>V | 0 | 26 | 2 | 40 | 0,8706 | -0,0476 | 0,0000 |
| Rv2647 | Rv2647 | 1V>V | 0 | 26 | 2 | 40 | 0,8706 | -0,0476 | 0,0000 |
| Rv2681 | Rv2681 | 267D>G | 0 | 26 | 2 | 40 | 0,8706 | -0,0476 | 0,0000 |
| Rv2703 | sigA | 389D>G | 0 | 26 | 2 | 40 | 0,8706 | -0,0476 | 0,0000 |
| Rv2733c | Rv2733c | 60S>A | 0 | 26 | 2 | 40 | 0,8706 | -0,0476 | 0,0000 |
| Rv2735c | Rv2735c | 261Y>H | 0 | 26 | 2 | 40 | 0,8706 | -0,0476 | 0,0000 |
| Rv2782c | pepR | 225G>R | 0 | 26 | 2 | 40 | 0,8706 | -0,0476 | 0,0000 |
| Rv2832c | ugpC | 254R>W | 0 | 26 | 2 | 40 | 0,8706 | -0,0476 | 0,0000 |
| Rv2834c | ugpE | 59T>M | 0 | 26 | 2 | 40 | 0,8706 | -0,0476 | 0,0000 |
| Rv2854 | Rv2854 | 37G>S | 0 | 26 | 2 | 40 | 0,8706 | -0,0476 | 0,0000 |
| Rv2860c | glnA4 | 249S>N | 0 | 26 | 2 | 40 | 0,8706 | -0,0476 | 0,0000 |
| Rv2892c | Rv2891 | 205S>S | 0 | 26 | 2 | 40 | 0,8706 | -0,0476 | 0,0000 |
| Rv2897c | Rv2897c | 15D>G | 0 | 26 | 2 | 40 | 0,8706 | -0,0476 | 0,0000 |
| Rv2913c | Rv2913c | 493P>S | 0 | 26 | 2 | 40 | 0,8706 | -0,0476 | 0,0000 |
| Rv2918c | glnD | 469T>P | 0 | 26 | 2 | 40 | 0,8706 | -0,0476 | 0,0000 |
| Rv2920c | amt | 71A>T | 0 | 26 | 2 | 40 | 0,8706 | -0,0476 | 0,0000 |
| Rv2926c | Rv2926c | 164P>S | 0 | 26 | 2 | 40 | 0,8706 | -0,0476 | 0,0000 |
| Rv2933 | ppsC | 805P>S | 0 | 26 | 2 | 40 | 0,8706 | -0,0476 | 0,0000 |
| Rv2946c | pks1 | 880T>I | 0 | 26 | 2 | 40 | 0,8706 | -0,0476 | 0,0000 |
| Rv2947c | pks15 | 290R>L | 0 | 26 | 2 | 40 | 0,8706 | -0,0476 | 0,0000 |
| Rv2950c | fadD29 | 563R>G | 0 | 26 | 2 | 40 | 0,8706 | -0,0476 | 0,0000 |
| Rv2979c | Rv2979c | 161I>S | 0 | 26 | 2 | 40 | 0,8706 | -0,0476 | 0,0000 |

| Rv3011c | gatA | 233A>T | 0 | 26 | 2 | 40 | 0,8706 | -0,0476 | 0,0000 |
| --- | --- | --- | --- | --- | --- | --- | --- | --- | --- |
| Rv3014c | ligA | 448R>L | 0 | 26 | 2 | 40 | 0,8706 | -0,0476 | 0,0000 |
| Rv3062 | ligB | 192A>T | 0 | 26 | 2 | 40 | 0,8706 | -0,0476 | 0,0000 |
| Rv3063 | cstA | 314R>H | 0 | 26 | 2 | 40 | 0,8706 | -0,0476 | 0,0000 |
| Rv3063 | cstA | 7S>* | 0 | 26 | 2 | 40 | 0,8706 | -0,0476 | 0,0000 |
| Rv3071 | Rv3071 | 125A>S | 0 | 26 | 2 | 40 | 0,8706 | -0,0476 | 0,0000 |
| Rv3079c | Rv3079c | 220G>V | 0 | 26 | 2 | 40 | 0,8706 | -0,0476 | 0,0000 |
| Rv3099c | Rv3099c | 213S>P | 0 | 26 | 2 | 40 | 0,8706 | -0,0476 | 0,0000 |
| Rv3113 | Rv3113 | 91V>A | 0 | 26 | 2 | 40 | 0,8706 | -0,0476 | 0,0000 |
| Rv3122 | Rv3122 | 77T>A | 0 | 26 | 2 | 40 | 0,8706 | -0,0476 | 0,0000 |
| Rv3146 | nuoB | 47G>A | 0 | 26 | 2 | 40 | 0,8706 | -0,0476 | 0,0000 |
| Rv3150 | nuoF | 378G>S | 0 | 26 | 2 | 40 | 0,8706 | -0,0476 | 0,0000 |
| Rv3194c | Rv3194c | 300K>N | 0 | 26 | 2 | 40 | 0,8706 | -0,0476 | 0,0000 |
| Rv3224 | Rv3224 | 21I>T | 0 | 26 | 2 | 40 | 0,8706 | -0,0476 | 0,0000 |
| Rv3227 | aroA | 88A>T | 0 | 26 | 2 | 40 | 0,8706 | -0,0476 | 0,0000 |
| Rv3233c | Rv3233c | 82S>G | 0 | 26 | 2 | 40 | 0,8706 | -0,0476 | 0,0000 |
| Rv3241c | Rv3241c | 141G>R | 0 | 26 | 2 | 40 | 0,8706 | -0,0476 | 0,0000 |
| Rv3305c | amiA1 | 216V>I | 0 | 26 | 2 | 40 | 0,8706 | -0,0476 | 0,0000 |
| Rv3305c | amiA1 | 326R>W | 0 | 26 | 2 | 40 | 0,8706 | -0,0476 | 0,0000 |
| Rv3312A | Rv3312A | 6C>F | 0 | 26 | 2 | 40 | 0,8706 | -0,0476 | 0,0000 |
| Rv3327 | Rv3327 | 112R>P | 0 | 26 | 2 | 40 | 0,8706 | -0,0476 | 0,0000 |
| Rv3350c | PPE56 | 2221L>V | 0 | 26 | 2 | 40 | 0,8706 | -0,0476 | 0,0000 |
| Rv3350c | PPE56 | 2223L>I | 0 | 26 | 2 | 40 | 0,8706 | -0,0476 | 0,0000 |
| Rv3350c | PPE56 | 2232I>V | 0 | 26 | 2 | 40 | 0,8706 | -0,0476 | 0,0000 |
| Rv3365c | Rv3365c | 154V>M | 0 | 26 | 2 | 40 | 0,8706 | -0,0476 | 0,0000 |
| Rv3373 | echA18 | 40R>H | 0 | 26 | 2 | 40 | 0,8706 | -0,0476 | 0,0000 |
| Rv3388 | PE_PGRS52 | 230G>D | 0 | 26 | 2 | 40 | 0,8706 | -0,0476 | 0,0000 |
| Rv3391 | acrA1 | 330G>E | 0 | 26 | 2 | 40 | 0,8706 | -0,0476 | 0,0000 |
| Rv3445c | esxU | 33L>P | 0 | 26 | 2 | 40 | 0,8706 | -0,0476 | 0,0000 |
| Rv3447c | Rv3447c | 385S>N | 0 | 26 | 2 | 40 | 0,8706 | -0,0476 | 0,0000 |
| Rv3449 | mycP4 | 92Q>* | 0 | 26 | 2 | 40 | 0,8706 | -0,0476 | 0,0000 |
| Rv3467 | Rv3467 | 303P>H | 0 | 26 | 2 | 40 | 0,8706 | -0,0476 | 0,0000 |
| Rv3479 | Rv3479 | 446F>L | 0 | 26 | 2 | 40 | 0,8706 | -0,0476 | 0,0000 |

| Rv3497c | mce4C | 253S>Y | 0 | 26 | 2 | 40 | 0,8706 | -0,0476 | 0,0000 |
| --- | --- | --- | --- | --- | --- | --- | --- | --- | --- |
| Rv3505 | fadE27 | 30R>H | 0 | 26 | 2 | 40 | 0,8706 | -0,0476 | 0,0000 |
| Rv3508 | PE_PGRS54 | 764G>A | 0 | 26 | 2 | 40 | 0,8706 | -0,0476 | 0,0000 |
| Rv3526 | Rv3526 | 342D>G | 0 | 26 | 2 | 40 | 0,8706 | -0,0476 | 0,0000 |
| Rv3529c | Rv3529c | 91K>E | 0 | 26 | 2 | 40 | 0,8706 | -0,0476 | 0,0000 |
| Rv3541c | Rv3541c | 102G>S | 0 | 26 | 2 | 40 | 0,8706 | -0,0476 | 0,0000 |
| Rv3565 | aspB | 358R>Q | 0 | 26 | 2 | 40 | 0,8706 | -0,0476 | 0,0000 |
| Rv3596c | clpC1 | 165P>L | 0 | 26 | 2 | 40 | 0,8706 | -0,0476 | 0,0000 |
| Rv3618 | Rv3618 | 74R>W | 0 | 26 | 2 | 40 | 0,8706 | -0,0476 | 0,0000 |
| Rv3639c | Rv3639c | 134R>G | 0 | 26 | 2 | 40 | 0,8706 | -0,0476 | 0,0000 |
| Rv3680 | Rv3680 | 378N>D | 0 | 26 | 2 | 40 | 0,8706 | -0,0476 | 0,0000 |
| Rv3689 | Rv3689 | 410Y>S | 0 | 26 | 2 | 40 | 0,8706 | -0,0476 | 0,0000 |
| Rv3691 | Rv3691 | 283A>S | 0 | 26 | 2 | 40 | 0,8706 | -0,0476 | 0,0000 |
| Rv3714c | Rv3714c | 271R>G | 0 | 26 | 2 | 40 | 0,8706 | -0,0476 | 0,0000 |
| Rv3736 | Rv3736 | 104A>T | 0 | 26 | 2 | 40 | 0,8706 | -0,0476 | 0,0000 |
| Rv3749c | Rv3749c | 81K>N | 0 | 26 | 2 | 40 | 0,8706 | -0,0476 | 0,0000 |
| Rv3791 | Rv3791 | 45D>N | 0 | 26 | 2 | 40 | 0,8706 | -0,0476 | 0,0000 |
| Rv3792 | Rv3792 | 43A>V | 0 | 26 | 2 | 40 | 0,8706 | -0,0476 | 0,0000 |
| Rv3795 | embB | 497Q>P | 0 | 26 | 2 | 40 | 0,8706 | -0,0476 | 0,0000 |
| Rv3807c | Rv3807c | 75P>L | 0 | 26 | 2 | 40 | 0,8706 | -0,0476 | 0,0000 |
| Rv3822 | Rv3822 | 270D>N | 0 | 26 | 2 | 40 | 0,8706 | -0,0476 | 0,0000 |
| Rv3824c | papA1 | 271Q>K | 0 | 26 | 2 | 40 | 0,8706 | -0,0476 | 0,0000 |
| Rv3850 | Rv3850 | 85R>P | 0 | 26 | 2 | 40 | 0,8706 | -0,0476 | 0,0000 |
| Rv3869 | Rv3869 | 79R>G | 0 | 26 | 2 | 40 | 0,8706 | -0,0476 | 0,0000 |
| Rv3897c | Rv3897c | 30Q>P | 0 | 26 | 2 | 40 | 0,8706 | -0,0476 | 0,0000 |
| Rv3899c | Rv3899c | 376A>P | 0 | 26 | 2 | 40 | 0,8706 | -0,0476 | 0,0000 |
| Rv3906c | Rv3906c | 10G>R | 0 | 26 | 2 | 40 | 0,8706 | -0,0476 | 0,0000 |
| Rv3910 | Rv3910 | 1114V>L | 0 | 26 | 2 | 40 | 0,8706 | -0,0476 | 0,0000 |
| Rv3913 | trxB2 | 9R>L | 0 | 26 | 2 | 40 | 0,8706 | -0,0476 | 0,0000 |
| Rv3918c | parA | 141P>R | 0 | 26 | 2 | 40 | 0,8706 | -0,0476 | 0,0000 |
| Rv3919c | gidB | 30G>R | 0 | 26 | 2 | 40 | 0,8706 | -0,0476 | 0,0000 |
| Rv3920c | Rv3920c | 162A>V | 0 | 26 | 2 | 40 | 0,8706 | -0,0476 | 0,0000 |
| Rv0092 | ctpA | 536R>S | 1 | 25 | 5 | 37 | 0,8726 | -0,0806 | 0,2960 |

| Rv0101 | nrp | 1280L>F | 1 | 25 | 5 | 37 | 0,8726 | -0,0806 | 0,2960 |
| --- | --- | --- | --- | --- | --- | --- | --- | --- | --- |
| Rv0204c | Rv0204c | 33R>C | 1 | 25 | 5 | 37 | 0,8726 | -0,0806 | 0,2960 |
| Rv0205 | Rv0205 | 51A>V | 1 | 25 | 5 | 37 | 0,8726 | -0,0806 | 0,2960 |
| Rv0206c | mmpL3 | 803P>R | 1 | 25 | 5 | 37 | 0,8726 | -0,0806 | 0,2960 |
| Rv0236c | Rv0236c | 326A>T | 1 | 25 | 5 | 37 | 0,8726 | -0,0806 | 0,2960 |
| Rv0237 | lpqI | 240V>A | 1 | 25 | 5 | 37 | 0,8726 | -0,0806 | 0,2960 |
| Rv0284 | Rv0284 | 732P>L | 1 | 25 | 5 | 37 | 0,8726 | -0,0806 | 0,2960 |
| Rv0376c | Rv0376c | 198M>T | 1 | 25 | 5 | 37 | 0,8726 | -0,0806 | 0,2960 |
| Rv0386 | Rv0386 | 896R>C | 1 | 25 | 5 | 37 | 0,8726 | -0,0806 | 0,2960 |
| Rv0425c | ctpH | 66N>T | 1 | 25 | 5 | 37 | 0,8726 | -0,0806 | 0,2960 |
| Rv0453 | PPE11 | 504M>V | 1 | 25 | 5 | 37 | 0,8726 | -0,0806 | 0,2960 |
| Rv0620 | galK | 119A>V | 1 | 25 | 5 | 37 | 0,8726 | -0,0806 | 0,2960 |
| Rv0721 | rpsE | 105V>A | 1 | 25 | 5 | 37 | 0,8726 | -0,0806 | 0,2960 |
| Rv0746 | PE_PGRS9 | 191E>G | 1 | 25 | 5 | 37 | 0,8726 | -0,0806 | 0,2960 |
| Rv0746 | PE_PGRS9 | 252T>A | 1 | 25 | 5 | 37 | 0,8726 | -0,0806 | 0,2960 |
| Rv0746 | PE_PGRS9 | 280N>D | 1 | 25 | 5 | 37 | 0,8726 | -0,0806 | 0,2960 |
| Rv0746 | PE_PGRS9 | 320T>A | 1 | 25 | 5 | 37 | 0,8726 | -0,0806 | 0,2960 |
| Rv0746 | PE_PGRS9 | 445T>A | 1 | 25 | 5 | 37 | 0,8726 | -0,0806 | 0,2960 |
| Rv0881 | Rv0881 | 247R>W | 1 | 25 | 5 | 37 | 0,8726 | -0,0806 | 0,2960 |
| Rv0882 | Rv0882 | 46G>C | 1 | 25 | 5 | 37 | 0,8726 | -0,0806 | 0,2960 |
| Rv0938 | Rv0938 | 516L>F | 1 | 25 | 5 | 37 | 0,8726 | -0,0806 | 0,2960 |
| Rv0976c | Rv0976c | 2R>C | 1 | 25 | 5 | 37 | 0,8726 | -0,0806 | 0,2960 |
| Rv0986 | Rv0986 | 32D>E | 1 | 25 | 5 | 37 | 0,8726 | -0,0806 | 0,2960 |
| Rv1161 | narG | 1000R>H | 1 | 25 | 5 | 37 | 0,8726 | -0,0806 | 0,2960 |
| Rv1198 | esxL | 20Q>L | 1 | 25 | 5 | 37 | 0,8726 | -0,0806 | 0,2960 |
| Rv1286 | cysN | 415L>S | 1 | 25 | 5 | 37 | 0,8726 | -0,0806 | 0,2960 |
| Rv1417 | Rv1417 | 102S>Q | 1 | 25 | 5 | 37 | 0,8726 | -0,0806 | 0,2960 |
| Rv1431 | Rv1431 | 172G>R | 1 | 25 | 5 | 37 | 0,8726 | -0,0806 | 0,2960 |
| Rv1446c | opcA | 116P>L | 1 | 25 | 5 | 37 | 0,8726 | -0,0806 | 0,2960 |
| Rv1518 | Rv1518 | 143Q>E | 1 | 25 | 5 | 37 | 0,8726 | -0,0806 | 0,2960 |
| Rv1599 | hisD | 75A>V | 1 | 25 | 5 | 37 | 0,8726 | -0,0806 | 0,2960 |
| Rv1614 | lgt | 29C>S | 1 | 25 | 5 | 37 | 0,8726 | -0,0806 | 0,2960 |
| Rv1633 | uvrB | 381R>H | 1 | 25 | 5 | 37 | 0,8726 | -0,0806 | 0,2960 |

| Rv1662 | pks8 | 1124M>V | 1 | 25 | 5 | 37 | 0,8726 | -0,0806 | 0,2960 |
| --- | --- | --- | --- | --- | --- | --- | --- | --- | --- |
| Rv1843c | guaB1 | 82A>T | 1 | 25 | 5 | 37 | 0,8726 | -0,0806 | 0,2960 |
| Rv1870c | Rv1870c | 123G>D | 1 | 25 | 5 | 37 | 0,8726 | -0,0806 | 0,2960 |
| Rv1933c | fadE18 | 196A>V | 1 | 25 | 5 | 37 | 0,8726 | -0,0806 | 0,2960 |
| Rv1939 | Rv1939 | 86A>V | 1 | 25 | 5 | 37 | 0,8726 | -0,0806 | 0,2960 |
| Rv1983 | PE_PGRS35 | 471P>S | 1 | 25 | 5 | 37 | 0,8726 | -0,0806 | 0,2960 |
| Rv2176 | pknL | 50M>I | 1 | 25 | 5 | 37 | 0,8726 | -0,0806 | 0,2960 |
| Rv2231c | cobC | 207D>G | 1 | 25 | 5 | 37 | 0,8726 | -0,0806 | 0,2960 |
| Rv2264c | Rv2264c | 250L>R | 1 | 25 | 5 | 37 | 0,8726 | -0,0806 | 0,2960 |
| Rv2265 | Rv2265 | 330V>I | 1 | 25 | 5 | 37 | 0,8726 | -0,0806 | 0,2960 |
| Rv2379c | mbtF | 792P>L | 1 | 25 | 5 | 37 | 0,8726 | -0,0806 | 0,2960 |
| Rv2398c | cysW | 236A>P | 1 | 25 | 5 | 37 | 0,8726 | -0,0806 | 0,2960 |
| Rv2424c | Rv2424c | 295H>Y | 1 | 25 | 5 | 37 | 0,8726 | -0,0806 | 0,2960 |
| Rv2637 | dedA | 147V>I | 1 | 25 | 5 | 37 | 0,8726 | -0,0806 | 0,2960 |
| Rv2690c | Rv2690c | 457V>A | 1 | 25 | 5 | 37 | 0,8726 | -0,0806 | 0,2960 |
| Rv2799 | Rv2799 | 31A>V | 1 | 25 | 5 | 37 | 0,8726 | -0,0806 | 0,2960 |
| Rv2886c | Rv2886c | 153A>V | 1 | 25 | 5 | 37 | 0,8726 | -0,0806 | 0,2960 |
| Rv2924c | fpg | 70A>V | 1 | 25 | 5 | 37 | 0,8726 | -0,0806 | 0,2960 |
| Rv2984 | ppk | 254F>L | 1 | 25 | 5 | 37 | 0,8726 | -0,0806 | 0,2960 |
| Rv2998 | Rv2998 | 116A>P | 1 | 25 | 5 | 37 | 0,8726 | -0,0806 | 0,2960 |
| Rv3085 | Rv3085 | 251V>L | 1 | 25 | 5 | 37 | 0,8726 | -0,0806 | 0,2960 |
| Rv3130c | tgs1 | 237P>A | 1 | 25 | 5 | 37 | 0,8726 | -0,0806 | 0,2960 |
| Rv3195 | Rv3195 | 143D>N | 1 | 25 | 5 | 37 | 0,8726 | -0,0806 | 0,2960 |
| Rv3224A | Rv3224A | 28I>S | 1 | 25 | 5 | 37 | 0,8726 | -0,0806 | 0,2960 |
| Rv3302c | glpD2 | 167G>S | 1 | 25 | 5 | 37 | 0,8726 | -0,0806 | 0,2960 |
| Rv3359 | Rv3359 | 74G>C | 1 | 25 | 5 | 37 | 0,8726 | -0,0806 | 0,2960 |
| Rv3375 | amiD | 74A>S | 1 | 25 | 5 | 37 | 0,8726 | -0,0806 | 0,2960 |
| Rv3478 | PPE60 | 67A>V | 1 | 25 | 5 | 37 | 0,8726 | -0,0806 | 0,2960 |
| Rv3531c | Rv3531c | 238R>Q | 1 | 25 | 5 | 37 | 0,8726 | -0,0806 | 0,2960 |
| Rv3554 | fdxB | 658R>P | 1 | 25 | 5 | 37 | 0,8726 | -0,0806 | 0,2960 |
| Rv3591c | Rv3591c | 30S>F | 1 | 25 | 5 | 37 | 0,8726 | -0,0806 | 0,2960 |
| Rv3698 | Rv3698 | 182D>Y | 1 | 25 | 5 | 37 | 0,8726 | -0,0806 | 0,2960 |
| Rv3705A | Rv3705A | 15A>P | 1 | 25 | 5 | 37 | 0,8726 | -0,0806 | 0,2960 |

| Rv3705c | Rv3705c | 145D>A | 1 | 25 | 5 | 37 | 0,8726 | -0,0806 | 0,2960 |
| --- | --- | --- | --- | --- | --- | --- | --- | --- | --- |
| Rv3825c | pks2 | 605T>A | 1 | 25 | 5 | 37 | 0,8726 | -0,0806 | 0,2960 |
| Rv3871 | Rv3871 | 288P>S | 1 | 25 | 5 | 37 | 0,8726 | -0,0806 | 0,2960 |
| Rv3881c | Rv3881c | 255Q>P | 1 | 25 | 5 | 37 | 0,8726 | -0,0806 | 0,2960 |
| Rv3903c | Rv3903c | 411D>N | 1 | 25 | 5 | 37 | 0,8726 | -0,0806 | 0,2960 |
| Rv4008 | Rv4008 | 37H>Y | 1 | 25 | 5 | 37 | 0,8726 | -0,0806 | 0,2960 |
| Rv2627c | Rv2627c | 104R>G | 25 | 1 | 42 | 0 | 0,8998 | -0,0385 | 0,0000 |
| Rv3428c | Rv3428c | 327S>C | 8 | 18 | 20 | 22 | 0,9150 | -0,1685 | 0,4889 |
| Rv0180c | Rv0180c | 204A>P | 0 | 26 | 3 | 39 | 0,9183 | -0,0714 | 0,0000 |
| Rv0180c | Rv0180c | 3Q>* | 0 | 26 | 3 | 39 | 0,9183 | -0,0714 | 0,0000 |
| Rv0197 | Rv0197 | 44Y>C | 0 | 26 | 3 | 39 | 0,9183 | -0,0714 | 0,0000 |
| Rv0731c | Rv0731c | 93Y>C | 0 | 26 | 3 | 39 | 0,9183 | -0,0714 | 0,0000 |
| Rv0846c | Rv0846c | 448M>T | 0 | 26 | 3 | 39 | 0,9183 | -0,0714 | 0,0000 |
| Rv0971c | echA7 | 47E>V | 0 | 26 | 3 | 39 | 0,9183 | -0,0714 | 0,0000 |
| Rv1067c | PE_PGRS19 | 111L>W | 0 | 26 | 3 | 39 | 0,9183 | -0,0714 | 0,0000 |
| Rv1091 | PE_PGRS22 | 757G>C | 0 | 26 | 3 | 39 | 0,9183 | -0,0714 | 0,0000 |
| Rv1098c | fumC | 117A>T | 0 | 26 | 3 | 39 | 0,9183 | -0,0714 | 0,0000 |
| Rv1145 | mmpL13a | 217S>P | 0 | 26 | 3 | 39 | 0,9183 | -0,0714 | 0,0000 |
| Rv1199c | Rv1199c | 9T>A | 0 | 26 | 3 | 39 | 0,9183 | -0,0714 | 0,0000 |
| Rv1215c | Rv1215c | 468P>L | 0 | 26 | 3 | 39 | 0,9183 | -0,0714 | 0,0000 |
| Rv1935c | echA13 | 127W>* | 0 | 26 | 3 | 39 | 0,9183 | -0,0714 | 0,0000 |
| Rv2041c | Rv2041c | 370H>Y | 0 | 26 | 3 | 39 | 0,9183 | -0,0714 | 0,0000 |
| Rv2042c | Rv2042c | 74I>V | 0 | 26 | 3 | 39 | 0,9183 | -0,0714 | 0,0000 |
| Rv2234 | ptpA | 37A>T | 0 | 26 | 3 | 39 | 0,9183 | -0,0714 | 0,0000 |
| Rv2628 | Rv2628 | 2S>P | 0 | 26 | 3 | 39 | 0,9183 | -0,0714 | 0,0000 |
| Rv2702 | ppgK | 93A>S | 0 | 26 | 3 | 39 | 0,9183 | -0,0714 | 0,0000 |
| Rv2881c | cdsA | 301V>I | 0 | 26 | 3 | 39 | 0,9183 | -0,0714 | 0,0000 |
| Rv3411c | guaB2 | 38H>D | 0 | 26 | 3 | 39 | 0,9183 | -0,0714 | 0,0000 |
| Rv3424c | Rv3424c | 36K>Q | 0 | 26 | 3 | 39 | 0,9183 | -0,0714 | 0,0000 |
| Rv3511 | PE_PGRS55 | 429G>S | 0 | 26 | 3 | 39 | 0,9183 | -0,0714 | 0,0000 |
| Rv3737 | Rv3737 | 108I>V | 0 | 26 | 3 | 39 | 0,9183 | -0,0714 | 0,0000 |
| Rv3820c | papA2 | 419V>G | 0 | 26 | 3 | 39 | 0,9183 | -0,0714 | 0,0000 |
| Rv3467 | Rv3467 | 315K>E | 20 | 6 | 38 | 4 | 0,9374 | -0,1355 | 0,3509 |

| Rv1361c | PPE19 | 55T>V | 0 | 26 | 4 | 38 | 0,9476 | -0,0952 | 0,0000 |
| --- | --- | --- | --- | --- | --- | --- | --- | --- | --- |
| Rv2512c | Rv2512c | 9T>A | 0 | 26 | 4 | 38 | 0,9476 | -0,0952 | 0,0000 |
| Rv1917c | PPE34 | 676S>A | 24 | 2 | 42 | 0 | 0,9660 | -0,0769 | 0,0000 |
| Rv2037c | Rv2037c | 312C>Y | 24 | 2 | 42 | 0 | 0,9660 | -0,0769 | 0,0000 |
| Rv2039c | Rv2039c | 131V>F | 24 | 2 | 42 | 0 | 0,9660 | -0,0769 | 0,0000 |
| Rv0336 | Rv0336 | 496P>H | 0 | 26 | 5 | 37 | 0,9662 | -0,1190 | 0,0000 |
| Rv3425 | PPE57 | 128T>A | 3 | 23 | 13 | 29 | 0,9667 | -0,1941 | 0,2910 |
