# Supplementary material for: A multiple genome analysis of Mycobacterium tuberculosis reveals specific novel genes and mutations associated with pyrazinamide resistance
Source: BMC Genomics. 2017 Oct 11;18:769. doi: 10.1186/s12864-017-4146-z (PMC5637355; doi:10.1186/s12864-017-4146-z)
Supplement: Supplementary file 3 — Complete results of the analysis by gene, showing for each gene the number of resistant strains with at least one mutation (M-Res), without mutation (NM-Res) and analogously for the susceptible strains (M-Sus and NM-Sus, respectively). The P-value of the Proportion Test, the Youden Index and Odds Ratio are also included. (DOCX 610 kb) [file 12864_2017_4146_MOESM3_ESM.docx]

Additional file 3: Table 3. Complete results of the analysis by gene, showing for each gene the number of resistant strains with at least one mutation (M-Res), without mutation (NM-Res) and analogously for the susceptible strains (M-Sus and NM-Sus, respectively). The P-value of the Proportion Test, the Youden Index and Odds Ratio are also included.

| **Synonym** | **Gen Name** | **M-Res** | **NM-Res** | **M-Sus** | **NM-Sus** | **P-value (Proportion test)** | **Youden index** | **Odds Ratio** |
| --- | --- | --- | --- | --- | --- | --- | --- | --- |
| Rv2043c  Rv0667  Rv3795  Rv1908c  Rv2853  Rv1313c Rv2505c Rv0280  Rv3236c Rv0278c Rv1556  Rv1630  Rv2718c Rv2948c Rv3242c Rv1396c Rv2052c Rv0532  Rv1753c  Rv3511  Rv0642c Rv0948c Rv1569  Rv1884c Rv0095c Rv0747  Rv0107c  Rv1872c | pncA rpoB embB katG  PE_PGRS48  Rv1313c fadD35  PPE3  Rv3236c  PE_PGRS3  Rv1556 rpsA nrdR fadD22  Rv3242c  PE_PGRS25  Rv2052c  PE_PGRS6  PPE24  PE_PGRS55 mmaA4  Rv0948c bioF1 rpfC Rv0095c  PE_PGRS10 ctpI lldD2 | 17  23  16  22  23  19  5  22  11  18  3  3  3  3  3  19  7  23  17  21  12  12  12  12  15  15  10  10 | 9  3  10  4  3  7  21  4  15  8  23  23  23  23  23  7  19  3  9  5  14  14  14  14  11  11  16  16 | 4  14  7  21  24  17  1  24  7  17  0  0  0  0  0  19  3  27  16  23  12  12  12  12  17  17  9  9 | 38  28  35  21  18  25  41  18  35  25  42  42  42  42  42  23  39  15  26  19  30  30  30  30  25  25  33  33 | 0.0000  0.0000  0.0001  0.0020  0.0033  0.0044  0.0086  0.0093  0.0099  0.0106  0.0122  0.0122  0.0122  0.0122  0.0122  0.0123  0.0126  0.0140  0.0143  0.0146  0.0702  0.0702  0.0702  0.0702  0.0835  0.0835  0.0641  0.0641 | 0.5586  0.5513  0.4487  0.3462  0.3132  0.3260  0.1685  0.2747  0.2564  0.2875  0.1154  0.1154  0.1154  0.1154  0.1154  0.2784  0.1978  0.2418  0.2729  0.2601  0.1758  0.1758  0.1758  0.1758  0.1722  0.1722  0.1703  0.1703 | 17.9444  15.3333  8.0000  5.5000  5.7500  3.9916  9.7619  4.1250  3.6667  3.3088  ND ND ND ND ND  3.2857  4.7895  4.2593  3.0694  3.4696  2.1429  2.1429  2.1429  2.1429  2.0053  2.0053  2.2917  2.2917 |

| Rv3508 | PE_PGRS54 | 11 | 15 | 8 | 34 | 0.0189 | 0.2326 | 3.1167 |
| --- | --- | --- | --- | --- | --- | --- | --- | --- |
| Rv0958 | Rv0958 | 26 | 0 | 36 | 6 | 0.0218 | 0.1429 | ND |
| Rv2543 | lppA | 9 | 17 | 8 | 34 | 0.0748 | 0.1557 | 2.2500 |
| Rv3888c | Rv3888c | 9 | 17 | 8 | 34 | 0.0748 | 0.1557 | 2.2500 |
| Rv1606 | hisI | 12 | 14 | 13 | 29 | 0.1032 | 0.1520 | 1.9121 |
| Rv2716 | Rv2716 | 12 | 14 | 13 | 29 | 0.1032 | 0.1520 | 1.9121 |
| Rv2177c | Rv2177c | 7 | 19 | 5 | 37 | 0.0572 | 0.1502 | 2.7263 |
| Rv3512 | PE_PGRS56 | 7 | 19 | 5 | 37 | 0.0572 | 0.1502 | 2.7263 |
| Rv0631c | recC | 15 | 11 | 18 | 24 | 0.1171 | 0.1484 | 1.8182 |
| Rv0850 | Rv0850 | 15 | 11 | 18 | 24 | 0.1171 | 0.1484 | 1.8182 |
| Rv2764c | thyA | 15 | 11 | 18 | 24 | 0.1171 | 0.1484 | 1.8182 |
| Rv1319c | Rv1319c | 23 | 3 | 31 | 11 | 0.0732 | 0.1465 | 2.7204 |
| Rv2078 | Rv2078 | 26 | 0 | 36 | 6 | 0.0218 | 0.1429 | ND |
| Rv0297 | PE_PGRS5 | 4 | 22 | 1 | 41 | 0.0229 | 0.1300 | 7.4545 |
| Rv0739 | Rv0739 | 4 | 22 | 1 | 41 | 0.0229 | 0.1300 | 7.4545 |
| Rv2487c | PE_PGRS42 | 4 | 22 | 1 | 41 | 0.0229 | 0.1300 | 7.4545 |
| Rv2777c | Rv2777c | 4 | 22 | 1 | 41 | 0.0229 | 0.1300 | 7.4545 |
| Rv3297 | nei | 4 | 22 | 1 | 41 | 0.0229 | 0.1300 | 7.4545 |
| Rv1148c | Rv1148c | 8 | 18 | 7 | 35 | 0.0864 | 0.1410 | 2.2222 |
| Rv2561 | Rv2561 | 16 | 10 | 20 | 22 | 0.1319 | 0.1392 | 1.7600 |
| Rv3864 | Rv3864 | 16 | 10 | 20 | 22 | 0.1319 | 0.1392 | 1.7600 |
| Rv0853c | pdc | 6 | 20 | 4 | 38 | 0.0626 | 0.1355 | 2.8500 |
| Rv0987 | Rv0987 | 6 | 20 | 4 | 38 | 0.0626 | 0.1355 | 2.8500 |
| Rv1450c | PE_PGRS27 | 6 | 20 | 4 | 38 | 0.0626 | 0.1355 | 2.8500 |
| Rv3463 | Rv3463 | 6 | 20 | 4 | 38 | 0.0626 | 0.1355 | 2.8500 |
| Rv0015c | pknA | 14 | 12 | 17 | 25 | 0.1410 | 0.1337 | 1.7157 |
| Rv3720 | Rv3720 | 14 | 12 | 17 | 25 | 0.1410 | 0.1337 | 1.7157 |
| Rv2560 | Rv2560 | 25 | 1 | 33 | 9 | 0.0233 | 0.1758 | 6.8182 |
| Rv2935 | ppsE | 8 | 18 | 5 | 37 | 0.0273 | 0.1886 | 3.2889 |
| Rv1937 | Rv1937 | 5 | 21 | 2 | 40 | 0.0282 | 0.1447 | 4.7619 |
| Rv2783c | gpsI | 5 | 21 | 2 | 40 | 0.0282 | 0.1447 | 4.7619 |
| Rv3507 | PE_PGRS53 | 5 | 21 | 2 | 40 | 0.0282 | 0.1447 | 4.7619 |
| Rv2022c | Rv2022c | 12 | 14 | 14 | 28 | 0.1452 | 0.1282 | 1.7143 |
| Rv2897c | Rv2897c | 7 | 19 | 6 | 36 | 0.0989 | 0.1264 | 2.2105 |

| Rv0797 | Rv0797 | 15 | 11 | 19 | 23 | 0.1591 | 0.1245 | 1.6507 |
| --- | --- | --- | --- | --- | --- | --- | --- | --- |
| Rv3786c | Rv3786c | 15 | 11 | 19 | 23 | 0.1591 | 0.1245 | 1.6507 |
| Rv0572c | Rv0572c | 23 | 3 | 32 | 10 | 0.1056 | 0.1227 | 2.3958 |
| Rv3190c | Rv3190c | 23 | 3 | 32 | 10 | 0.1056 | 0.1227 | 2.3958 |
| Rv3823c | mmpL8 | 10 | 16 | 11 | 31 | 0.1436 | 0.1227 | 1.7614 |
| Rv0149 | Rv0149 | 5 | 21 | 3 | 39 | 0.0664 | 0.1209 | 3.0952 |
| Rv0399c | lpqK | 5 | 21 | 3 | 39 | 0.0664 | 0.1209 | 3.0952 |
| Rv0466 | Rv0466 | 5 | 21 | 3 | 39 | 0.0664 | 0.1209 | 3.0952 |
| Rv0574c | Rv0574c | 5 | 21 | 3 | 39 | 0.0664 | 0.1209 | 3.0952 |
| Rv0682 | rpsL | 5 | 21 | 3 | 39 | 0.0664 | 0.1209 | 3.0952 |
| Rv1288 | Rv1288 | 5 | 21 | 3 | 39 | 0.0664 | 0.1209 | 3.0952 |
| Rv1500 | Rv1500 | 5 | 21 | 3 | 39 | 0.0664 | 0.1209 | 3.0952 |
| Rv1547 | dnaE | 5 | 21 | 3 | 39 | 0.0664 | 0.1209 | 3.0952 |
| Rv2008c | Rv2008c | 5 | 21 | 3 | 39 | 0.0664 | 0.1209 | 3.0952 |
| Rv2326c | Rv2326c | 5 | 21 | 3 | 39 | 0.0664 | 0.1209 | 3.0952 |
| Rv2570 | Rv2570 | 5 | 21 | 3 | 39 | 0.0664 | 0.1209 | 3.0952 |
| Rv2617c | Rv2617c | 5 | 21 | 3 | 39 | 0.0664 | 0.1209 | 3.0952 |
| Rv2959c | Rv2959c | 5 | 21 | 3 | 39 | 0.0664 | 0.1209 | 3.0952 |
| Rv3239c | Rv3239c | 5 | 21 | 3 | 39 | 0.0664 | 0.1209 | 3.0952 |
| Rv3558 | PPE64 | 5 | 21 | 3 | 39 | 0.0664 | 0.1209 | 3.0952 |
| Rv3838c | pheA | 5 | 21 | 3 | 39 | 0.0664 | 0.1209 | 3.0952 |
| Rv3869 | Rv3869 | 5 | 21 | 3 | 39 | 0.0664 | 0.1209 | 3.0952 |
| Rv0172 | mce1D | 18 | 8 | 24 | 18 | 0.1594 | 0.1209 | 1.6875 |
| Rv3659c | Rv3659c | 5 | 21 | 2 | 40 | 0.0282 | 0.1447 | 4.7619 |
| Rv2802c | Rv2802c | 6 | 20 | 3 | 39 | 0.0298 | 0.1593 | 3.9000 |
| Rv0218 | Rv0218 | 26 | 0 | 37 | 5 | 0.0338 | 0.1190 | ND |
| Rv0545c | pitA | 26 | 0 | 37 | 5 | 0.0338 | 0.1190 | ND |
| Rv0785 | Rv0785 | 26 | 0 | 37 | 5 | 0.0338 | 0.1190 | ND |
| Rv0938 | Rv0938 | 26 | 0 | 37 | 5 | 0.0338 | 0.1190 | ND |
| Rv0974c | accD2 | 26 | 0 | 37 | 5 | 0.0338 | 0.1190 | ND |
| Rv1394c | cyp132 | 26 | 0 | 37 | 5 | 0.0338 | 0.1190 | ND |
| Rv1449c | tkt | 26 | 0 | 37 | 5 | 0.0338 | 0.1190 | ND |
| Rv1604 | impA | 26 | 0 | 37 | 5 | 0.0338 | 0.1190 | ND |
| Rv1644 | tsnR | 26 | 0 | 37 | 5 | 0.0338 | 0.1190 | ND |

| Rv1900c | lipJ | 26 | 0 | 37 | 5 | 0.0338 | 0.1190 | ND |
| --- | --- | --- | --- | --- | --- | --- | --- | --- |
| Rv2072c | cobL | 26 | 0 | 37 | 5 | 0.0338 | 0.1190 | ND |
| Rv2236c | cobD | 26 | 0 | 37 | 5 | 0.0338 | 0.1190 | ND |
| Rv2379c | mbtF | 26 | 0 | 37 | 5 | 0.0338 | 0.1190 | ND |
| Rv2398c | cysW | 26 | 0 | 37 | 5 | 0.0338 | 0.1190 | ND |
| Rv2691 | ceoB | 26 | 0 | 37 | 5 | 0.0338 | 0.1190 | ND |
| Rv2729c | Rv2729c | 26 | 0 | 37 | 5 | 0.0338 | 0.1190 | ND |
| Rv3383c | idsB | 26 | 0 | 37 | 5 | 0.0338 | 0.1190 | ND |
| Rv3468c | Rv3468c | 26 | 0 | 37 | 5 | 0.0338 | 0.1190 | ND |
| Rv3521 | Rv3521 | 26 | 0 | 37 | 5 | 0.0338 | 0.1190 | ND |
| Rv0244c | fadE5 | 8 | 18 | 8 | 34 | 0.1341 | 0.1172 | 1.8889 |
| Rv3630 | Rv3630 | 26 | 0 | 37 | 5 | 0.0338 | 0.1190 | ND |
| Rv3731 | ligC | 26 | 0 | 37 | 5 | 0.0338 | 0.1190 | ND |
| Rv0002 | dnaN | 2 | 24 | 0 | 42 | 0.0340 | 0.0769 | ND |
| Rv0074 | Rv0074 | 2 | 24 | 0 | 42 | 0.0340 | 0.0769 | ND |
| Rv0161 | Rv0161 | 2 | 24 | 0 | 42 | 0.0340 | 0.0769 | ND |
| Rv0493c | Rv0493c | 16 | 10 | 21 | 21 | 0.1766 | 0.1154 | 1.6000 |
| Rv3824c | papA1 | 16 | 10 | 21 | 21 | 0.1766 | 0.1154 | 1.6000 |
| Rv1650 | pheT | 11 | 15 | 13 | 29 | 0.1705 | 0.1136 | 1.6359 |
| Rv2994 | Rv2994 | 11 | 15 | 13 | 29 | 0.1705 | 0.1136 | 1.6359 |
| Rv3021c | PPE47 | 19 | 7 | 26 | 16 | 0.1720 | 0.1117 | 1.6703 |
| Rv1774 | Rv1774 | 4 | 22 | 2 | 40 | 0.0667 | 0.1062 | 3.6364 |
| Rv2425c | Rv2425c | 4 | 22 | 2 | 40 | 0.0667 | 0.1062 | 3.6364 |
| Rv2934 | ppsD | 4 | 22 | 2 | 40 | 0.0667 | 0.1062 | 3.6364 |
| Rv3922c | Rv3922c | 4 | 22 | 2 | 40 | 0.0667 | 0.1062 | 3.6364 |
| Rv0355c | PPE8 | 25 | 1 | 36 | 6 | 0.0843 | 0.1044 | 4.1667 |
| Rv1155 | Rv1155 | 7 | 19 | 7 | 35 | 0.1547 | 0.1026 | 1.8421 |
| Rv1047 | Rv1047 | 20 | 6 | 28 | 14 | 0.1835 | 0.1026 | 1.6667 |
| Rv3023c | Rv3023c | 20 | 6 | 28 | 14 | 0.1835 | 0.1026 | 1.6667 |
| Rv3115 | Rv3115 | 20 | 6 | 28 | 14 | 0.1835 | 0.1026 | 1.6667 |
| Rv1945 | Rv1945 | 10 | 16 | 12 | 30 | 0.1984 | 0.0989 | 1.5625 |
| Rv3506 | fadD17 | 10 | 16 | 12 | 30 | 0.1984 | 0.0989 | 1.5625 |
| Rv0406c | pks6 | 23 | 3 | 33 | 9 | 0.1493 | 0.0989 | 2.0909 |
| Rv0538 | Rv0538 | 23 | 3 | 33 | 9 | 0.1493 | 0.0989 | 2.0909 |

| Rv0727c | fucA | 23 | 3 | 33 | 9 | 0.1493 | 0.0989 | 2.0909 |
| --- | --- | --- | --- | --- | --- | --- | --- | --- |
| Rv1446c | opcA | 23 | 3 | 33 | 9 | 0.1493 | 0.0989 | 2.0909 |
| Rv1459c | Rv1459c | 23 | 3 | 33 | 9 | 0.1493 | 0.0989 | 2.0909 |
| Rv1486c | Rv1486c | 23 | 3 | 33 | 9 | 0.1493 | 0.0989 | 2.0909 |
| Rv1860 | apa | 23 | 3 | 33 | 9 | 0.1493 | 0.0989 | 2.0909 |
| Rv2090 | Rv2090 | 23 | 3 | 33 | 9 | 0.1493 | 0.0989 | 2.0909 |
| Rv2178c | aroG | 23 | 3 | 33 | 9 | 0.1493 | 0.0989 | 2.0909 |
| Rv2688c | Rv2688c | 23 | 3 | 33 | 9 | 0.1493 | 0.0989 | 2.0909 |
| Rv3245c | mtrB | 23 | 3 | 33 | 9 | 0.1493 | 0.0989 | 2.0909 |
| Rv3329 | Rv3329 | 23 | 3 | 33 | 9 | 0.1493 | 0.0989 | 2.0909 |
| Rv3833 | Rv3833 | 23 | 3 | 33 | 9 | 0.1493 | 0.0989 | 2.0909 |
| Rv0638 | secE | 5 | 21 | 4 | 38 | 0.1255 | 0.0971 | 2.2619 |
| Rv0977 | PE_PGRS16 | 5 | 21 | 4 | 38 | 0.1255 | 0.0971 | 2.2619 |
| Rv1204c | Rv1204c | 5 | 21 | 4 | 38 | 0.1255 | 0.0971 | 2.2619 |
| Rv1508c | Rv1508c | 5 | 21 | 4 | 38 | 0.1255 | 0.0971 | 2.2619 |
| Rv2252 | Rv2252 | 5 | 21 | 4 | 38 | 0.1255 | 0.0971 | 2.2619 |
| Rv2790c | ltp1 | 5 | 21 | 4 | 38 | 0.1255 | 0.0971 | 2.2619 |
| Rv3854c | ethA | 5 | 21 | 4 | 38 | 0.1255 | 0.0971 | 2.2619 |
| Rv0109 | PE_PGRS1 | 26 | 0 | 38 | 4 | 0.0524 | 0.0952 | ND |
| Rv0989c | grcC2 | 26 | 0 | 38 | 4 | 0.0524 | 0.0952 | ND |
| Rv2566 | Rv2566 | 26 | 0 | 38 | 4 | 0.0524 | 0.0952 | ND |
| Rv2825c | Rv2825c | 26 | 0 | 38 | 4 | 0.0524 | 0.0952 | ND |
| Rv2981c | ddl | 26 | 0 | 38 | 4 | 0.0524 | 0.0952 | ND |
| Rv3042c | serB2 | 26 | 0 | 38 | 4 | 0.0524 | 0.0952 | ND |
| Rv0030 | Rv0030 | 13 | 13 | 17 | 25 | 0.2211 | 0.0952 | 1.4706 |
| Rv0059 | Rv0059 | 13 | 13 | 17 | 25 | 0.2211 | 0.0952 | 1.4706 |
| Rv0134 | ephF | 13 | 13 | 17 | 25 | 0.2211 | 0.0952 | 1.4706 |
| Rv0152c | PE2 | 13 | 13 | 17 | 25 | 0.2211 | 0.0952 | 1.4706 |
| Rv0181c | Rv0181c | 13 | 13 | 17 | 25 | 0.2211 | 0.0952 | 1.4706 |
| Rv0264c | Rv0264c | 13 | 13 | 17 | 25 | 0.2211 | 0.0952 | 1.4706 |
| Rv0366c | Rv0366c | 13 | 13 | 17 | 25 | 0.2211 | 0.0952 | 1.4706 |
| Rv0446c | Rv0446c | 13 | 13 | 17 | 25 | 0.2211 | 0.0952 | 1.4706 |
| Rv0472c | Rv0472c | 13 | 13 | 17 | 25 | 0.2211 | 0.0952 | 1.4706 |
| Rv0655 | mkl | 13 | 13 | 17 | 25 | 0.2211 | 0.0952 | 1.4706 |

| Rv1075c | Rv1075c | 13 | 13 | 17 | 25 | 0.2211 | 0.0952 | 1.4706 |
| --- | --- | --- | --- | --- | --- | --- | --- | --- |
| Rv1278 | Rv1278 | 13 | 13 | 17 | 25 | 0.2211 | 0.0952 | 1.4706 |
| Rv1292 | argS | 13 | 13 | 17 | 25 | 0.2211 | 0.0952 | 1.4706 |
| Rv1347c | Rv1347c | 13 | 13 | 17 | 25 | 0.2211 | 0.0952 | 1.4706 |
| Rv1537 | dinX | 13 | 13 | 17 | 25 | 0.2211 | 0.0952 | 1.4706 |
| Rv1674c | Rv1674c | 13 | 13 | 17 | 25 | 0.2211 | 0.0952 | 1.4706 |
| Rv1987 | Rv1987 | 13 | 13 | 17 | 25 | 0.2211 | 0.0952 | 1.4706 |
| Rv2438c | nadE | 13 | 13 | 17 | 25 | 0.2211 | 0.0952 | 1.4706 |
| Rv2446c | Rv2446c | 13 | 13 | 17 | 25 | 0.2211 | 0.0952 | 1.4706 |
| Rv2768c | PPE43 | 13 | 13 | 17 | 25 | 0.2211 | 0.0952 | 1.4706 |
| Rv2922c | smc | 13 | 13 | 17 | 25 | 0.2211 | 0.0952 | 1.4706 |
| Rv3179 | Rv3179 | 13 | 13 | 17 | 25 | 0.2211 | 0.0952 | 1.4706 |
| Rv3217c | Rv3217c | 13 | 13 | 17 | 25 | 0.2211 | 0.0952 | 1.4706 |
| Rv3234c | Rv3234c | 13 | 13 | 17 | 25 | 0.2211 | 0.0952 | 1.4706 |
| Rv3439c | Rv3439c | 13 | 13 | 17 | 25 | 0.2211 | 0.0952 | 1.4706 |
| Rv3544c | fadE28 | 13 | 13 | 17 | 25 | 0.2211 | 0.0952 | 1.4706 |
| Rv3693 | Rv3693 | 13 | 13 | 17 | 25 | 0.2211 | 0.0952 | 1.4706 |
| Rv3775 | lipE | 13 | 13 | 17 | 25 | 0.2211 | 0.0952 | 1.4706 |
| Rv0143c | Rv0143c | 8 | 18 | 9 | 33 | 0.1937 | 0.0934 | 1.6296 |
| Rv3727 | Rv3727 | 16 | 10 | 22 | 20 | 0.2299 | 0.0916 | 1.4545 |
| Rv0167 | yrbE1A | 3 | 23 | 1 | 41 | 0.0594 | 0.0916 | 5.3478 |
| Rv0327c | cyp135A1 | 3 | 23 | 1 | 41 | 0.0594 | 0.0916 | 5.3478 |
| Rv0370c | Rv0370c | 3 | 23 | 1 | 41 | 0.0594 | 0.0916 | 5.3478 |
| Rv0423c | thiC | 3 | 23 | 1 | 41 | 0.0594 | 0.0916 | 5.3478 |
| Rv0698 | Rv0698 | 3 | 23 | 1 | 41 | 0.0594 | 0.0916 | 5.3478 |
| Rv1092c | coaA | 3 | 23 | 1 | 41 | 0.0594 | 0.0916 | 5.3478 |
| Rv1126c | Rv1126c | 3 | 23 | 1 | 41 | 0.0594 | 0.0916 | 5.3478 |
| Rv1187 | rocA | 3 | 23 | 1 | 41 | 0.0594 | 0.0916 | 5.3478 |
| Rv1207 | folP2 | 3 | 23 | 1 | 41 | 0.0594 | 0.0916 | 5.3478 |
| Rv1221 | sigE | 3 | 23 | 1 | 41 | 0.0594 | 0.0916 | 5.3478 |
| Rv1231c | Rv1231c | 3 | 23 | 1 | 41 | 0.0594 | 0.0916 | 5.3478 |
| Rv1255c | Rv1255c | 3 | 23 | 1 | 41 | 0.0594 | 0.0916 | 5.3478 |
| Rv1438 | tpiA | 3 | 23 | 1 | 41 | 0.0594 | 0.0916 | 5.3478 |
| Rv1749c | Rv1749c | 3 | 23 | 1 | 41 | 0.0594 | 0.0916 | 5.3478 |

| Rv1848 | ureA | 3 | 23 | 1 | 41 | 0.0594 | 0.0916 | 5.3478 |
| --- | --- | --- | --- | --- | --- | --- | --- | --- |
| Rv1883c | Rv1883c | 3 | 23 | 1 | 41 | 0.0594 | 0.0916 | 5.3478 |
| Rv1965 | yrbE3B | 3 | 23 | 1 | 41 | 0.0594 | 0.0916 | 5.3478 |
| Rv1967 | mce3B | 3 | 23 | 1 | 41 | 0.0594 | 0.0916 | 5.3478 |
| Rv1991c | Rv1991c | 3 | 23 | 1 | 41 | 0.0594 | 0.0916 | 5.3478 |
| Rv1998c | Rv1998c | 3 | 23 | 1 | 41 | 0.0594 | 0.0916 | 5.3478 |
| Rv2019 | Rv2019 | 3 | 23 | 1 | 41 | 0.0594 | 0.0916 | 5.3478 |
| Rv2021c | Rv2021c | 3 | 23 | 1 | 41 | 0.0594 | 0.0916 | 5.3478 |
| Rv2046 | lppI | 3 | 23 | 1 | 41 | 0.0594 | 0.0916 | 5.3478 |
| Rv2483c | plsC | 3 | 23 | 1 | 41 | 0.0594 | 0.0916 | 5.3478 |
| Rv2813 | Rv2813 | 3 | 23 | 1 | 41 | 0.0594 | 0.0916 | 5.3478 |
| Rv3061c | fadE22 | 3 | 23 | 1 | 41 | 0.0594 | 0.0916 | 5.3478 |
| Rv3090 | Rv3090 | 3 | 23 | 1 | 41 | 0.0594 | 0.0916 | 5.3478 |
| Rv3104c | Rv3104c | 3 | 23 | 1 | 41 | 0.0594 | 0.0916 | 5.3478 |
| Rv3341 | metX | 3 | 23 | 1 | 41 | 0.0594 | 0.0916 | 5.3478 |
| Rv3431c | Rv3431c | 3 | 23 | 1 | 41 | 0.0594 | 0.0916 | 5.3478 |
| Rv3454 | Rv3454 | 3 | 23 | 1 | 41 | 0.0594 | 0.0916 | 5.3478 |
| Rv3573c | fadE34 | 3 | 23 | 1 | 41 | 0.0594 | 0.0916 | 5.3478 |
| Rv3579c | Rv3579c | 3 | 23 | 1 | 41 | 0.0594 | 0.0916 | 5.3478 |
| Rv3664c | dppC | 3 | 23 | 1 | 41 | 0.0594 | 0.0916 | 5.3478 |
| Rv3746c | PE34 | 3 | 23 | 1 | 41 | 0.0594 | 0.0916 | 5.3478 |
| Rv3860 | Rv3860 | 3 | 23 | 1 | 41 | 0.0594 | 0.0916 | 5.3478 |
| Rv1355c | moeY | 6 | 20 | 6 | 36 | 0.1777 | 0.0879 | 1.8000 |
| Rv2339 | mmpL9 | 6 | 20 | 6 | 36 | 0.1777 | 0.0879 | 1.8000 |
| Rv3057c | Rv3057c | 19 | 7 | 27 | 15 | 0.2257 | 0.0879 | 1.5079 |
| Rv0791c | Rv0791c | 14 | 12 | 19 | 23 | 0.2450 | 0.0861 | 1.4123 |
| Rv3657c | Rv3657c | 9 | 17 | 11 | 31 | 0.2294 | 0.0842 | 1.4920 |
| Rv3798 | Rv3798 | 9 | 17 | 11 | 31 | 0.2294 | 0.0842 | 1.4920 |
| Rv0021c | Rv0021c | 4 | 22 | 3 | 39 | 0.1386 | 0.0824 | 2.3636 |
| Rv0584 | Rv0584 | 4 | 22 | 3 | 39 | 0.1386 | 0.0824 | 2.3636 |
| Rv0940c | Rv0940c | 4 | 22 | 3 | 39 | 0.1386 | 0.0824 | 2.3636 |
| Rv1429 | Rv1429 | 4 | 22 | 3 | 39 | 0.1386 | 0.0824 | 2.3636 |
| Rv2351c | plcA | 4 | 22 | 3 | 39 | 0.1386 | 0.0824 | 2.3636 |
| Rv2368c | phoH1 | 4 | 22 | 3 | 39 | 0.1386 | 0.0824 | 2.3636 |

| Rv2396 | PE_PGRS41 | 4 | 22 | 3 | 39 | 0.1386 | 0.0824 | 2.3636 |
| --- | --- | --- | --- | --- | --- | --- | --- | --- |
| Rv2958c | Rv2958c | 4 | 22 | 3 | 39 | 0.1386 | 0.0824 | 2.3636 |
| Rv2997 | Rv2997 | 4 | 22 | 3 | 39 | 0.1386 | 0.0824 | 2.3636 |
| Rv3379c | dxs2 | 4 | 22 | 3 | 39 | 0.1386 | 0.0824 | 2.3636 |
| Rv2512c | Rv2512c | 17 | 9 | 24 | 18 | 0.2498 | 0.0824 | 1.4167 |
| Rv3451 | cut3 | 20 | 6 | 29 | 13 | 0.2409 | 0.0788 | 1.4943 |
| Rv2383c | mbtB | 7 | 19 | 8 | 34 | 0.2233 | 0.0788 | 1.5658 |
| Rv3897c | Rv3897c | 7 | 19 | 8 | 34 | 0.2233 | 0.0788 | 1.5658 |
| Rv0231 | fadE4 | 2 | 24 | 0 | 42 | 0.0340 | 0.0769 | ND |
| Rv0273c | Rv0273c | 2 | 24 | 0 | 42 | 0.0340 | 0.0769 | ND |
| Rv0275c | Rv0275c | 2 | 24 | 0 | 42 | 0.0340 | 0.0769 | ND |
| Rv0329c | Rv0329c | 2 | 24 | 0 | 42 | 0.0340 | 0.0769 | ND |
| Rv0362 | mgtE | 2 | 24 | 0 | 42 | 0.0340 | 0.0769 | ND |
| Rv0389 | purT | 2 | 24 | 0 | 42 | 0.0340 | 0.0769 | ND |
| Rv0548c | menB | 2 | 24 | 0 | 42 | 0.0340 | 0.0769 | ND |
| Rv0735 | sigL | 2 | 24 | 0 | 42 | 0.0340 | 0.0769 | ND |
| Rv0787A | Rv0787A | 2 | 24 | 0 | 42 | 0.0340 | 0.0769 | ND |
| Rv0921 | Rv0921 | 2 | 24 | 0 | 42 | 0.0340 | 0.0769 | ND |
| Rv0937c | Rv0937c | 2 | 24 | 0 | 42 | 0.0340 | 0.0769 | ND |
| Rv0939 | Rv0939 | 2 | 24 | 0 | 42 | 0.0340 | 0.0769 | ND |
| Rv0994 | moeA1 | 2 | 24 | 0 | 42 | 0.0340 | 0.0769 | ND |
| Rv1024 | Rv1024 | 2 | 24 | 0 | 42 | 0.0340 | 0.0769 | ND |
| Rv1034c | Rv1034c | 2 | 24 | 0 | 42 | 0.0340 | 0.0769 | ND |
| Rv1052 | Rv1052 | 2 | 24 | 0 | 42 | 0.0340 | 0.0769 | ND |
| Rv1060 | Rv1060 | 2 | 24 | 0 | 42 | 0.0340 | 0.0769 | ND |
| Rv1188 | Rv1188 | 2 | 24 | 0 | 42 | 0.0340 | 0.0769 | ND |
| Rv1327c | glgE | 2 | 24 | 0 | 42 | 0.0340 | 0.0769 | ND |
| Rv1334 | Rv1334 | 2 | 24 | 0 | 42 | 0.0340 | 0.0769 | ND |
| Rv1467c | fadE15 | 2 | 24 | 0 | 42 | 0.0340 | 0.0769 | ND |
| Rv1539 | lspA | 2 | 24 | 0 | 42 | 0.0340 | 0.0769 | ND |
| Rv1541c | lprI | 2 | 24 | 0 | 42 | 0.0340 | 0.0769 | ND |
| Rv1544 | Rv1544 | 2 | 24 | 0 | 42 | 0.0340 | 0.0769 | ND |
| Rv1612 | trpB | 2 | 24 | 0 | 42 | 0.0340 | 0.0769 | ND |
| Rv1653 | argJ | 2 | 24 | 0 | 42 | 0.0340 | 0.0769 | ND |

| Rv1654 | argB | 2 | 24 | 0 | 42 | 0.0340 | 0.0769 | ND |
| --- | --- | --- | --- | --- | --- | --- | --- | --- |
| Rv1742 | Rv1742 | 2 | 24 | 0 | 42 | 0.0340 | 0.0769 | ND |
| Rv1752 | Rv1752 | 2 | 24 | 0 | 42 | 0.0340 | 0.0769 | ND |
| Rv1779c | Rv1779c | 2 | 24 | 0 | 42 | 0.0340 | 0.0769 | ND |
| Rv1818c | PE_PGRS33 | 2 | 24 | 0 | 42 | 0.0340 | 0.0769 | ND |
| Rv1865c | Rv1865c | 2 | 24 | 0 | 42 | 0.0340 | 0.0769 | ND |
| Rv1960c | Rv1960c | 2 | 24 | 0 | 42 | 0.0340 | 0.0769 | ND |
| Rv2036 | Rv2036 | 2 | 24 | 0 | 42 | 0.0340 | 0.0769 | ND |
| Rv2205c | Rv2205c | 2 | 24 | 0 | 42 | 0.0340 | 0.0769 | ND |
| Rv2210c | ilvE | 2 | 24 | 0 | 42 | 0.0340 | 0.0769 | ND |
| Rv2317 | uspB | 2 | 24 | 0 | 42 | 0.0340 | 0.0769 | ND |
| Rv2370c | Rv2370c | 2 | 24 | 0 | 42 | 0.0340 | 0.0769 | ND |
| Rv2428 | ahpC | 2 | 24 | 0 | 42 | 0.0340 | 0.0769 | ND |
| Rv2465c | Rv2465c | 2 | 24 | 0 | 42 | 0.0340 | 0.0769 | ND |
| Rv2557 | Rv2557 | 2 | 24 | 0 | 42 | 0.0340 | 0.0769 | ND |
| Rv2646 | Rv2646 | 2 | 24 | 0 | 42 | 0.0340 | 0.0769 | ND |
| Rv2657c | Rv2657c | 2 | 24 | 0 | 42 | 0.0340 | 0.0769 | ND |
| Rv2675c | Rv2675c | 2 | 24 | 0 | 42 | 0.0340 | 0.0769 | ND |
| Rv2833c | ugpB | 2 | 24 | 0 | 42 | 0.0340 | 0.0769 | ND |
| Rv2857c | Rv2857c | 2 | 24 | 0 | 42 | 0.0340 | 0.0769 | ND |
| Rv3039c | echA17 | 2 | 24 | 0 | 42 | 0.0340 | 0.0769 | ND |
| Rv3049c | Rv3049c | 2 | 24 | 0 | 42 | 0.0340 | 0.0769 | ND |
| Rv3083 | Rv3083 | 2 | 24 | 0 | 42 | 0.0340 | 0.0769 | ND |
| Rv3094c | Rv3094c | 2 | 24 | 0 | 42 | 0.0340 | 0.0769 | ND |
| Rv3158 | nuoN | 2 | 24 | 0 | 42 | 0.0340 | 0.0769 | ND |
| Rv3223c | sigH | 2 | 24 | 0 | 42 | 0.0340 | 0.0769 | ND |
| Rv3301c | phoY1 | 2 | 24 | 0 | 42 | 0.0340 | 0.0769 | ND |
| Rv3362c | Rv3362c | 2 | 24 | 0 | 42 | 0.0340 | 0.0769 | ND |
| Rv3393 | iunH | 2 | 24 | 0 | 42 | 0.0340 | 0.0769 | ND |
| Rv3410c | guaB3 | 2 | 24 | 0 | 42 | 0.0340 | 0.0769 | ND |
| Rv3492c | Rv3492c | 2 | 24 | 0 | 42 | 0.0340 | 0.0769 | ND |
| Rv3494c | mce4F | 2 | 24 | 0 | 42 | 0.0340 | 0.0769 | ND |
| Rv3767c | Rv3767c | 2 | 24 | 0 | 42 | 0.0340 | 0.0769 | ND |
| Rv3883c | mycP1 | 2 | 24 | 0 | 42 | 0.0340 | 0.0769 | ND |

| Rv2741 | PE_PGRS47 | 21 | 5 | 25 | 17 | 0.0344 | 0.2125 | 2.8560 |
| --- | --- | --- | --- | --- | --- | --- | --- | --- |
| Rv0833 | PE_PGRS13 | 17 | 9 | 18 | 24 | 0.0354 | 0.2253 | 2.5185 |
| Rv0668 | rpoC | 16 | 10 | 17 | 25 | 0.0456 | 0.2106 | 2.3529 |
| Rv0613c | Rv0613c | 15 | 11 | 21 | 21 | 0.2684 | 0.0769 | 1.3636 |
| Rv0990c | Rv0990c | 15 | 11 | 21 | 21 | 0.2684 | 0.0769 | 1.3636 |
| Rv0236c | Rv0236c | 23 | 3 | 34 | 8 | 0.2069 | 0.0751 | 1.8039 |
| Rv1597 | Rv1597 | 23 | 3 | 34 | 8 | 0.2069 | 0.0751 | 1.8039 |
| Rv1733c | Rv1733c | 23 | 3 | 34 | 8 | 0.2069 | 0.0751 | 1.8039 |
| Rv2807 | Rv2807 | 23 | 3 | 34 | 8 | 0.2069 | 0.0751 | 1.8039 |
| Rv2565 | Rv2565 | 10 | 16 | 13 | 29 | 0.2624 | 0.0751 | 1.3942 |
| Rv0933 | pstB | 5 | 21 | 5 | 37 | 0.2036 | 0.0733 | 1.7619 |
| Rv1877 | Rv1877 | 5 | 21 | 5 | 37 | 0.2036 | 0.0733 | 1.7619 |
| Rv2685 | arsB1 | 5 | 21 | 5 | 37 | 0.2036 | 0.0733 | 1.7619 |
| Rv3018c | PPE46 | 5 | 21 | 5 | 37 | 0.2036 | 0.0733 | 1.7619 |
| Rv0159c | PE3 | 26 | 0 | 39 | 3 | 0.0817 | 0.0714 | ND |
| Rv0787 | Rv0787 | 26 | 0 | 39 | 3 | 0.0817 | 0.0714 | ND |
| Rv0848 | cysK2 | 26 | 0 | 39 | 3 | 0.0817 | 0.0714 | ND |
| Rv1498c | Rv1498c | 26 | 0 | 39 | 3 | 0.0817 | 0.0714 | ND |
| Rv1502 | Rv1502 | 26 | 0 | 39 | 3 | 0.0817 | 0.0714 | ND |
| Rv1548c | PPE21 | 26 | 0 | 39 | 3 | 0.0817 | 0.0714 | ND |
| Rv1809 | PPE33 | 26 | 0 | 39 | 3 | 0.0817 | 0.0714 | ND |
| Rv2079 | Rv2079 | 26 | 0 | 39 | 3 | 0.0817 | 0.0714 | ND |
| Rv2226 | Rv2226 | 26 | 0 | 39 | 3 | 0.0817 | 0.0714 | ND |
| Rv3093c | Rv3093c | 26 | 0 | 39 | 3 | 0.0817 | 0.0714 | ND |
| Rv3447c | Rv3447c | 26 | 0 | 39 | 3 | 0.0817 | 0.0714 | ND |
| Rv1256c | cyp130 | 13 | 13 | 18 | 24 | 0.2827 | 0.0714 | 1.3333 |
| Rv1318c | Rv1318c | 13 | 13 | 18 | 24 | 0.2827 | 0.0714 | 1.3333 |
| Rv1722 | Rv1722 | 13 | 13 | 18 | 24 | 0.2827 | 0.0714 | 1.3333 |
| Rv2791c | Rv2791c | 13 | 13 | 18 | 24 | 0.2827 | 0.0714 | 1.3333 |
| Rv2864c | Rv2864c | 13 | 13 | 18 | 24 | 0.2827 | 0.0714 | 1.3333 |
| Rv3092c | Rv3092c | 13 | 13 | 18 | 24 | 0.2827 | 0.0714 | 1.3333 |
| Rv3636 | Rv3636 | 13 | 13 | 18 | 24 | 0.2827 | 0.0714 | 1.3333 |
| Rv3802c | Rv3802c | 13 | 13 | 18 | 24 | 0.2827 | 0.0714 | 1.3333 |
| Rv0400c | fadE7 | 8 | 18 | 10 | 32 | 0.2636 | 0.0696 | 1.4222 |

| Rv1551 | plsB1 | 8 | 18 | 10 | 32 | 0.2636 | 0.0696 | 1.4222 |
| --- | --- | --- | --- | --- | --- | --- | --- | --- |
| Rv3529c | Rv3529c | 8 | 18 | 10 | 32 | 0.2636 | 0.0696 | 1.4222 |
| Rv3879c | Rv3879c | 8 | 18 | 10 | 32 | 0.2636 | 0.0696 | 1.4222 |
| Rv3900c | Rv3900c | 8 | 18 | 10 | 32 | 0.2636 | 0.0696 | 1.4222 |
| Rv0110 | Rv0110 | 3 | 23 | 2 | 40 | 0.1491 | 0.0678 | 2.6087 |
| Rv0131c | fadE1 | 3 | 23 | 2 | 40 | 0.1491 | 0.0678 | 2.6087 |
| Rv0725c | Rv0724A | 3 | 23 | 2 | 40 | 0.1491 | 0.0678 | 2.6087 |
| Rv0854 | Rv0854 | 3 | 23 | 2 | 40 | 0.1491 | 0.0678 | 2.6087 |
| Rv1901 | cinA | 3 | 23 | 2 | 40 | 0.1491 | 0.0678 | 2.6087 |
| Rv1931c | Rv1931c | 3 | 23 | 2 | 40 | 0.1491 | 0.0678 | 2.6087 |
| Rv2025c | Rv2025c | 3 | 23 | 2 | 40 | 0.1491 | 0.0678 | 2.6087 |
| Rv2124c | metH | 3 | 23 | 2 | 40 | 0.1491 | 0.0678 | 2.6087 |
| Rv2275 | Rv2275 | 3 | 23 | 2 | 40 | 0.1491 | 0.0678 | 2.6087 |
| Rv2574 | Rv2574 | 3 | 23 | 2 | 40 | 0.1491 | 0.0678 | 2.6087 |
| Rv2850c | Rv2850c | 3 | 23 | 2 | 40 | 0.1491 | 0.0678 | 2.6087 |
| Rv3448 | Rv3448 | 3 | 23 | 2 | 40 | 0.1491 | 0.0678 | 2.6087 |
| Rv3877 | Rv3877 | 3 | 23 | 2 | 40 | 0.1491 | 0.0678 | 2.6087 |
| Rv1175c | fadH | 24 | 2 | 36 | 6 | 0.2061 | 0.0659 | 2.0000 |
| Rv0096 | PPE1 | 11 | 15 | 15 | 27 | 0.2933 | 0.0659 | 1.3200 |
| Rv0086 | hycQ | 9 | 17 | 12 | 30 | 0.3001 | 0.0604 | 1.3235 |
| Rv1668c | Rv1668c | 9 | 17 | 12 | 30 | 0.3001 | 0.0604 | 1.3235 |
| Rv0045c | Rv0045c | 4 | 22 | 4 | 38 | 0.2330 | 0.0586 | 1.7273 |
| Rv0209 | Rv0209 | 4 | 22 | 4 | 38 | 0.2330 | 0.0586 | 1.7273 |
| Rv0533c | fabH | 4 | 22 | 4 | 38 | 0.2330 | 0.0586 | 1.7273 |
| Rv0644c | mmaA2 | 4 | 22 | 4 | 38 | 0.2330 | 0.0586 | 1.7273 |
| Rv0697 | Rv0697 | 4 | 22 | 4 | 38 | 0.2330 | 0.0586 | 1.7273 |
| Rv1046c | Rv1046c | 4 | 22 | 4 | 38 | 0.2330 | 0.0586 | 1.7273 |
| Rv1326c | glgB | 4 | 22 | 4 | 38 | 0.2330 | 0.0586 | 1.7273 |
| Rv1452c | PE_PGRS28 | 4 | 22 | 4 | 38 | 0.2330 | 0.0586 | 1.7273 |
| Rv2276 | cyp121 | 4 | 22 | 4 | 38 | 0.2330 | 0.0586 | 1.7273 |
| Rv3586 | Rv3586 | 4 | 22 | 4 | 38 | 0.2330 | 0.0586 | 1.7273 |
| Rv3908 | Rv3908 | 4 | 22 | 4 | 38 | 0.2330 | 0.0586 | 1.7273 |
| Rv1128c | Rv1128c | 12 | 14 | 17 | 25 | 0.3227 | 0.0568 | 1.2605 |
| Rv1787 | PPE25 | 12 | 14 | 17 | 25 | 0.3227 | 0.0568 | 1.2605 |

| Rv0032 | bioF2 | 7 | 19 | 9 | 33 | 0.3019 | 0.0549 | 1.3509 |
| --- | --- | --- | --- | --- | --- | --- | --- | --- |
| Rv0980c | PE_PGRS18 | 7 | 19 | 9 | 33 | 0.3019 | 0.0549 | 1.3509 |
| Rv1595 | nadB | 7 | 19 | 9 | 33 | 0.3019 | 0.0549 | 1.3509 |
| Rv1651c | PE_PGRS30 | 7 | 19 | 9 | 33 | 0.3019 | 0.0549 | 1.3509 |
| Rv0037c | Rv0037c | 2 | 24 | 1 | 41 | 0.1500 | 0.0531 | 3.4167 |
| Rv0042c | Rv0042c | 2 | 24 | 1 | 41 | 0.1500 | 0.0531 | 3.4167 |
| Rv0067c | Rv0067c | 2 | 24 | 1 | 41 | 0.1500 | 0.0531 | 3.4167 |
| Rv0111 | Rv0111 | 2 | 24 | 1 | 41 | 0.1500 | 0.0531 | 3.4167 |
| Rv0160c | PE4 | 2 | 24 | 1 | 41 | 0.1500 | 0.0531 | 3.4167 |
| Rv0168 | yrbE1B | 2 | 24 | 1 | 41 | 0.1500 | 0.0531 | 3.4167 |
| Rv0215c | fadE3 | 2 | 24 | 1 | 41 | 0.1500 | 0.0531 | 3.4167 |
| Rv0257 | Rv0257 | 2 | 24 | 1 | 41 | 0.1500 | 0.0531 | 3.4167 |
| Rv0321 | dcd | 2 | 24 | 1 | 41 | 0.1500 | 0.0531 | 3.4167 |
| Rv0381c | Rv0381c | 2 | 24 | 1 | 41 | 0.1500 | 0.0531 | 3.4167 |
| Rv0416 | thiS | 2 | 24 | 1 | 41 | 0.1500 | 0.0531 | 3.4167 |
| Rv0458 | Rv0458 | 2 | 24 | 1 | 41 | 0.1500 | 0.0531 | 3.4167 |
| Rv0541c | Rv0541c | 2 | 24 | 1 | 41 | 0.1500 | 0.0531 | 3.4167 |
| Rv0566c | Rv0566c | 2 | 24 | 1 | 41 | 0.1500 | 0.0531 | 3.4167 |
| Rv0608 | Rv0608 | 2 | 24 | 1 | 41 | 0.1500 | 0.0531 | 3.4167 |
| Rv0695 | Rv0695 | 2 | 24 | 1 | 41 | 0.1500 | 0.0531 | 3.4167 |
| Rv0894 | Rv0894 | 2 | 24 | 1 | 41 | 0.1500 | 0.0531 | 3.4167 |
| Rv0934 | pstS1 | 2 | 24 | 1 | 41 | 0.1500 | 0.0531 | 3.4167 |
| Rv1032c | trcS | 2 | 24 | 1 | 41 | 0.1500 | 0.0531 | 3.4167 |
| Rv1079 | metB | 2 | 24 | 1 | 41 | 0.1500 | 0.0531 | 3.4167 |
| Rv1129c | Rv1129c | 2 | 24 | 1 | 41 | 0.1500 | 0.0531 | 3.4167 |
| Rv1138c | Rv1138c | 2 | 24 | 1 | 41 | 0.1500 | 0.0531 | 3.4167 |
| Rv1158c | Rv1158c | 2 | 24 | 1 | 41 | 0.1500 | 0.0531 | 3.4167 |
| Rv1621c | cydD | 2 | 24 | 1 | 41 | 0.1500 | 0.0531 | 3.4167 |
| Rv1737c | narK2 | 2 | 24 | 1 | 41 | 0.1500 | 0.0531 | 3.4167 |
| Rv1738 | Rv1738 | 2 | 24 | 1 | 41 | 0.1500 | 0.0531 | 3.4167 |
| Rv1771 | Rv1771 | 2 | 24 | 1 | 41 | 0.1500 | 0.0531 | 3.4167 |
| Rv1781c | malQ | 2 | 24 | 1 | 41 | 0.1500 | 0.0531 | 3.4167 |
| Rv1846c | Rv1846c | 2 | 24 | 1 | 41 | 0.1500 | 0.0531 | 3.4167 |
| Rv1868 | Rv1868 | 2 | 24 | 1 | 41 | 0.1500 | 0.0531 | 3.4167 |

| Rv1946c | lppG | 2 | 24 | 1 | 41 | 0.1500 | 0.0531 | 3.4167 |
| --- | --- | --- | --- | --- | --- | --- | --- | --- |
| Rv1968 | mce3C | 2 | 24 | 1 | 41 | 0.1500 | 0.0531 | 3.4167 |
| Rv2056c | rpsN | 2 | 24 | 1 | 41 | 0.1500 | 0.0531 | 3.4167 |
| Rv2157c | murF | 2 | 24 | 1 | 41 | 0.1500 | 0.0531 | 3.4167 |
| Rv2262c | Rv2262c | 2 | 24 | 1 | 41 | 0.1500 | 0.0531 | 3.4167 |
| Rv2284 | lipM | 2 | 24 | 1 | 41 | 0.1500 | 0.0531 | 3.4167 |
| Rv2382c | mbtC | 2 | 24 | 1 | 41 | 0.1500 | 0.0531 | 3.4167 |
| Rv2395 | Rv2395 | 2 | 24 | 1 | 41 | 0.1500 | 0.0531 | 3.4167 |
| Rv2447c | folC | 2 | 24 | 1 | 41 | 0.1500 | 0.0531 | 3.4167 |
| Rv2551c | Rv2551c | 2 | 24 | 1 | 41 | 0.1500 | 0.0531 | 3.4167 |
| Rv2601 | speE | 2 | 24 | 1 | 41 | 0.1500 | 0.0531 | 3.4167 |
| Rv2823c | Rv2823c | 2 | 24 | 1 | 41 | 0.1500 | 0.0531 | 3.4167 |
| Rv2961 | Rv2961 | 2 | 24 | 1 | 41 | 0.1500 | 0.0531 | 3.4167 |
| Rv2975c | Rv2975c | 2 | 24 | 1 | 41 | 0.1500 | 0.0531 | 3.4167 |
| Rv3096 | Rv3096 | 2 | 24 | 1 | 41 | 0.1500 | 0.0531 | 3.4167 |
| Rv3120 | Rv3120 | 2 | 24 | 1 | 41 | 0.1500 | 0.0531 | 3.4167 |
| Rv3148 | nuoD | 2 | 24 | 1 | 41 | 0.1500 | 0.0531 | 3.4167 |
| Rv3170 | aofH | 2 | 24 | 1 | 41 | 0.1500 | 0.0531 | 3.4167 |
| Rv3256c | Rv3256c | 2 | 24 | 1 | 41 | 0.1500 | 0.0531 | 3.4167 |
| Rv3344c | PE_PGRS49 | 2 | 24 | 1 | 41 | 0.1500 | 0.0531 | 3.4167 |
| Rv3380c | Rv3380c | 2 | 24 | 1 | 41 | 0.1500 | 0.0531 | 3.4167 |
| Rv3423c | alr | 2 | 24 | 1 | 41 | 0.1500 | 0.0531 | 3.4167 |
| Rv3434c | Rv3434c | 2 | 24 | 1 | 41 | 0.1500 | 0.0531 | 3.4167 |
| Rv3472 | Rv3472 | 2 | 24 | 1 | 41 | 0.1500 | 0.0531 | 3.4167 |
| Rv3653 | PE_PGRS61 | 2 | 24 | 1 | 41 | 0.1500 | 0.0531 | 3.4167 |
| Rv3704c | gshA | 2 | 24 | 1 | 41 | 0.1500 | 0.0531 | 3.4167 |
| Rv3808c | glfT | 2 | 24 | 1 | 41 | 0.1500 | 0.0531 | 3.4167 |
| Rv4007 | Rv4007 | 2 | 24 | 1 | 41 | 0.1500 | 0.0531 | 3.4167 |
| Rv0412c | Rv0412c | 23 | 3 | 35 | 7 | 0.2809 | 0.0513 | 1.5333 |
| Rv1161 | narG | 5 | 21 | 6 | 36 | 0.2952 | 0.0495 | 1.4286 |
| Rv0064 | Rv0064 | 26 | 0 | 40 | 2 | 0.1294 | 0.0476 | ND |
| Rv0962c | lprP | 26 | 0 | 40 | 2 | 0.1294 | 0.0476 | ND |
| Rv3894c | Rv3894c | 26 | 0 | 40 | 2 | 0.1294 | 0.0476 | ND |
| Rv2854 | Rv2854 | 13 | 13 | 19 | 23 | 0.3511 | 0.0476 | 1.2105 |

| Rv3279c | birA | 8 | 18 | 11 | 31 | 0.3413 | 0.0458 | 1.2525 |
| --- | --- | --- | --- | --- | --- | --- | --- | --- |
| Rv0132c | fgd2 | 3 | 23 | 3 | 39 | 0.2673 | 0.0440 | 1.6957 |
| Rv0277c | Rv0277c | 3 | 23 | 3 | 39 | 0.2673 | 0.0440 | 1.6957 |
| Rv0298 | Rv0298 | 3 | 23 | 3 | 39 | 0.2673 | 0.0440 | 1.6957 |
| Rv0341 | iniB | 3 | 23 | 3 | 39 | 0.2673 | 0.0440 | 1.6957 |
| Rv0508 | Rv0508 | 3 | 23 | 3 | 39 | 0.2673 | 0.0440 | 1.6957 |
| Rv0513 | Rv0513 | 3 | 23 | 3 | 39 | 0.2673 | 0.0440 | 1.6957 |
| Rv0716 | rplE | 3 | 23 | 3 | 39 | 0.2673 | 0.0440 | 1.6957 |
| Rv0961 | Rv0961 | 3 | 23 | 3 | 39 | 0.2673 | 0.0440 | 1.6957 |
| Rv0999 | Rv0999 | 3 | 23 | 3 | 39 | 0.2673 | 0.0440 | 1.6957 |
| Rv1020 | mfd | 3 | 23 | 3 | 39 | 0.2673 | 0.0440 | 1.6957 |
| Rv1189 | sigI | 3 | 23 | 3 | 39 | 0.2673 | 0.0440 | 1.6957 |
| Rv1308 | atpA | 3 | 23 | 3 | 39 | 0.2673 | 0.0440 | 1.6957 |
| Rv1324 | Rv1324 | 3 | 23 | 3 | 39 | 0.2673 | 0.0440 | 1.6957 |
| Rv1337 | Rv1337 | 3 | 23 | 3 | 39 | 0.2673 | 0.0440 | 1.6957 |
| Rv1504c | Rv1504c | 3 | 23 | 3 | 39 | 0.2673 | 0.0440 | 1.6957 |
| Rv1508A | Rv1508A | 3 | 23 | 3 | 39 | 0.2673 | 0.0440 | 1.6957 |
| Rv1600 | hisC1 | 3 | 23 | 3 | 39 | 0.2673 | 0.0440 | 1.6957 |
| Rv1607 | chaA | 3 | 23 | 3 | 39 | 0.2673 | 0.0440 | 1.6957 |
| Rv1673c | Rv1673c | 3 | 23 | 3 | 39 | 0.2673 | 0.0440 | 1.6957 |
| Rv1727 | Rv1727 | 3 | 23 | 3 | 39 | 0.2673 | 0.0440 | 1.6957 |
| Rv1747 | Rv1747 | 3 | 23 | 3 | 39 | 0.2673 | 0.0440 | 1.6957 |
| Rv1750c | fadD1 | 3 | 23 | 3 | 39 | 0.2673 | 0.0440 | 1.6957 |
| Rv1863c | Rv1863c | 3 | 23 | 3 | 39 | 0.2673 | 0.0440 | 1.6957 |
| Rv1886c | fbpB | 3 | 23 | 3 | 39 | 0.2673 | 0.0440 | 1.6957 |
| Rv2030c | Rv2030c | 3 | 23 | 3 | 39 | 0.2673 | 0.0440 | 1.6957 |
| Rv2214c | ephD | 3 | 23 | 3 | 39 | 0.2673 | 0.0440 | 1.6957 |
| Rv2237 | Rv2237 | 3 | 23 | 3 | 39 | 0.2673 | 0.0440 | 1.6957 |
| Rv2373c | dnaJ2 | 3 | 23 | 3 | 39 | 0.2673 | 0.0440 | 1.6957 |
| Rv2385 | mbtJ | 3 | 23 | 3 | 39 | 0.2673 | 0.0440 | 1.6957 |
| Rv2403c | lppR | 3 | 23 | 3 | 39 | 0.2673 | 0.0440 | 1.6957 |
| Rv2414c | Rv2414c | 3 | 23 | 3 | 39 | 0.2673 | 0.0440 | 1.6957 |
| Rv2452c | Rv2452c | 3 | 23 | 3 | 39 | 0.2673 | 0.0440 | 1.6957 |
| Rv2511 | orn | 3 | 23 | 3 | 39 | 0.2673 | 0.0440 | 1.6957 |

| Rv2612c | pgsA1 | 3 | 23 | 3 | 39 | 0.2673 | 0.0440 | 1.6957 |
| --- | --- | --- | --- | --- | --- | --- | --- | --- |
| Rv2664 | Rv2664 | 3 | 23 | 3 | 39 | 0.2673 | 0.0440 | 1.6957 |
| Rv2739c | Rv2739c | 3 | 23 | 3 | 39 | 0.2673 | 0.0440 | 1.6957 |
| Rv2824c | Rv2824c | 3 | 23 | 3 | 39 | 0.2673 | 0.0440 | 1.6957 |
| Rv2870c | dxr | 3 | 23 | 3 | 39 | 0.2673 | 0.0440 | 1.6957 |
| Rv2930 | fadD26 | 3 | 23 | 3 | 39 | 0.2673 | 0.0440 | 1.6957 |
| Rv3058c | Rv3058c | 3 | 23 | 3 | 39 | 0.2673 | 0.0440 | 1.6957 |
| Rv3106 | fprA | 3 | 23 | 3 | 39 | 0.2673 | 0.0440 | 1.6957 |
| Rv3224 | Rv3224 | 3 | 23 | 3 | 39 | 0.2673 | 0.0440 | 1.6957 |
| Rv3252c | alkB | 3 | 23 | 3 | 39 | 0.2673 | 0.0440 | 1.6957 |
| Rv3355c | Rv3355c | 3 | 23 | 3 | 39 | 0.2673 | 0.0440 | 1.6957 |
| Rv3421c | Rv3421c | 3 | 23 | 3 | 39 | 0.2673 | 0.0440 | 1.6957 |
| Rv3580c | cysS | 3 | 23 | 3 | 39 | 0.2673 | 0.0440 | 1.6957 |
| Rv3587c | Rv3587c | 3 | 23 | 3 | 39 | 0.2673 | 0.0440 | 1.6957 |
| Rv3673c | Rv3673c | 3 | 23 | 3 | 39 | 0.2673 | 0.0440 | 1.6957 |
| Rv3712 | Rv3712 | 3 | 23 | 3 | 39 | 0.2673 | 0.0440 | 1.6957 |
| Rv3762c | Rv3762c | 3 | 23 | 3 | 39 | 0.2673 | 0.0440 | 1.6957 |
| Rv3907c | pcnA | 3 | 23 | 3 | 39 | 0.2673 | 0.0440 | 1.6957 |
| Rv3915 | Rv3915 | 3 | 23 | 3 | 39 | 0.2673 | 0.0440 | 1.6957 |
| Rv3097c | lipY | 11 | 15 | 16 | 26 | 0.3650 | 0.0421 | 1.1917 |
| Rv3884c | Rv3884c | 11 | 15 | 16 | 26 | 0.3650 | 0.0421 | 1.1917 |
| Rv0062 | celA1 | 6 | 20 | 8 | 34 | 0.3448 | 0.0403 | 1.2750 |
| Rv0092 | ctpA | 6 | 20 | 8 | 34 | 0.3448 | 0.0403 | 1.2750 |
| Rv1384 | carB | 6 | 20 | 8 | 34 | 0.3448 | 0.0403 | 1.2750 |
| Rv1518 | Rv1518 | 6 | 20 | 8 | 34 | 0.3448 | 0.0403 | 1.2750 |
| Rv1995 | Rv1995 | 6 | 20 | 8 | 34 | 0.3448 | 0.0403 | 1.2750 |
| Rv2308 | Rv2308 | 6 | 20 | 8 | 34 | 0.3448 | 0.0403 | 1.2750 |
| Rv0004 | Rv0004 | 1 | 25 | 0 | 42 | 0.1002 | 0.0385 | ND |
| Rv0010c | Rv0010c | 1 | 25 | 0 | 42 | 0.1002 | 0.0385 | ND |
| Rv0028 | Rv0028 | 1 | 25 | 0 | 42 | 0.1002 | 0.0385 | ND |
| Rv0060 | Rv0060 | 1 | 25 | 0 | 42 | 0.1002 | 0.0385 | ND |
| Rv0069c | sdaA | 1 | 25 | 0 | 42 | 0.1002 | 0.0385 | ND |
| Rv0077c | Rv0077c | 1 | 25 | 0 | 42 | 0.1002 | 0.0385 | ND |
| Rv0083 | Rv0083 | 1 | 25 | 0 | 42 | 0.1002 | 0.0385 | ND |

| Rv0084 | hycD | 1 | 25 | 0 | 42 | 0.1002 | 0.0385 | ND |
| --- | --- | --- | --- | --- | --- | --- | --- | --- |
| Rv0088 | Rv0088 | 1 | 25 | 0 | 42 | 0.1002 | 0.0385 | ND |
| Rv0121c | Rv0121c | 1 | 25 | 0 | 42 | 0.1002 | 0.0385 | ND |
| Rv0122 | Rv0122 | 1 | 25 | 0 | 42 | 0.1002 | 0.0385 | ND |
| Rv0184 | Rv0184 | 1 | 25 | 0 | 42 | 0.1002 | 0.0385 | ND |
| Rv0202c | mmpL11 | 1 | 25 | 0 | 42 | 0.1002 | 0.0385 | ND |
| Rv0214 | fadD4 | 1 | 25 | 0 | 42 | 0.1002 | 0.0385 | ND |
| Rv0222 | echA1 | 1 | 25 | 0 | 42 | 0.1002 | 0.0385 | ND |
| Rv0224c | Rv0224c | 1 | 25 | 0 | 42 | 0.1002 | 0.0385 | ND |
| Rv0232 | Rv0232 | 1 | 25 | 0 | 42 | 0.1002 | 0.0385 | ND |
| Rv0258c | Rv0258c | 1 | 25 | 0 | 42 | 0.1002 | 0.0385 | ND |
| Rv0263c | Rv0263c | 1 | 25 | 0 | 42 | 0.1002 | 0.0385 | ND |
| Rv0310c | Rv0310c | 1 | 25 | 0 | 42 | 0.1002 | 0.0385 | ND |
| Rv0352 | dnaJ1 | 1 | 25 | 0 | 42 | 0.1002 | 0.0385 | ND |
| Rv0357c | purA | 1 | 25 | 0 | 42 | 0.1002 | 0.0385 | ND |
| Rv0448c | Rv0448c | 1 | 25 | 0 | 42 | 0.1002 | 0.0385 | ND |
| Rv0470A | Rv0470A | 1 | 25 | 0 | 42 | 0.1002 | 0.0385 | ND |
| Rv0496 | Rv0496 | 1 | 25 | 0 | 42 | 0.1002 | 0.0385 | ND |
| Rv0527 | ccdA | 1 | 25 | 0 | 42 | 0.1002 | 0.0385 | ND |
| Rv0564c | gpsA | 1 | 25 | 0 | 42 | 0.1002 | 0.0385 | ND |
| Rv0591 | mce2C | 1 | 25 | 0 | 42 | 0.1002 | 0.0385 | ND |
| Rv0611c | Rv0611c | 1 | 25 | 0 | 42 | 0.1002 | 0.0385 | ND |
| Rv0683 | rpsG | 1 | 25 | 0 | 42 | 0.1002 | 0.0385 | ND |
| Rv0684 | fusA1 | 1 | 25 | 0 | 42 | 0.1002 | 0.0385 | ND |
| Rv0705 | rpsS | 1 | 25 | 0 | 42 | 0.1002 | 0.0385 | ND |
| Rv0757 | phoP | 1 | 25 | 0 | 42 | 0.1002 | 0.0385 | ND |
| Rv0775 | Rv0775 | 1 | 25 | 0 | 42 | 0.1002 | 0.0385 | ND |
| Rv0806c | cpsY | 1 | 25 | 0 | 42 | 0.1002 | 0.0385 | ND |
| Rv0906 | Rv0906 | 1 | 25 | 0 | 42 | 0.1002 | 0.0385 | ND |
| Rv0929 | pstC2 | 1 | 25 | 0 | 42 | 0.1002 | 0.0385 | ND |
| Rv1069c | Rv1069c | 1 | 25 | 0 | 42 | 0.1002 | 0.0385 | ND |
| Rv1111c | Rv1111c | 1 | 25 | 0 | 42 | 0.1002 | 0.0385 | ND |
| Rv1210 | tagA | 1 | 25 | 0 | 42 | 0.1002 | 0.0385 | ND |
| Rv1222 | Rv1222 | 1 | 25 | 0 | 42 | 0.1002 | 0.0385 | ND |

| Rv1234 | Rv1234 | 1 | 25 | 0 | 42 | 0.1002 | 0.0385 | ND |
| --- | --- | --- | --- | --- | --- | --- | --- | --- |
| Rv1238 | sugC | 1 | 25 | 0 | 42 | 0.1002 | 0.0385 | ND |
| Rv1245c | Rv1245c | 1 | 25 | 0 | 42 | 0.1002 | 0.0385 | ND |
| Rv1257c | Rv1257c | 1 | 25 | 0 | 42 | 0.1002 | 0.0385 | ND |
| Rv1269c | Rv1269c | 1 | 25 | 0 | 42 | 0.1002 | 0.0385 | ND |
| Rv1270c | lprA | 1 | 25 | 0 | 42 | 0.1002 | 0.0385 | ND |
| Rv1307 | atpH | 1 | 25 | 0 | 42 | 0.1002 | 0.0385 | ND |
| Rv1311 | atpC | 1 | 25 | 0 | 42 | 0.1002 | 0.0385 | ND |
| Rv1322 | Rv1322 | 1 | 25 | 0 | 42 | 0.1002 | 0.0385 | ND |
| Rv1332 | Rv1332 | 1 | 25 | 0 | 42 | 0.1002 | 0.0385 | ND |
| Rv1345 | fadD33 | 1 | 25 | 0 | 42 | 0.1002 | 0.0385 | ND |
| Rv1365c | rsfA | 1 | 25 | 0 | 42 | 0.1002 | 0.0385 | ND |
| Rv1455 | Rv1455 | 1 | 25 | 0 | 42 | 0.1002 | 0.0385 | ND |
| Rv1480 | Rv1480 | 1 | 25 | 0 | 42 | 0.1002 | 0.0385 | ND |
| Rv1525 | wbbL2 | 1 | 25 | 0 | 42 | 0.1002 | 0.0385 | ND |
| Rv1656 | argF | 1 | 25 | 0 | 42 | 0.1002 | 0.0385 | ND |
| Rv1659 | argH | 1 | 25 | 0 | 42 | 0.1002 | 0.0385 | ND |
| Rv1682 | Rv1682 | 1 | 25 | 0 | 42 | 0.1002 | 0.0385 | ND |
| Rv1688 | mpg | 1 | 25 | 0 | 42 | 0.1002 | 0.0385 | ND |
| Rv1717 | Rv1717 | 1 | 25 | 0 | 42 | 0.1002 | 0.0385 | ND |
| Rv1726 | Rv1726 | 1 | 25 | 0 | 42 | 0.1002 | 0.0385 | ND |
| Rv1764 | Rv1764 | 1 | 25 | 0 | 42 | 0.1002 | 0.0385 | ND |
| Rv1855c | Rv1855c | 1 | 25 | 0 | 42 | 0.1002 | 0.0385 | ND |
| Rv1867 | Rv1867 | 1 | 25 | 0 | 42 | 0.1002 | 0.0385 | ND |
| Rv1928c | Rv1928c | 1 | 25 | 0 | 42 | 0.1002 | 0.0385 | ND |
| Rv1944c | Rv1944c | 1 | 25 | 0 | 42 | 0.1002 | 0.0385 | ND |
| Rv1955 | Rv1954c | 1 | 25 | 0 | 42 | 0.1002 | 0.0385 | ND |
| Rv1961 | Rv1961 | 1 | 25 | 0 | 42 | 0.1002 | 0.0385 | ND |
| Rv1996 | Rv1996 | 1 | 25 | 0 | 42 | 0.1002 | 0.0385 | ND |
| Rv2020c | Rv2020c | 1 | 25 | 0 | 42 | 0.1002 | 0.0385 | ND |
| Rv2049c | Rv2049c | 1 | 25 | 0 | 42 | 0.1002 | 0.0385 | ND |
| Rv2108 | PPE36 | 1 | 25 | 0 | 42 | 0.1002 | 0.0385 | ND |
| Rv2118c | Rv2118c | 1 | 25 | 0 | 42 | 0.1002 | 0.0385 | ND |
| Rv2133c | Rv2133c | 1 | 25 | 0 | 42 | 0.1002 | 0.0385 | ND |

| Rv2134c | Rv2134c | 1 | 25 | 0 | 42 | 0.1002 | 0.0385 | ND |
| --- | --- | --- | --- | --- | --- | --- | --- | --- |
| Rv2183c | Rv2183c | 1 | 25 | 0 | 42 | 0.1002 | 0.0385 | ND |
| Rv2188c | Rv2188c | 1 | 25 | 0 | 42 | 0.1002 | 0.0385 | ND |
| Rv2295 | Rv2295 | 1 | 25 | 0 | 42 | 0.1002 | 0.0385 | ND |
| Rv2303c | Rv2303c | 1 | 25 | 0 | 42 | 0.1002 | 0.0385 | ND |
| Rv2318 | uspC | 1 | 25 | 0 | 42 | 0.1002 | 0.0385 | ND |
| Rv2332 | mez | 1 | 25 | 0 | 42 | 0.1002 | 0.0385 | ND |
| Rv2344c | dgt | 1 | 25 | 0 | 42 | 0.1002 | 0.0385 | ND |
| Rv2362c | recO | 1 | 25 | 0 | 42 | 0.1002 | 0.0385 | ND |
| Rv2384 | mbtA | 1 | 25 | 0 | 42 | 0.1002 | 0.0385 | ND |
| Rv2393 | Rv2393 | 1 | 25 | 0 | 42 | 0.1002 | 0.0385 | ND |
| Rv2406c | Rv2406c | 1 | 25 | 0 | 42 | 0.1002 | 0.0385 | ND |
| Rv2413c | Rv2413c | 1 | 25 | 0 | 42 | 0.1002 | 0.0385 | ND |
| Rv2423 | Rv2423 | 1 | 25 | 0 | 42 | 0.1002 | 0.0385 | ND |
| Rv2457c | clpX | 1 | 25 | 0 | 42 | 0.1002 | 0.0385 | ND |
| Rv2478c | Rv2478c | 1 | 25 | 0 | 42 | 0.1002 | 0.0385 | ND |
| Rv2479c | Rv2479c | 1 | 25 | 0 | 42 | 0.1002 | 0.0385 | ND |
| Rv2508c | Rv2508c | 1 | 25 | 0 | 42 | 0.1002 | 0.0385 | ND |
| Rv2676c | Rv2676c | 1 | 25 | 0 | 42 | 0.1002 | 0.0385 | ND |
| Rv2730 | Rv2730 | 1 | 25 | 0 | 42 | 0.1002 | 0.0385 | ND |
| Rv2737c | recA | 1 | 25 | 0 | 42 | 0.1002 | 0.0385 | ND |
| Rv2738c | Rv2738c | 1 | 25 | 0 | 42 | 0.1002 | 0.0385 | ND |
| Rv2784c | lppU | 1 | 25 | 0 | 42 | 0.1002 | 0.0385 | ND |
| Rv2793c | truB | 1 | 25 | 0 | 42 | 0.1002 | 0.0385 | ND |
| Rv2819c | Rv2819c | 1 | 25 | 0 | 42 | 0.1002 | 0.0385 | ND |
| Rv2856 | nicT | 1 | 25 | 0 | 42 | 0.1002 | 0.0385 | ND |
| Rv2888c | amiC | 1 | 25 | 0 | 42 | 0.1002 | 0.0385 | ND |
| Rv2890c | rpsB | 1 | 25 | 0 | 42 | 0.1002 | 0.0385 | ND |
| Rv2900c | fdhF | 1 | 25 | 0 | 42 | 0.1002 | 0.0385 | ND |
| Rv2932 | ppsB | 1 | 25 | 0 | 42 | 0.1002 | 0.0385 | ND |
| Rv3003c | ilvB1 | 1 | 25 | 0 | 42 | 0.1002 | 0.0385 | ND |
| Rv3009c | gatB | 1 | 25 | 0 | 42 | 0.1002 | 0.0385 | ND |
| Rv3036c | TB22.2 | 1 | 25 | 0 | 42 | 0.1002 | 0.0385 | ND |
| Rv3053c | nrdH | 1 | 25 | 0 | 42 | 0.1002 | 0.0385 | ND |

| Rv3073c | Rv3073c | 1 | 25 | 0 | 42 | 0.1002 | 0.0385 | ND |
| --- | --- | --- | --- | --- | --- | --- | --- | --- |
| Rv3086 | adhD | 1 | 25 | 0 | 42 | 0.1002 | 0.0385 | ND |
| Rv3147 | nuoC | 1 | 25 | 0 | 42 | 0.1002 | 0.0385 | ND |
| Rv3176c | mesT | 1 | 25 | 0 | 42 | 0.1002 | 0.0385 | ND |
| Rv3205c | Rv3205c | 1 | 25 | 0 | 42 | 0.1002 | 0.0385 | ND |
| Rv3249c | Rv3249c | 1 | 25 | 0 | 42 | 0.1002 | 0.0385 | ND |
| Rv3281 | Rv3281 | 1 | 25 | 0 | 42 | 0.1002 | 0.0385 | ND |
| Rv3309c | upp | 1 | 25 | 0 | 42 | 0.1002 | 0.0385 | ND |
| Rv3310 | Rv3310 | 1 | 25 | 0 | 42 | 0.1002 | 0.0385 | ND |
| Rv3322c | Rv3322c | 1 | 25 | 0 | 42 | 0.1002 | 0.0385 | ND |
| Rv3346c | Rv3346c | 1 | 25 | 0 | 42 | 0.1002 | 0.0385 | ND |
| Rv3377c | Rv3377c | 1 | 25 | 0 | 42 | 0.1002 | 0.0385 | ND |
| Rv3390 | lpqD | 1 | 25 | 0 | 42 | 0.1002 | 0.0385 | ND |
| Rv3419c | gcp | 1 | 25 | 0 | 42 | 0.1002 | 0.0385 | ND |
| Rv3452 | cut4 | 1 | 25 | 0 | 42 | 0.1002 | 0.0385 | ND |
| Rv3470c | ilvB2 | 1 | 25 | 0 | 42 | 0.1002 | 0.0385 | ND |
| Rv3480c | Rv3480c | 1 | 25 | 0 | 42 | 0.1002 | 0.0385 | ND |
| Rv3498c | mce4B | 1 | 25 | 0 | 42 | 0.1002 | 0.0385 | ND |
| Rv3517 | Rv3517 | 1 | 25 | 0 | 42 | 0.1002 | 0.0385 | ND |
| Rv3524 | Rv3524 | 1 | 25 | 0 | 42 | 0.1002 | 0.0385 | ND |
| Rv3532 | PPE61 | 1 | 25 | 0 | 42 | 0.1002 | 0.0385 | ND |
| Rv3543c | fadE29 | 1 | 25 | 0 | 42 | 0.1002 | 0.0385 | ND |
| Rv3605c | Rv3605c | 1 | 25 | 0 | 42 | 0.1002 | 0.0385 | ND |
| Rv3610c | ftsH | 1 | 25 | 0 | 42 | 0.1002 | 0.0385 | ND |
| Rv3662c | Rv3662c | 1 | 25 | 0 | 42 | 0.1002 | 0.0385 | ND |
| Rv3675 | Rv3675 | 1 | 25 | 0 | 42 | 0.1002 | 0.0385 | ND |
| Rv3685c | cyp137 | 1 | 25 | 0 | 42 | 0.1002 | 0.0385 | ND |
| Rv3721c | dnaZX | 1 | 25 | 0 | 42 | 0.1002 | 0.0385 | ND |
| Rv3738c | PPE66 | 1 | 25 | 0 | 42 | 0.1002 | 0.0385 | ND |
| Rv3765c | Rv3765c | 1 | 25 | 0 | 42 | 0.1002 | 0.0385 | ND |
| Rv3766 | Rv3766 | 1 | 25 | 0 | 42 | 0.1002 | 0.0385 | ND |
| Rv3779 | Rv3779 | 1 | 25 | 0 | 42 | 0.1002 | 0.0385 | ND |
| Rv3809c | glf | 1 | 25 | 0 | 42 | 0.1002 | 0.0385 | ND |
| Rv3832c | Rv3832c | 1 | 25 | 0 | 42 | 0.1002 | 0.0385 | ND |

| Rv3852 | hns | 1 | 25 | 0 | 42 | 0.1002 | 0.0385 | ND |
| --- | --- | --- | --- | --- | --- | --- | --- | --- |
| Rv3855 | ethR | 1 | 25 | 0 | 42 | 0.1002 | 0.0385 | ND |
| Rv3921c | Rv3921c | 1 | 25 | 0 | 42 | 0.1002 | 0.0385 | ND |
| Rv0405 | pks6 | 9 | 17 | 13 | 29 | 0.3768 | 0.0366 | 1.1810 |
| Rv1087 | PE_PGRS21 | 9 | 17 | 13 | 29 | 0.3768 | 0.0366 | 1.1810 |
| Rv2402 | Rv2402 | 9 | 17 | 13 | 29 | 0.3768 | 0.0366 | 1.1810 |
| Rv3263 | Rv3263 | 9 | 17 | 13 | 29 | 0.3768 | 0.0366 | 1.1810 |
| Rv0982 | mprB | 4 | 22 | 5 | 37 | 0.3403 | 0.0348 | 1.3455 |
| Rv1027c | kdpE | 4 | 22 | 5 | 37 | 0.3403 | 0.0348 | 1.3455 |
| Rv1280c | oppA | 4 | 22 | 5 | 37 | 0.3403 | 0.0348 | 1.3455 |
| Rv1705c | PPE22 | 4 | 22 | 5 | 37 | 0.3403 | 0.0348 | 1.3455 |
| Rv1999c | Rv1999c | 4 | 22 | 5 | 37 | 0.3403 | 0.0348 | 1.3455 |
| Rv2484c | Rv2484c | 4 | 22 | 5 | 37 | 0.3403 | 0.0348 | 1.3455 |
| Rv2712c | Rv2712c | 4 | 22 | 5 | 37 | 0.3403 | 0.0348 | 1.3455 |
| Rv2733c | Rv2733c | 4 | 22 | 5 | 37 | 0.3403 | 0.0348 | 1.3455 |
| Rv3139 | fadE24 | 4 | 22 | 5 | 37 | 0.3403 | 0.0348 | 1.3455 |
| Rv3429 | PPE59 | 4 | 22 | 5 | 37 | 0.3403 | 0.0348 | 1.3455 |
| Rv3773c | Rv3773c | 4 | 22 | 5 | 37 | 0.3403 | 0.0348 | 1.3455 |
| Rv3797 | fadE35 | 4 | 22 | 5 | 37 | 0.3403 | 0.0348 | 1.3455 |
| Rv2082 | Rv2082 | 25 | 1 | 39 | 3 | 0.2872 | 0.0330 | 1.9231 |
| Rv1198 | esxL | 12 | 14 | 18 | 24 | 0.3951 | 0.0330 | 1.1429 |
| Rv3892c | PPE69 | 20 | 6 | 31 | 11 | 0.3866 | 0.0311 | 1.1828 |
| Rv0515 | Rv0515 | 7 | 19 | 10 | 32 | 0.3866 | 0.0311 | 1.1789 |
| Rv1353c | Rv1353c | 7 | 19 | 10 | 32 | 0.3866 | 0.0311 | 1.1789 |
| Rv1521 | fadD25 | 7 | 19 | 10 | 32 | 0.3866 | 0.0311 | 1.1789 |
| Rv1577c | Rv1577c | 7 | 19 | 10 | 32 | 0.3866 | 0.0311 | 1.1789 |
| Rv0235c | Rv0235c | 2 | 24 | 2 | 40 | 0.3089 | 0.0293 | 1.6667 |
| Rv0317c | glpQ2 | 2 | 24 | 2 | 40 | 0.3089 | 0.0293 | 1.6667 |
| Rv0630c | recB | 2 | 24 | 2 | 40 | 0.3089 | 0.0293 | 1.6667 |
| Rv0754 | PE_PGRS11 | 2 | 24 | 2 | 40 | 0.3089 | 0.0293 | 1.6667 |
| Rv0892 | Rv0892 | 2 | 24 | 2 | 40 | 0.3089 | 0.0293 | 1.6667 |
| Rv0946c | pgi | 2 | 24 | 2 | 40 | 0.3089 | 0.0293 | 1.6667 |
| Rv1133c | metE | 2 | 24 | 2 | 40 | 0.3089 | 0.0293 | 1.6667 |
| Rv1140 | Rv1140 | 2 | 24 | 2 | 40 | 0.3089 | 0.0293 | 1.6667 |

| Rv1350 | fabG | 2 | 24 | 2 | 40 | 0.3089 | 0.0293 | 1.6667 |
| --- | --- | --- | --- | --- | --- | --- | --- | --- |
| Rv2634c | PE_PGRS46 | 2 | 24 | 2 | 40 | 0.3089 | 0.0293 | 1.6667 |
| Rv2689c | Rv2689c | 2 | 24 | 2 | 40 | 0.3089 | 0.0293 | 1.6667 |
| Rv2707 | Rv2707 | 2 | 24 | 2 | 40 | 0.3089 | 0.0293 | 1.6667 |
| Rv2753c | dapA | 2 | 24 | 2 | 40 | 0.3089 | 0.0293 | 1.6667 |
| Rv2996c | serA1 | 2 | 24 | 2 | 40 | 0.3089 | 0.0293 | 1.6667 |
| Rv3016 | lpqA | 2 | 24 | 2 | 40 | 0.3089 | 0.0293 | 1.6667 |
| Rv3227 | aroA | 2 | 24 | 2 | 40 | 0.3089 | 0.0293 | 1.6667 |
| Rv3270 | ctpC | 2 | 24 | 2 | 40 | 0.3089 | 0.0293 | 1.6667 |
| Rv3370c | dnaE2 | 2 | 24 | 2 | 40 | 0.3089 | 0.0293 | 1.6667 |
| Rv3608c | folP1 | 2 | 24 | 2 | 40 | 0.3089 | 0.0293 | 1.6667 |
| Rv3660c | Rv3660c | 2 | 24 | 2 | 40 | 0.3089 | 0.0293 | 1.6667 |
| Rv3811 | Rv3811 | 2 | 24 | 2 | 40 | 0.3089 | 0.0293 | 1.6667 |
| Rv3886c | mycP2 | 2 | 24 | 2 | 40 | 0.3089 | 0.0293 | 1.6667 |
| Rv1199c | Rv1199c | 15 | 11 | 23 | 19 | 0.4065 | 0.0293 | 1.1265 |
| Rv3842c | glpQ1 | 15 | 11 | 23 | 19 | 0.4065 | 0.0293 | 1.1265 |
| Rv1159 | pimE | 5 | 21 | 7 | 35 | 0.3938 | 0.0256 | 1.1905 |
| Rv1387 | PPE20 | 5 | 21 | 7 | 35 | 0.3938 | 0.0256 | 1.1905 |
| Rv1762c | Rv1762c | 5 | 21 | 7 | 35 | 0.3938 | 0.0256 | 1.1905 |
| Rv1769 | Rv1769 | 5 | 21 | 7 | 35 | 0.3938 | 0.0256 | 1.1905 |
| Rv2692 | ceoC | 5 | 21 | 7 | 35 | 0.3938 | 0.0256 | 1.1905 |
| Rv0082 | Rv0082 | 26 | 0 | 41 | 1 | 0.2140 | 0.0238 | ND |
| Rv0323c | Rv0323c | 26 | 0 | 41 | 1 | 0.2140 | 0.0238 | ND |
| Rv2650c | Rv2650c | 26 | 0 | 41 | 1 | 0.2140 | 0.0238 | ND |
| Rv2668 | Rv2668 | 26 | 0 | 41 | 1 | 0.2140 | 0.0238 | ND |
| Rv3063 | cstA | 26 | 0 | 41 | 1 | 0.2140 | 0.0238 | ND |
| Rv0175 | Rv0175 | 13 | 13 | 20 | 22 | 0.4243 | 0.0238 | 1.1000 |
| Rv0696 | Rv0696 | 13 | 13 | 20 | 22 | 0.4243 | 0.0238 | 1.1000 |
| Rv1251c | Rv1251c | 8 | 18 | 12 | 30 | 0.4234 | 0.0220 | 1.1111 |
| Rv0171 | mce1C | 3 | 23 | 4 | 38 | 0.3952 | 0.0201 | 1.2391 |
| Rv0226c | Rv0226c | 3 | 23 | 4 | 38 | 0.3952 | 0.0201 | 1.2391 |
| Rv0290 | Rv0290 | 3 | 23 | 4 | 38 | 0.3952 | 0.0201 | 1.2391 |
| Rv0421c | Rv0421c | 3 | 23 | 4 | 38 | 0.3952 | 0.0201 | 1.2391 |
| Rv0522 | gabP | 3 | 23 | 4 | 38 | 0.3952 | 0.0201 | 1.2391 |

| Rv0537c | Rv0537c | 3 | 23 | 4 | 38 | 0.3952 | 0.0201 | 1.2391 |
| --- | --- | --- | --- | --- | --- | --- | --- | --- |
| Rv0641 | rplA | 3 | 23 | 4 | 38 | 0.3952 | 0.0201 | 1.2391 |
| Rv0893c | Rv0893c | 3 | 23 | 4 | 38 | 0.3952 | 0.0201 | 1.2391 |
| Rv0972c | fadE12 | 3 | 23 | 4 | 38 | 0.3952 | 0.0201 | 1.2391 |
| Rv1730c | Rv1730c | 3 | 23 | 4 | 38 | 0.3952 | 0.0201 | 1.2391 |
| Rv1731 | gabD2 | 3 | 23 | 4 | 38 | 0.3952 | 0.0201 | 1.2391 |
| Rv1980c | mpt64 | 3 | 23 | 4 | 38 | 0.3952 | 0.0201 | 1.2391 |
| Rv2062c | cobN | 3 | 23 | 4 | 38 | 0.3952 | 0.0201 | 1.2391 |
| Rv2127 | ansP1 | 3 | 23 | 4 | 38 | 0.3952 | 0.0201 | 1.2391 |
| Rv2248 | Rv2248 | 3 | 23 | 4 | 38 | 0.3952 | 0.0201 | 1.2391 |
| Rv2409c | PE24 | 3 | 23 | 4 | 38 | 0.3952 | 0.0201 | 1.2391 |
| Rv2782c | pepR | 3 | 23 | 4 | 38 | 0.3952 | 0.0201 | 1.2391 |
| Rv2881c | cdsA | 3 | 23 | 4 | 38 | 0.3952 | 0.0201 | 1.2391 |
| Rv3015c | Rv3015c | 3 | 23 | 4 | 38 | 0.3952 | 0.0201 | 1.2391 |
| Rv3089 | fadD13 | 3 | 23 | 4 | 38 | 0.3952 | 0.0201 | 1.2391 |
| Rv3193c | Rv3193c | 3 | 23 | 4 | 38 | 0.3952 | 0.0201 | 1.2391 |
| Rv3199c | nudC | 3 | 23 | 4 | 38 | 0.3952 | 0.0201 | 1.2391 |
| Rv3220c | Rv3220c | 3 | 23 | 4 | 38 | 0.3952 | 0.0201 | 1.2391 |
| Rv3446c | Rv3446c | 3 | 23 | 4 | 38 | 0.3952 | 0.0201 | 1.2391 |
| Rv3538 | Rv3538 | 3 | 23 | 4 | 38 | 0.3952 | 0.0201 | 1.2391 |
| Rv3649 | Rv3649 | 3 | 23 | 4 | 38 | 0.3952 | 0.0201 | 1.2391 |
| Rv3772 | hisC2 | 3 | 23 | 4 | 38 | 0.3952 | 0.0201 | 1.2391 |
| Rv3839 | Rv3839 | 3 | 23 | 4 | 38 | 0.3952 | 0.0201 | 1.2391 |
| Rv0271c | fadE6 | 6 | 20 | 9 | 33 | 0.4367 | 0.0165 | 1.1000 |
| Rv0319 | pcp | 6 | 20 | 9 | 33 | 0.4367 | 0.0165 | 1.1000 |
| Rv0717 | rpsN | 6 | 20 | 9 | 33 | 0.4367 | 0.0165 | 1.1000 |
| Rv1056 | Rv1056 | 6 | 20 | 9 | 33 | 0.4367 | 0.0165 | 1.1000 |
| Rv1184c | Rv1184c | 6 | 20 | 9 | 33 | 0.4367 | 0.0165 | 1.1000 |
| Rv1615 | Rv1615 | 6 | 20 | 9 | 33 | 0.4367 | 0.0165 | 1.1000 |
| Rv1825 | Rv1825 | 6 | 20 | 9 | 33 | 0.4367 | 0.0165 | 1.1000 |
| Rv1897c | Rv1897c | 6 | 20 | 9 | 33 | 0.4367 | 0.0165 | 1.1000 |
| Rv2245 | kasA | 6 | 20 | 9 | 33 | 0.4367 | 0.0165 | 1.1000 |
| Rv2544 | lppB | 6 | 20 | 9 | 33 | 0.4367 | 0.0165 | 1.1000 |
| Rv2687c | Rv2687c | 6 | 20 | 9 | 33 | 0.4367 | 0.0165 | 1.1000 |

| Rv2911 | dacB2 | 6 | 20 | 9 | 33 | 0.4367 | 0.0165 | 1.1000 |
| --- | --- | --- | --- | --- | --- | --- | --- | --- |
| Rv2962c | Rv2962c | 6 | 20 | 9 | 33 | 0.4367 | 0.0165 | 1.1000 |
| Rv3088 | Rv3088 | 6 | 20 | 9 | 33 | 0.4367 | 0.0165 | 1.1000 |
| Rv3430c | PPE59 | 6 | 20 | 9 | 33 | 0.4367 | 0.0165 | 1.1000 |
| Rv3535c | Rv3535c | 6 | 20 | 9 | 33 | 0.4367 | 0.0165 | 1.1000 |
| Rv3843c | Rv3843c | 6 | 20 | 9 | 33 | 0.4367 | 0.0165 | 1.1000 |
| Rv3871 | Rv3871 | 6 | 20 | 9 | 33 | 0.4367 | 0.0165 | 1.1000 |
| Rv0016c | pbpA | 1 | 25 | 1 | 41 | 0.3641 | 0.0147 | 1.6400 |
| Rv0024 | Rv0024 | 1 | 25 | 1 | 41 | 0.3641 | 0.0147 | 1.6400 |
| Rv0087 | hycE | 1 | 25 | 1 | 41 | 0.3641 | 0.0147 | 1.6400 |
| Rv0099 | fadD10 | 1 | 25 | 1 | 41 | 0.3641 | 0.0147 | 1.6400 |
| Rv0124 | PE_PGRS2 | 1 | 25 | 1 | 41 | 0.3641 | 0.0147 | 1.6400 |
| Rv0163 | Rv0163 | 1 | 25 | 1 | 41 | 0.3641 | 0.0147 | 1.6400 |
| Rv0227c | Rv0227c | 1 | 25 | 1 | 41 | 0.3641 | 0.0147 | 1.6400 |
| Rv0255c | cobQ1 | 1 | 25 | 1 | 41 | 0.3641 | 0.0147 | 1.6400 |
| Rv0339c | Rv0339c | 1 | 25 | 1 | 41 | 0.3641 | 0.0147 | 1.6400 |
| Rv0363c | fba | 1 | 25 | 1 | 41 | 0.3641 | 0.0147 | 1.6400 |
| Rv0392c | ndhA | 1 | 25 | 1 | 41 | 0.3641 | 0.0147 | 1.6400 |
| Rv0422c | thiD | 1 | 25 | 1 | 41 | 0.3641 | 0.0147 | 1.6400 |
| Rv0567 | Rv0567 | 1 | 25 | 1 | 41 | 0.3641 | 0.0147 | 1.6400 |
| Rv0646c | lipG | 1 | 25 | 1 | 41 | 0.3641 | 0.0147 | 1.6400 |
| Rv0756c | Rv0756c | 1 | 25 | 1 | 41 | 0.3641 | 0.0147 | 1.6400 |
| Rv0783c | emrB | 1 | 25 | 1 | 41 | 0.3641 | 0.0147 | 1.6400 |
| Rv0796 | Rv0796 | 1 | 25 | 1 | 41 | 0.3641 | 0.0147 | 1.6400 |
| Rv0830 | Rv0830 | 1 | 25 | 1 | 41 | 0.3641 | 0.0147 | 1.6400 |
| Rv0870c | Rv0870c | 1 | 25 | 1 | 41 | 0.3641 | 0.0147 | 1.6400 |
| Rv0908 | ctpE | 1 | 25 | 1 | 41 | 0.3641 | 0.0147 | 1.6400 |
| Rv0923c | Rv0923c | 1 | 25 | 1 | 41 | 0.3641 | 0.0147 | 1.6400 |
| Rv0926c | Rv0926c | 1 | 25 | 1 | 41 | 0.3641 | 0.0147 | 1.6400 |
| Rv1005c | pabB | 1 | 25 | 1 | 41 | 0.3641 | 0.0147 | 1.6400 |
| Rv1061 | Rv1061 | 1 | 25 | 1 | 41 | 0.3641 | 0.0147 | 1.6400 |
| Rv1121 | zwf1 | 1 | 25 | 1 | 41 | 0.3641 | 0.0147 | 1.6400 |
| Rv1323 | fadA4 | 1 | 25 | 1 | 41 | 0.3641 | 0.0147 | 1.6400 |
| Rv1369c | Rv1369c | 1 | 25 | 1 | 41 | 0.3641 | 0.0147 | 1.6400 |

| Rv1423 | whiA | 1 | 25 | 1 | 41 | 0.3641 | 0.0147 | 1.6400 |
| --- | --- | --- | --- | --- | --- | --- | --- | --- |
| Rv1460 | Rv1460 | 1 | 25 | 1 | 41 | 0.3641 | 0.0147 | 1.6400 |
| Rv1470 | trxA | 1 | 25 | 1 | 41 | 0.3641 | 0.0147 | 1.6400 |
| Rv1506c | Rv1506c | 1 | 25 | 1 | 41 | 0.3641 | 0.0147 | 1.6400 |
| Rv1550 | fadD11.1 | 1 | 25 | 1 | 41 | 0.3641 | 0.0147 | 1.6400 |
| Rv1670 | Rv1670 | 1 | 25 | 1 | 41 | 0.3641 | 0.0147 | 1.6400 |
| Rv1744c | Rv1744c | 1 | 25 | 1 | 41 | 0.3641 | 0.0147 | 1.6400 |
| Rv1756c | Rv1756c | 1 | 25 | 1 | 41 | 0.3641 | 0.0147 | 1.6400 |
| Rv1817 | Rv1817 | 1 | 25 | 1 | 41 | 0.3641 | 0.0147 | 1.6400 |
| Rv1842c | Rv1842c | 1 | 25 | 1 | 41 | 0.3641 | 0.0147 | 1.6400 |
| Rv1866 | Rv1866 | 1 | 25 | 1 | 41 | 0.3641 | 0.0147 | 1.6400 |
| Rv1922 | Rv1922 | 1 | 25 | 1 | 41 | 0.3641 | 0.0147 | 1.6400 |
| Rv1963c | mce3R | 1 | 25 | 1 | 41 | 0.3641 | 0.0147 | 1.6400 |
| Rv2038c | Rv2038c | 1 | 25 | 1 | 41 | 0.3641 | 0.0147 | 1.6400 |
| Rv2073c | Rv2073c | 1 | 25 | 1 | 41 | 0.3641 | 0.0147 | 1.6400 |
| Rv2106 | Rv2106 | 1 | 25 | 1 | 41 | 0.3641 | 0.0147 | 1.6400 |
| Rv2129c | Rv2129c | 1 | 25 | 1 | 41 | 0.3641 | 0.0147 | 1.6400 |
| Rv2163c | pbpB | 1 | 25 | 1 | 41 | 0.3641 | 0.0147 | 1.6400 |
| Rv2167c | Rv2167c | 1 | 25 | 1 | 41 | 0.3641 | 0.0147 | 1.6400 |
| Rv2266 | cyp124 | 1 | 25 | 1 | 41 | 0.3641 | 0.0147 | 1.6400 |
| Rv2279 | Rv2279 | 1 | 25 | 1 | 41 | 0.3641 | 0.0147 | 1.6400 |
| Rv2280 | Rv2280 | 1 | 25 | 1 | 41 | 0.3641 | 0.0147 | 1.6400 |
| Rv2297 | Rv2297 | 1 | 25 | 1 | 41 | 0.3641 | 0.0147 | 1.6400 |
| Rv2314c | Rv2314c | 1 | 25 | 1 | 41 | 0.3641 | 0.0147 | 1.6400 |
| Rv2334 | cysK1 | 1 | 25 | 1 | 41 | 0.3641 | 0.0147 | 1.6400 |
| Rv2355 | Rv2355 | 1 | 25 | 1 | 41 | 0.3641 | 0.0147 | 1.6400 |
| Rv2376c | cfp2 | 1 | 25 | 1 | 41 | 0.3641 | 0.0147 | 1.6400 |
| Rv2408 | PE24 | 1 | 25 | 1 | 41 | 0.3641 | 0.0147 | 1.6400 |
| Rv2427c | proA | 1 | 25 | 1 | 41 | 0.3641 | 0.0147 | 1.6400 |
| Rv2552c | aroE | 1 | 25 | 1 | 41 | 0.3641 | 0.0147 | 1.6400 |
| Rv2592c | ruvB | 1 | 25 | 1 | 41 | 0.3641 | 0.0147 | 1.6400 |
| Rv2618 | Rv2618 | 1 | 25 | 1 | 41 | 0.3641 | 0.0147 | 1.6400 |
| Rv2649 | Rv2649 | 1 | 25 | 1 | 41 | 0.3641 | 0.0147 | 1.6400 |
| Rv2653c | Rv2653c | 1 | 25 | 1 | 41 | 0.3641 | 0.0147 | 1.6400 |

| Rv2670c | Rv2670c | 1 | 25 | 1 | 41 | 0.3641 | 0.0147 | 1.6400 |
| --- | --- | --- | --- | --- | --- | --- | --- | --- |
| Rv2684 | arsA | 1 | 25 | 1 | 41 | 0.3641 | 0.0147 | 1.6400 |
| Rv2725c | hflX | 1 | 25 | 1 | 41 | 0.3641 | 0.0147 | 1.6400 |
| Rv2751 | Rv2751 | 1 | 25 | 1 | 41 | 0.3641 | 0.0147 | 1.6400 |
| Rv2814c | Rv2814c | 1 | 25 | 1 | 41 | 0.3641 | 0.0147 | 1.6400 |
| Rv2858c | aldC | 1 | 25 | 1 | 41 | 0.3641 | 0.0147 | 1.6400 |
| Rv2871 | Rv2871 | 1 | 25 | 1 | 41 | 0.3641 | 0.0147 | 1.6400 |
| Rv2951c | Rv2951c | 1 | 25 | 1 | 41 | 0.3641 | 0.0147 | 1.6400 |
| Rv2973c | recG | 1 | 25 | 1 | 41 | 0.3641 | 0.0147 | 1.6400 |
| Rv3020c | esxS | 1 | 25 | 1 | 41 | 0.3641 | 0.0147 | 1.6400 |
| Rv3035 | Rv3035 | 1 | 25 | 1 | 41 | 0.3641 | 0.0147 | 1.6400 |
| Rv3043c | ctaD | 1 | 25 | 1 | 41 | 0.3641 | 0.0147 | 1.6400 |
| Rv3052c | nrdI | 1 | 25 | 1 | 41 | 0.3641 | 0.0147 | 1.6400 |
| Rv3067 | Rv3067 | 1 | 25 | 1 | 41 | 0.3641 | 0.0147 | 1.6400 |
| Rv3185 | Rv3185 | 1 | 25 | 1 | 41 | 0.3641 | 0.0147 | 1.6400 |
| Rv3187 | Rv3187 | 1 | 25 | 1 | 41 | 0.3641 | 0.0147 | 1.6400 |
| Rv3287c | rsbW | 1 | 25 | 1 | 41 | 0.3641 | 0.0147 | 1.6400 |
| Rv3326 | Rv3326 | 1 | 25 | 1 | 41 | 0.3641 | 0.0147 | 1.6400 |
| Rv3378c | Rv3378c | 1 | 25 | 1 | 41 | 0.3641 | 0.0147 | 1.6400 |
| Rv3395A | Rv3395A | 1 | 25 | 1 | 41 | 0.3641 | 0.0147 | 1.6400 |
| Rv3403c | Rv3403c | 1 | 25 | 1 | 41 | 0.3641 | 0.0147 | 1.6400 |
| Rv3436c | glmS | 1 | 25 | 1 | 41 | 0.3641 | 0.0147 | 1.6400 |
| Rv3475 | Rv3475 | 1 | 25 | 1 | 41 | 0.3641 | 0.0147 | 1.6400 |
| Rv3481c | Rv3481c | 1 | 25 | 1 | 41 | 0.3641 | 0.0147 | 1.6400 |
| Rv3570c | Rv3570c | 1 | 25 | 1 | 41 | 0.3641 | 0.0147 | 1.6400 |
| Rv3694c | Rv3694c | 1 | 25 | 1 | 41 | 0.3641 | 0.0147 | 1.6400 |
| Rv3696c | glpK | 1 | 25 | 1 | 41 | 0.3641 | 0.0147 | 1.6400 |
| Rv3741c | Rv3741c | 1 | 25 | 1 | 41 | 0.3641 | 0.0147 | 1.6400 |
| Rv3743c | ctpJ | 1 | 25 | 1 | 41 | 0.3641 | 0.0147 | 1.6400 |
| Rv3763 | lpqH | 1 | 25 | 1 | 41 | 0.3641 | 0.0147 | 1.6400 |
| Rv3796 | Rv3796 | 1 | 25 | 1 | 41 | 0.3641 | 0.0147 | 1.6400 |
| Rv3804c | fbpA | 1 | 25 | 1 | 41 | 0.3641 | 0.0147 | 1.6400 |
| Rv3806c | Rv3806c | 1 | 25 | 1 | 41 | 0.3641 | 0.0147 | 1.6400 |
| Rv3909 | Rv3909 | 1 | 25 | 1 | 41 | 0.3641 | 0.0147 | 1.6400 |

| Rv0035 | fadD34 | 9 | 17 | 14 | 28 | 0.4568 | 0.0128 | 1.0588 |
| --- | --- | --- | --- | --- | --- | --- | --- | --- |
| Rv1196 | PPE18 | 9 | 17 | 14 | 28 | 0.4568 | 0.0128 | 1.0588 |
| Rv3159c | PPE53 | 9 | 17 | 14 | 28 | 0.4568 | 0.0128 | 1.0588 |
| Rv0071 | Rv0071 | 4 | 22 | 6 | 36 | 0.4505 | 0.0110 | 1.0909 |
| Rv1286 | cysN | 4 | 22 | 6 | 36 | 0.4505 | 0.0110 | 1.0909 |
| Rv2905 | lppW | 4 | 22 | 6 | 36 | 0.4505 | 0.0110 | 1.0909 |
| Rv3449 | mycP4 | 4 | 22 | 6 | 36 | 0.4505 | 0.0110 | 1.0909 |
| Rv3497c | mce4C | 4 | 22 | 6 | 36 | 0.4505 | 0.0110 | 1.0909 |
| Rv3590c | PE_PGRS58 | 4 | 22 | 6 | 36 | 0.4505 | 0.0110 | 1.0909 |
| Rv0233 | nrdB | 25 | 1 | 40 | 2 | 0.4291 | 0.0092 | 1.2500 |
| Rv2933 | ppsC | 12 | 14 | 19 | 23 | 0.4706 | 0.0092 | 1.0376 |
| Rv3882c | Rv3882c | 12 | 14 | 19 | 23 | 0.4706 | 0.0092 | 1.0376 |
| Rv1041c | Rv1041c | 7 | 19 | 11 | 31 | 0.4735 | 0.0073 | 1.0383 |
| Rv0495c | Rv0495c | 2 | 24 | 3 | 39 | 0.4664 | 0.0055 | 1.0833 |
| Rv0813c | Rv0813c | 2 | 24 | 3 | 39 | 0.4664 | 0.0055 | 1.0833 |
| Rv0957 | purH | 2 | 24 | 3 | 39 | 0.4664 | 0.0055 | 1.0833 |
| Rv1030 | kdpB | 2 | 24 | 3 | 39 | 0.4664 | 0.0055 | 1.0833 |
| Rv1360 | Rv1360 | 2 | 24 | 3 | 39 | 0.4664 | 0.0055 | 1.0833 |
| Rv1434 | Rv1434 | 2 | 24 | 3 | 39 | 0.4664 | 0.0055 | 1.0833 |
| Rv1511 | gmdA | 2 | 24 | 3 | 39 | 0.4664 | 0.0055 | 1.0833 |
| Rv1718 | Rv1718 | 2 | 24 | 3 | 39 | 0.4664 | 0.0055 | 1.0833 |
| Rv1770 | Rv1770 | 2 | 24 | 3 | 39 | 0.4664 | 0.0055 | 1.0833 |
| Rv1888c | Rv1888c | 2 | 24 | 3 | 39 | 0.4664 | 0.0055 | 1.0833 |
| Rv2158c | murE | 2 | 24 | 3 | 39 | 0.4664 | 0.0055 | 1.0833 |
| Rv2213 | pepB | 2 | 24 | 3 | 39 | 0.4664 | 0.0055 | 1.0833 |
| Rv2241 | aceE | 2 | 24 | 3 | 39 | 0.4664 | 0.0055 | 1.0833 |
| Rv2285 | Rv2285 | 2 | 24 | 3 | 39 | 0.4664 | 0.0055 | 1.0833 |
| Rv2323c | Rv2323c | 2 | 24 | 3 | 39 | 0.4664 | 0.0055 | 1.0833 |
| Rv2381c | mbtD | 2 | 24 | 3 | 39 | 0.4664 | 0.0055 | 1.0833 |
| Rv2877c | Rv2877c | 2 | 24 | 3 | 39 | 0.4664 | 0.0055 | 1.0833 |
| Rv2885c | Rv2885c | 2 | 24 | 3 | 39 | 0.4664 | 0.0055 | 1.0833 |
| Rv2946c | pks1 | 2 | 24 | 3 | 39 | 0.4664 | 0.0055 | 1.0833 |
| Rv3080c | pknK | 2 | 24 | 3 | 39 | 0.4664 | 0.0055 | 1.0833 |
| Rv3300c | Rv3300c | 2 | 24 | 3 | 39 | 0.4664 | 0.0055 | 1.0833 |

| Rv3489 | Rv3489 | 2 | 24 | 3 | 39 | 0.4664 | 0.0055 | 1.0833 |
| --- | --- | --- | --- | --- | --- | --- | --- | --- |
| Rv3850 | Rv3850 | 2 | 24 | 3 | 39 | 0.4664 | 0.0055 | 1.0833 |
| Rv0371c | Rv0371c | 5 | 21 | 8 | 34 | 0.4926 | 0.0018 | 1.0119 |
| Rv0750 | Rv0750 | 5 | 21 | 8 | 34 | 0.4926 | 0.0018 | 1.0119 |
| Rv0751c | mmsB | 5 | 21 | 8 | 34 | 0.4926 | 0.0018 | 1.0119 |
| Rv1552 | frdA | 5 | 21 | 8 | 34 | 0.4926 | 0.0018 | 1.0119 |
| Rv2347c | esxP | 5 | 21 | 8 | 34 | 0.4926 | 0.0018 | 1.0119 |
| Rv2578c | Rv2578c | 5 | 21 | 8 | 34 | 0.4926 | 0.0018 | 1.0119 |
| Rv3416 | whiB3 | 5 | 21 | 8 | 34 | 0.4926 | 0.0018 | 1.0119 |
| Rv3619c | esxV | 5 | 21 | 8 | 34 | 0.4926 | 0.0018 | 1.0119 |
| Rv1760 | Rv1760 | 18 | 8 | 29 | 13 | 0.4937 | 0.0018 | 1.0086 |
| Rv0003 | recF | 26 | 0 | 42 | 0 | . | 0.0000 | ND |
| Rv0006 | gyrA | 26 | 0 | 42 | 0 | . | 0.0000 | ND |
| Rv0008c | Rv0008c | 26 | 0 | 42 | 0 | . | 0.0000 | ND |
| Rv0012 | Rv0012 | 26 | 0 | 42 | 0 | . | 0.0000 | ND |
| Rv0018c | ppp | 26 | 0 | 42 | 0 | . | 0.0000 | ND |
| Rv0058 | dnaB | 26 | 0 | 42 | 0 | . | 0.0000 | ND |
| Rv0068 | Rv0068 | 26 | 0 | 42 | 0 | . | 0.0000 | ND |
| Rv0103c | ctpB | 26 | 0 | 42 | 0 | . | 0.0000 | ND |
| Rv0120c | fusA2 | 26 | 0 | 42 | 0 | . | 0.0000 | ND |
| Rv0127 | Rv0127 | 26 | 0 | 42 | 0 | . | 0.0000 | ND |
| Rv0174 | mce1F | 26 | 0 | 42 | 0 | . | 0.0000 | ND |
| Rv0192 | Rv0192 | 26 | 0 | 42 | 0 | . | 0.0000 | ND |
| Rv0193c | Rv0193c | 26 | 0 | 42 | 0 | . | 0.0000 | ND |
| Rv0194 | Rv0194 | 26 | 0 | 42 | 0 | . | 0.0000 | ND |
| Rv0197 | Rv0197 | 26 | 0 | 42 | 0 | . | 0.0000 | ND |
| Rv0204c | Rv0204c | 26 | 0 | 42 | 0 | . | 0.0000 | ND |
| Rv0259c | Rv0259c | 26 | 0 | 42 | 0 | . | 0.0000 | ND |
| Rv0284 | Rv0284 | 26 | 0 | 42 | 0 | . | 0.0000 | ND |
| Rv0292 | Rv0292 | 26 | 0 | 42 | 0 | . | 0.0000 | ND |
| Rv0315 | Rv0315 | 26 | 0 | 42 | 0 | . | 0.0000 | ND |
| Rv0318c | Rv0318c | 26 | 0 | 42 | 0 | . | 0.0000 | ND |
| Rv0338c | Rv0338c | 26 | 0 | 42 | 0 | . | 0.0000 | ND |
| Rv0388c | PPE9 | 26 | 0 | 42 | 0 | . | 0.0000 | ND |

| Rv0395 | Rv0395 | 26 | 0 | 42 | 0 | . | 0.0000 | ND |
| --- | --- | --- | --- | --- | --- | --- | --- | --- |
| Rv0417 | thiG | 26 | 0 | 42 | 0 | . | 0.0000 | ND |
| Rv0425c | ctpH | 26 | 0 | 42 | 0 | . | 0.0000 | ND |
| Rv0442c | PPE10 | 26 | 0 | 42 | 0 | . | 0.0000 | ND |
| Rv0473 | Rv0473 | 26 | 0 | 42 | 0 | . | 0.0000 | ND |
| Rv0507 | mmpL2 | 26 | 0 | 42 | 0 | . | 0.0000 | ND |
| Rv0556 | Rv0556 | 26 | 0 | 42 | 0 | . | 0.0000 | ND |
| Rv0589 | mce2A | 26 | 0 | 42 | 0 | . | 0.0000 | ND |
| Rv0658c | Rv0658c | 26 | 0 | 42 | 0 | . | 0.0000 | ND |
| Rv0676c | mmpL5 | 26 | 0 | 42 | 0 | . | 0.0000 | ND |
| Rv0758 | phoR | 26 | 0 | 42 | 0 | . | 0.0000 | ND |
| Rv0800 | pepC | 26 | 0 | 42 | 0 | . | 0.0000 | ND |
| Rv0812 | Rv0812 | 26 | 0 | 42 | 0 | . | 0.0000 | ND |
| Rv0829 | Rv0829 | 26 | 0 | 42 | 0 | . | 0.0000 | ND |
| Rv0859 | fadA | 26 | 0 | 42 | 0 | . | 0.0000 | ND |
| Rv0881 | Rv0881 | 26 | 0 | 42 | 0 | . | 0.0000 | ND |
| Rv0890c | Rv0890c | 26 | 0 | 42 | 0 | . | 0.0000 | ND |
| Rv0891c | Rv0891c | 26 | 0 | 42 | 0 | . | 0.0000 | ND |
| Rv0930 | pstA1 | 26 | 0 | 42 | 0 | . | 0.0000 | ND |
| Rv0964c | Rv0964c | 26 | 0 | 42 | 0 | . | 0.0000 | ND |
| Rv0966c | Rv0966c | 26 | 0 | 42 | 0 | . | 0.0000 | ND |
| Rv0983 | pepD | 26 | 0 | 42 | 0 | . | 0.0000 | ND |
| Rv0993 | galU | 26 | 0 | 42 | 0 | . | 0.0000 | ND |
| Rv1007c | metG | 26 | 0 | 42 | 0 | . | 0.0000 | ND |
| Rv1008 | tatD | 26 | 0 | 42 | 0 | . | 0.0000 | ND |
| Rv1028c | kdpD | 26 | 0 | 42 | 0 | . | 0.0000 | ND |
| Rv1093 | glyA | 26 | 0 | 42 | 0 | . | 0.0000 | ND |
| Rv1154c | Rv1154c | 26 | 0 | 42 | 0 | . | 0.0000 | ND |
| Rv1180 | pks3 | 26 | 0 | 42 | 0 | . | 0.0000 | ND |
| Rv1186c | Rv1186c | 26 | 0 | 42 | 0 | . | 0.0000 | ND |
| Rv1230c | Rv1230c | 26 | 0 | 42 | 0 | . | 0.0000 | ND |
| Rv1232c | Rv1232c | 26 | 0 | 42 | 0 | . | 0.0000 | ND |
| Rv1239c | corA | 26 | 0 | 42 | 0 | . | 0.0000 | ND |
| Rv1266c | pknH | 26 | 0 | 42 | 0 | . | 0.0000 | ND |

| Rv1300 | hemK | 26 | 0 | 42 | 0 | . | 0.0000 | ND |
| --- | --- | --- | --- | --- | --- | --- | --- | --- |
| Rv1320c | Rv1320c | 26 | 0 | 42 | 0 | . | 0.0000 | ND |
| Rv1321 | Rv1321 | 26 | 0 | 42 | 0 | . | 0.0000 | ND |
| Rv1330c | Rv1330c | 26 | 0 | 42 | 0 | . | 0.0000 | ND |
| Rv1364c | Rv1364c | 26 | 0 | 42 | 0 | . | 0.0000 | ND |
| Rv1374c | Rv1374c | 26 | 0 | 42 | 0 | . | 0.0000 | ND |
| Rv1378c | Rv1378c | 26 | 0 | 42 | 0 | . | 0.0000 | ND |
| Rv1453 | Rv1453 | 26 | 0 | 42 | 0 | . | 0.0000 | ND |
| Rv1462 | Rv1462 | 26 | 0 | 42 | 0 | . | 0.0000 | ND |
| Rv1554 | frdC | 26 | 0 | 42 | 0 | . | 0.0000 | ND |
| Rv1570 | bioD | 26 | 0 | 42 | 0 | . | 0.0000 | ND |
| Rv1602 | hisH | 26 | 0 | 42 | 0 | . | 0.0000 | ND |
| Rv1618 | tesB1 | 26 | 0 | 42 | 0 | . | 0.0000 | ND |
| Rv1647 | Rv1647 | 26 | 0 | 42 | 0 | . | 0.0000 | ND |
| Rv1662 | pks8 | 26 | 0 | 42 | 0 | . | 0.0000 | ND |
| Rv1704c | cycA | 26 | 0 | 42 | 0 | . | 0.0000 | ND |
| Rv1716 | Rv1716 | 26 | 0 | 42 | 0 | . | 0.0000 | ND |
| Rv1739c | Rv1739c | 26 | 0 | 42 | 0 | . | 0.0000 | ND |
| Rv1783 | Rv1783 | 26 | 0 | 42 | 0 | . | 0.0000 | ND |
| Rv1807 | PPE31 | 26 | 0 | 42 | 0 | . | 0.0000 | ND |
| Rv1812c | Rv1812c | 26 | 0 | 42 | 0 | . | 0.0000 | ND |
| Rv1815 | Rv1815 | 26 | 0 | 42 | 0 | . | 0.0000 | ND |
| Rv1895 | Rv1895 | 26 | 0 | 42 | 0 | . | 0.0000 | ND |
| Rv1925 | fadD31 | 26 | 0 | 42 | 0 | . | 0.0000 | ND |
| Rv1971 | mce3F | 26 | 0 | 42 | 0 | . | 0.0000 | ND |
| Rv1979c | Rv1979c | 26 | 0 | 42 | 0 | . | 0.0000 | ND |
| Rv2017 | Rv2017 | 26 | 0 | 42 | 0 | . | 0.0000 | ND |
| Rv2024c | Rv2024c | 26 | 0 | 42 | 0 | . | 0.0000 | ND |
| Rv2083 | Rv2083 | 26 | 0 | 42 | 0 | . | 0.0000 | ND |
| Rv2125 | Rv2125 | 26 | 0 | 42 | 0 | . | 0.0000 | ND |
| Rv2155c | murD | 26 | 0 | 42 | 0 | . | 0.0000 | ND |
| Rv2337c | Rv2337c | 26 | 0 | 42 | 0 | . | 0.0000 | ND |
| Rv2377c | mbtH | 26 | 0 | 42 | 0 | . | 0.0000 | ND |
| Rv2436 | rbsK | 26 | 0 | 42 | 0 | . | 0.0000 | ND |

| Rv2450c | rpfE | 26 | 0 | 42 | 0 | . | 0.0000 | ND |
| --- | --- | --- | --- | --- | --- | --- | --- | --- |
| Rv2458 | mmuM | 26 | 0 | 42 | 0 | . | 0.0000 | ND |
| Rv2476c | gdh | 26 | 0 | 42 | 0 | . | 0.0000 | ND |
| Rv2482c | plsB2 | 26 | 0 | 42 | 0 | . | 0.0000 | ND |
| Rv2495c | pdhC | 26 | 0 | 42 | 0 | . | 0.0000 | ND |
| Rv2513 | Rv2513 | 26 | 0 | 42 | 0 | . | 0.0000 | ND |
| Rv2542 | Rv2542 | 26 | 0 | 42 | 0 | . | 0.0000 | ND |
| Rv2567 | Rv2567 | 26 | 0 | 42 | 0 | . | 0.0000 | ND |
| Rv2584c | apt | 26 | 0 | 42 | 0 | . | 0.0000 | ND |
| Rv2585c | Rv2585c | 26 | 0 | 42 | 0 | . | 0.0000 | ND |
| Rv2611c | Rv2611c | 26 | 0 | 42 | 0 | . | 0.0000 | ND |
| Rv2702 | ppgK | 26 | 0 | 42 | 0 | . | 0.0000 | ND |
| Rv2771c | Rv2771c | 26 | 0 | 42 | 0 | . | 0.0000 | ND |
| Rv2794c | Rv2794c | 26 | 0 | 42 | 0 | . | 0.0000 | ND |
| Rv2812 | Rv2812 | 26 | 0 | 42 | 0 | . | 0.0000 | ND |
| Rv2830c | Rv2830c | 26 | 0 | 42 | 0 | . | 0.0000 | ND |
| Rv2874 | dipZ | 26 | 0 | 42 | 0 | . | 0.0000 | ND |
| Rv2896c | Rv2896c | 26 | 0 | 42 | 0 | . | 0.0000 | ND |
| Rv2917 | Rv2917 | 26 | 0 | 42 | 0 | . | 0.0000 | ND |
| Rv2931 | ppsA | 26 | 0 | 42 | 0 | . | 0.0000 | ND |
| Rv2947c | pks15 | 26 | 0 | 42 | 0 | . | 0.0000 | ND |
| Rv2982c | gpsA | 26 | 0 | 42 | 0 | . | 0.0000 | ND |
| Rv2999 | lppY | 26 | 0 | 42 | 0 | . | 0.0000 | ND |
| Rv3011c | gatA | 26 | 0 | 42 | 0 | . | 0.0000 | ND |
| Rv3062 | ligB | 26 | 0 | 42 | 0 | . | 0.0000 | ND |
| Rv3077 | Rv3077 | 26 | 0 | 42 | 0 | . | 0.0000 | ND |
| Rv3121 | cyp141 | 26 | 0 | 42 | 0 | . | 0.0000 | ND |
| Rv3137 | Rv3137 | 26 | 0 | 42 | 0 | . | 0.0000 | ND |
| Rv3144c | PPE52 | 26 | 0 | 42 | 0 | . | 0.0000 | ND |
| Rv3151 | nuoG | 26 | 0 | 42 | 0 | . | 0.0000 | ND |
| Rv3204 | Rv3204 | 26 | 0 | 42 | 0 | . | 0.0000 | ND |
| Rv3213c | Rv3213c | 26 | 0 | 42 | 0 | . | 0.0000 | ND |
| Rv3303c | lpdA | 26 | 0 | 42 | 0 | . | 0.0000 | ND |
| Rv3317 | sdhD | 26 | 0 | 42 | 0 | . | 0.0000 | ND |

| Rv3328c | sigJ | 26 | 0 | 42 | 0 | . | 0.0000 | ND |
| --- | --- | --- | --- | --- | --- | --- | --- | --- |
| Rv3331 | sugI | 26 | 0 | 42 | 0 | . | 0.0000 | ND |
| Rv3399 | Rv3399 | 26 | 0 | 42 | 0 | . | 0.0000 | ND |
| Rv3408 | Rv3408 | 26 | 0 | 42 | 0 | . | 0.0000 | ND |
| Rv3479 | Rv3479 | 26 | 0 | 42 | 0 | . | 0.0000 | ND |
| Rv3516 | echA19 | 26 | 0 | 42 | 0 | . | 0.0000 | ND |
| Rv3522 | ltp4 | 26 | 0 | 42 | 0 | . | 0.0000 | ND |
| Rv3593 | lpqF | 26 | 0 | 42 | 0 | . | 0.0000 | ND |
| Rv3616c | Rv3616c | 26 | 0 | 42 | 0 | . | 0.0000 | ND |
| Rv3711c | dnaQ | 26 | 0 | 42 | 0 | . | 0.0000 | ND |
| Rv3719 | Rv3719 | 26 | 0 | 42 | 0 | . | 0.0000 | ND |
| Rv3737 | Rv3737 | 26 | 0 | 42 | 0 | . | 0.0000 | ND |
| Rv3764c | Rv3764c | 26 | 0 | 42 | 0 | . | 0.0000 | ND |
| Rv3776 | Rv3776 | 26 | 0 | 42 | 0 | . | 0.0000 | ND |
| Rv3827c | Rv3827c | 26 | 0 | 42 | 0 | . | 0.0000 | ND |
| Rv3872 | PE35 | 26 | 0 | 42 | 0 | . | 0.0000 | ND |
| Rv3896c | Rv3896c | 26 | 0 | 42 | 0 | . | 0.0000 | ND |
| Rv3919c | gidB | 26 | 0 | 42 | 0 | . | 0.0000 | ND |
| Rv1023 | eno | 8 | 18 | 13 | 29 | 0.5063 | -0.0018 | 0.9915 |
| Rv2059 | Rv2059 | 8 | 18 | 13 | 29 | 0.5063 | -0.0018 | 0.9915 |
| Rv3174 | Rv3174 | 8 | 18 | 13 | 29 | 0.5063 | -0.0018 | 0.9915 |
| Rv3911 | sigM | 8 | 18 | 13 | 29 | 0.5063 | -0.0018 | 0.9915 |
| Rv0166 | fadD5 | 3 | 23 | 5 | 37 | 0.5182 | -0.0037 | 0.9652 |
| Rv0210 | Rv0210 | 3 | 23 | 5 | 37 | 0.5182 | -0.0037 | 0.9652 |
| Rv0237 | lpqI | 3 | 23 | 5 | 37 | 0.5182 | -0.0037 | 0.9652 |
| Rv0252 | nirB | 3 | 23 | 5 | 37 | 0.5182 | -0.0037 | 0.9652 |
| Rv1048c | Rv1048c | 3 | 23 | 5 | 37 | 0.5182 | -0.0037 | 0.9652 |
| Rv1336 | cysM | 3 | 23 | 5 | 37 | 0.5182 | -0.0037 | 0.9652 |
| Rv1373 | Rv1373 | 3 | 23 | 5 | 37 | 0.5182 | -0.0037 | 0.9652 |
| Rv1565c | Rv1565c | 3 | 23 | 5 | 37 | 0.5182 | -0.0037 | 0.9652 |
| Rv1918c | PPE35 | 3 | 23 | 5 | 37 | 0.5182 | -0.0037 | 0.9652 |
| Rv1939 | Rv1939 | 3 | 23 | 5 | 37 | 0.5182 | -0.0037 | 0.9652 |
| Rv2380c | mbtE | 3 | 23 | 5 | 37 | 0.5182 | -0.0037 | 0.9652 |
| Rv2471 | aglA | 3 | 23 | 5 | 37 | 0.5182 | -0.0037 | 0.9652 |

| Rv2490c | PE_PGRS43 | 3 | 23 | 5 | 37 | 0.5182 | -0.0037 | 0.9652 |
| --- | --- | --- | --- | --- | --- | --- | --- | --- |
| Rv2724c | fadE20 | 3 | 23 | 5 | 37 | 0.5182 | -0.0037 | 0.9652 |
| Rv3074 | Rv3074 | 3 | 23 | 5 | 37 | 0.5182 | -0.0037 | 0.9652 |
| Rv3140 | fadE23 | 3 | 23 | 5 | 37 | 0.5182 | -0.0037 | 0.9652 |
| Rv0359 | Rv0359 | 6 | 20 | 10 | 32 | 0.5276 | -0.0073 | 0.9600 |
| Rv0557 | pimB | 6 | 20 | 10 | 32 | 0.5276 | -0.0073 | 0.9600 |
| Rv0816c | thiX | 6 | 20 | 10 | 32 | 0.5276 | -0.0073 | 0.9600 |
| Rv0914c | Rv0914c | 6 | 20 | 10 | 32 | 0.5276 | -0.0073 | 0.9600 |
| Rv1039c | PPE15 | 6 | 20 | 10 | 32 | 0.5276 | -0.0073 | 0.9600 |
| Rv1108c | xseA | 6 | 20 | 10 | 32 | 0.5276 | -0.0073 | 0.9600 |
| Rv1316c | ogt | 6 | 20 | 10 | 32 | 0.5276 | -0.0073 | 0.9600 |
| Rv1386 | PE15 | 6 | 20 | 10 | 32 | 0.5276 | -0.0073 | 0.9600 |
| Rv1652 | argC | 6 | 20 | 10 | 32 | 0.5276 | -0.0073 | 0.9600 |
| Rv1811 | mgtC | 6 | 20 | 10 | 32 | 0.5276 | -0.0073 | 0.9600 |
| Rv1878 | glnA3 | 6 | 20 | 10 | 32 | 0.5276 | -0.0073 | 0.9600 |
| Rv2573 | Rv2573 | 6 | 20 | 10 | 32 | 0.5276 | -0.0073 | 0.9600 |
| Rv2694c | Rv2694c | 6 | 20 | 10 | 32 | 0.5276 | -0.0073 | 0.9600 |
| Rv2697c | dut | 6 | 20 | 10 | 32 | 0.5276 | -0.0073 | 0.9600 |
| Rv2748c | ftsK | 6 | 20 | 10 | 32 | 0.5276 | -0.0073 | 0.9600 |
| Rv3084 | lipR | 6 | 20 | 10 | 32 | 0.5276 | -0.0073 | 0.9600 |
| Rv3125c | PPE49 | 6 | 20 | 10 | 32 | 0.5276 | -0.0073 | 0.9600 |
| Rv3200c | Rv3200c | 6 | 20 | 10 | 32 | 0.5276 | -0.0073 | 0.9600 |
| Rv3534c | Rv3534c | 6 | 20 | 10 | 32 | 0.5276 | -0.0073 | 0.9600 |
| Rv3545c | cyp125 | 6 | 20 | 10 | 32 | 0.5276 | -0.0073 | 0.9600 |
| Rv3873 | PPE68 | 6 | 20 | 10 | 32 | 0.5276 | -0.0073 | 0.9600 |
| Rv3874 | esxB | 6 | 20 | 10 | 32 | 0.5276 | -0.0073 | 0.9600 |
| Rv2101 | helZ | 14 | 12 | 23 | 19 | 0.5294 | -0.0092 | 0.9638 |
| Rv0075 | Rv0075 | 1 | 25 | 2 | 40 | 0.5709 | -0.0092 | 0.8000 |
| Rv0211 | pckA | 1 | 25 | 2 | 40 | 0.5709 | -0.0092 | 0.8000 |
| Rv0212c | nadR | 1 | 25 | 2 | 40 | 0.5709 | -0.0092 | 0.8000 |
| Rv0213c | Rv0213c | 1 | 25 | 2 | 40 | 0.5709 | -0.0092 | 0.8000 |
| Rv0266c | oplA | 1 | 25 | 2 | 40 | 0.5709 | -0.0092 | 0.8000 |
| Rv0669c | Rv0669c | 1 | 25 | 2 | 40 | 0.5709 | -0.0092 | 0.8000 |
| Rv1050 | Rv1050 | 1 | 25 | 2 | 40 | 0.5709 | -0.0092 | 0.8000 |

| Rv1104 | Rv1104 | 1 | 25 | 2 | 40 | 0.5709 | -0.0092 | 0.8000 |
| --- | --- | --- | --- | --- | --- | --- | --- | --- |
| Rv1179c | Rv1179c | 1 | 25 | 2 | 40 | 0.5709 | -0.0092 | 0.8000 |
| Rv1273c | Rv1273c | 1 | 25 | 2 | 40 | 0.5709 | -0.0092 | 0.8000 |
| Rv1348 | Rv1348 | 1 | 25 | 2 | 40 | 0.5709 | -0.0092 | 0.8000 |
| Rv1391 | dfp | 1 | 25 | 2 | 40 | 0.5709 | -0.0092 | 0.8000 |
| Rv1587c | Rv1587c | 1 | 25 | 2 | 40 | 0.5709 | -0.0092 | 0.8000 |
| Rv1714 | Rv1714 | 1 | 25 | 2 | 40 | 0.5709 | -0.0092 | 0.8000 |
| Rv1724c | Rv1724c | 1 | 25 | 2 | 40 | 0.5709 | -0.0092 | 0.8000 |
| Rv1743 | pknE | 1 | 25 | 2 | 40 | 0.5709 | -0.0092 | 0.8000 |
| Rv1881c | lppE | 1 | 25 | 2 | 40 | 0.5709 | -0.0092 | 0.8000 |
| Rv1902c | nanT | 1 | 25 | 2 | 40 | 0.5709 | -0.0092 | 0.8000 |
| Rv2077A | Rv2077A | 1 | 25 | 2 | 40 | 0.5709 | -0.0092 | 0.8000 |
| Rv2138 | lppL | 1 | 25 | 2 | 40 | 0.5709 | -0.0092 | 0.8000 |
| Rv2211c | gcvT | 1 | 25 | 2 | 40 | 0.5709 | -0.0092 | 0.8000 |
| Rv2267c | Rv2267c | 1 | 25 | 2 | 40 | 0.5709 | -0.0092 | 0.8000 |
| Rv2281 | pitB | 1 | 25 | 2 | 40 | 0.5709 | -0.0092 | 0.8000 |
| Rv2329c | narK1 | 1 | 25 | 2 | 40 | 0.5709 | -0.0092 | 0.8000 |
| Rv2586c | secF | 1 | 25 | 2 | 40 | 0.5709 | -0.0092 | 0.8000 |
| Rv2591 | PE_PGRS44 | 1 | 25 | 2 | 40 | 0.5709 | -0.0092 | 0.8000 |
| Rv2678c | hemE | 1 | 25 | 2 | 40 | 0.5709 | -0.0092 | 0.8000 |
| Rv2713 | sthA | 1 | 25 | 2 | 40 | 0.5709 | -0.0092 | 0.8000 |
| Rv2797c | Rv2797c | 1 | 25 | 2 | 40 | 0.5709 | -0.0092 | 0.8000 |
| Rv3197 | Rv3197 | 1 | 25 | 2 | 40 | 0.5709 | -0.0092 | 0.8000 |
| Rv3373 | echA18 | 1 | 25 | 2 | 40 | 0.5709 | -0.0092 | 0.8000 |
| Rv3401 | Rv3401 | 1 | 25 | 2 | 40 | 0.5709 | -0.0092 | 0.8000 |
| Rv3597c | lsr2 | 1 | 25 | 2 | 40 | 0.5709 | -0.0092 | 0.8000 |
| Rv3799c | accD4 | 1 | 25 | 2 | 40 | 0.5709 | -0.0092 | 0.8000 |
| Rv3822 | Rv3822 | 1 | 25 | 2 | 40 | 0.5709 | -0.0092 | 0.8000 |
| Rv3859c | gltB | 1 | 25 | 2 | 40 | 0.5709 | -0.0092 | 0.8000 |
| Rv3899c | Rv3899c | 1 | 25 | 2 | 40 | 0.5709 | -0.0092 | 0.8000 |
| Rv3920c | Rv3920c | 1 | 25 | 2 | 40 | 0.5709 | -0.0092 | 0.8000 |
| Rv2769c | PE27 | 9 | 17 | 15 | 27 | 0.5367 | -0.0110 | 0.9529 |
| Rv3901c | Rv3901c | 9 | 17 | 15 | 27 | 0.5367 | -0.0110 | 0.9529 |
| Rv0922 | Rv0922 | 4 | 22 | 7 | 35 | 0.5555 | -0.0128 | 0.9091 |

| Rv1130 | Rv1130 | 4 | 22 | 7 | 35 | 0.5555 | -0.0128 | 0.9091 |
| --- | --- | --- | --- | --- | --- | --- | --- | --- |
| Rv2828c | Rv2828c | 4 | 22 | 7 | 35 | 0.5555 | -0.0128 | 0.9091 |
| Rv3476c | kgtP | 4 | 22 | 7 | 35 | 0.5555 | -0.0128 | 0.9091 |
| Rv0407 | fgd1 | 7 | 19 | 12 | 30 | 0.5585 | -0.0165 | 0.9211 |
| Rv0688 | Rv0688 | 7 | 19 | 12 | 30 | 0.5585 | -0.0165 | 0.9211 |
| Rv0885 | Rv0885 | 7 | 19 | 12 | 30 | 0.5585 | -0.0165 | 0.9211 |
| Rv1162 | narH | 7 | 19 | 12 | 30 | 0.5585 | -0.0165 | 0.9211 |
| Rv1479 | moxR1 | 7 | 19 | 12 | 30 | 0.5585 | -0.0165 | 0.9211 |
| Rv1588c | Rv1588c | 7 | 19 | 12 | 30 | 0.5585 | -0.0165 | 0.9211 |
| Rv1709 | Rv1709 | 7 | 19 | 12 | 30 | 0.5585 | -0.0165 | 0.9211 |
| Rv1759c | wag22 | 7 | 19 | 12 | 30 | 0.5585 | -0.0165 | 0.9211 |
| Rv1773c | Rv1773c | 7 | 19 | 12 | 30 | 0.5585 | -0.0165 | 0.9211 |
| Rv1800 | PPE28 | 7 | 19 | 12 | 30 | 0.5585 | -0.0165 | 0.9211 |
| Rv1957 | Rv1957 | 7 | 19 | 12 | 30 | 0.5585 | -0.0165 | 0.9211 |
| Rv2002 | fabG3 | 7 | 19 | 12 | 30 | 0.5585 | -0.0165 | 0.9211 |
| Rv2016 | Rv2016 | 7 | 19 | 12 | 30 | 0.5585 | -0.0165 | 0.9211 |
| Rv2631 | Rv2631 | 7 | 19 | 12 | 30 | 0.5585 | -0.0165 | 0.9211 |
| Rv3396c | guaA | 7 | 19 | 12 | 30 | 0.5585 | -0.0165 | 0.9211 |
| Rv3624c | hpt | 7 | 19 | 12 | 30 | 0.5585 | -0.0165 | 0.9211 |
| Rv3703c | Rv3703c | 7 | 19 | 12 | 30 | 0.5585 | -0.0165 | 0.9211 |
| Rv3792 | Rv3792 | 7 | 19 | 12 | 30 | 0.5585 | -0.0165 | 0.9211 |
| Rv3878 | Rv3878 | 7 | 19 | 12 | 30 | 0.5585 | -0.0165 | 0.9211 |
| Rv0039c | Rv0039c | 2 | 24 | 4 | 38 | 0.6021 | -0.0183 | 0.7917 |
| Rv0061 | Rv0061 | 2 | 24 | 4 | 38 | 0.6021 | -0.0183 | 0.7917 |
| Rv0078A | Rv0078A | 2 | 24 | 4 | 38 | 0.6021 | -0.0183 | 0.7917 |
| Rv0115 | hddA | 2 | 24 | 4 | 38 | 0.6021 | -0.0183 | 0.7917 |
| Rv0117 | oxyS | 2 | 24 | 4 | 38 | 0.6021 | -0.0183 | 0.7917 |
| Rv0154c | fadE2 | 2 | 24 | 4 | 38 | 0.6021 | -0.0183 | 0.7917 |
| Rv0169 | mce1A | 2 | 24 | 4 | 38 | 0.6021 | -0.0183 | 0.7917 |
| Rv0182c | sigG | 2 | 24 | 4 | 38 | 0.6021 | -0.0183 | 0.7917 |
| Rv0185 | Rv0185 | 2 | 24 | 4 | 38 | 0.6021 | -0.0183 | 0.7917 |
| Rv0198c | Rv0198c | 2 | 24 | 4 | 38 | 0.6021 | -0.0183 | 0.7917 |
| Rv0221 | Rv0221 | 2 | 24 | 4 | 38 | 0.6021 | -0.0183 | 0.7917 |
| Rv0223c | Rv0223c | 2 | 24 | 4 | 38 | 0.6021 | -0.0183 | 0.7917 |

| Rv0261c | narK3 | 2 | 24 | 4 | 38 | 0.6021 | -0.0183 | 0.7917 |
| --- | --- | --- | --- | --- | --- | --- | --- | --- |
| Rv0282 | Rv0282 | 2 | 24 | 4 | 38 | 0.6021 | -0.0183 | 0.7917 |
| Rv0302 | Rv0302 | 2 | 24 | 4 | 38 | 0.6021 | -0.0183 | 0.7917 |
| Rv0311 | Rv0311 | 2 | 24 | 4 | 38 | 0.6021 | -0.0183 | 0.7917 |
| Rv0325 | Rv0325 | 2 | 24 | 4 | 38 | 0.6021 | -0.0183 | 0.7917 |
| Rv0398c | Rv0398c | 2 | 24 | 4 | 38 | 0.6021 | -0.0183 | 0.7917 |
| Rv0408 | pta | 2 | 24 | 4 | 38 | 0.6021 | -0.0183 | 0.7917 |
| Rv0428c | Rv0428c | 2 | 24 | 4 | 38 | 0.6021 | -0.0183 | 0.7917 |
| Rv0444c | Rv0444c | 2 | 24 | 4 | 38 | 0.6021 | -0.0183 | 0.7917 |
| Rv0452 | Rv0452 | 2 | 24 | 4 | 38 | 0.6021 | -0.0183 | 0.7917 |
| Rv0492c | Rv0492c | 2 | 24 | 4 | 38 | 0.6021 | -0.0183 | 0.7917 |
| Rv0529 | ccsA | 2 | 24 | 4 | 38 | 0.6021 | -0.0183 | 0.7917 |
| Rv0530 | Rv0530 | 2 | 24 | 4 | 38 | 0.6021 | -0.0183 | 0.7917 |
| Rv0575c | Rv0575c | 2 | 24 | 4 | 38 | 0.6021 | -0.0183 | 0.7917 |
| Rv0673 | echA4 | 2 | 24 | 4 | 38 | 0.6021 | -0.0183 | 0.7917 |
| Rv0679c | Rv0679c | 2 | 24 | 4 | 38 | 0.6021 | -0.0183 | 0.7917 |
| Rv0691c | Rv0691c | 2 | 24 | 4 | 38 | 0.6021 | -0.0183 | 0.7917 |
| Rv0699 | Rv0699 | 2 | 24 | 4 | 38 | 0.6021 | -0.0183 | 0.7917 |
| Rv0728c | serA2 | 2 | 24 | 4 | 38 | 0.6021 | -0.0183 | 0.7917 |
| Rv0745 | Rv0745 | 2 | 24 | 4 | 38 | 0.6021 | -0.0183 | 0.7917 |
| Rv0768 | aldA | 2 | 24 | 4 | 38 | 0.6021 | -0.0183 | 0.7917 |
| Rv0814c | sseC2 | 2 | 24 | 4 | 38 | 0.6021 | -0.0183 | 0.7917 |
| Rv0834c | PE_PGRS14 | 2 | 24 | 4 | 38 | 0.6021 | -0.0183 | 0.7917 |
| Rv0836c | Rv0836c | 2 | 24 | 4 | 38 | 0.6021 | -0.0183 | 0.7917 |
| Rv0844c | narL | 2 | 24 | 4 | 38 | 0.6021 | -0.0183 | 0.7917 |
| Rv0862c | Rv0862c | 2 | 24 | 4 | 38 | 0.6021 | -0.0183 | 0.7917 |
| Rv0874c | Rv0874c | 2 | 24 | 4 | 38 | 0.6021 | -0.0183 | 0.7917 |
| Rv0889c | citA | 2 | 24 | 4 | 38 | 0.6021 | -0.0183 | 0.7917 |
| Rv0907 | Rv0907 | 2 | 24 | 4 | 38 | 0.6021 | -0.0183 | 0.7917 |
| Rv0959 | Rv0959 | 2 | 24 | 4 | 38 | 0.6021 | -0.0183 | 0.7917 |
| Rv0988 | Rv0988 | 2 | 24 | 4 | 38 | 0.6021 | -0.0183 | 0.7917 |
| Rv1014c | pth | 2 | 24 | 4 | 38 | 0.6021 | -0.0183 | 0.7917 |
| Rv1042c | Rv1041c | 2 | 24 | 4 | 38 | 0.6021 | -0.0183 | 0.7917 |
| Rv1054 | Rv1054 | 2 | 24 | 4 | 38 | 0.6021 | -0.0183 | 0.7917 |

| Rv1076 | lipU | 2 | 24 | 4 | 38 | 0.6021 | -0.0183 | 0.7917 |
| --- | --- | --- | --- | --- | --- | --- | --- | --- |
| Rv1086 | Rv1086 | 2 | 24 | 4 | 38 | 0.6021 | -0.0183 | 0.7917 |
| Rv1090 | celA2b | 2 | 24 | 4 | 38 | 0.6021 | -0.0183 | 0.7917 |
| Rv1102c | Rv1102c | 2 | 24 | 4 | 38 | 0.6021 | -0.0183 | 0.7917 |
| Rv1125 | Rv1125 | 2 | 24 | 4 | 38 | 0.6021 | -0.0183 | 0.7917 |
| Rv1149 | Rv1149 | 2 | 24 | 4 | 38 | 0.6021 | -0.0183 | 0.7917 |
| Rv1160 | mutT2 | 2 | 24 | 4 | 38 | 0.6021 | -0.0183 | 0.7917 |
| Rv1168c | PPE17 | 2 | 24 | 4 | 38 | 0.6021 | -0.0183 | 0.7917 |
| Rv1212c | Rv1212c | 2 | 24 | 4 | 38 | 0.6021 | -0.0183 | 0.7917 |
| Rv1217c | Rv1217c | 2 | 24 | 4 | 38 | 0.6021 | -0.0183 | 0.7917 |
| Rv1224 | tatB | 2 | 24 | 4 | 38 | 0.6021 | -0.0183 | 0.7917 |
| Rv1248c | kgd | 2 | 24 | 4 | 38 | 0.6021 | -0.0183 | 0.7917 |
| Rv1367c | Rv1367c | 2 | 24 | 4 | 38 | 0.6021 | -0.0183 | 0.7917 |
| Rv1383 | carA | 2 | 24 | 4 | 38 | 0.6021 | -0.0183 | 0.7917 |
| Rv1400c | lipI | 2 | 24 | 4 | 38 | 0.6021 | -0.0183 | 0.7917 |
| Rv1420 | uvrC | 2 | 24 | 4 | 38 | 0.6021 | -0.0183 | 0.7917 |
| Rv1439c | Rv1439c | 2 | 24 | 4 | 38 | 0.6021 | -0.0183 | 0.7917 |
| Rv1442 | bisC | 2 | 24 | 4 | 38 | 0.6021 | -0.0183 | 0.7917 |
| Rv1458c | Rv1458c | 2 | 24 | 4 | 38 | 0.6021 | -0.0183 | 0.7917 |
| Rv1489 | Rv1489 | 2 | 24 | 4 | 38 | 0.6021 | -0.0183 | 0.7917 |
| Rv1515c | Rv1515c | 2 | 24 | 4 | 38 | 0.6021 | -0.0183 | 0.7917 |
| Rv1520 | Rv1520 | 2 | 24 | 4 | 38 | 0.6021 | -0.0183 | 0.7917 |
| Rv1532c | Rv1532c | 2 | 24 | 4 | 38 | 0.6021 | -0.0183 | 0.7917 |
| Rv1563c | treY | 2 | 24 | 4 | 38 | 0.6021 | -0.0183 | 0.7917 |
| Rv1634 | Rv1634 | 2 | 24 | 4 | 38 | 0.6021 | -0.0183 | 0.7917 |
| Rv1640c | lysS | 2 | 24 | 4 | 38 | 0.6021 | -0.0183 | 0.7917 |
| Rv1649 | pheS | 2 | 24 | 4 | 38 | 0.6021 | -0.0183 | 0.7917 |
| Rv1681 | moeX | 2 | 24 | 4 | 38 | 0.6021 | -0.0183 | 0.7917 |
| Rv1765c | Rv1765c | 2 | 24 | 4 | 38 | 0.6021 | -0.0183 | 0.7917 |
| Rv1802 | PPE30 | 2 | 24 | 4 | 38 | 0.6021 | -0.0183 | 0.7917 |
| Rv1808 | PPE32 | 2 | 24 | 4 | 38 | 0.6021 | -0.0183 | 0.7917 |
| Rv1823 | Rv1823 | 2 | 24 | 4 | 38 | 0.6021 | -0.0183 | 0.7917 |
| Rv1826 | gcvH | 2 | 24 | 4 | 38 | 0.6021 | -0.0183 | 0.7917 |
| Rv1837c | glcB | 2 | 24 | 4 | 38 | 0.6021 | -0.0183 | 0.7917 |

| Rv1847 | Rv1847 | 2 | 24 | 4 | 38 | 0.6021 | -0.0183 | 0.7917 |
| --- | --- | --- | --- | --- | --- | --- | --- | --- |
| Rv1904 | Rv1904 | 2 | 24 | 4 | 38 | 0.6021 | -0.0183 | 0.7917 |
| Rv1912c | fadB5 | 2 | 24 | 4 | 38 | 0.6021 | -0.0183 | 0.7917 |
| Rv1927 | Rv1927 | 2 | 24 | 4 | 38 | 0.6021 | -0.0183 | 0.7917 |
| Rv1934c | fadE17 | 2 | 24 | 4 | 38 | 0.6021 | -0.0183 | 0.7917 |
| Rv1936 | Rv1936 | 2 | 24 | 4 | 38 | 0.6021 | -0.0183 | 0.7917 |
| Rv1938 | ephB | 2 | 24 | 4 | 38 | 0.6021 | -0.0183 | 0.7917 |
| Rv1948c | Rv1948c | 2 | 24 | 4 | 38 | 0.6021 | -0.0183 | 0.7917 |
| Rv1972 | Rv1972 | 2 | 24 | 4 | 38 | 0.6021 | -0.0183 | 0.7917 |
| Rv1986 | Rv1986 | 2 | 24 | 4 | 38 | 0.6021 | -0.0183 | 0.7917 |
| Rv2057c | rpmG | 2 | 24 | 4 | 38 | 0.6021 | -0.0183 | 0.7917 |
| Rv2071c | cobM | 2 | 24 | 4 | 38 | 0.6021 | -0.0183 | 0.7917 |
| Rv2075c | Rv2075c | 2 | 24 | 4 | 38 | 0.6021 | -0.0183 | 0.7917 |
| Rv2088 | pknJ | 2 | 24 | 4 | 38 | 0.6021 | -0.0183 | 0.7917 |
| Rv2089c | pepE | 2 | 24 | 4 | 38 | 0.6021 | -0.0183 | 0.7917 |
| Rv2096c | Rv2096c | 2 | 24 | 4 | 38 | 0.6021 | -0.0183 | 0.7917 |
| Rv2139 | pyrD | 2 | 24 | 4 | 38 | 0.6021 | -0.0183 | 0.7917 |
| Rv2153c | murG | 2 | 24 | 4 | 38 | 0.6021 | -0.0183 | 0.7917 |
| Rv2160c | Rv2160A | 2 | 24 | 4 | 38 | 0.6021 | -0.0183 | 0.7917 |
| Rv2187 | fadD15 | 2 | 24 | 4 | 38 | 0.6021 | -0.0183 | 0.7917 |
| Rv2274c | Rv2274c | 2 | 24 | 4 | 38 | 0.6021 | -0.0183 | 0.7917 |
| Rv2366c | Rv2366c | 2 | 24 | 4 | 38 | 0.6021 | -0.0183 | 0.7917 |
| Rv2400c | subI | 2 | 24 | 4 | 38 | 0.6021 | -0.0183 | 0.7917 |
| Rv2433c | Rv2433c | 2 | 24 | 4 | 38 | 0.6021 | -0.0183 | 0.7917 |
| Rv2440c | obgE | 2 | 24 | 4 | 38 | 0.6021 | -0.0183 | 0.7917 |
| Rv2477c | Rv2477c | 2 | 24 | 4 | 38 | 0.6021 | -0.0183 | 0.7917 |
| Rv2485c | lipQ | 2 | 24 | 4 | 38 | 0.6021 | -0.0183 | 0.7917 |
| Rv2502c | accD1 | 2 | 24 | 4 | 38 | 0.6021 | -0.0183 | 0.7917 |
| Rv2516c | Rv2516c | 2 | 24 | 4 | 38 | 0.6021 | -0.0183 | 0.7917 |
| Rv2562 | Rv2562 | 2 | 24 | 4 | 38 | 0.6021 | -0.0183 | 0.7917 |
| Rv2564 | glnQ | 2 | 24 | 4 | 38 | 0.6021 | -0.0183 | 0.7917 |
| Rv2596 | Rv2596 | 2 | 24 | 4 | 38 | 0.6021 | -0.0183 | 0.7917 |
| Rv2629 | Rv2629 | 2 | 24 | 4 | 38 | 0.6021 | -0.0183 | 0.7917 |
| Rv2672 | Rv2672 | 2 | 24 | 4 | 38 | 0.6021 | -0.0183 | 0.7917 |

| Rv2719c | Rv2719c | 2 | 24 | 4 | 38 | 0.6021 | -0.0183 | 0.7917 |
| --- | --- | --- | --- | --- | --- | --- | --- | --- |
| Rv2736c | recX | 2 | 24 | 4 | 38 | 0.6021 | -0.0183 | 0.7917 |
| Rv2770c | PPE44 | 2 | 24 | 4 | 38 | 0.6021 | -0.0183 | 0.7917 |
| Rv2786c | ribF | 2 | 24 | 4 | 38 | 0.6021 | -0.0183 | 0.7917 |
| Rv2805 | Rv2804c | 2 | 24 | 4 | 38 | 0.6021 | -0.0183 | 0.7917 |
| Rv2831 | echA16 | 2 | 24 | 4 | 38 | 0.6021 | -0.0183 | 0.7917 |
| Rv2838c | Rv2837c | 2 | 24 | 4 | 38 | 0.6021 | -0.0183 | 0.7917 |
| Rv2862c | Rv2862c | 2 | 24 | 4 | 38 | 0.6021 | -0.0183 | 0.7917 |
| Rv2869c | Rv2869c | 2 | 24 | 4 | 38 | 0.6021 | -0.0183 | 0.7917 |
| Rv2891 | Rv2891 | 2 | 24 | 4 | 38 | 0.6021 | -0.0183 | 0.7917 |
| Rv2899c | fdhD | 2 | 24 | 4 | 38 | 0.6021 | -0.0183 | 0.7917 |
| Rv2952 | Rv2952 | 2 | 24 | 4 | 38 | 0.6021 | -0.0183 | 0.7917 |
| Rv2964 | purU | 2 | 24 | 4 | 38 | 0.6021 | -0.0183 | 0.7917 |
| Rv2971 | Rv2971 | 2 | 24 | 4 | 38 | 0.6021 | -0.0183 | 0.7917 |
| Rv3034c | Rv3034c | 2 | 24 | 4 | 38 | 0.6021 | -0.0183 | 0.7917 |
| Rv3047c | Rv3047c | 2 | 24 | 4 | 38 | 0.6021 | -0.0183 | 0.7917 |
| Rv3114 | Rv3114 | 2 | 24 | 4 | 38 | 0.6021 | -0.0183 | 0.7917 |
| Rv3161c | Rv3161c | 2 | 24 | 4 | 38 | 0.6021 | -0.0183 | 0.7917 |
| Rv3169 | Rv3169 | 2 | 24 | 4 | 38 | 0.6021 | -0.0183 | 0.7917 |
| Rv3202c | Rv3202c | 2 | 24 | 4 | 38 | 0.6021 | -0.0183 | 0.7917 |
| Rv3257c | manB | 2 | 24 | 4 | 38 | 0.6021 | -0.0183 | 0.7917 |
| Rv3272 | Rv3272 | 2 | 24 | 4 | 38 | 0.6021 | -0.0183 | 0.7917 |
| Rv3283 | sseA | 2 | 24 | 4 | 38 | 0.6021 | -0.0183 | 0.7917 |
| Rv3293 | pcd | 2 | 24 | 4 | 38 | 0.6021 | -0.0183 | 0.7917 |
| Rv3345c | PE_PGRS49 | 2 | 24 | 4 | 38 | 0.6021 | -0.0183 | 0.7917 |
| Rv3389c | Rv3389c | 2 | 24 | 4 | 38 | 0.6021 | -0.0183 | 0.7917 |
| Rv3391 | acrA1 | 2 | 24 | 4 | 38 | 0.6021 | -0.0183 | 0.7917 |
| Rv3402c | Rv3402c | 2 | 24 | 4 | 38 | 0.6021 | -0.0183 | 0.7917 |
| Rv3407 | Rv3407 | 2 | 24 | 4 | 38 | 0.6021 | -0.0183 | 0.7917 |
| Rv3433c | Rv3433c | 2 | 24 | 4 | 38 | 0.6021 | -0.0183 | 0.7917 |
| Rv3487c | lipF | 2 | 24 | 4 | 38 | 0.6021 | -0.0183 | 0.7917 |
| Rv3490 | otsA | 2 | 24 | 4 | 38 | 0.6021 | -0.0183 | 0.7917 |
| Rv3567c | Rv3567c | 2 | 24 | 4 | 38 | 0.6021 | -0.0183 | 0.7917 |
| Rv3671c | Rv3671c | 2 | 24 | 4 | 38 | 0.6021 | -0.0183 | 0.7917 |

| Rv3674c | nth | 2 | 24 | 4 | 38 | 0.6021 | -0.0183 | 0.7917 |
| --- | --- | --- | --- | --- | --- | --- | --- | --- |
| Rv3676 | Rv3676 | 2 | 24 | 4 | 38 | 0.6021 | -0.0183 | 0.7917 |
| Rv3682 | ponA2 | 2 | 24 | 4 | 38 | 0.6021 | -0.0183 | 0.7917 |
| Rv3715c | recR | 2 | 24 | 4 | 38 | 0.6021 | -0.0183 | 0.7917 |
| Rv3722c | Rv3722c | 2 | 24 | 4 | 38 | 0.6021 | -0.0183 | 0.7917 |
| Rv3724B | cut5b | 2 | 24 | 4 | 38 | 0.6021 | -0.0183 | 0.7917 |
| Rv3725 | Rv3725 | 2 | 24 | 4 | 38 | 0.6021 | -0.0183 | 0.7917 |
| Rv3758c | proV | 2 | 24 | 4 | 38 | 0.6021 | -0.0183 | 0.7917 |
| Rv3770c | Rv3770c | 2 | 24 | 4 | 38 | 0.6021 | -0.0183 | 0.7917 |
| Rv3782 | Rv3782 | 2 | 24 | 4 | 38 | 0.6021 | -0.0183 | 0.7917 |
| Rv3788 | Rv3788 | 2 | 24 | 4 | 38 | 0.6021 | -0.0183 | 0.7917 |
| Rv3835 | Rv3835 | 2 | 24 | 4 | 38 | 0.6021 | -0.0183 | 0.7917 |
| Rv3837c | Rv3837c | 2 | 24 | 4 | 38 | 0.6021 | -0.0183 | 0.7917 |
| Rv3913 | trxB2 | 2 | 24 | 4 | 38 | 0.6021 | -0.0183 | 0.7917 |
| Rv3916c | Rv3916c | 2 | 24 | 4 | 38 | 0.6021 | -0.0183 | 0.7917 |
| Rv2048c | pks12 | 15 | 11 | 25 | 17 | 0.5593 | -0.0183 | 0.9273 |
| Rv3365c | Rv3365c | 15 | 11 | 25 | 17 | 0.5593 | -0.0183 | 0.9273 |
| Rv0825c | Rv0825c | 10 | 16 | 17 | 25 | 0.5655 | -0.0201 | 0.9191 |
| Rv1183 | mmpL10 | 5 | 21 | 9 | 33 | 0.5862 | -0.0220 | 0.8730 |
| Rv2300c | Rv2300c | 5 | 21 | 9 | 33 | 0.5862 | -0.0220 | 0.8730 |
| Rv2731 | Rv2731 | 5 | 21 | 9 | 33 | 0.5862 | -0.0220 | 0.8730 |
| Rv0011c | Rv0011c | 0 | 26 | 1 | 41 | 0.7860 | -0.0238 | 0.0000 |
| Rv0023 | Rv0023 | 0 | 26 | 1 | 41 | 0.7860 | -0.0238 | 0.0000 |
| Rv0027 | Rv0027 | 0 | 26 | 1 | 41 | 0.7860 | -0.0238 | 0.0000 |
| Rv0043c | Rv0043c | 0 | 26 | 1 | 41 | 0.7860 | -0.0238 | 0.0000 |
| Rv0044c | Rv0044c | 0 | 26 | 1 | 41 | 0.7860 | -0.0238 | 0.0000 |
| Rv0049 | Rv0049 | 0 | 26 | 1 | 41 | 0.7860 | -0.0238 | 0.0000 |
| Rv0052 | Rv0052 | 0 | 26 | 1 | 41 | 0.7860 | -0.0238 | 0.0000 |
| Rv0056 | rplI | 0 | 26 | 1 | 41 | 0.7860 | -0.0238 | 0.0000 |
| Rv0078 | Rv0078 | 0 | 26 | 1 | 41 | 0.7860 | -0.0238 | 0.0000 |
| Rv0079 | Rv0079 | 0 | 26 | 1 | 41 | 0.7860 | -0.0238 | 0.0000 |
| Rv0085 | hycP | 0 | 26 | 1 | 41 | 0.7860 | -0.0238 | 0.0000 |
| Rv0091 | mtn | 0 | 26 | 1 | 41 | 0.7860 | -0.0238 | 0.0000 |
| Rv0106 | Rv0106 | 0 | 26 | 1 | 41 | 0.7860 | -0.0238 | 0.0000 |

| Rv0112 | gca | 0 | 26 | 1 | 41 | 0.7860 | -0.0238 | 0.0000 |
| --- | --- | --- | --- | --- | --- | --- | --- | --- |
| Rv0126 | treS | 0 | 26 | 1 | 41 | 0.7860 | -0.0238 | 0.0000 |
| Rv0155 | pntAa | 0 | 26 | 1 | 41 | 0.7860 | -0.0238 | 0.0000 |
| Rv0183 | Rv0183 | 0 | 26 | 1 | 41 | 0.7860 | -0.0238 | 0.0000 |
| Rv0189c | ilvD | 0 | 26 | 1 | 41 | 0.7860 | -0.0238 | 0.0000 |
| Rv0208c | trmB | 0 | 26 | 1 | 41 | 0.7860 | -0.0238 | 0.0000 |
| Rv0217c | lipW | 0 | 26 | 1 | 41 | 0.7860 | -0.0238 | 0.0000 |
| Rv0234c | gabD1 | 0 | 26 | 1 | 41 | 0.7860 | -0.0238 | 0.0000 |
| Rv0239 | Rv0239 | 0 | 26 | 1 | 41 | 0.7860 | -0.0238 | 0.0000 |
| Rv0241c | Rv0241c | 0 | 26 | 1 | 41 | 0.7860 | -0.0238 | 0.0000 |
| Rv0260c | Rv0260c | 0 | 26 | 1 | 41 | 0.7860 | -0.0238 | 0.0000 |
| Rv0265c | Rv0265c | 0 | 26 | 1 | 41 | 0.7860 | -0.0238 | 0.0000 |
| Rv0267 | narU | 0 | 26 | 1 | 41 | 0.7860 | -0.0238 | 0.0000 |
| Rv0269c | Rv0269c | 0 | 26 | 1 | 41 | 0.7860 | -0.0238 | 0.0000 |
| Rv0274 | Rv0274 | 0 | 26 | 1 | 41 | 0.7860 | -0.0238 | 0.0000 |
| Rv0283 | Rv0283 | 0 | 26 | 1 | 41 | 0.7860 | -0.0238 | 0.0000 |
| Rv0296c | Rv0296c | 0 | 26 | 1 | 41 | 0.7860 | -0.0238 | 0.0000 |
| Rv0306 | Rv0306 | 0 | 26 | 1 | 41 | 0.7860 | -0.0238 | 0.0000 |
| Rv0312 | Rv0312 | 0 | 26 | 1 | 41 | 0.7860 | -0.0238 | 0.0000 |
| Rv0320 | Rv0320 | 0 | 26 | 1 | 41 | 0.7860 | -0.0238 | 0.0000 |
| Rv0334 | rmlA | 0 | 26 | 1 | 41 | 0.7860 | -0.0238 | 0.0000 |
| Rv0372c | Rv0372c | 0 | 26 | 1 | 41 | 0.7860 | -0.0238 | 0.0000 |
| Rv0393 | Rv0393 | 0 | 26 | 1 | 41 | 0.7860 | -0.0238 | 0.0000 |
| Rv0410c | pknG | 0 | 26 | 1 | 41 | 0.7860 | -0.0238 | 0.0000 |
| Rv0447c | ufaA1 | 0 | 26 | 1 | 41 | 0.7860 | -0.0238 | 0.0000 |
| Rv0450c | mmpL4 | 0 | 26 | 1 | 41 | 0.7860 | -0.0238 | 0.0000 |
| Rv0480c | Rv0480c | 0 | 26 | 1 | 41 | 0.7860 | -0.0238 | 0.0000 |
| Rv0482 | murB | 0 | 26 | 1 | 41 | 0.7860 | -0.0238 | 0.0000 |
| Rv0490 | senX3 | 0 | 26 | 1 | 41 | 0.7860 | -0.0238 | 0.0000 |
| Rv0492A | Rv0492A | 0 | 26 | 1 | 41 | 0.7860 | -0.0238 | 0.0000 |
| Rv0497 | Rv0497 | 0 | 26 | 1 | 41 | 0.7860 | -0.0238 | 0.0000 |
| Rv0500 | proC | 0 | 26 | 1 | 41 | 0.7860 | -0.0238 | 0.0000 |
| Rv0511 | hemD | 0 | 26 | 1 | 41 | 0.7860 | -0.0238 | 0.0000 |
| Rv0519c | Rv0519c | 0 | 26 | 1 | 41 | 0.7860 | -0.0238 | 0.0000 |

| Rv0563 | htpX | 0 | 26 | 1 | 41 | 0.7860 | -0.0238 | 0.0000 |
| --- | --- | --- | --- | --- | --- | --- | --- | --- |
| Rv0577 | TB27.3 | 0 | 26 | 1 | 41 | 0.7860 | -0.0238 | 0.0000 |
| Rv0582 | Rv0582 | 0 | 26 | 1 | 41 | 0.7860 | -0.0238 | 0.0000 |
| Rv0585c | Rv0585c | 0 | 26 | 1 | 41 | 0.7860 | -0.0238 | 0.0000 |
| Rv0586 | Rv0586 | 0 | 26 | 1 | 41 | 0.7860 | -0.0238 | 0.0000 |
| Rv0587 | yrbE2A | 0 | 26 | 1 | 41 | 0.7860 | -0.0238 | 0.0000 |
| Rv0600c | Rv0600c | 0 | 26 | 1 | 41 | 0.7860 | -0.0238 | 0.0000 |
| Rv0604 | lpqO | 0 | 26 | 1 | 41 | 0.7860 | -0.0238 | 0.0000 |
| Rv0610c | Rv0610c | 0 | 26 | 1 | 41 | 0.7860 | -0.0238 | 0.0000 |
| Rv0632c | echA3 | 0 | 26 | 1 | 41 | 0.7860 | -0.0238 | 0.0000 |
| Rv0645c | mmaA1 | 0 | 26 | 1 | 41 | 0.7860 | -0.0238 | 0.0000 |
| Rv0654 | Rv0654 | 0 | 26 | 1 | 41 | 0.7860 | -0.0238 | 0.0000 |
| Rv0659c | Rv0659c | 0 | 26 | 1 | 41 | 0.7860 | -0.0238 | 0.0000 |
| Rv0662c | Rv0662c | 0 | 26 | 1 | 41 | 0.7860 | -0.0238 | 0.0000 |
| Rv0670 | end | 0 | 26 | 1 | 41 | 0.7860 | -0.0238 | 0.0000 |
| Rv0672 | fadE8 | 0 | 26 | 1 | 41 | 0.7860 | -0.0238 | 0.0000 |
| Rv0690c | Rv0690c | 0 | 26 | 1 | 41 | 0.7860 | -0.0238 | 0.0000 |
| Rv0701 | rplC | 0 | 26 | 1 | 41 | 0.7860 | -0.0238 | 0.0000 |
| Rv0711 | atsA | 0 | 26 | 1 | 41 | 0.7860 | -0.0238 | 0.0000 |
| Rv0733 | adk | 0 | 26 | 1 | 41 | 0.7860 | -0.0238 | 0.0000 |
| Rv0742 | PE_PGRS8 | 0 | 26 | 1 | 41 | 0.7860 | -0.0238 | 0.0000 |
| Rv0767c | Rv0767c | 0 | 26 | 1 | 41 | 0.7860 | -0.0238 | 0.0000 |
| Rv0778 | cyp126 | 0 | 26 | 1 | 41 | 0.7860 | -0.0238 | 0.0000 |
| Rv0786c | Rv0786c | 0 | 26 | 1 | 41 | 0.7860 | -0.0238 | 0.0000 |
| Rv0804 | Rv0804 | 0 | 26 | 1 | 41 | 0.7860 | -0.0238 | 0.0000 |
| Rv0807 | Rv0807 | 0 | 26 | 1 | 41 | 0.7860 | -0.0238 | 0.0000 |
| Rv0811c | Rv0811c | 0 | 26 | 1 | 41 | 0.7860 | -0.0238 | 0.0000 |
| Rv0827c | Rv0827c | 0 | 26 | 1 | 41 | 0.7860 | -0.0238 | 0.0000 |
| Rv0832 | PE_PGRS12 | 0 | 26 | 1 | 41 | 0.7860 | -0.0238 | 0.0000 |
| Rv0837c | Rv0837c | 0 | 26 | 1 | 41 | 0.7860 | -0.0238 | 0.0000 |
| Rv0838 | lpqR | 0 | 26 | 1 | 41 | 0.7860 | -0.0238 | 0.0000 |
| Rv0849 | Rv0849 | 0 | 26 | 1 | 41 | 0.7860 | -0.0238 | 0.0000 |
| Rv0861c | ercc3 | 0 | 26 | 1 | 41 | 0.7860 | -0.0238 | 0.0000 |
| Rv0864 | moaC | 0 | 26 | 1 | 41 | 0.7860 | -0.0238 | 0.0000 |

| Rv0866 | moaE2 | 0 | 26 | 1 | 41 | 0.7860 | -0.0238 | 0.0000 |
| --- | --- | --- | --- | --- | --- | --- | --- | --- |
| Rv0875c | Rv0875c | 0 | 26 | 1 | 41 | 0.7860 | -0.0238 | 0.0000 |
| Rv0883c | Rv0883c | 0 | 26 | 1 | 41 | 0.7860 | -0.0238 | 0.0000 |
| Rv0895 | Rv0895 | 0 | 26 | 1 | 41 | 0.7860 | -0.0238 | 0.0000 |
| Rv0905 | echA6 | 0 | 26 | 1 | 41 | 0.7860 | -0.0238 | 0.0000 |
| Rv0913c | Rv0913c | 0 | 26 | 1 | 41 | 0.7860 | -0.0238 | 0.0000 |
| Rv0944 | Rv0944 | 0 | 26 | 1 | 41 | 0.7860 | -0.0238 | 0.0000 |
| Rv0950c | Rv0950c | 0 | 26 | 1 | 41 | 0.7860 | -0.0238 | 0.0000 |
| Rv0953c | Rv0953c | 0 | 26 | 1 | 41 | 0.7860 | -0.0238 | 0.0000 |
| Rv0973c | accA2 | 0 | 26 | 1 | 41 | 0.7860 | -0.0238 | 0.0000 |
| Rv0978c | PE_PGRS17 | 0 | 26 | 1 | 41 | 0.7860 | -0.0238 | 0.0000 |
| Rv1001 | arcA | 0 | 26 | 1 | 41 | 0.7860 | -0.0238 | 0.0000 |
| Rv1003 | Rv1003 | 0 | 26 | 1 | 41 | 0.7860 | -0.0238 | 0.0000 |
| Rv1009 | rpfB | 0 | 26 | 1 | 41 | 0.7860 | -0.0238 | 0.0000 |
| Rv1011 | ispE | 0 | 26 | 1 | 41 | 0.7860 | -0.0238 | 0.0000 |
| Rv1013 | pks16 | 0 | 26 | 1 | 41 | 0.7860 | -0.0238 | 0.0000 |
| Rv1018c | glmU | 0 | 26 | 1 | 41 | 0.7860 | -0.0238 | 0.0000 |
| Rv1025 | Rv1025 | 0 | 26 | 1 | 41 | 0.7860 | -0.0238 | 0.0000 |
| Rv1035c | Rv1035c | 0 | 26 | 1 | 41 | 0.7860 | -0.0238 | 0.0000 |
| Rv1063c | Rv1063c | 0 | 26 | 1 | 41 | 0.7860 | -0.0238 | 0.0000 |
| Rv1065 | Rv1065 | 0 | 26 | 1 | 41 | 0.7860 | -0.0238 | 0.0000 |
| Rv1078 | pra | 0 | 26 | 1 | 41 | 0.7860 | -0.0238 | 0.0000 |
| Rv1084 | Rv1084 | 0 | 26 | 1 | 41 | 0.7860 | -0.0238 | 0.0000 |
| Rv1088 | PE9 | 0 | 26 | 1 | 41 | 0.7860 | -0.0238 | 0.0000 |
| Rv1094 | desA2 | 0 | 26 | 1 | 41 | 0.7860 | -0.0238 | 0.0000 |
| Rv1096 | Rv1096 | 0 | 26 | 1 | 41 | 0.7860 | -0.0238 | 0.0000 |
| Rv1131 | gltA1 | 0 | 26 | 1 | 41 | 0.7860 | -0.0238 | 0.0000 |
| Rv1132 | Rv1132 | 0 | 26 | 1 | 41 | 0.7860 | -0.0238 | 0.0000 |
| Rv1135c | PPE16 | 0 | 26 | 1 | 41 | 0.7860 | -0.0238 | 0.0000 |
| Rv1137c | Rv1137c | 0 | 26 | 1 | 41 | 0.7860 | -0.0238 | 0.0000 |
| Rv1165 | typA | 0 | 26 | 1 | 41 | 0.7860 | -0.0238 | 0.0000 |
| Rv1173 | fbiC | 0 | 26 | 1 | 41 | 0.7860 | -0.0238 | 0.0000 |
| Rv1176c | Rv1176c | 0 | 26 | 1 | 41 | 0.7860 | -0.0238 | 0.0000 |
| Rv1182 | papA3 | 0 | 26 | 1 | 41 | 0.7860 | -0.0238 | 0.0000 |

| Rv1185c | fadD21 | 0 | 26 | 1 | 41 | 0.7860 | -0.0238 | 0.0000 |
| --- | --- | --- | --- | --- | --- | --- | --- | --- |
| Rv1213 | glgC | 0 | 26 | 1 | 41 | 0.7860 | -0.0238 | 0.0000 |
| Rv1219c | Rv1219c | 0 | 26 | 1 | 41 | 0.7860 | -0.0238 | 0.0000 |
| Rv1235 | lpqY | 0 | 26 | 1 | 41 | 0.7860 | -0.0238 | 0.0000 |
| Rv1240 | mdh | 0 | 26 | 1 | 41 | 0.7860 | -0.0238 | 0.0000 |
| Rv1246c | Rv1246c | 0 | 26 | 1 | 41 | 0.7860 | -0.0238 | 0.0000 |
| Rv1267c | embR | 0 | 26 | 1 | 41 | 0.7860 | -0.0238 | 0.0000 |
| Rv1271c | Rv1271c | 0 | 26 | 1 | 41 | 0.7860 | -0.0238 | 0.0000 |
| Rv1272c | Rv1272c | 0 | 26 | 1 | 41 | 0.7860 | -0.0238 | 0.0000 |
| Rv1283c | oppB | 0 | 26 | 1 | 41 | 0.7860 | -0.0238 | 0.0000 |
| Rv1296 | thrB | 0 | 26 | 1 | 41 | 0.7860 | -0.0238 | 0.0000 |
| Rv1303 | Rv1303 | 0 | 26 | 1 | 41 | 0.7860 | -0.0238 | 0.0000 |
| Rv1304 | atpB | 0 | 26 | 1 | 41 | 0.7860 | -0.0238 | 0.0000 |
| Rv1315 | murA | 0 | 26 | 1 | 41 | 0.7860 | -0.0238 | 0.0000 |
| Rv1357c | Rv1357c | 0 | 26 | 1 | 41 | 0.7860 | -0.0238 | 0.0000 |
| Rv1366 | Rv1366 | 0 | 26 | 1 | 41 | 0.7860 | -0.0238 | 0.0000 |
| Rv1371 | Rv1371 | 0 | 26 | 1 | 41 | 0.7860 | -0.0238 | 0.0000 |
| Rv1381 | pyrC | 0 | 26 | 1 | 41 | 0.7860 | -0.0238 | 0.0000 |
| Rv1382 | Rv1382 | 0 | 26 | 1 | 41 | 0.7860 | -0.0238 | 0.0000 |
| Rv1390 | rpoZ | 0 | 26 | 1 | 41 | 0.7860 | -0.0238 | 0.0000 |
| Rv1399c | lipH | 0 | 26 | 1 | 41 | 0.7860 | -0.0238 | 0.0000 |
| Rv1403c | Rv1403c | 0 | 26 | 1 | 41 | 0.7860 | -0.0238 | 0.0000 |
| Rv1407 | fmu | 0 | 26 | 1 | 41 | 0.7860 | -0.0238 | 0.0000 |
| Rv1410c | Rv1410c | 0 | 26 | 1 | 41 | 0.7860 | -0.0238 | 0.0000 |
| Rv1419 | Rv1419 | 0 | 26 | 1 | 41 | 0.7860 | -0.0238 | 0.0000 |
| Rv1430 | PE16 | 0 | 26 | 1 | 41 | 0.7860 | -0.0238 | 0.0000 |
| Rv1433 | Rv1433 | 0 | 26 | 1 | 41 | 0.7860 | -0.0238 | 0.0000 |
| Rv1469 | ctpD | 0 | 26 | 1 | 41 | 0.7860 | -0.0238 | 0.0000 |
| Rv1473 | Rv1473 | 0 | 26 | 1 | 41 | 0.7860 | -0.0238 | 0.0000 |
| Rv1478 | Rv1478 | 0 | 26 | 1 | 41 | 0.7860 | -0.0238 | 0.0000 |
| Rv1481 | Rv1481 | 0 | 26 | 1 | 41 | 0.7860 | -0.0238 | 0.0000 |
| Rv1487 | Rv1487 | 0 | 26 | 1 | 41 | 0.7860 | -0.0238 | 0.0000 |
| Rv1492 | mutA | 0 | 26 | 1 | 41 | 0.7860 | -0.0238 | 0.0000 |
| Rv1498A | Rv1498A | 0 | 26 | 1 | 41 | 0.7860 | -0.0238 | 0.0000 |

| Rv1501 | Rv1501 | 0 | 26 | 1 | 41 | 0.7860 | -0.0238 | 0.0000 |
| --- | --- | --- | --- | --- | --- | --- | --- | --- |
| Rv1503c | Rv1503c | 0 | 26 | 1 | 41 | 0.7860 | -0.0238 | 0.0000 |
| Rv1513 | Rv1513 | 0 | 26 | 1 | 41 | 0.7860 | -0.0238 | 0.0000 |
| Rv1534 | Rv1534 | 0 | 26 | 1 | 41 | 0.7860 | -0.0238 | 0.0000 |
| Rv1536 | ileS | 0 | 26 | 1 | 41 | 0.7860 | -0.0238 | 0.0000 |
| Rv1545 | Rv1545 | 0 | 26 | 1 | 41 | 0.7860 | -0.0238 | 0.0000 |
| Rv1558 | Rv1558 | 0 | 26 | 1 | 41 | 0.7860 | -0.0238 | 0.0000 |
| Rv1560 | Rv1560 | 0 | 26 | 1 | 41 | 0.7860 | -0.0238 | 0.0000 |
| Rv1562c | treZ | 0 | 26 | 1 | 41 | 0.7860 | -0.0238 | 0.0000 |
| Rv1566c | Rv1566c | 0 | 26 | 1 | 41 | 0.7860 | -0.0238 | 0.0000 |
| Rv1568 | bioA | 0 | 26 | 1 | 41 | 0.7860 | -0.0238 | 0.0000 |
| Rv1598c | Rv1598c | 0 | 26 | 1 | 41 | 0.7860 | -0.0238 | 0.0000 |
| Rv1603 | hisA | 0 | 26 | 1 | 41 | 0.7860 | -0.0238 | 0.0000 |
| Rv1617 | pykA | 0 | 26 | 1 | 41 | 0.7860 | -0.0238 | 0.0000 |
| Rv1626 | Rv1626 | 0 | 26 | 1 | 41 | 0.7860 | -0.0238 | 0.0000 |
| Rv1627c | Rv1627c | 0 | 26 | 1 | 41 | 0.7860 | -0.0238 | 0.0000 |
| Rv1635c | Rv1635c | 0 | 26 | 1 | 41 | 0.7860 | -0.0238 | 0.0000 |
| Rv1637c | Rv1637c | 0 | 26 | 1 | 41 | 0.7860 | -0.0238 | 0.0000 |
| Rv1639c | Rv1639c | 0 | 26 | 1 | 41 | 0.7860 | -0.0238 | 0.0000 |
| Rv1648 | Rv1648 | 0 | 26 | 1 | 41 | 0.7860 | -0.0238 | 0.0000 |
| Rv1667c | Rv1667c | 0 | 26 | 1 | 41 | 0.7860 | -0.0238 | 0.0000 |
| Rv1669 | Rv1669 | 0 | 26 | 1 | 41 | 0.7860 | -0.0238 | 0.0000 |
| Rv1671 | Rv1671 | 0 | 26 | 1 | 41 | 0.7860 | -0.0238 | 0.0000 |
| Rv1677 | dsbF | 0 | 26 | 1 | 41 | 0.7860 | -0.0238 | 0.0000 |
| Rv1679 | fadE16 | 0 | 26 | 1 | 41 | 0.7860 | -0.0238 | 0.0000 |
| Rv1692 | Rv1692 | 0 | 26 | 1 | 41 | 0.7860 | -0.0238 | 0.0000 |
| Rv1694 | tlyA | 0 | 26 | 1 | 41 | 0.7860 | -0.0238 | 0.0000 |
| Rv1697 | Rv1697 | 0 | 26 | 1 | 41 | 0.7860 | -0.0238 | 0.0000 |
| Rv1699 | pyrG | 0 | 26 | 1 | 41 | 0.7860 | -0.0238 | 0.0000 |
| Rv1702c | Rv1702c | 0 | 26 | 1 | 41 | 0.7860 | -0.0238 | 0.0000 |
| Rv1703c | Rv1703c | 0 | 26 | 1 | 41 | 0.7860 | -0.0238 | 0.0000 |
| Rv1706c | PPE23 | 0 | 26 | 1 | 41 | 0.7860 | -0.0238 | 0.0000 |
| Rv1751 | Rv1751 | 0 | 26 | 1 | 41 | 0.7860 | -0.0238 | 0.0000 |
| Rv1784 | Rv1784 | 0 | 26 | 1 | 41 | 0.7860 | -0.0238 | 0.0000 |

| Rv1795 | Rv1795 | 0 | 26 | 1 | 41 | 0.7860 | -0.0238 | 0.0000 |
| --- | --- | --- | --- | --- | --- | --- | --- | --- |
| Rv1796 | mycP5 | 0 | 26 | 1 | 41 | 0.7860 | -0.0238 | 0.0000 |
| Rv1814 | erg3 | 0 | 26 | 1 | 41 | 0.7860 | -0.0238 | 0.0000 |
| Rv1816 | Rv1816 | 0 | 26 | 1 | 41 | 0.7860 | -0.0238 | 0.0000 |
| Rv1829 | Rv1829 | 0 | 26 | 1 | 41 | 0.7860 | -0.0238 | 0.0000 |
| Rv1850 | ureC | 0 | 26 | 1 | 41 | 0.7860 | -0.0238 | 0.0000 |
| Rv1864c | Rv1864c | 0 | 26 | 1 | 41 | 0.7860 | -0.0238 | 0.0000 |
| Rv1879 | Rv1879 | 0 | 26 | 1 | 41 | 0.7860 | -0.0238 | 0.0000 |
| Rv1882c | Rv1882c | 0 | 26 | 1 | 41 | 0.7860 | -0.0238 | 0.0000 |
| Rv1888A | Rv1888A | 0 | 26 | 1 | 41 | 0.7860 | -0.0238 | 0.0000 |
| Rv1889c | Rv1889c | 0 | 26 | 1 | 41 | 0.7860 | -0.0238 | 0.0000 |
| Rv1896c | Rv1896c | 0 | 26 | 1 | 41 | 0.7860 | -0.0238 | 0.0000 |
| Rv1909c | furA | 0 | 26 | 1 | 41 | 0.7860 | -0.0238 | 0.0000 |
| Rv1923 | lipD | 0 | 26 | 1 | 41 | 0.7860 | -0.0238 | 0.0000 |
| Rv1929c | Rv1929c | 0 | 26 | 1 | 41 | 0.7860 | -0.0238 | 0.0000 |
| Rv1942c | Rv1942c | 0 | 26 | 1 | 41 | 0.7860 | -0.0238 | 0.0000 |
| Rv1949c | Rv1949c | 0 | 26 | 1 | 41 | 0.7860 | -0.0238 | 0.0000 |
| Rv1956 | Rv1956 | 0 | 26 | 1 | 41 | 0.7860 | -0.0238 | 0.0000 |
| Rv1973 | Rv1973 | 0 | 26 | 1 | 41 | 0.7860 | -0.0238 | 0.0000 |
| Rv1984c | cfp21 | 0 | 26 | 1 | 41 | 0.7860 | -0.0238 | 0.0000 |
| Rv1985c | Rv1985c | 0 | 26 | 1 | 41 | 0.7860 | -0.0238 | 0.0000 |
| Rv1997 | ctpF | 0 | 26 | 1 | 41 | 0.7860 | -0.0238 | 0.0000 |
| Rv2014 | Rv2014 | 0 | 26 | 1 | 41 | 0.7860 | -0.0238 | 0.0000 |
| Rv2027c | Rv2027c | 0 | 26 | 1 | 41 | 0.7860 | -0.0238 | 0.0000 |
| Rv2040c | Rv2040c | 0 | 26 | 1 | 41 | 0.7860 | -0.0238 | 0.0000 |
| Rv2045c | lipT | 0 | 26 | 1 | 41 | 0.7860 | -0.0238 | 0.0000 |
| Rv2095c | Rv2095c | 0 | 26 | 1 | 41 | 0.7860 | -0.0238 | 0.0000 |
| Rv2100 | Rv2100 | 0 | 26 | 1 | 41 | 0.7860 | -0.0238 | 0.0000 |
| Rv2122c | hisE | 0 | 26 | 1 | 41 | 0.7860 | -0.0238 | 0.0000 |
| Rv2123 | PPE37 | 0 | 26 | 1 | 41 | 0.7860 | -0.0238 | 0.0000 |
| Rv2130c | cysS | 0 | 26 | 1 | 41 | 0.7860 | -0.0238 | 0.0000 |
| Rv2136c | uppP | 0 | 26 | 1 | 41 | 0.7860 | -0.0238 | 0.0000 |
| Rv2159c | Rv2159c | 0 | 26 | 1 | 41 | 0.7860 | -0.0238 | 0.0000 |
| Rv2161c | Rv2161c | 0 | 26 | 1 | 41 | 0.7860 | -0.0238 | 0.0000 |

| Rv2186c | Rv2186c | 0 | 26 | 1 | 41 | 0.7860 | -0.0238 | 0.0000 |
| --- | --- | --- | --- | --- | --- | --- | --- | --- |
| Rv2191 | Rv2191 | 0 | 26 | 1 | 41 | 0.7860 | -0.0238 | 0.0000 |
| Rv2196 | qcrB | 0 | 26 | 1 | 41 | 0.7860 | -0.0238 | 0.0000 |
| Rv2197c | Rv2197c | 0 | 26 | 1 | 41 | 0.7860 | -0.0238 | 0.0000 |
| Rv2201 | asnB | 0 | 26 | 1 | 41 | 0.7860 | -0.0238 | 0.0000 |
| Rv2209 | Rv2209 | 0 | 26 | 1 | 41 | 0.7860 | -0.0238 | 0.0000 |
| Rv2216 | Rv2216 | 0 | 26 | 1 | 41 | 0.7860 | -0.0238 | 0.0000 |
| Rv2218 | lipA | 0 | 26 | 1 | 41 | 0.7860 | -0.0238 | 0.0000 |
| Rv2219 | Rv2219 | 0 | 26 | 1 | 41 | 0.7860 | -0.0238 | 0.0000 |
| Rv2220 | glnA1 | 0 | 26 | 1 | 41 | 0.7860 | -0.0238 | 0.0000 |
| Rv2253 | Rv2253 | 0 | 26 | 1 | 41 | 0.7860 | -0.0238 | 0.0000 |
| Rv2257c | Rv2257c | 0 | 26 | 1 | 41 | 0.7860 | -0.0238 | 0.0000 |
| Rv2259 | adhE2 | 0 | 26 | 1 | 41 | 0.7860 | -0.0238 | 0.0000 |
| Rv2268c | cyp128 | 0 | 26 | 1 | 41 | 0.7860 | -0.0238 | 0.0000 |
| Rv2282c | Rv2282c | 0 | 26 | 1 | 41 | 0.7860 | -0.0238 | 0.0000 |
| Rv2294 | Rv2294 | 0 | 26 | 1 | 41 | 0.7860 | -0.0238 | 0.0000 |
| Rv2307B | Rv2307B | 0 | 26 | 1 | 41 | 0.7860 | -0.0238 | 0.0000 |
| Rv2311 | Rv2311 | 0 | 26 | 1 | 41 | 0.7860 | -0.0238 | 0.0000 |
| Rv2315c | Rv2315c | 0 | 26 | 1 | 41 | 0.7860 | -0.0238 | 0.0000 |
| Rv2320c | rocE | 0 | 26 | 1 | 41 | 0.7860 | -0.0238 | 0.0000 |
| Rv2325c | Rv2325c | 0 | 26 | 1 | 41 | 0.7860 | -0.0238 | 0.0000 |
| Rv2336 | Rv2336 | 0 | 26 | 1 | 41 | 0.7860 | -0.0238 | 0.0000 |
| Rv2345 | Rv2345 | 0 | 26 | 1 | 41 | 0.7860 | -0.0238 | 0.0000 |
| Rv2365c | Rv2365c | 0 | 26 | 1 | 41 | 0.7860 | -0.0238 | 0.0000 |
| Rv2399c | cysT | 0 | 26 | 1 | 41 | 0.7860 | -0.0238 | 0.0000 |
| Rv2404c | lepA | 0 | 26 | 1 | 41 | 0.7860 | -0.0238 | 0.0000 |
| Rv2405 | Rv2405 | 0 | 26 | 1 | 41 | 0.7860 | -0.0238 | 0.0000 |
| Rv2416c | eis | 0 | 26 | 1 | 41 | 0.7860 | -0.0238 | 0.0000 |
| Rv2426c | Rv2426c | 0 | 26 | 1 | 41 | 0.7860 | -0.0238 | 0.0000 |
| Rv2449c | Rv2449c | 0 | 26 | 1 | 41 | 0.7860 | -0.0238 | 0.0000 |
| Rv2469c | Rv2469c | 0 | 26 | 1 | 41 | 0.7860 | -0.0238 | 0.0000 |
| Rv2474c | Rv2474c | 0 | 26 | 1 | 41 | 0.7860 | -0.0238 | 0.0000 |
| Rv2481c | Rv2481c | 0 | 26 | 1 | 41 | 0.7860 | -0.0238 | 0.0000 |
| Rv2486 | echA14 | 0 | 26 | 1 | 41 | 0.7860 | -0.0238 | 0.0000 |

| Rv2510c | Rv2510c | 0 | 26 | 1 | 41 | 0.7860 | -0.0238 | 0.0000 |
| --- | --- | --- | --- | --- | --- | --- | --- | --- |
| Rv2528c | mrr | 0 | 26 | 1 | 41 | 0.7860 | -0.0238 | 0.0000 |
| Rv2531c | Rv2531c | 0 | 26 | 1 | 41 | 0.7860 | -0.0238 | 0.0000 |
| Rv2532c | Rv2532c | 0 | 26 | 1 | 41 | 0.7860 | -0.0238 | 0.0000 |
| Rv2539c | aroK | 0 | 26 | 1 | 41 | 0.7860 | -0.0238 | 0.0000 |
| Rv2549c | Rv2549c | 0 | 26 | 1 | 41 | 0.7860 | -0.0238 | 0.0000 |
| Rv2555c | alaS | 0 | 26 | 1 | 41 | 0.7860 | -0.0238 | 0.0000 |
| Rv2558 | Rv2558 | 0 | 26 | 1 | 41 | 0.7860 | -0.0238 | 0.0000 |
| Rv2563 | Rv2563 | 0 | 26 | 1 | 41 | 0.7860 | -0.0238 | 0.0000 |
| Rv2602 | Rv2602 | 0 | 26 | 1 | 41 | 0.7860 | -0.0238 | 0.0000 |
| Rv2615c | PE_PGRS45 | 0 | 26 | 1 | 41 | 0.7860 | -0.0238 | 0.0000 |
| Rv2623 | TB31.7 | 0 | 26 | 1 | 41 | 0.7860 | -0.0238 | 0.0000 |
| Rv2630 | Rv2630 | 0 | 26 | 1 | 41 | 0.7860 | -0.0238 | 0.0000 |
| Rv2636 | Rv2636 | 0 | 26 | 1 | 41 | 0.7860 | -0.0238 | 0.0000 |
| Rv2644c | Rv2644c | 0 | 26 | 1 | 41 | 0.7860 | -0.0238 | 0.0000 |
| Rv2645 | Rv2645 | 0 | 26 | 1 | 41 | 0.7860 | -0.0238 | 0.0000 |
| Rv2655c | Rv2655c | 0 | 26 | 1 | 41 | 0.7860 | -0.0238 | 0.0000 |
| Rv2656c | Rv2656c | 0 | 26 | 1 | 41 | 0.7860 | -0.0238 | 0.0000 |
| Rv2659c | Rv2659c | 0 | 26 | 1 | 41 | 0.7860 | -0.0238 | 0.0000 |
| Rv2667 | clpC2 | 0 | 26 | 1 | 41 | 0.7860 | -0.0238 | 0.0000 |
| Rv2674 | Rv2674 | 0 | 26 | 1 | 41 | 0.7860 | -0.0238 | 0.0000 |
| Rv2683 | Rv2683 | 0 | 26 | 1 | 41 | 0.7860 | -0.0238 | 0.0000 |
| Rv2686c | Rv2686c | 0 | 26 | 1 | 41 | 0.7860 | -0.0238 | 0.0000 |
| Rv2696c | Rv2696c | 0 | 26 | 1 | 41 | 0.7860 | -0.0238 | 0.0000 |
| Rv2700 | Rv2700 | 0 | 26 | 1 | 41 | 0.7860 | -0.0238 | 0.0000 |
| Rv2721c | Rv2721c | 0 | 26 | 1 | 41 | 0.7860 | -0.0238 | 0.0000 |
| Rv2723 | Rv2723 | 0 | 26 | 1 | 41 | 0.7860 | -0.0238 | 0.0000 |
| Rv2728c | Rv2728c | 0 | 26 | 1 | 41 | 0.7860 | -0.0238 | 0.0000 |
| Rv2775 | Rv2775 | 0 | 26 | 1 | 41 | 0.7860 | -0.0238 | 0.0000 |
| Rv2789c | fadE21 | 0 | 26 | 1 | 41 | 0.7860 | -0.0238 | 0.0000 |
| Rv2795c | Rv2795c | 0 | 26 | 1 | 41 | 0.7860 | -0.0238 | 0.0000 |
| Rv2796c | lppV | 0 | 26 | 1 | 41 | 0.7860 | -0.0238 | 0.0000 |
| Rv2818c | Rv2818c | 0 | 26 | 1 | 41 | 0.7860 | -0.0238 | 0.0000 |
| Rv2827c | Rv2827c | 0 | 26 | 1 | 41 | 0.7860 | -0.0238 | 0.0000 |

| Rv2845c | proS | 0 | 26 | 1 | 41 | 0.7860 | -0.0238 | 0.0000 |
| --- | --- | --- | --- | --- | --- | --- | --- | --- |
| Rv2847c | cysG | 0 | 26 | 1 | 41 | 0.7860 | -0.0238 | 0.0000 |
| Rv2859c | Rv2859c | 0 | 26 | 1 | 41 | 0.7860 | -0.0238 | 0.0000 |
| Rv2861c | mapB | 0 | 26 | 1 | 41 | 0.7860 | -0.0238 | 0.0000 |
| Rv2872 | Rv2872 | 0 | 26 | 1 | 41 | 0.7860 | -0.0238 | 0.0000 |
| Rv2873 | mpt83 | 0 | 26 | 1 | 41 | 0.7860 | -0.0238 | 0.0000 |
| Rv2883c | pyrH | 0 | 26 | 1 | 41 | 0.7860 | -0.0238 | 0.0000 |
| Rv2908c | Rv2908c | 0 | 26 | 1 | 41 | 0.7860 | -0.0238 | 0.0000 |
| Rv2910c | Rv2910c | 0 | 26 | 1 | 41 | 0.7860 | -0.0238 | 0.0000 |
| Rv2916c | ffh | 0 | 26 | 1 | 41 | 0.7860 | -0.0238 | 0.0000 |
| Rv2928 | tesA | 0 | 26 | 1 | 41 | 0.7860 | -0.0238 | 0.0000 |
| Rv2939 | papA5 | 0 | 26 | 1 | 41 | 0.7860 | -0.0238 | 0.0000 |
| Rv2942 | mmpL7 | 0 | 26 | 1 | 41 | 0.7860 | -0.0238 | 0.0000 |
| Rv2945c | lppX | 0 | 26 | 1 | 41 | 0.7860 | -0.0238 | 0.0000 |
| Rv2953 | Rv2953 | 0 | 26 | 1 | 41 | 0.7860 | -0.0238 | 0.0000 |
| Rv2954c | Rv2954c | 0 | 26 | 1 | 41 | 0.7860 | -0.0238 | 0.0000 |
| Rv2957 | Rv2957 | 0 | 26 | 1 | 41 | 0.7860 | -0.0238 | 0.0000 |
| Rv2978c | Rv2978c | 0 | 26 | 1 | 41 | 0.7860 | -0.0238 | 0.0000 |
| Rv2989 | Rv2989 | 0 | 26 | 1 | 41 | 0.7860 | -0.0238 | 0.0000 |
| Rv3001c | ilvC | 0 | 26 | 1 | 41 | 0.7860 | -0.0238 | 0.0000 |
| Rv3012c | gatC | 0 | 26 | 1 | 41 | 0.7860 | -0.0238 | 0.0000 |
| Rv3022A | PE29 | 0 | 26 | 1 | 41 | 0.7860 | -0.0238 | 0.0000 |
| Rv3025c | iscS | 0 | 26 | 1 | 41 | 0.7860 | -0.0238 | 0.0000 |
| Rv3026c | Rv3026c | 0 | 26 | 1 | 41 | 0.7860 | -0.0238 | 0.0000 |
| Rv3028c | fixB | 0 | 26 | 1 | 41 | 0.7860 | -0.0238 | 0.0000 |
| Rv3030 | Rv3030 | 0 | 26 | 1 | 41 | 0.7860 | -0.0238 | 0.0000 |
| Rv3031 | Rv3031 | 0 | 26 | 1 | 41 | 0.7860 | -0.0238 | 0.0000 |
| Rv3038c | Rv3038c | 0 | 26 | 1 | 41 | 0.7860 | -0.0238 | 0.0000 |
| Rv3046c | Rv3046c | 0 | 26 | 1 | 41 | 0.7860 | -0.0238 | 0.0000 |
| Rv3055 | Rv3055 | 0 | 26 | 1 | 41 | 0.7860 | -0.0238 | 0.0000 |
| Rv3072c | Rv3072c | 0 | 26 | 1 | 41 | 0.7860 | -0.0238 | 0.0000 |
| Rv3103c | Rv3103c | 0 | 26 | 1 | 41 | 0.7860 | -0.0238 | 0.0000 |
| Rv3105c | prfB | 0 | 26 | 1 | 41 | 0.7860 | -0.0238 | 0.0000 |
| Rv3109 | moaA1 | 0 | 26 | 1 | 41 | 0.7860 | -0.0238 | 0.0000 |

| Rv3116 | moeB2 | 0 | 26 | 1 | 41 | 0.7860 | -0.0238 | 0.0000 |
| --- | --- | --- | --- | --- | --- | --- | --- | --- |
| Rv3129 | Rv3129 | 0 | 26 | 1 | 41 | 0.7860 | -0.0238 | 0.0000 |
| Rv3136 | PPE51 | 0 | 26 | 1 | 41 | 0.7860 | -0.0238 | 0.0000 |
| Rv3138 | pflA | 0 | 26 | 1 | 41 | 0.7860 | -0.0238 | 0.0000 |
| Rv3143 | Rv3143 | 0 | 26 | 1 | 41 | 0.7860 | -0.0238 | 0.0000 |
| Rv3167c | Rv3167c | 0 | 26 | 1 | 41 | 0.7860 | -0.0238 | 0.0000 |
| Rv3178 | Rv3178 | 0 | 26 | 1 | 41 | 0.7860 | -0.0238 | 0.0000 |
| Rv3182 | Rv3182 | 0 | 26 | 1 | 41 | 0.7860 | -0.0238 | 0.0000 |
| Rv3212 | Rv3212 | 0 | 26 | 1 | 41 | 0.7860 | -0.0238 | 0.0000 |
| Rv3216 | Rv3216 | 0 | 26 | 1 | 41 | 0.7860 | -0.0238 | 0.0000 |
| Rv3221A | Rv3221A | 0 | 26 | 1 | 41 | 0.7860 | -0.0238 | 0.0000 |
| Rv3226c | Rv3226c | 0 | 26 | 1 | 41 | 0.7860 | -0.0238 | 0.0000 |
| Rv3240c | secA1 | 0 | 26 | 1 | 41 | 0.7860 | -0.0238 | 0.0000 |
| Rv3243c | Rv3243c | 0 | 26 | 1 | 41 | 0.7860 | -0.0238 | 0.0000 |
| Rv3244c | lpqB | 0 | 26 | 1 | 41 | 0.7860 | -0.0238 | 0.0000 |
| Rv3247c | tmk | 0 | 26 | 1 | 41 | 0.7860 | -0.0238 | 0.0000 |
| Rv3254 | Rv3254 | 0 | 26 | 1 | 41 | 0.7860 | -0.0238 | 0.0000 |
| Rv3266c | rmlD | 0 | 26 | 1 | 41 | 0.7860 | -0.0238 | 0.0000 |
| Rv3273 | Rv3273 | 0 | 26 | 1 | 41 | 0.7860 | -0.0238 | 0.0000 |
| Rv3274c | fadE25 | 0 | 26 | 1 | 41 | 0.7860 | -0.0238 | 0.0000 |
| Rv3292 | Rv3292 | 0 | 26 | 1 | 41 | 0.7860 | -0.0238 | 0.0000 |
| Rv3324c | moaC | 0 | 26 | 1 | 41 | 0.7860 | -0.0238 | 0.0000 |
| Rv3333c | Rv3333c | 0 | 26 | 1 | 41 | 0.7860 | -0.0238 | 0.0000 |
| Rv3339c | icd1 | 0 | 26 | 1 | 41 | 0.7860 | -0.0238 | 0.0000 |
| Rv3367 | PE_PGRS51 | 0 | 26 | 1 | 41 | 0.7860 | -0.0238 | 0.0000 |
| Rv3371 | Rv3371 | 0 | 26 | 1 | 41 | 0.7860 | -0.0238 | 0.0000 |
| Rv3372 | otsB2 | 0 | 26 | 1 | 41 | 0.7860 | -0.0238 | 0.0000 |
| Rv3382c | lytB1 | 0 | 26 | 1 | 41 | 0.7860 | -0.0238 | 0.0000 |
| Rv3406 | Rv3406 | 0 | 26 | 1 | 41 | 0.7860 | -0.0238 | 0.0000 |
| Rv3409c | choD | 0 | 26 | 1 | 41 | 0.7860 | -0.0238 | 0.0000 |
| Rv3413c | Rv3413c | 0 | 26 | 1 | 41 | 0.7860 | -0.0238 | 0.0000 |
| Rv3415c | Rv3415c | 0 | 26 | 1 | 41 | 0.7860 | -0.0238 | 0.0000 |
| Rv3417c | groEL | 0 | 26 | 1 | 41 | 0.7860 | -0.0238 | 0.0000 |
| Rv3426 | PPE58 | 0 | 26 | 1 | 41 | 0.7860 | -0.0238 | 0.0000 |

| Rv3432c | gadB | 0 | 26 | 1 | 41 | 0.7860 | -0.0238 | 0.0000 |
| --- | --- | --- | --- | --- | --- | --- | --- | --- |
| Rv3442c | rpsI | 0 | 26 | 1 | 41 | 0.7860 | -0.0238 | 0.0000 |
| Rv3450c | Rv3450c | 0 | 26 | 1 | 41 | 0.7860 | -0.0238 | 0.0000 |
| Rv3455c | truA | 0 | 26 | 1 | 41 | 0.7860 | -0.0238 | 0.0000 |
| Rv3482c | Rv3482c | 0 | 26 | 1 | 41 | 0.7860 | -0.0238 | 0.0000 |
| Rv3504 | fadE26 | 0 | 26 | 1 | 41 | 0.7860 | -0.0238 | 0.0000 |
| Rv3510c | Rv3510c | 0 | 26 | 1 | 41 | 0.7860 | -0.0238 | 0.0000 |
| Rv3525c | Rv3525c | 0 | 26 | 1 | 41 | 0.7860 | -0.0238 | 0.0000 |
| Rv3551 | Rv3551 | 0 | 26 | 1 | 41 | 0.7860 | -0.0238 | 0.0000 |
| Rv3555c | Rv3555c | 0 | 26 | 1 | 41 | 0.7860 | -0.0238 | 0.0000 |
| Rv3556c | fadA6 | 0 | 26 | 1 | 41 | 0.7860 | -0.0238 | 0.0000 |
| Rv3560c | fadE30 | 0 | 26 | 1 | 41 | 0.7860 | -0.0238 | 0.0000 |
| Rv3562 | fadE31 | 0 | 26 | 1 | 41 | 0.7860 | -0.0238 | 0.0000 |
| Rv3576 | lppH | 0 | 26 | 1 | 41 | 0.7860 | -0.0238 | 0.0000 |
| Rv3577 | Rv3577 | 0 | 26 | 1 | 41 | 0.7860 | -0.0238 | 0.0000 |
| Rv3589 | mutY | 0 | 26 | 1 | 41 | 0.7860 | -0.0238 | 0.0000 |
| Rv3604c | Rv3604c | 0 | 26 | 1 | 41 | 0.7860 | -0.0238 | 0.0000 |
| Rv3621c | PPE65 | 0 | 26 | 1 | 41 | 0.7860 | -0.0238 | 0.0000 |
| Rv3623 | lpqG | 0 | 26 | 1 | 41 | 0.7860 | -0.0238 | 0.0000 |
| Rv3626c | Rv3626c | 0 | 26 | 1 | 41 | 0.7860 | -0.0238 | 0.0000 |
| Rv3640c | Rv3640c | 0 | 26 | 1 | 41 | 0.7860 | -0.0238 | 0.0000 |
| Rv3645 | Rv3645 | 0 | 26 | 1 | 41 | 0.7860 | -0.0238 | 0.0000 |
| Rv3647c | Rv3647c | 0 | 26 | 1 | 41 | 0.7860 | -0.0238 | 0.0000 |
| Rv3648c | cspA | 0 | 26 | 1 | 41 | 0.7860 | -0.0238 | 0.0000 |
| Rv3652 | PE_PGRS60 | 0 | 26 | 1 | 41 | 0.7860 | -0.0238 | 0.0000 |
| Rv3658c | Rv3658c | 0 | 26 | 1 | 41 | 0.7860 | -0.0238 | 0.0000 |
| Rv3661 | Rv3661 | 0 | 26 | 1 | 41 | 0.7860 | -0.0238 | 0.0000 |
| Rv3663c | dppD | 0 | 26 | 1 | 41 | 0.7860 | -0.0238 | 0.0000 |
| Rv3668c | Rv3668c | 0 | 26 | 1 | 41 | 0.7860 | -0.0238 | 0.0000 |
| Rv3669 | Rv3669 | 0 | 26 | 1 | 41 | 0.7860 | -0.0238 | 0.0000 |
| Rv3687c | rsfB | 0 | 26 | 1 | 41 | 0.7860 | -0.0238 | 0.0000 |
| Rv3695 | Rv3695 | 0 | 26 | 1 | 41 | 0.7860 | -0.0238 | 0.0000 |
| Rv3697c | Rv3697c | 0 | 26 | 1 | 41 | 0.7860 | -0.0238 | 0.0000 |
| Rv3706c | Rv3706c | 0 | 26 | 1 | 41 | 0.7860 | -0.0238 | 0.0000 |

| Rv3716c | Rv3716c | 0 | 26 | 1 | 41 | 0.7860 | -0.0238 | 0.0000 |
| --- | --- | --- | --- | --- | --- | --- | --- | --- |
| Rv3728 | Rv3728 | 0 | 26 | 1 | 41 | 0.7860 | -0.0238 | 0.0000 |
| Rv3744 | Rv3744 | 0 | 26 | 1 | 41 | 0.7860 | -0.0238 | 0.0000 |
| Rv3754 | tyrA | 0 | 26 | 1 | 41 | 0.7860 | -0.0238 | 0.0000 |
| Rv3755c | Rv3755c | 0 | 26 | 1 | 41 | 0.7860 | -0.0238 | 0.0000 |
| Rv3780 | Rv3780 | 0 | 26 | 1 | 41 | 0.7860 | -0.0238 | 0.0000 |
| Rv3785 | Rv3785 | 0 | 26 | 1 | 41 | 0.7860 | -0.0238 | 0.0000 |
| Rv3812 | PE_PGRS62 | 0 | 26 | 1 | 41 | 0.7860 | -0.0238 | 0.0000 |
| Rv3813c | Rv3813c | 0 | 26 | 1 | 41 | 0.7860 | -0.0238 | 0.0000 |
| Rv3815c | Rv3815c | 0 | 26 | 1 | 41 | 0.7860 | -0.0238 | 0.0000 |
| Rv3819 | Rv3819 | 0 | 26 | 1 | 41 | 0.7860 | -0.0238 | 0.0000 |
| Rv3821 | Rv3821 | 0 | 26 | 1 | 41 | 0.7860 | -0.0238 | 0.0000 |
| Rv3829c | Rv3829c | 0 | 26 | 1 | 41 | 0.7860 | -0.0238 | 0.0000 |
| Rv3830c | Rv3830c | 0 | 26 | 1 | 41 | 0.7860 | -0.0238 | 0.0000 |
| Rv3834c | serS | 0 | 26 | 1 | 41 | 0.7860 | -0.0238 | 0.0000 |
| Rv3847 | Rv3847 | 0 | 26 | 1 | 41 | 0.7860 | -0.0238 | 0.0000 |
| Rv3857c | Rv3857c | 0 | 26 | 1 | 41 | 0.7860 | -0.0238 | 0.0000 |
| Rv3858c | gltD | 0 | 26 | 1 | 41 | 0.7860 | -0.0238 | 0.0000 |
| Rv3862c | whiB6 | 0 | 26 | 1 | 41 | 0.7860 | -0.0238 | 0.0000 |
| Rv3865 | Rv3865 | 0 | 26 | 1 | 41 | 0.7860 | -0.0238 | 0.0000 |
| Rv3867 | Rv3867 | 0 | 26 | 1 | 41 | 0.7860 | -0.0238 | 0.0000 |
| Rv3885c | Rv3885c | 0 | 26 | 1 | 41 | 0.7860 | -0.0238 | 0.0000 |
| Rv3905c | esxF | 0 | 26 | 1 | 41 | 0.7860 | -0.0238 | 0.0000 |
| Rv3914 | trxC | 0 | 26 | 1 | 41 | 0.7860 | -0.0238 | 0.0000 |
| Rv3917c | parB | 0 | 26 | 1 | 41 | 0.7860 | -0.0238 | 0.0000 |
| Rv4002 | Rv1118c | 0 | 26 | 1 | 41 | 0.7860 | -0.0238 | 0.0000 |
| Rv4004 | Rv4004 | 0 | 26 | 1 | 41 | 0.7860 | -0.0238 | 0.0000 |
| Rv4013 | Rv4013 | 0 | 26 | 1 | 41 | 0.7860 | -0.0238 | 0.0000 |
| Rv0486 | Rv0486 | 8 | 18 | 14 | 28 | 0.5869 | -0.0256 | 0.8889 |
| Rv0808 | purF | 8 | 18 | 14 | 28 | 0.5869 | -0.0256 | 0.8889 |
| Rv1461 | Rv1461 | 8 | 18 | 14 | 28 | 0.5869 | -0.0256 | 0.8889 |
| Rv1505c | Rv1505c | 8 | 18 | 14 | 28 | 0.5869 | -0.0256 | 0.8889 |
| Rv0014c | pknB | 3 | 23 | 6 | 36 | 0.6274 | -0.0275 | 0.7826 |
| Rv0136 | cyp138 | 3 | 23 | 6 | 36 | 0.6274 | -0.0275 | 0.7826 |

| Rv0419 | lpqM | 3 | 23 | 6 | 36 | 0.6274 | -0.0275 | 0.7826 |
| --- | --- | --- | --- | --- | --- | --- | --- | --- |
| Rv3805c | Rv3805c | 3 | 23 | 6 | 36 | 0.6274 | -0.0275 | 0.7826 |
| Rv0097 | Rv0097 | 6 | 20 | 11 | 31 | 0.6134 | -0.0311 | 0.8455 |
| Rv0365c | Rv0365c | 6 | 20 | 11 | 31 | 0.6134 | -0.0311 | 0.8455 |
| Rv0415 | thiO | 6 | 20 | 11 | 31 | 0.6134 | -0.0311 | 0.8455 |
| Rv0437c | psd | 6 | 20 | 11 | 31 | 0.6134 | -0.0311 | 0.8455 |
| Rv0578c | PE_PGRS7 | 6 | 20 | 11 | 31 | 0.6134 | -0.0311 | 0.8455 |
| Rv0741 | Rv0741 | 6 | 20 | 11 | 31 | 0.6134 | -0.0311 | 0.8455 |
| Rv1016c | lpqT | 6 | 20 | 11 | 31 | 0.6134 | -0.0311 | 0.8455 |
| Rv1029 | kdpA | 6 | 20 | 11 | 31 | 0.6134 | -0.0311 | 0.8455 |
| Rv1249c | Rv1249c | 6 | 20 | 11 | 31 | 0.6134 | -0.0311 | 0.8455 |
| Rv1553 | frdB | 6 | 20 | 11 | 31 | 0.6134 | -0.0311 | 0.8455 |
| Rv1557 | mmpL6 | 6 | 20 | 11 | 31 | 0.6134 | -0.0311 | 0.8455 |
| Rv1571 | Rv1571 | 6 | 20 | 11 | 31 | 0.6134 | -0.0311 | 0.8455 |
| Rv1788 | PE18 | 6 | 20 | 11 | 31 | 0.6134 | -0.0311 | 0.8455 |
| Rv2147c | Rv2147c | 6 | 20 | 11 | 31 | 0.6134 | -0.0311 | 0.8455 |
| Rv2296 | Rv2296 | 6 | 20 | 11 | 31 | 0.6134 | -0.0311 | 0.8455 |
| Rv2503c | scoB | 6 | 20 | 11 | 31 | 0.6134 | -0.0311 | 0.8455 |
| Rv2709 | Rv2709 | 6 | 20 | 11 | 31 | 0.6134 | -0.0311 | 0.8455 |
| Rv2761c | hsdS | 6 | 20 | 11 | 31 | 0.6134 | -0.0311 | 0.8455 |
| Rv2787 | Rv2787 | 6 | 20 | 11 | 31 | 0.6134 | -0.0311 | 0.8455 |
| Rv2875 | mpt70 | 6 | 20 | 11 | 31 | 0.6134 | -0.0311 | 0.8455 |
| Rv2923c | acyP | 6 | 20 | 11 | 31 | 0.6134 | -0.0311 | 0.8455 |
| Rv3306c | amiB1 | 6 | 20 | 11 | 31 | 0.6134 | -0.0311 | 0.8455 |
| Rv3478 | PPE60 | 6 | 20 | 11 | 31 | 0.6134 | -0.0311 | 0.8455 |
| Rv3533c | PPE62 | 6 | 20 | 11 | 31 | 0.6134 | -0.0311 | 0.8455 |
| Rv0063 | Rv0063 | 1 | 25 | 3 | 39 | 0.7128 | -0.0330 | 0.5200 |
| Rv0070c | glyA | 1 | 25 | 3 | 39 | 0.7128 | -0.0330 | 0.5200 |
| Rv0162c | adhE1 | 1 | 25 | 3 | 39 | 0.7128 | -0.0330 | 0.5200 |
| Rv0656c | Rv0656c | 1 | 25 | 3 | 39 | 0.7128 | -0.0330 | 0.5200 |
| Rv0687 | fabG | 1 | 25 | 3 | 39 | 0.7128 | -0.0330 | 0.5200 |
| Rv0755c | PPE12 | 1 | 25 | 3 | 39 | 0.7128 | -0.0330 | 0.5200 |
| Rv0822c | Rv0822c | 1 | 25 | 3 | 39 | 0.7128 | -0.0330 | 0.5200 |
| Rv0992c | Rv0992c | 1 | 25 | 3 | 39 | 0.7128 | -0.0330 | 0.5200 |

| Rv1057 | Rv1057 | 1 | 25 | 3 | 39 | 0.7128 | -0.0330 | 0.5200 |
| --- | --- | --- | --- | --- | --- | --- | --- | --- |
| Rv1144 | Rv1144 | 1 | 25 | 3 | 39 | 0.7128 | -0.0330 | 0.5200 |
| Rv1145 | mmpL13a | 1 | 25 | 3 | 39 | 0.7128 | -0.0330 | 0.5200 |
| Rv1223 | htrA | 1 | 25 | 3 | 39 | 0.7128 | -0.0330 | 0.5200 |
| Rv1284 | Rv1284 | 1 | 25 | 3 | 39 | 0.7128 | -0.0330 | 0.5200 |
| Rv1484 | inhA | 1 | 25 | 3 | 39 | 0.7128 | -0.0330 | 0.5200 |
| Rv1540 | Rv1540 | 1 | 25 | 3 | 39 | 0.7128 | -0.0330 | 0.5200 |
| Rv1735c | Rv1735c | 1 | 25 | 3 | 39 | 0.7128 | -0.0330 | 0.5200 |
| Rv1834 | Rv1834 | 1 | 25 | 3 | 39 | 0.7128 | -0.0330 | 0.5200 |
| Rv2185c | TB16.3 | 1 | 25 | 3 | 39 | 0.7128 | -0.0330 | 0.5200 |
| Rv2622 | Rv2622 | 1 | 25 | 3 | 39 | 0.7128 | -0.0330 | 0.5200 |
| Rv2868c | ispG | 1 | 25 | 3 | 39 | 0.7128 | -0.0330 | 0.5200 |
| Rv2894c | xerC | 1 | 25 | 3 | 39 | 0.7128 | -0.0330 | 0.5200 |
| Rv3008 | Rv3008 | 1 | 25 | 3 | 39 | 0.7128 | -0.0330 | 0.5200 |
| Rv3024c | mnmA | 1 | 25 | 3 | 39 | 0.7128 | -0.0330 | 0.5200 |
| Rv3078 | hab | 1 | 25 | 3 | 39 | 0.7128 | -0.0330 | 0.5200 |
| Rv3111 | moaC | 1 | 25 | 3 | 39 | 0.7128 | -0.0330 | 0.5200 |
| Rv3119 | moaE1 | 1 | 25 | 3 | 39 | 0.7128 | -0.0330 | 0.5200 |
| Rv3414c | sigD | 1 | 25 | 3 | 39 | 0.7128 | -0.0330 | 0.5200 |
| Rv3438 | Rv3438 | 1 | 25 | 3 | 39 | 0.7128 | -0.0330 | 0.5200 |
| Rv3469c | mhpE | 1 | 25 | 3 | 39 | 0.7128 | -0.0330 | 0.5200 |
| Rv3566c | nat | 1 | 25 | 3 | 39 | 0.7128 | -0.0330 | 0.5200 |
| Rv3710 | leuA | 1 | 25 | 3 | 39 | 0.7128 | -0.0330 | 0.5200 |
| Rv3876 | Rv3876 | 1 | 25 | 3 | 39 | 0.7128 | -0.0330 | 0.5200 |
| Rv0050 | ponA1 | 14 | 12 | 24 | 18 | 0.6049 | -0.0330 | 0.8750 |
| Rv0324 | Rv0324 | 9 | 17 | 16 | 26 | 0.6138 | -0.0348 | 0.8603 |
| Rv0436c | pssA | 9 | 17 | 16 | 26 | 0.6138 | -0.0348 | 0.8603 |
| Rv0565c | Rv0565c | 9 | 17 | 16 | 26 | 0.6138 | -0.0348 | 0.8603 |
| Rv0619 | galTb | 9 | 17 | 16 | 26 | 0.6138 | -0.0348 | 0.8603 |
| Rv1127c | ppdK | 9 | 17 | 16 | 26 | 0.6138 | -0.0348 | 0.8603 |
| Rv1218c | Rv1218c | 9 | 17 | 16 | 26 | 0.6138 | -0.0348 | 0.8603 |
| Rv1448c | tal | 9 | 17 | 16 | 26 | 0.6138 | -0.0348 | 0.8603 |
| Rv1517 | Rv1517 | 9 | 17 | 16 | 26 | 0.6138 | -0.0348 | 0.8603 |
| Rv1592c | Rv1592c | 9 | 17 | 16 | 26 | 0.6138 | -0.0348 | 0.8603 |

| Rv1915 | aceAa | 9 | 17 | 16 | 26 | 0.6138 | -0.0348 | 0.8603 |
| --- | --- | --- | --- | --- | --- | --- | --- | --- |
| Rv2109c | prcA | 9 | 17 | 16 | 26 | 0.6138 | -0.0348 | 0.8603 |
| Rv2307c | Rv2307c | 9 | 17 | 16 | 26 | 0.6138 | -0.0348 | 0.8603 |
| Rv2316 | uspA | 9 | 17 | 16 | 26 | 0.6138 | -0.0348 | 0.8603 |
| Rv2333c | Rv2333c | 9 | 17 | 16 | 26 | 0.6138 | -0.0348 | 0.8603 |
| Rv2360c | Rv2360c | 9 | 17 | 16 | 26 | 0.6138 | -0.0348 | 0.8603 |
| Rv2439c | proB | 9 | 17 | 16 | 26 | 0.6138 | -0.0348 | 0.8603 |
| Rv2494 | Rv2494 | 9 | 17 | 16 | 26 | 0.6138 | -0.0348 | 0.8603 |
| Rv2756c | hsdM | 9 | 17 | 16 | 26 | 0.6138 | -0.0348 | 0.8603 |
| Rv3113 | Rv3113 | 9 | 17 | 16 | 26 | 0.6138 | -0.0348 | 0.8603 |
| Rv3777 | Rv3777 | 9 | 17 | 16 | 26 | 0.6138 | -0.0348 | 0.8603 |
| Rv3910 | Rv3910 | 9 | 17 | 16 | 26 | 0.6138 | -0.0348 | 0.8603 |
| Rv1037c | esxI | 4 | 22 | 8 | 34 | 0.6499 | -0.0366 | 0.7727 |
| Rv1785c | cyp143 | 4 | 22 | 8 | 34 | 0.6499 | -0.0366 | 0.7727 |
| Rv2455c | Rv2455c | 4 | 22 | 8 | 34 | 0.6499 | -0.0366 | 0.7727 |
| Rv2627c | Rv2627c | 25 | 1 | 42 | 0 | 0.8998 | -0.0385 | 0.0000 |
| Rv3347c | PPE55 | 20 | 6 | 34 | 8 | 0.6552 | -0.0403 | 0.7843 |
| Rv0026 | Rv0026 | 7 | 19 | 13 | 29 | 0.6385 | -0.0403 | 0.8219 |
| Rv0770 | Rv0770 | 7 | 19 | 13 | 29 | 0.6385 | -0.0403 | 0.8219 |
| Rv0803 | purL | 7 | 19 | 13 | 29 | 0.6385 | -0.0403 | 0.8219 |
| Rv1328 | glgP | 7 | 19 | 13 | 29 | 0.6385 | -0.0403 | 0.8219 |
| Rv1982c | Rv1982c | 7 | 19 | 13 | 29 | 0.6385 | -0.0403 | 0.8219 |
| Rv2067c | Rv2067c | 7 | 19 | 13 | 29 | 0.6385 | -0.0403 | 0.8219 |
| Rv2394 | ggtB | 7 | 19 | 13 | 29 | 0.6385 | -0.0403 | 0.8219 |
| Rv3435c | Rv3435c | 7 | 19 | 13 | 29 | 0.6385 | -0.0403 | 0.8219 |
| Rv0104 | Rv0104 | 2 | 24 | 5 | 37 | 0.7107 | -0.0421 | 0.6167 |
| Rv0191 | Rv0191 | 2 | 24 | 5 | 37 | 0.7107 | -0.0421 | 0.6167 |
| Rv0245 | Rv0245 | 2 | 24 | 5 | 37 | 0.7107 | -0.0421 | 0.6167 |
| Rv0276 | Rv0276 | 2 | 24 | 5 | 37 | 0.7107 | -0.0421 | 0.6167 |
| Rv0404 | fadD30 | 2 | 24 | 5 | 37 | 0.7107 | -0.0421 | 0.6167 |
| Rv0536 | galE3 | 2 | 24 | 5 | 37 | 0.7107 | -0.0421 | 0.6167 |
| Rv0629c | recD | 2 | 24 | 5 | 37 | 0.7107 | -0.0421 | 0.6167 |
| Rv0663 | atsD | 2 | 24 | 5 | 37 | 0.7107 | -0.0421 | 0.6167 |
| Rv0773c | ggtA | 2 | 24 | 5 | 37 | 0.7107 | -0.0421 | 0.6167 |

| Rv0845 | Rv0845 | 2 | 24 | 5 | 37 | 0.7107 | -0.0421 | 0.6167 |
| --- | --- | --- | --- | --- | --- | --- | --- | --- |
| Rv0855 | far | 2 | 24 | 5 | 37 | 0.7107 | -0.0421 | 0.6167 |
| Rv0858c | Rv0858c | 2 | 24 | 5 | 37 | 0.7107 | -0.0421 | 0.6167 |
| Rv0918 | Rv0918 | 2 | 24 | 5 | 37 | 0.7107 | -0.0421 | 0.6167 |
| Rv1206 | fadD6 | 2 | 24 | 5 | 37 | 0.7107 | -0.0421 | 0.6167 |
| Rv1368 | lprF | 2 | 24 | 5 | 37 | 0.7107 | -0.0421 | 0.6167 |
| Rv1441c | PE_PGRS26 | 2 | 24 | 5 | 37 | 0.7107 | -0.0421 | 0.6167 |
| Rv1522c | mmpL12 | 2 | 24 | 5 | 37 | 0.7107 | -0.0421 | 0.6167 |
| Rv1629 | polA | 2 | 24 | 5 | 37 | 0.7107 | -0.0421 | 0.6167 |
| Rv1916 | aceAb | 2 | 24 | 5 | 37 | 0.7107 | -0.0421 | 0.6167 |
| Rv1966 | mce3A | 2 | 24 | 5 | 37 | 0.7107 | -0.0421 | 0.6167 |
| Rv1978 | Rv1978 | 2 | 24 | 5 | 37 | 0.7107 | -0.0421 | 0.6167 |
| Rv2000 | Rv2000 | 2 | 24 | 5 | 37 | 0.7107 | -0.0421 | 0.6167 |
| Rv2015c | Rv2015c | 2 | 24 | 5 | 37 | 0.7107 | -0.0421 | 0.6167 |
| Rv2180c | Rv2180c | 2 | 24 | 5 | 37 | 0.7107 | -0.0421 | 0.6167 |
| Rv2247 | accD6 | 2 | 24 | 5 | 37 | 0.7107 | -0.0421 | 0.6167 |
| Rv2290 | lppO | 2 | 24 | 5 | 37 | 0.7107 | -0.0421 | 0.6167 |
| Rv2349c | plcC | 2 | 24 | 5 | 37 | 0.7107 | -0.0421 | 0.6167 |
| Rv2391 | nirA | 2 | 24 | 5 | 37 | 0.7107 | -0.0421 | 0.6167 |
| Rv2488c | Rv2488c | 2 | 24 | 5 | 37 | 0.7107 | -0.0421 | 0.6167 |
| Rv2519 | PE26 | 2 | 24 | 5 | 37 | 0.7107 | -0.0421 | 0.6167 |
| Rv2693c | Rv2693c | 2 | 24 | 5 | 37 | 0.7107 | -0.0421 | 0.6167 |
| Rv2941 | fadD28 | 2 | 24 | 5 | 37 | 0.7107 | -0.0421 | 0.6167 |
| Rv3051c | nrdE | 2 | 24 | 5 | 37 | 0.7107 | -0.0421 | 0.6167 |
| Rv3059 | cyp136 | 2 | 24 | 5 | 37 | 0.7107 | -0.0421 | 0.6167 |
| Rv3201c | Rv3201c | 2 | 24 | 5 | 37 | 0.7107 | -0.0421 | 0.6167 |
| Rv3282 | maf | 2 | 24 | 5 | 37 | 0.7107 | -0.0421 | 0.6167 |
| Rv3296 | lhr | 2 | 24 | 5 | 37 | 0.7107 | -0.0421 | 0.6167 |
| Rv3465 | rmlC | 2 | 24 | 5 | 37 | 0.7107 | -0.0421 | 0.6167 |
| Rv3537 | Rv3537 | 2 | 24 | 5 | 37 | 0.7107 | -0.0421 | 0.6167 |
| Rv3540c | ltp2 | 2 | 24 | 5 | 37 | 0.7107 | -0.0421 | 0.6167 |
| Rv3563 | fadE32 | 2 | 24 | 5 | 37 | 0.7107 | -0.0421 | 0.6167 |
| Rv3598c | lysS | 2 | 24 | 5 | 37 | 0.7107 | -0.0421 | 0.6167 |
| Rv3801c | fadD32 | 2 | 24 | 5 | 37 | 0.7107 | -0.0421 | 0.6167 |

| Rv3826 | fadD23 | 2 | 24 | 5 | 37 | 0.7107 | -0.0421 | 0.6167 |
| --- | --- | --- | --- | --- | --- | --- | --- | --- |
| Rv3898c | Rv3898c | 2 | 24 | 5 | 37 | 0.7107 | -0.0421 | 0.6167 |
| Rv2984 | ppk | 10 | 16 | 18 | 24 | 0.6398 | -0.0440 | 0.8333 |
| Rv0540 | Rv0540 | 5 | 21 | 10 | 32 | 0.6709 | -0.0458 | 0.7619 |
| Rv3411c | guaB2 | 5 | 21 | 10 | 32 | 0.6709 | -0.0458 | 0.7619 |
| Rv3863 | Rv3863 | 5 | 21 | 10 | 32 | 0.6709 | -0.0458 | 0.7619 |
| Rv0005 | gyrB | 0 | 26 | 2 | 40 | 0.8706 | -0.0476 | 0.0000 |
| Rv0031 | Rv0031 | 0 | 26 | 2 | 40 | 0.8706 | -0.0476 | 0.0000 |
| Rv0036c | Rv0036c | 0 | 26 | 2 | 40 | 0.8706 | -0.0476 | 0.0000 |
| Rv0057 | Rv0057 | 0 | 26 | 2 | 40 | 0.8706 | -0.0476 | 0.0000 |
| Rv0073 | Rv0073 | 0 | 26 | 2 | 40 | 0.8706 | -0.0476 | 0.0000 |
| Rv0093c | Rv0093c | 0 | 26 | 2 | 40 | 0.8706 | -0.0476 | 0.0000 |
| Rv0102 | Rv0102 | 0 | 26 | 2 | 40 | 0.8706 | -0.0476 | 0.0000 |
| Rv0113 | gmhA | 0 | 26 | 2 | 40 | 0.8706 | -0.0476 | 0.0000 |
| Rv0125 | pepA | 0 | 26 | 2 | 40 | 0.8706 | -0.0476 | 0.0000 |
| Rv0139 | Rv0139 | 0 | 26 | 2 | 40 | 0.8706 | -0.0476 | 0.0000 |
| Rv0157 | pntB | 0 | 26 | 2 | 40 | 0.8706 | -0.0476 | 0.0000 |
| Rv0176 | Rv0176 | 0 | 26 | 2 | 40 | 0.8706 | -0.0476 | 0.0000 |
| Rv0186 | bglS | 0 | 26 | 2 | 40 | 0.8706 | -0.0476 | 0.0000 |
| Rv0253 | nirD | 0 | 26 | 2 | 40 | 0.8706 | -0.0476 | 0.0000 |
| Rv0291 | mycP3 | 0 | 26 | 2 | 40 | 0.8706 | -0.0476 | 0.0000 |
| Rv0303 | Rv0303 | 0 | 26 | 2 | 40 | 0.8706 | -0.0476 | 0.0000 |
| Rv0332 | Rv0332 | 0 | 26 | 2 | 40 | 0.8706 | -0.0476 | 0.0000 |
| Rv0367c | Rv0367c | 0 | 26 | 2 | 40 | 0.8706 | -0.0476 | 0.0000 |
| Rv0374c | Rv0374c | 0 | 26 | 2 | 40 | 0.8706 | -0.0476 | 0.0000 |
| Rv0384c | clpB | 0 | 26 | 2 | 40 | 0.8706 | -0.0476 | 0.0000 |
| Rv0402c | mmpL1 | 0 | 26 | 2 | 40 | 0.8706 | -0.0476 | 0.0000 |
| Rv0424c | Rv0424c | 0 | 26 | 2 | 40 | 0.8706 | -0.0476 | 0.0000 |
| Rv0434 | Rv0434 | 0 | 26 | 2 | 40 | 0.8706 | -0.0476 | 0.0000 |
| Rv0509 | hemA | 0 | 26 | 2 | 40 | 0.8706 | -0.0476 | 0.0000 |
| Rv0535 | pnp | 0 | 26 | 2 | 40 | 0.8706 | -0.0476 | 0.0000 |
| Rv0568 | cyp135B1 | 0 | 26 | 2 | 40 | 0.8706 | -0.0476 | 0.0000 |
| Rv0579 | Rv0579 | 0 | 26 | 2 | 40 | 0.8706 | -0.0476 | 0.0000 |
| Rv0580c | Rv0580c | 0 | 26 | 2 | 40 | 0.8706 | -0.0476 | 0.0000 |

| Rv0628c | Rv0628c | 0 | 26 | 2 | 40 | 0.8706 | -0.0476 | 0.0000 |
| --- | --- | --- | --- | --- | --- | --- | --- | --- |
| Rv0648 | Rv0648 | 0 | 26 | 2 | 40 | 0.8706 | -0.0476 | 0.0000 |
| Rv0653c | Rv0653c | 0 | 26 | 2 | 40 | 0.8706 | -0.0476 | 0.0000 |
| Rv0694 | lldD1 | 0 | 26 | 2 | 40 | 0.8706 | -0.0476 | 0.0000 |
| Rv0737 | Rv0737 | 0 | 26 | 2 | 40 | 0.8706 | -0.0476 | 0.0000 |
| Rv0857 | Rv0857 | 0 | 26 | 2 | 40 | 0.8706 | -0.0476 | 0.0000 |
| Rv0873 | fadE10 | 0 | 26 | 2 | 40 | 0.8706 | -0.0476 | 0.0000 |
| Rv0878c | PPE13 | 0 | 26 | 2 | 40 | 0.8706 | -0.0476 | 0.0000 |
| Rv0897c | Rv0897c | 0 | 26 | 2 | 40 | 0.8706 | -0.0476 | 0.0000 |
| Rv0899 | ompA | 0 | 26 | 2 | 40 | 0.8706 | -0.0476 | 0.0000 |
| Rv0902c | prrB | 0 | 26 | 2 | 40 | 0.8706 | -0.0476 | 0.0000 |
| Rv0903c | prrA | 0 | 26 | 2 | 40 | 0.8706 | -0.0476 | 0.0000 |
| Rv0904c | accD3 | 0 | 26 | 2 | 40 | 0.8706 | -0.0476 | 0.0000 |
| Rv0949 | uvrD1 | 0 | 26 | 2 | 40 | 0.8706 | -0.0476 | 0.0000 |
| Rv1002c | Rv1002c | 0 | 26 | 2 | 40 | 0.8706 | -0.0476 | 0.0000 |
| Rv1004c | Rv1004c | 0 | 26 | 2 | 40 | 0.8706 | -0.0476 | 0.0000 |
| Rv1068c | PE_PGRS20 | 0 | 26 | 2 | 40 | 0.8706 | -0.0476 | 0.0000 |
| Rv1083 | mca | 0 | 26 | 2 | 40 | 0.8706 | -0.0476 | 0.0000 |
| Rv1089 | PE9 | 0 | 26 | 2 | 40 | 0.8706 | -0.0476 | 0.0000 |
| Rv1115 | Rv1115 | 0 | 26 | 2 | 40 | 0.8706 | -0.0476 | 0.0000 |
| Rv1116 | Rv1116 | 0 | 26 | 2 | 40 | 0.8706 | -0.0476 | 0.0000 |
| Rv1171 | Rv1171 | 0 | 26 | 2 | 40 | 0.8706 | -0.0476 | 0.0000 |
| Rv1181 | pks4 | 0 | 26 | 2 | 40 | 0.8706 | -0.0476 | 0.0000 |
| Rv1192 | Rv1192 | 0 | 26 | 2 | 40 | 0.8706 | -0.0476 | 0.0000 |
| Rv1253 | deaD | 0 | 26 | 2 | 40 | 0.8706 | -0.0476 | 0.0000 |
| Rv1295 | thrC | 0 | 26 | 2 | 40 | 0.8706 | -0.0476 | 0.0000 |
| Rv1297 | rho | 0 | 26 | 2 | 40 | 0.8706 | -0.0476 | 0.0000 |
| Rv1310 | atpD | 0 | 26 | 2 | 40 | 0.8706 | -0.0476 | 0.0000 |
| Rv1376 | Rv1376 | 0 | 26 | 2 | 40 | 0.8706 | -0.0476 | 0.0000 |
| Rv1393c | Rv1393c | 0 | 26 | 2 | 40 | 0.8706 | -0.0476 | 0.0000 |
| Rv1489A | Rv1489A | 0 | 26 | 2 | 40 | 0.8706 | -0.0476 | 0.0000 |
| Rv1575 | Rv1575 | 0 | 26 | 2 | 40 | 0.8706 | -0.0476 | 0.0000 |
| Rv1609 | trpE | 0 | 26 | 2 | 40 | 0.8706 | -0.0476 | 0.0000 |
| Rv1610 | Rv1610 | 0 | 26 | 2 | 40 | 0.8706 | -0.0476 | 0.0000 |

| Rv1683 | Rv1683 | 0 | 26 | 2 | 40 | 0.8706 | -0.0476 | 0.0000 |
| --- | --- | --- | --- | --- | --- | --- | --- | --- |
| Rv1754c | Rv1754c | 0 | 26 | 2 | 40 | 0.8706 | -0.0476 | 0.0000 |
| Rv1776c | Rv1776c | 0 | 26 | 2 | 40 | 0.8706 | -0.0476 | 0.0000 |
| Rv1797 | Rv1797 | 0 | 26 | 2 | 40 | 0.8706 | -0.0476 | 0.0000 |
| Rv1810 | Rv1810 | 0 | 26 | 2 | 40 | 0.8706 | -0.0476 | 0.0000 |
| Rv1831 | Rv1831 | 0 | 26 | 2 | 40 | 0.8706 | -0.0476 | 0.0000 |
| Rv1832 | gcvB | 0 | 26 | 2 | 40 | 0.8706 | -0.0476 | 0.0000 |
| Rv1840c | PE_PGRS34 | 0 | 26 | 2 | 40 | 0.8706 | -0.0476 | 0.0000 |
| Rv1859 | modC | 0 | 26 | 2 | 40 | 0.8706 | -0.0476 | 0.0000 |
| Rv1880c | cyp140 | 0 | 26 | 2 | 40 | 0.8706 | -0.0476 | 0.0000 |
| Rv1887 | Rv1887 | 0 | 26 | 2 | 40 | 0.8706 | -0.0476 | 0.0000 |
| Rv1894c | Rv1894c | 0 | 26 | 2 | 40 | 0.8706 | -0.0476 | 0.0000 |
| Rv1907c | Rv1907c | 0 | 26 | 2 | 40 | 0.8706 | -0.0476 | 0.0000 |
| Rv1952 | Rv1952 | 0 | 26 | 2 | 40 | 0.8706 | -0.0476 | 0.0000 |
| Rv1958c | Rv1958c | 0 | 26 | 2 | 40 | 0.8706 | -0.0476 | 0.0000 |
| Rv2001 | Rv2001 | 0 | 26 | 2 | 40 | 0.8706 | -0.0476 | 0.0000 |
| Rv2004c | Rv2004c | 0 | 26 | 2 | 40 | 0.8706 | -0.0476 | 0.0000 |
| Rv2013 | Rv2013 | 0 | 26 | 2 | 40 | 0.8706 | -0.0476 | 0.0000 |
| Rv2035 | Rv2035 | 0 | 26 | 2 | 40 | 0.8706 | -0.0476 | 0.0000 |
| Rv2113 | Rv2113 | 0 | 26 | 2 | 40 | 0.8706 | -0.0476 | 0.0000 |
| Rv2119 | Rv2119 | 0 | 26 | 2 | 40 | 0.8706 | -0.0476 | 0.0000 |
| Rv2152c | murC | 0 | 26 | 2 | 40 | 0.8706 | -0.0476 | 0.0000 |
| Rv2181 | Rv2181 | 0 | 26 | 2 | 40 | 0.8706 | -0.0476 | 0.0000 |
| Rv2207 | cobT | 0 | 26 | 2 | 40 | 0.8706 | -0.0476 | 0.0000 |
| Rv2230c | Rv2230c | 0 | 26 | 2 | 40 | 0.8706 | -0.0476 | 0.0000 |
| Rv2298 | Rv2298 | 0 | 26 | 2 | 40 | 0.8706 | -0.0476 | 0.0000 |
| Rv2305 | Rv2305 | 0 | 26 | 2 | 40 | 0.8706 | -0.0476 | 0.0000 |
| Rv2340c | PE_PGRS39 | 0 | 26 | 2 | 40 | 0.8706 | -0.0476 | 0.0000 |
| Rv2343c | dnaG | 0 | 26 | 2 | 40 | 0.8706 | -0.0476 | 0.0000 |
| Rv2348c | Rv2348c | 0 | 26 | 2 | 40 | 0.8706 | -0.0476 | 0.0000 |
| Rv2367c | Rv2367c | 0 | 26 | 2 | 40 | 0.8706 | -0.0476 | 0.0000 |
| Rv2445c | ndk | 0 | 26 | 2 | 40 | 0.8706 | -0.0476 | 0.0000 |
| Rv2460c | clpP2 | 0 | 26 | 2 | 40 | 0.8706 | -0.0476 | 0.0000 |
| Rv2501c | accA1 | 0 | 26 | 2 | 40 | 0.8706 | -0.0476 | 0.0000 |

| Rv2514c | Rv2514c | 0 | 26 | 2 | 40 | 0.8706 | -0.0476 | 0.0000 |
| --- | --- | --- | --- | --- | --- | --- | --- | --- |
| Rv2522c | Rv2522c | 0 | 26 | 2 | 40 | 0.8706 | -0.0476 | 0.0000 |
| Rv2546 | Rv2546 | 0 | 26 | 2 | 40 | 0.8706 | -0.0476 | 0.0000 |
| Rv2575 | Rv2575 | 0 | 26 | 2 | 40 | 0.8706 | -0.0476 | 0.0000 |
| Rv2577 | Rv2577 | 0 | 26 | 2 | 40 | 0.8706 | -0.0476 | 0.0000 |
| Rv2581c | Rv2581c | 0 | 26 | 2 | 40 | 0.8706 | -0.0476 | 0.0000 |
| Rv2583c | relA | 0 | 26 | 2 | 40 | 0.8706 | -0.0476 | 0.0000 |
| Rv2587c | secD | 0 | 26 | 2 | 40 | 0.8706 | -0.0476 | 0.0000 |
| Rv2601A | Rv2601A | 0 | 26 | 2 | 40 | 0.8706 | -0.0476 | 0.0000 |
| Rv2608 | PPE42 | 0 | 26 | 2 | 40 | 0.8706 | -0.0476 | 0.0000 |
| Rv2614c | thrS | 0 | 26 | 2 | 40 | 0.8706 | -0.0476 | 0.0000 |
| Rv2647 | Rv2647 | 0 | 26 | 2 | 40 | 0.8706 | -0.0476 | 0.0000 |
| Rv2681 | Rv2681 | 0 | 26 | 2 | 40 | 0.8706 | -0.0476 | 0.0000 |
| Rv2703 | sigA | 0 | 26 | 2 | 40 | 0.8706 | -0.0476 | 0.0000 |
| Rv2720 | lexA | 0 | 26 | 2 | 40 | 0.8706 | -0.0476 | 0.0000 |
| Rv2735c | Rv2735c | 0 | 26 | 2 | 40 | 0.8706 | -0.0476 | 0.0000 |
| Rv2752c | Rv2752c | 0 | 26 | 2 | 40 | 0.8706 | -0.0476 | 0.0000 |
| Rv2780 | ald | 0 | 26 | 2 | 40 | 0.8706 | -0.0476 | 0.0000 |
| Rv2834c | ugpE | 0 | 26 | 2 | 40 | 0.8706 | -0.0476 | 0.0000 |
| Rv2860c | glnA4 | 0 | 26 | 2 | 40 | 0.8706 | -0.0476 | 0.0000 |
| Rv2892c | Rv2891 | 0 | 26 | 2 | 40 | 0.8706 | -0.0476 | 0.0000 |
| Rv2913c | Rv2913c | 0 | 26 | 2 | 40 | 0.8706 | -0.0476 | 0.0000 |
| Rv2915c | Rv2915c | 0 | 26 | 2 | 40 | 0.8706 | -0.0476 | 0.0000 |
| Rv2920c | amt | 0 | 26 | 2 | 40 | 0.8706 | -0.0476 | 0.0000 |
| Rv2921c | ftsY | 0 | 26 | 2 | 40 | 0.8706 | -0.0476 | 0.0000 |
| Rv2950c | fadD29 | 0 | 26 | 2 | 40 | 0.8706 | -0.0476 | 0.0000 |
| Rv2991 | Rv2991 | 0 | 26 | 2 | 40 | 0.8706 | -0.0476 | 0.0000 |
| Rv2992c | gltX | 0 | 26 | 2 | 40 | 0.8706 | -0.0476 | 0.0000 |
| Rv3014c | ligA | 0 | 26 | 2 | 40 | 0.8706 | -0.0476 | 0.0000 |
| Rv3032 | Rv3032 | 0 | 26 | 2 | 40 | 0.8706 | -0.0476 | 0.0000 |
| Rv3040c | Rv3040c | 0 | 26 | 2 | 40 | 0.8706 | -0.0476 | 0.0000 |
| Rv3075c | Rv3075c | 0 | 26 | 2 | 40 | 0.8706 | -0.0476 | 0.0000 |
| Rv3079c | Rv3079c | 0 | 26 | 2 | 40 | 0.8706 | -0.0476 | 0.0000 |
| Rv3099c | Rv3099c | 0 | 26 | 2 | 40 | 0.8706 | -0.0476 | 0.0000 |

| Rv3122 | Rv3122 | 0 | 26 | 2 | 40 | 0.8706 | -0.0476 | 0.0000 |
| --- | --- | --- | --- | --- | --- | --- | --- | --- |
| Rv3146 | nuoB | 0 | 26 | 2 | 40 | 0.8706 | -0.0476 | 0.0000 |
| Rv3191c | Rv3191c | 0 | 26 | 2 | 40 | 0.8706 | -0.0476 | 0.0000 |
| Rv3194c | Rv3194c | 0 | 26 | 2 | 40 | 0.8706 | -0.0476 | 0.0000 |
| Rv3233c | Rv3233c | 0 | 26 | 2 | 40 | 0.8706 | -0.0476 | 0.0000 |
| Rv3241c | Rv3241c | 0 | 26 | 2 | 40 | 0.8706 | -0.0476 | 0.0000 |
| Rv3277 | Rv3277 | 0 | 26 | 2 | 40 | 0.8706 | -0.0476 | 0.0000 |
| Rv3312A | Rv3312A | 0 | 26 | 2 | 40 | 0.8706 | -0.0476 | 0.0000 |
| Rv3314c | deoA | 0 | 26 | 2 | 40 | 0.8706 | -0.0476 | 0.0000 |
| Rv3526 | Rv3526 | 0 | 26 | 2 | 40 | 0.8706 | -0.0476 | 0.0000 |
| Rv3541c | Rv3541c | 0 | 26 | 2 | 40 | 0.8706 | -0.0476 | 0.0000 |
| Rv3565 | aspB | 0 | 26 | 2 | 40 | 0.8706 | -0.0476 | 0.0000 |
| Rv3625c | mesJ | 0 | 26 | 2 | 40 | 0.8706 | -0.0476 | 0.0000 |
| Rv3684 | Rv3684 | 0 | 26 | 2 | 40 | 0.8706 | -0.0476 | 0.0000 |
| Rv3787c | Rv3787c | 0 | 26 | 2 | 40 | 0.8706 | -0.0476 | 0.0000 |
| Rv3807c | Rv3807c | 0 | 26 | 2 | 40 | 0.8706 | -0.0476 | 0.0000 |
| Rv3918c | parA | 0 | 26 | 2 | 40 | 0.8706 | -0.0476 | 0.0000 |
| Rv0368c | Rv0368c | 8 | 18 | 15 | 27 | 0.6623 | -0.0495 | 0.8000 |
| Rv0995 | rimJ | 8 | 18 | 15 | 27 | 0.6623 | -0.0495 | 0.8000 |
| Rv1290c | Rv1290c | 8 | 18 | 15 | 27 | 0.6623 | -0.0495 | 0.8000 |
| Rv1361c | PPE19 | 8 | 18 | 15 | 27 | 0.6623 | -0.0495 | 0.8000 |
| Rv1538c | ansA | 8 | 18 | 15 | 27 | 0.6623 | -0.0495 | 0.8000 |
| Rv1821 | secA2 | 8 | 18 | 15 | 27 | 0.6623 | -0.0495 | 0.8000 |
| Rv3515c | fadD19 | 8 | 18 | 15 | 27 | 0.6623 | -0.0495 | 0.8000 |
| Rv3523 | ltp3 | 8 | 18 | 15 | 27 | 0.6623 | -0.0495 | 0.8000 |
| Rv0118c | oxcA | 3 | 23 | 7 | 35 | 0.7191 | -0.0513 | 0.6522 |
| Rv0206c | mmpL3 | 3 | 23 | 7 | 35 | 0.7191 | -0.0513 | 0.6522 |
| Rv0304c | PPE5 | 3 | 23 | 7 | 35 | 0.7191 | -0.0513 | 0.6522 |
| Rv1044 | Rv1044 | 3 | 23 | 7 | 35 | 0.7191 | -0.0513 | 0.6522 |
| Rv1067c | PE_PGRS19 | 3 | 23 | 7 | 35 | 0.7191 | -0.0513 | 0.6522 |
| Rv1163 | narJ | 3 | 23 | 7 | 35 | 0.7191 | -0.0513 | 0.6522 |
| Rv1205 | Rv1205 | 3 | 23 | 7 | 35 | 0.7191 | -0.0513 | 0.6522 |
| Rv2077c | Rv2077c | 3 | 23 | 7 | 35 | 0.7191 | -0.0513 | 0.6522 |
| Rv2103c | Rv2103c | 3 | 23 | 7 | 35 | 0.7191 | -0.0513 | 0.6522 |

| Rv2839c | infB | 3 | 23 | 7 | 35 | 0.7191 | -0.0513 | 0.6522 |
| --- | --- | --- | --- | --- | --- | --- | --- | --- |
| Rv3870 | Rv3870 | 3 | 23 | 7 | 35 | 0.7191 | -0.0513 | 0.6522 |
| Rv1358 | Rv1358 | 6 | 20 | 12 | 30 | 0.6911 | -0.0549 | 0.7500 |
| Rv3037c | Rv3037c | 6 | 20 | 12 | 30 | 0.6911 | -0.0549 | 0.7500 |
| Rv0823c | Rv0823c | 1 | 25 | 4 | 38 | 0.8083 | -0.0568 | 0.3800 |
| Rv1264 | Rv1264 | 1 | 25 | 4 | 38 | 0.8083 | -0.0568 | 0.3800 |
| Rv1281c | oppD | 1 | 25 | 4 | 38 | 0.8083 | -0.0568 | 0.3800 |
| Rv1289 | Rv1289 | 1 | 25 | 4 | 38 | 0.8083 | -0.0568 | 0.3800 |
| Rv1372 | Rv1372 | 1 | 25 | 4 | 38 | 0.8083 | -0.0568 | 0.3800 |
| Rv1468c | PE_PGRS29 | 1 | 25 | 4 | 38 | 0.8083 | -0.0568 | 0.3800 |
| Rv1695 | ppnK | 1 | 25 | 4 | 38 | 0.8083 | -0.0568 | 0.3800 |
| Rv1768 | PE_PGRS31 | 1 | 25 | 4 | 38 | 0.8083 | -0.0568 | 0.3800 |
| Rv1914c | Rv1914c | 1 | 25 | 4 | 38 | 0.8083 | -0.0568 | 0.3800 |
| Rv2338c | moeW | 1 | 25 | 4 | 38 | 0.8083 | -0.0568 | 0.3800 |
| Rv2342 | Rv2342 | 1 | 25 | 4 | 38 | 0.8083 | -0.0568 | 0.3800 |
| Rv2652c | Rv2652c | 1 | 25 | 4 | 38 | 0.8083 | -0.0568 | 0.3800 |
| Rv2666 | Rv2666 | 1 | 25 | 4 | 38 | 0.8083 | -0.0568 | 0.3800 |
| Rv3225c | Rv3225c | 1 | 25 | 4 | 38 | 0.8083 | -0.0568 | 0.3800 |
| Rv3349c | Rv3349c | 1 | 25 | 4 | 38 | 0.8083 | -0.0568 | 0.3800 |
| Rv3550 | echA20 | 1 | 25 | 4 | 38 | 0.8083 | -0.0568 | 0.3800 |
| Rv3651 | Rv3651 | 1 | 25 | 4 | 38 | 0.8083 | -0.0568 | 0.3800 |
| Rv3831 | Rv3831 | 1 | 25 | 4 | 38 | 0.8083 | -0.0568 | 0.3800 |
| Rv3887c | Rv3887c | 1 | 25 | 4 | 38 | 0.8083 | -0.0568 | 0.3800 |
| Rv0048c | Rv0048c | 9 | 17 | 17 | 25 | 0.6856 | -0.0586 | 0.7785 |
| Rv0380c | Rv0380c | 9 | 17 | 17 | 25 | 0.6856 | -0.0586 | 0.7785 |
| Rv0588 | yrbE2B | 9 | 17 | 17 | 25 | 0.6856 | -0.0586 | 0.7785 |
| Rv1497 | lipL | 9 | 17 | 17 | 25 | 0.6856 | -0.0586 | 0.7785 |
| Rv3729 | Rv3729 | 9 | 17 | 17 | 25 | 0.6856 | -0.0586 | 0.7785 |
| Rv3759c | proX | 9 | 17 | 17 | 25 | 0.6856 | -0.0586 | 0.7785 |
| Rv3327 | Rv3327 | 22 | 4 | 38 | 4 | 0.7670 | -0.0586 | 0.5789 |
| Rv0170 | mce1B | 4 | 22 | 9 | 33 | 0.7310 | -0.0604 | 0.6667 |
| Rv2940c | mas | 4 | 22 | 9 | 33 | 0.7310 | -0.0604 | 0.6667 |
| Rv3124 | Rv3124 | 4 | 22 | 9 | 33 | 0.7310 | -0.0604 | 0.6667 |
| Rv3343c | PPE54 | 4 | 22 | 9 | 33 | 0.7310 | -0.0604 | 0.6667 |

| Rv0331 | Rv0331 | 7 | 19 | 14 | 28 | 0.7109 | -0.0641 | 0.7368 |
| --- | --- | --- | --- | --- | --- | --- | --- | --- |
| Rv0969 | ctpV | 7 | 19 | 14 | 28 | 0.7109 | -0.0641 | 0.7368 |
| Rv1523 | Rv1523 | 7 | 19 | 14 | 28 | 0.7109 | -0.0641 | 0.7368 |
| Rv1920 | Rv1920 | 7 | 19 | 14 | 28 | 0.7109 | -0.0641 | 0.7368 |
| Rv2176 | pknL | 7 | 19 | 14 | 28 | 0.7109 | -0.0641 | 0.7368 |
| Rv2621c | Rv2621c | 7 | 19 | 14 | 28 | 0.7109 | -0.0641 | 0.7368 |
| Rv0013 | trpG | 2 | 24 | 6 | 36 | 0.7939 | -0.0659 | 0.5000 |
| Rv0576 | Rv0576 | 2 | 24 | 6 | 36 | 0.7939 | -0.0659 | 0.5000 |
| Rv1142c | echA10 | 2 | 24 | 6 | 36 | 0.7939 | -0.0659 | 0.5000 |
| Rv1193 | fadD36 | 2 | 24 | 6 | 36 | 0.7939 | -0.0659 | 0.5000 |
| Rv1329c | dinG | 2 | 24 | 6 | 36 | 0.7939 | -0.0659 | 0.5000 |
| Rv1835c | Rv1835c | 2 | 24 | 6 | 36 | 0.7939 | -0.0659 | 0.5000 |
| Rv1969 | mce3D | 2 | 24 | 6 | 36 | 0.7939 | -0.0659 | 0.5000 |
| Rv2047c | Rv2047c | 2 | 24 | 6 | 36 | 0.7939 | -0.0659 | 0.5000 |
| Rv2346c | esxO | 2 | 24 | 6 | 36 | 0.7939 | -0.0659 | 0.5000 |
| Rv2492 | Rv2492 | 2 | 24 | 6 | 36 | 0.7939 | -0.0659 | 0.5000 |
| Rv2979c | Rv2979c | 2 | 24 | 6 | 36 | 0.7939 | -0.0659 | 0.5000 |
| Rv2998 | Rv2998 | 2 | 24 | 6 | 36 | 0.7939 | -0.0659 | 0.5000 |
| Rv3081 | Rv3081 | 2 | 24 | 6 | 36 | 0.7939 | -0.0659 | 0.5000 |
| Rv3087 | Rv3087 | 2 | 24 | 6 | 36 | 0.7939 | -0.0659 | 0.5000 |
| Rv3445c | esxU | 2 | 24 | 6 | 36 | 0.7939 | -0.0659 | 0.5000 |
| Rv3618 | Rv3618 | 2 | 24 | 6 | 36 | 0.7939 | -0.0659 | 0.5000 |
| Rv3620c | esxW | 2 | 24 | 6 | 36 | 0.7939 | -0.0659 | 0.5000 |
| Rv3680 | Rv3680 | 2 | 24 | 6 | 36 | 0.7939 | -0.0659 | 0.5000 |
| Rv3691 | Rv3691 | 2 | 24 | 6 | 36 | 0.7939 | -0.0659 | 0.5000 |
| Rv3714c | Rv3714c | 2 | 24 | 6 | 36 | 0.7939 | -0.0659 | 0.5000 |
| Rv3800c | pks13 | 2 | 24 | 6 | 36 | 0.7939 | -0.0659 | 0.5000 |
| Rv3903c | Rv3903c | 2 | 24 | 6 | 36 | 0.7939 | -0.0659 | 0.5000 |
| Rv0376c | Rv0376c | 10 | 16 | 19 | 23 | 0.7085 | -0.0678 | 0.7566 |
| Rv3467 | Rv3467 | 23 | 3 | 40 | 2 | 0.8509 | -0.0678 | 0.3833 |
| Rv0094c | Rv0094c | 5 | 21 | 11 | 31 | 0.7446 | -0.0696 | 0.6710 |
| Rv2443 | dctA | 5 | 21 | 11 | 31 | 0.7446 | -0.0696 | 0.6710 |
| Rv3825c | pks2 | 5 | 21 | 11 | 31 | 0.7446 | -0.0696 | 0.6710 |
| Rv0020c | TB39.8 | 0 | 26 | 3 | 39 | 0.9183 | -0.0714 | 0.0000 |

| Rv0140 | Rv0140 | 0 | 26 | 3 | 39 | 0.9183 | -0.0714 | 0.0000 |
| --- | --- | --- | --- | --- | --- | --- | --- | --- |
| Rv0305c | PPE6 | 0 | 26 | 3 | 39 | 0.9183 | -0.0714 | 0.0000 |
| Rv0471c | Rv0471c | 0 | 26 | 3 | 39 | 0.9183 | -0.0714 | 0.0000 |
| Rv0570 | nrdZ | 0 | 26 | 3 | 39 | 0.9183 | -0.0714 | 0.0000 |
| Rv0713 | Rv0713 | 0 | 26 | 3 | 39 | 0.9183 | -0.0714 | 0.0000 |
| Rv0729 | xylB | 0 | 26 | 3 | 39 | 0.9183 | -0.0714 | 0.0000 |
| Rv0731c | Rv0731c | 0 | 26 | 3 | 39 | 0.9183 | -0.0714 | 0.0000 |
| Rv0846c | Rv0846c | 0 | 26 | 3 | 39 | 0.9183 | -0.0714 | 0.0000 |
| Rv0915c | PPE14 | 0 | 26 | 3 | 39 | 0.9183 | -0.0714 | 0.0000 |
| Rv1098c | fumC | 0 | 26 | 3 | 39 | 0.9183 | -0.0714 | 0.0000 |
| Rv1122 | gnd2 | 0 | 26 | 3 | 39 | 0.9183 | -0.0714 | 0.0000 |
| Rv1200 | Rv1200 | 0 | 26 | 3 | 39 | 0.9183 | -0.0714 | 0.0000 |
| Rv1215c | Rv1215c | 0 | 26 | 3 | 39 | 0.9183 | -0.0714 | 0.0000 |
| Rv1244 | lpqZ | 0 | 26 | 3 | 39 | 0.9183 | -0.0714 | 0.0000 |
| Rv1277 | Rv1277 | 0 | 26 | 3 | 39 | 0.9183 | -0.0714 | 0.0000 |
| Rv1425 | Rv1425 | 0 | 26 | 3 | 39 | 0.9183 | -0.0714 | 0.0000 |
| Rv1638 | uvrA | 0 | 26 | 3 | 39 | 0.9183 | -0.0714 | 0.0000 |
| Rv1663 | pks17 | 0 | 26 | 3 | 39 | 0.9183 | -0.0714 | 0.0000 |
| Rv1672c | Rv1672c | 0 | 26 | 3 | 39 | 0.9183 | -0.0714 | 0.0000 |
| Rv2041c | Rv2041c | 0 | 26 | 3 | 39 | 0.9183 | -0.0714 | 0.0000 |
| Rv2151c | ftsQ | 0 | 26 | 3 | 39 | 0.9183 | -0.0714 | 0.0000 |
| Rv2217 | lipB | 0 | 26 | 3 | 39 | 0.9183 | -0.0714 | 0.0000 |
| Rv2234 | ptpA | 0 | 26 | 3 | 39 | 0.9183 | -0.0714 | 0.0000 |
| Rv2310 | Rv2310 | 0 | 26 | 3 | 39 | 0.9183 | -0.0714 | 0.0000 |
| Rv2435c | Rv2435c | 0 | 26 | 3 | 39 | 0.9183 | -0.0714 | 0.0000 |
| Rv2444c | rne | 0 | 26 | 3 | 39 | 0.9183 | -0.0714 | 0.0000 |
| Rv2628 | Rv2628 | 0 | 26 | 3 | 39 | 0.9183 | -0.0714 | 0.0000 |
| Rv2926c | Rv2926c | 0 | 26 | 3 | 39 | 0.9183 | -0.0714 | 0.0000 |
| Rv3071 | Rv3071 | 0 | 26 | 3 | 39 | 0.9183 | -0.0714 | 0.0000 |
| Rv3150 | nuoF | 0 | 26 | 3 | 39 | 0.9183 | -0.0714 | 0.0000 |
| Rv3305c | amiA1 | 0 | 26 | 3 | 39 | 0.9183 | -0.0714 | 0.0000 |
| Rv3505 | fadE27 | 0 | 26 | 3 | 39 | 0.9183 | -0.0714 | 0.0000 |
| Rv3596c | clpC1 | 0 | 26 | 3 | 39 | 0.9183 | -0.0714 | 0.0000 |
| Rv3736 | Rv3736 | 0 | 26 | 3 | 39 | 0.9183 | -0.0714 | 0.0000 |

| Rv3784 | Rv3784 | 0 | 26 | 3 | 39 | 0.9183 | -0.0714 | 0.0000 |
| --- | --- | --- | --- | --- | --- | --- | --- | --- |
| Rv3791 | Rv3791 | 0 | 26 | 3 | 39 | 0.9183 | -0.0714 | 0.0000 |
| Rv3794 | embA | 0 | 26 | 3 | 39 | 0.9183 | -0.0714 | 0.0000 |
| Rv0732 | secY | 3 | 23 | 8 | 34 | 0.7931 | -0.0751 | 0.5543 |
| Rv1317c | alkA | 3 | 23 | 8 | 34 | 0.7931 | -0.0751 | 0.5543 |
| Rv1363c | Rv1363c | 3 | 23 | 8 | 34 | 0.7931 | -0.0751 | 0.5543 |
| Rv1463 | Rv1463 | 3 | 23 | 8 | 34 | 0.7931 | -0.0751 | 0.5543 |
| Rv2537c | aroD | 3 | 23 | 8 | 34 | 0.7931 | -0.0751 | 0.5543 |
| Rv3299c | atsB | 3 | 23 | 8 | 34 | 0.7931 | -0.0751 | 0.5543 |
| Rv3666c | dppA | 3 | 23 | 8 | 34 | 0.7931 | -0.0751 | 0.5543 |
| Rv3820c | papA2 | 3 | 23 | 8 | 34 | 0.7931 | -0.0751 | 0.5543 |
| Rv0151c | PE1 | 11 | 15 | 21 | 21 | 0.7316 | -0.0769 | 0.7333 |
| Rv1917c | PPE34 | 24 | 2 | 42 | 0 | 0.9660 | -0.0769 | 0.0000 |
| Rv2037c | Rv2037c | 24 | 2 | 42 | 0 | 0.9660 | -0.0769 | 0.0000 |
| Rv2039c | Rv2039c | 24 | 2 | 42 | 0 | 0.9660 | -0.0769 | 0.0000 |
| Rv0158 | Rv0158 | 6 | 20 | 13 | 29 | 0.7591 | -0.0788 | 0.6692 |
| Rv1194c | Rv1194c | 6 | 20 | 13 | 29 | 0.7591 | -0.0788 | 0.6692 |
| Rv2473 | Rv2473 | 6 | 20 | 13 | 29 | 0.7591 | -0.0788 | 0.6692 |
| Rv0205 | Rv0205 | 1 | 25 | 5 | 37 | 0.8726 | -0.0806 | 0.2960 |
| Rv0721 | rpsE | 1 | 25 | 5 | 37 | 0.8726 | -0.0806 | 0.2960 |
| Rv0746 | PE_PGRS9 | 1 | 25 | 5 | 37 | 0.8726 | -0.0806 | 0.2960 |
| Rv0976c | Rv0976c | 1 | 25 | 5 | 37 | 0.8726 | -0.0806 | 0.2960 |
| Rv0986 | Rv0986 | 1 | 25 | 5 | 37 | 0.8726 | -0.0806 | 0.2960 |
| Rv1417 | Rv1417 | 1 | 25 | 5 | 37 | 0.8726 | -0.0806 | 0.2960 |
| Rv1599 | hisD | 1 | 25 | 5 | 37 | 0.8726 | -0.0806 | 0.2960 |
| Rv1614 | lgt | 1 | 25 | 5 | 37 | 0.8726 | -0.0806 | 0.2960 |
| Rv1633 | uvrB | 1 | 25 | 5 | 37 | 0.8726 | -0.0806 | 0.2960 |
| Rv1843c | guaB1 | 1 | 25 | 5 | 37 | 0.8726 | -0.0806 | 0.2960 |
| Rv1870c | Rv1870c | 1 | 25 | 5 | 37 | 0.8726 | -0.0806 | 0.2960 |
| Rv1921c | lppF | 1 | 25 | 5 | 37 | 0.8726 | -0.0806 | 0.2960 |
| Rv2242 | Rv2242 | 1 | 25 | 5 | 37 | 0.8726 | -0.0806 | 0.2960 |
| Rv2265 | Rv2265 | 1 | 25 | 5 | 37 | 0.8726 | -0.0806 | 0.2960 |
| Rv2637 | dedA | 1 | 25 | 5 | 37 | 0.8726 | -0.0806 | 0.2960 |
| Rv2690c | Rv2690c | 1 | 25 | 5 | 37 | 0.8726 | -0.0806 | 0.2960 |

| Rv2799 | Rv2799 | 1 | 25 | 5 | 37 | 0.8726 | -0.0806 | 0.2960 |
| --- | --- | --- | --- | --- | --- | --- | --- | --- |
| Rv2886c | Rv2886c | 1 | 25 | 5 | 37 | 0.8726 | -0.0806 | 0.2960 |
| Rv2924c | fpg | 1 | 25 | 5 | 37 | 0.8726 | -0.0806 | 0.2960 |
| Rv3085 | Rv3085 | 1 | 25 | 5 | 37 | 0.8726 | -0.0806 | 0.2960 |
| Rv3130c | tgs1 | 1 | 25 | 5 | 37 | 0.8726 | -0.0806 | 0.2960 |
| Rv3224A | Rv3224A | 1 | 25 | 5 | 37 | 0.8726 | -0.0806 | 0.2960 |
| Rv3302c | glpD2 | 1 | 25 | 5 | 37 | 0.8726 | -0.0806 | 0.2960 |
| Rv3359 | Rv3359 | 1 | 25 | 5 | 37 | 0.8726 | -0.0806 | 0.2960 |
| Rv3375 | amiD | 1 | 25 | 5 | 37 | 0.8726 | -0.0806 | 0.2960 |
| Rv3698 | Rv3698 | 1 | 25 | 5 | 37 | 0.8726 | -0.0806 | 0.2960 |
| Rv3705A | Rv3705A | 1 | 25 | 5 | 37 | 0.8726 | -0.0806 | 0.2960 |
| Rv4008 | Rv4008 | 1 | 25 | 5 | 37 | 0.8726 | -0.0806 | 0.2960 |
| Rv0046c | ino1 | 9 | 17 | 18 | 24 | 0.7502 | -0.0824 | 0.7059 |
| Rv1527c | pks5 | 9 | 17 | 18 | 24 | 0.7502 | -0.0824 | 0.7059 |
| Rv3466 | Rv3466 | 9 | 17 | 18 | 24 | 0.7502 | -0.0824 | 0.7059 |
| Rv2800 | Rv2800 | 7 | 19 | 15 | 27 | 0.7743 | -0.0879 | 0.6632 |
| Rv3195 | Rv3195 | 7 | 19 | 15 | 27 | 0.7743 | -0.0879 | 0.6632 |
| Rv0386 | Rv0386 | 2 | 24 | 7 | 35 | 0.8557 | -0.0897 | 0.4167 |
| Rv0457c | Rv0457c | 2 | 24 | 7 | 35 | 0.8557 | -0.0897 | 0.4167 |
| Rv0465c | Rv0465c | 2 | 24 | 7 | 35 | 0.8557 | -0.0897 | 0.4167 |
| Rv1622c | cydB | 2 | 24 | 7 | 35 | 0.8557 | -0.0897 | 0.4167 |
| Rv3209 | Rv3209 | 2 | 24 | 7 | 35 | 0.8557 | -0.0897 | 0.4167 |
| Rv3424c | Rv3424c | 2 | 24 | 7 | 35 | 0.8557 | -0.0897 | 0.4167 |
| Rv3793 | embC | 10 | 16 | 20 | 22 | 0.7701 | -0.0916 | 0.6875 |
| Rv0945 | Rv0945 | 5 | 21 | 12 | 30 | 0.8063 | -0.0934 | 0.5952 |
| Rv1226c | Rv1226c | 5 | 21 | 12 | 30 | 0.8063 | -0.0934 | 0.5952 |
| Rv2714 | Rv2714 | 5 | 21 | 12 | 30 | 0.8063 | -0.0934 | 0.5952 |
| Rv3350c | PPE56 | 5 | 21 | 12 | 30 | 0.8063 | -0.0934 | 0.5952 |
| Rv0931c | pknD | 0 | 26 | 4 | 38 | 0.9476 | -0.0952 | 0.0000 |
| Rv0971c | echA7 | 0 | 26 | 4 | 38 | 0.9476 | -0.0952 | 0.0000 |
| Rv1333 | Rv1333 | 0 | 26 | 4 | 38 | 0.9476 | -0.0952 | 0.0000 |
| Rv1559 | ilvA | 0 | 26 | 4 | 38 | 0.9476 | -0.0952 | 0.0000 |
| Rv1844c | gnd1 | 0 | 26 | 4 | 38 | 0.9476 | -0.0952 | 0.0000 |
| Rv1935c | echA13 | 0 | 26 | 4 | 38 | 0.9476 | -0.0952 | 0.0000 |

| Rv2042c | Rv2042c | 0 | 26 | 4 | 38 | 0.9476 | -0.0952 | 0.0000 |
| --- | --- | --- | --- | --- | --- | --- | --- | --- |
| Rv2643 | arsC | 0 | 26 | 4 | 38 | 0.9476 | -0.0952 | 0.0000 |
| Rv2918c | glnD | 0 | 26 | 4 | 38 | 0.9476 | -0.0952 | 0.0000 |
| Rv3388 | PE_PGRS52 | 0 | 26 | 4 | 38 | 0.9476 | -0.0952 | 0.0000 |
| Rv3639c | Rv3639c | 0 | 26 | 4 | 38 | 0.9476 | -0.0952 | 0.0000 |
| Rv3749c | Rv3749c | 0 | 26 | 4 | 38 | 0.9476 | -0.0952 | 0.0000 |
| Rv3906c | Rv3906c | 0 | 26 | 4 | 38 | 0.9476 | -0.0952 | 0.0000 |
| Rv1431 | Rv1431 | 3 | 23 | 9 | 33 | 0.8507 | -0.0989 | 0.4783 |
| Rv2264c | Rv2264c | 3 | 23 | 9 | 33 | 0.8507 | -0.0989 | 0.4783 |
| Rv2424c | Rv2424c | 3 | 23 | 9 | 33 | 0.8507 | -0.0989 | 0.4783 |
| Rv3395c | Rv3395c | 3 | 23 | 9 | 33 | 0.8507 | -0.0989 | 0.4783 |
| Rv3531c | Rv3531c | 3 | 23 | 9 | 33 | 0.8507 | -0.0989 | 0.4783 |
| Rv3591c | Rv3591c | 3 | 23 | 9 | 33 | 0.8507 | -0.0989 | 0.4783 |
| Rv0001 | dnaA | 1 | 25 | 6 | 36 | 0.9157 | -0.1044 | 0.2400 |
| Rv0336 | Rv0336 | 1 | 25 | 6 | 36 | 0.9157 | -0.1044 | 0.2400 |
| Rv0882 | Rv0882 | 1 | 25 | 6 | 36 | 0.9157 | -0.1044 | 0.2400 |
| Rv1983 | PE_PGRS35 | 1 | 25 | 6 | 36 | 0.9157 | -0.1044 | 0.2400 |
| Rv2221c | glnE | 1 | 25 | 6 | 36 | 0.9157 | -0.1044 | 0.2400 |
| Rv2832c | ugpC | 1 | 25 | 6 | 36 | 0.9157 | -0.1044 | 0.2400 |
| Rv3705c | Rv3705c | 1 | 25 | 6 | 36 | 0.9157 | -0.1044 | 0.2400 |
| Rv3881c | Rv3881c | 1 | 25 | 6 | 36 | 0.9157 | -0.1044 | 0.2400 |
| Rv0041 | leuS | 2 | 24 | 8 | 34 | 0.9006 | -0.1136 | 0.3542 |
| Rv0594 | mce2F | 2 | 24 | 8 | 34 | 0.9006 | -0.1136 | 0.3542 |
| Rv1091 | PE_PGRS22 | 0 | 26 | 5 | 37 | 0.9662 | -0.1190 | 0.0000 |
| Rv2006 | otsB1 | 0 | 26 | 5 | 37 | 0.9662 | -0.1190 | 0.0000 |
| Rv0453 | PPE11 | 8 | 18 | 18 | 24 | 0.8406 | -0.1209 | 0.5926 |
| Rv0101 | nrp | 3 | 23 | 10 | 32 | 0.8944 | -0.1227 | 0.4174 |
| Rv2328 | PE23 | 3 | 23 | 10 | 32 | 0.8944 | -0.1227 | 0.4174 |
| Rv2524c | fas | 3 | 23 | 10 | 32 | 0.8944 | -0.1227 | 0.4174 |
| Rv3689 | Rv3689 | 3 | 23 | 10 | 32 | 0.8944 | -0.1227 | 0.4174 |
| Rv1933c | fadE18 | 1 | 25 | 7 | 35 | 0.9446 | -0.1282 | 0.2000 |
| Rv2231c | cobC | 1 | 25 | 7 | 35 | 0.9446 | -0.1282 | 0.2000 |
| Rv0180c | Rv0180c | 0 | 26 | 6 | 36 | 0.9782 | -0.1429 | 0.0000 |
| Rv0620 | galK | 3 | 23 | 11 | 31 | 0.9268 | -0.1465 | 0.3676 |

| Rv3554 | fdxB | 3 | 23 | 11 | 31 | 0.9268 | -0.1465 | 0.3676 |
| --- | --- | --- | --- | --- | --- | --- | --- | --- |
| Rv3428c | Rv3428c | 8 | 18 | 20 | 22 | 0.9150 | -0.1685 | 0.4889 |
| Rv1661 | pks7 | 2 | 24 | 11 | 31 | 0.9703 | -0.1850 | 0.2348 |
| Rv3425 | PPE57 | 3 | 23 | 13 | 29 | 0.9667 | -0.1941 | 0.2910 |
