# Supplementary material for: A multiple genome analysis of Mycobacterium tuberculosis reveals specific novel genes and mutations associated with pyrazinamide resistance
Source: BMC Genomics. 2017 Oct 11;18:769. doi: 10.1186/s12864-017-4146-z (PMC5637355; doi:10.1186/s12864-017-4146-z)
Supplement: Supplementary file 4 — Table of the 110 genes that were significantly associated with PZA-resistance at gene level, which also have mutations significantly associated with PZA-resistance at the mutation level. The association p-value at mutation level was re-calculated excluding the strains harboring mutations strongly related with PZA resistance. These genes were annotated with their molecular function. (DOCX 297 kb) [file 12864_2017_4146_MOESM4_ESM.docx]

Additional file 4: Table S4 (Supplementary Material): Table of the 110 genes that were significantly associated with PZA-resistance at gene level, which also have mutations significantly associated with

PZA-resistance at the mutation level. The association p-value at mutation level was re-calculated excluding the strains harboring mutations strongly related with PZA resistance. These genes were annotated with their molecular function.

|  | | | | Mutation in PZAse |  | | | | |
| --- | --- | --- | --- | --- | --- | --- | --- | --- | --- |
| Synonym  Gene_Name  Mutation pValue_Gene  pValue_Mutation | N_Res_PZA | N_Sus_PZA | Strain  Res_PZA | Gene  Protein  Promoter | PZAse_activity  Mut_Crit_PZAse | Res_PZA_NoCrit_p ncA | NEW_pValue_Muta tion | Function | Location |
| Rv0667 rpoB 450S>L 0 0 | 19 | 5 | SLM100 R  LE486 R TBV5362 R TBDM425 R CSV10399 R CSV5769 R SLM036 R CSV11678 R LN2358 R MDRDM1098 R MDRDM260 R LN3756 R LE492 R ME1473 R MDRDM627 R LN180 R SLM040 R SLM056 R TBV5000 R LEI371M S CSV383 S LN55 S  LE13 S  LN2978 S | - - T-11C  T35G D12A -  - - - C145T D49N - A280G F94L - C232A G78C - A190C Y64D - G185A P62L - A545C L182W - C145T D49N - T403G T135P - T403G T135P - T35G D12A - A100C Y34D -  - - - A392C V131G - T170C H57R -  - - -  - - - | A. Exp.  24.06 *  100  0.12 *  55.18  18.12 * N. A.  N. A. N. A.  0.12 *  0.05 *  0.05 *  24.06 *  53.59  100  N. A.  N. A. *  100  100 | 11 | 0.002 | RNA polymerase | Cytosolic |
| Rv2043c pncA 12D>A 0 0.034 | 2 | 0 | LE492 R  LE486 R | T35G D12A -  T35G D12A - | 24.06 *  24.06 * | 0 | . | Pzase | Cytosolic |
| Rv3795 embB 354D>A 0.0001 0.0122 | 3 | 0 | LN180 R  LN3756 R  MDRDM260 R | A392C V131G -  T403G T135P -  T403G T135P - | N. A.  0.05 *  0.05 * | 1 | 0.1002 | Cell Wall  formation / Ethambutol | Cytosolic |
|  |  |  | ME1473 R  TBV5000 R MDRDM627 R CSV11678 R SLM056 R LE486 R SLM036 R LE492 R LN2358 R TBV5362 R | A100C Y34D -  - - -  - - - G185A P62L -  - - - T35G D12A - A190C Y64D - T35G D12A - A545C L182W -  - - - | 53.59  100  100  N. A.  100  24.06 * N. A.  24.06 * N. A.  100 |  |  |  |  |

| Rv1908c katG 315S>T 0.002 0 | 22 | 13 | TBDM425 R MDRDM1098 R CSV10399 R SLM040 R SLM088 R SLM063 R MDRDM2491 R LN180 R SLM100 R MDRDM260 R SLM060 R LN3756 R LEI_79M S MDRMA203 S TBV4952 S MDRMA1565 S MDRMA701 S LNI2900LJ S MDRMA2019 S LE13 S LNI763LJ S TBDM2489 S MDRDM827 S TBDM2444 S LN1856 S | C145T D49N - C145T D49N - A280G F94L - T170C H57R -  - - - A416G V139A -  A392C V131G -  - - T-11C T403G T135P -  - - T-11C T403G T135P - | 0.12 *  0.12 *  55.18  N. A. * N. A.  N. A.  N. A. A. Exp.  0.05 * A. Exp.  0.05 * | 15 | 0.0147 | peroxidase | Cytosolic |
| --- | --- | --- | --- | --- | --- | --- | --- | --- | --- |
| Rv2853 PE_PG 180R>G 0.0033 0.0033  RS48 | 23 | 24 | CSV4644 R  TBV5365 R SLM063 R SLM040 R ME1473 R TBV5000 R SLM100 R TBDM425 R SLM056 R TBV5362 R SLM088 R MDRDM627 R LN180 R CSV10399 R CSV11678 R MDRDM2491 R LE492 R LN2358 R CSV4519 R MDRDM1098 R SLM036 R LE486 R SLM060 R CSV383 S LN1856 S | T152C H51R -  - - - A416G V139A - T170C H57R - A100C Y34D -  - - -  - - T-11C C145T D49N -  - - -  - - -  - - -  - - - A392C V131G - A280G F94L - G185A P62L -  T35G D12A - A545C L182W -  - - - C145T D49N - A190C Y64D - T35G D12A -  - - T-11C | 0.02 *  100  N. A.  N. A. *  53.59  100  A. Exp.  0.12 *  100  100  N. A.  100  N. A.  55.18  N. A.  24.06 * N. A.  100  0.12 * N. A.  24.06 * A. Exp. | 17 | 0.2498 | avoiding inmune system | Secreted |

|  |  |  | LN1100 S LNI2900LJ S TBDM2444 S MDRMA2260 S TBDM1506 S LE103 S LN55 S TBV4768 S MDRDM827 S MDRMA1565 S MDRMA701 S LN3584 S CSV9577 S TBDM2489 S LEI371M S TBV4952 S LE76 S LNI763LJ S MDRMA203 S TBV4766 S MDRMA2019 S MDRMA863 S |  |  |  |  |  |  |
| --- | --- | --- | --- | --- | --- | --- | --- | --- | --- |
| Rv1313c Rv1313 433L>R 0.0044 0.0044 c | 19 | 17 | ME1473 R  SLM060 R LN3756 R TBDM425 R LE492 R CSV11678 R MDRDM2491 R SLM040 R CSV10399 R SLM056 R MDRDM627 R CSV4519 R CSV5769 R LN2358 R MDRDM260 R LE486 R SLM100 R MDRDM1098 R SLM036 R LE13 S TBV4768 S LN55 S TBDM1506 S MDRMA2019 S MDRMA701 S TBDM2189 S LEI_63LJ S LN2978 S | A100C Y34D -  - - T-11C T403G T135P - C145T D49N - T35G D12A - G185A P62L -  T170C H57R - A280G F94L -  - - -  - - -  - - - C232A G78C - A545C L182W - T403G T135P - T35G D12A -  - - T-11C C145T D49N - A190C Y64D - | 53.59  A. Exp.  0.05 *  0.12 *  24.06 * N. A.  N. A. *  55.18  100  100  100  18.12 * N. A.  0.05 *  24.06 * A. Exp.  0.12 *  N. A. | 11 | 0.4407 | Transposase | Cytosolic |

|  |  |  | LN3584 S LEI_79M S LN1100 S LNI_3672LJ S CSV383 S LNI_3695LJ S MDRMA1565 S LNI317LJ S |  |  |  |  |  |  |
| --- | --- | --- | --- | --- | --- | --- | --- | --- | --- |
| Rv2505c fadD35 202Y>H 0.0086 0.034 | 2 | 0 | LN180 R  SLM063 R | A392C V131G -  A416G V139A - | N. A.  N. A. | 2 | 0.034 | fatty acid  biosynthesis | #N/A |
| Rv0280 PPE3 337S>P 0.0093 0.0093 | 22 | 24 | LN2358 R  SLM100 R LE486 R ME1473 R CSV4519 R MDRDM2491 R SLM088 R SLM040 R TBV5000 R SLM060 R SLM063 R CSV11678 R SLM036 R SLM056 R MDRDM627 R TBV5362 R TBV5365 R CSV10399 R LE492 R MDRDM1098 R TBDM425 R CSV4644 R LN3584 S LE103 S LNI2900LJ S MDRDM827 S CSV9577 S MDRMA1565 S MDRMA2019 S TBV4766 S LE76 S MDRMA863 S TBDM2489 S TBV4952 S TBDM2444 S MDRMA2260 S LN1100 S TBV4768 S LN55 S LN1856 S | A545C L182W -  - - T-11C T35G D12A - A100C Y34D -  - - -  - - - T170C H57R -  - - -  - - T-11C A416G V139A - G185A P62L - A190C Y64D -  - - -  - - -  - - -  - - - A280G F94L - T35G D12A - C145T D49N - C145T D49N - T152C H51R - | N. A.  A. Exp.  24.06 *  53.59  100  N. A.  N. A. *  100  A. Exp. N. A. N. A. N. A.  100  100  100  100  55.18  24.06 *  0.12 *  0.12 *  0.02 * | 16 | 0.3602 | avoiding inmune system | Secreted |

|  |  |  | LEI371M S TBDM1506 S CSV383 S MDRMA203 S MDRMA701 S LNI763LJ S |  |  |  |  |  |  |
| --- | --- | --- | --- | --- | --- | --- | --- | --- | --- |
| Rv3236c Rv3236 300V>I 0.0099 0.034 c | 2 | 0 | LN3756 R  MDRDM260 R | T403G T135P -  T403G T135P - | 0.05 *  0.05 * | 0 | . | cation  transporter | Transmembra  ne |
| PE_PG  Rv0278c RS3 807R>G 0.0106 0.0106 | 18 | 17 | TBV5365 R  MDRDM1098 R CSV4519 R LN3756 R MDRDM627 R LE486 R TBDM425 R SLM060 R MDRDM260 R CSV5769 R SLM056 R TBV5000 R LN2358 R CSV4644 R CSV11678 R SLM036 R ME1473 R CSV10399 R MDRMA2260 S CSV3611 S TBDM2489 S LE103 S LN1856 S MDRDM827 S LEI371M S MDRMA2019 S MDRMA203 S LNI2900LJ S TBDM2444 S MDRMA2441 S LN3584 S MDRMA701 S TBDM2189 S CSV9577 S LNI763LJ S | - - -  C145T D49N -  - - - T403G T135P -  - - - T35G D12A - C145T D49N -  - - T-11C T403G T135P - C232A G78C -  - - -  - - - A545C L182W - T152C H51R - G185A P62L - A190C Y64D - A100C Y34D - A280G F94L - | 100  0.12 *  100  0.05 *  100  24.06 *  0.12 * A. Exp.  0.05 *  18.12 *  100  100  N. A.  0.02 * N. A.  N. A.  53.59  55.18 | 11 | 0.4407 | avoiding inmune system | Secreted |
| Rv1556 Rv1556 164L>V 0.0122 0.034 | 2 | 0 | MDRDM1098 R  TBDM425 R | C145T D49N -  C145T D49N - | 0.12 *  0.12 * | 0 | . | Transciption  factor | Cytosolic |
| Rv1630 rpsA 432M>T 0.0122 0.034 | 2 | 0 | MDRDM1098 R  TBDM425 R | C145T D49N -  C145T D49N - | 0.12 *  0.12 * | 0 | . | RpsA | Cytosolic |
| Rv2718c nrdR 95A>V 0.0122 0.0122 | 3 | 0 | LN2358 R  MDRDM1098 R  TBDM425 R | A545C L182W -  C145T D49N -  C145T D49N - | N. A.  0.12 *  0.12 * | 1 | 0.1002 | Transcriptional  repressor  (Negative | Cytosolic |

| Rv2948c fadD22 2R>W 0.0122 0.034 | 2 | 0 | TBDM425 R  MDRDM1098 R | C145T D49N -  C145T D49N - | 0.12 *  0.12 * | 0 | . | fatty acid  biosynthesis | Cytosolic |
| --- | --- | --- | --- | --- | --- | --- | --- | --- | --- |
| Rv3242c Rv3242 148R>W 0.0122 0.034 c | 2 | 0 | TBDM425 R  MDRDM1098 R | C145T D49N -  C145T D49N - | 0.12 *  0.12 * | 0 | . | uncharacterized | uncharacteriz  ed |
| Rv1396c PE_PG 66R>S 0.0123 0.0123  RS25 | 19 | 19 | SLM060 R  TBV5365 R MDRDM1098 R ME1473 R LE486 R LN2358 R SLM088 R MDRDM627 R SLM063 R CSV11678 R CSV4519 R SLM100 R CSV4644 R MDRDM2491 R CSV10399 R SLM040 R TBV5000 R TBDM425 R SLM036 R MDRMA863 S CSV383 S LN1856 S TBV4768 S LE76 S LNI763LJ S MDRDM827 S LN1100 S MDRMA1565 S TBDM2444 S MDRMA203 S TBDM2489 S TBV4952 S MDRMA2019 S LN55 S LNI2900LJ S MDRMA701 S TBDM1506 S TBV4766 S | - - T-11C  - - - C145T D49N - A100C Y34D - T35G D12A - A545C L182W -  - - -  - - - A416G V139A - G185A P62L -  - - -  - - T-11C T152C H51R -  A280G F94L - T170C H57R -  - - - C145T D49N - A190C Y64D - | A. Exp.  100  0.12 *  53.59  24.06 * N. A.  N. A.  100  N. A. N. A.  100  A. Exp.  0.02 *  55.18  N. A. *  100  0.12 * N. A. | 14 | 0.245 | avoiding inmune system | Secreted |
| Rv2052c Rv2052 429A>T 0.0126 0.034 c | 2 | 0 | MDRDM1098 R  TBDM425 R | C145T D49N -  C145T D49N - | 0.12 *  0.12 * | 0 | . | Hydrolase | Cytosolic |
|  |  |  | SLM100 R  MDRDM260 R MDRDM1098 R CSV4519 R MDRDM2491 R CSV4644 R | - - T-11C  T403G T135P - C145T D49N -  - - -  T152C H51R - | A. Exp.  0.05 *  0.12 *  100  0.02 * |  |  |  |  |

| Rv0532 PE_PG 227D>G 0.014 0.014  RS6 | 23 | 27 | TBDM425 R CSV5769 R CSV10399 R SLM040 R LN3756 R TBV5365 R CSV11678 R ME1473 R LE486 R SLM056 R LE492 R TBV5000 R SLM063 R SLM036 R LN2358 R SLM060 R SLM088 R TBDM2489 S CSV3611 S TBDM1506 S LN55 S CSV9577 S MDRMA203 S TBV4766 S MDRDM827 S MDRMA701 S LNI317LJ S MDRMA2441 S LN1856 S TBDM2444 S LE76 S LN1100 S TBV4768 S MDRMA1565 S MDRMA2019 S LNI763LJ S TBDM2189 S TBV4952 S LE103 S LEI371M S CSV383 S MDRMA863 S LN3584 S LNI2900LJ S | C145T D49N - C232A G78C - A280G F94L - T170C H57R - T403G T135P -  - - - G185A P62L - A100C Y34D - T35G D12A -  - - - T35G D12A -  - - - A416G V139A - A190C Y64D - A545C L182W -  - - T-11C  - - - | 0.12 *  18.12 *  55.18  N. A. *  0.05 *  100  N. A.  53.59  24.06 *  100  24.06 *  100  N. A. N. A. N. A.  A. Exp. N. A. | 14 | 0.8037 | avoiding inmune system | Secreted |
| --- | --- | --- | --- | --- | --- | --- | --- | --- | --- |
|  |  |  | LE486 R  SLM100 R SLM056 R SLM060 R LN2358 R SLM063 R | T35G D12A -  - - T-11C  - - -  - - T-11C A545C L182W - A416G V139A - | 24.06 *  A. Exp.  100  A. Exp. N. A. N. A. |  |  |  |  |

| Rv1753c PPE24 488N>T 0.0143 0.0143 | 17 | 16 | CSV4519 R CSV4644 R LE492 R TBV5365 R CSV10399 R MDRDM1098 R TBDM425 R SLM036 R MDRDM2491 R SLM040 R CSV11678 R MDRDM827 S CSV9577 S TBDM2444 S MDRMA863 S LN55 S TBDM1506 S LNI2900LJ S MDRMA2019 S CSV383 S MDRMA1565 S LN1100 S TBV4952 S MDRMA203 S LNI763LJ S LE103 S TBV4768 S | - - - T152C H51R - T35G D12A -  - - - A280G F94L - C145T D49N - C145T D49N - A190C Y64D -  T170C H57R - G185A P62L - | 100  0.02 *  24.06 *  100  55.18  0.12 *  0.12 * N. A.  N. A. * N. A. | 11 | 0.365 | avoiding inmune system | Secreted |
| --- | --- | --- | --- | --- | --- | --- | --- | --- | --- |
| Rv3511 PE_PG 396N>D 0.0146 0.0146  RS55 | 21 | 23 | SLM100 R  LN2358 R SLM056 R LN3756 R CSV4519 R SLM036 R TBV5365 R MDRDM260 R SLM060 R MDRDM2491 R SLM088 R MDRDM1098 R CSV10399 R CSV5769 R CSV4644 R CSV11678 R LE486 R TBDM425 R SLM040 R ME1473 R SLM063 R MDRMA203 S TBDM2489 S | - - T-11C  A545C L182W -  - - - T403G T135P -  - - - A190C Y64D -  - - - T403G T135P -  - - T-11C  - - - C145T D49N - A280G F94L - C232A G78C - T152C H51R - G185A P62L - T35G D12A - C145T D49N - T170C H57R - A100C Y34D - A416G V139A - | A. Exp.  N. A.  100  0.05 *  100  N. A.  100  0.05 * A. Exp.  N. A.  0.12 *  55.18  18.12 *  0.02 * N. A.  24.06 *  0.12 * N. A. *  53.59  N. A. | 13 | 0.6489 | avoiding inmune system | Secreted |

|  |  |  | LN3584 S MDRMA701 S MDRMA863 S MDRDM827 S LN55 S MDRMA1565 S CSV383 S CSV9577 S LNI763LJ S LN1856 S CSV3611 S LNI317LJ S TBDM2444 S LNI2900LJ S TBV4952 S LE103 S TBV4768 S MDRMA2441 S MDRMA2019 S LEI371M S TBDM1506 S |  |  |  |  |  |  |
| --- | --- | --- | --- | --- | --- | --- | --- | --- | --- |
| PE_PG  Rv3508 RS54 1180S>A 0.0189 0.0048 | 11 | 6 | SLM088 R  TBV5365 R LN3756 R SLM100 R SLM056 R SLM040 R SLM060 R MDRDM2491 R CSV4519 R CSV10399 R MDRDM260 R LE103 S MDRMA863 S TBDM1506 S LEI371M S TBDM2444 S CSV9577 S | - - -  - - - T403G T135P -  - - T-11C  - - - T170C H57R -  - - T-11C  - - - A280G F94L - T403G T135P - | N. A.  100  0.05 * A. Exp.  100  N. A. * A. Exp.  100  55.18  0.05 * | 8 | 0.0512 | avoiding inmune system | Secreted |
|  |  |  | ME1473 R  LE486 R TBV5365 R CSV10399 R LN3756 R SLM100 R SLM088 R CSV5769 R SLM060 R LE492 R TBV5362 R SLM056 R | A100C Y34D -  T35G D12A -  - - - A280G F94L - T403G T135P -  - - T-11C  - - - C232A G78C -  - - T-11C T35G D12A -  - - -  - - - | 53.59  24.06 *  100  55.18  0.05 * A. Exp.  N. A.  18.12 * A. Exp.  24.06 *  100  100 |  |  |  |  |

Rv0958 Rv0958 274S>P 0.0218 0.0218 26

| TBDM425 | R | C145T | D49N | - 0.12 | * |
| --- | --- | --- | --- | --- | --- |
| CSV4519 | R | - | - | - 100 |  |
| MDRDM2491 | R |  |  |  |  |
| SLM040 | R | T170C | H57R | - N. A. | * |
| SLM063 | R | A416G | V139A | - N. A. |  |
| SLM036 | R | A190C | Y64D | - N. A. |  |
| TBV5000 | R | - | - | - 100 |  |
| CSV11678 | R | G185A | P62L | - N. A. |  |
| LN2358 | R | A545C | L182W | - N. A. |  |
| MDRDM1098 | R | C145T | D49N | - 0.12 | * |
| MDRDM260 | R | T403G | T135P | - 0.05 | * |
| CSV4644 | R | T152C | H51R | - 0.02 | * |
| LN180 | R | A392C | V131G | - N. A. |  |
| MDRDM627 | R | - | - | - 100 |  |
| LN2978 | S |  | | | |
| TBDM_2699 | S |  |  |  |  |
| LN55 | S |  |  |  |  |
| MDRMA203 | S |  |  |  |  |
| LE103 | S |  |  |  |  |
| LE13 | S |  |  |  |  |
| MDRMA701 | S |  |  |  |  |
| TBDM1506 | S |  |  |  |  |
| MDRDM827 | S |  |  |  |  |
| MDRMA863 | S |  |  |  |  |
| LEI371M | S |  |  |  |  |
| TBV4766 | S |  |  |  |  |
| LNI_3588M | S |  |  |  |  |
| MDRMA2019 | S |  |  |  |  |
| TBDM2489 | S |  |  |  |  |
| LNI763LJ | S |  |  |  |  |
| LNI_3589LJ | S |  |  |  |  |
| TBDM2444 | S |  |  |  |  |
| LNI_3672LJ | S |  |  |  |  |
| LN1856 | S |  |  |  |  |
| LN3584 | S |  |  |  |  |
| LNI_3695LJ | S |  |  |  |  |
| TBDM_2717 | S |  |  |  |  |
| TBDM2189 | S |  |  |  |  |
| LN1100 | S |  |  |  |  |
| LEI_63LJ | S |  |  |  |  |
| MDRMA1565 | S |  |  |  |  |
| TBV4768 | S |  |  |  |  |
| CSV383 | S |  |  |  |  |
| LNI317LJ | S |  |  |  |  |
| TBV4952 | S |  |  |  |  |
| LEI_79M | S |  |  |  |  |
| MDRMA2260 | S |  |  |  |  |
| LNI2900LJ | S |  |  |  |  |
| CSV9577  LE76 | S  S |  |  |  |  |

36 17 0.9753 Quelatase Mg / DNA regulation

Cytosolic

| Rv2487c PE_PG 313G>R 0.0229 0.0122  RS42 | 3 | 0 | CSV5769 R  LN3756 R  MDRDM260 R | C232A G78C -  T403G T135P -  T403G T135P - | 18.12 *  0.05 *  0.05 * | 0 | . | avoiding inmune system | Secreted |
| --- | --- | --- | --- | --- | --- | --- | --- | --- | --- |
| Rv2777c Rv2777 112P>S 0.0229 0.034 c | 2 | 0 | LN180 R  SLM063 R | A392C V131G -  A416G V139A - | N. A.  N. A. | 2 | 0.034 | uncharacterized | uncharacteriz  ed |
| Rv3297 nei 111I>F 0.0229 0.034 | 2 | 0 | MDRDM1098 R  TBDM425 R | C145T D49N -  C145T D49N - | 0.12 *  0.12 * | 0 | . | endonuclease | Cytosolic |
| Rv2560 Rv2560 134N>S 0.0233 0.034 | 2 | 0 | LN3756 R  MDRDM260 R | T403G T135P -  T403G T135P - | 0.05 *  0.05 * | 0 | . | Uncharacterized | Transmembra  ne |
| Rv2935 ppsE 1463T>P 0.0273 0.0122 | 3 | 0 | MDRDM1098 R  TBDM425 R  LN2358 R | C145T D49N -  C145T D49N -  A545C L182W - | 0.12 *  0.12 *  N. A. | 1 | 0.1002 | fatty acid biosynthesis | Cytosolic |
| Rv1937 Rv1937 552A>G 0.0282 0.034 | 2 | 0 | MDRDM260 R  LN3756 R | T403G T135P -  T403G T135P - | 0.05 *  0.05 * | 0 | . | Oxidoreductase | Cytosolic |
| Rv2783c gpsI 96P>R 0.0282 0.034 | 2 | 0 | LN3756 R  MDRDM260 R | T403G T135P -  T403G T135P - | 0.05 *  0.05 * | 0 | . | RNA proccesing | #N/A |
| Rv3507 PE_PG 1170A>G 0.0282 0.034  RS53 | 2 | 0 | MDRDM260 R  LN3756 R | T403G T135P -  T403G T135P - | 0.05 *  0.05 * | 0 | . | avoiding inmune  system | Secreted |
| Rv3659  Rv3659c c 337D>A 0.0282 0.0122 | 3 | 0 | TBDM425 R  LN2358 R  MDRDM1098 R | C145T D49N -  A545C L182W -  C145T D49N - | 0.12 *  N. A.  0.12 * | 1 | 0.1002 | Transferase | Cytosolic |
| Rv2802c Rv2802 198R>W 0.0298 0.034 c | 2 | 0 | TBDM425 R  MDRDM1098 R | C145T D49N -  C145T D49N - | 0.12 *  0.12 * | 0 | . | uncharacterized | uncharacteriz  ed |
|  |  |  | CSV5769 R  TBV5000 R LN2358 R TBDM425 R SLM060 R LE492 R SLM088 R SLM063 R ME1473 R CSV4519 R LN180 R SLM040 R SLM036 R TBV5362 R MDRDM627 R MDRDM1098 R CSV11678 R SLM100 R CSV4644 R MDRDM260 R LE486 R MDRDM2491 R SLM056 R LN3756 R TBV5365 R CSV10399 R LN2978 S | C232A G78C -  - - - A545C L182W - C145T D49N -  - - T-11C T35G D12A -  - - - A416G V139A - A100C Y34D -  - - - A392C V131G - T170C H57R - A190C Y64D -  - - -  - - - C145T D49N - G185A P62L -  - - T-11C T152C H51R - T403G T135P - T35G D12A -  - - - T403G T135P -  - - - A280G F94L - | 18.12 *  100  N. A.  0.12 * A. Exp.  24.06 * N. A.  N. A.  53.59  100  N. A.  N. A. * N. A.  100  100  0.12 * N. A.  A. Exp.  0.02 *  0.05 *  24.06 *  100  0.05 *  100  55.18 |  |  |  |  |

| Rv0218 Rv0218 316C>R 0.0338 0.0338 | 26 | 37 | MDRMA701 S LE76 S LNI_3589LJ S LN1100 S TBV4768 S LNI_3695LJ S TBDM1506 S TBV4766 S MDRMA203 S CSV3611 S LEI371M S LN3584 S LN55 S TBDM2487 S TBDM_2717 S MDRMA1565 S MDRMA2019 S MDRMA2441 S LE13 S LE103 S MDRMA2260 S TBV4952 S MDRMA863 S CSV383 S LNI317LJ S LEI_410LJ S LNI_3668M S LNI_3672LJ S MDRDM827 S TBDM2189 S TBDM2444 S LNI763LJ S TBDM2489 S CSV9577 S LN1856 S LNI2900LJ S |  |  | 17 | 0.9878 | electron carrier activity | Transmembra ne |
| --- | --- | --- | --- | --- | --- | --- | --- | --- | --- |
|  |  |  | LE492 R  CSV10399 R TBV5000 R SLM088 R LN2358 R LN3756 R SLM100 R SLM040 R MDRDM2491 R TBDM425 R SLM036 R LE486 R MDRDM1098 R CSV5769 R | T35G D12A -  A280G F94L -  - - -  - - - A545C L182W - T403G T135P -  - - T-11C T170C H57R -  C145T D49N - A190C Y64D - T35G D12A - C145T D49N - C232A G78C - | 24.06 *  55.18  100  N. A. N. A.  0.05 * A. Exp.  N. A. *  0.12 * N. A.  24.06 *  0.12 *  18.12 * |  |  |  |  |

| Rv0545c pitA 49P>S 0.0338 0.0338 | 26 | 37 | SLM056 R CSV11678 R MDRDM260 R SLM063 R SLM060 R MDRDM627 R CSV4644 R TBV5365 R LN180 R ME1473 R TBV5362 R CSV4519 R LN1856 S LNI_3672LJ S LNI_3589LJ S LE13 S TBDM2444 S TBDM1506 S LEI371M S TBDM_2699 S LN2978 S TBDM2189 S MDRMA701 S LE103 S MDRMA203 S MDRMA863 S MDRMA2260 S MDRDM827 S CSV383 S LN55 S LN1100 S TBV4766 S MDRMA2082 S MDRMA2019 S LNI_3588M S LEI_63LJ S LE76 S LNI_3695LJ S TBDM_2717 S TBDM2489 S MDRMA1565 S CSV9577 S LN3584 S LNI2900LJ S LNI317LJ S LNI763LJ S LEI_79M S TBV4768 S TBV4952 S | - - - G185A P62L - T403G T135P - A416G V139A -  - - T-11C  - - - T152C H51R -  - - - A392C V131G - A100C Y34D -  - - -  - - - | 100  N. A.  0.05 * N. A.  A. Exp.  100  0.02 *  100  N. A.  53.59  100  100 | 17 | 0.9878 | P uptake (low affinity) | Transmembra ne |
| --- | --- | --- | --- | --- | --- | --- | --- | --- | --- |
|  |  |  | CSV4644 R | T152C H51R - | 0.02 * |  |  |  |  |

|  | | | | | | | TBDM425 | R | C145T | D49N | - | 0.12 | * |  | | | |
| --- | --- | --- | --- | --- | --- | --- | --- | --- | --- | --- | --- | --- | --- | --- | --- | --- | --- |
|  |  |  |  |  |  |  | LE486 | R | T35G | D12A | - | 24.06 | * |  |  |  |  |
|  |  |  |  |  |  |  | TBV5365 | R | - | - | - | 100 |  |  |  |  |  |
|  |  |  |  |  |  |  | ME1473 | R | A100C | Y34D | - | 53.59 |  |  |  |  |  |
|  |  |  |  |  |  |  | CSV4519 | R | - | - | - | 100 |  |  |  |  |  |
|  |  |  |  |  |  |  | SLM100 | R | - | - | T-11C | A. Exp. |  |  |  |  |  |
|  |  |  |  |  |  |  | LN180 | R | A392C | V131G | - | N. A. |  |  |  |  |  |
|  |  |  |  |  |  |  | TBV5362 | R | - | - | - | 100 |  |  |  |  |  |
|  |  |  |  |  |  |  | CSV5769 | R | C232A | G78C | - | 18.12 | * |  |  |  |  |
|  |  |  |  |  |  |  | SLM063 | R | A416G | V139A | - | N. A. |  |  |  |  |  |
|  |  |  |  |  |  |  | SLM060 | R | - | - | T-11C | A. Exp. |  |  |  |  |  |
|  |  |  |  |  |  |  | LE492 | R | T35G | D12A | - | 24.06 | * |  |  |  |  |
|  |  |  |  |  |  |  | SLM056 | R | - | - | - | 100 |  |  |  |  |  |
|  |  |  |  |  |  |  | SLM040 | R | T170C | H57R | - | N. A. | * |  |  |  |  |
|  |  |  |  |  |  |  | MDRDM1098 | R | C145T | D49N | - | 0.12 | * |  |  |  |  |
|  |  |  |  |  |  |  | SLM036 | R | A190C | Y64D | - | N. A. |  |  |  |  |  |
|  |  |  |  |  |  |  | MDRDM627 | R | - | - | - | 100 |  |  |  |  |  |
|  |  |  |  |  |  |  | CSV10399 | R | A280G | F94L | - | 55.18 |  |  |  |  |  |
|  |  |  |  |  |  |  | TBV5000 | R | - | - | - | 100 |  |  |  |  |  |
|  |  |  |  |  |  |  | MDRDM260 | R | T403G | T135P | - | 0.05 | * |  |  |  |  |
|  |  |  |  |  |  |  | MDRDM2491 | R |  |  |  |  |  |  |  |  |  |
|  |  |  |  |  |  |  | LN2358 | R | A545C | L182W | - | N. A. |  |  |  |  |  |
|  |  |  |  |  |  |  | CSV11678 | R | G185A | P62L | - | N. A. |  |  |  |  |  |
|  |  |  |  |  |  |  | LN3756 | R | T403G | T135P | - | 0.05 | * |  |  |  |  |
|  |  |  |  |  |  |  | SLM088 | R | - | - | - | N. A. |  |  |  |  |  |
|  |  |  |  |  |  |  | LEI_63LJ | S |  |  |  |  |  |  |  |  |  |
|  |  |  |  |  |  |  | TBDM2444 | S |  |  |  |  |  |  |  |  |  |
|  |  |  |  |  |  |  | MDRMA701 | S |  |  |  |  |  |  |  |  |  |
|  |  |  |  |  |  |  | LNI_3672LJ | S |  |  |  |  |  |  |  |  |  |
|  |  |  |  |  |  |  | LNI763LJ | S |  |  |  |  |  |  |  |  |  |
| Rv0785 | Rv0785 | 408C>F | 0.0338 | 0.0338 | 26 | 37 | TBDM_2699 | S |  |  |  |  |  | 17 | 0.9878 | Deshydrogenase | Cytosolic |
|  | | | | | | | MDRMA2082 | S |  | | | | | | | | |
|  |  |  |  |  |  |  | LNI_3588M | S |  |  |  |  |  |  |  |  |  |
|  |  |  |  |  |  |  | MDRMA2260 | S |  |  |  |  |  |  |  |  |  |
|  |  |  |  |  |  |  | LNI_3695LJ | S |  |  |  |  |  |  |  |  |  |
|  |  |  |  |  |  |  | LN55 | S |  |  |  |  |  |  |  |  |  |
|  |  |  |  |  |  |  | TBV4768 | S |  |  |  |  |  |  |  |  |  |
|  |  |  |  |  |  |  | TBDM_2717 | S |  |  |  |  |  |  |  |  |  |
|  |  |  |  |  |  |  | LEI371M | S |  |  |  |  |  |  |  |  |  |
|  |  |  |  |  |  |  | LN2978 | S |  |  |  |  |  |  |  |  |  |
|  |  |  |  |  |  |  | LNI317LJ | S |  |  |  |  |  |  |  |  |  |
|  |  |  |  |  |  |  | TBV4952 | S |  |  |  |  |  |  |  |  |  |
|  |  |  |  |  |  |  | TBDM2489 | S |  |  |  |  |  |  |  |  |  |
|  |  |  |  |  |  |  | TBDM1506 | S |  |  |  |  |  |  |  |  |  |
|  |  |  |  |  |  |  | CSV383 | S |  |  |  |  |  |  |  |  |  |
|  |  |  |  |  |  |  | MDRMA2019 | S |  |  |  |  |  |  |  |  |  |
|  |  |  |  |  |  |  | LE76 | S |  |  |  |  |  |  |  |  |  |
|  |  |  |  |  |  |  | MDRDM827 | S |  |  |  |  |  |  |  |  |  |
|  |  |  |  |  |  |  | MDRMA203 | S |  |  |  |  |  |  |  |  |  |
|  |  |  |  |  |  |  | LE13 | S |  |  |  |  |  |  |  |  |  |

|  |  |  | LN3584 S LN1100 S LNI2900LJ S LEI_79M S TBDM2189 S TBV4766 S LN1856 S MDRMA1565 S CSV9577 S LE103 S MDRMA863 S LNI_3589LJ S |  |  |  |  |  |  |
| --- | --- | --- | --- | --- | --- | --- | --- | --- | --- |
| Rv0938 Rv0938 344C>R 0.0338 0.0338 | 26 | 37 | CSV11678 R  SLM036 R MDRDM627 R TBV5362 R SLM063 R LE492 R TBDM425 R LN2358 R SLM056 R LN3756 R SLM100 R TBV5000 R CSV10399 R MDRDM1098 R ME1473 R CSV4644 R MDRDM260 R CSV4519 R TBV5365 R SLM060 R LE486 R SLM040 R SLM088 R MDRDM2491 R CSV5769 R LN180 R LE76 S LN1856 S LN2978 S LE103 S TBV4768 S LNI_3588M S MDRMA1565 S LEI_79M S CSV9577 S MDRMA701 S TBDM2189 S LEI371M S | G185A P62L -  A190C Y64D -  - - -  - - - A416G V139A - T35G D12A - C145T D49N - A545C L182W -  - - - T403G T135P -  - - T-11C  - - - A280G F94L - C145T D49N - A100C Y34D - T152C H51R - T403G T135P -  - - -  - - -  - - T-11C T35G D12A - T170C H57R -  - - -  C232A G78C - A392C V131G - | N. A.  N. A.  100  100  N. A.  24.06 *  0.12 * N. A.  100  0.05 * A. Exp.  100  55.18  0.12 *  53.59  0.02 *  0.05 *  100  100  A. Exp.  24.06 * N. A. * N. A.  18.12 * N. A. | 17 | 0.9878 | DNA replication  (NHJ protein) | Cytosolic |

|  |  |  | LN1100 S MDRMA2082 S LNI763LJ S LNI_3589LJ S MDRMA863 S LEI_63LJ S TBV4952 S MDRDM827 S LN55 S CSV383 S MDRMA2019 S LE13 S TBDM1506 S TBDM_2717 S LNI2900LJ S MDRMA2260 S TBDM2489 S LN3584 S LNI_3672LJ S TBDM2444 S LNI_3695LJ S MDRMA203 S TBDM_2699 S LNI317LJ S TBV4766 S |  |  |  |  |  |  |
| --- | --- | --- | --- | --- | --- | --- | --- | --- | --- |
|  |  |  | LN3756 R  CSV11678 R CSV4519 R SLM036 R ME1473 R MDRDM1098 R CSV5769 R MDRDM627 R TBV5365 R CSV4644 R SLM060 R LN180 R SLM088 R TBV5000 R MDRDM260 R SLM100 R LE486 R SLM063 R TBV5362 R SLM056 R TBDM425 R CSV10399 R MDRDM2491 R LE492 R SLM040 R | T403G T135P -  G185A P62L -  - - - A190C Y64D - A100C Y34D - C145T D49N - C232A G78C -  - - -  - - - T152C H51R -  - - T-11C A392C V131G -  - - -  - - - T403G T135P -  - - T-11C T35G D12A - A416G V139A -  - - -  - - - C145T D49N - A280G F94L -  T35G D12A - T170C H57R - | 0.05 *  N. A.  100  N. A.  53.59  0.12 *  18.12 *  100  100  0.02 * A. Exp.  N. A. N. A.  100  0.05 * A. Exp.  24.06 * N. A.  100  100  0.12 *  55.18  24.06 * N. A. * |  |  |  |  |

| Rv0974c accD2 51N>K 0.0338 0.0338 | 26 | 37 | LN2358 R LNI317LJ S TBDM1506 S MDRMA2019 S LN55 S MDRMA1565 S TBDM2189 S LN1856 S TBV4766 S MDRMA2082 S LNI763LJ S MDRMA203 S CSV383 S LEI_63LJ S TBV4768 S LNI2900LJ S TBDM_2717 S TBDM2444 S LN3584 S LN1100 S TBV4952 S TBDM_2699 S LE103 S CSV9577 S LNI_3672LJ S MDRMA863 S LE76 S  LE13 S LNI_3695LJ S MDRMA2260 S LNI_3588M S LEI371M S LN2978 S LEI_79M S MDRMA701 S MDRDM827 S LNI_3589LJ S TBDM2489 S | A545C L182W - | N. A. | 17 | 0.9878 | malonyl-CoA  biosynthesis | Cytosolic |
| --- | --- | --- | --- | --- | --- | --- | --- | --- | --- |
|  |  |  | SLM060 R  LE492 R SLM040 R CSV11678 R MDRDM2491 R MDRDM260 R SLM063 R TBV5362 R CSV10399 R SLM088 R SLM100 R MDRDM627 R | - - T-11C  T35G D12A - T170C H57R - G185A P62L -  T403G T135P - A416G V139A -  - - - A280G F94L -  - - -  - - T-11C  - - - | A. Exp.  24.06 * N. A. * N. A.  0.05 * N. A.  100  55.18  N. A. A. Exp.  100 |  |  |  |  |

Cytochrome

|  | | | | | | | LN180 | R | A392C | V131G | - N. A. |  | | |
| --- | --- | --- | --- | --- | --- | --- | --- | --- | --- | --- | --- | --- | --- | --- |
|  |  |  |  |  |  |  | TBV5000 | R | - | - | - 100 |  |  |  |
|  |  |  |  |  |  |  | TBV5365 | R | - | - | - 100 |  |  |  |
|  |  |  |  |  |  |  | SLM056 | R | - | - | - 100 |  |  |  |
|  |  |  |  |  |  |  | ME1473 | R | A100C | Y34D | - 53.59 |  |  |  |
|  |  |  |  |  |  |  | MDRDM1098 | R | C145T | D49N | - 0.12 | * |  |  |
|  |  |  |  |  |  |  | TBDM425 | R | C145T | D49N | - 0.12 | * |  |  |
|  |  |  |  |  |  |  | LN3756 | R | T403G | T135P | - 0.05 | * |  |  |
|  |  |  |  |  |  |  | CSV4644 | R | T152C | H51R | - 0.02 | * |  |  |
|  |  |  |  |  |  |  | CSV4519 | R | - | - | - 100 |  |  |  |
|  |  |  |  |  |  |  | LN2358 | R | A545C | L182W | - N. A. |  |  |  |
|  |  |  |  |  |  |  | SLM036 | R | A190C | Y64D | - N. A. |  |  |  |
|  |  |  |  |  |  |  | CSV5769 | R | C232A | G78C | - 18.12 | * |  |  |
|  |  |  |  |  |  |  | LE486 | R | T35G | D12A | - 24.06 | * |  |  |
|  |  |  |  |  |  |  | LN2978 | S |  |  |  |  |  |  |
|  |  |  |  |  |  |  | TBV4768 | S |  |  |  |  |  |  |
|  |  |  |  |  |  |  | MDRMA2260 | S |  |  |  |  |  |  |
|  |  |  |  |  |  |  | TBDM_2699 | S |  |  |  |  |  |  |
|  |  |  |  |  |  |  | TBV4952 | S |  |  |  |  |  |  |
| Rv1394c | cyp132 | 135R>L | 0.0338 | 0.0338 | 26 | 37 | TBDM2489 | S |  |  |  |  | 17 | 0.9878 |
|  | | | | | | | CSV9577 | S |  | | | | | |
|  |  |  |  |  |  |  | LE103 | S |  |  |  |  |  |  |
|  |  |  |  |  |  |  | MDRDM827 | S |  |  |  |  |  |  |
|  |  |  |  |  |  |  | LNI2900LJ | S |  |  |  |  |  |  |
|  |  |  |  |  |  |  | CSV383 | S |  |  |  |  |  |  |
|  |  |  |  |  |  |  | LEI371M | S |  |  |  |  |  |  |
|  |  |  |  |  |  |  | LNI_3588M | S |  |  |  |  |  |  |
|  |  |  |  |  |  |  | LN3584 | S |  |  |  |  |  |  |
|  |  |  |  |  |  |  | LNI_3589LJ | S |  |  |  |  |  |  |
|  |  |  |  |  |  |  | MDRMA701 | S |  |  |  |  |  |  |
|  |  |  |  |  |  |  | TBDM2189 | S |  |  |  |  |  |  |
|  |  |  |  |  |  |  | LEI_79M | S |  |  |  |  |  |  |
|  |  |  |  |  |  |  | MDRMA2019 | S |  |  |  |  |  |  |
|  |  |  |  |  |  |  | MDRMA863 | S |  |  |  |  |  |  |
|  |  |  |  |  |  |  | LN1100 | S |  |  |  |  |  |  |
|  |  |  |  |  |  |  | LE76 | S |  |  |  |  |  |  |
|  |  |  |  |  |  |  | LN1856 | S |  |  |  |  |  |  |
|  |  |  |  |  |  |  | TBV4766 | S |  |  |  |  |  |  |
|  |  |  |  |  |  |  | LNI_3695LJ | S |  |  |  |  |  |  |
|  |  |  |  |  |  |  | LE13 | S |  |  |  |  |  |  |
|  |  |  |  |  |  |  | LNI317LJ | S |  |  |  |  |  |  |
|  |  |  |  |  |  |  | TBDM1506 | S |  |  |  |  |  |  |
|  |  |  |  |  |  |  | MDRMA1565 | S |  |  |  |  |  |  |
|  |  |  |  |  |  |  | MDRMA2082 | S |  |  |  |  |  |  |
|  |  |  |  |  |  |  | MDRMA203 | S |  |  |  |  |  |  |
|  |  |  |  |  |  |  | LEI_63LJ | S |  |  |  |  |  |  |
|  |  |  |  |  |  |  | LNI_3672LJ | S |  |  |  |  |  |  |
|  |  |  |  |  |  |  | TBDM2444 | S |  |  |  |  |  |  |
|  |  |  |  |  |  |  | LNI763LJ | S |  |  |  |  |  |  |
|  |  |  |  |  |  |  | LN55 | S |  |  |  |  |  |  |

P450 -

|  |  |  | TBDM_2717 S |  |  |  |  |  |  |
| --- | --- | --- | --- | --- | --- | --- | --- | --- | --- |
| Rv1449c tkt 18Y>D 0.0338 0.0338 | 26 | 37 | LN3756 R  CSV4519 R CSV5769 R LN2358 R MDRDM260 R SLM036 R SLM040 R SLM088 R CSV11678 R LN180 R MDRDM627 R SLM063 R SLM060 R CSV10399 R TBDM425 R TBV5365 R ME1473 R LE486 R LE492 R SLM056 R TBV5362 R MDRDM2491 R MDRDM1098 R CSV4644 R SLM100 R TBV5000 R MDRMA863 S LEI_63LJ S LNI_3672LJ S LE13 S MDRMA203 S LNI_3588M S TBDM_2699 S LNI763LJ S MDRMA2260 S LN1856 S LN1100 S TBDM1506 S LNI2900LJ S LN55 S CSV9577 S LN2978 S TBV4766 S TBDM2489 S TBDM2444 S LEI_79M S TBV4768 S LEI371M S LE103 S | T403G T135P -  - - - C232A G78C - A545C L182W - T403G T135P - A190C Y64D - T170C H57R -  - - - G185A P62L - A392C V131G -  - - - A416G V139A -  - - T-11C A280G F94L - C145T D49N -  - - - A100C Y34D - T35G D12A - T35G D12A -  - - -  - - -  C145T D49N - T152C H51R -  - - T-11C  - - - | 0.05 *  100  18.12 * N. A.  0.05 * N. A.  N. A. * N. A.  N. A. N. A.  100  N. A. A. Exp.  55.18  0.12 *  100  53.59  24.06 *  24.06 *  100  100  0.12 *  0.02 * A. Exp.  100 | 17 | 0.9878 | Transferase | Cytosolic |

|  |  |  | LNI317LJ S MDRMA2019 S LE76 S MDRMA2082 S CSV383 S MDRMA701 S TBDM2189 S MDRMA1565 S LNI_3589LJ S MDRDM827 S LNI_3695LJ S LN3584 S TBDM_2717 S TBV4952 S |  |  |  |  |  |  |
| --- | --- | --- | --- | --- | --- | --- | --- | --- | --- |
| Rv1604 impA 124P>Q 0.0338 0.0338 | 26 | 37 | MDRDM2491 R  SLM063 R TBDM425 R LE486 R CSV4644 R TBV5365 R SLM056 R SLM036 R CSV5769 R SLM060 R MDRDM260 R LE492 R SLM100 R CSV10399 R MDRDM1098 R CSV11678 R LN180 R SLM040 R SLM088 R TBV5000 R TBV5362 R ME1473 R CSV4519 R LN3756 R LN2358 R MDRDM627 R TBDM2444 S LEI371M S TBDM2189 S LN1856 S MDRMA203 S LNI317LJ S LEI_79M S CSV383 S TBV4768 S TBV4766 S | A416G V139A - C145T D49N - T35G D12A - T152C H51R -  - - -  - - - A190C Y64D - C232A G78C -  - - T-11C T403G T135P - T35G D12A -  - - T-11C A280G F94L - C145T D49N - G185A P62L - A392C V131G - T170C H57R -  - - -  - - -  - - - A100C Y34D -  - - - T403G T135P - A545C L182W -  - - - | N. A.  0.12 *  24.06 *  0.02 *  100  100  N. A.  18.12 * A. Exp.  0.05 *  24.06 * A. Exp.  55.18  0.12 * N. A.  N. A.  N. A. * N. A.  100  100  53.59  100  0.05 * N. A.  100 | 17 | 0.9878 | Sulfur  Metabolism | #N/A |

|  |  |  | TBDM2489 S TBDM_2699 S MDRMA2260 S LNI_3589LJ S MDRDM827 S TBDM_2717 S LN1100 S MDRMA701 S MDRMA2019 S LN3584 S LE103 S MDRMA863 S CSV9577 S TBV4952 S LNI_3672LJ S LNI763LJ S LNI_3695LJ S MDRMA2082 S LN55 S LNI_3588M S LE13 S LN2978 S LE76 S LEI_63LJ S MDRMA1565 S TBDM1506 S LNI2900LJ S |  |  |  |  |  |  |
| --- | --- | --- | --- | --- | --- | --- | --- | --- | --- |
|  |  |  | ME1473 R  LN2358 R CSV4644 R SLM100 R LN180 R MDRDM260 R CSV5769 R TBV5362 R TBV5000 R CSV10399 R SLM060 R TBV5365 R SLM063 R CSV11678 R LE492 R LN3756 R LE486 R SLM036 R CSV4519 R SLM056 R MDRDM1098 R SLM088 R MDRDM627 R | A100C Y34D -  A545C L182W - T152C H51R -  - - T-11C A392C V131G - T403G T135P - C232A G78C -  - - -  - - - A280G F94L -  - - T-11C  - - - A416G V139A - G185A P62L - T35G D12A - T403G T135P - T35G D12A - A190C Y64D -  - - -  - - - C145T D49N -  - - -  - - - | 53.59  N. A.  0.02 * A. Exp.  N. A.  0.05 *  18.12 *  100  100  55.18  A. Exp.  100  N. A. N. A.  24.06 *  0.05 *  24.06 * N. A.  100  100  0.12 * N. A.  100 |  |  |  |  |

| Rv1644 tsnR 232L>P 0.0338 0.0338 | 26 | 37 | SLM040 R MDRDM2491 R TBDM425 R MDRDM827 S LN1100 S TBV4768 S LE103 S MDRMA2019 S MDRMA203 S LNI_3672LJ S LE13 S LN55 S TBDM2444 S LNI_3588M S TBV4766 S TBV4952 S LNI_3695LJ S LEI_63LJ S CSV383 S LEI_79M S LEI371M S MDRMA2082 S TBDM1506 S LE76 S LNI763LJ S LN2978 S TBDM_2717 S LNI2900LJ S MDRMA701 S MDRMA1565 S TBDM2189 S MDRMA2260 S LNI_3589LJ S LN3584 S LN1856 S TBDM2489 S LNI317LJ S TBDM_2699 S MDRMA863 S CSV9577 S | T170C H57R - C145T D49N - | N. A. *  0.12 * | 17 | 0.9878 | 23S rRNA methyl transferase | Cytosolic |
| --- | --- | --- | --- | --- | --- | --- | --- | --- | --- |
|  |  |  | TBV5365 R  CSV4519 R CSV10399 R SLM060 R MDRDM2491 R LN180 R LN2358 R ME1473 R TBDM425 R MDRDM627 R | - - -  - - - A280G F94L -  - - T-11C  A392C V131G - A545C L182W - A100C Y34D - C145T D49N -  - - - | 100  100  55.18  A. Exp.  N. A. N. A.  53.59  0.12 *  100 |  |  |  |  |

|  | | | | | | | MDRDM1098 | R | C145T | D49N | - | 0.12 | * |  | | | |
| --- | --- | --- | --- | --- | --- | --- | --- | --- | --- | --- | --- | --- | --- | --- | --- | --- | --- |
|  |  |  |  |  |  |  | SLM063 | R | A416G | V139A | - | N. A. |  |  |  |  |  |
|  |  |  |  |  |  |  | CSV11678 | R | G185A | P62L | - | N. A. |  |  |  |  |  |
|  |  |  |  |  |  |  | SLM100 | R | - | - | T-11C | A. Exp. |  |  |  |  |  |
|  |  |  |  |  |  |  | SLM088 | R | - | - | - | N. A. |  |  |  |  |  |
|  |  |  |  |  |  |  | LE492 | R | T35G | D12A | - | 24.06 | * |  |  |  |  |
|  |  |  |  |  |  |  | LN3756 | R | T403G | T135P | - | 0.05 | * |  |  |  |  |
|  |  |  |  |  |  |  | SLM056 | R | - | - | - | 100 |  |  |  |  |  |
|  |  |  |  |  |  |  | SLM040 | R | T170C | H57R | - | N. A. | * |  |  |  |  |
|  |  |  |  |  |  |  | LE486 | R | T35G | D12A | - | 24.06 | * |  |  |  |  |
|  |  |  |  |  |  |  | MDRDM260 | R | T403G | T135P | - | 0.05 | * |  |  |  |  |
|  |  |  |  |  |  |  | SLM036 | R | A190C | Y64D | - | N. A. |  |  |  |  |  |
|  |  |  |  |  |  |  | CSV5769 | R | C232A | G78C | - | 18.12 | * |  |  |  |  |
|  |  |  |  |  |  |  | TBV5000 | R | - | - | - | 100 |  |  |  |  |  |
|  |  |  |  |  |  |  | CSV4644 | R | T152C | H51R | - | 0.02 | * |  |  |  |  |
|  |  |  |  |  |  |  | TBV5362 | R | - | - | - | 100 |  |  |  |  |  |
|  |  |  |  |  |  |  | LEI371M | S |  |  |  |  |  |  |  |  |  |
|  |  |  |  |  |  |  | LNI_3588M | S |  |  |  |  |  |  |  |  |  |
|  |  |  |  |  |  |  | MDRMA701 | S |  |  |  |  |  |  |  |  |  |
|  |  |  |  |  |  |  | TBDM1506 | S |  |  |  |  |  |  |  |  |  |
|  |  |  |  |  |  |  | LNI_3589LJ | S |  |  |  |  |  |  |  | cAMP/cGMP |  |
| Rv1900c | lipJ | 204I>M | 0.0338 | 0.0338 | 26 | 37 | MDRMA203 | S |  |  |  |  |  | 17 | 0.9878 | biosynthetic | Cytosolic |
|  | | | | | | | LE13 | S |  |  |  |  |  |  |  | process |  |
|  |  |  |  |  |  |  | TBV4768 | S |  | | | | | | | | |
|  |  |  |  |  |  |  | TBV4952 | S |  |  |  |  |  |  |  |  |  |
|  |  |  |  |  |  |  | TBV4766 | S |  |  |  |  |  |  |  |  |  |
|  |  |  |  |  |  |  | CSV9577 | S |  |  |  |  |  |  |  |  |  |
|  |  |  |  |  |  |  | LEI_63LJ | S |  |  |  |  |  |  |  |  |  |
|  |  |  |  |  |  |  | TBDM_2699 | S |  |  |  |  |  |  |  |  |  |
|  |  |  |  |  |  |  | TBDM2489 | S |  |  |  |  |  |  |  |  |  |
|  |  |  |  |  |  |  | MDRMA863 | S |  |  |  |  |  |  |  |  |  |
|  |  |  |  |  |  |  | MDRMA2019 | S |  |  |  |  |  |  |  |  |  |
|  |  |  |  |  |  |  | TBDM2189 | S |  |  |  |  |  |  |  |  |  |
|  |  |  |  |  |  |  | LNI2900LJ | S |  |  |  |  |  |  |  |  |  |
|  |  |  |  |  |  |  | LN3584 | S |  |  |  |  |  |  |  |  |  |
|  |  |  |  |  |  |  | CSV383 | S |  |  |  |  |  |  |  |  |  |
|  |  |  |  |  |  |  | LNI317LJ | S |  |  |  |  |  |  |  |  |  |
|  |  |  |  |  |  |  | MDRMA2260 | S |  |  |  |  |  |  |  |  |  |
|  |  |  |  |  |  |  | LN1856 | S |  |  |  |  |  |  |  |  |  |
|  |  |  |  |  |  |  | LN2978 | S |  |  |  |  |  |  |  |  |  |
|  |  |  |  |  |  |  | MDRDM827 | S |  |  |  |  |  |  |  |  |  |
|  |  |  |  |  |  |  | LEI_79M | S |  |  |  |  |  |  |  |  |  |
|  |  |  |  |  |  |  | TBDM_2717 | S |  |  |  |  |  |  |  |  |  |
|  |  |  |  |  |  |  | LE103 | S |  |  |  |  |  |  |  |  |  |
|  |  |  |  |  |  |  | LN1100 | S |  |  |  |  |  |  |  |  |  |
|  |  |  |  |  |  |  | MDRMA1565 | S |  |  |  |  |  |  |  |  |  |
|  |  |  |  |  |  |  | TBDM2444 | S |  |  |  |  |  |  |  |  |  |
|  |  |  |  |  |  |  | LNI_3695LJ | S |  |  |  |  |  |  |  |  |  |
|  |  |  |  |  |  |  | MDRMA2082 | S |  |  |  |  |  |  |  |  |  |
|  |  |  |  |  |  |  | LNI763LJ | S |  |  |  |  |  |  |  |  |  |

|  |  |  | LN55 S LE76 S LNI_3672LJ S |  |  |  |  |  |  |
| --- | --- | --- | --- | --- | --- | --- | --- | --- | --- |
| Rv2072c cobL 205L>P 0.0338 0.0338 | 26 | 37 | LE486 R  TBV5362 R CSV11678 R TBV5000 R LE492 R TBV5365 R SLM060 R SLM100 R CSV4519 R CSV4644 R CSV10399 R SLM036 R MDRDM627 R MDRDM2491 R MDRDM1098 R SLM088 R TBDM425 R SLM040 R LN3756 R CSV5769 R MDRDM260 R SLM056 R ME1473 R LN2358 R LN180 R SLM063 R LNI_3672LJ S TBDM2489 S TBDM2444 S LE103 S MDRMA2260 S CSV383 S LNI2900LJ S LNI_3695LJ S LNI763LJ S LN1856 S TBDM_2717 S MDRMA701 S LEI_63LJ S LN55 S LN3584 S LNI_3589LJ S MDRMA203 S CSV9577 S LN1100 S TBV4768 S LE76 S | T35G D12A -  - - - G185A P62L -  - - - T35G D12A -  - - -  - - T-11C  - - T-11C  - - - T152C H51R - A280G F94L - A190C Y64D -  - - -  C145T D49N -  - - - C145T D49N - T170C H57R - T403G T135P - C232A G78C - T403G T135P -  - - - A100C Y34D - A545C L182W - A392C V131G - A416G V139A - | 24.06 *  100  N. A.  100  24.06 *  100  A. Exp. A. Exp.  100  0.02 *  55.18  N. A.  100  0.12 * N. A.  0.12 * N. A. *  0.05 *  18.12 *  0.05 *  100  53.59  N. A. N. A. N. A. | 17 | 0.9878 | Cobalamin Biosynthetic Process | Cytosolic |

|  |  |  | LNI_3588M S TBDM2189 S LEI_79M S LNI317LJ S LE13 S MDRDM827 S MDRMA2082 S TBV4952 S LEI371M S LN2978 S TBDM1506 S MDRMA1565 S TBV4766 S TBDM_2699 S MDRMA863 S MDRMA2019 S |  |  |  |  |  |  |
| --- | --- | --- | --- | --- | --- | --- | --- | --- | --- |
| Rv2236c cobD 79S>C 0.0338 0.0338 | 26 | 37 | LE492 R  ME1473 R SLM088 R SLM040 R CSV10399 R TBV5365 R CSV4519 R SLM100 R TBDM425 R SLM060 R LN3756 R CSV11678 R TBV5000 R SLM036 R SLM056 R CSV5769 R MDRDM260 R MDRDM1098 R LN2358 R LN180 R LE486 R MDRDM627 R MDRDM2491 R TBV5362 R CSV4644 R SLM063 R LNI_3672LJ S LNI763LJ S TBDM2444 S TBV4766 S CSV383 S TBDM_2717 S TBDM2189 S LNI_3695LJ S | T35G D12A -  A100C Y34D -  - - - T170C H57R - A280G F94L -  - - -  - - -  - - T-11C C145T D49N -  - - T-11C T403G T135P - G185A P62L -  - - - A190C Y64D -  - - - C232A G78C - T403G T135P - C145T D49N - A545C L182W - A392C V131G - T35G D12A -  - - -  - - - T152C H51R - A416G V139A - | 24.06 *  53.59  N. A.  N. A. *  55.18  100  100  A. Exp.  0.12 * A. Exp.  0.05 * N. A.  100  N. A.  100  18.12 *  0.05 *  0.12 * N. A.  N. A.  24.06 *  100  100  0.02 * N. A. | 17 | 0.9878 | Cobalamin Biosynthetic Process | Cytosolic |

|  |  |  | TBV4768 S MDRMA2019 S LN1856 S MDRMA1565 S LNI2900LJ S LN55 S MDRMA2260 S LEI371M S LNI_3589LJ S LNI317LJ S MDRMA863 S TBV4952 S MDRMA701 S LNI_3588M S TBDM2489 S LEI_79M S LN3584 S MDRDM827 S CSV9577 S LN2978 S LN1100 S LE103 S MDRMA2082 S LE76 S TBDM_2699 S LEI_63LJ S LE13 S TBDM1506 S MDRMA203 S |  |  |  |  |  |  |
| --- | --- | --- | --- | --- | --- | --- | --- | --- | --- |
|  |  |  | SLM036 R  LE486 R SLM100 R SLM063 R CSV4644 R TBV5365 R LN3756 R LE492 R MDRDM260 R SLM060 R CSV11678 R LN2358 R LN180 R CSV5769 R MDRDM627 R CSV10399 R TBV5362 R MDRDM2491 R SLM040 R TBV5000 R ME1473 R | A190C Y64D -  T35G D12A -  - - T-11C A416G V139A - T152C H51R -  - - - T403G T135P - T35G D12A - T403G T135P -  - - T-11C G185A P62L - A545C L182W - A392C V131G - C232A G78C -  - - - A280G F94L -  - - -  T170C H57R -  - - - A100C Y34D - | N. A.  24.06 * A. Exp.  N. A.  0.02 *  100  0.05 *  24.06 *  0.05 * A. Exp.  N. A.  N. A. N. A.  18.12 *  100  55.18  100  N. A. *  100  53.59 |  |  |  |  |

| Rv2379c mbtF 589E>D 0.0338 0.0338 | 26 | 37 | SLM056 R MDRDM1098 R CSV4519 R SLM088 R TBDM425 R LNI317LJ S LNI2900LJ S MDRMA203 S MDRMA2019 S LNI_3588M S MDRMA863 S LNI_3695LJ S LEI_63LJ S LEI371M S MDRMA2082 S LNI_3589LJ S LN1856 S TBV4952 S CSV9577 S LN1100 S TBDM_2717 S LE103 S LN3584 S LE76 S MDRMA1565 S TBV4766 S TBDM_2699 S LE13 S MDRDM827 S LEI_79M S LN55 S MDRMA2260 S CSV383 S TBDM2444 S LN2978 S LNI_3672LJ S TBV4768 S LNI763LJ S TBDM2189 S MDRMA701 S TBDM2489 S TBDM1506 S | - - - C145T D49N -  - - -  - - - C145T D49N - | 100  0.12 *  100  N. A.  0.12 * | 17 | 0.9878 | #N/A | #N/A |
| --- | --- | --- | --- | --- | --- | --- | --- | --- | --- |
|  |  |  | SLM088 R  SLM056 R LE492 R CSV4644 R LE486 R SLM063 R CSV11678 R SLM036 R | - - -  - - - T35G D12A - T152C H51R - T35G D12A - A416G V139A - G185A P62L - A190C Y64D - | N. A.  100  24.06 *  0.02 *  24.06 * N. A.  N. A. N. A. |  |  |  |  |

|  | | | | | | | LN3756 | R | T403G | T135P | - | 0.05 | * |  | | | |
| --- | --- | --- | --- | --- | --- | --- | --- | --- | --- | --- | --- | --- | --- | --- | --- | --- | --- |
|  |  |  |  |  |  |  | SLM060 | R | - | - | T-11C | A. Exp. |  |  |  |  |  |
|  |  |  |  |  |  |  | CSV4519 | R | - | - | - | 100 |  |  |  |  |  |
|  |  |  |  |  |  |  | LN180 | R | A392C | V131G | - | N. A. |  |  |  |  |  |
|  |  |  |  |  |  |  | MDRDM260 | R | T403G | T135P | - | 0.05 | * |  |  |  |  |
|  |  |  |  |  |  |  | SLM100 | R | - | - | T-11C | A. Exp. |  |  |  |  |  |
|  |  |  |  |  |  |  | MDRDM2491 | R |  |  |  |  |  |  |  |  |  |
|  |  |  |  |  |  |  | TBDM425 | R | C145T | D49N | - | 0.12 | * |  |  |  |  |
|  |  |  |  |  |  |  | MDRDM1098 | R | C145T | D49N | - | 0.12 | * |  |  |  |  |
|  |  |  |  |  |  |  | CSV5769 | R | C232A | G78C | - | 18.12 | * |  |  |  |  |
|  |  |  |  |  |  |  | MDRDM627 | R | - | - | - | 100 |  |  |  |  |  |
|  |  |  |  |  |  |  | TBV5362 | R | - | - | - | 100 |  |  |  |  |  |
|  |  |  |  |  |  |  | ME1473 | R | A100C | Y34D | - | 53.59 |  |  |  |  |  |
|  |  |  |  |  |  |  | TBV5365 | R | - | - | - | 100 |  |  |  |  |  |
|  |  |  |  |  |  |  | LN2358 | R | A545C | L182W | - | N. A. |  |  |  |  |  |
|  |  |  |  |  |  |  | CSV10399 | R | A280G | F94L | - | 55.18 |  |  |  |  |  |
|  |  |  |  |  |  |  | SLM040 | R | T170C | H57R | - | N. A. | * |  |  |  |  |
|  |  |  |  |  |  |  | TBV5000 | R | - | - | - | 100 |  |  |  |  |  |
|  |  |  |  |  |  |  | MDRMA701 | S |  |  |  |  |  |  |  |  |  |
|  |  |  |  |  |  |  | LNI763LJ | S |  |  |  |  |  |  |  |  |  |
|  |  |  |  |  |  |  | MDRMA1565 | S |  |  |  |  |  |  |  |  |  |
|  |  |  |  |  |  |  | MDRMA2260 | S |  |  |  |  |  |  |  |  |  |
|  |  |  |  |  |  |  | TBDM2189 | S |  |  |  |  |  |  |  |  |  |
| Rv2398c | cysW | 141G>A | 0.0338 | 0.0338 | 26 | 37 | TBV4952 | S |  |  |  |  |  | 17 | 0.9878 | ABC transporter | Membrane |
|  | | | | | | | CSV383 | S |  | | | | | | | | |
|  |  |  |  |  |  |  | LN55 | S |  |  |  |  |  |  |  |  |  |
|  |  |  |  |  |  |  | LN2978 | S |  |  |  |  |  |  |  |  |  |
|  |  |  |  |  |  |  | MDRDM827 | S |  |  |  |  |  |  |  |  |  |
|  |  |  |  |  |  |  | LEI_79M | S |  |  |  |  |  |  |  |  |  |
|  |  |  |  |  |  |  | MDRMA203 | S |  |  |  |  |  |  |  |  |  |
|  |  |  |  |  |  |  | LNI_3672LJ | S |  |  |  |  |  |  |  |  |  |
|  |  |  |  |  |  |  | MDRMA2019 | S |  |  |  |  |  |  |  |  |  |
|  |  |  |  |  |  |  | TBV4766 | S |  |  |  |  |  |  |  |  |  |
|  |  |  |  |  |  |  | LE103 | S |  |  |  |  |  |  |  |  |  |
|  |  |  |  |  |  |  | TBDM2489 | S |  |  |  |  |  |  |  |  |  |
|  |  |  |  |  |  |  | TBDM2444 | S |  |  |  |  |  |  |  |  |  |
|  |  |  |  |  |  |  | MDRMA863 | S |  |  |  |  |  |  |  |  |  |
|  |  |  |  |  |  |  | LNI_3695LJ | S |  |  |  |  |  |  |  |  |  |
|  |  |  |  |  |  |  | LE76 | S |  |  |  |  |  |  |  |  |  |
|  |  |  |  |  |  |  | TBDM_2717 | S |  |  |  |  |  |  |  |  |  |
|  |  |  |  |  |  |  | LNI_3589LJ | S |  |  |  |  |  |  |  |  |  |
|  |  |  |  |  |  |  | CSV9577 | S |  |  |  |  |  |  |  |  |  |
|  |  |  |  |  |  |  | LE13 | S |  |  |  |  |  |  |  |  |  |
|  |  |  |  |  |  |  | LEI371M | S |  |  |  |  |  |  |  |  |  |
|  |  |  |  |  |  |  | TBV4768 | S |  |  |  |  |  |  |  |  |  |
|  |  |  |  |  |  |  | LNI_3588M | S |  |  |  |  |  |  |  |  |  |
|  |  |  |  |  |  |  | LNI317LJ | S |  |  |  |  |  |  |  |  |  |
|  |  |  |  |  |  |  | MDRMA2082 | S |  |  |  |  |  |  |  |  |  |
|  |  |  |  |  |  |  | TBDM_2699 | S |  |  |  |  |  |  |  |  |  |
|  |  |  |  |  |  |  | LEI_63LJ | S |  |  |  |  |  |  |  |  |  |

|  |  |  | LN1100 S LNI2900LJ S LN1856 S LN3584 S TBDM1506 S |  |  |  |  |  |  |
| --- | --- | --- | --- | --- | --- | --- | --- | --- | --- |
| Rv2691 ceoB 117T>A 0.0338 0.0338 | 26 | 37 | TBDM425 R  LE486 R LN3756 R CSV5769 R SLM060 R CSV11678 R LN2358 R TBV5000 R TBV5362 R MDRDM627 R SLM063 R CSV10399 R LE492 R SLM056 R ME1473 R CSV4519 R SLM036 R SLM100 R CSV4644 R MDRDM2491 R MDRDM260 R SLM040 R SLM088 R LN180 R MDRDM1098 R TBV5365 R CSV9577 S LE13 S MDRMA2019 S MDRMA203 S LEI_63LJ S MDRMA701 S LN1856 S TBV4952 S TBDM_2699 S LNI_3695LJ S LNI_3672LJ S LE103 S TBDM1506 S TBDM2444 S TBV4766 S TBDM2489 S MDRMA2260 S MDRMA863 S LN1100 S | C145T D49N -  T35G D12A - T403G T135P - C232A G78C -  - - T-11C G185A P62L - A545C L182W -  - - -  - - -  - - - A416G V139A - A280G F94L - T35G D12A -  - - - A100C Y34D -  - - - A190C Y64D -  - - T-11C T152C H51R -  T403G T135P - T170C H57R -  - - -  A392C V131G - C145T D49N -  - - - | 0.12 *  24.06 *  0.05 *  18.12 * A. Exp.  N. A. N. A.  100  100  100  N. A.  55.18  24.06 *  100  53.59  100  N. A. A. Exp.  0.02 *  0.05 * N. A. * N. A.  N. A.  0.12 *  100 | 17 | 0.9878 | Transmembrane Transporter Potassium | Transmembra ne |

|  |  |  | LN2978 S LN55 S MDRMA1565 S LNI317LJ S TBDM2189 S LNI_3589LJ S CSV383 S LE76 S LNI2900LJ S LNI_3588M S MDRMA2082 S TBV4768 S LNI763LJ S LN3584 S LEI371M S LEI_79M S MDRDM827 S TBDM_2717 S |  |  |  |  |  |  |
| --- | --- | --- | --- | --- | --- | --- | --- | --- | --- |
| Rv2729  Rv2729c 202A>E 0.0338 0.0338 | 26 | 37 | MDRDM2491 R  TBDM425 R TBV5000 R CSV4519 R CSV5769 R SLM036 R LN2358 R CSV4644 R SLM100 R LN180 R TBV5365 R ME1473 R SLM060 R CSV11678 R MDRDM627 R LN3756 R TBV5362 R MDRDM260 R MDRDM1098 R SLM040 R CSV10399 R LE492 R LE486 R SLM063 R SLM088 R SLM056 R TBV4766 S TBDM_2717 S MDRDM827 S TBDM2489 S LNI_3672LJ S TBV4768 S | C145T D49N -  - - -  - - - C232A G78C - A190C Y64D - A545C L182W - T152C H51R -  - - T-11C A392C V131G -  - - - A100C Y34D -  - - T-11C G185A P62L -  - - - T403G T135P -  - - - T403G T135P - C145T D49N - T170C H57R - A280G F94L - T35G D12A - T35G D12A - A416G V139A -  - - -  - - - | 0.12 *  100  100  18.12 * N. A.  N. A.  0.02 * A. Exp.  N. A.  100  53.59  A. Exp. N. A.  100  0.05 *  100  0.05 *  0.12 * N. A. *  55.18  24.06 *  24.06 * N. A.  N. A.  100 | 17 | 0.9878 | uncharacterized | Transmembra |

c ne

| MDRMA2082  TBV4952  LN2978  LN1100  LNI2900LJ MDRMA863  TBDM_2699  LEI371M TBDM2189  MDRMA701  LNI_3695LJ TBDM2444  LN1856  MDRMA1565  MDRMA2260  LEI_63LJ LN3584  LNI763LJ LE76  LNI317LJ CSV383  CSV9577  LNI_3589LJ MDRMA2019  LN55  LE13  MDRMA203  LE103  LNI_3588M TBDM1506 | S S S S S S S S S S S S S S S S S S S S S S S S S S S S S S |  | | | | |
| --- | --- | --- | --- | --- | --- | --- |
| LEI_79M | S |  |  |  |  |  |
| TBV5000 | R | - | - | - | 100 |  |
| LE486  MDRDM2491  SLM040 | R  R R | T35G  T170C | D12A  H57R | -  - | 24.06  N. A. | *  * |
| CSV4519 | R | - | - | - | 100 |  |
| LN3756 | R | T403G | T135P | - | 0.05 | * |
| LN180 | R | A392C | V131G | - | N. A. |  |
| MDRDM260 | R | T403G | T135P | - | 0.05 | * |
| CSV4644 | R | T152C | H51R | - | 0.02 | * |
| SLM060 | R | - | - | T-11C | A. Exp. |  |
| TBV5362 | R | - | - | - | 100 |  |
| CSV10399 | R | A280G | F94L | - | 55.18 |  |
| CSV5769 | R | C232A | G78C | - | 18.12 | * |
| SLM100 | R | - | - | T-11C | A. Exp. |  |
| LN2358 | R | A545C | L182W | - | N. A. |  |
| MDRDM1098 | R | C145T | D49N | - | 0.12 | * |
| TBDM425 | R | C145T | D49N | - | 0.12 | * |
| ME1473 | R | A100C | Y34D | - | 53.59 |  |
| TBV5365 | R | - | - | - | 100 |  |

| Rv3383c idsB 132V>G 0.0338 0.0338 | 26 | 37 | SLM063 R SLM036 R SLM088 R CSV11678 R MDRDM627 R SLM056 R LE492 R MDRMA2082 S LNI_3695LJ S LEI_79M S TBV4766 S LNI2900LJ S TBDM2489 S TBV4768 S LE76 S LEI371M S TBDM_2699 S MDRMA203 S TBDM1506 S LE13 S MDRMA701 S LN2978 S LEI_63LJ S LNI317LJ S MDRDM827 S MDRMA2019 S MDRMA1565 S LNI_3589LJ S TBDM_2717 S TBV4952 S TBDM2444 S LN1856 S LNI763LJ S MDRMA863 S LNI_3588M S CSV383 S LE103 S LNI_3672LJ S LN3584 S MDRMA2260 S CSV9577 S TBDM2189 S LN55 S LN1100 S | A416G V139A - A190C Y64D -  - - - G185A P62L -  - - -  - - - T35G D12A - | N. A. N. A. N. A. N. A.  100  100  24.06 * | 17 | 0.9878 | Isoprenoid Biosynthetic Process | Cytosolic |
| --- | --- | --- | --- | --- | --- | --- | --- | --- | --- |
|  |  |  | SLM040 R  MDRDM627 R MDRDM2491 R TBV5362 R CSV4519 R TBV5365 R | T170C H57R -  - - -  - - -  - - -  - - - | N. A. *  100  100  100  100 |  |  |  |  |

Rv3468c

|  | | | | | MDRDM260 | R | T403G | T135P | - | 0.05 | * |  | | | |
| --- | --- | --- | --- | --- | --- | --- | --- | --- | --- | --- | --- | --- | --- | --- | --- |
|  |  |  |  |  | CSV11678 | R | G185A | P62L | - | N. A. |  |  |  |  |  |
|  |  |  |  |  | LN2358 | R | A545C | L182W | - | N. A. |  |  |  |  |  |
|  |  |  |  |  | SLM088 | R | - | - | - | N. A. |  |  |  |  |  |
|  |  |  |  |  | ME1473 | R | A100C | Y34D | - | 53.59 |  |  |  |  |  |
|  |  |  |  |  | TBDM425 | R | C145T | D49N | - | 0.12 | * |  |  |  |  |
|  |  |  |  |  | SLM036 | R | A190C | Y64D | - | N. A. |  |  |  |  |  |
|  |  |  |  |  | LN3756 | R | T403G | T135P | - | 0.05 | * |  |  |  |  |
|  |  |  |  |  | CSV4644 | R | T152C | H51R | - | 0.02 | * |  |  |  |  |
|  |  |  |  |  | SLM056 | R | - | - | - | 100 |  |  |  |  |  |
|  |  |  |  |  | LN180 | R | A392C | V131G | - | N. A. |  |  |  |  |  |
|  |  |  |  |  | SLM100 | R | - | - | T-11C | A. Exp. |  |  |  |  |  |
|  |  |  |  |  | LE486 | R | T35G | D12A | - | 24.06 | * |  |  |  |  |
|  |  |  |  |  | SLM063 | R | A416G | V139A | - | N. A. |  |  |  |  |  |
|  |  |  |  |  | CSV5769 | R | C232A | G78C | - | 18.12 | * |  |  |  |  |
|  |  |  |  |  | LE492 | R | T35G | D12A | - | 24.06 | * |  |  |  |  |
|  |  |  |  |  | CSV10399 | R | A280G | F94L | - | 55.18 |  |  |  |  |  |
|  |  |  |  |  | TBV5000 | R | - | - | - | 100 |  |  |  |  |  |
|  |  |  |  |  | MDRDM1098 | R | C145T | D49N | - | 0.12 | * |  |  |  |  |
|  |  |  |  |  | SLM060 | R | - | - | T-11C | A. Exp. |  |  |  |  |  |
|  |  |  |  |  | LE76 | S |  |  |  |  |  |  |  |  |  |
|  |  |  |  |  | MDRMA2260 | S |  |  |  |  |  |  |  |  |  |
|  |  |  |  |  | MDRMA1565 | S |  |  |  |  |  |  |  |  |  |
|  |  |  |  |  | LEI_79M | S |  |  |  |  |  |  |  |  |  |
|  |  |  |  |  | LN2978 | S |  |  |  |  |  |  |  |  |  |
| 62I>V | 0.0338 | 0.0338 | 26 | 37 | LE13 | S |  |  |  |  |  | 17 | 0.9878 | Deshydrogenase | Cytosolic |
|  |  |  |  |  | LNI317LJ | S |  | | | | | | | | |
|  |  |  |  |  | CSV9577 | S |  |  |  |  |  |  |  |  |  |
|  |  |  |  |  | LN55 | S |  |  |  |  |  |  |  |  |  |
|  |  |  |  |  | MDRMA863 | S |  |  |  |  |  |  |  |  |  |
|  |  |  |  |  | LNI_3695LJ | S |  |  |  |  |  |  |  |  |  |
|  |  |  |  |  | MDRMA2082 | S |  |  |  |  |  |  |  |  |  |
|  |  |  |  |  | TBDM1506 | S |  |  |  |  |  |  |  |  |  |
|  |  |  |  |  | TBV4768 | S |  |  |  |  |  |  |  |  |  |
|  |  |  |  |  | LE103 | S |  |  |  |  |  |  |  |  |  |
|  |  |  |  |  | MDRMA2019 | S |  |  |  |  |  |  |  |  |  |
|  |  |  |  |  | TBDM2189 | S |  |  |  |  |  |  |  |  |  |
|  |  |  |  |  | TBDM_2699 | S |  |  |  |  |  |  |  |  |  |
|  |  |  |  |  | TBDM2489 | S |  |  |  |  |  |  |  |  |  |
|  |  |  |  |  | MDRDM827 | S |  |  |  |  |  |  |  |  |  |
|  |  |  |  |  | LNI_3589LJ | S |  |  |  |  |  |  |  |  |  |
|  |  |  |  |  | LN3584 | S |  |  |  |  |  |  |  |  |  |
|  |  |  |  |  | TBV4952 | S |  |  |  |  |  |  |  |  |  |
|  |  |  |  |  | MDRMA701 | S |  |  |  |  |  |  |  |  |  |
|  |  |  |  |  | LNI_3672LJ | S |  |  |  |  |  |  |  |  |  |
|  |  |  |  |  | MDRMA203 | S |  |  |  |  |  |  |  |  |  |
|  |  |  |  |  | TBV4766 | S |  |  |  |  |  |  |  |  |  |
|  |  |  |  |  | CSV383 | S |  |  |  |  |  |  |  |  |  |
|  |  |  |  |  | TBDM_2717 | S |  |  |  |  |  |  |  |  |  |
|  |  |  |  |  | LEI_63LJ | S |  |  |  |  |  |  |  |  |  |

Rv3468 c

|  |  |  | LN1100 S TBDM2444 S LNI_3588M S LNI2900LJ S LEI371M S LNI763LJ S LN1856 S |  |  |  |  |  |  |
| --- | --- | --- | --- | --- | --- | --- | --- | --- | --- |
| Rv3521 Rv3521 295N>D 0.0338 0.0338 | 26 | 37 | SLM100 R  CSV4519 R MDRDM1098 R LE486 R TBV5365 R LE492 R CSV5769 R TBDM425 R CSV11678 R SLM060 R CSV10399 R MDRDM627 R SLM063 R TBV5362 R ME1473 R TBV5000 R MDRDM260 R SLM036 R LN180 R SLM056 R LN3756 R CSV4644 R LN2358 R MDRDM2491 R SLM088 R SLM040 R LE13 S MDRMA701 S TBV4766 S TBDM_2717 S LNI_3695LJ S TBDM1506 S MDRMA2260 S TBDM_2699 S TBDM2189 S MDRMA1565 S LNI_3672LJ S MDRMA2019 S LNI763LJ S LE103 S LN2978 S LNI_3589LJ S LN55 S | - - T-11C  - - - C145T D49N - T35G D12A -  - - - T35G D12A - C232A G78C - C145T D49N - G185A P62L -  - - T-11C A280G F94L -  - - - A416G V139A -  - - - A100C Y34D -  - - - T403G T135P - A190C Y64D - A392C V131G -  - - - T403G T135P - T152C H51R - A545C L182W -  - - - T170C H57R - | A. Exp.  100  0.12 *  24.06 *  100  24.06 *  18.12 *  0.12 * N. A.  A. Exp.  55.18  100  N. A.  100  53.59  100  0.05 * N. A.  N. A.  100  0.05 *  0.02 * N. A.  N. A.  N. A. * | 17 | 0.9878 | uncharacterized | uncharacteriz ed |

|  |  |  | TBV4768 S MDRDM827 S CSV383 S LE76 S LNI2900LJ S LN3584 S TBDM2444 S LN1100 S LN1856 S LEI_63LJ S TBV4952 S TBDM2489 S LEI371M S LNI_3588M S MDRMA203 S MDRMA863 S CSV9577 S LEI_79M S MDRMA2082 S LNI317LJ S |  |  |  |  |  |  |
| --- | --- | --- | --- | --- | --- | --- | --- | --- | --- |
|  |  |  | LE486 R  SLM100 R TBDM425 R LN3756 R SLM036 R TBV5365 R SLM040 R LN2358 R TBV5362 R MDRDM627 R CSV4519 R SLM088 R SLM063 R ME1473 R CSV4644 R SLM056 R MDRDM260 R CSV11678 R SLM060 R LN180 R MDRDM2491 R MDRDM1098 R LE492 R CSV10399 R CSV5769 R TBV5000 R LNI763LJ S LNI2900LJ S CSV383 S TBDM_2717 S | T35G D12A -  - - T-11C C145T D49N - T403G T135P - A190C Y64D -  - - - T170C H57R - A545C L182W -  - - -  - - -  - - -  - - - A416G V139A - A100C Y34D - T152C H51R -  - - - T403G T135P - G185A P62L -  - - T-11C A392C V131G -  C145T D49N - T35G D12A - A280G F94L - C232A G78C -  - - - | 24.06 *  A. Exp.  0.12 *  0.05 * N. A.  100  N. A. * N. A.  100  100  100  N. A. N. A.  53.59  0.02 *  100  0.05 * N. A.  A. Exp. N. A.  0.12 *  24.06 *  55.18  18.12 *  100 |  |  |  |  |

| Rv3630 Rv3630 40A>T 0.0338 0.0338 | 26 | 37 | LNI_3589LJ S LEI_63LJ S LNI317LJ S LE13 S LN1100 S LNI_3672LJ S TBV4952 S TBDM2489 S LN3584 S MDRMA701 S LEI_79M S MDRMA2082 S TBDM_2699 S LEI371M S MDRMA1565 S LNI_3695LJ S LN2978 S LNI_3588M S MDRDM827 S TBV4768 S TBDM2444 S MDRMA2019 S LE76 S TBDM1506 S LN55 S LN1856 S MDRMA2260 S TBV4766 S CSV9577 S TBDM2189 S LE103 S MDRMA203 S MDRMA863 S |  |  | 17 | 0.9878 | Uncharacterized | Transmembra ne |
| --- | --- | --- | --- | --- | --- | --- | --- | --- | --- |
|  |  |  | TBV5362 R  SLM060 R SLM088 R SLM100 R CSV5769 R LE486 R SLM056 R TBV5365 R LN3756 R TBV5000 R SLM063 R MDRDM627 R LE492 R CSV11678 R LN180 R SLM036 R MDRDM1098 R | - - -  - - T-11C  - - -  - - T-11C C232A G78C - T35G D12A -  - - -  - - - T403G T135P -  - - - A416G V139A -  - - - T35G D12A - G185A P62L - A392C V131G - A190C Y64D - C145T D49N - | 100  A. Exp. N. A.  A. Exp.  18.12 *  24.06 *  100  100  0.05 *  100  N. A.  100  24.06 * N. A.  N. A. N. A.  0.12 * |  |  |  |  |

| Rv3731 ligC 313R>H 0.0338 0.0338 | 26 | 37 | CSV10399 R ME1473 R SLM040 R CSV4519 R CSV4644 R MDRDM260 R TBDM425 R LN2358 R MDRDM2491 R LN1100 S LE76 S LN2978 S TBDM_2717 S MDRMA203 S LN3584 S LNI763LJ S LNI_3589LJ S TBDM2444 S LNI_3672LJ S LEI_79M S MDRDM827 S LNI317LJ S TBDM_2699 S LE103 S LNI2900LJ S TBDM2489 S LN1856 S LE13 S CSV9577 S CSV383 S LNI_3695LJ S TBV4952 S LEI_63LJ S LNI_3588M S MDRMA1565 S TBDM2189 S MDRMA2260 S MDRMA863 S LEI371M S TBDM1506 S MDRMA701 S TBV4766 S MDRMA2019 S LN55 S TBV4768 S MDRMA2082 S | A280G F94L - A100C Y34D - T170C H57R -  - - - T152C H51R - T403G T135P - C145T D49N - A545C L182W - | 55.18  53.59  N. A. *  100  0.02 *  0.05 *  0.12 * N. A. | 17 | 0.9878 | DNA ligase C | Cytosolic |
| --- | --- | --- | --- | --- | --- | --- | --- | --- | --- |
| Rv0002 dnaN 307R>L 0.034 0.034 | 2 | 0 | LE486 R  LE492 R | T35G D12A -  T35G D12A - | 24.06 *  24.06 * | 0 | . | DNA pol Beta | Cytosolic |
| Rv0161 Rv0161 87E>K 0.034 0.034 | 2 | 0 | TBDM425 R  MDRDM1098 R | C145T D49N -  C145T D49N - | 0.12 *  0.12 * | 0 | . | Oxidoreductase | Cytosolic |

| Rv0231 fadE4 148M>T 0.034 0.034 | 2 | 0 | TBDM425 R  MDRDM1098 R | C145T D49N -  C145T D49N - | 0.12 *  0.12 * | 0 | . | Acyl-CoA  dehydrogenase | Cytosolic |
| --- | --- | --- | --- | --- | --- | --- | --- | --- | --- |
| TetR  Rv0273c family 167C>Y 0.034 0.034 | 4 | 0 | MDRDM1098 R  TBDM425 R TBDM425 R MDRDM1098 R | C145T D49N -  C145T D49N - C145T D49N - C145T D49N - | 0.12 *  0.12 *  0.12 *  0.12 * | 0 | . | Transciption factor | Cytosolic |
| Rv0329c MitM 142A>T 0.034 0.034 | 2 | 0 | MDRDM1098 R  TBDM425 R | C145T D49N -  C145T D49N - | 0.12 *  0.12 * | 0 | . | Metiltransferase | Cytosolic |
| Rv0389 purT 378E>* 0.034 0.034 | 2 | 0 | MDRDM260 R  LN3756 R | T403G T135P -  T403G T135P - | 0.05 *  0.05 * | 0 | . | Formyltransfera  se (pentose | Cytosolic |
| Rv0548c menB 122T>N 0.034 0.034 | 2 | 0 | MDRDM260 R  LN3756 R | T403G T135P -  T403G T135P - | 0.05 *  0.05 * | 0 | . | Quinol/quinone  Metabolism | Cytosolic |
| Rv0735 sigL 160H>Y 0.034 0.034 | 2 | 0 | SLM063 R  LN180 R | A416G V139A -  A392C V131G - | N. A.  N. A. | 2 | 0.034 | RNA pol Sigma  Factor | Cytosolic |
| Rv0787A Rv0787 71D>N 0.034 0.034  A | 2 | 0 | LN180 R  SLM063 R | A392C V131G -  A416G V139A - | N. A.  N. A. | 2 | 0.034 | Purine  Biosynthetic | Cytosolic |
| Rv0921 Rv0921 139G>R 0.034 0.034 | 2 | 0 | LN3756 R  MDRDM260 R | T403G T135P -  T403G T135P - | 0.05 *  0.05 * | 0 | . | Posible  Resolvase | Cytosolic |
| Rv0937c Rv0937 233F>S 0.034 0.034 c | 2 | 0 | LN3756 R  MDRDM260 R | T403G T135P -  T403G T135P - | 0.05 *  0.05 * | 0 | . | DNA  recombination | Cytosolic |
| Rv0994 moeA1 394T>A 0.034 0.034 | 2 | 0 | SLM063 R  LN180 R | A416G V139A -  A392C V131G - | N. A.  N. A. | 2 | 0.034 | Cofactor  Biosynthesis | Cytosolic |
| Rv1024 Rv1024 158P>T 0.034 0.034 | 2 | 0 | LE486 R  LE492 R | T35G D12A -  T35G D12A - | 24.06 *  24.06 * | 0 | . | Uncharacterized | Membrane |
| Rv1034c Rv1034 49G>A 0.034 0.034 c | 2 | 0 | TBDM425 R  MDRDM1098 R | C145T D49N -  C145T D49N - | 0.12 *  0.12 * | 0 | . | Transposase | Cytosolic |
| Rv1052 Rv1052 14G>S 0.034 0.034 | 2 | 0 | TBDM425 R  MDRDM1098 R | C145T D49N -  C145T D49N - | 0.12 *  0.12 * | 0 | . | Uncharacterized | uncharacteriz  ed |
| Rv1060 Rv1060 52A>V 0.034 0.034 | 2 | 0 | LN3756 R  MDRDM260 R | T403G T135P -  T403G T135P - | 0.05 *  0.05 * | 0 | . | Uncharacterized | uncharacteriz  ed |
| Rv1188 Rv1188 244I>V 0.034 0.034 | 2 | 0 | MDRDM1098 R  TBDM425 R | C145T D49N -  C145T D49N - | 0.12 *  0.12 * | 0 | . | Glutamate  Biosynthesis | Cell Wall |
| Rv1327c glgE 8T>A 0.034 0.034 | 2 | 0 | LN180 R  SLM063 R | A392C V131G -  A416G V139A - | N. A.  N. A. | 2 | 0.034 | Glycan  Biosynthesis | Cytosolic |
| Rv1334 Rv1334 42G>A 0.034 0.034 | 2 | 0 | MDRDM260 R  LN3756 R | T403G T135P -  T403G T135P - | 0.05 *  0.05 * | 0 | . | Aminoacid  Biosynthesis | Cytosolic |
| Rv1539 lspA 2P>S 0.034 0.034 | 2 | 0 | MDRDM1098 R  TBDM425 R | C145T D49N -  C145T D49N - | 0.12 *  0.12 * | 0 | . | avoiding inmune  system | Membrane |
| Rv1541c lprI 143D>A 0.034 0.034 | 2 | 0 | MDRDM260 R  LN3756 R | T403G T135P -  T403G T135P - | 0.05 *  0.05 * | 0 | . | avoiding inmune  system | Membrane |
| Rv1544 Rv1544 35P>R 0.034 0.034 | 2 | 0 | LN3756 R  MDRDM260 R | T403G T135P -  T403G T135P - | 0.05 *  0.05 * | 0 | . | avoiding inmune  system | Membrane |
| Rv1612 trpB 276V>I 0.034 0.034 | 2 | 0 | LE486 R  LE492 R | T35G D12A -  T35G D12A - | 24.06 *  24.06 * | 0 | . | Aminoacid  Biosynthesis | Cytosolic |
| Rv1653 argJ 72L>R 0.034 0.034 | 2 | 0 | LN3756 R  MDRDM260 R | T403G T135P -  T403G T135P - | 0.05 *  0.05 * | 0 | . | Aminoacid  Biosynthesis | Cytosolic |
| Rv1742 Rv1742 185Y>C 0.034 0.034 | 2 | 0 | LN180 R  SLM063 R | A392C V131G -  A416G V139A - | N. A.  N. A. | 2 | 0.034 | Uncharacterized | uncharacteriz  ed |
|  |  |  | LN3756 R | T403G T135P - | 0.05 * |  |  |  | uncharacteriz |

Rv1752 Rv1752

| 46G>E | 0.034 | 0.034 | 2 | 0 |
| --- | --- | --- | --- | --- |

0 . Uncharacterized

|  | | | | | | | MDRDM260 | R | T403G | T135P | - 0.05 | * | ed | | | |
| --- | --- | --- | --- | --- | --- | --- | --- | --- | --- | --- | --- | --- | --- | --- | --- | --- |
| Rv1865c | Rv1865  c | 208G>R | 0.034 | 0.034 | 2 | 0 | MDRDM1098  TBDM425 | R  R | C145T  C145T | D49N  D49N | - 0.12  - 0.12 | *  * | 0 | . | Probable Membrane  Deshydrogenase | |
| Rv1960c | Rv1960  c | 30S>T | 0.034 | 0.034 | 2 | 0 | MDRDM260  LN3756 | R  R | T403G  T403G | T135P  T135P | - 0.05  - 0.05 | *  * | 0 | . | Antitoxin Secreted | |
| Rv2036 | Rv2036 | 209A>V | 0.034 | 0.034 | 2 | 0 | MDRDM1098  TBDM425 | R  R | C145T  C145T | D49N  D49N | - 0.12  - 0.12 | *  * | 0 | . | DNA damage- Cytosolic inducible | |
| Rv2205c | Rv2205  c | 301Q>* | 0.034 | 0.034 | 2 | 0 | MDRDM260  LN3756 | R  R | T403G  T403G | T135P  T135P | - 0.05  - 0.05 | *  * | 0 | . | Uncharacterized Cell Wall | |
| Rv2210c | ilvE | 91V>M | 0.034 | 0.034 | 2 | 0 | MDRDM260  LN3756 | R  R | T403G  T403G | T135P  T135P | - 0.05  - 0.05 | *  * | 0 | . | Aminoacid Cytosolic  Biosynthesis | |
| Rv2317 | uspB | 265V>F | 0.034 | 0.034 | 2 | 0 | SLM063  LN180 | R  R | A416G  A392C | V139A  V131G | - N. A.  - N. A. |  | 2 | 0.034 | ABC transporter Membrane | |
| Rv2370c | Rv2370  c | 293Q>E | 0.034 | 0.034 | 2 | 0 | MDRDM260  LN3756 | R  R | T403G  T403G | T135P  T135P | - 0.05  - 0.05 | *  * | 0 | . Uncharacterized uncharacteriz ed | | |
| Rv2465c | Rv2465  c | 114M>I | 0.034 | 0.034 | 2 | 0 | TBDM425  MDRDM1098 | R  R | C145T  C145T | D49N  D49N | - 0.12  - 0.12 | *  * | 0 | . | Pentose  Pathway | Cytosolic  uncharacteriz ed  Cytosolic uncharacteriz  ed  uncharacteriz ed  Membrane uncharacteriz  ed |
| Rv2557 | Rv2557 | 128W>* | 0.034 | 0.034 | 2 | 0 | LN180  SLM063 | R  R | A392C  A416G | V131G  V139A | - N. A.  - N. A. |  | 2 | 0.034 | Uncharacterized |  |
| Rv2646 | Rv2646 | 316Y>D | 0.034 | 0.034 | 2 | 0 | TBV5362  TBV5000 | R  R | -  - | -  - | - 100  - 100 |  | 2 | 0.034 | Integrase |  |
| Rv2657c | Rv2657  c | 57R>G | 0.034 | 0.034 | 2 | 0 | TBDM425  MDRDM1098 | R  R | C145T  C145T | D49N  D49N | - 0.12  - 0.12 | *  * | 0 | . | Uncharacterized |  |
| Rv2675c | Rv2675  c | 40V>M | 0.034 | 0.034 | 2 | 0 | LN3756  MDRDM260 | R  R | T403G  T403G | T135P  T135P | - 0.05  - 0.05 | *  * | 0 | . | Uncharacterized |  |
| Rv2833c | ugpB | 214N>T | 0.034 | 0.034 | 2 | 0 | TBDM425  MDRDM1098 | R  R | C145T  C145T | D49N  D49N | - 0.12  - 0.12 | *  * | 0 | . | ABC transporter |  |
| Rv2857c | Rv2857  c | 164V>G | 0.034 | 0.034 | 2 | 0 | LN180  SLM063 | R  R | A392C  A416G | V131G  V139A | - N. A.  - N. A. |  | 2 | 0.034 | Uncharacterized |  |

Rv3039c echA17 244G>D 0.034 0.034 2

0 MDRDM260 R T403G T135P - 0.05 * LN3756 R T403G T135P - 0.05 *

0 . fatty acid biosynthesis

Cytosolic

Rv3049c Rv3049 c

57V>I 0.034 0.034 2 0

TBDM425 R C145T D49N - 0.12 * MDRDM1098 R C145T D49N - 0.12 *

0 . Oxidoreductase Cytosolic

Rv3094c Rv3094 c

325D>N 0.034 0.034 2

0 TBDM425 R C145T D49N - 0.12 * MDRDM1098 R C145T D49N - 0.12 *

0 . fatty acid biosynthesis

Cytosolic

Rv3158 nuoN 336V>I 0.034 0.034 2 0

MDRDM260 R T403G T135P - 0.05 * LN3756 R T403G T135P - 0.05 *

0 . NADH reductase Membrane

Rv3223c sigH 16G>R 0.034 0.034 2

0 MDRDM1098 R C145T D49N - 0.12 * TBDM425 R C145T D49N - 0.12 *

0 . RNA pol Sigma

Factor

Cytosolic

Rv3301c phoY1 5Y>S 0.034 0.034 2 0

|  |  |  |  |  |  |
| --- | --- | --- | --- | --- | --- |
|  |  |  |  |  |  |

MDRDM260 R T403G T135P - 0.05 * LN3756 R T403G T135P - 0.05 *

0 . P Uptake Membrane

| Rv3362c Rv3362 80V>A  c | | | 0.034 | 0.034 | 2 | 0 | SLM063 R A416G V139A - N. A. 2  LN180 R A392C V131G - N. A. | 0.034 Uncharacterized uncharacteriz ed | |
| --- | --- | --- | --- | --- | --- | --- | --- | --- | --- |
| Rv3393 | iunH | 31G>S | 0.034 | 0.034 | 2 | 0 | LN180 R A392C V131G - N. A. 2  SLM063 R A416G V139A - N. A. | 0.034 | Nucleoside Cytosolic  Hydrolase |
| Rv3410c | guaB3 | 36A>T | 0.034 | 0.034 | 2 | 0 | LN180 R A392C V131G - N. A. 2  SLM063 R A416G V139A - N. A. | 0.034 | Oxidoreductase Membrane |
| Rv3492c Rv3492 74T>M  c | | | 0.034 | 0.034 | 2 | 0 MDRDM1098 R C145T D49N - 0.12 * 0 . Mammalian cell Membrane  TBDM425 R C145T D49N - 0.12 * entry protein | | | |

| Rv3494c mce4F 537G>V 0.034 0.034 | 2 | 0 | LN3756 R  MDRDM260 R | T403G T135P -  T403G T135P - | 0.05 *  0.05 * | 0 | . | Mammalian cell  entry protein | Membrane |
| --- | --- | --- | --- | --- | --- | --- | --- | --- | --- |
| Rv3767c Rv3767 90F>C 0.034 0.034 c | 2 | 0 | LN180 R  SLM063 R | A392C V131G -  A416G V139A - | N. A.  N. A. | 2 | 0.034 | Metiltransferase | Cytosolic |
| Rv3883c mycP1 159E>G 0.034 0.034 | 2 | 0 | LN3756 R  MDRDM260 R | T403G T135P -  T403G T135P - | 0.05 *  0.05 * | 0 | . | Protease | Membrane |
| Rv2741 PE_PG 271S>G 0.0344 0.0344  RS47 | 21 | 25 | ME1473 R  SLM088 R CSV4644 R MDRDM2491 R CSV10399 R SLM040 R SLM060 R LN2358 R LE486 R TBDM425 R TBV5365 R SLM063 R LN3756 R CSV11678 R MDRDM627 R CSV4519 R SLM100 R CSV5769 R MDRDM1098 R MDRDM260 R SLM056 R CSV9577 S LN55 S MDRMA2441 S LN1100 S LEI371M S TBDM2189 S TBDM2489 S CSV3611 S LNI2900LJ S MDRDM827 S MDRMA2019 S LN3584 S TBV4952 S CSV383 S MDRMA863 S MDRMA701 S TBDM1506 S TBV4768 S TBDM2444 S MDRMA203 S LN1856 S LE103 S LNI763LJ S | A100C Y34D -  - - - T152C H51R -  A280G F94L - T170C H57R -  - - T-11C A545C L182W - T35G D12A - C145T D49N -  - - - A416G V139A - T403G T135P - G185A P62L -  - - -  - - -  - - T-11C C232A G78C - C145T D49N - T403G T135P -  - - - | 53.59  N. A.  0.02 *  55.18  N. A. * A. Exp.  N. A.  24.06 *  0.12 *  100  N. A.  0.05 * N. A.  100  100  A. Exp.  18.12 *  0.12 *  0.05 *  100 | 13 | 0.7789 | avoiding inmune system | Secreted |

|  |  |  | TBV4766 S  MDRMA1565 S |  |  |  |  |  |  |
| --- | --- | --- | --- | --- | --- | --- | --- | --- | --- |
| PE_PG  Rv0833 RS13 584S>G 0.0354 0.0354 | 17 | 18 | TBDM425 R  SLM056 R CSV5769 R CSV4519 R CSV10399 R SLM100 R SLM088 R LN3756 R CSV4644 R SLM040 R SLM060 R SLM063 R TBV5365 R MDRDM1098 R SLM036 R LN2358 R CSV11678 R CSV3611 S MDRMA203 S LE103 S LN1100 S LN1856 S TBDM2489 S LEI371M S CSV9577 S LNI2900LJ S TBV4952 S LE76 S MDRDM827 S LN3584 S TBDM1506 S TBDM2444 S LNI763LJ S MDRMA701 S MDRMA2441 S | C145T D49N -  - - - C232A G78C -  - - - A280G F94L -  - - T-11C  - - - T403G T135P - T152C H51R - T170C H57R -  - - T-11C A416G V139A -  - - - C145T D49N - A190C Y64D - A545C L182W - G185A P62L - | 0.12 *  100  18.12 *  100  55.18  A. Exp. N. A.  0.05 *  0.02 * N. A. * A. Exp.  N. A.  100  0.12 * N. A.  N. A. N. A. | 11 | 0.5178 | avoiding inmune system | Secreted |
| Rv0668 rpoC 1252V>M 0.0456 0.034 | 2 | 0 | TBV5362 R  TBV5000 R | - - -  - - - | 100  100 | 2 | 0.034 | RNA polymerase | Cytosolic |
